# Utilizing the subtractive proteomics approach to design ensemble vaccine against *Candida lusitaniae* for immune response stimulation; a bioinformatics study

**DOI:** 10.1371/journal.pone.0316264

**Published:** 2025-02-06

**Authors:** Habiba Naz, Rimsha Timotheous, Muhammad Farhan Sarwar, Tariq Nadeem, Mudassar Fareed Awan, Sajed Ali, Sophia Awais, Irfan Ahmed

**Affiliations:** 1 Department of Biotechnology, Knowledge Unit of Science (KUSC), University of Management and Technology Sialkot, Sialkot, Pakistan; 2 Centre of Excellence in Molecular Biology, University of the Punjab, Lahore, Pakistan; 3 Faculty of Pharmacy, IBADAT International University Islamabad, Sihala, Islamabad; 4 Department of Clinical Laboratory Sciences, College of Applied Medical Sciences, King Khalid University, Abha, Saudi Arabia; Cholistan University of Veterinary and Animal Sciences, PAKISTAN

## Abstract

Vaccines have always been one of the promising therapeutic sources against many pathogens including infectious fungi. *Candida lusitaniae* is also one of those fungi which is responsible for different infections in human beings including vaginitis, endocarditis, endophthalmitis and blood stream infections. There is thus, a need to adopt effective therapeutic strategies to tackle such infections. Vaccine is one of those efficient therapeutic agents which stimulates immune response and prevents a certain infection to get hazardous. Keeping in view this very important concept, we have designed *in-silico* vaccine against *C*. *lusitaniae* by following the subtractive proteomics approach. Initially, the screening of therapeutic targets was performed to identify potent vaccine candidates from the whole proteome of *C*. *lusitaniae*. Several significant factors were taken into account in this context, such as stability index, IFN status, allergenicity, and antigenicity. As a result, four distinct proteins that were both antigenic and non-allergenic, were selected from the whole proteome. Furthermore, physiochemical investigation revealed that these vaccine candidates were stable and that their IFN status was positive. Notably, each of these proteins was non-homologous to human beings. This particular attribute of the selected proteins i.e., to be non-homologous, was made in order to possess the ability to trigger an immunological response in host (humans). Furthermore, the whole proteome (WP) vaccine was constructed accordingly. The structural modelling of all the selected vaccine candidates was then performed to proceed them further for docking with the human toll-like receptor 2 (TLR2). Afterwards, the codon optimization was executed, followed by *in-silico* cloning of the final vaccine construct. The pet28A plasmid was incorporated for this purpose while, the SnapGene tool was utilized for this particular analysis. Ultimately, the immune simulations were executed to assess the immune response of the designed vaccine (WP). Upon final results, it was found that highest count of IgG and IgM was achieved i.e., up to 700000 between the days 8 to 13 and then slowly neutralized till the day 30. These results signified that the designed vaccine possessed the potential to stimulate the required immune response.

## Introduction

*Candida lusitaniae* also known as *Clavispora lusitaniae* by the teleform name [1], is a human pathogen which belongs to the yeast specie of genus candida which was identified in 1979. Human infections have been linked to this emerging pathogen *Candida lusitaniae* which is an opportunistic haploid yeast and most commonly associated with immunocompromised patients who frequently have multiple medical conditions [2]. *C*. *lusitaniae* can cause oral thrush, vaginitis (superficial infections), endocarditis, endophthalmitis (deep-seated infections of tissues), single organ fungemia (pulmonary fungemia), and blood stream infections, depending on the immune system of the hosts [3, 4]. Although, *C*. *lusitaniae* is a rare pathogen, its increased prevalence in hospitalized patients, patients receiving prolonged antibiotic therapy, patients undergoing chemotherapies or bone marrow transplants, and the ones with underlying malignancies, has sparked special interest in it as a nosocomial pathogen [5–7]. The use of catheters increases the risk of developing candidiasis because they are a major reservoir for yeasts including, *C*. *lusitaniae* which can cause fungemia [5]. Different reported studies have shown, how *C*. *lusitaniae* can create biofilms that lead to endogenous infections [8, 9]. While, similar to other agents that cause candidiasis, it has also been documented that this species can be transmitted through interpersonal contact in the intensive care unit (ICU) [10]. *C*. *lusitaniae* is accountable for roughly 1.7% of all genitourinary candidiasis cases in ambulatory patients and 19.3% of fungemia cases in cancer patients [5, 11, 12].

As far as, the treatment methods of *C*. *lusitaniae* oriented infections are concerned, this pathogen was found to respond well to standard antifungal treatments. But, it has garnered attention due to certain isolates’ resistance to fluconazole, 5-fluorocytosine, and amphotericin B like anti-fungal drugs [13]. Susceptibility testing, however, might be necessary to determine the best course of action because certain strains of *C*. *lusitaniae* are resistant to this medication. Azoles, which prevent the synthesis of ergosterol, a part of the fungal cell membrane, include fluconazole, itraconazole, voriconazole, and posaconazole. However, by changing the target enzyme or the drug efflux pumps, certain strains of *C*. *lusitaniae* may become resistant to these drugs, particularly fluconazole. Eschinocandins, which prevent the synthesis of beta-D-glucan which is a part of the fungal cell wall, including caspofungin, micafungin, and anidulafungin [14].

Clinical reports have indicated that the combination of amphotericin B and fluconazole antifungals can result in the production of multi-resistant isolates of *C*. *lusitaniae* [15]. Therefore, it is suggested that treatment through this kind of combined drugs should be avoided as it may lead to various diseases including deep-seated infections in immunocompromised patients [14]. Hence, there is a need to adopt unconventional approaches to treat *Candida lusitaniae* infections. Therefore, it’s critical to keep an eye on the yeast’s susceptibility to various antifungal medications and to move towards different therapeutic strategies like vaccination in such scenarios. In this aspect, the subtractive proteomics is a technique that mines a pathogen’s proteome for possible targets for vaccine candidates by using computational methods. The objective is to subtract the proteins that are similar or common by comparing the proteomes of the pathogen, host, and closely related organisms. In this way, the pathogen-specific or pathogen-essential proteins can be chosen as potential candidates for vaccine development. The challenges of emerging infections, complex diseases, and antibiotic resistance can be addressed with the aid of this subtractive proteomics approach.

In the current study, the nuclear genome sequence of *Candida lusitaniae* comprising of 12.11Mbp having 6153 protein coding genes and five pseudogenes, was incorporated [2]. While, by the employment of computational algorithms and subtractive proteomics methods, the most appropriate proteins were chosen from this massive collection of data in order to create effective vaccine against *Candida lusitaniae*. *In-silico* design of the vaccine exploit the safety and thermo-dynamic stability of the construct through the verification of certain necessary parameters like antigenicity, allergenicity and physiochemical properties. In this investigation, outer membrane proteins that are thought to be extremely virulent, antigenic, and vital to interactions between hosts and pathogen, were looked into [16]. Afterwards, the selected proteins were utilized to predict Helper T lymphocyte (HTL), B-cell, and cytotoxic T lymphocyte (CTL) epitopes. With the aim to cover the whole proteome of *Candida lusitaniae*, the vaccine was designed by using the most suitable epitopes for immunization. Additionally, the intended vaccine candidates were immunologically assessed by employing molecular docking and immune simulations which indicated the significance and precision of the designed vaccines in terms of inducing the immune response. This study will provide direction to design real-time vaccine against this pathogen which could aid in combating nosocomial infections caused by *Candida lusitaniae*. Nevertheless, additional experimental validations are necessary to validate the safety and effectiveness of the suggested vaccine candidates.

## Materials and methods

### Retrieval of *Candida lusitaniae* proteome and subtractive proteomics

The reference proteome of *Candida lusitaniae* (Uniprot ID: UP000007703) was retrieved from UniProt (https://www.uniprot.org/) which contained 5932 proteins [17]. This whole proteome is also given in the S2 File. Subtractive proteomics approach was followed to identify the proteins that can be incorporated for vaccine designing against *Candida lusitaniae*. Initially, the sub-cellular localization was analyzed to screen out the extra-cellular proteins by using CELLO (http://cello.life.nctu.edu.tw/) which is a well-known tool to predict the sub-cellular localization of the given proteins. This tool works on the basis of support vector machine (SVM) algorithm [18]. Afterwards, the BLASTp tool (https://blast.ncbi.nlm.nih.gov/Blast.cgi?PAGE=Proteins) was incorporated in order to identify the homologous and non-homologous proteins between the pathogen and host (*Homo sapiens*) by using the non-redundant protein sequences (nr) database. It was done to avoid autoimmune response, thus, only non-homologous proteins were selected. The non-homologous proteins were further analyzed for paralogs prediction which was achieved by performing the phylogenetic analysis by utilizing MEGA11 [19].

### Prioritization of the essential proteins

The proteins that were essential for the survival and pathogenicity of the *Candida lusitaniae* were prioritized. These proteins were potent candidates for the vaccine development. As mentioned previously, the cellular localization of the essential proteins were also analyzed because according to the studies it has been reported that extracellular proteins are highly suitable for the development of vaccines since, they play a crucial role in host-pathogen interaction as well as pathogenicity. The antigenicity of the selected proteins that were essential as well as extracellular was also identified with the help of VaxiJen tool (https://www.ddg-pharmfac.net/vaxijen/VaxiJen/VaxiJen.html) [20]. This particular analysis was done by selecting fungi as target organism while, the threshold value was set to 0.5. The shortlisted proteins were further examined by AlgPred (https://webs.iiitd.edu.in/raghava/algpred/submission.html) to estimate the allergenicity status [21]. Resultantly, the non-allergen proteins but with the high antigenicity scores were selected for further analyses.

### Mapping of immunogenic peptides in the target proteins

The selected non-allergen proteins with high antigenicity were further analyzed for immunogenic peptides. In this regard, the cytotoxic T-lymphocytes, Helper T-lymphocytes and B-cell epitopes were predicted. The NetCTL 1.2 (https://services.healthtech.dtu.dk/services/NetCTL-1.2/) was employed in order to predict the cytotoxic T-lymphocytes epitopes [22]. This server predicted the CTL epitopes on the basis of some of the key values including, transportation efficiency, TAP (Transport Associated with Antigen Processing), MHC I binding peptides prediction, and proteasomal C terminal cleavage. The threshold value in this analysis was set to 0.75. Whereas, the affinity score of each peptide was determined by its IC50 value. Peptides having IC50 values less than 50 nM possessed the high binding affinity while, those with IC50 values less than 500 nM were the intermediates and, the ones with IC50 values less than 5000 nM exhibited weak epitope binding affinity. The affinity scores and percentile rankings of the peptides were inversely related, showing that higher the binding affinity, the lower would be the percentile rank, and vice versa.

Furthermore, the prediction of Helper T-lymphocytes was achieved by employing an online server IEDB (https://www.iedb.org/) by using the seven sets of human reference alleles HLAs [23]. In this context, the allergenicity of each MHC-II epitope was determined by AllerTOP v.2.0 (https://www.ddg-pharmfac.net/AllerTOP/) [24]. Moreover, the antigenicity of each epitope was assessed by VaxiJen tool (https://www.ddg-pharmfac.net/vaxijen/VaxiJen/VaxiJen.html). The epitopes which were non-allergen and possessed high antigenicity were chosen for the development of vaccine. Additionally, IFN-epitope server (https://webs.iiitd.edu.in/raghava/ifnepitope/predict.php) was incorporated to analyze the interferon-gamma triggering MHC-II epitopes [25]. This server also utilizes the support vector machine (SVM) score for the prediction of interferon inducing properties of each peptide. The SVM score serves as a measure of the likelihood of a peptide to induce an IFN-gamma response. Lastly, the B-cell epitopes were predicted by the ABCpred (https://webs.iiitd.edu.in/raghava/abcpred/ABC_submission.html). This server had >65% accuracy, 0.49 sensitivity and 0.75 specificity.

### Construction of multi epitopes subunit vaccine

The B-cells, Cytotoxic T-cells and Helper T-lymphocyte epitopes having high binding affinity and antigenicity with the non-allergen nature were employed for designing the final putative vaccine construct. The selected epitopes were linked with the help of different linkers. These linkers were preferred on the basis of data provided by already reported studies [26, 27]. Specific linkers were used for each epitope. The AAY linker was employed for CTL epitopes, GPGPG for HTL and KK linkers for the B-cell epitopes to construct the putative vaccine. These linkers were critical for allowing and maintaining the epitope presentation and separation and, inhibiting the folding in order to promote a successful immune response [28]. Furthermore, the use of an adjuvant improves the immunogenic potential of MEVC peptide vaccine designs. Following an EAAAK linker, all of the MEVC constructs were treated to the addition of a non-toxic adjuvant named human beta defensin-2 (hBD-2) [29]. The hBD-2 possesses the ability to self-produce at the levels, sufficient to elicit a powerful immune response against the attached antigen [30]. The adjuvant was linked to the N-terminal of the final candidate vaccine construct using the EAAK linker [31]. This approach was used in the development of a vaccine targeting particular proteins and a whole proteome vaccine, which is also constructed in this study [27]. These linkers are very important in assisting the prevention of neo-epitopes, ultimately providing structural stability to the vaccine constructs hence, enhancing the immunogenicity [32].

### Prediction of physicochemical properties

To analyze the physiochemical properties of the constructed putative vaccine, an online tool ProtParam (https://web.expasy.org/protparam/) was incorporated [33] This tool predicts different physiochemical attributes including instability index, theoretical PI, *in-vivo* and *in-vitro* half-life, amino acid composition, and grand average of hydropathy (GRAVY).

### Prediction of 2D and 3D structure of the designed vaccines

Secondary structure of vaccine construct was predicted through an online **server** SOPMA (https://npsa.lyon.inserm.fr/cgi-bin/npsa_automat.pl?page=/NPSA/npsa_seccons.html). SOPMA(Self-Optimized Prediction method with alignment) is used to predict the structure and topology of membrane proteins by means of learning machine models, which are trained on the available data of protein sequences’ features, especially the amino acid composition and hydrophobicity profiles [34]. Thereby, the model is iteratively refined through validation and optimization in order to adjust its original prediction ability for accurately predicting both the transmembrane segments and their orientation in new protein sequences [35]. Furthermore, the tertiary structure of the putative vaccines was predicted by an online server trRosetta (https://yanglab.nankai.edu.cn/trRosetta/) [36]. It is a server for automated comparative modeling of three-dimensional (3D) protein structures. The trRosetta is an algorithm for fast and accurate protein structure prediction. Afterwards, the predicted protein structure were refined by using an online server Galaxy Refine (https://galaxy.seoklab.org/cgi-bin/submit.cgi?type=REFINE) [37]. This process was essential for enhancing the accuracy of the predicted structure [38]. For this particular analysis, the protein data bank *(*.*pdb)* file of the predicted model was subjected as input file for further processing. During this refinement, the poor rotamers are removed which enhance the structural quality of the given protein model.

### Discontinuous B cell epitopes

The ElliPro server (ElliPro: Antibody Epitope Prediction (iedb.org)) was used to perform the discontinuous B-cell epitope prediction of the 3D structure of the whole proteome vaccine construct, with default parameters (0.5 as the threshold for the Protrusion Index and a 6 Å threshold for the distance) [39]. ElliPro predicted discontinuous epitopes based on surface exposure and spatial clustering of residues in the 3D structure, each residue being assigned a score of the Protrusion Index [40]. The epitopes according to their PI values, higher values indicating higher possibilities of being immune-recognized, were considered for further analysis [41].

### Disulfide engineering of the vaccine construct

It was essential to increase the improved protein model’s stability before moving on to the next step. Disulfide bonds are covalent interactions that confirm exact geometric conformations, simulating the stabilizing molecular interaction and contributing significantly to the stability of protein models [42]. A unique method for introducing disulfide links into the target protein structure is disulfide engineering. In order to carry out disulfide engineering, the final vaccine construct’s refined model was processed through the Disulfide by Design 2 (Disulfide by Design) (http://cptweb.cpt.wayne.edu/DbD2/). The server processed input data by analyzing protein sequences and structures [43]. The design algorithm proposed optimal bonds based on stability and user constraints. Validation ensured bonds do not disrupt protein structure, with optional simulations for dynamic stability [44]. Outputs included predicted bonds, design proposals, and visualizations. The post-processing provided 3D visualization tools and experimental validation [45]. In order to find residue pairs that could be utilized for disulfide engineering, the revised protein model was first uploaded and ran. Using the Disulfide by Design 2.0 server’s construct mutate function, a total of three pairs of residues were chosen to be mutated with cysteine residues.

### TLR2-vaccine docking

The designed vaccine construct was docked with the TLR2 (Human Toll-like Receptor 2). As TLR2 receptor recognizes a wide variety of pathogen-associated molecular patterns, TLR2 is used as a receptor in docking studies [46]. It is capable of binding with diverse antigens and started signaling cascades that go on to activate the innate immune response, leading to cytokines and chemokine production critical in promoting effective adaptive immunity [47]. TLR2 forms heterodimers with other members of the family of Toll-like receptors, such as TLR1 and TLR6, which help in the broader range of recognition of antigens and make it a very good candidate for an evaluation of how effectively a vaccine construct will be able to elicit immune stimulation [48]. Docking studies including TLR2 have applications in estimating potential effectiveness and immunogenicity of vaccine candidates [49]. For this purpose, the HADDOCK 2.4 server (https://wenmr.science.uu.nl/haddock2.4/) was employed [50]. HADDOCK makes use of biochemical and/or biophysical interaction data such as chemical shift perturbation data, resulting from NMR titration experiments or mutagenesis data to perform the docking analysis. This information is introduced as ambiguous interaction restraints (AIRs) to perform the docking process. However, before the protein-protein docking, the sequence of TLR2 was retrieved from UniProt database (accession ID: O60603) [51] and afterwards, its structure was modelled by incorporating trRosetta (https://yanglab.qd.sdu.edu.cn/trRosetta/). The TLR2 structure was prepared prior to docking, by removing the water molecules, heteroatoms, and other atoms.

### Molecular dynamics (MD) simulations

After the docking process, molecular dynamics (MD) simulations of the docked complexes were performed [52]. MD simulations are a sophisticated computational technique providing insights into the dynamic behavior of atoms within molecules. Researchers use MD simulations to observe and analyze atomic movements over time, applying physical laws in a virtual environment to explore complex molecular dynamics [53]. These simulations are crucial for studying the interactions in the docked complexes, offering a deeper understanding of binding mechanisms, complex dynamics, and the influence of environmental factors. Moreover, by exploring the effects of physical parameters like temperature and pressure, the comprehensive understanding of how external factors impact molecular behavior in a computational setting are also acquired. Additionally, MD simulations assess the structural stability of complexes under varying conditions. In this study, MD simulations were performed by using an online server iMODS (https://imods.iqfr.csic.es/) [54]. The iMODS is a versatile toolkit that enables us to conduct Normal Mode analysis (NMA) of the internal coordinates of the docked structures. It allows for vibrational analysis, motion animations, morphing trajectories, and Monte-Carlo simulations at various scales of resolution. It also enables to gain insights into the movements and interactions of atoms over time, providing valuable information about the dynamics and behavior of biomolecular systems. To better understand all such fluctuations of the docked structures (vaccine candidates-TLR2) in the current study, the complexes were subjected to iMODS by keeping the default parameters to perform the simulations.

### Codon optimization and *in-silico* cloning

The Java codon adaptation (JCat) tool (https://www.jcat.de/) was utilized to clone the multi-epitope subunit vaccine in a suitable vector. This analysis was done for codon optimization and reverse translation to get the nucleotide sequence which was used for cloning [55]. It was also employed to ensure, whether the vaccine construct was highly expressed after cloning in a particular vector. In this tool, three parameters were selected including, bacterial ribosome binding sites, Rho-independent transcription termination, and the restriction enzyme cleavage sites. Moreover, the Jcat calculated the GC content and CAI score of the built vaccine to optimize the reversely transcribed vaccine construct for bacterial expression. The potential vaccine sequence was manually cloned using BspAPI and PsaAI restriction enzymes, and the translated sequence of nucleotides from jcat was then cloned onto the pet28a+ plasmid by employing the SnapGene (https://www.snapgene.com/).

### Immune simulations

In order to create an immunogenic profile of the designed putative vaccine, the immune simulations were performed on C-ImmSim (https://kraken.iac.rm.cnr.it/C-IMMSIM/) [56]. C-ImmSim is a dynamic agent-based simulator for evaluating the body’s immunological responses to antigens. C-ImmSim predicts immunological interactions and epitopes using an agent-based method and the particular scoring matrix, PSSM. The generation of antibodies, interferon, and cytokines was assessed after submitting the prepared vaccine to the web server, by following the default simulation parameters.

**Fig 1:**
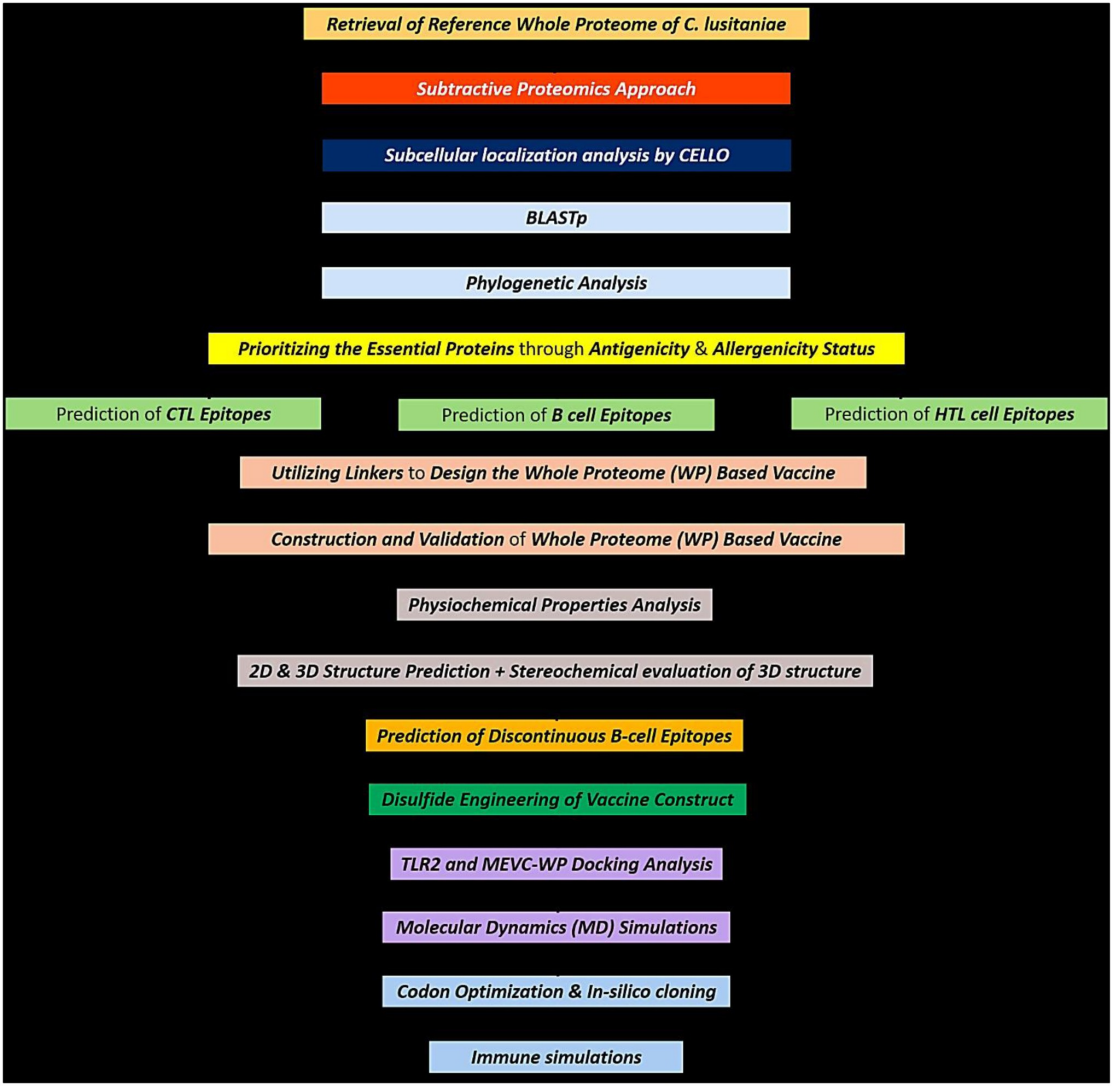
The methodological workflow depicting various approaches followed in this study.

The overall methodology followed in this study can be assessed more conveniently by observing the schematic workflow represented in the Fig 1. Moreover, the details of all of the tools and servers incorporated in this work are also included in the S1 File.

## Results

### Proteome subtraction

**Fig 2:**
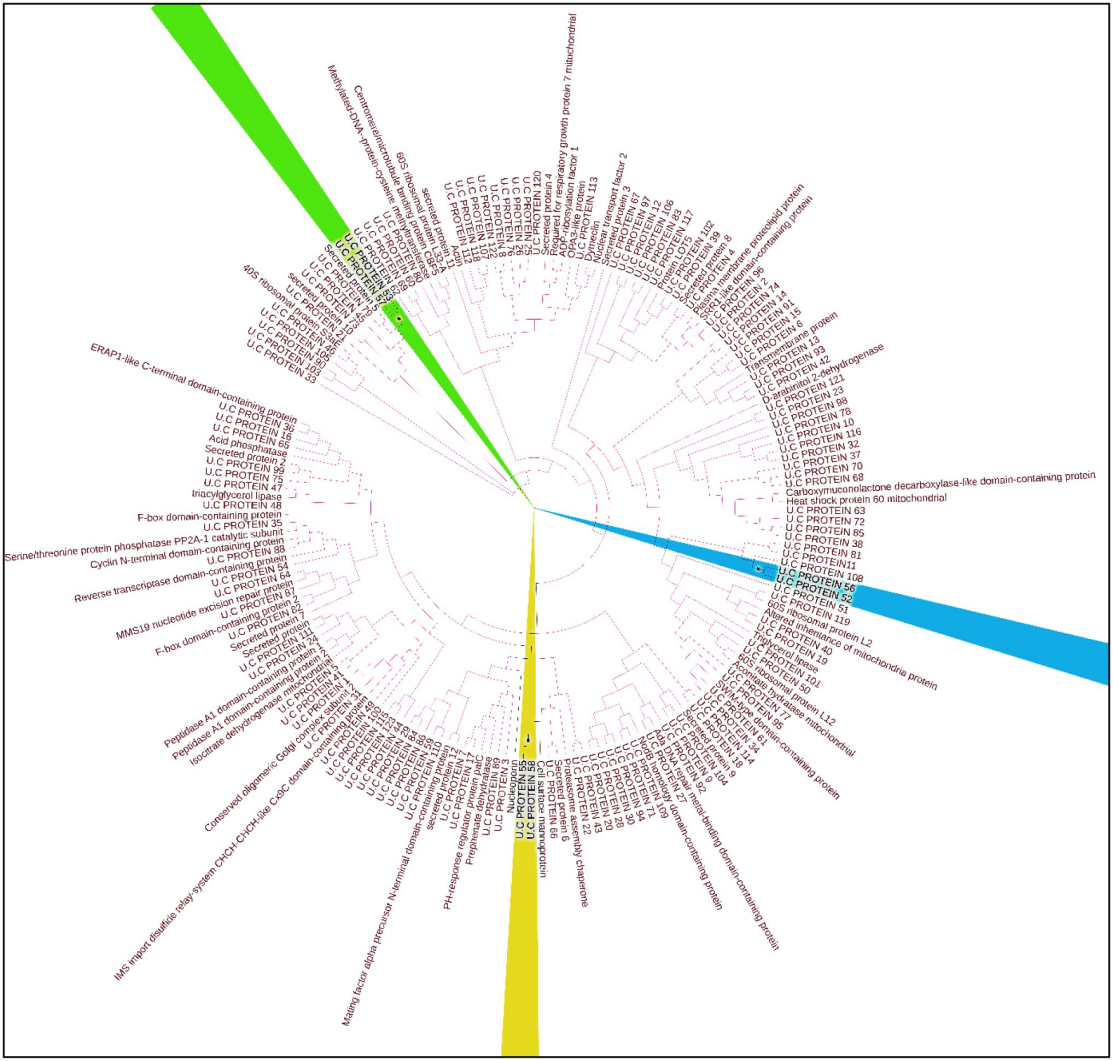
Phylogenetic tree to determine the paralogs. Multiple clades can be observed which are highlighted with different colors, where each clade consists of proteins possessing the similar characteristics.

The process of mining pathogen proteomes for therapeutic targets and to develop therapeutic strategies is greatly aided by their annotation. Target protein identification and characterization are crucial to prioritize vaccine targets and to develop strategies for the therapy of various diseases. Therefore, genome and proteome-wide, as well as functional genomics-based identification and verification of therapeutic biomarkers are frequently used to diagnose various illnesses. In the current study, we used the subtractive proteomics approach to identify the putative targets of vaccines against the emerging pathogen Candida lusitaniae. In this context, the reference proteome of Candida lusitaniae containing 5932 proteins was subjected to CELLO (version 2.0) for the screening of extracellular proteins. Since, the extracellular proteins have effective role in the viral pathogenesis hence, they were considered as targets for vaccine design. Initially, 221 extracellular proteins were shortlisted as putative targets. Furthermore, these extracellular proteins were subjected to BLASTp for further screening of the putative targets. This screening was resulted in the identification of 184 non-homologous and 37 homologous proteins. The homologous proteins were then excluded as they shared high homology with the human genome. The selection of non-homologous proteins is a strategic choice aimed at minimizing the risk of cross-reactivity with human proteins, thereby increasing the safety profile of the vaccine candidates. By opting for non-homologous proteins, we can significantly reduce the likelihood of triggering harmful or autoimmune responses within the host organism. This careful selection process is crucial for enhancing the specificity and safety of the designed vaccine, ensuring that it will effectively target Candida lusitaniae while minimizing the potential for adverse effects on the human body. Ultimately, by focusing on non-homologous proteins, we have tried to create vaccine that is not only potent against infectious agents but also exhibit a high level of safety and precision in their immune response activation. Therefore, after separating the 184 non-homologous proteins, their phylogenetic analysis was performed for determining any possible paralogs. The phylogenetic tree was constructed by employing the maximum likelihood method in MEGA11 software to analyze the presence of paralogs. To determine the reliability and robustness of the tree topology, bootstrapping technique was used while, its value was set to 100. High bootstrap values (>70–80%) indicate the reliability of interior braches of the constructed tee. The Fig 2 highlights three proteins which exhibited the bootstrap value of 99 which illustrated their high similarity to each other, suggesting that they were paralogs. These three proteins were excluded and the remaining 181 were then proceeded for further analysis.

Afterwards, 13 proteins were selected on the basis of their essential genes from the remaining non-homologous proteins. These proteins were further subjected for allergenicity and antigenicity estimation. Consequently, four proteins were determined as highly antigenic and non-allergenic. These 4 proteins were then finally evaluated as the putative targets for vaccine design. According to their specified names which are mentioned on UniProt, these proteins were named as the SVIM type domain containing protein, F box domain containing protein, secreted protein, and heat shock protein and are found to be essential for *Candida lusitaniae* pathogenesis. The SVIM type domain containing protein is mainly involved in gene regulation, DNA repair and development. This protein exhibited the antigenicity score of 0.9 and was determined to be the non-allergen. The other protein F-box domain containing protein possessed the function in the cell cycle, signal transduction, developmental and stress response. The antigenicity score was 0.92 and was determined as non-allergen. While the third targeted protein was found to be involved in the biofilm formation, immune invasion, and tissue invasion. It showed the antigenicity score of 0.92 and was non-allergen. The fourth selected protein for the putative vaccine design had the function in pathogenicity and antifungal resistance. This protein’s antigenicity score was 0.75 and was also found non-allergenic. The amino acid sequences of these four proteins were retrieved from UniProt database and were further subjected to different servers for screening of CTL, HTL and B cell epitopes. The following Table 1 illustrates the collective properties of the selected protein targets for putative vaccine design.

**Table 1:** The short-listed putative targets for vaccine designing along their functions, antigenic score and allergen property.

ID	Name	Function	Antigenicity	Allergenicity
**C4XWH4**	SWIM type domain containing protein	gene regulation, DNA repair, and development	0.73	Non-allergen
**C4Y788**	F-box domain containing protein	cell cycle, signal transduction, development, and stress response	0.87	Non-allergen
**C4YAY0**	Secreted protein	Biofilm formation, immune invasion, tissue invasion	0.92	Non-allergen
**C4XXE5**	Heat shock protein	Pathogenicity and antifungal resistance	0.75	Non-allergen

### Immune based epitopes screening of targeted proteins

B cells and T lymphocytes which are involved in adaptive immunity, have the ability to recognize the antigenic components [57]. T cells have various receptors which aid in recognizing the antigens while, the MHC molecules which are present on T cells, are crucial for the epitope presentation [58]. Immunoglobulins which are secreted after B cells differentiation, function as antibodies and perform different tasks such as pathogen neutralization and targeted antigen destruction. B cells indirectly kill pathogens by activating the complement system which destroys the pathogens by different pathways [59]. B cell and CTL in particular, are essential part of humoral and cell mediated immunity [59]. Recent advancements in the computational modeling and the development of specific structural vaccination algorithms forecasts the validity of epitopes as a fantastic substitute for rational vaccine design. Here, we looked forward the potent CTL, B cells and HTL epitopes of the previously selected *Candida lusitaniae* proteins. Among the screening of CTL epitopes a total of 7, 11, 9 and 13 MHC binders were identified for each protein respectively. Similarly, the number of B cell epitopes identified for each protein were 29, 26, 30 and 34 respectively, and, for HTL there were 2038, 2227, 3403 and 3914 epitopes for each of the proteins.

Further analysis was done on these epitopes to determine their antigenicity and allergenicity. For vaccine design, it was mandatory for each epitope to be non-allergen and highly antigenic in nature. For C4XWH4 protein, two CTL epitopes YVDSLYTFL, RLDLDTLEV with the antigenicity scores of 0.8 and 1.8, respectively and having the high affinity score, were selected. While, the combined affinity score of two epitopes was greater than 1. For C4Y788 protein, two epitopes i.e., FTQFSSLKV, AIGEQVRLY at the corresponding position of 188 to 197 and 323 to 332, were selected. These epitopes were non-allergen, and their combined antigenicity score was also greater than one. Along with the allergenicity and antigenicity, the high binding affinity was also kept in consideration. Further, the affinity scores of secreted and heat shock protein were also computed which were found to be in the range of 1.0–2.3. For the third protein (C4YAY0), the two epitopes i.e., LSVSTFILY, LTADFWLVV having the antigenicity score of 0.9 and 1.5 respectively, were selected. In the similar fashion, the epitopes FSDSSSGGV and SLDSTNLNL with the binding scores of 1.7 and 1.0, respectively, were selected for the fourth protein (C4X6E5). The summarized results of this overall analysis are summarized in the following Table 2.

**Table 2:** Summarized results of the predicted cytotoxic T-cell (CTL) epitopes, their respective positions, affinity score, antigenicity and allergen status.

Name	CTL	Start	End	Affinity score	MHC Binder	Antigenicity	Antigen status	Allergenicity
**C4XWH4**	YVDSLYTFL RLDLDTLEV	27 161	36 169	1.3 0.9	7	0.8 1.87	Antigen	Non-allergen
**C4Y788**	FTQFSSLKV AIGEQVRLY	188 323	197 331	0.9 0.8	11	1.1 3.0	Antigen	Non-allergen
**C4YAY0**	LSVSTFILY LTADFWLVV	11 77	19 86	2.3 1.4	9	0.9 1.5	Antigen	Non-allergen
**C4XXE5**	FSDSSSGGV SLDSTNLNL	145 277	154 286	1.7 1.0	13	1.7 1.2	Antigen	Non-allergen

Afterwards, similar approach was followed to predict the B cell epitopes on the basis of antigenicity, binding score and the allergenicity status. In this aspect, the 2 epitopes of 16 amino acids for each protein were selected. For SVIM type domain containing protein, the two epitopes i.e., PGLVSRRPYVDSLYTF, HGMHLGPQVPKGKHVP were selected. The antigenicity score was 1.4 for both epitopes and were non allergen. Their binding scores were computed as 0.89 and 0.93, respectively. Furthermore, two epitopes were predicted for F box domain containing protein. The amino acid residues in these epitopes were TLEQFSPDSNAARYSN and TSMGEAPQESFSLAEQ at the corresponding positions of 28 and 256 with the binding score of 0.83 and 0.92 and were found to be non-allergen. The antigenicity score of the selected epitopes were calculated as 0.8 and 0.9, respectively. Furthermore, for the secreted and heat shock protein, the epitopes which possessed the effective antigenicity and binding score, were selected. Similarly, two epitopes which are GGGLGEKESHVSGQLD and ASLGQHQIDKRRHVAQ at the corresponding positions of 201 and 235 respectively in the C4YAY0 protein, were selected. The antigenicity score was 1 for both epitopes. The epitopes selected for C4XXE5 protein were LGEDGTTGEDGNITQS and GDEVRRDVTTVKLHTF. These epitopes exhibited the binding score of 0.90 and 0.86, respectively and, were shortlisted for the putative vaccine design. The combined results of all these B cell epitopes are presented in the Table 3.

**Table 3:** Predicted cytotoxic B Cell epitopes along their respective positions, binding score, number of epitopes identified and profiling of antigenicity and allergenicity.

Name	B Cell	Start	Binding Score	Antigenicity	Epitopes Identified	Allergenicity
**C4XWH4**	PGLVSRRPYVDSL YTF HGMHLGPQVPKGKHVP	19 190	0.93 0.89	1.4 1.4	25	Non-allergen
**C4Y788**	TLEQFSPDSNAARYSN TSMGEAPQESFSLAEQ	28 256	0.83 0.92	0.8 0.9	26	Non-allergen
**C4YAY0**	GGGLGEKESHVSGQLD ASLGQHQIDKRRHVAQ	201 235	0.87 0.85	1.0 1.1	30	Non-allergen
**C4XXE5**	LGEDGTTGEDGNITQS GDEVRRDVTTVKLHTF	252 349	0.90 0.86	2.6 1.3	34	Non-allergen

For the potential HTL epitope prediction, the SVM method was incorporated for C4XWH4 protein. As a result, two interferon (IFN) inducing HTL epitopes i.e., ‘KNVSTVHVDSDKTVL and MLEVLGLRLDLDTLE‘, were selected. While, for C4Y788 protein, the epitopes with the high antigenicity score of 1.1 and 1.0 which presented the non-allergenicity status and were also the potential IFN inducing agents, were selected. Whereas two epitopes for the secreted protein which was exhibiting positive IFN value were selected. Each of the HTL epitope was comprising 15 amino acid residues. For the fourth protein the two IFN inducing HTL epitopes were shortlisted. The antigenicity score was ranging from 0.9 to 2.3 for the last two proteins. The overall results of HTL epitopes along their respective scores are shown in the Table 4.

**Table 4:** Summarized results of predicted HTL epitopes, the method utilized, IFN status and antigenicity and allergenicity profiling.

UniProt ID/Putative	HTL-Epitopes	Method	IFN	Antigenicity score	Antigenicity Status	Allergenicity	Numbers
**C4XWH4**	KNVSTVHVDSDKTVL MLEVLGLRLDLDTLE	SVM	POSITIVE	**1.5679** **2.6906**	YES	NON-ALLERGEN NON-ALLERGEN	2038
**C4Y788**	LEQFSPDSNAARYSN TLEQFSPDSNAARYS	SVM	POSITIVE	**1.1132** **1.0166**	YES	NON-ALLERGEN	2227
**C4YAY0**	LGHNGLGLQLGHGTG QSVTAQAWSVCCGQD	SVM	POSITIVE	**2.3777** **2.1439**	YES	NON-ALLERGEN	3403
**C4XXE5**	TQLLVKVFCLGLDVV DFLGSGDFLLVLLQK	SVM	POSITIVE	**1.7238** **0.9555**	YES	NON-ALLERGEN	3914

### Multi-epitope vaccine constructs (MEVC’s) of the selected proteins

Bioinformatics analysis has provided a platform which assists in the identification of targets during designing of the therapeutic agents against human pathogens. This approach was followed for the designing of multi epitope vaccine constructs against the four pre-analyzed proteins of *Candida lusitaniae*. These multi-epitope vaccine constructs were designed by joining the shortlisted six highly antigenic CTL, HTL and B cell epitopes for each protein with the help of different peptide linkers such as EAAK, AAY, GPGPG and KK, as illustrated in Table 5. Furthermore, the immunogenic potential for MEVC peptide vaccine design was increased by the adjuvant. After an EAAK linker, non-toxic adjuvant human beta defensin2 (hBD2) was added to each of the designed MEVC constructs. With its high expression levels and ability to self-produce, hBD-2 can trigger a strong immune response against the attached antigen. For each protein, the constructs comprising of 165 amino acids for each protein were built by linking the epitopes with the help of adjuvant and linkers. These constructs were named as MEVC1, MEVC2, MEVC3 and MEVC4 having the antigenicity score of 1.2, 0.9, 1.18, and 1.12, respectively and were found to be non-allergen.

**Table 5:** Shortlisted vaccine candidates along their respective sequences, number of amino acid residues, antigenicity and allergenicity status.

UniProt IDs/Vaccine Name	Protein Specific and Proteome-wide MEVC Constructs	Number of Amino Acids	Antigenicity score	Antigenicity Status	Allergenicity
**C4X WH4**	MRVLYLLFSFLFIFLMPLPGVFGGIGDPVTCL KSGAICHPVFCPRRYKQIGTCGLPGTKCCKK PEAAKYVDSLYTFLAAYRLDLDTLEVGPGP GKNVSTVHVDSDKTVLGPGPGMLEVLGLRL DLDTLE KKPGLVSRRPYVDSLYTFKKHGMHLGPQVP KGKHVP	165	1.2371	YES	NON-ALLERGEN
**C4Y7 88**	MRVLYLLFSFLFIFLMPLPGVFGGIGDPVTCL KSGAIC HPVFCPRRYKQIGTCGLPGTKCCKKPEAAKF TQFSSLKVAAYAIGEQVRLY GPGPGLEQFSPDSNAARYSNGPGPGTLEQFS PDSNAARYSKK TSMGEAPQESFSLAEQKKTLEQFSPDSNAAR YSN	165	0.9043	YES	NON-ALLERGEN
**C4Y AY0**	MRVLYLLFSFLFIFLMPLPGVFGGIGDPVTCL KSGAIC HPVFCPRRYKQIGTCGLPGTKCCKKPEAAKL SVSTFILY AAYLTADFWLVVGPGPGLGHNGLGLQLGH GTGGPGPGQSVTAQAWSVCCGQD KKGGGLGEKESHVSGQLDKKASLGQHQIDK RRHVAQ	165	1.1898	YES	NON-ALLERGEN
**C4X XE5**	MRVLYLLFSFLFIFLMPLPGVFGGIGDPVTCL KSGAICH PVFCPRRYKQIGTCGLPGTKCCKKPEAAK FSDSSSGGVAAYSLDSTNLNLGPGPGTQLLV KVFCLGLDVVGPGPG DFLGSGDFLLVLLQKKKLGEDGTTGEDGNIT QSKKGDEVRRDVTTVKLHTF	165	1.1295	YES	NON-ALLERGEN
**Whole Proteome**	MRVLYLLFSFLFIFLMPLPGVFGGIGDPVTCL KSGAICHPVFCPRRYKQIGTCGLPGTKCCKK PEAAKYVDSLYTFLAAYRLDLDTLEVAAYF TQFSSLKVAAYAIGEQVRLYAAYLSVSTFIL YAAYLTADFWLVVAAYFSDSSSGGVAAYSL DSTNLNLGPGPGKNVSTVHVDSDKTVLGPG PGMLEVLGLRLDLDTLEGPGPGLEQFSPDSN AARYSNGPGPGTLEQFSPDSNAARYSGPGPG LGHNGLGLQLGHGTGGPGPGQSVTAQAWS VCCGQDGPGPGTQLLVKVFCLGLDVVGPGP GDFLGSGDFLLVLLQKKKPGLVSRRPYVDS LYTFKKHGMHLGPQVPKGKHVPKKTSMGE APQESFSLAEQKKTLEQFSPDSNAARYSNKK ASLGQHQIDKRRHVAQKKLGEDGTTGEDG NITQSKKGDEVRRDVTTVKLHTF	447	1.3249	YES	NON-ALLERGEN

### Whole proteome vaccine construct

**Fig 3:**
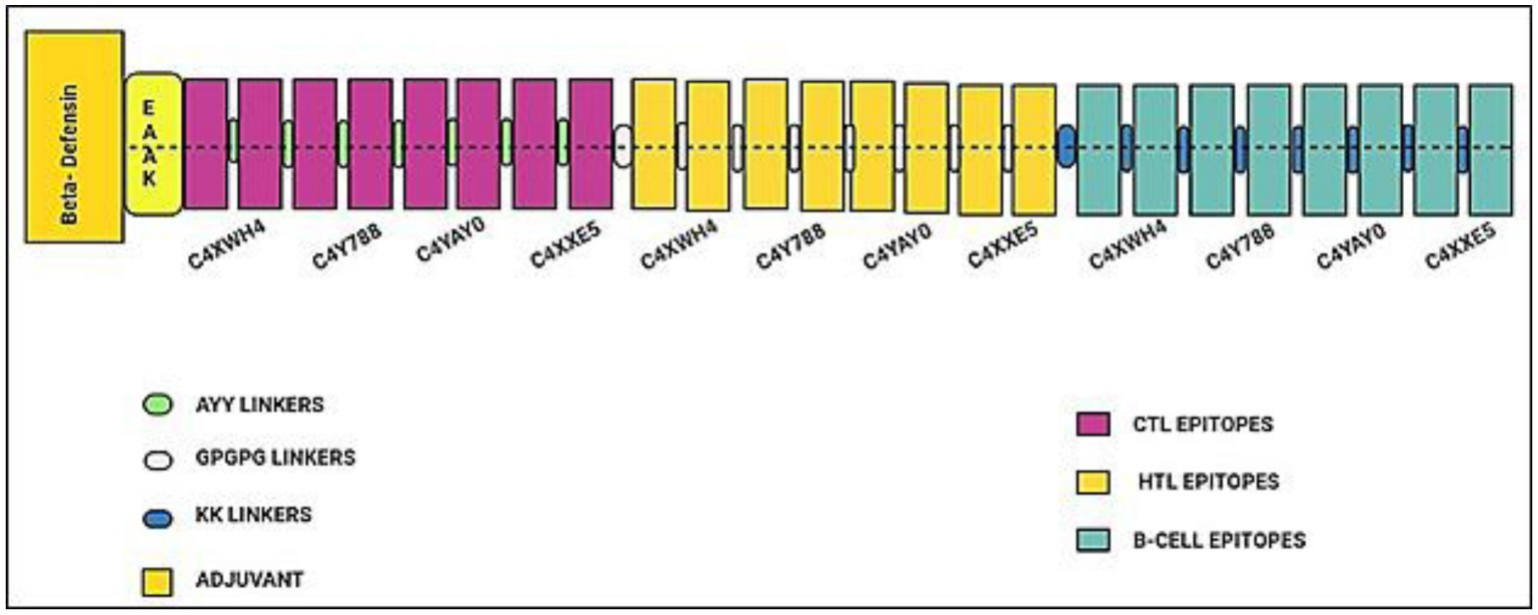
MEVC-WP topographical organization, with adjuvant position, CTL HTL, B cell epitopes, and corresponding linkers displayed in various colors.

By connecting adjuvant and 8 CTL, 8 HTL and 8 B cell epitopes with the help of above linkers, the whole proteome based multi epitope vaccine construct (WP-MEVC) was designed. The final construct possessed the length of 447 amino acids. The experimental feasibility of the final construct was high, as it displayed an antigenicity score over 1. The general schematic diagram of proposed whole proteome vaccine construct which includes epitopes from various proteins and appropriate additional linker is shown in Fig 3. Each mapped epitope can be well observed in a distinct color.

### Physiochemical properties

The physiochemical properties provides insights of various crucial factors regarding the physical and chemical attributes of the proteins under study. These attributes include the stability index, no. of amino acid residues, half-life and number of positively and negatively charges residues, among other important properties. Thus, ProtParam server was employed to analyze the physiochemical perspectives of the MEVC’s. Molecular weight 17 to 47 kD, theoretical Pi 8.68 to 9.30, and other variables assessing the vaccine stability and viability for future experimental designs, were also among these parameters. In addition, the half-life, GRAVY (grand average of hydropathicity), aliphatic index and thermostability were also examined. The Table 6 demonstrates various physiochemical characteristics for all the MEVC’s under study.

**Table 6:** The details of the physiochemical properties of the selected vaccine candidates including stability status, no. of amino acid residues, theoretical pI, no. of negatively and positively charged residues, among other crucial parameters.

UniProt IDs/ Vaccine name	Stability	No. of Amino Acids	Stability score	Molecular weight (D)	Theoretical pI	Negative charged residues (Asp+glu)	Positive charged residues (Arg + Lys)	Half life	Total no. of atoms	Aliphatic index	Hydropathicity (GRAVY)
**C4X WH4**	STA BLE	1 6 5	30.5 1	1803 2.38	9.25	13	21	mammals 30 hours yeast >20 hours E. coli >10 hours	257 6	93.2 1	0.061
**C4Y 788**	STA Ble	1 6 5	38.4 3	1787 8.48	9.05	12	18	mammals 30 hours yeast >20 hours E. coli >10 hours	249 5	63.9 4	-0.254
**C4Y AY0**	STA BLE	1 6 5	36.9 7	1741 0.35	9.30	8	17	mammals 30 hours yeast >20 hours E. coli >10 hours	245 7	84.4 8	0.064
**C4X XE5**	STA BLE	1 6 5	34.0 4	1747 4.40	8.69	14	18	mammals 30 hours yeast >20 hours E. coli >10 hours	248 0	89.7 0	0.133
**WP**	STA BLE	4 4 7	33.8 5	4759 2.30	8.68	38	44	mammals 30 hours yeast >20 hours E. coli >10 hours	668 9	80.0 4	-0.171

### Secondary structure prediction and 3D modeling of multi-epitope vaccine construct

**Fig 4:**
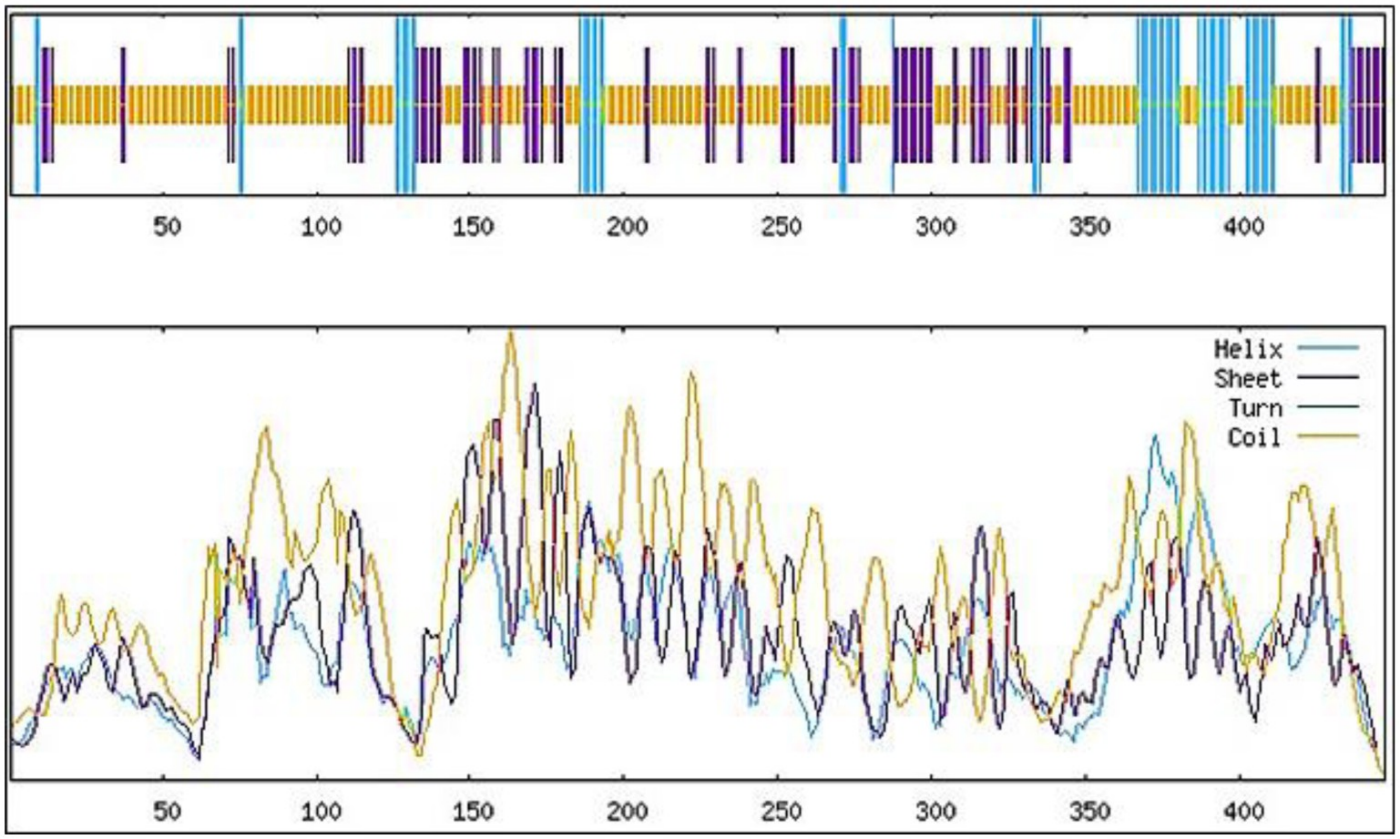
Secondary structure prediction of whole proteome vaccine construct.

**Fig 5:**
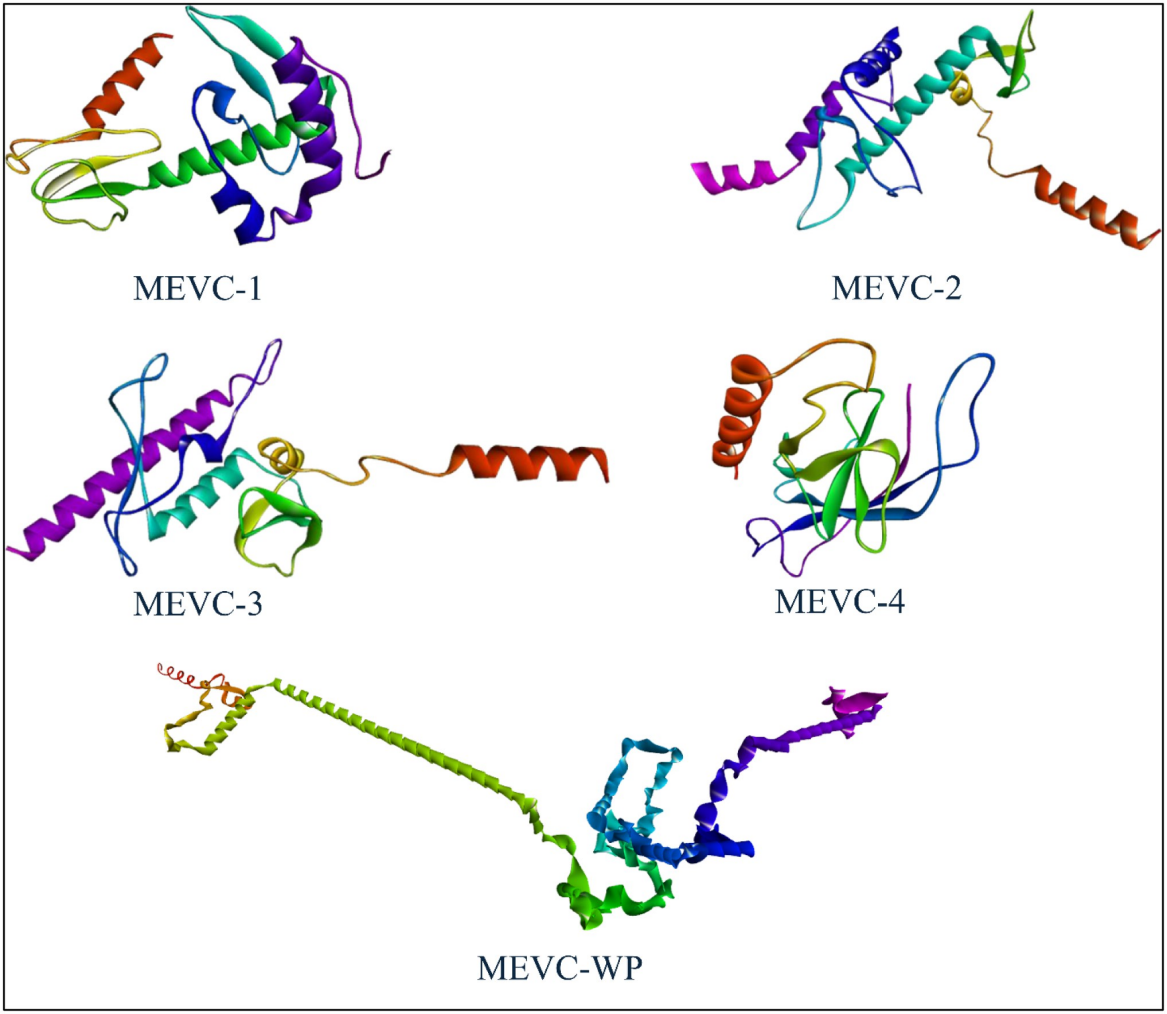
Representations of three dimensional models of multi-epitope vaccine constructs; MEVC1, MEVC2, MEVC3, MEVC4, Whole proteome vaccine construct (WP-MEVC).

**Fig 6:**
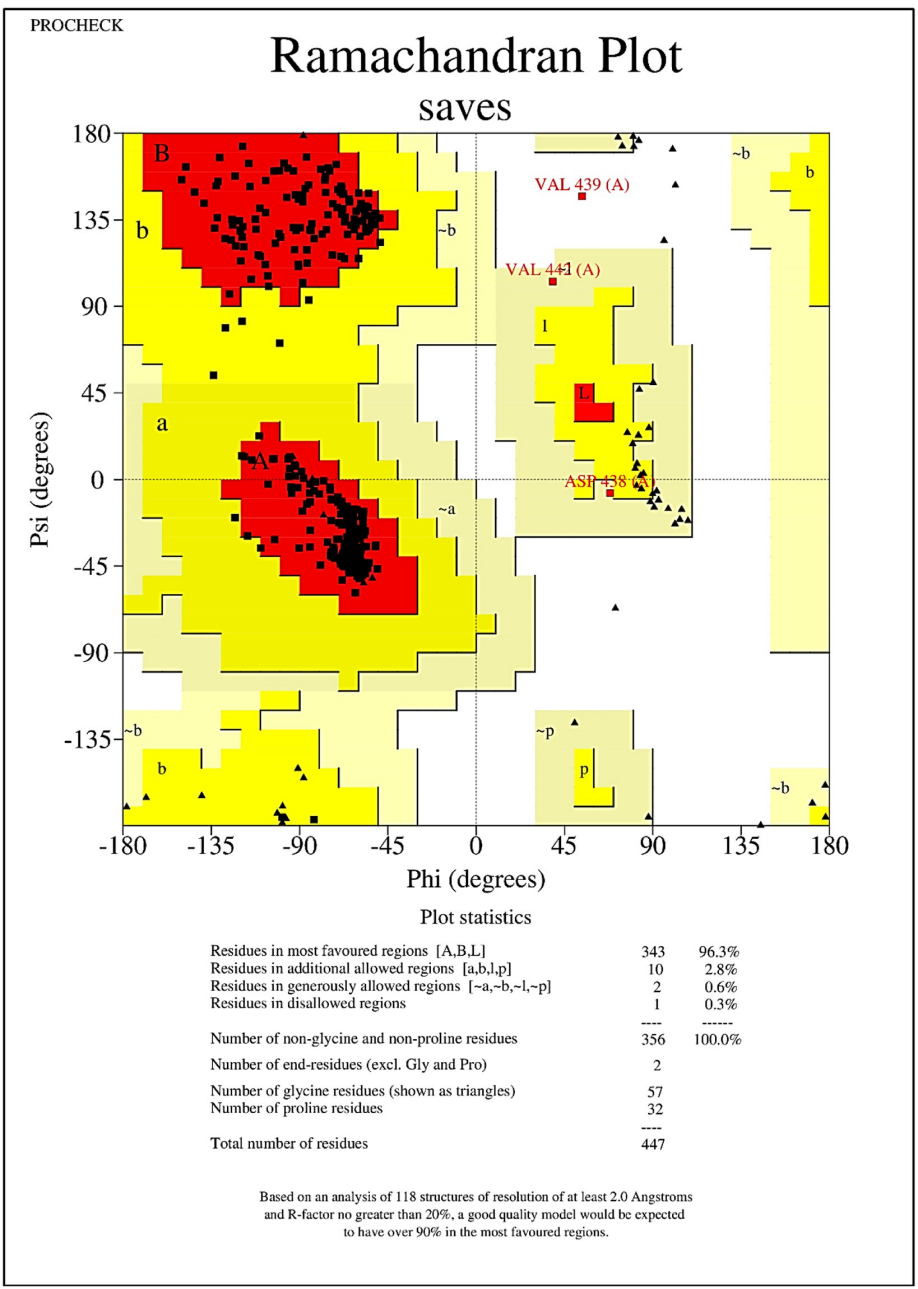
Ramachandran plot, displaying the allocation of amino acid residues in most favorable, additionally allowed, generously allowed and disallowed regions. It can be observed that 96.3% of the residues lie in the most favorable region, signifying the good quality of the modelled structure (MEVC-WP).

The results of SOPMA showed that the whole proteome vaccine construct consisted of 63.31% of random coils, which provided a secondary structure with a lot of flexibility. Alpha helices represented 14.32%, causing in the formation of a stable helical region in them while, 22.37% was constituted of extended strands or beta-sheets, providing some domains with very stable core regions. These results can be visualized in other interesting layouts in the form of peaks and bars in Fig 4. The graphical data showed the structures distributed throughout the protein, presenting the regions of flexibility interspersed with more rigid and structured areas, highlighting a versatile protein. While, for the 3D structure modeling of vaccine constructs, the trRosetta server was used which is the web-based platform for the efficient and accurate protein structure prediction. It develops the protein structure using direct energy minimizations with a constrained Rosetta. This server predicts the entire residue geometries of proteins including distances and orientation. In current scenario, three dimensional (3D) structure modeling for each of the constructed MEVC’s was performed with the utilities of the trRosetta server. While, the generated models were visualized BIOVIA Discovery Studio Visualizer. The refined best models are shown in the Fig 5 which were proceeded for further analysis. The whole proteome vaccine construct was refined using galaxy refine. For the validation of the predicted structure of whole proteome vaccine construct, Ramachandran plot which is available at PROCHECK server (https://saves.mbi.ucla.edu/), was incorporated. This plot was basically used to validate and assess the quality of the predicted protein structure and to identify the potential errors presents in the backbone conformation of that protein. The resulted Ramachandran plot suggested that most of the amino acids residues were present in the most favorable regions which indicated the good quality of the predicted structure. Ramachandran plot also generates the results in the form of Ramachandran scores. If this score is greater than 90% then, it indicates that the predicted protein structure is of good quality. In our context, the Ramachandran score of 96.3% was computed which suggested that the predicted structure was stereochemically valid. The results of Ramachandran plot are illustrated in the Fig 6.

### Discontinuous B cell epitope prediction

**Fig 7:**
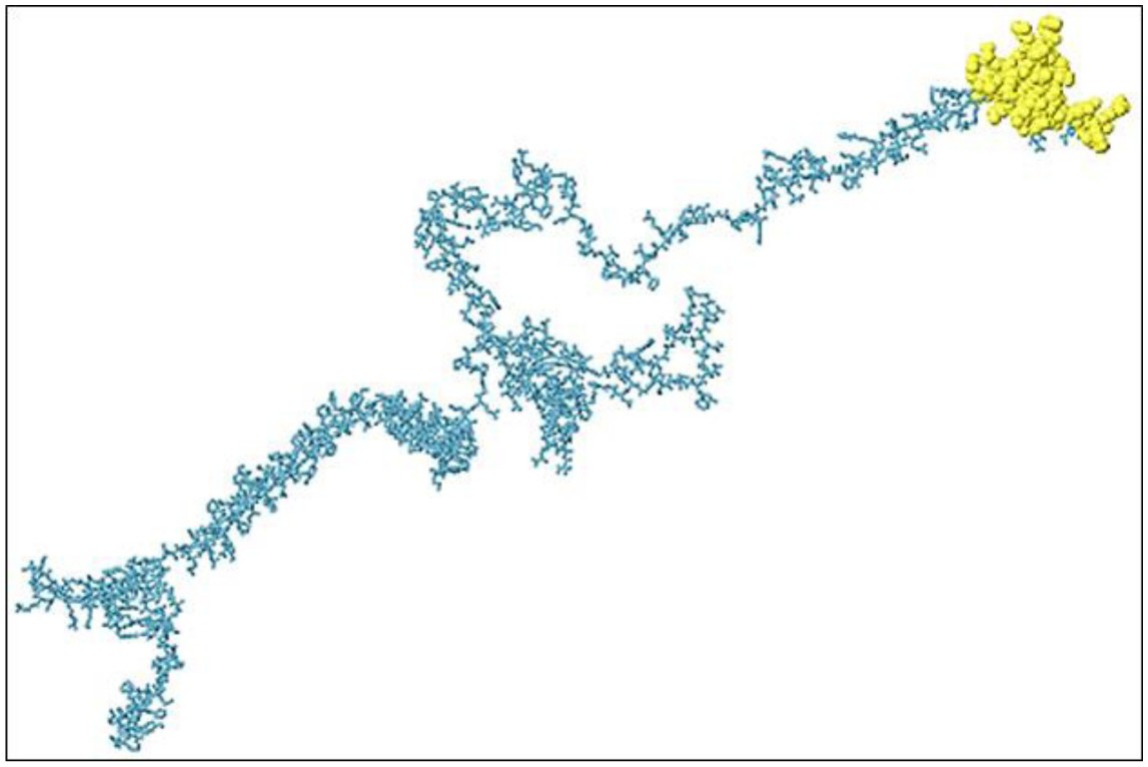
The illustration of discontinuous B cell epitope in surface exposed portion which can be observed by yellow color region of the vaccine construct.

This particular analysis identified six discontinuous B-cell epitopes in the whole proteome vaccine construct, each consisting of surface-exposed residues likely to be recognized by antibodies. Each of these epitopes had different protrusion index (PI) scores which indicated their accessibility at the surface level, where epitope 1 composed of 38 amino acids and it displayed the highest PI value of 0.922, which means it can be recognized by antibodies very easily. In addition, epitope 2 (90 residues) had a PI score of 0.738 while epitope 3 (39 residues) illustrated a value of 0.702, suggesting that these epitopes were also found to be highly exposed at their surfaces thus making them excellent targets for immune response inducing agents. However, others exhibited progressively lower but surface-exposed PI scores with a lesser degree of relevancy as compared with the three, mentioned above in terms of protein structural stability. These predictions highlighted key regions for antibody interaction that are critical for vaccine design. The Fig 7 represent the discontinuous B cell epitope of the vaccine construct.

### Disulfide engineering

**Fig 8:**
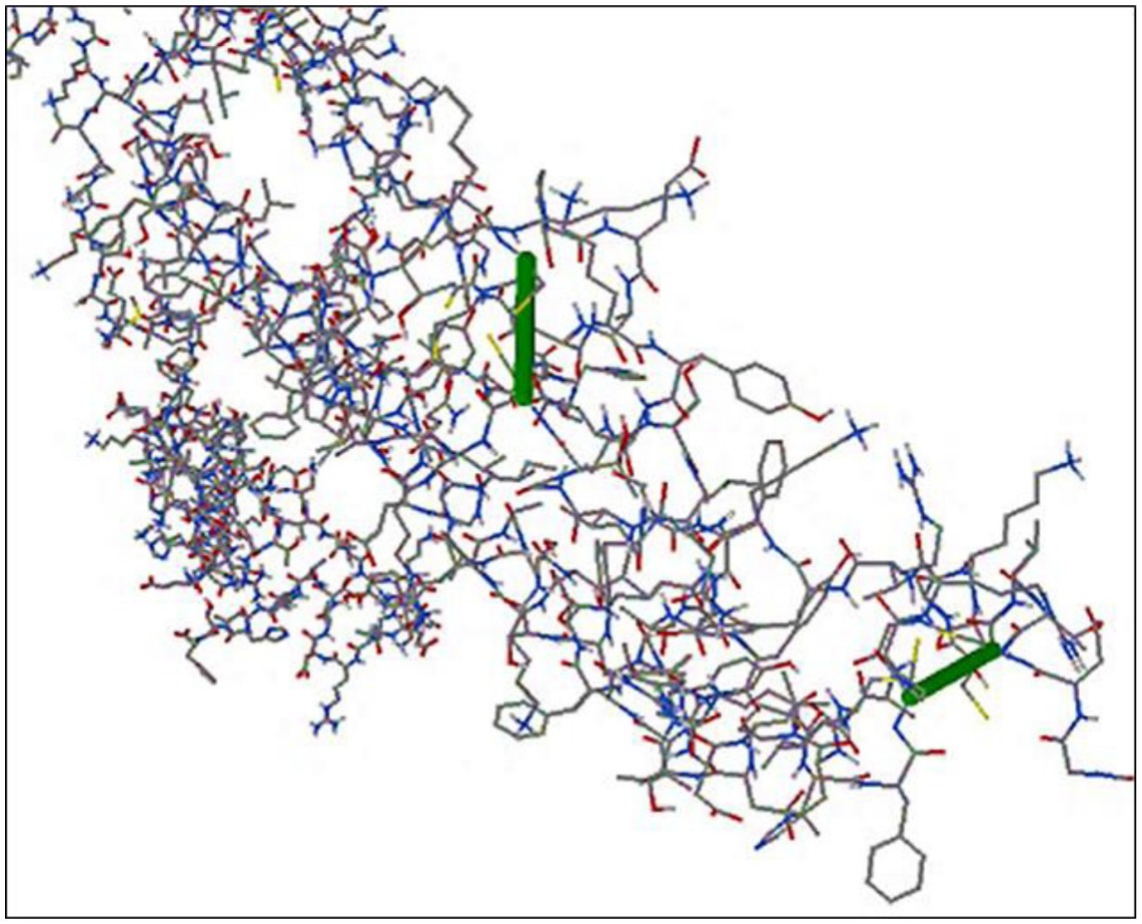
The representation of disulfide bonds in order to enhance the stability of the vaccine construct while, green areas in the figure highlight those regions where disulfide bonds were included.

The disulfide engineering was performed in order to stabilize the predicted structure of the final vaccine constructs. It was discovered that a total of 17 pairs of residues might be utilized in this way. However, following assessment of additional factors such as energy and Chi3 value, only three pairs of residues Pro28-Cys23, Cys53-Cy60, and Ser383-Ser386 were determined to be final since their values were seen to be falling within the permitted range, i.e., energy values must be less than 2.2 and Chi3 values must be between -87 and +97 degree37. As a result, two pairs as shown in Fig 8 successfully formed disulfide bonds, such that bond distance, angles (like Chi3), and the energy minimized structure resulted in favorable conditions for disulfide bonds. Two different areas where disulfide bonds were included during this particular analysis can be observed in the Fig 8.

### MEVCs-TLR2 docking analysis and demonstration of interacting docked residues

**Fig 9:**
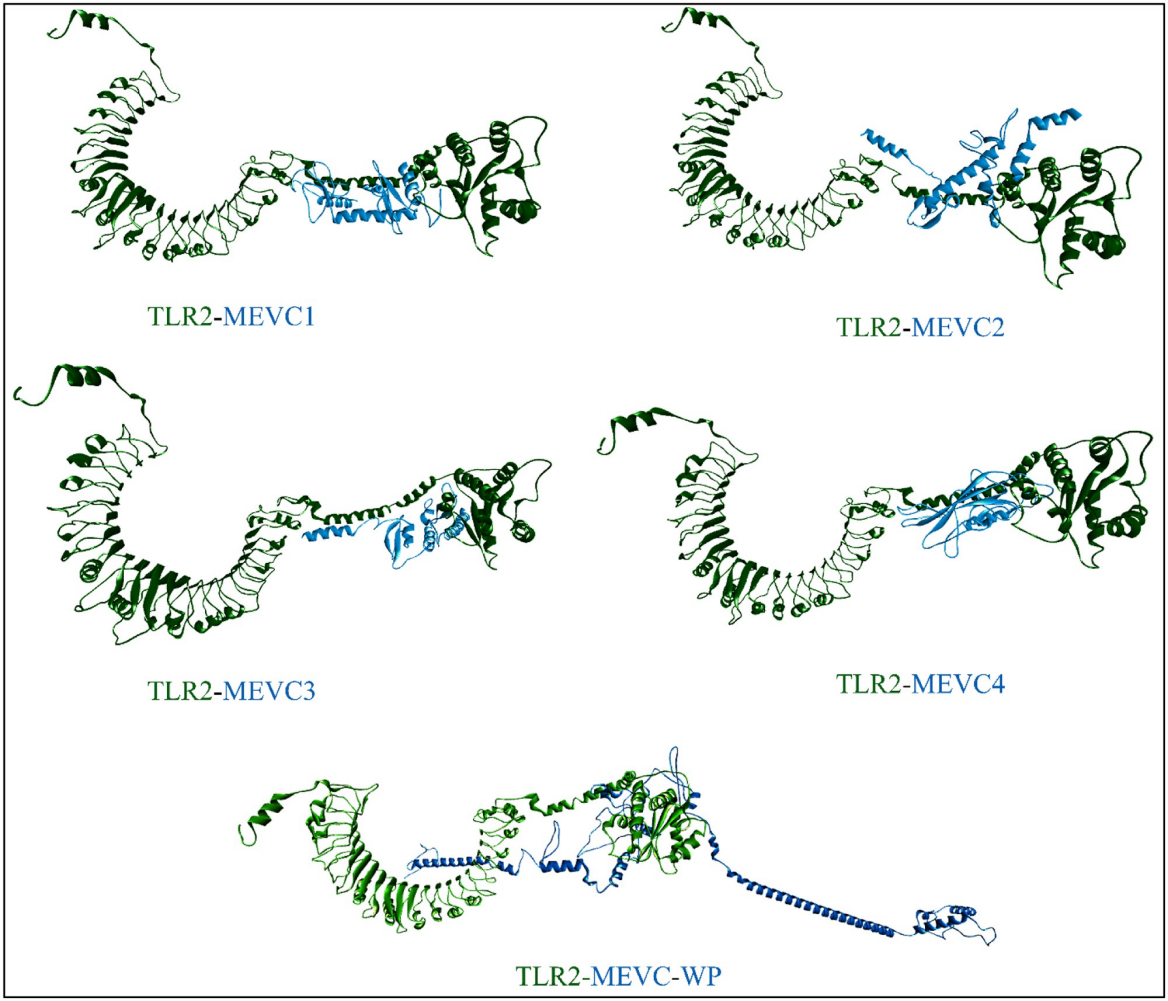
Illustration of docking interactions of MEVC’s with TLR2; MEVC1-TLR2, MEVC2-TLR2, MEVC3-TLR2, MEVC4-TLR2, WP-TLR2.

**Fig 10:**
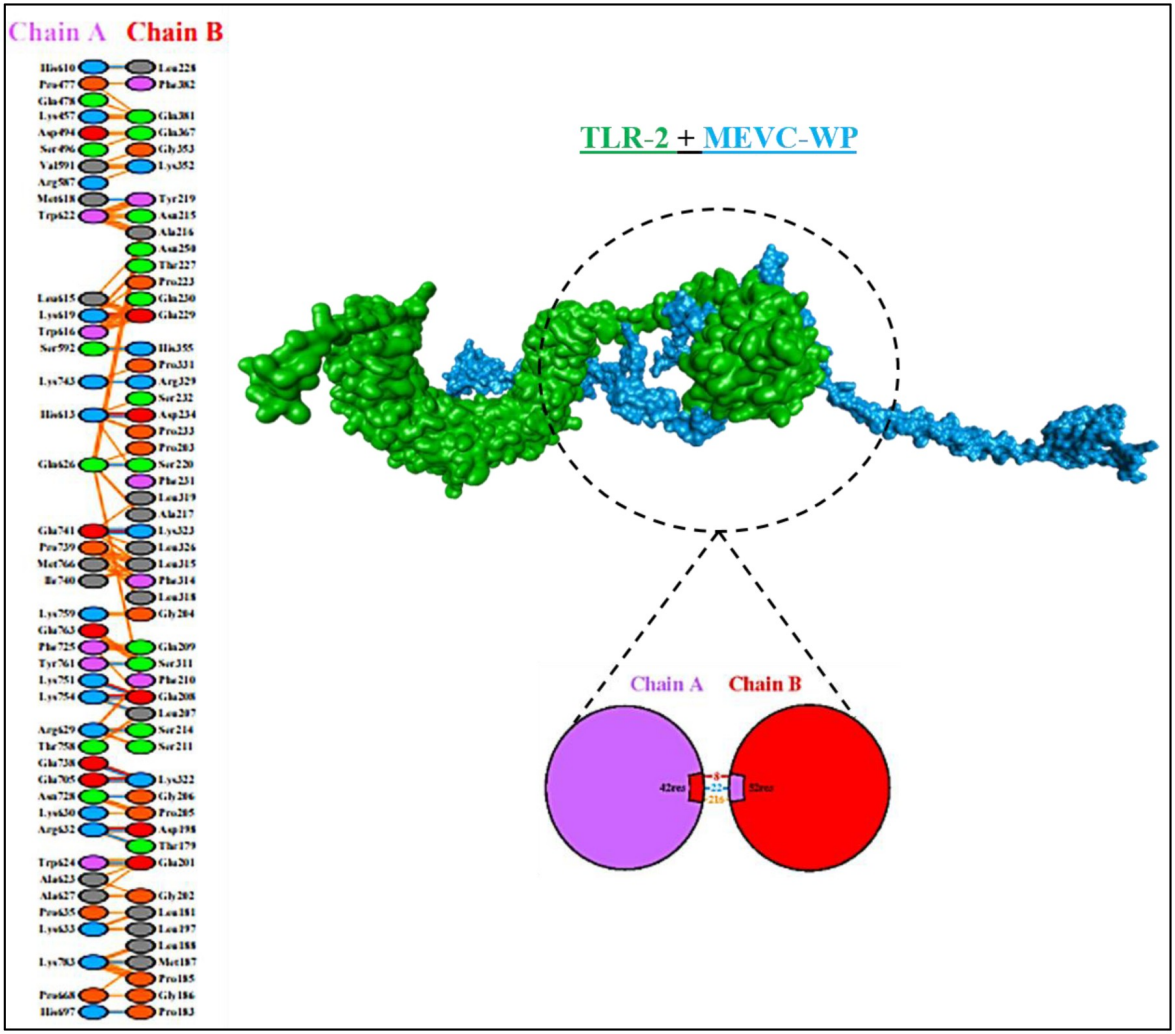
Illustration of the interacting docked residues of the final vaccine construct and human toll-like receptor 2, resulted by PDBsum.

The HADDOCK server was used to assess the interaction between the toll like receptor 2 (TLR2) and the vaccine constructs. The docking results include the analysis of Z value by the HADDOCK server. The Z score in this server indicates that how many standard deviations occur from the average values. The lower score indicates the better interaction and quality of the docked complexes. The docking analysis typically predicted the conformation, position, and orientation of vaccine with TLR2 receptor site. All of the resulted three dimensional docked complexes are shown in the Fig 9 in which two distinct colors, green and blue highlights the docked TLR2 and MEVC’s, respectively. While, the respective Z-scores of all the docked complexes are demonstrated in Table 7. These scores of all the docked complexes fall in an appropriate range of -1.4 to -2.3 which signified the stable docking interactions. Furthermore, the in-depth analysis of the specified docked residues in each of the docked complex was also performed in order to comprehend the number of H-bonds, salt bridges and other interactions. This task was performed by utilizing the PDBsum server (https://www.ebi.ac.uk/thornton-srv/databases/pdbsum/). In this regard, MEVC1-TLR2 construct formed 3 salt bridges, 11 hydrogen bonds and 221 non-bonded interactions. MECV2-TLR 2 exhibited 12 hydrogen bonds, 265 non-bonded interactions, and 6 salt bridges. Whereas, MEVC3-TLR2 and MEVC4-TLR2 constructs formed 3 salt bridges, 19 and 11 corresponding hydrogen bonds and presented 288 and 235 non-bonded interactions, respectively. Lastly, the results of WP-TLR2 analysis displayed 8 salt bridges, 22 hydrogen bonds and 216 non-bonded interactions. Moreover, all of the constructs had a relatively consistent GC% of 52–55%, which suggested a balanced nucleotide composition. All of the relevant parameters of docking analysis along their respective scores for each docking complex are mentioned in the Table 7. Whereas, to comprehend the docking interactions of TLR2-WP vaccine construct, the results of PDBsum are also illustrated in the Fig 10.

**Table 7:** The results of docking interactions including the Z-values, CAI, GC% among other important parameters along their respective scores.

Name	Z value	CAI	GC%	Salt bridges	H bond	Non- bonded
MEVC1-TLR2	-1.4	1.0	54	3	11	221
MECV2-TLR2	-1.8	1.0	52.7	6	12	265
MEVC3-TLR2	-2.3	0.95	55	3	19	288
MEVC4-TLR2	-1.6	1.0	53	3	11	235
WP-TLR2	-1.6	0.98	54	7	13	254

### Molecular dynamics simulations of TLR2+MEVC-WP docked complex

**Fig 11:**
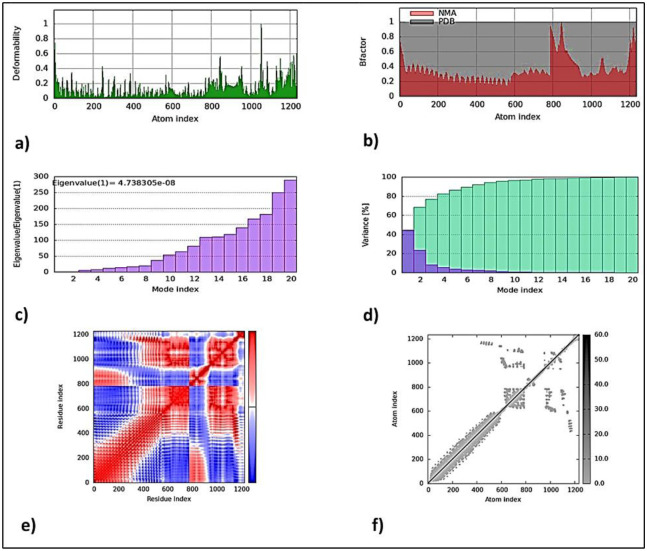
MD Simulation results of TLR2+MEVC-WP complex; **a**: Deformability map, **b**: Bfactor map, **c**: Eigenvalue graph, **d**: Variance map, **e**: Co-variance map, **f**: Elastic network model map.

The analysis of molecular dynamics simulations revealed observable differences at specific atomic intervals in the TLR2 and MEVC-WP docked complex. Deep insights into the docking analysis have been provided by molecular dynamics simulations, allowing for a more thorough comprehension of the complex interactions between two molecules. The Fig 11 shows a topographical representation with peaks indicating how flexible the docked structure is, with different residue sites showing different degrees of flexibility. These peaks are particularly prominent at the beginning of the atom index continuum. The B-factor graph’s dynamic fluctuations validate this observation, highlighting the relevance of normal mode analysis within the complex. The eigenvalue map, which shows how much energy is needed to deform a docked complex, exhibited eigenvalue score of 4.73×10−08, indicating the strong structural stability of TLR2 and MEVC-WP docked complex. On the contrary, the variance map which is inversely related to eigenvalues, is explained by means of a graphical representation that clarifies the cumulative and individual variances of amino acid residues. Three critical aspects that control the dynamics of the interaction between TLR2 and MEVC-WP docked complex are shown by the covariance map which comprised of the correlated (red), uncorrelated (blue), and anti-correlated (white) movements of component residues. The covariance map, in particular, highlighted strongly correlated motions in the parameter space from 0 to 1200 residue indices on both axes, interspersed with uncorrelated and anti-correlated interactions at various loci. Finally, the elastic network model explored the target structure’s pliancy, which was indicated by different grayscale intensities. In current perspective, this particular analysis suggested that the previously discussed correlated residues were highly flexible in the context of TLR2 and MEVC-WP docked complex. This proposed that the docked complex of TLR2 and MEVC-WP possessed significant levels of flexibility which would enable the docked structure to be stable upon certain fluctuations.

### Codon optimization and in-silico cloning

**Fig 12:**
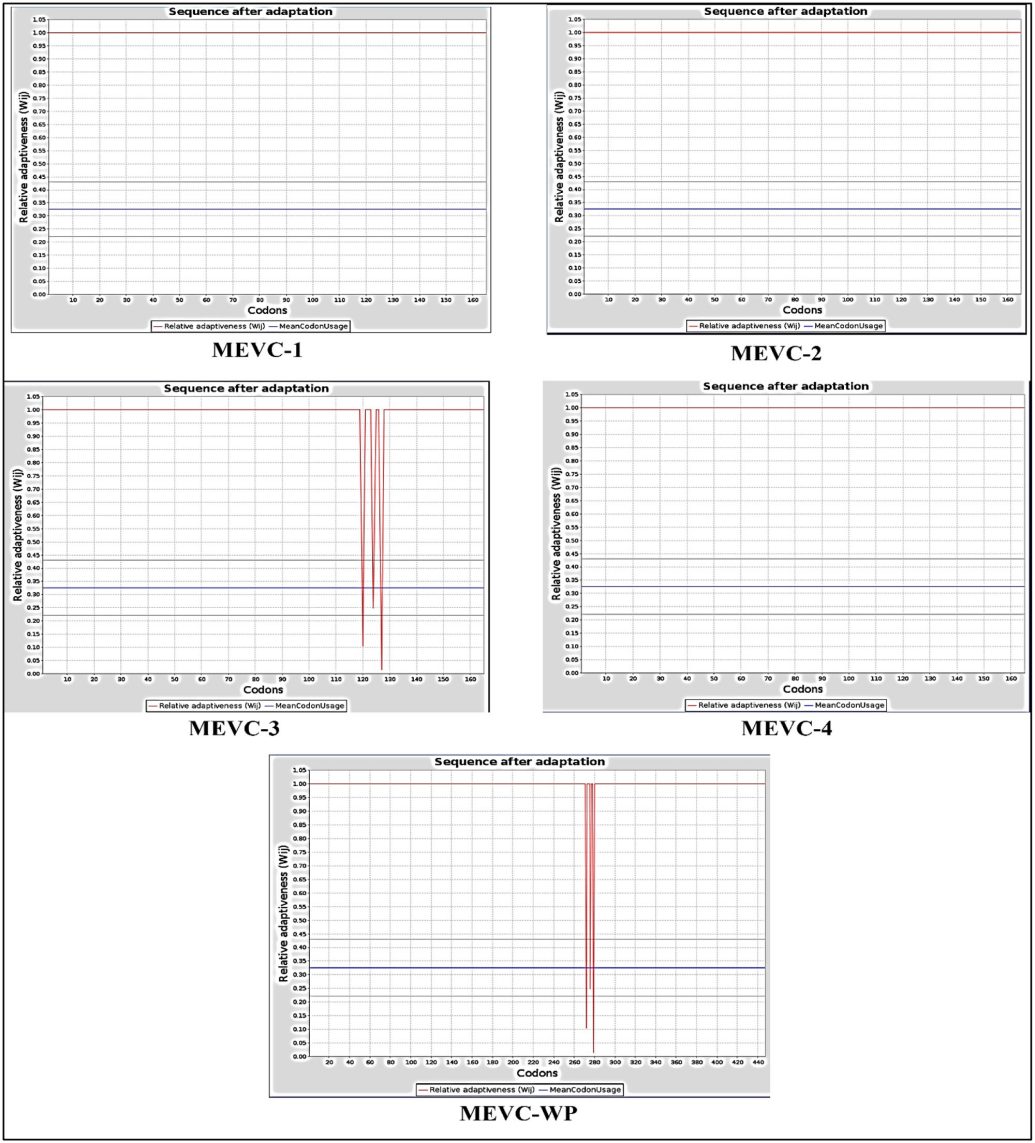
Codon optimization maps for all of the multi-epitope vaccine constructs including MEVC1, MEVC2, MEVC3, MEVC4 and MEVCWP.

Codon adaptation index (CAI) is a numerical measure in bioinformatics that assesses how well a gene or DNA sequence aligns with the preferred codon usage of a reference set, often the highly expressed genes in a specific organism. The relative adaptiveness of a codon is determined by comparing its frequency in the gene being studied to its highest frequency in the reference set. This comparison results in a ratio between 0.1 and 1, where 1 signifies a perfect match to the codon usage bias of the reference set. If the CAI value is higher, it means that the gene’s codon usage closely matches that of the reference set, indicating better adaptation or optimization for efficient translation. The JCcat server was used to optimize the expression of all developed vaccine sequences in the *E*. *coli*. K12 strain. The corresponding nucleotides for every vaccine construct were obtained by optimizing the codons and performing reverse translation. For every vaccine construct codon optimization index CAI values were also computed through the JCat server. The CAI values for MEVC1, MEVC2 and MEVC4 were 1.0. While, for MEVC3 and whole proteome-MEVC, the CAI values were 0.95 and 0.98, respectively. These values indicated high degree of relativeness between the codon usage of multi-epitope vaccine constructs and *E*. *coli*. The overall results produced by the JCat server were in the form of graphs plotted between relative adaptiveness and codons. In the Fig 12, y-axis of the graph represents relative adaptiveness of each codon. This metric indicated that on the basis of a reference set how well suited each codon was, in terms of its frequency of occurrence within the gene compared to the expected ideal frequencies. Higher values on y-axis represents the codons that were efficiently utilized and hence more adaptive in the context of translation efficiency and protein expression. Whereas, each point on x-axis represents a specific codon within the genetic sequence, being analyzed. The codons with relatively high adaptiveness are more favored in the gene sequence due to their optimal usage in the host organism’s translational machinery. Moreover, the higher adaptiveness also likely lead to efficient protein expression and proper folding, potentially impacting the functionality and yield of the encoded protein.

**Fig 13:**
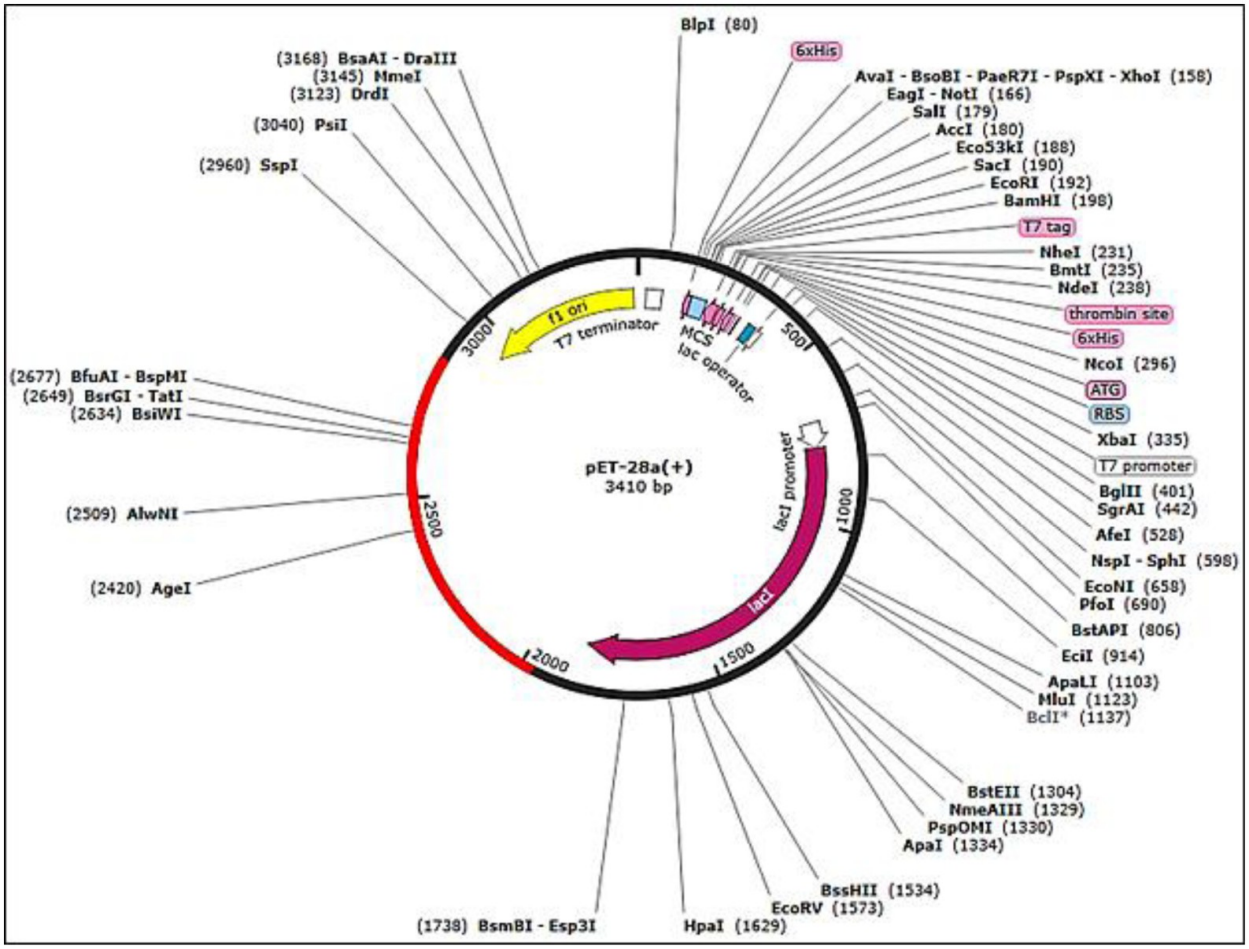
The pet-28a vector’s *in-silico* plasmid map displays vaccine sequences that were cloned, using XcmI and PsaAI as enzyme restriction sites. Additionally, it illustrates the cloned sequence of a designed vaccine to target the whole proteome which can be visualized in red color.

The high calculated CAI scores were in the range of 0.9 to 1.0 showing that vaccine protein was expressed at a high level in *E*. *coli*. While, all the percentages of the GC content that range from 52 to 55 was previously illustrated in the Table 7. Therefore, the corresponding nucleotide sequence was then employed to make the DNA fragment for *in-silico* cloning. Furthermore, SnapGene software was utilized for *in-silico* cloning. For this purpose, pet28a + vector was selected for cloning. The putative vaccine sequence was then inserted into the pet28a + by XcmI and PsaAI restriction enzymes of respective 5’ to 3’ ends which can be observed by red color in the Fig 13.

### Immune simulations of the final vaccine construct

**Fig 14:**
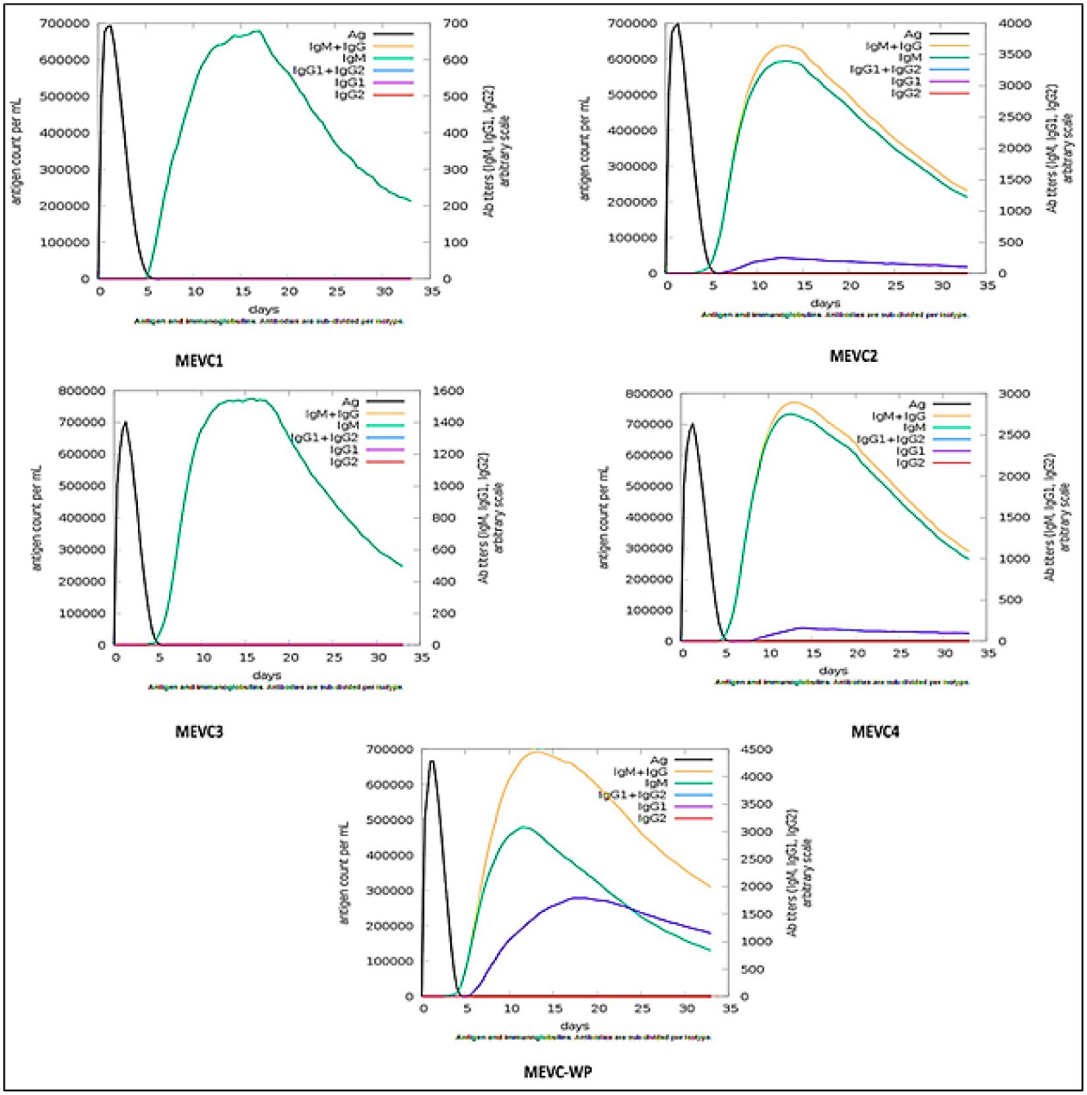
Representation of plotting the antigen count/ml/day against titers for each antigenic-designed vaccine in the immune simulation maps. The immune simulation maps for vaccines developed against the four distinct MEVCs (MEVC1, MEVC2, MEVC3, and MEVC4) as well as the entire proteome-based vaccine construct known as MEVCWP are also shown in this figure.

Each vaccine was analyzed as an injected antigen using *in-silico* immune simulations in order to assess the potential of antigen-based induction of immune response. In the Fig 14, the map showed that after the injection of MEVC1, it achieved high antigen count at the day 2 and then neutralized slowly till day 5. Then the antibody IgM achieved the level close to 700000 antigens count per milliliter between the days 7 to 20. For the second vaccine construct, it similarly achieved high antigen account at the day 2 and neutralized till the day 5. After this, the action of strong antibodies IgM and IgG achieved the antibody titer greater than 600000 antigen count per milliliter between the days 5 to 15 and then till day 30, it slowly neutralized to less than 300000. Along with this IgM, antibody also presented the figures of 600000 antigen count per milliliter. However, these variations were significant. The MEVC3 illustrated almost the same results as the MEVC1. The antigen count was 700000 on the day 2 and the IgG antibody titers was close to 800000 between the days 10 to 20 and then it neutralized slowly till the day 30. The MEVC4 displayed the map of antigen, IgG, IgM and IgG1. The antigen count was high on day 2 up to the 700000 antigens count per millimeter. IgM and IgG count showed the values, almost close to 800000 au/ml between the days 10 to 15. While, both of them exhibited slow neutralization till the day 30 up to the 280000 antigens count per milliliter. The IG1 antibody indicated the titers up to 400000 antigens count per milliliter from the day 13 to 30. For the whole proteome (WP) based vaccine construct, results exhibited that the antigen count was greater than 600000 on the day two and then this value continued to fall till the day 5. The strongest antibodies IgG and IgM represented the highest count up to the 700000 between the days 8 to 13 and then slowly neutralized till the day 30. The IgM antibody presented a count greater than 400000 on day 10 and then slowly utilized till day 30. Similarly, the IG1 antibody titer exhibited the graph close to the 300000 au/ml. These results suggested that the designed vaccine can trigger an effective immune response and possessed the potential to induce immunity. Hence, it can be proposed that designed vaccine candidates can trigger the immune response against the pathogen *Candida lusitaniae*. The results of all the immune simulations discussed above are illustrated below in the Fig 14.

## Discussion

This study primarily aimed to design a putative vaccine against *Candida lusitaniae* using a subtractive proteomics approach [60]. This method has been applied for successive systematic steps toward the identification of suitable vaccine targets from the pathogen proteome. First, the reference proteome of Candida lusitaniae was retrieved from the UniProt database and further analyzed using various web-based bioinformatics tools. Remaining extracellular proteins were analyzed with the CELLO tool to predict which may give an immune response. The authors of one similar study focused on computational design for a multiepitope vaccine against Langya henipavirus and targeted the surface proteins where antigens are likely to be expressed. They paid more attention to extracellular proteins because these are more accessible to the immune system and, subsequently, enhance the vaccine to provoke an even more aggressive response from the immune system. This approach stressed the selection of suitable protein targets toward the design of effective multi-epitope vaccines [61]. The identified proteins were subsequently subjected to BLASTp in order to distinguish between homologous and non-homologous proteins with respect to the host, *Homo sapiens* [62]. Phylogenetic analysis was conducted on the non-homologous proteins that may indicate a number of paralogs; however, this study did not find any paralogs. The next thing was to determine their allergenicity and antigenicity, which would allow the categorization of these proteins based on their ability to cause allergic responses. In the process, the group of proteins that showed high allergenicity and another group with no allergenicity led to the selection of the four proteins chosen for their further study and possible development as vaccines. Of note, a similar approach was previously reported in the design of a vaccine against *Candida auris*, where the authors used the tool CD-HIT to identify paralogs instead of phylogenetic analysis [63]. A somewhat similar study was conducted with the aim of finding vaccine targets for *Brucella melitensis*; the authors conducted a proteome-wide screening as done in this study. However, they incorporated additional analyses to complement their findings. The analyses included BPGA and the investigation of transmembrane helices, both of which provided further insight into the characteristics of the proteins relevant to vaccine development [64]. While these additional analyses were performed, it is interesting to note that most of the methods used in their study were very similar to those used in our analysis. This highlights the efficiency and flexibility of the proteome-wide screening approach in identifying potential vaccine candidates against several pathogens.

After selecting potential proteins, a further screening was done to predict a set of epitopes including CTLs, HTLs, linear B-cell epitopes, and discontinuous B-cell epitopes. Studying Helper T-lymphocytes, particularly, interferon-gamma-inducing MHC-II epitopes were identified with the help of the online tool IFN-epitope. The epitope screening effort here focused on searching for epitopes with high antigenicity and non-allergenic properties, an important factor in a vaccine candidate construct. A similar study was done to construct a multi-epitope chimeric vaccine against Human Papillomavirus [65].

The IFN-epitope server uses advanced algorithms to predict and design peptides that stimulate IFN-gamma, along with MHC Class II binders and T-cell epitopes. The predicted CTL, HTL, and B-cell epitopes were then concatenated using various linkers, including KK, GPGPG, and AAY [28]. These linkers are important for enhancing epitope presentation, allowing sufficient spacing between epitopes and avoiding incorrect folding, which in turn enhances the overall potency of the immune response. hBD-2, an adjuvant proven to be harmless to humans, was added to MEVC in order to enhance its immunogenic efficacy. In the final version of the MEVC, WP-MEVC utilized an EAAAK linker, with the latter incorporating the hBD-2 adjuvant, which is known to express itself autonomously at sufficient levels capable of mounting a strong immune response toward the antigen of interest [66, 67]. In a similar study, epitopes with an adjuvant were coupled using an EAAAK linker while designing a multi-epitope vaccine against Nosocomial *Proteus penneri*. This approach has overhauled the role of linkers in improving stability and immunogenicity of vaccine constructs [68]. This holistic approach led to the development of a whole proteome-based final multi-epitope vaccine, the WP-MEVC. Selection of these linkers was supported by a comprehensive review of the literature, and there are analogous methodologies considered by other authors in the computer-aided design of potential multi-epitope vaccines, especially those targeting SARS-CoV-2 variants [69].

Following the design of the whole-proteome vaccine construct, we analyzed its stability and viability using an online webtool, ProtParam. This tool enabled the examination of a number of physicochemical characteristics of the vaccine construct, including its molecular weight, theoretical pI, half-life, GRAVY, and stability index. Through the evaluation of these parameters, a thorough comprehension of the vaccine construct’s structural and chemical properties was achieved, which is essential for its efficacy as a vaccine. Comparable methodologies have been utilized in various studies pertaining to the in-silico development of multi-epitope vaccines targeting pathogens including *Porphyromonas gingivalis*, *Streptococcus gordonii*, and *Cardiobacterium valvarum*, as well as in the formulation of mRNA-based vaccines [70–73]. These studies, combined with our own research findings, further emphasize the importance of computational methods and algorithms in the design and optimization of effective vaccine formulations. By conducting an in-depth examination of stability and viability, we determined that our whole-proteome vaccine construct possessed the necessary physicochemical properties to be considered a promising and effective vaccine candidate. Commonalities in methodology among various research efforts emphasize that computational techniques are integral in advancing vaccines, as such approaches yield essential knowledge regarding both structural and functional nature of the designs of vaccines.

In addition to the immunoinformatics and simulation-based strategies employed for the identification of epitopes, we modeled the tertiary structure of all the multi-epitope vaccine constructs (MEVCs) using the trRosetta server, which is renowned for its ability to predict protein structures with high accuracy. These models were then refined using the online web service Galaxy Refine. Further manipulation of improved structures was done with BIOVIA Discovery Studio Visualizer, from which the best models were selected for detailed study and analysis. The above approach ensures careful examination and enhancement of structural properties of vaccine constructs and provides a sound framework for future experimental and bioinformatics studies [74]. Design 2.0 online platform-mediated disulfide engineering was used to enhance the stability of our vaccine formulation. We were able to incorporate strategically placed disulfide bonds into the formulation, which enhanced its structural integrity, leading to its robust resistance to degradation. It is expected that these disulfide bridges will greatly enhance the vaccine’s ability to induce a strong and durable response against the intended pathogen by preserving its optimal conformation. In the development of a multi-epitope vaccine targeting the hepatitis E virus, a comparable methodology of disulfide engineering was utilized to improve the stability of the vaccine that was constructed [75].

Additionally, protein-protein docking was carried out using the designed whole-proteome vaccine construct (WP-MEVC) in combination with Human Toll-like Receptor-II (TLR-2) through the HADDOCK server. This docking study has enabled us to explore the interactions occurring between the vaccine construct and TLR-2 receptor-a crucial receptor in triggering the immune response. We got a top-ranked docked complex based on the docking score in PDB format for further studies. The particular interactions between the WP-MEVC and TLR-2 were investigated with the use of PDBsum, which provided useful data regarding different interaction types: hydrogen bonds, non-hydrogen bonds, and salt bridges. In this way, the methodology is connected to previous studies dealing with Bartonella bacilliformis, where a multi-epitope subunit vaccine was designed, and docking experiments with TLR-2 were accomplished [76]. Similar docking methods have been reported in studies that focus on the design of multiepitope subunit vaccines against *K*. *aerogenes* [77] and MERS-CoV [78]. Such approaches underscore the impact of protein-protein docking as an integral part of vaccine development, helping provide insights into the interactions that may enhance the immunogenic nature of vaccine candidates. By establishing a strong interaction between the WP-MEVC and TLR-2, we aim at increasing the capability of the vaccine to provoke a strong immune response.

Moreover, for the expression in the E. coli K-12 strain, all vaccination sequences were optimized using the JCat server for optimal expression. The codons for each of the vaccine constructs were optimized and reverse-translated into their corresponding nucleotides, which were then used to synthesize fragments for cloning purposes [79]. Sequences were in *silico* cloned in the pET28a+ plasmid using the SnapGene tool, with the assistance of XcmI and PsaAI restriction enzymes. Similar cloning strategies have been reported in studies that pursue a vaccine against Pegivirus; in these cases, in-*silico* cloning was performed using the pET28a+ plasmid, which points to the suitability of the aforementioned vector for vaccine construct expression in E. coli. [80]. In addition to codon optimization and cloning, we also explored each one of the developed vaccines as injectable antigens through in-silico immune simulations.

The simulations conducted assessed the potential antigen-mediated human immune response activation, and the results showed that the designed vaccines may provoke a high immune response and have the potential to induce immunity. Another previous study engaged in the design of a novel multi-epitope vaccine against HCV infection also included in silico expression in E. coli, which was an important input for optimizing the vaccine construct to be expressed in a bacterial system and subsequently experimentally validated, and maybe produced [81]. These findings align with a reported investigation that highlighted a similar approach in designing a peptide vaccine to enforce a humoral immune response against *Campylobacter jejuni* [82]. By optimizing codon usage and performing in-silico cloning in the pET28a+ plasmid, we have ensured that our vaccine constructs can be effectively expressed in *E*. *coli*, combined with in-silico immune simulations, further supports their potential to induce potent, protective immune responses targeted against the indicated pathogen.

## Conclusions

In this study, the subtractive proteomics approach was applied to accurately screen for potential protein targets suitable for vaccine design against *Candida lusitaniae*, a pathogenic fungus. Through this method, we identified four potent protein targets that could serve as effective candidates for vaccine development. For each of these target proteins, various computational techniques were employed to identify epitopes recognized by cytotoxic T lymphocytes (CTL), B cells, and helper T lymphocytes (HTL). These epitopes play crucial roles in eliciting immune responses against the pathogen. Additionally, we conducted in-depth analyses, including 3D modeling and evaluation of physiochemical properties. These analyses, along with consideration of other pertinent parameters, facilitated the development of a multi-epitope vaccine construct, aimed at maximizing immunogenicity and efficacy. Subsequently, the designed vaccine candidates were subjected to immunogenic analysis using immune simulation maps. The results from these simulations revealed significant levels of immune responses elicited by our vaccine candidates, suggesting their potential effectiveness in combating *Candida lusitaniae* infections. Based on our findings, it can be concluded that the designed whole proteome vaccine construct holds promise in inducing robust immune responses against *Candida lusitaniae*. This research represents a significant step forward in identifying epitopes for future vaccine development against this pathogen. However, further experimentation is warranted to validate the efficacy of the vaccine construct in real-time settings. Our work provides valuable insights and directions for future endeavors aimed at developing effective vaccine against *Candida lusitaniae*.

### Limitations of this study

This vaccine is designed on computational basis in which we targeted specific proteins and selected their epitopes on the basis of *in-silico* analysis. So, there is need for the experimental validation in wet-lab experimentation. All of the properties including the physiochemical, antigenic, allergenic, for the design of vaccine construct further require the experimental procedures so that it may lead to design a real-time vaccine using the recombinant DNA technology. Furthermore, using this multi-epitope vaccine construct against *Candida lusitaniae* as a potential therapeutic, it will be necessary to justify the clinical illustrations by incorporating various experimental approaches including the expression and the purification of MEVC’s.

## Supporting information

S1 File Amino acid sequence retrieved from UniProt. Online web servers incorporated in this research work.
(DOCX)

S2 File Reference proteome of *C*. *lusitaniae*. (DOCX)
