# Supplementary material for: Utilizing the subtractive proteomics approach to design ensemble vaccine against Candida lusitaniae for immune response stimulation; a bioinformatics study
Source: PLoS One. 2025 Feb 6;20(2):e0316264. doi: 10.1371/journal.pone.0316264 (PMC11801629; doi:10.1371/journal.pone.0316264)
Supplement: S1 File — Online web servers incorporated in this research work. (DOCX) [file pone.0316264.s001.docx]

**Amino acid sequence retrieved from UniProt:**

The reference proteome of *Candida lusitaniae* is included in the supporting information file 2 (S2).

**Online web servers incorporated in this research work:**

- **UniProt** (<https://www.uniprot.org/>) for amino acid sequence retrieval
- **CELLO** (<http://cello.life.nctu.edu.tw/>) for sub-cellular localization prediction
- **BLASTp tool** (<https://blast.ncbi.nlm.nih.gov/Blast.cgi?PAGE=Proteins>) for protein BLAST analysis
- **MEGA** (<https://www.megasoftware.net/>) for sequence alignment and the construction of phylogenetic tree
- **VaxiJen tool** (<https://www.ddg-pharmfac.net/vaxijen/VaxiJen/VaxiJen.html>) for the estimation of antigenicity of the selected proteins
- **AlgPred** (<https://webs.iiitd.edu.in/raghava/algpred/submission.html>) to estimate the allergenicity status of the selected proteins
- **NetCTL 1.2** (<https://services.healthtech.dtu.dk/services/NetCTL-1.2/>) to predict the cytotoxic T-lymphocytes (CTLs) epitopes
- **IEDB** (<https://www.iedb.org/>) for the prediction of Helper T-lymphocytes
- **AllerTOP v.2.0** (<https://www.ddg-pharmfac.net/AllerTOP/>) to determine the allergenicity of MHC-II epitopes
- **IFN-epitope server** (<https://webs.iiitd.edu.in/raghava/ifnepitope/predict.php>) to analyze the interferon-gamma triggering MHC-II epitopes
- **ABCpred** (<https://webs.iiitd.edu.in/raghava/abcpred/ABC_submission.html>) to predict the B-cell epitopes
- **ProtParam** (<https://web.expasy.org/protparam/>) for physiochemical properties analysis of the selected vaccine candidate proteins
- **SOPMA** (<https://npsa.lyon.inserm.fr/cgi-bin/npsa_automat.pl?page=/NPSA/npsa_seccons.html)> for the prediction of secondary structure of all the selected proteins
- **trRosetta** (<https://yanglab.nankai.edu.cn/trRosetta/>) to predict the three dimensional (3D) models of the selected vaccine candidate proteins
- **Galaxy Refine** (<https://galaxy.seoklab.org/cgi-bin/submit.cgi?type=REFINE>) for structural refinement of the predicted models
- **ElliPro server** ([ElliPro: Antibody Epitope Prediction (iedb.org)](http://tools.iedb.org/ellipro/)) to predict the discontinuous B-cell epitopes
- **Disulfide by Design 2** ([Disulfide by Design) (http://cptweb.cpt.wayne.edu/DbD2/)](http://cptweb.cpt.wayne.edu/DbD2/) for disulfide engineering of the final vaccine construct
- **HADDOCK 2.4 server** (<https://wenmr.science.uu.nl/haddock2.4/>) for protein-protein docking of the vaccine construct with the human toll-like receptor 2 (TLR2)
- **iMODS** (<https://imods.iqfr.csic.es/>) for molecular dynamics simulations of the vaccine-TLR2 docked complex to estimate the structural stability and flexibility
- **Java codon adaptation (JCat) tool** (<https://www.jcat.de/>) for codon optimization analysis
- **SnapGene** (<https://www.snapgene.com/>) for *in-silico* cloning
- **C-ImmSim** (<https://kraken.iac.rm.cnr.it/C-IMMSIM/>) for immune simulations of the vaccine construct

**The complete methodology and workflow followed in this study:**

**
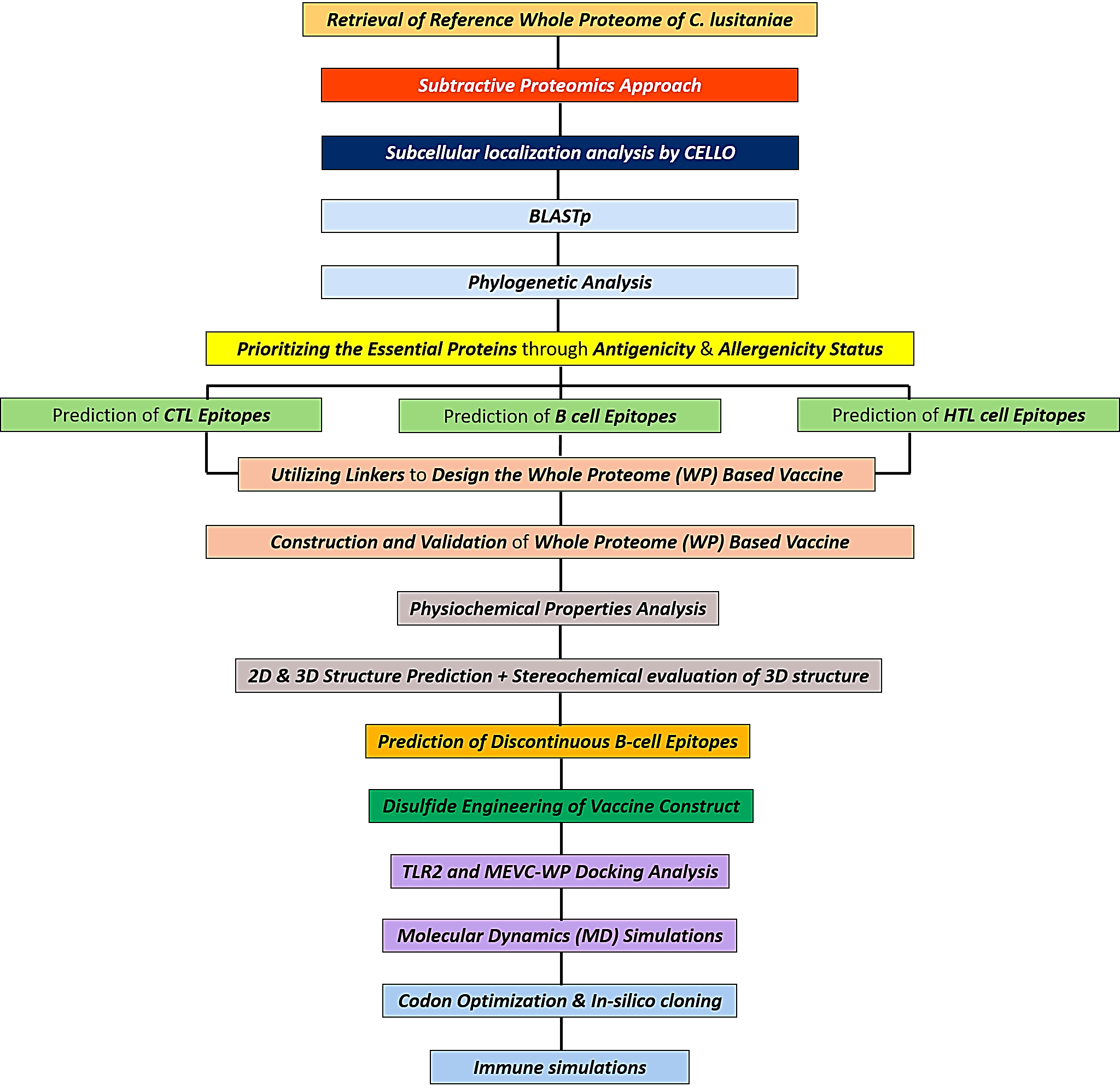
**

- The reference proteome sequence of *Candida lusitaniae* given in S2, was the initial and major source of producing further data during this research work because it was further subjected to various types of analyses. In this regard, various web servers were utilized in this work in a specific order so that the results of the previous tool could be proceeded further as an input of the subsequent online web servers.
