# Supplementary material for: Utilizing the subtractive proteomics approach to design ensemble vaccine against Candida lusitaniae for immune response stimulation; a bioinformatics study
Source: PLoS One. 2025 Feb 6;20(2):e0316264. doi: 10.1371/journal.pone.0316264 (PMC11801629; doi:10.1371/journal.pone.0316264)
Supplement: S2 File — (DOCX) [file pone.0316264.s002.docx]

**Reference Proteome of *C. lusitaniae***

>sp|C4Y9D5|ARO1_CLAL4 Pentafunctional AROM polypeptide OS=Clavispora lusitaniae (strain ATCC 42720) OX=306902 GN=ARO1 PE=3 SV=1

MSQVEKVSILGSDSIHVGYGIQDHIVEETLTNLKSSTYVIITDSNMEATAPYQTLSSKFE

AGLKEFRPESRLFYYAVSPGENNKSRETKAQVEDFLLQKGCTRDTVIIAVGGGVVGDMIG

FVAATFMRGVRVVQVPTTLLAMVDSSIGGKTAVDTPLGKNFVGAFHQPKYVFVDVSFLTT

LPTRQFINGMAEVVKTAAIWNEEEFTRLENFAKTFIAVVTSDNIDLATIKDDLVKTVLES

IRVKADVVSADEKESSLRNLLNFGHTIGHAIEAIVTPQALHGECVAVGMVKEAELARYWG

VLSPVAVARLVNCIAAYNLPTSVGDKIFVQRIGHKRHFIQINTLLEKMAIDKKNDGSKIR

TVILEAIGKCYQLKAHEVSKQDLSFVLTDETLVHPFQEETTPKENVVIPPGSKSISNRAL

ILAALGKGTVRIKNLLHSDDTKHMLAAVAALKGAEISTEDNGDTIVVKGNGGNFITCDEQ

LYLGNAGTASRFLTTVASLVNVNEQSNDYTVLTGNARMQERPVGPLVNALRANGSEIEYL

NNEGSLPLKIKAGKGLKGGRVELAATISSQYVSSILMCAPYAEKEVTLALVGGKPISQLY

IDMTIAMMKDFGISVTRDPHEEHTYHIPKGVYSNPGVYEVESDASSATYPLAFAAMTGTS

CTVPNIGSSSLQGDARFAVDVLKPMGCTVEQTSTSTTVRGPSKGSLKPLTHVDMEPMTDA

FLTASVVAAIANSSVPTQITGIANQRVKECNRILAMVDELAKFGVRAEELPDGIEIYGID

YKNLKVPSLENRGVCTYDDHRVAMSFSLLAGMCPEPVLITERSCTGKTWPGWWDVLHTKF

GVELEGYEEPKDLNDPALLVNKEVNGDKSIVVIGMRAAGKSTLSRWIADFMGFELIDLDT

VFEQTHGDIREYIKANGWGRFRELEAGIMKEYLTKCSSRHVISTGGGIVESEESRDILKS

YTKTGGIVLHLHRDLDETIVFLSSDTTRPAYVSEIKDVWARREKWYHECSNYHFYSSHCG

TEEEFKKLRHSFVSYLKTITGAGLSPVPKGRSFVLSLACSDLNDIAENLEDIVAGCEAIE

LRVDLLKDYSPSFVADQTAVLRKFVNLPIIYTIRTKGQGGNFPDEDVQALEQLSYLGIKL

GVDYLDVQLSNSEKFVKSIIEKKAFTKIIATHIDLAGLPWTRAEWDNKYNQGISLNADVI

QLVGFAHAFQDNIDLEQFRANHTITPLIAFNAGEHGKLSRVLNRTLTPVTSELLSNVSGN

GQLTVGEINRCFSEIGGLSRRNFYIVGNPISHSRSPQLHTAGYEKLNLPHRFSKFETDYA

QKVYEEVMTKPGFGGLAVTIPLKLDIMKYVSELSESAKIIGAVNTVTPLDGQPGKFYGDN

TDWYGITQSFVRHGVPSFGNSSVNGMVVGGGGTSRAAAFALHQMGCKKIYMVNRTTSKLH

EIKSSLPSDFNIEVLETVDQVEAADPVSLVVSCVPADKPLDSELLNKVERILYHGKNQEK

RSFVPTLLDAAYKPRVTPIMKIAEEKFGWAVVPGVEMLVNQGVLQFKVHTVFTPPYKNVY

EAVVDDNV

>tr|C4Y0R6|C4Y0R6_CLAL4 Phosphatidylserine decarboxylase proenzyme 2 OS=Clavispora lusitaniae (strain ATCC 42720) OX=306902 GN=PSD2 PE=3 SV=1

MRIKKRKADLYLLVNALRAADLVIPGDEKPSKSINAAFVVQLNKYKKRSKRRLNTSQPSW

DRQFAVPLKHGDYSQVLVLSVWSKSSREKSYLGELRLCIADLFIDQEAHKTEPQWYKLYS

DETEHSYVTGSVLLSFELLAPKQRRMNMSSGSNVSLTKENPKVPSLAVNPPTATQLKLSK

LNIKDDTIDEMFKKWICSLVVSSVNPATITPNEQGFYPSDFASADISGASEMSEVESSDD

ETKVTSKRLSHSEEPLQLLHDAQSKSLFMELGQTKHLDVPGSIDDVRSDVSVSSASSSCS

YVSDAYSDGGLYNEGGGEAEPKGRKHRRKRRASEKATRKPKYELRKRDVKGILFVEIISC

SDLPPFRNFTRTTFDMDPFIVVTFGKKTFRTSWKRHTLNPVYNERLVFEIMEHESNYNVQ

FSVLDKDHFSFHDKIADVTLPVQDLMNIASVPTKIAIKEPDDDLSNSELLESKDTDTSHH

SNEASGGDEEEFKPSEHLNVKLGNITEGYRNASFSSFGSPSDNLTSNSVIKLAENENLVK

VKKRKKLRRSRYTTSYVDTSLFKTIDLSLTLHKTKWAEKYSPTLKLRARYLTYESLRRDF

WRILLEQFTVSDTPGTMDYIELMSLLDTLGAEDSDQIVGKFFEKAKKSTWGGDSLDFNEI

IDCLEDLVTSESQSPGAKIFVFDRCPICCQEHLSKRDDLDIITHFAICASKDWSIVSKLL

VSSYDTPQIATRRWFSKILIKLSYGKYKLGSNSANILVQDRSTGIVMEEKMSVSVRLGIR

LLYKGLDKAKSKRIRTLLRKLSIKQGVKFDSPRSKRDIDSFIKFHKLNLADCLITDPSRF

ETFNDFFYRKLKPGARPIEGDDGIAVSPADCRCTTFTSVDEATELWIKGRNFTLAKLFNG

NFNDLEKTSLYNPKECCIGIFRLAPQDYHRFHSPVNGKIGPIKYIEGEYYTVNPMAIRSD

LDVYGENVRVVVPIVTETFGTVVLVGVGAMMVGSTIITVKEGQEVQRGDEIGYFKFGGST

VLLLFEKKYLQFDSDIVNNSKSCIETLVRVGQSIGHSPSVKEYKREHIEFDKQPQTFKLN

LIRAITGGDVSDPAKIDSWESQNLQITADDMDDLINDPELADYLTDEEGSIDSQ

>tr|C4Y4Q6|C4Y4Q6_CLAL4 NAD-dependent protein deacylase OS=Clavispora lusitaniae (strain ATCC 42720) OX=306902 GN=CLUG_02628 PE=3 SV=1

MSTQLSEFQECLRSSKRIVALVGAGLSASSGLATFRGSQGLWKNFNMIDLATPDAFYIDP

GLVWQFYSWRRYEALKAQPNAGHLALARLSRLRSKKFITITQNVDGLSARGGHDPKTLYE

IHGSLFELRCTSFTCTHVERGNTDVPLTPALTIDVDTEVGPSVTTATTSSASVSSNSTAT

TTATSSSPFFSPTKELNVDDLPKCPVCQSLMRPGVVWFGESLPLRVLDRIDTFLEAGPVD

LILVIGTSGTVYPANSYVDIVRQRGGSVAIFNTDIDQDVLEGKVAKTWGFKGDAAELLLI

ALSPLFEDSEEANS

>tr|C4Y5I8|C4Y5I8_CLAL4 Fatty acid synthase subunit beta OS=Clavispora lusitaniae (strain ATCC 42720) OX=306902 GN=CLUG_03422 PE=3 SV=1

MTTTHRPFSLSHGSIEFTTLVPTNLFFKYSQLKESFAKTLPVATEGFASDDEPASPAELY

AKFVGFTASLVDPDSPGEFSEILALVLQEFESGFFPESDIHTFAAQLLADETYPTTSLKV

KGVIKNYFDAVIRANAEIPKRSSELLKASGAKLAKSFAIFGGQGNTDDYFEELRELNHMY

HGLITDLLAQVASRLSGLVKTTENVDKIYTQGFDILNWLSSPDQTPDQDYLLSVPVSCPL

ICVIQLAHYVVTCKTLGVSPGEFRDHLVGATGHSQGLVTAVAIASSDSWDSFYENALKAV

TLLFFIGSRCLMAYPRTTLPPTMLQDSLENGEGRPSPMLSVRDLSREQVEKFIATTNEHL

PADKRVAISLVNGGRNLVVSGPPESLYGLNVTLRNNKAPSGLDQARIPHSERKLKFSNRF

LPIFAPFHSHLLEPATDMILADIEAEKLAFSAKDLRIPVFDTYSGENFQESAGDVTDRVV

QCITQLPVHWEQATAFEATHLLDFGPGGVSGVGVLTHRNKEGTGARVIIAGALDNAVDDD

YGFKQELFNRTAGSIKWAPNWLNEYKPKLAKNSAGKVYVDTKFSRLLGRAPLMIPGMTPT

TVNTEIVSAAINAGYHIELAGGGYFHAAGMEQALKEVSEQIKPGCGIGINLIYVNPRMLQ

WGIPLIKELRDKGFPIQSLAIGAGVPSLEVATEYIETLGLTHLGLKPGSIESINAVITIA

KAHPTFPIVLQWTGGRGGGHHSFEDFHQPILQMYAKIRKCSNIVLVAGSGFGSDEDTYPY

LTGSWSAKFKYPPMPFDGVLFGSRVMTAKEAHTSLEAKQLITQCTGVSDEQWESTYKKPT

GGIITVRSEMGEPIHKIATRGVMLWKELDDTIFNLPKNKMLEALAKKKDYIIGKLDKDFQ

KPWFARNASGVCDLEDMTYQEVANRMIELMYVKKHARWSDISLRNFFGDFLRRVEERFTT

KAGQESLVQNYAQLTSSPQEFTDKFFESFPAAKEQLISEEDCDFFLLCCARPFQKPAPFV

PVLDERFEFFFKKDSLWQSENLETVVDEDVQRTCILHGPVAAQFTNKVNEPIKEIMDNIH

EGHIAKLIKDEYNGDASKIPVVEYFGGKSPVALKKVSGVVVEESGSTVTYKIGSIVPDKQ

EWLDLLAGPKLNWLQALISTDRIVQDKNFTSNSVHDVLSPAANITVTVENYDDAKKTKLS

VYEAVQGDMKQVVEIKLNKDSLIELSLIEHRTADGKAVALPFLYTYKPEDGFAPIVEVME

GRNNRIKEFYWKLWFGHSVPVDFDINVNEVIPGDEVTVTGKDISEFTHAIGNTCEAFVPR

PGKQTLAPMDFAIVVGWKAIMMAIFPKTVDGDLLKLVHLSNGYRMVPGAAPLKKDDVVTT

NAVINAVLNQPSGKMVEVVGTIYRDSKPVMEVTSQFLYRGEYVDFENTFQKVSEDPVQVA

LKSAKDVAILRSKEWFHLEENIDLLGKVLTFRCNSTYYFKSAQVYSEINTTGDVYLELPT

KEVIRVGVVEYEADVSYGNPVTDYLARHGNTIEQAVKFENAIPISSGEELTSKAPSTNEP

YAKVSGDYNPIHVSRVFAAYARLPGTITHGMYSSGSIRALVEEWAANSIASRVRAFKANF

VGMVLPNDQLQTTLEHIGMINGRKIIKVETKNIETDAPVLVGEAEIEQPVTTYVFTGQGS

QEQGMGMDLYNSSEVAREVWDKADRHFVNNYGFSILDIVKNNPNELTIHFGGAKGRKIRE

NYISMMFETIGEDGEIKSEKIFKDIDHTTTSYTFMSPTGLLSATQFTQPALTLMEKASYE

DIKSKGLVPSDVMFAGHSLGEYSALSSLANVMPIESLVDVVFYRGMTMQVAVPRDELGRS

NYGMCAVNPTRVNPTFNDAALRFVVDEVSAKTGWLLEIVNYNVENTQYVTAGDLRALDTL

TNVLNVIKMNKIDIVKLQQQLSLDEVRDHLKEIISEVSAQSLAKPQPIDLQRGFAVIPLK

GISVPFHSSYLMSGVKPFQRFLCKKIPKSSVKPQDLIGKYIPNLTAKPFQITKEYFQEVY

ELTKSDKIKSIIDNWESFEKA

>tr|C4Y636|C4Y636_CLAL4 Inosine triphosphate pyrophosphatase OS=Clavispora lusitaniae (strain ATCC 42720) OX=306902 GN=HAM1 PE=3 SV=1

MSTITFVTGNANKLKEVLYILGGNNDSNTVGKFNIVNRSLDVEEIQGTIDEVTIHKAKSA

AQLIGGPVLVEDTCLAFDALNDLPGPYVKWFLKAIGLRGLVDMLYKFDDKGAKAVCTFGY

CEGPGKEVKLFQGITKGTIVESRGPQDFGWDSIFEPNGFTETYAEMEKATKNSISHRYKA

LEKVKTFLLEQ

>tr|C4Y6W4|C4Y6W4_CLAL4 Kynureninase OS=Clavispora lusitaniae (strain ATCC 42720) OX=306902 GN=BNA5 PE=3 SV=1

MASITTYTMNTKEEAQRLDSEFSSHRDKFVIPTFQSLGISNTEFRPSDSSIYLCGNSLGL

MPRSTKKAITDELDAWGERAVESHFNHPGKNASKTSWVDIDLPLVPLLAPIVGALNNEVA

VMGSLTANLNALLMSFYKPKGTKTKILFEKRAFPSDYYAFLNMVMLHGFDESHLVQIEVP

EGETFLETDLILQHIETYKEELALVCLPGIQYYTGQFFDIETITKAAHSHNIVVGWDLAH

AVGNVPLSLHDWGVDFAAWCSYKYLNSGPGAIAGIFVHEKHTKENSTSNFTPRLAGWWGN

NASDRFKMLEIFDPMQTALSYRQSNPSVIDCVALKASLEIFQEVGGVNELRTKSIQLTKF

MERLLTSSRFYLQPGADKAQFGFKILTPLKAEERGCQLSLLFQPHHDDPAHDVMEQVFSY

LHKNAIICDERRPDVIRLAPVPLYNSFHDVYIAIKRLEEALNHIEK

>tr|C4Y7M2|C4Y7M2_CLAL4 Phosphatidyl-N-methylethanolamine N-methyltransferase OS=Clavispora lusitaniae (strain ATCC 42720) OX=306902 GN=CLUG_04200 PE=3 SV=1

MKIEQLTHLASHFLDYVQWTPSLKFAVATISFNPIFWNIVARLEHRTHFLTKLAGGAYNG

CYLLAATIFSLGIYRDHVYHGALLAQPTYLPMVESGLVKALAVAAFGVGNVLVLSSMWAL

GVTGTYLGDYFGILMKERVTSFPFNVNDNPMYNGSTLCFLGTALWYGKPAGLLVTAFVFV

MYKIALFFEEPYTAKIYAHRNKTE

>tr|C4Y7R5|C4Y7R5_CLAL4 Sulfate adenylyltransferase OS=Clavispora lusitaniae (strain ATCC 42720) OX=306902 GN=MET3 PE=3 SV=1

MPIPVPHGGKLNDLVARDSAIKQDLLESIQNEKLPSLTLTERQLCDLELILNGGFSPLEG

FLNEEDYNSVVENMRLSSVKGDDGKGLLWPIPITLDVPEETARTFQPGAKIVLEDLRDQK

PLAILTVQSIYKPNKANEAEKVFRGDPEHPAVKYLFDTAGDIYIGGSIQGLNYPTHYDYV

SLRKTPAELRAEFERLGWDQQKIVAFQTRNPMHRAHRELTVRAAQDIGEDGHILVHPVVG

LTKPGDIDHHTRVKVYQQILKKYPEGLATISLLPLAMRMGGDREALWHALIRMNYGVDHF

IVGRDHAGPGKNSKGVDFYGPYDAQELLASVESELSPKIKIVPFRMVTYLPDEDRYAPID

TIDTKKVKTANISGTELRQRLRDGTEIPEWFSYPEVVKILRESNPPRSSQGFVIIIDSTG

SGQNDDYLAYALQSTLNQFAGGRRITRLNSSHNDAFLINELVKAGSGVIYTAKDDYGEIL

STVGEANSVVIKTGEKAVSGNTFSLEKAGDEESVRAILDAISQYLKEQGFYL

>tr|C4Y8E5|C4Y8E5_CLAL4 Phosphatidylserine decarboxylase proenzyme 1, mitochondrial OS=Clavispora lusitaniae (strain ATCC 42720) OX=306902 GN=PSD1 PE=3 SV=1

MVYRPALKPFTLKSKPYPHASFVHGPSAMNISQPLSRASTVAQINVQRSFHESSPDKGYR

GSYILPRIPRPKRSVFYYTTWTRNSKKLKIPGLSLSKRPFSSAPTQIKKKTQGIRRRFFS

AEARRKRKEKRMFVRWWTLTSIAVVLGGVAAKIRYEKAIHDQDDDDEFFSSDTIRPQSWH

LYAYSTLPLKAISRLWGQVNSIDLPVWLRSPSYRLYSAIFGVNLDEMDEPDLTTYNNLSE

FFYRKLRPGVRPIADSDLVSPADGKVLKFGVIENGEIEQVKGMTYSIDALLGLSAKKLAA

PTHSLEFDYDASHEAVIKRDEEFARLNGITYSLDDFLGGESEETYHLSQLEYKDKGDGTA

KGAKPSMQKDLSVAQNLAPTPLERLNLSKHNNLYFAVIYLAPGDYHRYHSPTNWVTTLRR

HFIGELFSVAPFFQKTLQGLFILNERVALLGYWKYGFFSMIPVGATNVGSIVVNFDKDLK

TNDVYEHEIYRQRSPSPGLSDDERTPLLKSDSSSSIDSVSSDKPKRLRKNTVYEATYTGA

SRLLGGFPLSKGEEVGGFKLGSTVVLVFEAPDNFHFNLEVGQKIKMGESLGDFA

>sp|C4YBJ8|FEN1_CLAL4 Flap endonuclease 1 OS=Clavispora lusitaniae (strain ATCC 42720) OX=306902 GN=FEN1 PE=3 SV=2

MGVKGLNQLIKEHAPDAFKEYQLKNLFGRKVAIDASMCLYQYLIAVRQQDGQQLTSEDGE

TTSHLSGMFYRTIRLVESGLKPMYVFDGKPPVLKGGELEKRLLKRQDALKQIEDLKETGT

VEELMKYEKRTVRASREQNDEAKKLLELMGIPYIVAPSEAEAQCAELARAGKVFAAASED

MDTLCYEPKYLLRHLTVAEARKMPIDQIDYEAMLKGLDMDRSTFVDLCILLGCDYCETIK

GVGPVTAFKLIKEHGSLDNIVKWIQENPEKTKYKVPENWPYDEAKQLFMNPEITKGDEVD

VKWNEPNVDGLVEFMVKQKGFSEERIRSGAEKLKKALKGGVQGRLDGFFTVVKSSPAKRK

PDAKDAKGKGKKKAKR

>tr|C4XVR2|C4XVR2_CLAL4 Cysteine proteinase 1, mitochondrial OS=Clavispora lusitaniae (strain ATCC 42720) OX=306902 GN=CLUG_00029 PE=3 SV=1

MSSFSVSNLSSWTEEFSQDLNTKLGASVLTNYNAEEALIDRATAIKNTTNVFNTSVSVEG

APVTNQRSSGRCWLFASGNVLRLPLMEKLSLKEFQFSQSYWFFYDKLEKCNFFLEKFVEN

IDSSPEEGVESRLNDFLLTDPTCDGGQFEMFINVAEKYGMIPHELYPDAYSATASRTLNF

LLKTKLREYAETLREAKRGNASQLPKLREEMQKELYRIMVMFLGQPPLPTDELVWEYKTK

NDEVKSMKLTPMSFYKETLGLDLTQWVSLLNDPRNPYDTIINIDKLGNVVGGKEVSYLNV

AIDDLAQYAVERIKSNNAVFFGTHTPIYMDKKRGIMDEKLYNYHLVNFNVTQDKASRIKY

KQSLMTHAMVLTAVHLDENGSPIRWRVENSWGKDSGQNGYYVMDHQYFKDYVYQIVVSKH

ELQNHHVKIFEDKANAVVLPPWDPMGALAILP

>tr|C4XVR9|C4XVR9_CLAL4 Autophagy-related protein 2 OS=Clavispora lusitaniae (strain ATCC 42720) OX=306902 GN=CLUG_00036 PE=3 SV=1

MYSQWIPQNLQKRLLLYILQQLSLFSEIDLPNLEEVSLNTIHLKDVLIDSDKVGKLRGCN

VRYGKLRNVELSGGMVGGVNFEIDGAEFVVALNLDSLETHQKDVSTLLAQSTADLASTIM

FENKGEPDFANESKSDSESSNEGKSPSSGNPKKSAFSGMMSRAVEIALSRLQVTVKNISV

KFVSEAADLVLYVKEFQFHSNNGCRHVSVKGISLAVARPNINPGHRNDAKETTKDKTGSE

KYTEDDHETSDDHSTSKDSSGTRSDTDEDEKSDSNAYDDGSLSNSMVFSHEEASSIYMSA

VSQSMKNDKLRPSEPIISSNETILLYVDDIHVKFDGLNPASNLSLDIQSVRIAAVPLMPT

ASLIFNTISKMLKLKNHNLKKQNRANRRAHSPHPSFPQYEVTSDEVPTENDEEENFTDLM

FNKLHVSEIIICLTSAISSTGEIASCSDDINIMFQNLNIKQKNKDLIYGGVEKFRILNYS

GGKESVLFEFDSSEISSSRTDSDKVPHSSPPPNGPRSKADIRFEIIQKFTNSQHSRETTA

LLSKNAIIKVDCDSLQYLTNFFTSLKTVYESVVSMMADLSLMRSLNEGKKVGTENQKSTS

NLLLLQTSSITSTVILSSSKLIKIVVLPILYNKADDQLSIQRVMVSLVSGGVETPFFSIP

SVALKTTIQSFKSFYLKKTNSIPHCSNLQSSNTLSCGEIQGTIDFKTLLSFYQGITSFVK

QFSAAIKCKVNALPESLADSPSQSPKAFSTAGLASSIYSKHSRFGRFKAKNRDHFHFGEQ

RSISSFRFMVQSLKLSVTSLFSRFGDLHIALDHFEFNLQEDSINGFVNDLSIERRFENQS

KQPFFHQLSKTTESPIVLFSHKTSDKSTSTDIILRKFCLEYYTQWLQLIEKNVTQGHNAE

EIVHLAPHDNNLQSGSKGTDFRVTLIDFCVGLTPSRLHSKLCLGISRGNMDFTIGKEQFY

IKSSFREPSINLIDNCKLLKEHKDLTHDESFSPHDMILKLGFLSIGHINTLHLGITVNSD

IEKIKERHKRLGIRGDLSLVDLKLNTDDISIGLAADSCHTLLQTINDLKTPIFFKNDEKF

RTTVSSEFEMPDDILSQIDAVQKNEGLKSFQHADMASSSPIFNMHQQETGSNLVIVDEYY

GETGSIVSEVERGIEGLSLESSHSNGGDSSLPMVEDHYAENKKNDAVKVFPFSLHVNLSE

IKIYLYDGYDWKHTRKALRKAIQDLEQKAQEMAKKSKNLPKKNNSEKVETSSLGCHGPSK

ANNNKVTFDDRLAYDDESAEEGNLSIDENLSDSGDEHQVAGTFFESIHLVMSEGDDPSDF

VNMINSQVQSDMRNDTSDHKTQHGTNDMINVNVKKYYKSLRLNRSGFHKGLIELKNFEIM

VTNFTSRDPRLDPTPYDSEPELVNCVDIRVGNVNVFDNVPTSTWNKLLTYMSAVGEREIG

TDMFRLSLTNVRPDPKLVFAEAIISFKLLPIRLYIDQDTLSFLTRFFEFKDTRFALPVEE

PIYIQKLVVDTIRLKFDYKPKKVDYAGIRSGYHAELANFFILDGADICLEKTTLYGISGF

PKLGEALKNVYGPYIQKYQLANILSGLSPLRSLVNLGGGVKDLVVVPYKEYKKDKRLVRS

LQKGTKSFAKTTTYELLKLGVKLASGTQVILENSEEYFGGEGRAARKSVSKSSTKSKKEP

SSVSRGGPSNNLLETSQLLKRSVKIEKDPFSTPKFYTSASLDEAEENELDSEDIEQSLLV

FNSVAGGFTGEKELETEKYESGNANNDDSGEEYEYEYEFDDHEPSEKTVSLYSNQPSNAK

EGLVSAYKSLGRNFKSTKRTLVSLKKELQAAESFQDSLASIAKSSPVIVIRPVIGTTEAV

IKTLTGISNQVDSTYMKESQDKYPTNDSK

>tr|C4XVW2|C4XVW2_CLAL4 Ubiquitin-conjugating enzyme E2 2 OS=Clavispora lusitaniae (strain ATCC 42720) OX=306902 GN=CLUG_00085 PE=3 SV=1

MSSPSIFFSVCILFLYTTISTYISILGSWWKREEFLRTRITTCRYPSGERWGLKCYLPYK

SSLIASKRFLTPHCRNLTMSTPAKRRLMRDFKRMQSDAPSGVSASPVPDNVMSWNAVIIG

PADTPFEDGTFRLVLQFDEQYPNKPPSVKFISEMFHPNVYASGELCLDILQNRWSPTYDV

SSILTSIQSLLNDPNISSPANVEAANLYKDHRSQYIKRVRETVERSWDEDDDEDGEEEDE

>tr|C4XW14|C4XW14_CLAL4 aspartate-semialdehyde dehydrogenase OS=Clavispora lusitaniae (strain ATCC 42720) OX=306902 GN=CLUG_00137 PE=3 SV=1

MALKKAGVLGATGSVGQRFILLLSNHPEFEIHALGASSRSAGKAYKDAVTWKQTDLLPES

AKSILVEECKPEGNFAECDVVFSGLDADVAGDIEKAFKEAGKVVISNAKNYRREADVPLV

VPIVNPEHLSVVEKKVQEAKAAGKPKPGVIVCISNCSTAGLVAPLKPLVDAYGPIDALTT

TTLQAISGAGFSPGVSGIDVLDNIVPFISGEEEKMEWETRKILGGTNKEGTEFVPLPYEQ

MKVSAQCNRVAVSDGHTECISFRFANRPAPSVEQIKQTMRDYVCDATKLGCPSAPKQTIH

VLEENDRPQPRLDRDRDAGYAVSVGRVREDPVLDFKMVVLSHNTIIGAAGAGILIAEILK

AKNVI

>tr|C4XW30|C4XW30_CLAL4 2-methoxy-6-polyprenyl-1,4-benzoquinol methylase, mitochondrial OS=Clavispora lusitaniae (strain ATCC 42720) OX=306902 GN=COQ5 PE=3 SV=1

MAKTKLITFSCLPRQRQGAPFSHSRIVSIVGAHVQGPPNYASSHFCKSVIYLLFLPTRIV

CRMSSRILFRRATAAKMHSLARCYAVPPQSQPENSGPDTTHFGFQTVKKDEKEKLIRGVF

SSVASNYDVMNDAMSFGVHRLWKSHFINRLDAGMRPGSSRPLDFLDVAGGTGDIAFGLLD

HAEKHYHDTQSTMTIADINPDMLAEGKLRYAKTKWNDGKNRVQFLEQNGETMDAIPDNSK

DVYTIAFGIRNFTNIQAGLNTAYRVLRPGGIFACLEFSHVDNPVVDYIYQAYSFSMLPLM

GQLIADDRDSYQYLVESIQRFPKQEEFSAMIEKAGFYVPEKGYENLTFGVASIHIGVKL

>tr|C4XW78|C4XW78_CLAL4 DNA 3'-5' helicase OS=Clavispora lusitaniae (strain ATCC 42720) OX=306902 GN=CLUG_00201 PE=3 SV=1

MAIQNNLKEAKAWLAAEKPQLPPSWLVDLLKKPVQPSAPFNFPLSASSATPSLRNGLSKI

PQEKSQPQPLQTSTQALVLEDLSDFSDNEDLKLIDTTELGLPRLKRQATLDERLLKKQKA

HIPEQNPRSGSAENGSSSPQHSGSLLTNESHNTTKGSSHIAVSSMSANAQSASHSRNLYS

AQKNTSSNILNHGSNFENNTPQTGSVHLQRGTASRNPSLQMSNAPSNGKPPVFTHQTSFT

SNIEPSRKLTPQSSSLANDSNVASLQNYIRICESKIALLSKWNAINESTSLSLDAKNLWH

TNNFLPQLQKIEASQHALRSSFSFLDPLPATQNLSKISTSSPNLSNLASGSILSPIHTTV

PQSPSVEVTEKRNEQNHVTDIRTSDDDMSGFSQIHHVTSPLNVMSPPAIPPEISVEATKT

STESEYKLNNAEARNFIMERRAAKPLPVSQTVIDDELEDDFGEQFMDGLHSSQVDDAATD

LSGFLARDDEDLSSVDGTYMTQPMSQTKSDIDEVESDEPNDTKDDDLLDEMRDIVLSQDV

AQRYGVKYDNVEPIEISDHEIEEENENENDDENMADFTTQLKEGHDEVVEITSEDEIPDA

DLNVLTELSQSRPLKIEVVSESDFSDDDQELIELSKSVGTKKPGEGKTIPPNSGHFIDEV

YDVLHNVFKLPDFRPNQLEAVVAALNGKDVFVLIPTGGGKSLCYQLPALVKGGKTRGVTI

VISPLISLMQDQVQHLRDRNINAAMISSRGTTEEKHAAIRELTSGQLDLVYLSPEMVNSS

NMIQRVLSKLYESNMLARVVVDEAHCVSSWGHDFRPDYQGMSLFKEKFPEVPIMALTATA

NEKVRLDIVHHLRMKNLVLLKQSFNRTNLFYEVRNKPPNLYEWIRDYVMGKMAGKTGIIY

CHSKQSCETTAQKLNDWGIKCMYYHAGMDPNERFDVQTQWQHNKIQLICATIAFGMGIDK

PDVRFVIHMYIPKSLEGYYQETGRAGRDGKESECIMFYSYKDARALQSLIQRDRNLEESA

RESHLSKLRQVVQYCENKTDCRRKQVLHFFNESFDPANCARKCDNCCSDVVSVTRDVTEH

CSNIVKMVQSLQADKVTVIHCQDVYKGSRGNKILKLGHHENPYHGKGRELDRGDMERIFF

HLQSQDCLLEYQVMKGGFASTYVKLGPNANSVLSGRNRVQLSFAQNSRPSTGNSNRAPRL

TASGNGSGIGHLRYQESFVSAREVRYEQEKENINVERSSSKITLEKHTFDGAVNASNEQA

VEQAYHELRNLRLQKSEELGYGRPHFFMSDTLLKEMAVKLPTNARDFAKLQNISKDQVSH

FQYFKRLLGTLSRERKRQSSQGDSATQHSTFTQANSTISEHASPYFQRSGPGSQVRKKSQ

GRKQRRPAHRKQAETRKGTPSQVRQMPI

>tr|C4XW90|C4XW90_CLAL4 Pre-mRNA-processing factor 19 OS=Clavispora lusitaniae (strain ATCC 42720) OX=306902 GN=CLUG_00213 PE=3 SV=1

MLCSLSGETPKEPVVSPKSGAIFERRLIESYLASTGKDPVSDEPLSVQELVPIKTQVPEV

TPPRPPTFNSIPTMLAAFQNEWDALALETYTLRKQLHTAREELSLALYQYDAAVRVAAKA

IRERDEARSALTELSAAFGAQEKQAGGSEKIPVQALNAARDRLFAEHKNQKVTLGVSSSS

KVQFSASTEDLDIDNVAFLSAEPVTQRTIVASTEQCHVLPDGKKLSLPDISETFFLSKSE

EKLPFLVSGRSLLGIDSGSENALDVSPKQIVPHPTEPLFCVLTDKGEWALCDRDNVLYKS

ENVPSTAIDLHVDGVLLGVGTASEVLIFDLTSTEQVAAISTKHAHVNKIQFALNGYWLVV

GSSSEDKSAVQIFDLRKNSLVHEIDFDAASDFVLDPSCSVLTTYSNLQLVAHLYVKKGKK

WYDAVSALSVQPLHALTLNSTAATVQETNTIEIIGLGEKHLCRYTLSLSQESV

>tr|C4XWM4|C4XWM4_CLAL4 ATP-dependent RNA helicase OS=Clavispora lusitaniae (strain ATCC 42720) OX=306902 GN=CLUG_00347 PE=3 SV=1

MVKHNKKGKKLDRKAKIQQEEEELSKLRKRIEDFDPAQDEKTISQFADLPITEATARGLK

EANFVSMTDIQRKCIPLALKGEDIMGTARTGSGKTLAFLVPVIERLVHSNITGLDGLAAL

IISPTRELAVQIFEVLTKIGKHNSFSAGLVTGGKDVQYEKERVARMNILVGTPGRVSQHL

NETFGMDTSNLQVLVLDEADRCLDMGFKKQIDSIVSHLSPERQTLLFSATQSDSIKDLAR

LSLTNPIKVGVSSDATLSATPETLDQYYVRIPLEEKLDVLWSFIKSHLKSKILVFFSSSK

QVQYTYESFRTLQPGISLLKLYGRHKQTSRLETTTKFSHAQYACLFATDIVARGLDFPAI

DWVVQVDCPEDAATYVHRVGRCARFGRPGKSLLMLTPSEEEGMLKRLKNQNIDIKIMNIK

QKSKKSIRPQLQSLCFKDPQMKNLGQRAFISYYRSVYIQKDKDVFKIEDIPAEKYAESLG

LPGAPKIKIKGGEGNKEKKNMSRQLMQLSKANDNGENEEDEDGKKKVRTKYDRMFERQNQ

TVLSKQYLTMTGTGIRDSKEDEDDEEDDFMKVKRQDHDLKEEELPDLTAPASKRSAKKAL

SKKLSIVNKGMPTKLKFDDEGKAHPIYELEDEKDFKAKGDAKKQKEAFVSKETEVMGEAD

LEDKEMARAKRQEKKRKRKEAERRSREDDDSDEEEETVVTLGTGDLDRDLESSSEEESEP

ATKKPKWFQNDKNVSRNDDAGLVELDAPETLEDLEALTSRLIGN

>tr|C4XWV8|C4XWV8_CLAL4 Ammonium-dependent carbamoyl phosphate synthetase OS=Clavispora lusitaniae (strain ATCC 42720) OX=306902 GN=CLUG_00431 PE=3 SV=1

MLLPSLRAKSFGLARSFCSSSLIRNATPSYQGGKLVQSLQKNGAQGATVDVSKVLVIGSG

GLSIGQAGEFDYSGSQAIKALKEANKTSILVNPNIATNQTSHALADEIYYLPVTPEYVTY

IIERERPDGILLTFGGQTGLNVGVQLDKMGVFDRYGVKVLGTPISTLETSEDRDLFAQAL

KEIDIPIAESIAVETVDAALEAAANVGYPIIVRSAYSLGGLGSGFAASEQELRDLAAQSL

SLAPQILVEKSLKGWKEVEYEVVRDKDGNCITVCNMENFDPLGIHTGDSIVVAPSQTLSD

EEYHMLRSAAIKIIRHLGVVGECNVQYALQPDGLDFRVIEVNARLSRSSALASKATGYPL

AYTAAKIALGHTLPELPNPVTKTTTANFEPSLDYIVTKIPRWDLAKFQHVKRDIGSAMKS

VGEVMAIGRTFEESFQKAIRQVDPSFCGVQGAQFDDLDAALANPTDRRWLAVGQALLHEN

YDVDRVHDLTKIDKWFLYKIMNIVHMYRELEAAKTLESIHHDLMSRAKKLGFSDKQIAQC

VGAKELEVRAARKKFGIVPFVKKIDTLAAEFPANTNYLYTTYNATCSDVDFNEHGTMVLG

SGVYRIGSSVEFDWCAVETARALRKAGHRTVMVNYNPETVSTDFDEVDRLYFEELSLERV

LDIYELETASGVVVSVGGQLPQNIALPLQEAGCNVLGTNPLDIDRAEDRHKFSSILDSIG

VDQPRWKELTSVAEAEKFANEVGYPVLVRPSYVLSGAAMSVIWSQKELDSKLTSAANVSQ

DHPVVISKFIEDAQEIDIDGVAYDGKVLVHAVSEHVENAGVHSGDATLVLPPQKLDSDVM

ARLKTIADKVAEAWKITGPFNMQVIKSDVDGETQLKVIECNIRASRSFPFVSKVLGTSFI

SVAAQALLGENVPAPVDLMSQKYNYVATKVPQFSFTRLAGADPFLGVEMASTGEIACFGD

SLVEAYWTSMQATMNFKVPLPGSGLLFGGDLSNEKLGHVAQTVSGLGYNFYTASPEVAEY

LSKYVSEEVEVIAFPKTDKRALREIFQEKSIGAVFNLARSRAESLLDEDYVMRRNAIDFG

IALFNEKNTAQLFAQCLREKIGTKIDFASVPSKVEVPHEVRRWSEFLGGKPV

>tr|C4XWW8|C4XWW8_CLAL4 Histone acetyltransferase type B catalytic subunit OS=Clavispora lusitaniae (strain ATCC 42720) OX=306902 GN=CLUG_00441 PE=3 SV=1

MPSDEQVNLAVASLQPEVWTSSSNEALSIFITDTKGSAQSFKPTFTYPIFGDAESIFGYK

DLSILLCFDAVTFRPFLNVKWGAKLDQTEVDPKTKMLEFLPESTVFKDELKWREEIDAES

QSYEIPGKIVGEPFSRDGEQYAIYRLDLASERGIELQKRLQILVLLFIEAGSFIDYSDPL

WDVYVMYKVSDPKFPEVVGFTTAYNYWKYPGHASFDEGKVETRKKISQFIVLPIHQGKKL

GGEMYSHLYKSWMQDGNVVEIVVEDPSESFDDLRDRVDFTRLVETKKLDLSSLTVAQASD

PKWFNELKTKEKLEKRQLQRLLEMAFFYQLSHNIASDTKKAVRLFVKRRLYEKNKEALAT

LDGPTRLDKLQTAYEALEADYHRILEPITINVKRAAPADEGVASKRSKIQ

>tr|C4XWX4|C4XWX4_CLAL4 alpha-1,2-Mannosidase OS=Clavispora lusitaniae (strain ATCC 42720) OX=306902 GN=CLUG_00447 PE=3 SV=1

MRLVALVIGMKWFLLLSACVLPVPAAEGPDIEWYKWGLPKERDMEDMKGDMEGMVVGDME

GDMEGDMEARDMEAIGVEAIDMEDASAISIASREASISTEATETTTASTGPKERGAEPEQ

ESEQPEPEAESEAEPEPESNRPESFLAEHVSAEPSFAAESGSFSSAAFTPAHLRSLRARA

GALFAHGWRAYRTVFPADEVRPLSCRALGPDRTDAFNLRNDAMGNVASTALDNLDTLILM

RQWHELAFVLRHLEKHQHHYFAQNATVQVFEAAIRWLGGLLSAHLLLSEIPQVQAVFPAY

DGFLLHMAHDLGRRLLPAYNTPSGIPLARINLAGHAVPPALNAETCTAGAATPVLEMTLL

SRLTGDGRFERHAARAFWALWRARSRAGLVPSAVDPHRGAWLNAATGVGASVDSFYEHAL

KGAIVFGDARLMDVFVRSYEALSAHSAQSVSGAAAYFALADTGSGAVAAPWIDSLGAFWP

GLQVLAGRLSDAVASHVVYMKIWNTFDVLPERWIFAAPPTGRSGQPGQQGRSVEPVRSVQ

SAVPLEWYPLRPELIESTYYLYRATRDAMYLEMGVRFLEAFETRFKDRCGFAGVQDVRTG

ERQDRMETFVLGESLKYLALLFDEATFLHTGMPGNWVFSTEAHPLWYTPGLGRRHAARFK

RKRARREIKKTKRQEGKSGKMNDIEKMKMDDVDTEYTDDIEYIDDIEYTDDIEYIGSDDN

SVFSSLWARLRSKDADGIEPAAPRLPFGPGIPPVASLLDSCEVRPQQFRPHRGPLASSAY

TRWPHLFLADARFRPTLVRPDYLGPQRSFELGASFLATHALGSPLQCAREPTSSELDVIL

DTLRRPEDYAMYVVRRPRPDHAFAKGDYVMPQLSGRLLLECVSAGSVDWANRRVSRAFVR

ARRPEMWASASAEVCRVAKLNGVAVARNATVWMERRHAETQSGVFTVACNGRIFIEGKYL

DNVRVY

>tr|C4XX21|C4XX21_CLAL4 Succinate dehydrogenase [ubiquinone] flavoprotein subunit, mitochondrial OS=Clavispora lusitaniae (strain ATCC 42720) OX=306902 GN=CLUG_00494 PE=3 SV=1

MLRSFRAFSTSRAARQVIGAAKGETVNSTKYLGQKYHVIDHEYDCVVVGAGGAGLRAAFG

LAEAGFKTACVTKLFPTRSHTVAAQGGINAALGNMHKDDWHWHMYDTVKGSDWLGDQDAI

HYMTKEAPQSIYELENYGVPFSRNEEGRIYQRAFGGQSKEYGKGGQAYRTCAVADRTGHA

LLHSLYGQSLRHDTHFFIEFFAMDLMMQDGECVGVVAYNQEDGTLHRFRAHKTVLATGGY

GRAYFSCTSAHTCTGDGYAMASRAGLPLQDLEFVQFHPSGIYGSGCLITEGARGEGGYLI

NSEGERFMERYAPHAKDLASRDVVSRAITMEINEGRGVGPEKDHMFLQLSHIPASVLKER

LPGISETAHIFAGVDVTKEPIPILPTVHYNMGGIPTRWTGEVLKKNEKGEDEVVPGLLAC

GEVACASVHGANRLGANSLLDLVVFGRAVSHTIRDSLTPGTPLKPVPKDLGLDSIANLDK

LRNANGTKSTAEIRLAMQKTMQKGCAVFRTQETLDECVKNINEVDETFKHVKTTDRSMIW

NSDLVETLELQNLLTCATQTAASAAARPESRGAHARDDYPDRNDAEWMKHTLSYQEKFGA

PVRLDYRNVISHTLDENDCKSVPPAKRVY

>tr|C4XX45|C4XX45_CLAL4 Imidazole glycerol phosphate synthase hisHF OS=Clavispora lusitaniae (strain ATCC 42720) OX=306902 GN=CLUG_00518 PE=3 SV=1

MDQTVHIIDVKCGNLQSLSNAIKRIGDYKIVFIEDASDFTKYDDSISKLIFPGVGNYGHF

VKELSERNLFEPIRTYIESGRDIMGICVGLQAFFKSSEESKGIDGMNLLDLDLLKFSSND

PCFAAHNQLKSIPHIGWNTISEIHHDSKIVESNTTLFNLNVRDKYYFVHSYAAILKDEKA

VEKLKAAEAKGWDFALSKYGSETFLAAVAYKGLFATQFHPEKSGVVGLKIIKAFLEGQKF

SESNDISTSSVEGLETTVGGLSRRIIACLDVRTNDSGDLVVTKGDQYNVRETSESGESNV

RNLGKPVELATKYYLQGADEITFLNITSFRNSPIKDLPMLEVLKRSAAQIFVPLTVGGGI

KDMVDPVTGELYPAVKIADLYFRSGADKVSIGSEAVTIAESYYANGKKKTGKSAIETISS

CFGNQAVVISVDPKRKYVSNPSDTSMSVIKIEDPNSFGPNGEEYCYYQVTSQGGRKTHDL

GALELCLACEDLGAGEILLNSIDYDGTNMGFNLQLLRQIKENVSIPVIASSGAGKPEHFE

EVFKMDCGVDAALGAGMFHRGDYDVLTVKKYLQEHGNMDVRIDTQIEL

>tr|C4XX47|C4XX47_CLAL4 HRDC domain-containing protein OS=Clavispora lusitaniae (strain ATCC 42720) OX=306902 GN=CLUG_00520 PE=3 SV=1

MSDKHALFDTVFPKTVKAVRAASALAAQDINFYTSLDKGISSDVKDASSSLLALANKLLS

KTTKDFEFIEYGEENIVSKLNWKRVSDVLDTCFEKADLALDEVKNPQRASTSHMIYLKDS

SDEKKATEVKVKPQENFRSTVDNSDSSPFKPRLENKPYALKSLEESLKLRFPDPTDDGKV

SAPHYAHPYEFEIMNQPYPESCLQVNKPVPSTDWATTAAKWVDTVDALQEMIEALRSSTE

IAIDLEHHDYRSYYGITCLMQISNREQDWIVDTLALHDDLRDLNEIFANPAILKVLHGAN

MDIIWLQRDLGLYIVSLFDTYHASKKLGFPKFSLAYLLENFAHFKTSKKYQLADWRIRPL

TDAMMQYARADTHFLLNIYDQLRNKLLNAGQGKVQEVLYESRKVASRRFEFNSFKQDQTD

NWMHSYGGMGQERWVMNQYNIEPERIEIVQALINWRDKVAREKDESTRYIMSNQVLANLS

SLVAPVDASKVHNAAGSQYSIVRQNSKELAELIEKYLATVGHSANNENVTSFDLTEVNYD

KASEISKHFTDLTKSVPTATNLSLSDKDSRLLSKFAGKSFSLSAESFEPSTNERIRISQE

ELMKRATLLSESLAENLTSFEIENEKSKIDNMEEAEEKIDKSATESPQRQSSEEDKDAIV

VLSGKKSKKSFNKKQSTADEELFDYSTVEEKVLANVKNGNTKKRSFDPFSRQGTGPKAAK

RSRNVPTGKSTSFVKKRN

>tr|C4XXK0|C4XXK0_CLAL4 Ceramide very long chain fatty acid hydroxylase OS=Clavispora lusitaniae (strain ATCC 42720) OX=306902 GN=CLUG_00673 PE=3 SV=1

MSKTLPLLSQADLAKHNTAQDCWVTLYNRKVYNVTKFLDEHPGGDDLILEYAGKDITEIM

GDAETHEHSESAYEMLEDGMLVGYLATPEEERDLLNNRNKTPVEVKLTKEAEEQVDLYEF

HDELPALEKLSIQTDFSDDNKKHKFLDLNKPLLPQMLTSTFDKDFYLDQVHRPRHYGKGS

APLFGNFLEPVSLTPWWVVPVVWLPVNLYIFSIGFSGQSKITALSFWALGLFVWTLIEYC

MHRFLFHLDGYLPNHRIFFTIHFLLHGVHHYLPMDKYRLVMPPTLFVVLAYPFYRLVFAV

LPYYIACSAFAGGTLGYIMYDVTHYVLHHTRLPKYFHDLKTYHLEHHYKNYELGFGVTSR

FWDVIFNTEITSTFEKRK

>tr|C4XXL7|C4XXL7_CLAL4 ATP-dependent RNA helicase DBP5 OS=Clavispora lusitaniae (strain ATCC 42720) OX=306902 GN=CLUG_00689 PE=3 SV=1

MAEKPTTDASELLASLSLQKEGHADVESAVSAAPKAETEKVDKEEKAPEPETNLIKSTYE

VQVKLADLQADPNSPLFSVKSFEELGLSPELLKGLYAMKYNKPSKIQEKALPLLLSNPPT

NMIGQSQSGTGKTAAFSLTLLSRVDESDNSVQAVCLAPARELARQTLEVIQTMGRFTKVT

SKLVVPGSYSADSTFNEHILVGTPGTLLDLIKRRRVNLSKVRVFVLDEADNMLDAQGLGD

QCVRVKKALPPTAQLVLFSATFPTEVRAYAERFVPNANSLELKQEELNVDGIKQLYMDCN

SEQHKFEVLCELYGLLTIGSSIIFVGTKRTADMLYQKMKQEGHTVSVLHGSLDNAERDRL

IDDFREGRSKVLITTNVLARGIDIASVSMVVNYDLPVDKDGKPDPSTYLHRIGRTGRFGR

VGVSISFVHDKRSYEVLKYIQQYFGDIEMTRVPTDDWDEVEKIVKKVIKN

>tr|C4XXQ0|C4XXQ0_CLAL4 Uridine kinase OS=Clavispora lusitaniae (strain ATCC 42720) OX=306902 GN=CLUG_00722 PE=3 SV=1

MSSLGKKKGRRYSRIAPHDDESSSFFTPSPMVDSKDGKSVSELDLRSTVTGRNEPTYIPP

WTEPYVIGIAGFSGSGKTSVSQRIIQELNQPWTVLLSFDNFYKPLTPEDYKLVAENNYDF

DTPDSIDIDLAVETLRSLKEGKKTEIPVYSFAQHARTDKKITIYGATVIIIEGIYALYDK

RLLDLMDIKVFVDTELDLCLARRLTRDILYRGRNVEGVIRQWERFVKPNSVASVAPTMQN

ADLVIPRGLDNSTAIDVMIKHIQNKLALKSAEHLERLKALGLSNKIELSSLDNLKLLPRN

NHTAGIHSILFDVHTERTDFIFYFDRIANLLIEQALEQLTCFEETSVSCPSGYEFKGLKP

THDFVAVSIIRSGDCFMNSLRKTFPDIPVGKLLIQSDSLTGEPQLHMEALPQINEESQYF

LFDAQIISGAGAIMAIQVLLDHEVKERNIVLISYLSTEVGVRRIFNVFPNITLIVGRLSN

MEEVNQEFNSEGFKDTDWMFRNRFIDSLYFGTD

>tr|C4XXS2|C4XXS2_CLAL4 DNA polymerase epsilon catalytic subunit OS=Clavispora lusitaniae (strain ATCC 42720) OX=306902 GN=CLUG_00744 PE=3 SV=1

MSVFCNTRRLSGGCNRKIEIEERNYSEKTLILLIDPPLGVCFRRQAENVAGSDWEILEYK

KDPMNPGAVKVYVFSGGKIHSFLFHIPKTIYATYKADVFFKPIPNCEIEKSTAILPNGHD

ATNLFKMTMTESTYNEQISSVDSILHDSKILGLYETEVSSIDRAIMDLGNIVRFDDTKVG

ALGKCLKNGFTTKDLIKVEKEAYLKKFDMDVVYLLHISTNSYEFFAIFTTWDSNVTMLIL

KPSSGAQELPDNISKIYKEVYEAKKSKLSKLYNFIDYMPEMTFDTSYFNNPSTLYKKLNS

VFRKFHESRSNKALLSIQSPYCNKVLDLLEAASDFPTIKLSVGEISLPAVGWQSLIAKRI

VNHYFSLASWLKNMTSLSSYGKVPLCNLQIETPGYLIDIEYSRRLTQNNVVLWWSNKPYP

DHGGFQNDHVQDFDNFDFITINSPEIYETACLEVEIGNLTINTILTSSLINEAEGTDLSD

DPLLGDANNGASTLALDTFSPVALGILRSMVKDWWDDAVSNNANADSMMNSLVSWVQRQD

SLLYDFTLHHHIHNLTSKALLQLVGEFRRMNANVVFANRSKLIIQTTKISVENSYAFGKY

VVNATRSKPLFNFLDLKIVRYWDILVWMDEFNFAGRCCTEITNDDVQQLLPVSKWHIKKF

LPIIFQNDFDDWLVIFLDALAKYKNETLLGSTQKGTQRVTQLAHILKGQQKMDSNDAEEE

DQFTGEVMNNFRKPLQRRIEKLYRRQNESILNPEMAKEYEFPTLPGSVLKMKNPVLELVK

FLCAVFALSKKRSIEVRMLRKDLLSVMDVKEFSPESIFSSPSASLKVSSVICDYCSFIRD

IDFCREEEKNIWTCTHCHHVYNKVFLEEELIAQLHRNISKYLTQDYTCQRCHAVKADNMS

EFCKCSGAWVETLKFDDFSKRVNIFRNVAKAYGMKMLLGLIDDYFI

>tr|C4XXT1|C4XXT1_CLAL4 S-methyl-5'-thioadenosine phosphorylase OS=Clavispora lusitaniae (strain ATCC 42720) OX=306902 GN=MEU1 PE=3 SV=1

MSLHREKVSLAELPHTYDKPIPLAVIGGTGLYDLPNLKPVARLTVSTPWGFPSAPITVSV

TESGHAIAFLARHGPHHDLLPSDVPSRANIAALKKIGVKAIIAFSAVGSLQPEIRPRDFV

VPTQIIDRTKGIRPSTFFEKGFVAHAMFGEPFDVKLNRIISDAIPATGFLDAFDSAAEPK

LHTKHHTNNGEDLTVVCMEGPQFSTRAESRLYRTWGGSVINMSVLPEAKLAREAEIAYQM

ICMSTDYDSWNESEEPVTVETVVGNLKANSANACKVAAKLIDVVSDDLYSGKSGLAADLE

GSMKYAVSTSTHGVKKELLEKMHYLFPGYWPVQ

>tr|C4XXT8|C4XXT8_CLAL4 DNA replication ATP-dependent helicase/nuclease OS=Clavispora lusitaniae (strain ATCC 42720) OX=306902 GN=CLUG_00760 PE=3 SV=1

MEKRERNSGKSPVSASKRPKSSLKHKKTSYFLPQNTLAYNPQSSISDKNNKIHAPKDRLL

EESRKKGPESTNPSIKDSSTENVSESFTPEPTETGQISKTHVQSTKLYDRSLEQHSSDDS

FEGVRWQPTTSPNKYARPILSSPLKNADLGDRSLRNVLTAETVVNELTDSVLCKYGLGMQ

NTLSQTPKNHRTSSDVAKSTLDMSPTLTRSKSFDPRSLQTTSTSLSSESAKVSNLNIWLD

KFQGPGHVTKAPSEPPMKAIQNSHVPSTNSNDDASSEEDPFLDDDTFLADLKIESFTTQG

IPFQQAPASKLDTPTELAHDAESASILHRDSPKSLSQRQSALKASTSSASSPTVSTTASP

NASPNTSLNASPKNSSPQIIPEESDPFSDDLDVVAIEKSAVATQATSEPTLPTKNGKRNT

GRLSPSPDSFEEENVGAKLSYTRADFVRYQIMSIMQTTYPHQNFKRKQLILTVADADEQT

TKIVIRGDSAELNFQEGDIINIIITSPESPKLVDNNNNLIIWNPDTLISSTVVADQLFCP

RKTVMNKRYVFPGEATIPLIVGTTVHEIFQACFITEQSSPEYMEQLLEVETKRRLLEIYS

MGDVLEETKDRIRKHFSFIEKWFNTFFKKPPSIIPTNKHQQNIKFSVADVLDVEESIWSP

MFGIKGMADVTLKANLEGESATGQFLLPMEIKTSKEYLSHHAQAALYSLLFKDRYNANIS

SFLMVYTSEEGSTKKHDISIPDLRSLVNLRNRISPYIKSGTRELPDIMRQQKCDNCVIQS

SCMTVNYLLEHGTPENSGLNDGVYEDLTEHLVQRQEYSDYLNYWDDLLTSEEEFVSRFNK

DLWILTSKEREEEQGKALSGMIITEKSEFVDNTSEFIYKFQRRDFKNIRPIDATIISKYD

RVIISDESGHFALAQGFVKHIDCGCIIISTRRKLVSTEVKNDRFHRAAVLRPTQSQSPQG

TQTETVIFRIDKDEMFYGMGVARFNILNLFLRDGDTKRRKLIVDLDTPRFSPVPVANVKK

DHFNSDQIRAFNKVFQTKDYCLILGMPGTGKTTVIAHLIRMLVEEKKSVLLSSYTNSAVD

NILLKVKEFGIDFIRIGNTSRVHPDIRPYIPGSDEKCVENYEDFIRIYQKPLVVAATCLS

MRDLAFNVRERFDYCIVDEASQVSMPLSLGPLSRCDKFVLVGDHYQLPPLVVHPNVNVKK

GLSRSLFQILADEHPQSIVELSFQYRMCKEIMLISNALVYNNRLQCGSETVAKQSLTIPN

PEALAAYIDPRSPLSHRWLSDVFKPENKVLFLNHDNMNAYERKVGENVSNLVEVELIRQI

VESLCLCGVDESKIGVMTLYRSQLKLLVQCFKHRPRLEILTADRFQGRDKECIIISFVRS

NKEKRVGDLLKDWRRVNVAVTRARSKLITVASKSTLSHADSIKDFVYLAEQKNWIYNLPS

SAYHVYQLPKPKDNFASQEIKKQPIKFGEKIISKHPVVKDILSDMNAIN

>sp|C4XXU2|MTNB_CLAL4 Methylthioribulose-1-phosphate dehydratase OS=Clavispora lusitaniae (strain ATCC 42720) OX=306902 GN=MDE1 PE=3 SV=1

MSSFFSDSPTHPANLICELCRLFYNNGWVTGTGGGISIRDVDGPNPNIVYIAPSGIQKER

LQPREMFVAELPGKILRSPNDDSDGQPLSPDLAKSFRYKPSACTPLFLSCYNMRDAGACI

HTHSQNAVMATLLFEDKVEFSMSHIEQIKALPHLQVDSDTGKVQKVGSMQFYDTMVLPII

DNTPHEEDLTDSLQEAIKNYPGATAVLVRRHGIYVWGETVWKAKVYNEAIDYLLELAIKM

HQAGIPLVKK

>sp|C4XXV8|GET1_CLAL4 Golgi to ER traffic protein 1 OS=Clavispora lusitaniae (strain ATCC 42720) OX=306902 GN=GET1 PE=3 SV=1

MFELQPSSIVVLVFCVLAIKVCISLIGKTTIQDRIWYLYTIGASKAGHSKFVALAQKREE

LVRVNKERRAISAQDEYAKWTKLNRQFDKLNSEVNDLAEATSSEKAQISKLVNLAIAATT

TAPIWFSRIWYRKVVLFYLPPKVFPYYIEWVLALPFIVTGGVGLTVWMFALNSVLSSLEF

LIKFYLEEPVKKPEAPAASEAQTKQ

>tr|C4XXY2|C4XXY2_CLAL4 NADPH--cytochrome P450 reductase OS=Clavispora lusitaniae (strain ATCC 42720) OX=306902 GN=NCP1 PE=3 SV=1

MLDTLDIAVITALALAVAYYFGKDYIVSSDPHSSGFLADDGSENDRDLSSTLKKNNKNAV

VFYASQTGTAEDYAHKLSKELTSKFGLRVLIADTADYDYDNVQNLDPDYLFFFVVATYGE

GEPTDNAVEFFNWLENEADQLANVKYTVFGLGNSTYEFYNAMGQKLNEKLESLGAERIAP

YGQGDDGIGTMDEDFLAWKESCFDALKTNLSLEERESVYEPSFKLTEDDSLSSSDAGVSN

GEPNKAYVDFTKDLTKGPFDHTHPYLAPITKTKELFNSKDRHCVHAEFDISGSNLRYTTG

DHLAIWPSNSNENVAQFLKCFGLAEKADTVFSLKALDSTVALPFPTPITYEAVVRHHLEI

SGPISRQAFASIAPFAPNEDAKKECLRLGGDKLVFAEEIHGKYLNLADALLSISKGLPWT

SVPFVFLIELIPSLQPRYYSISSSSMSEKTSIHVTAVVEAEEHDGHLVTGVATNLLKDIE

VHQNHSDDKTYASYDLNGPKNKFSNYKLPVHVRRSTFKLPSNPSTPIILVGPGTGLAPMR

GFIREKVKLLETSSNAPLGKILLFYGCRNKNEDFLYKDEWPEYSKVLGENFELDVAFSRE

DPNKKVYVQHKILARAKEINALLEKGAFIYVCGDASRMARDVQSTFVEILASERGISTER

AAELVRSFKVQNRYQEDVW

>tr|C4XXZ1|C4XXZ1_CLAL4 tRNA (guanine(37)-N1)-methyltransferase OS=Clavispora lusitaniae (strain ATCC 42720) OX=306902 GN=TRM5 PE=3 SV=1

MLKRFRSFPIFNRELFFSPARYLHLWNKTERRLIMQDKFGPPVNRDMRVLDRSFFKKDVN

LLVATFPDPKYLGNFVKACKGEILLLPGVKHIVPVENTRGVLLRQDIDDISTYEDKLSPT

ALEKIKEYGVSIKPYVLTLDYSFWKADDILNAVLPENLLDEIPTGFAQAGHIAHLNLRSE

FKPYGPLIGQVILDKNSKIETVVDKVDSIGTKFRTFKMKILAGKDDFIVEQSESGCKFRF

DFSSVYWNSRLSTEHERLITQFQPNEVVGDVFAGVGPFAVPAGKKNVLVLANDLNPESYK

YLKENISLNNVQQFVQPYNYDGREFIRESPRILLEWAKSEGKVQKTKTIKRRKVDPQTKE

KITTKDVEVTSVPIPKFFTNYVMNLPDSALTFLDEFVGLYSRFPEVEEAVKNDPDFKLPI

INVHCFEKYSPTETEPSMEELHRRVHAKIVKLIGFEAPFEKFSFHLVRKVSPTKPMFCVT

FELPHEVAFKK

>tr|C4XY27|C4XY27_CLAL4 adenosylmethionine decarboxylase OS=Clavispora lusitaniae (strain ATCC 42720) OX=306902 GN=CLUG_00850 PE=3 SV=1

MVAPAYIENSYVDHALSANLDSTFAFEGPEKLLEVWFWPSPKDIPSSVTSEGLKAIPLEK

WVSILDLVNCKILSMKSTPAVDAYLLSESSLFVFPHKMILKTCGTTTTLAALDEMFDIAR

EYCTVNGMPTKIDSKSVEKVFYSRRSFMFPEKQKHVHKDWKSEVELLNKHFVAGKSYVVG

DFTSDDHWYLYTGGSGSGRSAFGTSRDQTFEILMTRLDPNKAEMFSLERVPGPESVVDAD

HDLGHEFGETIMRSTGLNSIFDEKKTNVVPVGRSIMPSPELSDNMELSDDENEEKPPLEF

LHDAFAFSPCGFSSNSVSSDQDGYYYTLHITPECGWSYASFETNYPFSSKSNVSVTDVLL

RVLAIFSPGRFSMTMITDVASHGLSSSEIANHESLLELSKCDGALAKLGYKKHEKVIYDL

KGEHNLLYLNFEKQ

>tr|C4XY70|C4XY70_CLAL4 Kynurenine 3-monooxygenase OS=Clavispora lusitaniae (strain ATCC 42720) OX=306902 GN=BNA4 PE=3 SV=1

MSEQKSVGVVGAGLVGSLAALAFSSKGYNVTLFELRPDPRSDSESRKSLRSINLAVSDRG

IRAMKYVDEEMTSRVLEHVIPMKGRMIHDATGTKQESQIYGLFGESINSIDRQFLNVCLL

NEIDAAKVQTKFGHRLVGLGSVNEQKSYLEFQTKDSNIERFEFDFIVGADGAHSQFRYQL

QKTMRMDFSQKYIDMQYMELSIPPVKGKDPKSEERFAIDPNHLHIWPRKDYMLIALANGD

GSFTSTFFSPWGMIESFSNDTEYLEFFKTSFPDAYKLIGEEGLRTAFNSKVRGSLMQVEM

SPYTNPSHNAIVIGDAAHSMVPFYGQGMNCGFEDVHVLMKLIDKNNGDAKSAFAEYTGAR

RKDIQTICKLAMDNYYEMSTKVIDPFFLFRKKVDYFLGKYANGVLFPWIPMYTMISFRGD

ISYSKAVEIEKRQRRVLRCIEYVTVGSIAAIGLAKVAQYWDRFRR

>tr|C4XYC1|C4XYC1_CLAL4 Kinesin-like protein OS=Clavispora lusitaniae (strain ATCC 42720) OX=306902 GN=CLUG_00944 PE=3 SV=1

MIPVSSRQSSISVAVRVRPFTPAEEDKLVRESHVPLFVGDGSLQGTATDEQKASQGPKGL

RKIVKVVDDKMLIFDPPDTNPLSKMQKNAFPNGKGRIKDYRFVFDRLFDEHATQNEVYES

TTKPLLDSILDGFNATVFAYGATGCGKTHTISGTPENPGVIFLTMKELYERLHALSDTKI

VDVSISFLEIYNETIRDLLNPETSHKKLVLREDSSKRIVVSNLSSRSPSSVEEVMEIIML

GNSNRTCSPTEANAASSRSHAVLQINVVSRDRTASLSEEHTFATLSIIDLAGSERAAATK

NRGATLNEGANINKSLLALGNCINALCDPRRKNHVPYRNSKLTRLLKFSLGGNCKTVMIV

CISPSSQHYDETLNTLKYADRAKDIKTKLVRNRQNLDRHVGSYLKMITEQKQEIEELRQR

EATVVQNAIKQHDSSLKKCMTAITKNIQSLKSNLDKQVHEKWRKYFQLAKRKLLLLQSND

LSIMIDHLREITNNKNIYEEFHELIPTIKRILVQSEQLNNKCSEQIINLERQYDTPSEID

EILKSSTQHTLERLKEMDGWSDEFTDIFHQLIEALKDRLQKDFLVNSSILFDYLIGATND

YNYIPRGLSRLVQSLIAKKEDEETTGAAAILHDIRNILERMNDTDYDAAVEEATTHFMHV

KAELEEQRSRVDMAAGFSESTPQLSNRQRDKRFSTSPLRSSRAIKKLSKFPSGWNAGSNA

ESDISMDDSTIGRSDLDEDSPMSNRIIEKSLLDTLDLNHNVQSPPSKAITKADRKSRMPA

LTETKLSTHGSHDMLAKLPLLNKQASTKIVHNEIINTRDTFNFPAKAVDTPISPTIAHHL

PRHEFNNNE

>tr|C4XYC4|C4XYC4_CLAL4 18S rRNA aminocarboxypropyltransferase OS=Clavispora lusitaniae (strain ATCC 42720) OX=306902 GN=TSR3 PE=3 SV=1

MSPTHSTLWRTFPWQERPTFSRREFRTTRRSVSWHRPTMPAPPRTPSPSTKTFRSRVTLG

YKTHYVVRLFGCFFLSFFNHVFIGISSGPCASGMGSGHSFLICLIQKSQKTLSRVLWCKS

SVHPVTPSLFTCSTDEPLIILHQMGKGKNKPTEERASKSRTSNGHKSKQHHIRRGRQEAG

SQTKHASADFPVKLAMWDFDHCDPKRCSGKKLERLGYIKNLRVGQKFQGIVVSPNGTGVV

CPNDREIVETMGAAVVECSWARLDEIPFNKIGGKNERLLPYLVAANTVNYGRPWKLNCVE

ALAACFAIVGHWEWAETLLENFSWGLTFLEINKELIEVYQQCTDSESVTAAQDEWMAKLE

AEVQERKKQSAAGDVWMMGNVNRKGDSADEDDSETERSDEESEEVEYDNLGNIVRRGPAD

LAESEDESDSEEEPESEEYEYDKLGNIISKSTGLEQDMKTVQL

>tr|C4XYG1|C4XYG1_CLAL4 ATP-dependent 6-phosphofructokinase OS=Clavispora lusitaniae (strain ATCC 42720) OX=306902 GN=CLUG_00984 PE=3 SV=1

MAIPLVSGPSGAILLAPEKPLMAQTISFYSQLTGFSVVAQTDSSATLAGKDGASLRIELG

DTKSADQIAESISRWSTELQTRDWRSVAPHLTLCVSEFSELVSRLQSFKYPVQLYPNDLY

PTEAYTVDPLGHVVGFTTAKNPMSINPPVEKFTHGHEIAPSMSTTVSEKRATAPGATQRR

NIAVMTSGGDAPGMNACVRAVVRAAIFRGCRAFAVMEGYEGLVRGGPENIREMSWQDVRG

FLSEGGTNIGTARCMAFKERAGRLAGCKHMVEAGIDALIVCGGDGSLTGADLFRSEWPSL

IEELREKKEITAEQYEKHKHLNICGTVGSIDNDMATTDATIGAYSSLDRICKAVDYIDAT

ANSHSRAFVIEVMGRHCGWLALMAGIACSADYILIPEKPSSSKDWQDQMCDIVSKHRAKG

KRKTIVIVAEGAITNDLAPISAKEVKDVLVDRLHLDTRVTTLGHVQRGGTAVAYDRVLAT

LQGVEAVKAVLESTPETPSPLIGITENKIVRLSLVDAVRITKSVASAIESKDFAKAMSMR

DSEFVEHLHNFMAINSANHEEPTLPQEKRKRIAIINVGAPAGGMNSAVYAMATYCMSRGH

TPYAIHNGFSGLARHESVRSIDWLTIEGWTSVGGSEIGTNRATPQDTDIGMIAYYFEKYQ

FDGLILVGGFEAFASLDQLEKARSMYPSFRIPMVLIPATISNNVPGTEYSLGSDTCLNSL

MEYCDVVKQSASATRDRAFVIEVQGGNSGYIATYASLACGAQASYVPEEGIDLAQLELDV

RHLKEAFATEKGMSKSGKLILKSTNASKVLTAEVLANIMKSEANGEFDAKTAIPGHVQQG

GLPSPIDRTRGTRFAVKAVQFIENVYDDIAPYHDALDFPIDDKKVINTAAVLGIKSSHLT

FSSIRQLYDFETETESRMPKKISWSTVRDICDQLVGRTKLAAK

>tr|C4XYN1|C4XYN1_CLAL4 ATP-dependent DNA helicase OS=Clavispora lusitaniae (strain ATCC 42720) OX=306902 GN=CLUG_01054 PE=3 SV=1

MTNDSTSDNDLLNDDLDLHLLEKAEYELMEKKNSQRRITHHEVDESNLATYIYPTNLQVR

DYQYNIVYRAIFDNVLVALPTGLGKTFIASTVMLNFLRWFPRSKIIFMAPTKPLVAQQIK

ACCGITGISSSQVAILLDKTRKNRSTIWNEKQVFFTTPQVVENDLTRGIVNPKDVVLLVI

DEAHRARGNYAYNNVVKFLNRFNNSFRILALTATPASDVDGVQEIIDNLCISKVEVRTER

SIDIFKYLKRKVIERITVSPSPEIVDAIDMICQAIEPILETANQRKIYEITDPAKINAFA

ALDAQQKIIKNPNIPEGLKWSNYFILQLLVVVGQCFRRLNIYGIRSFYSYFYDKYTEFST

KYNNKKSTNHLAAKFYFHSSIKGLLHKCEGLVKDPTFLGHPKLEVLISELTSFFENTNNS

NSRVIVFTEFRESALDIVRALEAKGDHLKPHIFIGQAKEKEKFDEEKFLKKGKKSKGKDK

KQNSEQKQMLERPGSSSERAQISGMNQKMQKQLIKDFKKGSYNILVATSIGEEGLDIGEV

DLIVCYDSTSSPIKNVQRMGRTGRNRDGKVLLLFSSNEESKFDKAMGGYEYIQQHIINGN

MVTLHDQNRILPPGITPVAEEKLIEIPKENEDIKAEDDEDEIIKIATKYMTKTKTKSNKL

DAKKGPKTPAPKAQKQFFMPENVNQGFTPVSTMLKRRGEDESIRDKRQRLNSEKEDKRDP

DILDSFLDSDNDHDLLQTGSNNESAGLSNDYENGKHLEQNPTYGDSERISNKSSLPIVSN

ENLNSSDSSQVGKEIEYEHNADPSKRQLTIVDQLQAVQKRSFSDDANNDNGDDAFDDDDD

EILALARSSSFSFPQSSYGVTAEESNLVPIFTQNEGFLTDEQNSELYLSYYVPTDASALA

ECYVPSRTPQRGGRIGHGKVSLSILKSQEYMEKTNRQDKRDLLEVYKEVDTSAPTLGEFI

EF

>tr|C4XYP1|C4XYP1_CLAL4 Ribulose-phosphate 3-epimerase OS=Clavispora lusitaniae (strain ATCC 42720) OX=306902 GN=CLUG_01064 PE=3 SV=1

MVKAIIAPSILASDFANLGCNCHKMYDSGADWLHIDVMDGHFVPNISLGPPIITSLRKEI

PRKAEKTFFDCHMMVSEPEKWVPEIAKAGGDQYTFHYESTKDPLALIKLIKENGMKAACA

IKPGTSVDVLYEIGSELDMALVMTVEPGFGGQKFMADMMPKVEKLRTKFPHLNIQVDGGL

GKETVPAAAEAGANVIVAGTSCFTAPDPVVLIDFMRETVNNSLRAKGYYTE

>tr|C4XYR1|C4XYR1_CLAL4 Cysteine proteinase 1, mitochondrial OS=Clavispora lusitaniae (strain ATCC 42720) OX=306902 GN=CLUG_01084 PE=3 SV=1

MFPFCNWNSMGSNTSKETSSNGAPGSSGQEILNEKCTYFDSTSISELVNGLSLGAKDTSN

AITVSHLSSWEAKLLADPKNRLAQNALSKNDIAAITATSGTNLSLKDRYFFNVEVDTIGS

PSYLNNQKSSGRCWIFATCNVLRAHVIKNYNLDPQKFQLSQSYLFFYDKLEKANFFLENI

IDTAEEDLDSRLLQFLFSGSVSDGGQWDMIVNVINKYGIVPNEVFPDNAQAVSTSKLNYV

LTNKLREYALILRKLIAKGASKEEILGAKNAMNKEVYNIIALSLGTPPKPSDSFSWEFID

KDGKYSHFETSPVDFYQSHVKYDVSKRFSLINDPRNEYNALYTVDRLNNVYEGKPIEYVN

VDLPNIKKVAIKMLKDNEPIFFGSDVGKFGDTASGVLDTGAYDYSLVFNTNMNITKLERL

KTGSSAMTHAMVITGVHLDPKSGKPIRWKIENSWGDAVGDKGYFVMTDAWFDEYVFQIVT

SKKYVEKATYDIWKAKDFKVLPFYDPMGALA

>sp|C4XZ24|FLO11_CLAL4 Flocculation protein FLO11 OS=Clavispora lusitaniae (strain ATCC 42720) OX=306902 GN=FLO11 PE=3 SV=1

MLRGFFTLLIFVAFTAATAVINACPNQKFTFHAQVVNFPQATITVTDPSDNGDGTWDVTI

NFNADATMSLKSLSELKILSLSKTYFLYSYNLKVDNINNPGSWSQRVTVTPRSVGDYKTC

MPQFTIQYDWCSAGVTDWSECQNWKYQGSYDYITGCDNFDQSTGFSQKDAPDYCWDATLP

QSSSAVISSAAPSSSAPVVPSAPATPNTPSSPSDPNTINACPNQKFTFHAQVVNFPQATI

TVTDPSDNGDGTWDVTINFNAVATMSLKSLSELKILSLSKTYFLYSYNLKVDNINNPGSW

SQRVTVTPRSVGNYKTCMPQFTIQFDWCSAGVTDQSECQNWKYQGSYDYITGCDNFDQST

GFSQKDAPDYCWDVNVPSSSSAIVSSSAAPSSSSVPAAPSSSSVPVAPQSSSAPAVPSAP

ATPNTPTDPDTINACPNQKFTFHAQVVNFPQATITVTDPSDNGDGTWDVTINFNAVATMS

LKSLSELKILSLSKTYFLYSYNLKVDNINNPGSWSQRVTVTPRSVGNYKTCMPQFTIQYD

WCSAGVTDWSECQNWKYQGSYDYITGCDNFDQSTGFSQKDAPDYCWNEPKSSSSQSSATY

LTPHNFDVTSGDFSVPASSSVVSSQASSSVVSSQASSSVVSSSSSEVTSETPNDPQNRPP

PRTVGPEVSTVTFSSDSASSAISSRTARLSSSAASSGLSSSEVSSSEVSSTLPSSDASST

EASSVVSSDASSAITSEFHPHTLAPKFSLVSSYDFPPSSDSISTESSSQESSVVSSTDGS

SVVSSTEVSSKASSTDGSSAISSTEESSKVSSTDVSSSTEESSVVSSTEASSKVSSTEGS

SVVSSTEVSSKASSKISSRASSTEGSSKVSSTEVSSKVSSAEVSSTEVSSKVSSTEGSSA

VSSTEGSSVVSSDVSSKVSSTEVSSRASSKVSSKVSSTEESSKVSSTEVSSKVSSTDGSS

VVSSTEVSSKDSSSTTSEFHPHTLAPKFSLISSYDFPPSSDSISTESSSQESSVVSSTDG

SSVVSSTHGSSIVSSTEVSSKVSSTEGSSVVSSTEASSKVSSTEVSSKASSKTSSRVSST

EGSSVVSSTEVSSTASSSTTSEFHPHTLAPKFSLISSYDFPPSSDSISTESSSQESSVVS

STEGSSKVSSKVSSTDGSSVVSSTEGSSKVSSTEASSKASSTEGSSKVSSTEASSKASST

EGSSKVSSTEASSKVSSTKASSKVSSSKVSSTEASVVSSTEGSSKVSSTERSSKVSSTDA

SSVVSSTDGSSVVVSSTETSSVAYPTDSSSVVSSTEASATTSEFHPHTIAPKFSLITSDE

VSSTDGSSVVSSTEGSSKVSSTEGSSKASSTEGSSKVSSTEGSSKASSTEGSSVVSSTEG

SSKVSSTEASSKVSSTKVSSTEASVVSSTEGSSKVSSTEASVVSSTEASSVVSSTEASSV

ASSTEGSSVVSSTEGSSVVSSTEASSVASSTEGSSVVSSTEGSSVVSSTEASSVVSSTEG

SSVVSSTEGSSKVSSTEASSVASSTEGSSVVSSTEASSVVSSTDDSSIVSSTETTSTWPH

TIAPKFSLVTSSEPSTEQSSVASSTETSNSFTTTFPVDPRILIESSTSSTIFSSSETPIS

SFARYTNSSIVVSTPQPSEPAFSLPPPGQPVVPAFSSGSSFDASSSTNVDSSSASPSGSF

STDSSSGSLFVSSSDLTSSSTFSSASSTIASSGSSPSPSVPGYSSTDGSSSGVPSGSIPA

VPFGASSTDGSSYSVPSGSSPAVPSGSSSTDGSSYSVPSGSSPAVPSGSSSTDGFSSVAS

ESSPADPTGSQYATTSVPSASVTSDVSTSGFTSEQPTPSAPGTTTLTITSCDKSTCTTSY

KTTGITVRTTTIGSLVTKFTTYCPLTGVSSTPSPSEGVQTPTTGSPDVPSPSTTTITETT

CDETSCTTSYKTTGVTVITTTVGSIVTKYTTYCPLTGTSPSEPLNGVSTGVPHVPAPSTT

TITEVSCDDTTCATAYKTTGLTVVTTTIGSVVTEYTTYCPLSGTHSEVVETVSTIAPTAS

APSTTTITETSCDESSCTTAYKTTGVTVITTTINEMVTEYTTYCPLSGQSTATTPVGETS

TTPAGSKETTISSVYHSGSPVSTESGVVSSPSSSPLITIASTSGGASNVRLSVASLLVLL

PLFI

>tr|C4XZ51|C4XZ51_CLAL4 Uridylate kinase OS=Clavispora lusitaniae (strain ATCC 42720) OX=306902 GN=URA6 PE=3 SV=1

MYSRTVRVTPLARFARSRNLARVRFASGRPSPPSPSQGKILAALGLLAIGTTIVASVYNK

SHPMPMVEPAKNPAAFNKEAASGKPLKKPTFSPDEVTVVFVLGGPGSGKGTQCAKLVAEK

GFVHLSAGDLLRAEQKREGSKYGGLIAECIREGTIVPQEVTVALLEQAITEEHKKGNSRF

LVDGFPRKMDQALVFEEQIVPSAFTLFFECPEQVMLDRLLQRGKTSGRTDDNIDSIRKRF

RTFVDTSMPVVDYFDKKGKVVKLSCDQPIDSVYGEVKAALEKKGVV

>tr|C4XZ53|C4XZ53_CLAL4 PAN2-PAN3 deadenylation complex subunit PAN3 OS=Clavispora lusitaniae (strain ATCC 42720) OX=306902 GN=PAN3 PE=3 SV=1

MNINPDSARDIPCKNVLIYGYCKYENKGCAFAHPASSKSSTIKTNSANASTIATGDATDA

SPPAEPKRKFNMNTPSFQPSVSGLTNKFSALSPKLKEIPVFVPASMESSENPITQSSIAN

VSTATNSGSTGVPGASVNASTPAENNSPFTSRKFNVSTPSFTPSNTTEAPLSSPAANFTS

SAPTQPKLANPYASSSAADFMYHHSSGASAYPLHHHLYAPAPPPRLAMPLGRHETNASAM

FIPNDLRELLTRKNEATLQTMPHSTLPDHVGVYHTLVPIDSSFDNVSKEYHLPSHVYKVL

SNADGLPYAMRRIDYGTSLRIVNELPFRTVKRWRSVKNPNVIHLQDAFTSVAFSTQGEPS

LCLVYDYYPLADTLSERHLSRKLGSKLEPVTEDLLWTYAIQITGALLDIHQAGLYAGSSI

SLSKILVTNKNRVRLGAVCVDDILEFENLDSERNEIGSEAVVEKLQLGDVIRFGKIILDL

AATTLPAGLRNGSIEEMIDNLSRSSSISFSGEFLETLRVLNSAESEFSLINFYTRYLSQR

ALKLINGLQDSTDYYEGQLSSELENARLFRLMTKIDYLLSQSDKEDEVNGNLFVIKLFRD

FVFQTTDETGKPKVDLSRVLVNLNKLDVGVDEKILLVSREEDSCLMVSYKEIKDIMDQTF

RNIFR

>tr|C4XZ89|C4XZ89_CLAL4 Glutathione reductase OS=Clavispora lusitaniae (strain ATCC 42720) OX=306902 GN=CLUG_01271 PE=3 SV=1

MFAPSKQLLPKPVSRLLSINKHLTMAPVQDKIAKHYDYLVIGGGSGGVASARRAASYGAK

VLLIEGKYKQLGGTCVNVGCVPKKVMWYASDLASKRGHLKAYGFAGGDGHVKYGDFDWSL

IKSKRDAYVKRLNGIYERNLEKENVEYVYGFASFANSNGDVEVTLSADQEVPFLSKSFKK

DEKLLFSADKVLIATGGQPIIPPSVEGSELGINSDGFFELEKQPKSVAIVGVGYIGVEFA

GVFSSLGTETHLVARGDTVLRAFDDIIQTTVTDTYVNKLGVNVIKNSGSVTKVEKAGDKK

KVYLGNGDVLEVDVLIWTVGRKALTNMGLEKVDVKTQENGSIIADDYQQTSNPKIYSLGD

VVGKIELTPVAIAAGRRLSNRLFSGQEVYANDKLDYSNVPSVVFSHPEAGSIGLSTKQAV

EKYGKDNLKIYNSKFNAMYYAMMDSDDDKVPCVYRLICAGPEEKVVGLHIVGDSSSEILQ

GFGVAIKMGATKKDFDNCVAIHPTSAEELVTLR

>tr|C4XZB1|C4XZB1_CLAL4 ATP-dependent RNA helicase OS=Clavispora lusitaniae (strain ATCC 42720) OX=306902 GN=CLUG_01293 PE=3 SV=1

MSELSKEEKLRRRQEQLAKWRSKKTSSSSQNEDREPAPLEKSSTATPEEQKKLDRQRKLE

EWKRRKQEQQEGNSSGTTDAKKEDTPLEERQRRLEEWKQRRQTTEPGKEQSKGKSSLRKS

GLLTRIPKTSTKIPHLMKRKAVFESDDESTSEPVFKKPLIKDIKFGDANDAKEGNEERAE

VEAEDALDAFVQHLENEQMPELMSDVAMSEHEASDTEDSEAESDSDDKLLSLRLKNLQKG

KELSVVDHDSVDYMPFRKDFYQESQSVSDLTEEEVEELRLQMEGIKVKGSNCPRPIWMWS

QLGFSSTIMSLIEEKLEYKKPTPIQCQALPIIMSGRDILSIAKTGSGKTMAFVLPMLRHV

QEQPPLSKGDGPIALLLSPTRELALQIFKQLSIFTKKLGISACCCYGGSSIELQIAELKK

GCQVVVSTPGRLIDLLAANGGRVCNLRRVTYVVLDEADRMFDFGFEPQVNKIFSQVRPDR

QSILFSATFARKMEMLAKAILHDPIQVIVGGISVVSQEITQRVELFEVTENDNEDTIEKR

KFEKLLKVLKEFPSTKKLIFVEKQDSADKLMVKLLTENIPSLTIHGGKEQIDRKYAIKNF

SDNDSGVDVLIATSIAARGLDVKGLGLVVNYDPANHMEDYVHRVGRTGRAGNTGVAYTFV

TSKQERPITDLVKAMRLSKMPEDAIDQRLVEISNGFLTRVKDGEEKFRFGFGGKGLNKLD

EIRNSHMALERKVYEAESGSISKNENTKTKWTNTEGTPEINGEKIDLPEFKIIEGRAEET

SGPDKCKFHSRITINDLPQRARWYVVNRDSLGSIIEATSTSITNKGQYYAPNTKIPITTK

IGGRNVPAPPKLYLLIEGLTEESVNEANKMIKQKMIEGLEIASKEENSIPTGKYSV

>tr|C4XZC4|C4XZC4_CLAL4 Endonuclease III homolog OS=Clavispora lusitaniae (strain ATCC 42720) OX=306902 GN=NTG1 PE=3 SV=1

MPSKRSLIAEERPKRVRRSPRLVLDAETLKTEPEIKIESSLDKPLKEVFSDKSVTTTSFK

HEVDDAKVKVEIEASVSGNTDTFASPEIESKSNVFQCVSPSDILPGQPKNWNLIYNEVVK

MRALIVTPVDTMGCERIPETIAPGLIRRDPRAYRFRLLVSLMLSSQTKDEVTYVAVENLN

NFYKTKGFDGLCIEAILKSTEAEIDFCIQKVGFHRRKAVYIKKASELLNEKFNADIPKNI

KDTISLPGVGPKMGHLLLQAGWRINSGIGVDVHLHRLAQMWGWVPKSDKPESTRLALEDW

LPKKYWSDINPLLVGFGQTVCVPNAGNCDVCTLAAGLCSKANKKLSNAAVTEARLAKLAK

QRGDISGLIKLKHELQMLKKDSNV

>tr|C4XZC6|C4XZC6_CLAL4 CCR4-Not complex 3'-5'-exoribonuclease subunit Ccr4 OS=Clavispora lusitaniae (strain ATCC 42720) OX=306902 GN=CLUG_01308 PE=3 SV=1

MNINHKFQQSQQPGLPSQQILLQQLQQGQPQTSSQNSGSQANGSKSQFVNQDGLYPDSSF

YQRGGPSGAPGFYPQQFQQQGQQQQQQQQQQQQQQQQQQQQQKQQQTQQFGQYPGQNPIL

QQQGPLNTQKFMNQQSLLQQNLLQQQQQQQQQQQSQVSQLSNVQQMAGMPGFQTVNKVNS

INVDDPGSMYWQHQVSLCQLSRSEDMPHFYARQYAQNSRKNKNPYSETKSVTLIDATKTI

VAALEEQEKQQKASQTAAAPTALMRNKKVDLEEDEEQRVMARTQGKQLWCHLDLSGQGLL

NLSPKLFQYDFLESLYLNNNKLTSVPSVIKKLRGLRVLDLSHNRITEVAPELGLCYNLRY

LYLFDNNIKTLPNEFRNLIELLFLGIEGNPIDLKIANLIAEKGTKALISYFRDSEPTYPE

PNPRAWLLLKDDGEIIDPTTDPNAYANDRIGSKNSDTFTLMSYNTLCQHYATTKLYKYTP

SWALEWEFRRNSLKEEILRYNTDLICLQEVETRTYHEFWLPIMESAGYNGFFFCKSRSKT

MSESESKKVDGCATFFRASKFQLIQKQHLEYNTVCMGSDRYKKTKDLFNRFMNKDNIALI

TYLQHIETGEKIVLVNTHLHWDPAFNDVKALQVGILLEELQSMMKKFQHTNSVDDIKNSS

LIICGDFNSTKSSAVYQLFSTGAVSKHGDLEGKDYGRFTDEGFHHNFKLKSAYDSIASDF

PFTNFTPTFTEVIDYVWYSTNTLQVKGLLGKPDEEYYSHHVGFPNAHFPSDHIPLVTKFQ

IHKKGVAPGTKKAEFKPDFKSGSSRKT

>tr|C4XZJ1|C4XZJ1_CLAL4 tRNA-dihydrouridine(16/17) synthase [NAD(P)(+)] OS=Clavispora lusitaniae (strain ATCC 42720) OX=306902 GN=CLUG_01373 PE=3 SV=1

MTAEKLNGRALFEQIGRPKTIVAPMVDQSELAWRILSRRYGAELCYTPMFHARLFATEEK

YRNKMWSEWDGDREKDRPLVVQFCANDPEYLLQAAKFVEDKCDAVDLNLGCPQGIARKGN

YGAFLMDDWDLVYKLIRKLHDNLKCPVTAKIRVYDDWEKSLEYAKMVLSAGAQFITIHGR

TRDMKGQATGLANWKILRYLRDNLPSDQVFFANGNILYPSDINRCIDEVSCDAVMSAEGN

LYNPGVFWTKDDDKDKQFARVDKMLREYFDIVRTCPGEASRVAMKAHFFKLLHAFLNVHK

ELRPIIGQTSVNADFSVWDDIVKKVEAIVEDIFAQPNIAELDVITEGELQSWGGKYKTIP

YWRCQPYFRTVDGEKQNTRVLKVAASGVAEAKNEKKRPAEENTEPTKKIAVESVH

>tr|C4XZU7|C4XZU7_CLAL4 Ketol-acid reductoisomerase, mitochondrial OS=Clavispora lusitaniae (strain ATCC 42720) OX=306902 GN=CLUG_01479 PE=3 SV=1

MSFRNSAIRVARQASSNAAKQIAAKRALSALASRTTPRLAKSVAPLATRGIKTINFGGTE

EIVHERSDWPKEKLLEYFKDDTLALIGYGSQGYGQGLNLRDNGLNVIIGVRKNGASWKAA

IEDGWVPGENLFDVKEAIQKGTYVMNLLSDAAQSETWAELKPLITKGKTLYFSHGFSPVF

KDLTHVEPPSDVDVILAAPKGSGRTVRSLFLEGRGINSSYAVWNDVTGKAEEKAIALAVA

IGSGYVYQTTFEREVNSDLYGERGCLMGGIHGMFLAQYEVLRENGHTPSEAFNETVEEAT

QSLYPLIGKYGMDYMYDACSTTARRGALDWYPRFKDALKPVFEELYESVKNGSETQRSLD

FNSQPDYRERLEEELETIRNMEIWKVGKEVRKLRPENH

>tr|C4XZW8|C4XZW8_CLAL4 Eukaryotic translation initiation factor 6 OS=Clavispora lusitaniae (strain ATCC 42720) OX=306902 GN=TIF6 PE=3 SV=1

MASRTQFENSNEVGVFSKLTNSYCLVAVGGSENFYSAFEAELGDVIPIVRTTIAGTRIVG

RMTAGNRRGLLVPTQTTDQELMHLRNSLPDSVKIQRVEERLSALGNVICCNDYVALVHPD

IERETEELIADVLGVEVFRQTIAGNVLVGSYCSLSNQGGLVHPQTSIQDQEELSSLLQVP

LVAGTVNRGSSVVGAGMVVNDWCAVTGLDTTAPELSVVESIFRLQDAQPDAITGNLRDTL

IETYS

>tr|C4XZY5|C4XZY5_CLAL4 3-ketodihydrosphingosine reductase TSC10 OS=Clavispora lusitaniae (strain ATCC 42720) OX=306902 GN=CLUG_01517 PE=3 SV=1

MYWSKSQFQPQGKVSLIIGASQGVGADLALRLYEKGGIVILVARTESKLKEQVNRIVKIA

GEQTNSKGEKNIEYYVCDVANYDSCHQMWSHLIHSRNMDPDFIFCCAGTSVPKLFADLTG

KELANGVNINYMTALNTVHAGFKSVVEKHKDLKPQEFKKRHIILFSSVLASYSFTGYAQY

APMKAALVSLSNLLRQEMGAFNYRVTCVYPGNFESEGYFEEEKTKPAITKKIEGASKPIS

SLHCCDIILDQLSKGYDSIYTDFIGWVLGCSSLNVNPRNWGLFQVIVAFIFSIIAPVAES

IVSSDVKKFFQEKKEPSDEQSN

>tr|C4Y057|C4Y057_CLAL4 Crossover junction endonuclease MUS81 OS=Clavispora lusitaniae (strain ATCC 42720) OX=306902 GN=CLUG_01589 PE=3 SV=1

MIWLCRYEMGDFSEQKNRLRKSGINNVYYLVEEGGMADTERIMEMRKSIETSISMVITVS

NLFLHRFRRTDDTIDWLLTMSNILKEKYSQKRLIVIKPRTIHSQEEYLHMLQEFREKFDN

KEQAYECVHMLPVYQATLAKSNMRTVKEMFILMLMSVKGISLEKAVVIQSHFGTPKKLID

YYSIENRSLSESEKGLLISKLFQSQIGSKKINKAASIALYESWGKL

>tr|C4Y058|C4Y058_CLAL4 Crossover junction endonuclease MUS81 OS=Clavispora lusitaniae (strain ATCC 42720) OX=306902 GN=CLUG_01590 PE=3 SV=1

MISYSDAGIYCDSSFTSNPSARDFYSAWDGIKTLLKRELVECIGRAPKMYFLTESGKKMA

QLIIEQEGIESAPTDEVDFSFDNGIRISSDSPCMGSEGLFVSDSIVEKGHDAANKTVNGI

SYDIWVPGDFDIVLLVDNREIRSQNERDFFQRRITEKDVECDVRSLSVGDILWVAKHKTT

GKEVALNYVCERKRLDDLAMSIRDGRFLRTEK

>tr|C4Y087|C4Y087_CLAL4 Inosine-5'-monophosphate dehydrogenase OS=Clavispora lusitaniae (strain ATCC 42720) OX=306902 GN=CLUG_01619 PE=3 SV=1

MVLDCSSATSHLKTYPKKDGLDIRSLIDSSNFGGLTYNDFLILPGLINFPSSEVSLDSKL

TKKITLKSPFLSSPMDTVTEENMAIHMALLGGIGIIHHNCSAEEQAAMVKKVKKYENGFI

SDPVVVSPSVTVGEIKKMKERIGFSSFPVTENGKTGGKLVGIVTSRDVQFHDDDSTPVSE

VMTKDLITGKQGITLTEGNSLLRSSKKGKLPIVDSEGNLVSLISLTDLQKNQSYPDASKS

FHSKQLLCGAAIGTLPADRERLDKLVEAGLDVVVLDSSNGSSIFQLDMIKWIKNKYPDLE

VIAGNVVTREQAALLIEAGADGLRIGMGSGSICITQEVMACGRPQGTAVFNVCEFAKQFG

VPCIADGGIGNIGHIAKALALGASCVMMGGLLAGTSETPGDYFYRDGQRLKTYRGMGSID

AMQQTSTNANASTSRYFSESDKVLVAQGVSGSVIDKGSITKFVPYLFNGLQHSLQDIGVK

SVDELREKVYEGEVRFEFRTASAQLEGGVHGLHSYEKKLHN

>tr|C4Y098|C4Y098_CLAL4 DNA replication licensing factor MCM5 OS=Clavispora lusitaniae (strain ATCC 42720) OX=306902 GN=CLUG_01630 PE=3 SV=1

MSYERAEVFSAQVLPGEEPQDSSFNEITKAFRSFILEFRLNNQFIYRDQLRENLLIKNFY

LKVNSEHLIGFNEELNKKLSDEPGEMVPLFENAITDIAKRIAYLSNDDVPRDFPICQLIL

YSKSSTISIRNLDSEHISKIVRVSGIIISASVLTSRATMVSLICRNCKHTMKMKVASGFG

SLNLPSKCLATHNHDEVHSQQKCPPDPYVVVHDKSTFIDQQVLKLQESPDAVPVGEMPRH

ILVQVDRYLTNQVTPGTRVTLIGTYSIYQAKQRTSAAVNTVAIRNPYLKVLGIQTDVDTA

AQGLSFSEEEEEEFLRMSRMPNLYEVFSKSIAPSIYGNDDIKKAITCLLMSGSKKILPDG

MRLRGDINVLLLGDPGTAKSQLLKFVEKVSPISVYTSGKGSSAAGLTASVQRDTQTRDFY

LEGGAMVLADGGVVCIDEFDKMRDEDRVAIHEAMEQQTISIAKAGITTVLNSRTSVLAAA

NPIFGRYDDLKSPGENIDFQTTILSRFDMIFIVKDDHNESRDRSIAQHVMNIHTGNSAEN

EESEGEIPIDTMKRYIQYAKSKCAPRLSPEASEKLSSHFVAIRRRLQVNEADMNERSSIP

ITVRQLEAIIRITESLAKLTLSPIATVDHVDEAIRLFTASTMNAVDQGVQSGSLMATGKF

AEQIKLVEHELRRRLPIGWSTAYKTLRREIVDSGKAPAEALDKALHIMERHEVIRFRHQR

QNILRVGV

>tr|C4Y0A1|C4Y0A1_CLAL4 Pescadillo homolog OS=Clavispora lusitaniae (strain ATCC 42720) OX=306902 GN=NOP7 PE=3 SV=1

MARIKKKGKTGSAKNFITRSQAIRKLQVSLADFRRLCIFKGIYPREPRNKKKANKGSTAP

TTFYYSKDIQYLLHEPVLAKFREQKTFQKKLQRLLSKQELNDADKLERTRPKYTLDHVVK

ERYPTFLDALRDLDDPLNMLFLFANMPATTSVSHRVTKEASKLTNQWLAYVAKERLIKKV

FVSIKGVYYEASVKGQEVRWLVPFKFPANIPSDVDFRIMLTFLEFYSTLLHFVLFKLYSD

SNLVYPPPIDAEKLKGIGGLSSYVLKSKDQGIGSLLPAEKKEESVEDAEDSAEPETLSAQ

EIQNAIAADAKEDDQSEPVENVESVELDKFASANKTTGDLLAQPSEFASPSSTLFSKFTF

FVGREVPLDIMELCILSAGGVVVSEIALDEMKINHPEAYAKLDLSSITHQVVDRPKVVNK

IAGRTYVQPQWIFDSINKCELLPVGAYAPGETLPPHLSPWGDRAEYNPEAEPVEEGESGS

EEEVEIEGEELPDEDEEEDEALREQKELEMEAAGIQYSEAKDEQPKKKAKKTKKVGRRGR

EGVEDDHDVSQEEEVIQQDAIRYR

>tr|C4Y0I9|C4Y0I9_CLAL4 NAD(P)H-hydrate epimerase OS=Clavispora lusitaniae (strain ATCC 42720) OX=306902 GN=CLUG_01721 PE=3 SV=1

MFKTLSSKAAAQLDQELMSTGSFSIDQLMELAGLAVAKSIYKQYPPQNSKSKVLVLVGPG

NNGGDGLVCARHLKLWRSYEPVIFYPKRPKKDLYINLMKQLNDLGVQEISTLEEVKNLLQ

GSSVSVIIDALFGFSFKPPIREPFDDLISYLSSHSSEIAPVVSIDIPSGWDVDEGPLDTD

IKASMLVSLTAPKPCALKFAKQGKPHYLGGRFINDNVASKYDIVDLIAKYKEDELVVKL

>tr|C4Y0K9|C4Y0K9_CLAL4 Eukaryotic translation initiation factor 3 subunit G OS=Clavispora lusitaniae (strain ATCC 42720) OX=306902 GN=TIF35 PE=3 SV=1

MFQSWADAEDDIPTPEVTVNPDGTKTVISYRLNAKGQKVKITQKIKEVKVKERVHPSIAI

RRGWAKYGKEKHTPPGPDTRTTQLGEKVELKLGASWKELEKKEEEEKLEQKASLVSTQRL

RCRTCEGDHFTSKCPFKDTLGAETAGGAGTPEPSESGKYVPAHLRKDAPSKESRDDSCTL

RISQLNTFVNEQMLREELLAKYGPLQRATVLYNKETGESRGFAFVTFATEQKAAQALEEL

NGKGYHSLILRLEWSKRKKT

>tr|C4Y0L0|C4Y0L0_CLAL4 Kynurenine formamidase OS=Clavispora lusitaniae (strain ATCC 42720) OX=306902 GN=BNA7 PE=3 SV=1

MSKDCCSFNFFFFFFFFFDLLWSLGEQVQYKRPVPDSIGTGLDVATTNNSVVVFSSMTSI

SKEIAYGTHPLQRIKFFHYSPANTQCLVFVHGGAWRDPTNTYDDFNQLAQHLQSQIPNFN

FVGVNYRLSPEVKHPEHLMDLGAALSFLSTSYNMSNCVLLGHSVGATLLMQLVNYNKIIE

LSGVTVPAPLAVKISGMVFVDGIYDMVDLIEEYGSSYEEFVDNAFTNADGYINASQMTWK

CKDNFDAKFKVLVVQSLEDELLSLRQTKRFVEYLKNQKVIYELDTGDWGRHEEVYRREEL

AQTVKKYLESL

>sp|C4Y0Q6|MMM1_CLAL4 Maintenance of mitochondrial morphology protein 1 OS=Clavispora lusitaniae (strain ATCC 42720) OX=306902 GN=MMM1 PE=3 SV=1

MAGKADLGHTGISDNIVERQIFVPQPNNAWSFTQGLMCGQASVVVVLLVFIKFFVFSEAP

PSSGAAKSKKKDISGVIVKREAKNTDDDDGVENGDSAKLATILEKTYYDVNNHNPESLDW

FNVLIAQTISQLRCEALLSDNIYHSLNDFLETSDLPDFMDKITLTEIDIGDDFPIFSNCR

IQHSADGTGRLEAKIDVDLSDTLTLGIETRLLLNHPRPLTAVLPVQLTVSMVRFSGCLTV

SLVNTNDTDFVGTSKEENSSGGGTALMFSFSPDYRLEFSVKSLIGSRTKLQDVPKISDLV

DSKLRNWFSDRCVEPKFQVVRLPSMWPRSKNTREPVGAGKTEKVNGNE

>tr|C4Y0R8|C4Y0R8_CLAL4 Glutamyl-tRNA(Gln) amidotransferase subunit A, mitochondrial OS=Clavispora lusitaniae (strain ATCC 42720) OX=306902 GN=HER2 PE=3 SV=1

MASDKFNSIVHVLAQAARPLSPTGVLSGKTFAAKANIATSRAPVDCASRVLDSYTGPFTS

TAVRLLEQAGAVCIGTTNMDEFGMGSASTHSSHGAVVNPQYYPANHVAGGSSGGSAACVA

AGLADFALGTDTGGSVRQPAAWCGVVGFKPTYGSISRFGVVAYAQSLDTVGILARSVRTT

RDVFGVLDKWDDNDVTALPLSVRGRKAKEGPWVVGVPEQMLLGEVGPEALEALELALVGL

KKLGHKIVPVSIPALSRSLSAYYTLATAEAASNLARYDGVRYGENEKGHSFGVEVRRRVL

SGTYTLSSESGSHYKHATLVREKLVEELNCVFASAHSFVGVGEKHKDVARNGPDSGSGVD

VLLAPTVLGRAPERDVYDQITRNFIEGYAGDVLTVPASLAGLPTVSVPFNGVGIQVMAQH

GDDATALSVSEAVETIFAQ

>tr|C4Y0S9|C4Y0S9_CLAL4 ATP-dependent 6-phosphofructokinase OS=Clavispora lusitaniae (strain ATCC 42720) OX=306902 GN=CLUG_01811 PE=3 SV=1

MAIEGVSFISLVTADPTKFKEACEFYRRLGFRLAKNYSKLSSSGAAVAHHPKLSSGLCTD

SIREVWLESFPLQNTDENGCVVPWQELRFYNGDHTAPLAPSTCLKILLSPSEVIEPVFAG

LEFFSPELGKVAEILGSKEVSQEDEKNPKAKRVLATDPLGNHVSFSNTSEGRVFNSTKEY

IDYRTQLLVKEEEAQEPAPAAPTAAKKKKIAVMTSGGDSPGMNAAVRAVVRAGIYLGCDM

FAVYEGYEGLVRGGDLLRRMEWDDVRGFLSFGGTNIGTARCMAFKERKGRLAACKNMVDA

GVDALVVCGGDGSLTGADLFRSEWPSLIDELAQNGDITEEKAEYHRHLTIVGLVGSIDND

MASTDATIGAYSSLERISEMVDYIDETASSHSRAFVVEVMGRHCGWLGLMAGLSTGADYI

FIPEKPPRHGEWQEELKKVCSRHRGYGKRNTTVIVAEGAIDDQLQPITSEHVKNVLVDMK

LDTRITTLGHVQRGGTAVAYDRLLATLQGVEAVHAVLESTPETPSPMIGILENKIVRQPL

VESVRLTKSVAEAIENKDFEKAMSLRDSSFANAYETYLSTAIHDDGTQLLPENERLNIGI

VHVGASSSALNATTRAAALYCLSKGHKLFAIQDGFSGLAHSGVVKELSWMDVDGWHNRGG

SEIGTNRSLPSESFGNVAYYMQRYQFNGLLILGGFEAFTALHELESHKHEYPIFNIPKVV

IPATVSNNVPGTEYSLGSDTCLNQLVNYCDAIKQSASSTRRRVFVVEVQGGNSGYVACYA

GLVTGAMAVYRPEQNINLKSIREDIDLLTAVFKNDRGEDKNGKIVIRNEHASSVYSTELL

ADIFRDDSHGRFETRTAIPGHVQQGFTPSSMDRSYAVRFAVESVKYIEEWSRKAFNTIHR

DHTSHHSVNNIKMIELMRKEEKASVVIGIQGASLKFSDVNDLYEKDADVSLRKGRTVHWS

RLAEVGDILSGRLMLREGKA

>tr|C4Y0T9|C4Y0T9_CLAL4 ATP-dependent RNA helicase OS=Clavispora lusitaniae (strain ATCC 42720) OX=306902 GN=CLUG_01821 PE=3 SV=1

MAKKVKSSKRNEQPAKRKHEDEDVSNSDSEKLVNELDEDFDEVAQLLGDNVQDPEKKSSK

QQKKEEKLAEEEVTQSKAIKPAAQENDENDEGEDNYDFEEAGFSEPTLKAIRQMGFKKMT

KVQAKTIPPLLAGRDVLGAAKTGSGKTLAFLLPAIELLYSLKFKPRNGTGVVIISPTREL

ALQIFGVARELMEHHTQTFGIVIGGANRRQEADKLVKGVNLLIATPGRLLDHLQNTQGFV

FKNLKALVIDEADRILEIGFEEEMKQIIKILPNEDRQSMLFSATQTTKVEDLARMSLRPG

PLYINVVPESAASTADGLEQGYVVCESDKRFLLLFSFLKRNAKKKIIVFLSSCNCVKYFG

ELLNYIDLPVLDLHGKQKQAKRTNTFFEFCNAKQGILICTDVAARGLDIPAVDWIVQFDP

PDDPRDYIHRVGRTARGTQGKGKSLMFLTPSELGFLRYLKAANVPLNEYEFPANKIANVQ

SQLTKLIKSNFWLHQSAKDGYRSYLQAYASHHLKTVYQIDKLDLVKVAKSFGFDVPPKVN

ITIGASGKSVEKKHKKQRRN

>tr|C4Y0W2|C4Y0W2_CLAL4 Phospholipid-transporting ATPase OS=Clavispora lusitaniae (strain ATCC 42720) OX=306902 GN=CLUG_01844 PE=3 SV=1

MSHSGSTPQTRRKRGLSLRSQLFTKALASPSQPPIELASGPVHEEHTNDSSSKAGTTSTS

SSSFNDTSSYTSNISNAAAHASHTNNADANPYVPGTNTNHTAGKPPGPPPTIVVEEAEPY

YPPYSQYPQSAFSTTQLTALSSASSSASDARQPPKKSLWERARALVRPPAPDYSGAGREI

PVAVRQLSLTDRRTGREYCSNVITSSKYTVYTFLPKQIRAQLSKVANCYFLVVAIMQMVP

SWSTTGKFTTIIPLMIFMSISMAREGYDDWRRHGHDREENNKATHVLREDADLERFDTQS

IATIMTETLPTPSTFAGVAEPLSEDSVFSESARRRYNLRLDRAKWKDVRVGDVLCIKEDE

WVPADVLLLAVDGDDAYVETMALDGETNLKPKIAHAQLRALASRAASMKSIHTVVTAEDP

NSDLYNFEGHVALDGSTYALGPDNIVYRGSVLRNTRAVLGLVVFTGEETKIRMNNLRSPR

VKAPKLQRNINLIVLFMVCVVLALSAFSTMASRIYYSRDHKKMWYMFDSDVGVAPTFMGF

IIMYNTLIPLSLYVTMEIIKVMQLLFLQFDIDMYHVESNTPADAKTATILEELGQVSYIF

SDKTGTLTDNKMVFRKFSVCGCSWLHELDLLEKERSDPSDMARVASLRSIVPVETSTSII

RNSLDLTTVPTRSTWKSNAQPLKEQDIPTSLFLLKYVQSHPQTLFAKKVTFFLLSIALCH

TCSPKRVDSKSTNTSSYSLNEQEEPDIDHLETDSQIRYQASSPDELALIQAARDLGFILL

DKENNIAQIKTYPNGFNAAPKIEQYEVLDFIEFSSVRKRMSVIVKFPDSRVGVICKGADN

VIIDRLKNAEIAKKKARELLMNSADRKTMEAELVLQSRMSADMESRKSGGSLRASLDIAE

RMGSIDGIVNKDDHDLSEIAKKARRSLHIQQAKRYSLDESQIEQETGPNGPKVAIPNDAL

LVNEEYLIEKTLEHIEDFSTEGLRTLLYSFKWLSHGEYEEWAKEYQDAKISLVDRAKKVE

EVGEKIETNLQLLGATAIEDKLQEGVSEAIEKLSRAGIKLWMLTGDKRETAINIGYSCRI

IKDYSTVVVLSMDEGSETLVQRITEAGREMKAGRIAHCVVVVDGGTLTSIENDPTLFSIF

IELCIQVDSAICCRASPSQKAKMIESVRALKKRDVTLAVGDGANDIAMIQSADIGVGITG

QEGLQAARSADYAIAQFRFLLKLLLVNGRYNYVRTSKFVLCTFYKELLFYLTQCIYQRYT

NFTGTSMYESWSLSMFNTLFTSLPVICIGMFDKDLRPATLLAVPELYTTGRMYKAFNLKV

FLSYMVLAAMQSVGISFLAYFSWGFSAMRDNSLMPLGNCLFWALVIVINGKIQFLEMRNK

QWLAFASFFISTLGYGVWNVLIMLLHRGKNDIIYYVSYGLLEFGRDVTWWATLLMLFSVL

LLFDLLVKVLKFVIKPNDTELFQMFEKDINMRRTFEQNAMSELKQGWFMAKEPSTTKVHI

SKFIHKVFKIDIDRVVPNEVNQDHEGSAMQRKRAGTNTLPDELPPSGEGSAVKEADYESP

DLEGYEILPSGAKVKVKKEGIMSRFERKLHKKNEDPDIDEVLENRMREFEN

>tr|C4Y167|C4Y167_CLAL4 3-oxoacyl-[acyl-carrier-protein] synthase OS=Clavispora lusitaniae (strain ATCC 42720) OX=306902 GN=CLUG_01949 PE=3 SV=1

MTRVVVTGMGLITPLGVGLRSSWANLIANKSGLVATTTFDDYEQGWSQIPSKVVGRVPVK

SAGGKKVEPKGGENGSPATADRDASSASSAPGWCASDHMSAGDARRMAPFAQYAVAAAAE

ALADAQFVASDVDPTRVGVAVGSGIGGFGDAYDNSTAFASEGYRKVQPLFIPRLLANMAA

GHISIRHGFGGPVHAVSTACATGLNAIGDAYNFIQNGYADVMVCGGTEASLHPLALGAFA

RARSVVAGGEDDPQGASRPFDGARRGFVLGEGCGILVLEKLVHAQARGAPIYGEVLGYGL

AGDAHHITAPAESGDGAFRAMEQALSRAGVAPSEVDYVNAHATSTPLGDRAENTAMRRLF

GSSSRLAVSSTKGAVGHLLGAAGAVEAAFTLMAVRHDTVPATLNLTTPGGHPEDDPSAFC

FDYVPNHSRMHRTDLALCNSFGFGGVNTSVLFGKMR

>tr|C4Y170|C4Y170_CLAL4 Autophagy-related protein 9 OS=Clavispora lusitaniae (strain ATCC 42720) OX=306902 GN=CLUG_01952 PE=3 SV=1

MSRFDSQDSQSNTFGASDTFLSRVFGLHSVYNQPHDNYQLYEPQTSFSTQDGNVGSSAPP

SVSNVSKPDKNLLDSESDSEFSSDSDSPASPSLERADPAAFLSRPDPEELSSRSAPKLKF

SLPHPGQKKSSDRDLEAGLPLHTTPQQVSHRKDNDTQPPKVQWRRTPRRAVFPPKERALY

LWANIVNMDEFLIDVYFYYRGKGFANIVAGRVVDILILVFILSFTTFLKWGIDYDRFFNH

WSSNSNTNLTLQDLVIPNFFFSAVPFPVKVFLFGFACYIALRLVQLYLDYKYKLAELQNF

YKHLLNISNDIELMTISWSTIVERLMLLKDYNSLTSSTPNAPPHYITDLNSKVRLNAHDI

ANRIMRKENYMIAIINKNVLDLDIPVPSFLSSILSSKSVLTRTLEWNLKLCINNFIFNEK

GQINPSILKESSRNQNAQDLSSRFKMAAIINLVFCPFIVVYFVLLNFFRYFNEYKSNPAS

LLGLRQYTPWAEWKLREFNELPHFFIKRLHLSIGPANTYINQFPGGVLVMNVMSLVNFIS

GAILAVLVLMAVFFEDEEHNFWAFEITENRSTLFYISVFGSVWAVTANSLNTSTTTNSAE

NANTQGVSFFYDPEASLRYVSQFTHYLPSSWNKRLHTVEVKNEFCELYSLKILVILNEIL

SLILTPFVLWFKVSNSSSAIIDFFREYTIHVDGLGYVCYFAMFNFEEKDKNMMYDLNKRS

RKKPIKQGTSNAKAKSKLSDTDESSSDESDADLGDYYQDDKMIKSYMYFLESYGKAAEPK

RNGAALASTGVEGSSSKTRNAPVNRNVPTAQQDFSGYGGSPLESIYESAYSIKSEKMPRK

KGMLGMINQFSRQGFGS

>tr|C4Y1D6|C4Y1D6_CLAL4 Elongator complex protein 3 OS=Clavispora lusitaniae (strain ATCC 42720) OX=306902 GN=CLUG_02018 PE=3 SV=1

MPSLPPEKERFLQCCSDIASELVASLKSNKDTNLNGVVSRHSKKLKLKHQPRLTDIIAAI

PDQHKKYLLPKLKAKPVRTASGIAVVAVMCKPHRCPHIAYTGNICVYCPGGPDSDFEYST

QSYTGYEPTSMRAIRARYDPYEQARGRIDQLRSLGHSIDKVEYIVMGGTFMSLPVEYREH

FIAQLHNALSGYSGTDLTESIRFSQQAQTKCVGITIETRPDYCTETHLSDMLTYGCTRLE

VGVQSVYEDVARDTNRGHTVKAVCETFAVAKDAGYKVVSHMMPDLPNVGMERDLQQFVEY

FENPEFRTDGLKIYPTLVIRGTGLYELWKRGLYKSYSANALVDLVARIMALVPPWTRIYR

VQRDIPMPLVTSGVENGNLRELALARMKDFGTSCRDVRTREVGIQEVHHKVAPDQVELIR

RDYYANGGWETFLSYEDPKQDILIGLLRLRKAAKPFRRELKGQRTSLVRELHVYGSVVPL

HSRDPRKFQHQGFGTLLMEEAARIAKEEHGSTKISVISGVGVRNYYARLGYELDGPYMSK

ML

>sp|C4Y1F8|MAP2_CLAL4 Methionine aminopeptidase 2 OS=Clavispora lusitaniae (strain ATCC 42720) OX=306902 GN=MAP2 PE=3 SV=1

MASAQTGTEMSPHHVTRTYKHENFLSFISTMSEKETVETTQEPKQVVEPTQELEELAIDG

DQAAAKKKKSKKKKKKAVSLDKTYADGVFPEGQWMEYPLEVNSYRTTDEEKRYLDRQQNN

HWQDFRKGAEVHRRVRQKAQQQIKPGMTMLEIADLIENSIRTYTGNDHTLKQGIGFPTGL

SLNHVAAHYTPNSNDKVVLKYEDVMKVDIGVHVNGHIVDSAFTLTFDDKYDNLLTAVREA

TYTGVKEAGIDVRLNDIGAAVQEVMESYEVELDGKTYPVKCIRNLNGHNIGDYVIHSGKT

VPIVANGDMTKMEEGETFAIETFGTTGKGYVIPQGECSHYALNQDIDGVKLPSERAKSLV

KSIKDNFGTLPWCRRYLERAGEDKYLLALNQLVRAGVVEDYPPLVDTSGSYTAQYEHTIL

LHPHKKEVVSKGDDY

>sp|C4Y1G1|SHO1_CLAL4 High osmolarity signaling protein SHO1 OS=Clavispora lusitaniae (strain ATCC 42720) OX=306902 GN=SHO1 PE=3 SV=1

MGFRMANFLGDPFAISTVSFGVIAWIVAIAGAGSSASDNFPRFTWWGLVYEILLIIMVFL

LYLNNTIELYKFTLVGLLSVGFLYTTNSTNNLIYSSNSGNLCCAAGCILLSMLNFLWIVY

FGGHPESPSNQFIDSFAMKSSYAQQLPSEKNDDHEFAVPRSASGSQGFGVSDSRHSQLTN

SKSGYMSSSQLNGLENFSHTNVQNTGTVGASNPASMPNTVYNTNGTNTADSNFAVPVSTF

RYKARALYSYDASPDDINEISFVKDEILEVDDIDGKWWQARRANGQVGICPSNYVKLLD

>tr|C4Y1L9|C4Y1L9_CLAL4 Serine hydroxymethyltransferase OS=Clavispora lusitaniae (strain ATCC 42720) OX=306902 GN=CLUG_02101 PE=3 SV=1

MPYSLSESHKQMVEGRLADTDPEVDAIIKAEVDRQKHSIVLIASENFTTKAVFDALGTPM

CNKYSEGYPGARYYGGNEQIDKMELLCQERALEAFHVTPDKWGVNVQTLSGSPANLQVYQ

AIMKPHERLMGLDLPHGGHLSHGYQTDSRKISAVSTYFETMPYRVNLETGLIDYDMLEKT

AVLYRPKVLVAGTSAYCRLIDYKRMREIADKVGAYLVVDMAHISGLVAAGVIPSPFEYAD

IVTTTTHKSLRGPRGAMIFFRRGVRSINPKTGQEILYDLENPINFSVFPGHQGGPHNHTI

AALATALKQAATPEFKQYQEQVLKNAKVLEHEFLNKGYNLVSNGTDSHMVLVSLRDKNID

GARVETICERINIALNKNSIPGDKSALVPGGVRIGAPAMTTRGLGEEDFKKIVGYIDFAV

NYAKEIQASLPKEANKLKDFKKKVLEGEDAKLDAVKAEISQWAGEFPLST

>sp|C4Y1P0|DEF1_CLAL4 RNA polymerase II degradation factor 1 OS=Clavispora lusitaniae (strain ATCC 42720) OX=306902 GN=DEF1 PE=3 SV=1

MSQRKNHRGQQKKAAASPSKSAASSQLQTLSEMFPAWEADELAALLSEHHDDVEIVIDLI

VNNKVSKWEPIKKEPKPKKREEVITDSNATQTSHAHNDAKPKHSRERPKNEKRRERKPVQ

RKEPSAAVQAAQAASPAPAAAASTSSTSSASVPSNSWAAALSKDAKPKQKKEEPEVKEPA

QEQEQPKEEQEPAVAAATAAAASAASAPQETQAAPAAPAAPVAPTTTTTNDHAEKPATTW

ASAIKPKAKPIKKKAPEHVPEEEVSEVVVVETEVSDVVLPQQVAEVGVSFGSLALETEDV

PESQPEAQPKPEAQPESQPEPEVQPEAEPEIQQEPVQQEQVQPQQVQQQVQSQPEQPQQP

VPVQTQEQPQQPQQPQQPQQPQQPQAQQPQQPQQPQQPQQPQQQPQQQGQQPPHFEKPAQ

GYDYYPQFQQTQQYQQAAGSVPGQYAYPSFDYSAAYGQLGQAGLGSVASPGYYPAAVNGA

AKPAAPAPASAEIGQSPLVQPNNMGQQSMQSGQAQVPGAAPFGYPNYYNYFYNTPFYGNG

GMAASTTSYGAQQQPQSQQAAGENAPASGEPETNGVPAQAQANNQYYAQYYGQPNQFGSR

GGYPYSGYPASQPYPQSAGQEQPEGAQPSAPQGAPVPGVPSYPQQMPQYGAYQQFPQYGS

YQDSNQYRGWY

>sp|C4Y206|CHO2_CLAL4 Phosphatidylethanolamine N-methyltransferase OS=Clavispora lusitaniae (strain ATCC 42720) OX=306902 GN=CHO2 PE=3 SV=1

MSDQRKGITFDGETFTFPETNDMVKTLFDPTVHKSACELIIVTLLVMNSLVFYSVSNNQS

RINIFVGMYIFWRLCYNFGIGFLLNQQSNRFRLVKISEKMRLFDKGNKSFWARCVQTEVQ

SQMGPSYSISAHPVAFNTWLIFRKVVDLILMEDFITFMCVVVACAIDSDYQFLHGQPVWL

TTLRLVIGSILIVFNFWVKVNAHNTIKDYAWYWGDFFFRQINNDDLIFDGVFEMFPHPMY

SAGYVGYYGFALIAKSYTVLIVAVFGHFLQMVFLHLVENPHIDKIYGPPPNETSLKKLVK

LKDLSFFDNVAPLVGLVNFNILRASDIINLLNCLTYAVAIPTVSSLATYNIEAMGKVLFC

IAILIKGFESFVINGVLLLQSNYKTISEWYLANNLPVENSLNNFAVLYNSLINLTYSSFV

GMNVFKLLTKLKYQDLIITGHIYLRIFLGLLLIITQVMINTSIVDSIGYFGWFYGDFFIP

KASVLPQRAHLSKGGVYRYLNNPEQIFGVCGVMGVTMIVPTYDNFMICLLWMLNNFFRIN

FVERSHMIKIYGEREVSQDSGVMKTVKKHLLPESIQKKFEVSPTNEPKKRTNSVFIESFD

SFIKELRSKNTSPVVSKERLAEMSQNEFFSGSDYCLEIEGLEDSSFVPHSFIGEPIEVKF

RAPAKHSPKDWVGLYKVAHTSFSRYRTLVSSNNRWDWTGPEEQGTIVFSKEKLFCEEGLY

EFRYHLDGKHDVAFISAPFEIKLRHIEVPLESAEADELANQLRKYIFDHVVSGVDDNETP

IFVGISQTQDIVATYEHIAMLITKSTGVKVGKRFLIYNDNETGNKFTVGDLASRLINIKK

VMHELEGDEYLRIKKLE

>tr|C4Y236|C4Y236_CLAL4 4-hydroxybenzoate polyprenyltransferase, mitochondrial OS=Clavispora lusitaniae (strain ATCC 42720) OX=306902 GN=COQ2 PE=3 SV=1

MLHLSLGPARFLASRSAFLSPARNSLRLALSGAFSSGSRAFLHDQSGSSPQKSPKTVSAT

VPPVSSPVSTPTKPVFSAEELEAARLARLAGLGWLSKLPEKWIPYFELMRLEKPVGTLLL

LIPSFWGITMASYGITASFPVFASAIGLFSIGALVMRGAGCTINDIWDRNLDNQVARTME

RPITSGRVSVPQAVTWMVAQCLVGLAILLSLPFECFYLGALSLPFVAAYPLFKRFTHYPQ

VVLSICFSWGCLLGFPAVGAALNLWVAGPLFLSNFLWCMIYDTIYAHQDKNYDIKAGIKS

TALAWGDKTKPIMYAMGSLQVASFLTAGVMNSMGPFFYLTACWGFSRLFQQIKKVNLDDP

KSCWAAFTGNIRTGEILWLGMLIDYLLKLFGFL

>tr|C4Y288|C4Y288_CLAL4 Lon protease homolog 2, peroxisomal OS=Clavispora lusitaniae (strain ATCC 42720) OX=306902 GN=CLUG_02651 PE=3 SV=1

MPKFQSSTVIKDDSVTQQVVLPMYTLDSPLVLLPGLVYSVTFSRFKAAALLSRFRRQVET

IPVAKALLAEFDFDSAVGDATGVSEEALDGIKQFRGVENKVKSGSPSLSQKSESLSGFDS

LVVCISPNLDKISNPQKSLDALQSGIFTVSRVIGIVDDTTTVKVTFQALRRASKVTGAET

VRANEVAVSVVWNEALVEASKHYEEIWSVSKSLFSSIDRFVDEYRQVLNSQDKKQDLLTL

NPLANALFLQLAGSKDFAKAYSSLKKIVRSMTLADVRDPQTFSRVVDLLVAIVPFPNHEK

LKVLSALGLEKRVEIVKSMLRKMNEVFQTIRENNSMVNHWFYEEASNIQRANVVANQLKS

IRVVLEGLSKNSYEKQALVKPKNGLTGSRRRGNDGKGVNGEDDEDDDDMRAISEFIKQRL

PEITSISDDSKRLIVKDYKRIKSSTPGNADFHVIRNYLEIVSDLPWDKFVSKFQSNKEID

VSVAKQQLDEDHYGLENVKTRLLQYLVVLKLLASNAEDEYKQSKIEEAEKEKKMQEEKER

LKFTRESSSGETFIIPNDEARNSKEANSTKEASASQTKSLLASKNNKSPIIMLAGPPGVG

KTSLAKSVARVLGRKFQRISLGGVKDESEIRGHRRTYVGAMPGTIVQALRKASCMNPVIL

LDEIDKVVGGGSNMTKFNGDPAAALLEVLDPEQNTQFIDHYLGFPIDLSQVIFICTANEP

YNLSRPLLDRLEMIEIGAYDYGEKLIIGSKYLLPRQIKRNGFPDGATVSIDENVMKQVII

DYTREAGVRNFERKLGTICRFKAVEYSESLANSDVHYDPKVEIYDLAKYLGVPHSNSNSA

IIDVPILSSKYGVVNGLSYNADGSGSVLIFECVGIAGEKNGPTLKMTGRLGDVLMESAKI

GLTFIKSLLYRRLFNTADVESLIDRLGHLEIHLHVPSGAIQKDGPSAGITMALAFLSLLM

EKAVPSDIAMTGEITLRGSVLPIGGLKEKFLGAHLTGHVNRIIVPRDNRRDVLEEYVQKI

NDTSKLNALLKDDSQANFKNTAPEDYFAEKYGVQILYAKEFWDVVKHVWGEDLLMKTEET

RLSEYHL

>sp|C4Y2C6|RSSA_CLAL4 Small ribosomal subunit protein uS2 OS=Clavispora lusitaniae (strain ATCC 42720) OX=306902 GN=RPS0 PE=3 SV=1

MSLPESFALTAEDAKLLLAANVHLGSKNVQVHNEPYVYKTRPDGVNVINIAKTWEKIVLA

ARIIAAIPNASDVVVCSSRTFGQRAVLKFASHTGATPIAGRFTPGNFTNYITRSFKEPRL

VIVTDPRTDAQAIKESSYVNIPVIALSDVDSPSEYVDVAIPCNNKGKHSIGLIWWLLARE

VLRLRGIIPDREAEWSVMPDLYFYRDPEEIEQNAAEEARAGATEETEEVVAEAETEWNTE

TNVEDWADSGVTAAGEEAAASQW

>tr|C4Y2C9|C4Y2C9_CLAL4 Nicotinamide-nucleotide adenylyltransferase OS=Clavispora lusitaniae (strain ATCC 42720) OX=306902 GN=CLUG_02692 PE=3 SV=1

MDPTSDPNFLPPSRNHDFEPPAPSSQIPTNMPIQPLVLADLGSEVDAPAPHPSTRLHKEK

EPSAYHSKIPRRYNELMNSSSDEDQNGKHEEDHKNHQDGEHKEQPLGEQADGHKTSQSQP

MKAHVAQAAQTHKPILPPTIKVRSTQIADLEEVPHGIQRQAKSMSAYSFPTHRLATSLRD

DSKYPLVVVACGSFSPITYLHLRMFEMALDAISEQTRFEVIGGYYSPVSDNYKKQGLAPA

HHRVRMCELACERTSSWLMVDAWESLQPRYTRTALVLDHFNEEVNIKRGGIRTQSGEQRG

VKIMLLAGGDLIESMGEPDVWADQDLHHILGKYGCLIVERTGSDVRSFLLSHDIMYEHRR

NVLVIKQLIYNDISSTKIRLFIRRGMSVQYLLPNSVIRYIQEHRLYINDTEPVKQVMNDK

GD

>tr|C4Y2E7|C4Y2E7_CLAL4 Acireductone dioxygenase OS=Clavispora lusitaniae (strain ATCC 42720) OX=306902 GN=ADI1 PE=3 SV=1

MVSFFYHDNLDTPENFTDEHDSGIPVPPEKLAELGVIYRYIDNMPELEALAKERDYKNRD

QVKLNLESFGGDKEAYDAKMRQFYKEHYHEDEEIRYIVDGEGYFDVRDRGDRWIRAKLTK

NDLLILPAGIYHRFTLSSRLKNVTALRLFKDEPKWEAINRDEGRLTEARQQYARSIAV

>tr|C4Y2F5|C4Y2F5_CLAL4 CDP-diacylglycerol--serine O-phosphatidyltransferase OS=Clavispora lusitaniae (strain ATCC 42720) OX=306902 GN=CLUG_02718 PE=3 SV=1

MSYSSGFKKNESAIASDTEGDVEISDRPNPPLRRSSSLFSLSSNQEPLPRPDPEDYKNFL

DDNRHFSLIRNLHMADFITLLNGFSGFYSIISCLRFALTGQLHYVQRAHFFICLGLFFDF

FDGRVARLRNKASLMGQELDSLADLISFSVSPAIIAFAIGFRSTVDTLILAYWVLCGLTR

LARFNISTNNIPKDSTGKSQYFEGLPVPSNLIWVVTMAILVYRGLVFDKLPGGMIFAGTF

AEFHAVSLLFIVQGSAEISKSLRIPKP

>tr|C4Y2H2|C4Y2H2_CLAL4 Tubulin alpha chain OS=Clavispora lusitaniae (strain ATCC 42720) OX=306902 GN=CLUG_02735 PE=3 SV=1

MADQCDGLQGFLFTHSLGGGTGSGLGSLLLEQLSIDYGKKSKLEFAVYPAPQVSTSVVEP

YNTVLTTHTTLEHADCTFMVDNEAIYDMCRRNLGIARPNFDSLNSLIAQVVSSVTASLRF

DGSLNVDLNEFQTNLVPYPRIHFPLVSYAPVFSKVRANHESNSVSEITASCFEPGNQLVK

CDPRLGKYMATCLLYRGDVVTRDVQDSVAQIKAKKTVQLVDWCPTGFKIGICYQPPTAIQ

GSELASAKRAVCMLSNTTAIAEAWKRIDKKFDLMYSKRAFVHWYVGEGMEEGEFTEARED

LAALERDYEEVGTDSFPEEEEEY

>tr|C4Y2K2|C4Y2K2_CLAL4 Actin cytoskeleton-regulatory complex protein PAN1 OS=Clavispora lusitaniae (strain ATCC 42720) OX=306902 GN=CLUG_02765 PE=3 SV=1

MYNQYQQGGYSQNYGQYQQGYPLQQQSTGFYGAAPQQPGLFQGQQTGFPQQFQGQFAQQQ

AVPSQQQGFQPQQTGYAPLQAQKTGFSAALADVQENSDIKIPNMRLSFITASDQTKFEHL

FRTAVAKGENAVSGDTARDILLRSGLAPVLLAEIWALADTNKSGSLLFPEFALALHLCNM

ALRGDQLPHQLPEKWANEVQSFVDAINFSVPEDPAAILANTPFSSFAQDKSDWMTQGPAP

PTSFVPQKTGGGLVPLQPQQTAGLVPAQGFQPQSTGYQPLQPQTTGFQPLQPQTTGFQQL

QPQTTGYQSQGGQSGFQGIKPQTTGYQAQGMQSQGIQPQSTGFQPQAIQPQTTGYQAQGI

QPQSTGFQPQSTGFQPQSTGFQPQNTGYQNTGIQSLQPQSTGFLQAQPTGKPGQWGFVSM

PTGGIPGLNAMQQHFLPSAELPSHNLQNAMGGSLKSNVTWAITKQEKQIYDGIFAAWDTG

KQGYIQGDVAISIFGKSGLSRPDLESIWNLCDSSNRGKLNKDEFAVAMHLVYRRLNGYDI

PLRLPPELVPPSAKYLQDSVDTLKNSLKGGSAKKAAPAAKPTTSASRFKNDDDNVGYVSS

SRHKSRKPSESQGSVPNSKSRDLSVQELKKLIHEKRILLDALDVEDQHNSLARKQEDENN

YREIEHLKHQVIDVQKELNKYALGANEVEKKRLLEKLDHFTKDKVPSLMSQIYQVTADIT

QAKIELTKAKLKKQFPDWSPESGDEGIVGTGINGEVTEADIRKHKSKQLLRQRMAALTGK

PIPGGSNKDAEAQLQQEIEAAKRESESQQGMIKDIETSIKELEDGAAVHLQTSVKSEATS

SKWEEGNDVSSMMRAFIDELNAFAKAEANQPKSDQSMPVANSIQKPTKPSTSSSSSLAST

PTPKAKPAYKTPEERSAYIKAQAEKRMNERLAKLGISRNKNHEREEAAPEKAPEPKPEPK

VEQRAERQEAKQEPKPQPREVPEQPKPQPQVQQNVEQPKPQVASQIVEPTPVHAERNVPE

ESDDEEDAEYAALLKQKQEMEARKKEKELRKKQEREARLAKLKKEMAALEDDSDEEPAQV

QAVSYKPSSVHNEKKPNQSEFQPEAKTEPQAPVASKPAEQAPEQTKPSAETASASSETGP

HKSNPFAKFATGQPSTPGANSNPFFKPTNKTESVDQNKLAAQRASQRGLGSEDWSDEEEN

SSDDEEPNRAGAAKLASLLFGGMPQPLSRATTGNAGFQAQPSQPQAPEAPQASQPEQAPQ

PPQEAPAVPVPQGQPPVPETQPPVPQGQPPVPETQPPVPETQPPVPETQPPVPETQPPVP

ETQPPVPQAQPSVPVAGSAESAPPLSASSHISDDSSFDNTTDSSDDFATPSPQAFPPPPT

DGIPPPPPPQGMPPPPPPPVPMPPSDGVPPPPSAIPPPPMGIPPPPMGVPPPPSAVPPPP

QAPPPSFGSAPPAPGGPPPPPSFLSAPAPPSGGPSAVPNLGALLGQIQGGKALKKVDDSE

KHVAENSLAGRVL

>tr|C4Y2U6|C4Y2U6_CLAL4 ATP-dependent DNA helicase CHL1 OS=Clavispora lusitaniae (strain ATCC 42720) OX=306902 GN=CLUG_02859 PE=3 SV=1

MRKNERVTLWLMTRDYSHPYEPYKIQNELMDAIYETVNNGFKVGLFESPTGTGKTLSIIC

STMTWLRDFKQENDTNIAYKGNASDSSNESSDDEPDWVKAAHRKSILSRTKGRAIDYEKH

LQGLKDQVLDFTELEGRNNLKKKKESIEYDTFVPNDYHSDSEGNSIAVKNSRLSAENTAI

MKRLSGNDVESLYPPECPHTILFSSRTHSQLYQFAHQLQLPSFHSSLEEITEYTKFVALG

SRKQLCINDKVRSLGNLNSINDACVDLQKKDQPCDFFPKITKSGVSEKTKSFIDNCFVKV

NDIEDVASLGKKLEICPYYTVRNATDYAEIIALPYQLLLSKSARESLDLKIENSIVVIDE

AHNLIDTISSMNSVRLSLSELTMCINGLKLYLSKFARRLNSGNRIYLVKLIKLCQVVQKF

MTKTLSYKSGDKVDPLEIFQGNTGDLINVNKLNKYLSKSKIAYKIESYLEHVDDQISKQS

SSNPILFKVVQFLECLTNTSNEGEIIWDMQEENVTLQYILLDPSIIFEDVLSQAKCVLLC

GGTMEPMDDFKSYLFPNIPSNLVKTFTCDHLIPKENLKVFAVKATNNLDFEFSFQKRSQP

SMMRELGLLIARLCQSIPSGVVIFFPSYKYLSHVFSLWSSSGILERISSSKTVFQEPKDS

QEVESTLQEYSNAVKSSFKGAALFAVVGGKMAEGINFADDLARGVIVVGLPYPNAAATDL

VAKQNHIVRTLIAKGKSRSEAANASRDFYENICMRAVNQSIGRSIRHANDYSTIFLIDQR

YSRKNIRQKLSKWVRNQLSDSDQNIDEIIHSTEKFFEYHRYTVNK

>tr|C4Y2Z8|C4Y2Z8_CLAL4 Phosphotransferase OS=Clavispora lusitaniae (strain ATCC 42720) OX=306902 GN=CLUG_02911 PE=3 SV=1

MCRSFCRCVFSHFSVASFSDTVLHRDYATWRAQIQHVASAPPPVNTYTGHSPLSASMFLD

TQNFPQGSDMAENERVGIPDNAARDIGDIIVEIDTTDSTASDISLASSPSSSLSDPEPDS

VLATALTAFLGPVESGDVGGQARDLALSLDEALSKSDLSMIPCYLVDPSGEEHGRFLAID

LGGSTLRVAVIEIGQSEEKPSEQEQEQPEPEQTEPEQLQPEQEQEQPQPEPEQRNGSEPN

GSEDHCESSRRSRVRIVHSRSWTVEGRHKHVDEAFFDWLAANACSVLAASRQESQPASFL

QPLATGITWSFALEAAAPHRAKIAHMAKGYTLDARVAGRELGALVEAAMARRGVPVRVDA

ILNDSLAVYAAGRFFDPKTEAAMVLGTGFNVCCAVDVDDNLPAQKRLPGQKKVLYNAEAS

LFGCGLASALATCYDELVDARFGAELAFAPHLELDPESGAMFQPCELLAAGRYVPELVRH

VLVAMAQRREVFGAQKCIEPLQVAYSGMTGELVCMACESPGDDVVARALESHFQWAPRSV

SRADVAAFRLVSRAVVRRAAYVIASAVAGFVLFLASHNGRFKHHTITIGYVGSVMEHFHT

MRTAITELVNACEDVSALGVRVEFELVPESSLVGAAISAAYHADRGHEP

>sp|C4Y2Z9|SEY1_CLAL4 Protein SEY1 OS=Clavispora lusitaniae (strain ATCC 42720) OX=306902 GN=SEY1 PE=3 SV=1

MSNVPSPTVTLEGDSPDAAHEAVSSSSSSFVPVEIQDAIQIIDEHKQFNKQILDYISSRS

RQPGDYRIISVFGSQSTGKSTLLNHLFSTNFDVMDEVNRQQTTKGIWMAVSPGVSNSLPA

NAHVPPENILVMDVEGTDGRERGEDQDFERKAALFALSTSEVLIVNMWESQVGLYQGANM

GLLKTVFEVNLSLFGKAKLQNNDHKVLLLFVIRDHLGVTPMESLAATITQDLLRIWEGLN

KPADVAHLAFDDFFDLAFHTLSHKVLQNEKFLDDVRSLGNKFLDTSSESFLFKPNYHHDI

PIEGWTMYAENCWDQIDHNKDLDLPTQQILVAKFKCDEVAAQCFEEFAKVSHELKNVAVS

ATQSTEPIDYKDTGLGFQDMKQSVLEDYDLGASKYNKSVYQQKRATLAEKIDSTLQDVFA

IYAKHLVTTSLKAVSAGLSRKTRSGTFVEAMEKLKQSSAHDFSQALALISLDGALDTRPF

EKEYLAELEQLVSKQQIVELNSILSKALKKLNNGLSTCFVEELANPSELTWDHILEKFRG

LSKSALQKYETEEGDYDFRLGTLPSMNKRALKTFDFKSWELLDNLIHKYISKDNLLNILK

DRFDDKFRYDENGVPRLYQNTKELEGSFSESKTHALKAFPILTVARLSDGTEVIPKYDVR

DKKLKRQYETVREEKEAEEEDEDEWDSEDDENQRAFAELLSESEKAEVMAKFKREMDAKF

VETKRSIMQHVTQIPYYIYIVILVLGWNEFMAILRNPFFFTLLIMLAGATYVMYSMNLLG

PASIVVQRMANEALGLAKEKLREFVVDDHMQHGHNMKKMTTNDIELDDLSEEST

>tr|C4Y319|C4Y319_CLAL4 Serine/threonine-protein kinase RAD53 OS=Clavispora lusitaniae (strain ATCC 42720) OX=306902 GN=CLUG_02932 PE=3 SV=1

MEPTQPTQTQPTQKSPSVQEEEKNSDHVCRLICTTGQYQSFDLNKNQPTRSTASATKKTW

LFGRNADCDFVLSTCTRLSNKHFKLWLNLADKTLWIQDTSTNGTHLNGSRLVKGSNYIVN

QGDEIAVGVGVPKDVIRFVVLFSDLYNPSNSEKSSVIKDEGIYKDFIIKNETIGQGAFAT

VKKVIERSTGDSYAVKIINRRRAIHAGGKGAMLGVNRELEILRKLDHPNIVKLKSFYEDI

ENYYLVMELVPGGDLMDFVAANGAIGEDATQVITRQVLDGIAYVHKLGISHRDLKPDNIL

IMQDDPILVKITDFGLAKISDNATFMKTFCGTLAYVAPEVITGKYDHSQDSPVNYSNLVD

IWSLGCLVYVLLTSHLPFNGKTQTQMFQKIKSGEYHESPLNSYKISPEGRDFLNRCLQVD

PRNRIVAEDAIKHPWLADVEPYPSQESQLSQKVVSLSQSTSQQSRKIENGIQVNSSMSKL

DDDIMMRALDSDRNKKQKKVTNAEFKVPKRVIPLPQSQPLQPNSQNYPFVSPKKVDKPKS

SSQKKRSIDELSNSPQKKFKADPDIKRLSISRAEEIAPSDTCIILEPAPESIMKRPIYIR

QGVNPYAIGRNETCDTFINDDRMSKIHCLINKKRHPVLEASIYESPAHCLEDIWLLDFST

NSCFVNGVRLGKGRKVQIFNRDRVDFFVDDTCNQSMSFIVRINDTTGLFNGGEKVQDHKF

VNVVKFDAADAKLRPPIVAEPPSVKSHVNGSLSQQYSDGILAGNGGNFNSSLRIQRKVYD

HAKSSSQSSKRASLQSGQERPKNSWV

>tr|C4Y336|C4Y336_CLAL4 Histone acetyltransferase OS=Clavispora lusitaniae (strain ATCC 42720) OX=306902 GN=CLUG_02949 PE=3 SV=1

MTPVPSAKTPIKIEEGASVAEFGPEDIVSGCKVYVSKENSTRLAEILQIHYKKGHKRFYV

HYQDYNKRLDEWITPERVDFSRPMFLPEEKSEEKKQGKSSKTSKTSKTSKTSKTSKSSKA

SKDSVPPPSSEGTPLENDEMDLDNLNVQGLKSENEKVSREDEIEKLRTSGSMIQNHSEVA

RVRNLSSIILGEHIIEPWYFSPYPIELTEEDEIYICDFTLSYFGSRKQFERHRAKCSMKH

PPGNEIYRDEKVSFWEIDGRKQRTWCRNLCLLSKLFLDHKTLYYDVDPFLFYIMTKKSKH

GHHVVGYFSKEKESADGYNVACILTLPCYQKMGFGKLLIQFSYMLSKVENKVGSPEKPLS

DLGLLSYRAYWTDVLVKLLVERCNPHLYKKNNIHHEDRDSLSPPPRTGTSPPEITIEEIS

SITCMTTTDILHTLSTLQIIKYHKGQHIIVITDQIMALYEKLTKKVREKKKHELDPAKLQ

WTPPSFTANQLRFGW

>tr|C4Y365|C4Y365_CLAL4 Serine hydroxymethyltransferase OS=Clavispora lusitaniae (strain ATCC 42720) OX=306902 GN=CLUG_02978 PE=3 SV=1

MHQVRHEHVTHISGPSASQSGAKKTMAPTPLSIDIFTKNTNMLSRSVRALKHVPSKPFAR

SYALSAKAQALISKPVQEVDPEMASILQQEKDRQRNSITLIPSENFTSKAVMDLLGSEMQ

NKYSEGYPGERYYGGNEIIDKAESLCRQRALEAFDLSPEEWGVNVQPLSGAPANLYAYSA

VLEVGDRIMGLDLPHGGHLSHGYQTPSAKISYISKYFQTMPYRLDESTGLIDYDTLEKNA

VLFRPKVIVAGASAYSRVIDYKRMRAIADKVGAYLLSDMAHISGLVSAGVTPSPFPYSDI

VTTTTHKSLRGPRGAMIFFRKGIRKVTKKGKEIPYDLERKINFSVFPAHQGGPHNHTISA

LAVALKQCSYPEYKQYQQEVVDNAKAFADALKGKGFDLVSDGTDTHLILVDLRSKKIDGA

RVEAVLERANIAANKNTVPGDKSALFPSGLRVGTPAMTTRGFGPEEFAKVAEYFQRAVEI

AISLKEKEEGAVAKELLASFKKLADESAEVKALDEEVKAWAAQYPVPGDL

>tr|C4Y390|C4Y390_CLAL4 Methionine aminopeptidase OS=Clavispora lusitaniae (strain ATCC 42720) OX=306902 GN=CLUG_03003 PE=3 SV=1

MASVCCSPQCGKTTDSGLKCPVCLKNGISAVFCNQICFRSGWAIHKAVHPAEDNKPFDPF

PNFQYSGDLRASYPLSPRRTVPANIAKPDYAQNGKPVSELKNDRTGKITILTESQIKKMR

RVARISREILDKTASYIKPGITTDELDAILHKQCMAHNAYPSPLNYYNFPKSLCTSVNEV

ICHGIPDQTVLQDGDIINLDVTIYLSGYHSDLNETYYVGDKAKCDPDTVRLVETTRECLD

MAIDMVKPGLVFRDLGSVIEGHASKNGCSVVRTYCGHGINSLFHCQPNIPHYAKNKAIGV

AKPGMVFTIEPMLCLGTYKDITWPDNWTAATQDGKKSAQFEHMLLVTETGCEVLTARLDN

SPGGAVKRIE

>tr|C4Y3B6|C4Y3B6_CLAL4 DNA replication licensing factor MCM4 OS=Clavispora lusitaniae (strain ATCC 42720) OX=306902 GN=CLUG_03029 PE=3 SV=1

MSSSPVRSSRDSASQSQSQPVPSSPLFFASSSDPPSESSKREISSPLHYTSSSRAAGSSR

GHLGSSQPDLPSEDRITRRPARSDLNSDVLATPVKRRMFATSSDPVEGDADAEPVRVIWG

TNVSIQDCSNAFRDFLMSFKMKHRRIMDGQPVEPEDSQLYYVGQLTAMKDLGTTNLNLDA

ANLLAYPPTKKLYYQLINYPQEVIPIMDQTVKDCLVSLFSESAAVVAVDAIETNIYTIRP

YNINAVKKGMRELNPNDIDKLVSVKGLVLRASAIVPDMKVAFFKCSACDHTLAVEIDRGV

ISEPTKCPRAVCGQVNSMALVHNRSSFADKQVVKLQETPDMVPAGQTPHSVNLCVYDELV

DTCRAGDRVEVCGIFRSLPVRVNARQRAVKSLYKTYLDVVHVQKIDARRLGVDSSTAPPQ

DAHEVEQRRVLSPEDIERVREISQRDDLYEVLARSLAPSVYEMDDVKKGILLQLFGGTNK

QFRNGGRYRGDINVLLCGDPSTSKSQILQYVHKIAPRGIYTSGKGSSAVGLTAYITRDID

TKQLVLESGALVLSDGGVCCIDEFDKMSDATRSVLHEVMEQQTISIAKAGIITTLNARTA

ILASANPINSRYDPRLPVTANIDLPPPLLSRFDLVYLILDKVDESIDRHLARHITDMYLE

DEPESVSAHAVLPVETLSIYIQYAKENVHPQITAESKAELVRAYVDMRRLGDDARAADKR

ITATTRQLESMIRLSEAHAKMRLSPRVELVDVKEAVRLIKSAIKDYATDPITGRIDMDMV

QTGTTMAQRRMQEDLAHEVLALLDSPIRFSDLVTRINEKSSVRVENADLNECLRRLQQEG

KIVESGDTNRRTIRKLAVI

>sp|C4Y3C1|VPS10_CLAL4 Vacuolar protein sorting/targeting protein 10 OS=Clavispora lusitaniae (strain ATCC 42720) OX=306902 GN=VPS10 PE=3 SV=1

MNLRASCIAAFLCLLVQLCAADFSPEVTHRIVKGIGWDISYFEDSSNVIHVGEKMVEMSY

DDGVTWTEIETIGKSSDILVKFDPINVNRAFVFSRIGDTHFVTDDKGKTWRSFKVQPKKS

LKDRELGMHHVFNFKDDSVLIKLEYCKQYPEADDCDVSYFYSSNSLKSDPQLLPIDAKVC

VVNTLLDEPTILCVKDTRNSFGHVVKSELVSSKDLFKTSESIKHPGFDSGRIIDVRLESS

FLVVVTQKDRFNELSSISISVSKDGKTFDTADLAFQMAYGAIRFLPSDPQSLFLTVAAST

SKDTRPQGTLYSSDSSGLHFKKLLDNVDLGYSARVEYVHGVWLAKTFEEDPIVEPNDDYF

GSPIAKTSSHISIDNGLTWNKLEILNDDSCKLKDGCSLNLLRFDAIEDKNKYVTGPTPNI

LMGFGITGNTDNFFKAKTYISRDGGLTWTNVFSGPGIHAFGDQGNVIMYVPFSGRNHGPA

TEVRYSLNQGRSWGTYKLKEPCFPQELITTTDGTNTKFVLSGIVEHQQMSEVLYAFDFSK

AFDGNKCKDSDMEKVYARVSPKDGQPNCIYGFKESFMRRKADARCFIDRTFEDVKVDVEQ

CPCTQQDYECSPYFKLSEKGVCVPDPKKIAEKCKSESKDTLKLPDMQIVAENKCEQKSDS

SFVTETEFKCKDFTGENVPSSITIGQSEFDGFLSQYSYVATGPDLSDNLIVKTDTGLIYA

SNNGGTSFVRVPFKEKIRGFYVGPSLGRVIMITDGDVFYASNNGANTFYKYKVPTSASNG

DVTISFHPTDESRFIWLSGNCAVGSSTCVAYHTEDFGRSFEKLIDNAMACDYVSPVLDIT

STELIYCTVVDGNKKKLASSTNYFKEAEIEYVLDDIVAYAIKSNFVVVATLDEAHTMLKA

KVTADGFVFADVDFPSDFKIEAQTAFTILESSPHSIFMHVTTDSTSGHEKGAILKSNSNG

TYYVLSLSDVNRNEVGYVDYDRLGLLEGVLIANTATDSKEGKNKKTQISFNDGSQWNYIA

PPAVDSDGKEYACKGQPLSKCSLHLHGFTERPDYRDTFSSGSAVGLLIGMGNVGEYLKPV

TDDQTAAFMSADGGLTWKEIKKGVYHWDFGDQGTILLLVDAAKDTDEFIFSKDEGATWET

MKFASSPVKVLDLATVPSDTSLKFVIFAGSHQSQSSTQLFAVDFSQFFPRQCQVDLENPE

SDDFEYWTPMHPESTERCLFGHEAMYLRRAAGHDDCFIGSTPLDQGYKMVRNCTCTRNDY

ECDYNYFRDNDGTCKLVKGMNAANRMAEMCRKPGVFEYFEPTGYRKIPLSTCVGGKQFDA

LKPRACPGHEKEFNEAHGRDVGGSKIFILIFIPLLVFFSAVFFVYDRGIRRNGGFQKLGQ

IRLDDGDDDFNPIEENIVDVVVNKTVRGAIVVAAGTFAVFKTIRKIDRAMFDKVTSLLFG

RRPGQRNYVRVPDDEDELFGTFDENYEDELNEAADVDFNVDEEPEEFTELTAEPANVDER

LFDIDDQSEDDAVSAEHPEPESTHE

>sp|C4Y3D4|SEC11_CLAL4 Signal peptidase complex catalytic subunit SEC11 OS=Clavispora lusitaniae (strain ATCC 42720) OX=306902 GN=SEC11 PE=3 SV=1

MNLRQQLTQLLSIAYVFTSAFVAWKALSIVANSHSPIVVVLSGSMEPAFQRGDILFLWNR

DSQAKVGDVVVYEIKGKSIPIVHRVLREHHGKDKQFLLTKGDNNALDDLSLYARKQNYLN

QKTDLVGTVKAYLPKVGYVTILLTENMYFRYALLGFMGLSALLSGE

>sp|C4Y3K5|GATF_CLAL4 Glutamyl-tRNA(Gln) amidotransferase subunit F, mitochondrial OS=Clavispora lusitaniae (strain ATCC 42720) OX=306902 GN=GTF1 PE=3 SV=1

MFLRQFSTSPALLKGKVLPELKNAQEISQFLRKSTWNVHDLIPSKEHITNEVDSRVVRKM

LRLSGLDENLPEPELNRWAEMLNTHVAFINHVSDLHSSTKGEIGSSVFRLLASDHKPESP

LTLKELLRQVDEISDHVSDQRGERGFDTSELRTRINRAKSTAEKE

>tr|C4Y3Q9|C4Y3Q9_CLAL4 Chromatin-remodeling ATPase INO80 OS=Clavispora lusitaniae (strain ATCC 42720) OX=306902 GN=CLUG_03172 PE=3 SV=1

MNLQSILSENTPPPAAYSHNNVPHSNPSSLGPTKGTASAPYLANGMSEDPALFSHVDSSY

LKDFEDRVTVLTRLDHAMARAQNLSIIAAERERLTSAISRNESFASNYMDRMVMEDVLAD

PNASRSLRTINWGSTRTHPDESELEAKRLSLVENAAALLSADLKDDGDLVDDATGSKRAA

SFEANDTKRRKADDDGIDKSRSRDAKENGRLRIKMSLKEAENAAESSPAADAKSSPTKTK

ELKSVMKQYDNTFVAIWKDMSRKDGPKVSRLMQQSNQAKLINLKKTAILASREAKRWQLK

NNKNQKDLTSKARRAMREMFNFWKRNERLEREARKKHEKEIMDKAKKEEEDREAKRQSRK

LNFLITQTELYSHFISKKIKTSEIEGSDSDPNLKAANGSSHLDKYHGVEGAATDFNSIDF

DDDDEEALHRAAAANAQNALNSAKSKAEAFDDDPFRNPDTNGEEMNFQNPTLIGDMSVTQ

PKMLKCTLKEYQVKGLNWLANLYEQGINGILADEMGLGKTVQSISVLAYLAETHNIWGPY

LVVTPASTLHNWQQEISKFVPEFKVLPYWGNAKDRKVLRKFWDRKSVRYGKDAPFHVLVT

SYQLVVADAPYFQKMKWQYMILDEAQAIKSSQSSRWKSLLSFSCRNRLLLTGTPIQNSMQ

ELWALLHFIMPSLFDSHDEFSDWFSKDIESHAQSNTQLNEQQLKRLHVILKPFMLRRVKK

NVQSELGDKVEIDIYCDLTNRQKKYYQMLKSQISIMDLLSNSSNDESNQSLMNLVMQFRK

VCNHPDLFERADVESSFTFGTFAETSSFLRESELELYYSLKNSIHFDLPRQIYQELLVPS

EKSNVGSKNKIYEMFSIYNPENITHDDTLDNFGWLRFVDASPAELKQYTKKNLLERAISL

REYSSINYDGINRFKYFYDDDEQFVPQNKRLLISELNNDNCVKSNSVVFNQLFSIKDKVY

VDMYMNVLQAAAHPKVSSAPINLRCSNYMCSQDYDTVLFDQEIRSSLVPLSLNTELDLMK

DKVPISEYPKSDMLPAPINNFIDYSNIRMPSMTRFITESGKLSKLDKLLDELKQNDHRVL

VYFQMTKMMDLMEEFLTFRQHNYVRLDGSSKLEDRRDLVHDWQTKPEIFVFLLSTRAGGL

GINLTAADTVIFYDSDWNPTIDSQAMDRAHRLGQTKQVTVYRLLTKGTIEERMRDRAKQK

EQVQQVVMEGKSSFGKKKEDTASKKKDVAFLLLGGDENGDSNSKDTSGETSTEASTPKEP

MSQALEAMYHEGEGEFSGNITPVP

>tr|C4Y3U6|C4Y3U6_CLAL4 mRNA-capping enzyme subunit alpha OS=Clavispora lusitaniae (strain ATCC 42720) OX=306902 GN=CLUG_02318 PE=3 SV=1

MLKLSERDMPVIPGTKIENGELQELRLMLADLLRRKNTQFPGSQPVSFERNHIEVLKKRE

YFVCEKSDGLRCLLFLINDPVKGEGVFLITRENEFYFIPNIHFPLSVNEENGKTYHHGTL

LDGELVLETKNVSEPYLRYCIFDALVINEKDITNRPLPKRLGYITENVMKPFDSYKSKHP

EIVNSPDFPFKVSFKMMKSSYRANVVLSMQDQLFHESDGLIFTCAETPYVFGTDATLLKW

KPAHENTIDFKMYMEFKQFQDPDMDPRDPDSTYLDYDSLPERINLNVWKGGKEYEQFAQM

DLSEEDWEQLKGLNEPLQGRIVECRKKISNPPYWEMLRFRNDKSNGNHISVVEKILHSIE

DGVTEKELIDACPEIEKAWKAREYERHQRAKNTHHDTHLPAQMNTQPRVPPSTQPQNNVH

KRPLSSENTEQTKKPRVAEEQMLKDMMPDYEEDSESD

>sp|C4Y3W1|ENOPH_CLAL4 Enolase-phosphatase E1 OS=Clavispora lusitaniae (strain ATCC 42720) OX=306902 GN=UTR4 PE=3 SV=1

MSIALLDIEGTVCPITFVKDCLFPYFSKQYPSYLRDVSFPIDKSDGGLADVLAGFPKEAV

ASIDQLKNHIDDLVARDVKDPVLKSFQGLVWKEGYAKGDLKAPVYEDAIAFINRSKSVYI

YSSGSVGAQKLLFSHVDVNGASVDLTPKLKGYFDITTAGFKQEKDSYLKIAADIGCDPAD

VIFYSDNVLEVKAALEAGMASKVVVRPGNAELSESDKKSYECISSFTAE

>tr|C4Y457|C4Y457_CLAL4 Adenine deaminase OS=Clavispora lusitaniae (strain ATCC 42720) OX=306902 GN=AAH1 PE=3 SV=1

MAQYQCTEHVKSFLTELPKCEHHLHLEGTLEPSLLFELAERNNVSLPNHFPPTVEACNER

YANFADLQDFLDHYYIGMSVLIKEDDFYDLAMEYFSRAHKDGCLHSEVFFDPQGHVERGI

SVDVVVQGFDRACKDANAKFGTTNKLIMCLLRHLPAASGIETIDSTAKYFENGTIHGLGL

DSAEKPFPPELFTECYGLIKSKFPQVGLTAHAGEEGDHTYVSNSLDLLNVSRIDHGVNSK

NDEQLMKRLAEGRIMLSMCPLSNVKLQVVKDVSELPIALFLENDVPFSINVR

>tr|C4Y477|C4Y477_CLAL4 3-hydroxyanthranilate 3,4-dioxygenase OS=Clavispora lusitaniae (strain ATCC 42720) OX=306902 GN=BNA1 PE=3 SV=1

MLAPPINIKKWIEENSHLLQPPVNNFCLHRGGFTVMIVGGPNERTDYHVNQTPEYFHQLK

GHMCLKVVDDGEFKDIIINEGDSFLLPGNVPHNPVRFADTIGLVVEQDRPKDVNDKIRWY

CRNCRDKFHEVEFYCYDLGTQVKDAIVAFDQDMDARTCKKCGTLNYSKPQSV

>tr|C4Y4A1|C4Y4A1_CLAL4 Succinate--CoA ligase [ADP-forming] subunit beta, mitochondrial OS=Clavispora lusitaniae (strain ATCC 42720) OX=306902 GN=CLUG_02473 PE=3 SV=1

MYLQKISVIGRQIVNCLISITNRQICQKRFLSLHEYRSAALLESYGVGVPKGFAATTPEG

AYEAAKSLGTNELVIKAQALTGGRGKGHFDNGFQGGVKLISSPEEAKDLAGKMLHHKLIT

KQTGAAGKEVTAVYIVERRDARTEAYLAILMDRSTQLPMIVASAQGGMDIEGVAAKDPDA

IKTFSVDLNEGVTDELATKVASVLGYTEEAIPEAAKAVQNLYKVFTEKDCTQVEINPLSE

TPDHKVLAMDAKLGFDDNASFRQEEVFSWRDPTQEDPQESEASKYGLNFIKLDGNIANIV

NGAGLAMATMDIIKLYGGEPANFLDCGGTATPETIEKAFELILSDKKVNGIFVNIFGGIV

RCDYVAEGLIAATKNFKLDIPVVVRLQGTNMEKAKELIENSGLKLYAFEDLDPAAEKIVQ

LAPKA

>tr|C4Y4B6|C4Y4B6_CLAL4 Ribosome biogenesis protein ERB1 OS=Clavispora lusitaniae (strain ATCC 42720) OX=306902 GN=ERB1 PE=3 SV=1

MAKKSKDVTSAPISVNSKKRKPQEEEDDLQVSGMLTIDGDDSSEEEDDEEKEQDEEEKED

DEAEEHGDDESTGSEEDEEEAELNRILREEEGDVEGESENDSEEESSEDSEGHSEPESLN

DRLSGIKLKTIASEDNEEVHTKYSDGRPRILKKEIEPVYDSDDSDAENFNTIGNIPISAY

EEMPHIGYDINGKRIMRPAKGSALDQLLEQIELPEGWTGLLDQNTGESLKLSTEELELIR

KIQNNENTDESINPYEPTIEWFTSKTEVMPLTAVPEPKRRFMPSKHEAQRVMKIVRAIRE

GKIIPPNKAKEQVEEELHNFDLWTNEDNTEAHIMNLRAPKLAPPSNEESYNPPEEYLLTE

EEKQQWLETAPADRETSFLPQKYSALRKVPGYGESVRERFERCLDLYLAPRVRHNKLNID

PESLIPQLPSPKDLRPFPIKCSTVYQGHNGRIRTLSIDPQGLWLATGGDDGTVRVWEVLT

GRQVFKLDVIDIEENPEDRIDALEWHPDPSSPILAVAAAERVMLVVPSIFGFEIENNARV

KTEAGWGYGTFGNKRKDTDINVNDDDDESVETKAPEQPKAEVAKWYAPSAEQTSYGVAAI

VECKKPVKKLSWHRKGDYFVTVAPESKNTAVLIHQLSKHLSQSPFRKAKGTIMDAKFHPF

KPQLFVSSQRAIRIYDLAQQTLVKKLMPGARMLSGIDIHPRGDHLLASSYDKRVLWHDLD

LSATPYKTLRYHEKAVRSIKFHKGKLPLFASASDDGTIHVFHGTVYDDFMTNPLLVPLKK

LTGHKVVSSIGVLDLAWHPKEAWLFSAGADGTARLWTT

>tr|C4Y4H0|C4Y4H0_CLAL4 Pyruvate carboxylase OS=Clavispora lusitaniae (strain ATCC 42720) OX=306902 GN=CLUG_02542 PE=4 SV=1

MSVSGPSDLGKINQMRSESTVLGPMNKILVANRGEIPIRIFRTAHELSMQTVAIYSHEDR

LSMHRLKADESYVIGKKGQYSPVQAYLQIDEIINIAKKHNVNMIHPGYGFLSENSTFARK

VEEAGIAWIGPSYKTIDSVGDKVSARNLALANNVPVVPGTDGPIDTTEEAVAFVQKYGYP

VIIKAAFGGGGRGMRVVREGDSIEDAFNRAKSEALTAFGNGTCFIERFLDKPKHIEVQLL

ADNYGNVIHLFERDCSVQRRHQKVVEIAPAKNLPRSVRDAILTDAVTLAKSANYRNAGTA

EFLVDEQNRHYFIEINPRIQVEHTITEEITGVDIVAAQIQIAAGASLQQLGLLQDKITTR

GFAIQCRITTEDPAKGFQPDTGKIEVYRSAGGNGVRLDGGNGFAGAVISPHYDSMLVKCS

CSGSTYEIARRKMLRALIEFRIRGVKTNIPFLLTLLTNETFINGDCWTTFIDDTPSLFRM

VSSQNRATKLLNYLGDLAVNGSSIKGQVGVPKLTTDVLIPVLHDPQTGIAIDVDSPVQPR

GWRQVLLELGPEKFAQQVRKFNGTLITDTTWRDAHQSLLATRVRTVDLLNIAPTTAYALN

GAFSLECWGGATFDVSMRFLYEDPWARLRKLRELVPNIPFQMLLRGANGVAYSSLPDNAI

DQFVKQAKDNGVDIFRVFDALNDLEQLKVGVDAVKKAGGVVEATVCYSGDMLKEGKKYNL

DYYMNVVDHIVEMGTHFLGIKDMAGTMKPKAASLLVGTIRAKYPDLPIHVHTHDSAGTAV

ASMDAAARAGADVVDAASNAMSGMTSQPSISALLASFEGEVVHGLNEDLVRELDNYWAQM

RLLYSCFEADLKGPDPEVYQHEIPGGQLTNLLFQAQQLGLGEKWLLTKEKYKIANKILGD

VVKVTPTSKVVGDLAQFMVSNNLSEEDVIKLAPELDFPDSVLDFMEGLMGTPYGGFPEPL

RTNMLGNKRPKLDKRPGLTLAPVDFAKVKEELTSRYGGNITECDIASYVMYPKVYEDYRK

ILEKYGDLSVLPTRYFLKPLEINEEIVVEIEQGKTLIIKLLAVGEISKKTGTREVFFELN

GEMRSVTIDDKNVSIENKTRPKATQPNDVGAPMAGVIIEIRVKKDQEVKKGDPVAVLSAM

KMEMVISAPAAGKVGELQVKESDSVDSGDLITNILK

>tr|C4Y4H3|C4Y4H3_CLAL4 Acyl-coenzyme A diphosphatase SCS3 OS=Clavispora lusitaniae (strain ATCC 42720) OX=306902 GN=SCS3 PE=3 SV=1

MSSNELVYERHMALIHKTARKWKFLRLTTSEFIFAFSFILNFIVGKLLHVFSQKEEVYNY

YNDKGNIFNQWFVKKGWAWTTGAIALFYTIEVSKSGFRLKVLRGAVLRWLVATCWWYLFT

QWCFGLPIMDKVFVLTGGKCASVSAEKLARLTESLHLFTLVDGVYESSAISSSQCRRLKG

SWEGGHDPSGHVFLLVHSSLYMFHEIKPFWPGWKSLYRSIIRFTRNTNKDNVMAKSRALV

VSNPSILVLGLLGLWWFMLLMTNMYFHSLAEKLVGLVFGYIAVAAIYWLPRWM

>tr|C4Y4R2|C4Y4R2_CLAL4 Phosphomannomutase OS=Clavispora lusitaniae (strain ATCC 42720) OX=306902 GN=CLUG_02634 PE=3 SV=1

MSFANKEKPDVLVLFDVDGTLTPARLTISDEMKATLEALRKKVVIGFVGGSDLSKQVEQL

GATVLSDFDYCFSENGLTAYKLGQELASQSFINWIGEEKYNKLVKFILRYLSDIDLPIRR

GTFIEFRNGMINVSPIGRNASTQERNDYEVFDKENRVREKLVNALKEQFPDYGLTYSIGG

QISFDVFPTGWDKTYCLQHVADENFKEIHFFGDKSYKGGNDYEIYEDPRTIGHAVNSPAD

TIRILKELFDL

>sp|C4Y4X4|LCL3_CLAL4 Probable endonuclease LCL3 OS=Clavispora lusitaniae (strain ATCC 42720) OX=306902 GN=LCL3 PE=3 SV=1

MSDEPLSSSEESPSVSVLHPKVLLLSAGFTGAAAASYFLYGRYVRRVKTYLDLTPAILDG

QRKLYGKVTRVGDGDNFRFFHTPGGVLLGWGWLRKIPDTRSGLKDQTLMVRLCGVDAPER

SHFGKPAQPFSEEALQWLQSYVGGRSVTITPYSIDQYKRVVARAQVWRWTGKRDVSAEML

RNGLGVVYEANSGAEFGENEGWYRRLEEKAKRRRRGMWSLGSKLVTPGNFKRQ

>tr|C4Y4X5|C4Y4X5_CLAL4 Phosphoacetylglucosamine mutase OS=Clavispora lusitaniae (strain ATCC 42720) OX=306902 GN=CLUG_03209 PE=3 SV=1

MLADKVNAKAARHSRPANAAFTYGTAGFRMKAELLDYVNFTVGILAALRSKYLGGKTVGV

MITASHNPPADNGVKVVDPLGSMLESSWEAHATALANASHDQLVDRIEALVAELQIDLSV

PARVVVARDSRESSPRLSQATIDGIESIPDTSVHDFGLFTTPQLHYITRTSNDKAFGECS

EAGYYGKMATAFQKMHRGGKKIEVTVDSANGVGAAKIKELGQYLGDDVVFTLVNNAYDQP

ELLNFECGADFVKTNQKAPKGVSLEHGNLYGSFDGDADRLVHYYQADDGAFRLLDGDKIA

TLLAMFLQRLLAKIDTAKLKLEIAVVQTAYANGSATKYVEDVLKVSVRCTSTGVKHLHHE

AEKYDVGVYFEANGHGTVIFSPEAEKKIFGYFAASDEEADAIAMLQEFTRLINQTVGDAI

SDLLAVLIALDYLQLSPAQWDKEYTDLPNRLVKVVVPDRTVFKTTNAERTLVEPAGMQAK

IDALVAKYPQGRMFVRASGTEDAVRVYAEADTKEHAESLSAEGSALLQ

>sp|C4Y584|ARGJ_CLAL4 Arginine biosynthesis bifunctional protein ArgJ, mitochondrial OS=Clavispora lusitaniae (strain ATCC 42720) OX=306902 GN=CLUG_03318 PE=3 SV=1

MRISPRLFLSKAERFVPKSGVYPQGFAVGGIHCGIKKPDVLDLAMLQNTSGKDAVAAAVF

TQNKFKAAPVQVSAEILEKSGGAINSLIVNSGNANAVTGTKGLEDARSMVAATDEAAKNT

KTASLVMSTGVIGNRLPIGNILSGIPVLGQQLGSTHAHWLQCAQAICTTDSFPKMVSKQF

SLNGNTYALAGVAKGAGMICPNMATLLGFFATDAPVSRSALQSILAFAVDRSFNSISVDG

DMSTNDTIVAIANGAAGGPLIENEDTPAFAALRGEITAFAQQLASLVVRDGEGATKFITI

RVVNAKSYADAKCVASTVANSSLFKTAMFGNDANWGRILCAIGYAPVSGSSVDTTRTSVS

FVPSEGEPLRLLVNGEPETVDENRALEILQKRGFGGGNRFGHRRKPRGHISDV

>tr|C4Y5F2|C4Y5F2_CLAL4 Pterin-binding domain-containing protein OS=Clavispora lusitaniae (strain ATCC 42720) OX=306902 GN=CLUG_03386 PE=3 SV=1

MSDKVHISGLQGTAIVGHDHWQKPVPQPIVIDVTFSTDFAQASDADDLHFSLNYAVISNK

IARYLTSTHQRNFHSLGGLGQALMSELADERAACSAVNLKVAAPKLDIRSVVSYSSSIIE

SEANTDAKENSSSFPVEGVYRIEGLRALALIGVFTFEREAKQYVVVDVDMGVKGHLDVSR

ISEGLHSFLERTNFKTVEALIKACAQWVLSFAPETNWAAVRVAKPNAIVYTDSVGVSGKY

TRADFADMSKTGLESPESPYGAKSAADAISTTPFDLPVTSPTSFSGQHTVYVAFGSNEGS

PTANIARALQLINDHPQMHVDAVSALYVSKPMYVTAQSDFYNGVARVVVRDMAPHDVLSA

LKHIEYNELSRVKVVDNGPRTIDLDIVLYGKACVNTQDLIVPHRAMLSRTFVLAPLCELL

PPDFVHPVTAEPVHDHWAKLRATPADSALQESPVLEQVVPGSGERSLRLGPQRATHIMAI

FNATPDSFSDGGMHCDLTEAEIQAVARGMVEQGATIIDVGGCSTRPGSTQPSADEETRRV

VRVVRSIRAARDLDSVLLSIDTYRAGVAQAALAEGADIINDVSMGTHDAAIFDVVASSGC

GYVLSHSRGTPATMAKLTDYSYSGDDNLVEYWIGAQEALPETDRAVVQGVCRELARQVCA

AEARGVRKWQMILDPGVGFAKNTQQNLSLLRHASRLKQYAQLDTNANRYTSFQGMPVLLG

TSRKKFLGEITGTSRADERVVASAAAVVASVQQGADVVRVHDVRAAREAAQTADAIFRGV

H

>tr|C4Y5F8|C4Y5F8_CLAL4 t-SNARE coiled-coil homology domain-containing protein OS=Clavispora lusitaniae (strain ATCC 42720) OX=306902 GN=CLUG_03392 PE=3 SV=1

MSELFAVYESDFQLAIQEARSNISAASAAPADQRQAALQNIERSTDEALEILEQMNIEVQ

SLPSNQRSSHNAKLRQYRSEVDQSKRQLRELLDAEDRNQLFGQRYTDEDDGAHDSQRKQL

LSNAASLDRTSERLRDSQRVALETENIGGNILNDLRAQREQIVNSRNTLSNADTYVDKSI

QTLKSMSRRITANKFISYAIIAVLILLILLVLASKFW

>tr|C4Y5G4|C4Y5G4_CLAL4 Integrase catalytic domain-containing protein OS=Clavispora lusitaniae (strain ATCC 42720) OX=306902 GN=CLUG_03398 PE=4 SV=1

MSMSIKRHLSMFTPSTAPADAMKPSDSRSSSEDSFKESSGLVSGLPAPNTVVYTASFVAQ

SIEQVMSRCVIEDLNPFEIKKKYHFQEWYYCFKRKFQTTFTSGEEFCTLSRAEYAKIFSG

SEESIRQAVNCMDSCFHKCIHHLLQNKAFFTVPRILTREFLENEFIPSIKDSDTNAYLLA

NEMRREQLELRASHGDYQPLLFHLLALSASPEEIRRTFMRVFTSKVSTPKQFTDYAKSFS

EAASATQEIDQQIHFWFASIVDSHPHWLSKDKTVSKESKFDNRRLEKAPPAQPHSHNGKR

GNFRKKFSKPYSESSSKTNKSSGSNAGKANDGSLYLVSYNEDISVYPTCTFILDSASDIH

ITNDKTLLSHVRSTTREISHGVTHIVNTIGDMKIELSDGHQLVLPDVHYIPELPNIISFD

RFRQADGCIAIDCNGDLINVHTLKVVSPYTTRHLVLNVRVLPCDAAATHSHVASHTPPLS

LFLINRLDTVSEDPCSDSSPDTESVISNDAEQETDPSDTSDTLTVKADALSQSWHIKLGH

PGANQFAAFKRLLSIPKSVVHVPLTQCRGCLGAKTTNHFPKESRGVTPITQPFEVIHVDI

CGPFDSHPAYDNTRYFLTIVDRFSRYVTAIPLARKSEASTHIQTFILRSYNALRATHYPK

QFRSDNGTEFINDNLLVFLDEHGITPHFTHAHCSSQNGIAERMNRTLEDKARAQIAHGNI

PLAFWPEVIRYSAFILNWTPRSNLQHKTPTHCWFNNSSIPCPTFYPFGCTAQVTFPLEIR

ANKLAPNSLECVYLGPAVQRTGHRFFSYDLMTVFDSDQATFLPEDLYFIKHDARIRSLSI

NHNRLPTALLPSIKLPAPFKRLSPEDLFREKQDLANLFSTQPRTTEKSASPPPVVPADST

TSSITESSPMPSRKVAKTSSLTPSATLAQLSTSSATNPVTAPPASPPPPSSSSPPHYRTR

SYTRSLFPKLSSSTKGGRQHDHLLKFPARSQQVSVAQSNKSKKSKKRSHDEVLMVLRTDM

VDTPGHKEEIPSTTSVLHAATVYAENHLNNSRPIPNSYSEAMRAADKQNWLEACNSEMQA

HHENGTFTLVPLPPNVKPIGCRWVFNIKDKGLYKARLVAKGYTQKEGIDYEETFSPVIKH

TSLRLLLAIAGRLKMHVHQMDVKTAFLNGDLKEDLYMRQPPGYKAVSKNSEDKTTEYVLK

LNKSIYGLKQAPLVWNQTINKTLVSLGFKKTIHEPCIYYKFDNKDQTLVALYVDDMLIAG

TNLAKINQLKIHLGQVYQMKDLGVATKFIGMNLEISSTGIQVCMKDYIRNLLAEYNMTDC

NPVKTPANKTNLDDLPDSTDLPCDENEYRSIVGKLLYAANTVRYDINYIVSKLSRYFASP

KVKHMDAAKRVLRYLKGTPTFGLRLYIIWTKKAL

>tr|C4Y5I1|C4Y5I1_CLAL4 E3 ubiquitin protein ligase OS=Clavispora lusitaniae (strain ATCC 42720) OX=306902 GN=CLUG_03415 PE=3 SV=1

MEDRKRSLEDTEGETQAKRSVPEKKPLDSLSEDGPLTQQDVVYFKKEAIWRQMRFYKLQA

AELAAEVSKHERRFQAFIAVHSLLESWYRQVVAISKHESPPPLDLAADSAEIDAVLDERR

QLLAKLLQPLASASDEGMERVLDAVKLAADRDAAIKLQEAAQQEVDTLRAHIQNLQKEKD

RRESATLKRILENSRITSNEPEPRGNGSQANGSAQEETKKAEAQTTAEKEAFEKLTVEHA

EMKAAFSSLQSQLDEITQKLAETEKSGSDLESRLASLSEDDLNKNERYASVVSQNKFLAE

NLAQSERLKDDLVQRIRELESREGNLIALVNKELEEENTRLKESLSKSENDLVRIRAARD

ELLGKQAILKSEMENKKTNEELNKLNQMLSRRLGELEKTRQDERNMQQDAALEKLEKGEL

VKRLQILGEEVKEVEQAFQDTRAVALEKLKDLVDHESLVKKLTIEKNKADQKYFASMRLK

DSLVAENKILKTQTAKSQELVAKFNDLEKTYVGKIDVLTKSINDYRVIKENALHENVKLQ

EALKQMTKGRETASKEKAALKSDLETVRREKNELLDELKSKRLNESKLEARLKSTESLLQ

KYRSNNTSSILQEDQKQLEALRSITKCSVCSKNWKNTVITACGHVFCEACVQDRLAARLR

RCPTCNKGFASNDLLTIHL

>tr|C4Y5I7|C4Y5I7_CLAL4 Inositol hexakisphosphate and diphosphoinositol-pentakisphosphate kinase OS=Clavispora lusitaniae (strain ATCC 42720) OX=306902 GN=CLUG_03421 PE=3 SV=1

MDPKQPDTAEKKAMASIAPMLESFTPKTSAEENGMPMITRRQSEEENRTSSSSSWDAFPR

LNKTASNSSVGNGTGSITGGNTAANATGNGAPAPLTKIGKIGVCAMDAKAMSKPCRRILN

RLIENGEFETVIFGDKVILDESIENWPTCDFLISFFSTGFPLDKAIAYAKYRQPYMINDL

VLQKTLWDRRLVLCILNHANVPTPERLEISRDGGPRISQELKDKLVEVGLPQDAVDRLCN

QKEPTWYMEDEDTLCVDGKKMTKPFVEKPVDGEDHNVYIYYPAATGGGGRRLFRKIGNKS

SEFDPTLSSPRTEGSFIYEKFMDTDNFEDVKAYTVGPDFCHAETRKSPVVDGIVRRNTHG

KEIRFVTELTPAEKTMARNISRIFRQAICGFDLLRVNGQSFVIDVNGFSFVKDNNEYYDS

CAAILRRLFIEAKKRRDVLKTRIPPSLINQSQFEQKEQKWVFKGMVSVIRHADRTPKQKF

KYSFRSPLFISLLKGHKEEVIIRAVPDLRVVLQTVKVAEEKQLEDLKKLRQLREALEKKI

DFPGTKIQLKPSLNAADPEIVDKVQFILKWGGEPTHSAKHQASDVGEQLRQNIKLLNKEA

LNDVKVYTSSERRVIASAHLATCSMLGLEDLPDDFLIIRKDLLDDSNAAKDLMDKVKKKL

KPLLRQGAEAPRQFTWPPRMPQPFVVIKRVCELMNYHREIMHHNFEKHDVSKFQETWCCG

EDPQLFRERWDKLFSEFTTVEKTHPSKISELYDTMKYDALHNRHFLQNVFAYDPNDNELM

QRLAKVCGDTINSSGLVSEYPINILAMNNFKLPSDPTSSGNSASNSTANLSNIANSTASA

GSLGWVLKGATASVSESSANNESNTKGKPSLKAPDNPFDHPTFARLRELYRLSKVLFDFI

CPQEYGIKDEEKLDIGLLTSLPLAKQILSDIQDMKKHDKAAVVNYFTKESHIYTLLNIIY

GSQLPMKIARNALPELDYLSQIVFEIYESGDPNSPSGQKHSIRLSLSPGCHTQDPLDVSL

DDDHYIGCIPRISLTRHLDMDLVSQKLKSRFPRVSLPKKFTPVNISSPLTTAL

>tr|C4Y5K3|C4Y5K3_CLAL4 Ubiquinone biosynthesis monooxygenase COQ6, mitochondrial OS=Clavispora lusitaniae (strain ATCC 42720) OX=306902 GN=COQ6 PE=3 SV=1

MFLCTRRLATSAKPILQDIVVVGGGPAGLSIVAALKNSPRTKHLTCTLVEASSLAPARSF

AEQAPPELTNRVVSLTPHSVDFMRTRTCAWPLVHEDRVRFYDNMVAYDSQDSDARIHFDA

SNVFDASGAPGEIAAMAEVANIQASLLAQVEKTDATVLDNSKVVAIENEAEKVQATDEAA

KTEETAKAEAASAEAADWPVVTLSGGQQLRARLLVGADGQRSPVRQYAGIEARGWAYDRF

GVVGTLRLQYEDFRSIAWQRFLTTGPLAILPMPGDSATFVWSSTPELADVLLKTDDSLFP

QLINAAMVLDETDLQYIYGMLQANPADPNVAEEIEWRMSKFSPDELDDKFPVPVAEVLAG

SRARFPLRFSQADTYAAPRVALVGDAAHTVHPLAGQGLNMGQTDVEALVAAIEQGVARGM

DIGSMLVLEQYVARAWPSNHMLMGVCDKLHKLFSTDFGPIVALRGIGLKSLNSLDGVKDL

MIRAISGRA

>tr|C4Y5Q7|C4Y5Q7_CLAL4 ATP-dependent DNA helicase II subunit 1 OS=Clavispora lusitaniae (strain ATCC 42720) OX=306902 GN=CLUG_03491 PE=3 SV=1

MEEPDTSRQFEIKEGIAFLIDLSESLFVPVNELDHRSQLLEILQCINDLMSDMVITFPSN

GIGIYFYNARETGKKFPKNSGITKAFSLNDLNSSNIKLLTNIVRDELDGFKPLQDRYPVA

EKQLDNLHTVLETLLKEFQAKPQYNRKRLFWLTNSDKPYINPNLKDGLRTVMSDFEANRI

LVSPVFLETYPRDGDQKFDLSLYENIFLNTNFLARAQKNQEEGSPLLNTTVSSQIRSSIL

RLKEVRRIQFSCDLVLSDGPGIGGKFGCSVKGYTLFNHESIRQFRQVYTGGEGLKLVHHD

TSYLRSDTKESLDLDKESQPTQTLKGISVKFANDKEGVAENEKLLLFKQDVVEYMRGYSF

DHTPGEQSEDEQDGNDNDDDDNNEDDDESDRKPVPFSNPPYLKLLCFRDISKFQHLFNIK

PPVFITADSSDGMNSTSKEGGYTNSFNTFRSLYQSCLRLQRYAVLFGCVKRNSSPSLYAL

YPTNTEGSSTEIKDKQFPDGFLLITLPWLSEIRSLPDYMLTEHHRYFLPETESVVPPELA

SLCSKLMGLCKVDDSYDPSVHPNPVLNYFYKTIKQEALQIDIKDEDTSLEQNDWTVSRLL

SIRNSLQENVDTRQILQFINVYLNKIGNAEVAKRTNEDNNSGASKRAKAAPLTEAAIVAL

WQTNSWSKVTVAQLKEFMGKYPEIPSATRKADMVANITSFLESRQ

>tr|C4Y5R5|C4Y5R5_CLAL4 Glyceraldehyde-3-phosphate dehydrogenase OS=Clavispora lusitaniae (strain ATCC 42720) OX=306902 GN=CLUG_03499 PE=3 SV=1

MAITIGINGFGRIGRLVLRIALSRPDIKVVAVNDPFIAPEYAAYMFKYDSTHGRYKGEVS

SNGDKLVIDGKEITVYGERDPAQIPWGKAGVDFVIESTGVFTTTEGAQKHIDGGAKKVII

TAPSSDAPMFVVGVNEKKYAGEKIISNASCTTNCLAPLAKVINDTFGIEEGLMTTVHSIT

ATQKTVDGPSHKERRGGRTASGNIIPSSTGAAKAVGKVIPELNGKLTGMSLRVPTVDVSV

VDLTCKLKKSATYEEISAAIKKASEGELKGVLGYTEDAVVSTDFLGSSYSSIFDQKAGIL

LSPTFVKLISWYDNEFGYSTRVVDLLEHVAKN

>tr|C4Y5T8|C4Y5T8_CLAL4 Thiamine phosphate synthase/TenI domain-containing protein OS=Clavispora lusitaniae (strain ATCC 42720) OX=306902 GN=CLUG_03522 PE=3 SV=1

MAKMKLDLSAYLVTDSTMIPESSSFLSQVQQAVENGVTIVQLREKNISTRDFIDRAKDVL

KITRPRGVPLIINDRVDVALAVDADGVHVGQDDMPARIVRQLIGPHKILGVSCGNEEETT

EVCEQKVADYVGLGTLYPTQTKDVKNVCGPIGVRRSLQVLKKYKEQGTYVQSVAIGGINS

SNASKVMYQCRVPGYAVNGVAFVSCIMAAPDATQATKKLLDQLKNSPPWVTASTATLESL

QSKPLVHHITNNVVKHFSANVTLAVGGSPIMSELPDEFDELASLPLPTALVINLGTPSST

LMSVFLEGIKVYNNHGRPIVFDPVAAGASKARLDACRVLLNAGQVSVIKGNLGEILAIDK

LTSSSPFAKEANLMRGVDSVAEMGPAEISEVCKRVALEFQCVTVITGEVNHIFDGTRPDS

QVVTIQGGSPLMGSVVGIGCALGSVIGVFVACAQAQKAQLGQAVELALKVYNSAGRQAAV

DHKQGLGSYMIKFLDELSESAKPYA

>tr|C4Y5X1|C4Y5X1_CLAL4 Leukotriene A(4) hydrolase OS=Clavispora lusitaniae (strain ATCC 42720) OX=306902 GN=CLUG_03555 PE=3 SV=1

MDVVNSRRPKVSPEIDPSTLSNYTNFKVGPTKLSFDVDFEKKIVSGSVVYDLEKLTAVDN

VVLDTSVLKVKACSINGKSAAFSLSDVVGPLGSPLTIEAETPEKKLEVVVDFETTENCTA

LQFLDKEATDGKSSPYLFSQCQAIHARSLFPCFDTPAVKTAYKFTATSPLPVIMGGRPVS

VEGKVYTFDQPIPIPSYLVAIASGDITKLPIGPRSHVYCEGVKVKACQHEFEADMENFLQ

AAEKLVFNYEWDQYDALVLPSSFPYGGMENPNATFVTPTLISGDRENVDVIAHELAHSWS

GNLVTNCSWEHFWLNEGWTVYLERRIQGSIHGEATRHFSAIIGWSDLENSIKAMGDSAER

YSTLVQDLKDRSDPDDAFSTVPYEKGFNLLFHIEQTVGGKEVFDAFIPHYFKTFRYKSLD

TYQFLDTLYAFFADKKKELDSIDWDTWLYKPGMPPIKPAFDTTLVDQCYSLADKWFNAVS

KSSSTNLHSVFKPSDIVDFTSNQSVVFLDTITSYNKLPDFKWANHVDALKAMSEIYAAYS

TSSNAEVLFRWFVVQVGGHNTSYYDKLGQWLGTVGRMKFVRPGYVLLNTVDHDLAIKYFT

KFEATYHPICKAMVRKDLGLA

>tr|C4Y5Y1|C4Y5Y1_CLAL4 Uncharacterized protein OS=Clavispora lusitaniae (strain ATCC 42720) OX=306902 GN=CLUG_03565 PE=3 SV=1

MASYLPQKEFETVNIYNYDEVPENSSWASALPVAKGLYNPENEKDACGVGFTCHLRGETS

HKIVSDAKSLLCNMTHRGGELNPKDGDGAGLLSSIPHKFLVREFKYHFGVDLPPLGQYGT

GNVFFKKDEVVFEKSKKTFEKIASSLGLKVLGWRQVPHDSSILGPASLSREPYILQPAVV

LAETFGTDITPEEFEAKYKKDFEKKMFILRKQASHTIGLHNWFYICSLSSKTIVYKGQLA

PNQVYAYYYDLVNADYEAHFALVHSRFSTNTFPSWDRAQPLRIAAHNGEINTLRGNKNWM

RAKEGVMSSELFGEELEKLFPIIEEGGSDSAAFDNVLELLVINGVLSLPEAVMTMIPEAW

QNDKNIDMKKKAFYEWAACLMEPWDGPALFTFADDRYCGANLDRNGLRPCRYYVTDDERI

ICASEVGVIDVEPEKVLQKGRLQPGRMLLVDTKEGRIVDDRELKSSVASRFDFKSWVTAN

MITIDDLFAKLSTRDVDLDDYHMTEEGSKSVQTDPRLIAFGYSNEQMNVILAPMAEAKEA

LGSMGNDAALACISEQPRLLYEYFRQLFAQVTNPPIDPIREEIVMSLECYVGPQGNLLEM

KPSQCNRLLLKSPIISSKELNAIKHMQKVYPTWSVANIDITFERSEGIQGYINKIEEVCQ

EASKAISENKKVIILTDRATSFNRIPISALIATGAVHHHLVRQKQRSKVAIIVETAEARE

VHHTCCLVGYGADGINPYLAIETLCKMRDEGLLKVDFSNDKIISNYKSAVNAGIMKVMSK

MGISTLASYKGAQIFEALGIDNSVIDRCFAGTASRIKGITFEYIAQDAFSLHERGYPSRE

TIKPIALPETGEYHWRDGGEKHVNDPAAIASMQDAVKNKNEKAYEAYSKKEYDAIKHCTL

RGLLDFDFESATPVPIDQVEPWTEIVRRFFTGAMSYGSISMEAHSTLAVAMNRLGGKSNT

GEGGEDPARSQVNANGDTMRSSIKQIASGRFGVTSFYLADADELQIKMAQGAKPGEGGEL

PGHKVSESIGKTRHSTPGVGLISPPPHHDIYSIEDLKQLLYDLKCSNPRARTSVKLVSEV

GVGIVAAGVAKAGSENILVSGGDGGTGAAKWTSVKYAGLPWELGLAESHQTLVLNDLRGR

VILQTDGQIRTGRDVAIACLLGAEEWGFATTPLIAMGCIMMKKCHLNSCPVGIATQNPEL

RKKFKGTPESVINFFYYVANELRQYMAKLGFRTINEMVGKTELLKVRDDLRNTKNANIDL

SPILTPAHTIRPGVATHCVRKQDHKLHVRLDNKLIDESELTLSKGLPVTIDCNVVNTDRS

LGTTLSYRVSKIFGEQGLPHDTIHVNVKGSAGQSFGAFLASGITLELEGDANDYIGKGLS

GGRIIVYPPKESKFKAEDQIIAGNTAFFGATSGTAFIRGIAAERFAVRNSGAVIVTEGTG

DHGCEYMSGGRVVVLGSTGRNFAAGMCGGIAYVLDMAQDFMTKVNSETVELSSVTDPTEI

AFLRGLIEDHRHYTGSEVSDNILNDFNRILPRFVKVLPHDYKKVLEKEKQKQEEAKKNEL

NNFLKSIKEDPESDATNGEAAKIKRGHVNHAAKSAAHEPKVLDLEDTIKDTEVAKKAVAK

LDKTFGFMKYKRRNEKYRPAQERTKDWNEMTSRLTKEELQVETARCMDCGVPFCTSDTGC

PISNIIPKWNELVFKDRWYDALQRLMMTNNFPEFTGRICPAPCNGACVVGINDDPVNIKS

VECAIIDHAFEQGWIKPNPPQHRTGKTVAIIGSGPSGLAAADQLNRAGHSVTVYERSDRP

GGLLMYGIPNMKLDKRIVQRRTDLLAAEGVEFVCNTTVGEDISAEELKATNDAVIFAVGS

TIPRDLKIPGRDLKNIDFAMKLLHSNTKALLDGTLEEIRKTIAGKNVVVIGGGDTGNDCL

GTSTRHGAKSVTNFELLPLPPNARPKDNPWPQWPRVFRVDYGHTEVATHYGKDPREYSIL

SKEFVGDEEGNVKGINTVRVEWKRSDSGAWQMAEVPGSEEFFPAEVVLLSMGFVGPDADK

LQVNKTKRGTIGTTDPSGYRVSANENVFAAGDCRRGQSLVVWGIQEGRQCAKEVDVYLMG

TTRLPGNGSIEQRDFKLLEELAEKI

>tr|C4Y618|C4Y618_CLAL4 Succinate--CoA ligase [ADP-forming] subunit alpha, mitochondrial OS=Clavispora lusitaniae (strain ATCC 42720) OX=306902 GN=CLUG_03602 PE=3 SV=1

MLSKQFVRGFASTPVARNYASTIKNLKVNKDTKVIYQGFTGKQATFHAEQAIAYGTKVVG

GTNPKKAGTTHLDRPVFANVRDAIKETGATATGIFVPPPIAAAAIEEAIEAEIELAVAIT

EGIPQHDMVRIAQILKTQSKTRLVGPNCPGLIAPDQCKIGIMPSSIHKRGRVGIISRSGT

LTYEAVAQTTNVGLGQSLVIGMGGDPFPGTNFIDALTLYLNDPETEGIILIGEIGGTAEE

EASEFLKQHNLTRPEGPKPVVSFIAGVSAPPGRRMGHAGAIVAGGKGDAKSKIAALESAG

VVVEKSPARLGNSLLQEFKNKGLL

>tr|C4Y695|C4Y695_CLAL4 Putative tRNA (cytidine(32)/guanosine(34)-2'-O)-methyltransferase OS=Clavispora lusitaniae (strain ATCC 42720) OX=306902 GN=TRM7 PE=3 SV=1

MGKASKDKRDLYYRRAKEEGWRARSAFKLLQINEQFNIFDGVRRVVDLCAAPGSWSQVLS

RELNKNGDKKEAKIVAVDLQPMAPIDGVTCIQADITHPKTLQKILDLFGGEPADFVCSDG

APDVTGLHDLDEYIQAQLILSALQLTTCILKPGGTFVAKIFRGRDIDLLYSQLGYLFERV

ICAKPRSSRGTSLEAFIVCLGYSPRPGWNPKLELTKSTEEFFEDAGIGKSYILENMDLPQ

LEERDISTFISCGDLNEGDSDATYSLNSSAEKRNLQPVQMPTAPPYKKALEMKRNGGLAK

R

>tr|C4Y6B7|C4Y6B7_CLAL4 Serine/threonine-protein kinase Tel1 OS=Clavispora lusitaniae (strain ATCC 42720) OX=306902 GN=CLUG_03791 PE=3 SV=1

MSTFDLTRIVDALQSTKIKSRNDALGLLEGLPASKLRLETRQFNTLATALLKLIQRERDI

YAHNSTNPVVSRLSTASNFLWELVDEALKKPSGRQPRFKHLLAIVTTISSCFFTADSVIL

GPCATSFAKILKSSLSHQFFLTHLTEETWTKLYKFHKRALEDILDHKDDIHTLNETLLQE

LYQSLYLLIGGESPVVLLPLLKYDIYFPLLRILKLTLRLFKNRESMTVVVAFKVINKLLI

VLSTEDYMFLHELINIALRSFNMFVSSPLESIQKQIAIFLNLETVYRYVEISSLPKLIGS

DGIITSSDDEHESIPESSVQLYNIGSLIQSLMIQVQSVSVRLNPNDIGFTFASTNIEFCL

HGMYLKTDRAIPWLLLRGLSKLIVKYYDMREKLSPNIPLANFHEMSMHMSVSGGSMYKRQ

KLQDQKTHLWTCSNASSFFNSLITSSNDPKEQICGLQLLAFHAELFHLTENASPMAKEEH

ESSKTLDFNESTILDINLGYGDESQETIVAITNILKVLNVKSLTFWAFLACRSILFNTEI

NVASCNPTLTKRLHQILKLLIPFINDVEIGTLAADIFFHIVRNQSENDVSKLLDKTIISQ

LENLIDLLELSGPFSLHKTALGFFWAITKVYADAEVSRMSNFTQSLGRWFITKWNEKYVD

SSSGLKRFTGGRSPSPEDVSTFLLWMSGKNVLLDLSKSTIPLNSSLQDLEILLRSANDHS

ELQSFIVLDESVRNNEKAFTEISFPMLTQNEFVFDTIVARILDVSELVCHSGETSDVLSS

WASSISIIADILACNCLESSSLFRNSAHQLWNTLSTSIHSRHDASLALSCVLESKVTRKQ

LTDTFFPFNTFDSYFQRFFGRKDQPHEDNHKNDSDFDSEFSTQQKGKSRTASPTDVYDRS

SYEEAISYFKFYASLKKIDDENVLRCIETIPSKERLSCLRILLQSSSQNSTNNFNANWLI

RLVRALGEGPLSDQELDRCDTVIEVSCDLLMLTIPTAVANKEESLLKDCIDLLSYFISCC

QKSLLVTGKSQVCVWKMIFSLLIETDFEFANRSDIQTLFLDSLERFSNAMTIEIAQSVQK

FLNFLDLSEQMIFYKELFSRFKVYQESVERFATCCLFFCLISKGNFQLRISVLFNLIECT

KFEFFEPYLRQSIKLISELSKKHGVKSLFRSLRFELLKCWWGHQLNILKFPHHLFGYIDT

RSFLVENYKEIIAISLSIKHPKSEEEYIKIIDHLSKIRSSDRQSMLSDSLPLIVPLAYTT

EGVRNNIFKIIAHLFNEAYKAYMREKLLLQILQTIKLTDTKSEQAFKTALKKQSDPLFQS

EVILDTSAQAVVSTLSSVELIGALVRKYWSSEKSEFWNLKTVYFLVRHIGKDISDSVEND

TLTCLRRIKYVLSMSQIKINQFELVKLLIDICCPLLITSLRDEVLPILNSIDTSFLNLAS

PEKSIPLLFGLLNTASLVRDGMNNVSFFTSLDEILHFQGSKLTSALPLMRAMIDLVMGSS

IHLDMKSVEDFLVDPDFEGAIDKNLSTVFMLISRLYPHIASRQITTTNVRLIKIFIKMRH

SESSSDELKMWMSSTLSYFYLSGYAHENLDEFILDQEYEGVNKEEFVNKYGSLDPFMKSL

LSYGEKCDFINAAFIETILGALLWKYERRPVDVTKFLPFEEYYPILKEYLIPIDFHSCVL

LNSEYDMQIKSEDLNDFLAKFKDILVNDSYEVWVSQFLLSLIQEIAKQTSIASLLASSVL

QIPSLSVEILPSFICFYIFLTGESGAAKVYDFFQVYWSNFQRSPDDKSIDLIKNIILSIR

MGSVLQVESFRNLYKKLNVDSVFKVVKEGNLAKTSLMLFEDSIDGKIENVNWSDHRDTIA

TIYKSLGEEDMLSGLPEDVSFDYAINLIDDLGSSSEKMRYASGLLDASIALTGNSHSRNV

IRSMLDDGHLGTSSLVDRSLKDKEDSFEWAWKLNQWDVVTSSCPTNPHEAVFSYFKSIKE

DVSASQRIFENSILNMMNCSTEFSEGKTNYKRKRESAHSWFETMACIHVSNRILSSPICQ

FSEEIESFEKLTSWFQSASINFSENILQARQLAFVIYGSHALPVREGSISSITSGTSANH

DLCWQAAVGETFRRANLYQENLQLQKMVSASIFMNEFIHASDFSDDNIGTQLQRLSNFQA

AHSLWTQGKTGISLAMMNDLKETGLIFTFLHRTCVDKNLMKATLVKWLAESRQELGTRLL

ERLVDPMREDIEMVENVSSRSLIYHSLAHFCDSQYKSHTIEDQIKEYSKRVDRKKREIDE

IKTHYGQTSVSATEKKMVQKYYSRLKSQVTSDTSKLESLRETKRQFAKNAVTFFLKALLL

NDDADNDDMDKFFSLFLELSNEETLQQCIKNDLRFLPSYKPLIWCTQLLSRISNEKTTFQ

FSVQDLILRVFQEHPFHSLYYLVSLIYHEEYSKTSNLKSMLPRIEAAKKLREKLATLDLK

YTTEILLPIEQLCKECISLAELKTSKGRSLHLDKLQIGEYWLKHLPSIPPPTFSLAVSKT

GYSNIPYMMNIGHTVSIATSGISLPKIATFTLSNGDQHKMLLKFGTDDLRQDATMEQVFE

KVNNFLRRDKETRKRHLQVRTYKAVPLGHRAGVIQFVPNSKAFIEVIRPYHLKSDSLKGE

KARQMMKDCQTESAPHRISVYNRISQRIKPVLRQYFVDHFVTPDDWFDAQQVYTRGIASS

SMVGYVLGLGDRHCNNILLDQYTGEPVHIDLGVAFDQGKRLPIPETVPFRLTRDIVAGFG

FTGTRGTFSESCKHTLRVLRDYKEHILAILDVLRWDPLYSWSISPIRMKKLQEEDPGVEG

VNAHEDGSEASTAILTVKEKIHHEGLSTEATVRELIREATSEDNLALIYCGWCPFY

>tr|C4Y6I1|C4Y6I1_CLAL4 DNA replication licensing factor MCM6 OS=Clavispora lusitaniae (strain ATCC 42720) OX=306902 GN=CLUG_03764 PE=3 SV=1

MSNFVSSPRSIPSDAAQLLDSSRISSSLGRSANSPSSSQPNINFSDASQSQGARERDVRM

PASIRSAVEVPKVVDVTGEKVRESFEQFIEEFVDESSADDEWSGKIYLAQIESMRVFEYS

TLYVDYQHLVGRENGVLATAILEQYYRFNPFLLKGLHRLLKKYAPSLLYMNLLGNTGEDG

KQAHDNSSSTSSGSTVNERVIQISFFNLPVTHRIRDIRTDKIGSLMAISGTVTRTSEVRP

ELYKASFTCDMCSAVVDGIEQVFKYTEPTACPSCPNQSYWTLNVAKSQFIDWQKVRIQEN

SNEIPTGSMPRTLDVILRGEAVERANPGDKCKFTGTEIVIPDVSQLGLPGIKAQSIKESA

GNASGLNSGVSGLKSLGVRDLTYKLAFFACHVSSLISKASGGESGEREVEFQGPNDQEAL

LNSLSDSEVSQLKEMVKDEHVYDKLVRSVSPAVFGHETIKKGILLQLLGGVHKQTVDGIN

LRGDINICVVGDPSTSKSQFLKYVCNFSPRAVYTSGKASSAAGLTAAVVKDEETGEYTIE

AGALMLADNGICAIDEFDKMDITDQVAIHEAMEQQTISIAKAGIHATLNARTSILAAANP

IGGRYNRKMGLRANLNMTAPIMSRFDLFFVVLDDCNEKIDTQLASHIVDLHMLRDEAIDP

PYTAEQLSRYIKYAKTFKPKMTKDARDFLVAKYQELRSDDAQGLGRSSYRITVRQLESMI

RLSEAIARANCTEEITPTFVSEAYDLLRSSIIRVEMDDIQIDDEIDDVAMHDDDDEGGED

NEDAGRPESNYNAPDYSGRTQARRAPKATINYDKYVAMMNLIVKKISDDDSLGGEGLTKD

EIVDWYLHQKEDDLETEQDYHNERALAYKVLKRLEKDRILMSVTQNINDDVLPGDEYDRA

QQASRTVYIIHPNCAILDFFDNQTTERDNDVGEE

>tr|C4Y6V6|C4Y6V6_CLAL4 protein kinase C OS=Clavispora lusitaniae (strain ATCC 42720) OX=306902 GN=CLUG_03890 PE=3 SV=1

MSQSKDKVLADIRGKIERENKLIQGFQAVRKNTSNQEVIQRCNNQIRETQTNIEYLRDTL

DKLSMQQDTVETSGDERQRHRGSSVSKPLYSRFDLIKYNCPSLGHKIQYMLQQLQFKLQV

ENQYREANEKISHLYLMDGDKPSSNAAEGGKLDSDYRIQLLNKSLRKYQGMHVNVEEINR

DLEIMNIPKFARKSLTGRITINVVCIRDIDHIASPMSHKRAESFVSFKIDDVEKARTSDS

RTGKFNEDFVIEVDKAHEMEIAVYDKSNNQRIPVALTWILLSDIAEEIRKKKVANEQVTE

GWMPASSISSPSHGSAHANMTSANTPNIEGVMDTASSSSNKSPKNVQISSWVNLEPVGQI

LINLSFEKSQALGHATFMDALGRHGAIRQKKEEVYEQHGHQFVQKQFYNIMCCALCGEFL

RYTGFQCQDCRFLCHKKCYQKVVTKCISTSGTTSNDEAKLNHRIPHRFEPVSNRGTKWCC

HCGHILPWGRKNVRKCSECGVMCHSQCTHLVPDFCGMSMEMANKILATIKSTKQAPRIAQ

QPKSSSSAPEVPPKSASVSPQKPRQTGYETSYSNAQAEFPQTQDPRVRGAYNNVAEYEDE

THATKLPTTIRNEFQPAVDSPYTRHATRRPPPQQEVEELMPSQDVDDISTRHDYTFVSNR

SDDFEHVPQRTPIDHPQNNPFDIDQEQQTVFDNFDYHNQSSAITQVSSAQENVFMEPPHE

AQIRQQEEHHSSHRTHRSKRRRRIGLDDFQFLAVLGKGNFGKVMLAESRTTQKLCAIKVL

KKDFIVENEEAESVKTEKRVYLTANKEMHPFLLNLHCCFQTENRIYFVMEYISGGDLMWH

IQKSRFSAKRAKFYACEVLLALKYFHDNGIVYRDLKLDNILLTTEGHIKIADYGLCKENM

WYKSTTGTFCGTPQFMAPEIIAGKPYDRSVDWWAFGVLMFQMLLCQSPFKGEDEDDIFNA

IEHDDVRYPISMPRQTVLVLQALLTKDPAERLGSSERDALDIMEHPYFQDVNFDDVLNRR

IQPPYLPELTSEHDYSNFDQEFTSETPRLTPVETVLTSEMQEQFRGFSHIADDAPV

>tr|C4Y6X7|C4Y6X7_CLAL4 Putative lipase ATG15 OS=Clavispora lusitaniae (strain ATCC 42720) OX=306902 GN=CLUG_03911 PE=4 SV=1

MPNEKTPLTSRSRRRSKDWYLSCVLSIGTCLGFLLWFVFTQDRGQVLKQPLLAATPQTDT

PEHEMTAEDILPVGLSQEFSLKHIFHHGSGKYNRVHRRLDITPKFLEQYSDQLETPLTPD

EVKESSLHDAVSAMDWPNSMRDENPWTVSLHVRERARRAKVTRLAERHTPNFLDSYLEYA

RSVKGNPARLNAIGLEWDPETDIRVPDVNDRNTLVSLALVSSNAYVRLPQNDDDKKNSDW

IDVGDPWSPEDEHDDLNFGWLENGLRGHIFVSNDSKTVIIGIKGTSGAGLPGGGSDETMA

NDKTNDNLLFSCCCARVSYLWTTVCDCYEKTYTCNQDCLERELLRQDRYYQATLDLYRNV

TKLYPPETTDIWVTGHSLGGALASLLGRTYGLPVVAFEAPGEMLATKRLHLPQAPGIPKH

LEHIWHIGNTADPIFMGVCNGASSSCSMAGYAMETTCHTGQICVYDVVTDKGWHVNLLNH

RIHTVIDDIILAYNETAPCVEQPPCRDCFNWRFTSHDDSEPDKPSLPNPLKPRPTTSSTT

EKTQANTLSSSNIGQSTKTQTSSKSGSPPKPTETEKPQKCLKRTWYGWCSEWGDDE

>tr|C4Y6Y0|C4Y6Y0_CLAL4 Postreplication repair E3 ubiquitin-protein ligase RAD18 OS=Clavispora lusitaniae (strain ATCC 42720) OX=306902 GN=CLUG_03914 PE=3 SV=1

MADFDPSDWKNTRLPKLAQLDSLQRCLICKDFLRAPVMTSCNHTFCSQCIRQHLLSESSC

PLCKAEQFESNLKRVILLEEIVSCFQDLRPALIALATQSQEQDDIRSTQNETNSVLKNVG

HRDNMVSSSSEKEKKKLQEEKDSGVIEVPDTENVSCPVCNERMPADLLQRKHLDECLRGT

TRPKRRRTEISSFFQPRKKREIDHEHFYFSEAHKHHHEARRMPKMDYASLSTPKLKEKLA

QLHLSISGTRSQLEMRYNHYYLLHNSNLDSSRPVSDLELRQKLKQWERSHSAFSAAASPN

PVFGDSISHKSITDKDFSVSVWMDKYKQEFHALVRAAKRSRRKRGALESTITETTKSGVD

FEEVTVDKDDSIEKNGSKINQNGSSNRGTQSLQGEKITESSIGSFSVPGKSEELHAAETP

NAEKSSGVECHEPKIDASLNTASKIKINSKQDEVMNGDAAEFDFSSSTLFVKRHE

>tr|C4Y6Y7|C4Y6Y7_CLAL4 Ubiquinone biosynthesis O-methyltransferase, mitochondrial OS=Clavispora lusitaniae (strain ATCC 42720) OX=306902 GN=COQ3 PE=3 SV=1

MIRCPHIFQKTVIFRSLASTARRCHEFPKFTAASDDEMSHFNALASSWWDVDGPQRILHK

MNLLRMDYINEMVRTHLKLNEGVESEDEETFIPAFNVDLLPGPIKSTIIEEQEKKRDEIL

EKNKLNVLDVGCGGGILSESLARLRFVKSVRGIDLSSDVLEAARVHKSKDPVLNSKLSYE

LKAVEDLPRDEKYDIVTVFEMLEHVQYPSKVLAEVFDRVEVGGWVFLSTINRDLISWFTT

IFMGEHLLRIVPVGTHTLEKYINEQEIRQWIKKSDYAQSFTVADSRGCIYVPGCGWSFTE

CPQIGNYFMAIQRTS

>sp|C4Y732|EXO5_CLAL4 Exonuclease V, mitochondrial OS=Clavispora lusitaniae (strain ATCC 42720) OX=306902 GN=EXO5 PE=3 SV=1

MSRVLTFRVPPEQQQAPKPLLSDRSSTWPLLPQLAPTAVRPASPPSPKEELVLNVFGRGP

DSLLMPPKHSLPPPFAFYTQFNGDASYMAEPRLSVTKLLVSSWCELREYYEVYAGSPRRV

PTARLTQGTDYHRVLEERSHRAIDPSTVSARVEEILGEMPEERVLALTQGGSMAFKLAHQ

WVEQILVRCLAVAHTGYAREMHLHGFLDLTSGELATSKSTIGQGVLVNGIADMVRLEPAP

YGHDRALPWDPQAVLELGPALEGAKARMDRLATNHTLEVRDVKTRAYNNVPKQSSVVEAA

RDQCMYYAQFLTTLAQNEEYAYQSLVENFTRRHIQTSHPLGEAHAAALLITNFGVLVEDY

KALARGDALSFTPFDNATTFYVTEQPPEAYSLANFVDEPTFRTLLADFHGDYFADVDISV

LFREWKRPLTPAYFCARAAQALYLFEKLKPSSVCVEYHNVKTGRIIECKSFPFDKHTLEE

ASKRAAQFLGRYSTTHKHR

>tr|C4Y755|C4Y755_CLAL4 E3 ubiquitin-protein ligase listerin OS=Clavispora lusitaniae (strain ATCC 42720) OX=306902 GN=CLUG_03989 PE=3 SV=1

MSEKFSAHSREPGDLGYNGFVVSLFYLAPVEVEQIRDPHVVITLKGLTKKNAVTKEKRLA

EFLRMLSEGQMDVHDTFILMCWLQLYPRMAIDSSKAVRQLAHQIQATYMDRIGGKEFSKY

LKSSIASWLQSLYDEKAVSASSYRSLLESFGNDKERVNTKIWMVFHEQIINYCHAVLAHE

TASTITDERYETADDVLLKYYRAQNGAIQMLLKIISLANDNSDFVISESAMTQVNEILTH

EPFWDHLGLCCAGDSINVNVFRGYLSLLKILFASDDKGFPMPFTSRMDNVRSVYRLISKK

FIKHVKLQTGNASNVIYSSVLIDFWNTLTTLTSVTSWNDDVRKKHKLKKNFWVLGGSKSY

SRLKSYIKLGPCQSDPVFYVILKNFFCALAQAGIESEEDFLFLNFTSSKDAKTIIESSLG

AQFQEIGRINGFAYKDSCSQCLYQVLDLFQLPDSQSRTFAAYIFDLVLDGLSVPSRRVNE

KEIKQRSVHNLATYADHHDIDLISFNSALTNHLGRKEPFRTDEYQFTQSFESLCETYISL

LLELSSHDSFVDLQTKLVESLSELFEVEEVSMGFKLLTCFLRTSSEINGAVEEWAATLAS

YVTDTFVDQPLSVLELLLKKKIRLNEKEVFGDFFSKIAEVSPSDLAKLLVIAKKFDVNVK

EVPDLWNYLVELSKKTSRSSQENEAVFSYLGTPEIFGNVIGSSNTESFKLDLIQNISHLR

LPVLVHEDLQLPIKDLVSVALGHISSDASREFLALVEDKDLIKDSVFQRITKKSYADDFR

DIADYVAQNPHIMPLDAFQKEITMALNAIDLYSIAISNPLGQTAHIVETCTEQNTFLSEH

VLSIGKFLFDYLSFESQEVSVNLVTLIGLCAEYTQDYNFIVDVDRSSITLLDLKKDLLTY

FLEHCSVDNVVLAQFFNASVSDRSSLIFEMDSSVSGKGPFNALQFYHARIVAALFEPIFE

NMPSSTFETLDIQFSKLTGHPLKLAILLCTAKKFLDETNKLDRVQGYVFGEILGIRNSTQ

ILKEGPKWLSLASEFLKLGTTSKSLIEVLPKHKLGMLISSLAAWLECDIAYDDEFIGIRS

LMANFLTLLLPLTVTELPEKAWEVAVNLCLNNFATVQVQAKELELKYFTMKLLNVLSSLP

TLDHYPSWTESRISVIEELVELMINEEVEGYCSKCHNQPVALSNEMVERILKKENVPNKI

IGDNLDRFFDLVIHSRFLTMQRLATFLLGKYIRDSQEVFVVEHELKISSLHDQDTDNLKA

TLPTVLVDAISASPHFLADALDAGELHSAAKFLWSWLLIFTYFKDTTYSIKNDYISQLKE

KDVIRTLLDSIFSVVDVSDGTFTKSLVMGALDKNEKVDPEKCTIQSYDVLDGCIGEPVSY

EMRFLLVHLYYLSFQYFGTQVSQWYNEIRDLQLKQQVERFSVRFVSPILIDKMLVDIDQV

KAKLTSKDENLSIKISKVSNEIKSVYVIDEQTMEMVVKIPDSFPLANITVEGPMRLGVKE

NQWKAWLLASQRVVSLMNGSIIDCIELFNKNVNLHFSGFEECAICYSILHQDHSLPSKVC

PTCSNKFHSACLYKWFKSSGSSTCPLCRSAFNFKASRS

>tr|C4Y757|C4Y757_CLAL4 ATP-dependent DNA helicase RRM3 OS=Clavispora lusitaniae (strain ATCC 42720) OX=306902 GN=RRM3 PE=3 SV=1

MAQKKPRQRSIASFFAPAPARKKVSTPEKVVVARADSLLSEMASARSTFDDFASSDTSFG

SPEVTNFKNPVLSRVSSNLTGSPPRKNHGISGSSQEKRQFSRTFFDLDARPKKVPRRVPK

AASPSLSTSSVSLSVEQLAIIDAVVSRGENVFFTGSAGTGKSVVLREMVSRLYAKHGASH

VGVTASTGLAACNIGGSTVHKFLHIGIGTGSPQEIAVKIKRNGSARKKWTSLAVLIIDEI

SMIDGILFEKIDKIAQILRNSTAPFGGIQLVCTGDFFQLPPVSKNQSARFCFLSDSWRKA

IRNTFILTTVFRQKGDSELIEMLNSLRKGKLDDAMVAKFRQLGRKVVYDDNIEPTELYPT

RQEVKAANQQRLRFLPGQSNIYKAQDSEKDQFKRKLYDNLMCEEILELKKGAQVMYLKNH

PDNLVVNGSIGTVVGFITENLFKTLFDVLGEQTFCNMSEEYQQLLTLLCDLIGNRSMTSD

QTRVFNNLPPDLHAKASRLIMEAYKQDPNEERMPLVSFLVDGYPTVLYVRREEFTVDQGQ

AKKDGQGNEMTRSQLPLLLAWAMSIHKAQGQSIDRLRVDLRKIFERGQVYVALSRATNKE

HLEVINFDPRRITVAEEVLQFYSQISPVDVKREKSLAEVH

>tr|C4Y770|C4Y770_CLAL4 Formate dehydrogenase OS=Clavispora lusitaniae (strain ATCC 42720) OX=306902 GN=CLUG_04004 PE=3 SV=1

MSKPKVLLVLYEGKHHAKDEPKLYGCLENELGIRGFVESHGYELVSISEKDPIGDSEFDY

HLADAEIVITTPFFPAYLTRERIAEAPKLKLCITAGVGSDHVDLNAANERKITVAEVTGS

NVVSVAEHAVMTMLNLVRNFVPGHEQAMSGGWDIAAIAKDEYDLEDKVIATVGAGRIGYR

ILERLVAFNPKKLLYFDYQDLPKEAVDKLNAASKLFNGHDDIVERVEKLEDMLSRSDVVT

INCPLHEGSKGLFNKETISHMKDGAWLVNTARGAICVEKDVADAVESGKLRGYGGDVWYP

QPAPDHHPWRTFRNKYGGGNAMTPHVSGTSLDAQERYAAGTQAILKSYFEKSFDYRPQDV

IVVDGEYATKAYGQRK

>tr|C4Y771|C4Y771_CLAL4 Succinate dehydrogenase [ubiquinone] iron-sulfur subunit, mitochondrial OS=Clavispora lusitaniae (strain ATCC 42720) OX=306902 GN=CLUG_04005 PE=3 SV=1

MLKQMIRPMGRMSPMRTLATAASPKIKKFQIYRWNPDTPEVAPKMQTYEIDLNACGPMVL

DALLKIKNEQDSTLTFRRSCREGICGSCAMNIGGVNTLACLCKIDANTSKDTKIYPLPHM

YIVRDLVPDLTHFYKQYKSIQPYLQRDDHPADGRENLQSIEDRAKLDGLYECILCACCST

SCPSYWWNQQQYLGPAVLMQAYRWLIDSRDQASKFRKEMLQNSMSLYRCHTIMNCSRTCP

KGLNPGRAIAEIKKQLAFD

>tr|C4Y790|C4Y790_CLAL4 Sterol 3-beta-glucosyltransferase OS=Clavispora lusitaniae (strain ATCC 42720) OX=306902 GN=CLUG_04024 PE=3 SV=1

MQDNFKGTKLSALERRIMDSLKIEERHEQLSNVLPLYRRVTSALIPPLQYSIGFLHPHED

DSKEEGETEGEENKGEKKNPENLSSDEIDVACPLEEESASNSFLNALTTAVFYASAEPLK

SYRKGPPKEKEEQVYSVADETDQGVAAETLKEETNEQLQISEDEPKFGSVIDEKDIHDAS

SVSDSDISSDTSTLSEARRVLTGSGKVPDIDQMTPWEKSVFQKVNLLLNHDQGIFSYSTE

EKREEPDFEALPWYIANRLKKTFGLSDNDQFHGRFEAWLVRDVLLSGYIYLTSSSLCYYS

LLPGALSEEKDTKDDRKVEEGALGFKLGHYGDSYYNSVHKHLFWAVLTSSNLCLYTSSSE

MFFPVKVVDLKTASYCEVLTEPVGTLGQKSEQHDTKLSRSLSDSSLSQLESDASSIADSS

DENAESLQGGVWFRIVCDKKSYKFHTRNIFTARHWCNAITKQIFQLKNSNVHREVVLKIP

FDKILEFRKNFVLAEADEDIVLDNDTPVTFTAKYNTVIRKKGIDGEQQKNAIPQSGNVSY

DFVHFLLFNQGKDVWDAIEKLLAESQKLDVVTESDESTQGYENQGLENVPSIISTLEADL

ESSMSIIDKIARVNEDVYQLRERERYKFKNGVEEFAKLGKESPEKNNVVSKILSKPMKRK

GSSKIFCNSGSSGSTESSLNRILGEDGSGTDFLTDLVMDGHHLQFPRPFSVQTLKSLDLH

MSSSTKSRKELEKNYAKLVAKNKEDEDDVDPFLDEQDESSEITNESVYTNETGNTQDIKE

GRRGLKQRFKMLYSTSSIAPPRSDLRASSTESLSLKTDQDRESAMKKLRKYFHADPSEEL

MAIYKVYLRRSIPVYGRLFLGKEKLYFKSLLPGVSTKMILPLKNIAECRKRVGSVVKLAG

ISIVLHGREVICLEFRSKSARNECERLLSVQSKACQSTPKIERLQQEVELSTALQNSEDS

DELLEEIEKKEIEELAYRRVREARVRLLEDRLSTASGIEFPLIIGEDPIFFSEIKSSVSY

NFVLSTIGSRGDVQPYIALGKQLIVEGHNVTIATHGEFRDWIVSHGINFKEIAGNPSELM

SLMVRHGSMSVGFLKEASLKFRGWVGDLLSTSWKACQGADVLIESPSAMGGLHIAEALGI

PYMRAFTMPWSRTRAYPHAFIVPDQKRGGSYNLFTHVMFETVFWKGISGQVNKWRVETLG

IPRTNLARMRQTQIPFLYNMSPEIFPPSVDFPDWVKVTGYWFLNEGAGDYKPPDDLVTFL

KKARAENQKVVYIGFGSIVVKDAKSLTKAVVDAVLASGVRCVLNKGWSDKLNDEKAEKTK

IEVELPEEVFSCGSVPHDWLFKQVDAAVHHGGSGTTGATLRSGLPTIIKPFFGDQFFYAS

RVEDLGVGLALKKLNAKSLANALKSITTNTEFAMKAQAIAENMKQDTGVMNAVAAIYSEL

TYAKTLIRSIRLSNEQKRNSDDDLDSSAQLTGSESVDSDL

>tr|C4Y7D5|C4Y7D5_CLAL4 DNA replication licensing factor MCM3 OS=Clavispora lusitaniae (strain ATCC 42720) OX=306902 GN=CLUG_04113 PE=3 SV=1

MDDYDQPVDAVFGDRVRRFQEFLDQLNPAGSNYRAEIKDMLTKGRFRLCVSLDEIRDFDA

EFWRGLLDMPVDFLPACERALRDTVLTIYDPNDSRFQPLDDNQQFYISFRGAFGHHQVTP

RTISAGHLSKMVALEGIVTRASLVRPKVVRSVHYAEKTARFYAKSYRDNTTSFDAITTPA

VYPVEDPDGNRLTPEYGFSTYRDHQKIAVQEMPETAPAGQLPRSVDVVLDDDLVDLAKPG

DRVQIVGVYRALGGGSNNSSSFRTVVLANSVYPLHARSSGVAASEKLTDEDVRNINKLAK

ERRIFDILAQSLAPSIYGLDHIKKAVLLLLFGGVEKNLDNGSHLRGDINILMVGDPSTAK

SQMLRFVLNTAALAIATTGRGSSGVGLTAAVTSDKETGERRLEAGAMVLADRGVVCIDEF

DKMSDVDRVAIHEVMEQQTVTIAKAGIHTSLNARCSVIAAANPVFGQYDVHKDPHKNIAL

PDSLLSRFDLLFVVTDDVNQVKDRTVSEHVLRMHRFISPGMAEGEPVRERSAVSLAAGTE

VEEDAEQPVFEKFNSLLHAGVAGRAAKGAAPTLLSISFLKKYVQYAKQRIRPVLNKKASE

YIVATYTAFRNSDPADSYKRTAPVTARTLETLIRLATAHAKLRLSKTIEVRDAKVAEELL

RYALFKETAKTKPKRRRIAMFGEEDEDADLDDGVDEEAESDSEEITPPPDEEPVAEDAQQ

DDEIADEMDHLHLSRTAPLEASTHTNVFSAENLEGISAERYAVFLRVMANLMSSLLFENS

TGACPTEQVIESINEDLEQEEIFGPVEIESAFARMQDENKIMIGEGKVWKI

>tr|C4Y7F9|C4Y7F9_CLAL4 Adenylate kinase isoenzyme 6 homolog OS=Clavispora lusitaniae (strain ATCC 42720) OX=306902 GN=CLUG_04137 PE=3 SV=1

MVIICRTTHLISLITTSIMKSTRNHPNIIITGTPGCGKTSHAESLVSQLKGPYTHLNVSD

LAKERKCLESYDDELDTHVVDEDKLLDSLEIDLREGGAVVDWHCCDIFPERLIDLVVVLK

TDTDKLFDRLSKRGYKDNKIQENLDCEIMEVIWQDAAGAYIPEIVIPLASNSVEDMEENV

DRIAAWIENWVNDHPDGVTNEIDPEVLAAHQAQQEDDDETDE

>tr|C4Y7I2|C4Y7I2_CLAL4 Fatty acid synthase subunit alpha OS=Clavispora lusitaniae (strain ATCC 42720) OX=306902 GN=CLUG_04160 PE=3 SV=1

MRPEIEQELSHVLLTELLAYQFASPVRWIETQDVFLKEHNTERVVEIGPSPTLAGMASRT

IKAKYESYDAALSLQRQVLCYAKDAKEIYYTPDPADLAPPAKEEPAAAAAPAAAAPAAAA

PVAAAPAAPAAAPAGPAASISDEPVKASLLLHVLVAQKLKKPLEGVPMSKAIKDLVNGKS

TVQNEILGDLGKEFGSTPDKPEDTPLEELAEQFQDTFNGSLGKTSSSLIGRLMSSKMPGG

FSITVARKYLETRFGLGSGRQDSVLLMALVNEPPSRLGSDAEAKEFLDSVAQKYASSAGI

SLSSASAGSGGAAAGGAVIDSAALDALSAESKTLARQQLEVLARYLQVDLNKGAKSFIKE

KEASVLLQKELDLWEAEHGEFYAKGIKPIFSSLKARTYDSYWNWARQDVLSMYFDIIFGK

LTSVDRETINQCIQIMNRASPTLIKFMQYHIDHTPEYKGETYQLARRLGQQLIDNCKQVM

DVDPVYKDVSRITGPKTTVDSKGNIVYEEANKESVRKFEQYVYEMAQGGSMTKQVQPTIQ

EDLSRVYQAISKQATVSKNTKVELDKLYSQLVEFLEESKEIETAQATKTALQHQTSSSST

DEEETSASSDDEIASLPDKTSILQPVSSTIPPQTIPFLNLQKKGKNGWEFDHKLSSMYLD

GLESGAVNGLTFKDKHVLVTGAGAGSIGAEILQGLISGGAKVVVTTSRFSKKVTEYYQAM

YSRYGAAGSTLIVVPFNQGSKQDVDALISYIYDTKEGLGWDLDAIIPFAAIPENGNGLDN

IDSKSELAHRIMLTNLLRMLGAVKAKKTTDTRPAQVILPLSPNHGTFGFDGLYSESKISL

ETLFNRWYSEDWSTKLTICGAIIGWTRGTGLMSANNIIAEGIEKVGVRTFSQKEMAFNIL

GLLTPEVVNLCQEQPVMADLNGGLQFIDNLKEFTSKLRSDLVENADVRRAVSIENAIEQK

VVHGDNVDANYSKVSVQPRANMTFAFPDMKPYEEIKKISPDMENMLDLESVIVVTGFSEV

GPWGNARTRWEMEAYGEFSLEGCIEMAWIMGLIKYHNGNLKGKPYSGWVDAKTQQPVEDK

DIKAKYEEEILEHSGIRLIEPELFDGYDPKKKQMIQEVVIQHDLEPFEASKETAEQYKHQ

HGDKCEIFEIEESGEYTVKILKGATLFVPKALRFDRLVAGQIPTGWNAKNYGIPEDTISQ

VDPITLFVLVSTVEALLAAGITDPYEFYKYVHVSEVGNCSGSGMGGVSALRGMFKDRYSD

RPVQNDILQESFINTMSAWVNMLLLSSSGPIKTPVGACATAVESVDTGIETILSGKAKVC

IVGGYDDFQEEGSYEFANMNATSNAVDEFAHGRTPSEMSRPATTTRNGFMEAQGSGIQVI

MTADLAIKMGVPIYAVLAMTATATDKIGRSVPAPGKGILTTAREHHGDLKYPSAMMNVKY

RSRQLKKRLEQIGAWEASEIEYLSEEAELAKEEFGADFSVSEFMRERTEEIRREAKRQAA

DAKKQWGNFFYKNDPRIAPLRGSLAAFGLTIDDLGVASFHGTSTKANDKNESATINSMMK

HLGRSEGNPVFGVFQKYLTGHPKGAAGAWMLNGAIQILNSGIVPGNRNADNVDKVMEDFE

YVLYPSRSIQTDGIKAVSVTSFGFGQKGAQAVAVHPDYLFAVLDKNSYEDYASRVSSRNK

RAYRYMHNAITRNTMFVAKDKAPYADELEQPVYLDPLARVEESKTGLVFSKKGVQSNKSY

VSSISDATSKALSSLNKGSKGVGVDVELLSSLNLDNETFVERNFTSAEVEYASKSPFPQA

SFTGTWSAKEAVFKALGVKSQGAGASLKDIEIVRDSNGAPKVVLNGSAKEAAAKAGVKNV

SVSISHDDFQATAVALSEF

>tr|C4Y7I5|C4Y7I5_CLAL4 DNA replication licensing factor MCM7 OS=Clavispora lusitaniae (strain ATCC 42720) OX=306902 GN=MCM7 PE=3 SV=1

MATTVALPTVQINVNYAEIREHVTDFLTHYKTADSGIKYLQLLQDVANRDISTLYIELDD

IAAYQETIGIDLVAQIVKNAHHYLELFSSVVDDLMPEPTKDISYKDDVLDVILHQRRLRN

MRAAQESAEEMDQLRSGLSQAPESQSAPVAENVFPAQLTRRYHLYFKPLSAAKKALAVRE

VKGSHVGKYITVRGIVTRVSDVKPAVVVTAYTCDKCGYEVFQEVHSKVFQPLGECTSPVC

KTDNQRGQLFMSTRASRFSSFQEVKIQEMAAQVPVGHIPRTMALHVNGDLVRSMNPGDIV

DVAGIFLPSPYTGFRALRAGLLTETYLEVQHVRQHKKQYEQLEMSDEARERVMQLHAEGG

IYHRLAQSIAPEIYGHTDVKKMLLLLLCGGVTKEMGDGMRIRGDINVCLMGDPGVAKSQL

LKAINKIAPRSVYTTGRGSSGVGLTAAVMRDPVTDEMVLEGGALVLADNGICCIDEFDKM

EDGDRTAIHEVMEQQTISISKAGINTTLNARTSILAAANPLYGRYNPRLSPHENINLPAA

LLSRFDIMYLMLDQPSEAGDEQLARHVAYVHMHSHQPAMGFEPLDPQTIRHYISVARTFR

PVVPKEVGDYVVQSYISMRKESRRNEGSVRHFAPITPRTLLGVLRLSQALARIRFDNVVT

RNDVDEALRLIAQSKSSMEARDTEREDPTSSIMNHIRQFVVEAATPGVNVAELRARMLAK

GFTAEQLQSCIDEYSALGLFQLTDDGETLMVIGYDDMEM

>tr|C4Y7J4|C4Y7J4_CLAL4 Polyprenol reductase OS=Clavispora lusitaniae (strain ATCC 42720) OX=306902 GN=CLUG_04172 PE=3 SV=1

MLACILLHTSVFSQSGMLVCQAITAGFAAASALILFTKLYTPLNQLLAYGKVAQGPKSTQ

TGFARLVQIASRWTVPKQYFVHFYILFAVLQWLQLPWAKGKLLHTNFGLAWLLLTAQATR

RLMESLLLTQWGSKSRMHISHYFVGLYFYVCVAIVSFCGLVVAQEESKRPRQWLAIAFFA

LFSVDQYNNHRHLAALVKYSVPTFGMFRHVACAHYCDEIVIYFAVTVAAWTREPNMEVSV

ALFSAWVFVLVNLSVSGLESLRYYQTKFDDYTVQYAVVPFLL

>tr|C4Y7R6|C4Y7R6_CLAL4 6-phosphogluconate dehydrogenase, decarboxylating OS=Clavispora lusitaniae (strain ATCC 42720) OX=306902 GN=CLUG_04244 PE=3 SV=1

MGGAQGRLQILQKHLTNYASGDIGLIGLAVMGQNLILNAADKGFTVVAYNRTVSKVDEFM

NNEAKGKSIIGAHSIEELVANLKRPRRIILLVKAGKPVDAFIQQLLPHLEKGDIIIDGGN

SHFPDSNRRYEELKEKGILFVGSGVSGGEEGARHGPSLMPGGHPDAWPHIKDIFQSIAAK

SDGEPCCDWVGDAGAGHYVKMVHNGIEYGDMQLICEAYDLMKRVGKFSDKEIGDVFAKWN

NGVLDSFLIEITRDIMYFNDPTDGKPLVEKILDTAGQKGTGKWTAINALDLGMPVTLIGE

AVFSRCLSAIKDERVKASTVLTGPSVEGESPIKDKQKFVDDLEQALYASKIISYAQGFML

IREAAKEYGWKLNNPAIALMWRGGCIIRSVFLGEITAAYRENPDLENLLFHPFFNKAVIK

AQQGWRSTIGKAVEYGVPVPAFSTALSFYDGYRSAQLPANLLQAQRDYFGAHTFQVLPGQ

ENDFLKKDQWIHVNWTGRGGNISASTYDA

>tr|C4Y7S1|C4Y7S1_CLAL4 Phospholipid-transporting ATPase OS=Clavispora lusitaniae (strain ATCC 42720) OX=306902 GN=CLUG_04249 PE=3 SV=1

MREESESKGSASSADGNFKEERLDGSLKGSPSPVQKTYTVDDLRAAQPPTFKTKMQAYFF

RKGLADRPEYLQEIYSNMARKIYVNQPPPPQMLDEEGKPILNYPRNKIRTTKYTPLSFVP

KNLLFQFRNVANTYFLILVILGAFQVFGVASPGLAAVPLIVIVCITAIKDAFEDYKRGTS

DSELNNCPIHLLEGVHNYNVESDFVGPWRRFKKMCTRGLKRTNRFFKKAFISMFGSKTSK

TEFARKQAAQRNEALHRVSTVESEYSFHTRPSMTSKRSRKSHQSQRYRAPTKRHPNTLLN

PEVQKANLSNPEAKSPCFKNRKWKDISVGDFIRVRANEEIPADIVIISCSDIEGNCYVET

KNLDGETNLKTKSSLHCAGTSDLKHSVDVGNTKFWIECDAPNPHLYSFRGTIHYENYDEQ

GQLVNPDEREAITNDNVLLRGCTLRNTKWVIGVVVYTGTETKIVLNSGITPEKVSKISRE

LNLSVIINFVLLFILCFISGLINGLFYDKHNTSRVYFEFAAYSSTSAGNGVLSFFVALII

YQSLVPISLYISIEIIKTAQAFFIYSDVKMYYERLDFPCMPKSWSISDDLGQIEYIFSDK

TGTLTQNVMEFKKCTINGTSYGLAYTEAKQGMDKRQGIDIVKESERWNEAIKKDKADMVD

NLTNYVTNDQFREDALTFVSNKYVEDTVLPHTRNAEQKKANEDFMLALALCHTVVTEENP

TDHGLNDFKAESPDEAALVAVARDLGFVFRERLRKTLVLDIYGQRKEYQWLYTIPFTSAR

KRMSCILKTPEGKVLLITKGADNVIYERLASGTSDEILKKTALHLEDFAKEGLRTLCIAQ

KEIDEKEFDEWHERAKEANAVIDDSRDALIEDLNNEMERGLTLLGGTAIEDRLQQGVPDS

ISILSDAGIKLWVLTGDRIETAINIGFSCNLLGNDMKLLVVRPDENDPSNSQFVDDLLDE

YLNENFNIRTNTEEDIQQALTAARADHSVPMSNTALIIDGAALNIVFGDNPSLRQKFLLL

GKQCNSVICCRVSPAQKAQVVRVVKENLGVMTLAIGDGANDVAMIQAANVGVGIAGEEGR

QAVMSSDYAVGQFRYLTRLLLVHGRWSYKRLAEMVPCFFYKNVLFTMTCFWYGIYNDFDG

SYLFEYTFLMFYNLAFTSLPVIILAVFDQDVSDTISLIVPQLYRSGILGLEWSQFKFVWY

MFDGVYESVIAFFFPYLIYYRSFQNHEGLPVDHRFWMGVLVCAISVTACNTYVLLQQYRW

DWLTLLINALSTLVVFFWTGVWSVRAWVGEFYKAGAQLLGTLTFWCCFFVSVVACVLPRF

CHDFLKRSFAPKDIDIIREQVREGYYKDYPDGYDPTDVKDIERHRILQRLNEGDTELLEK

VEHVVEANAHESPSPDKDRKISRAFKSIKRHATLSRSRRGTKADKSRYNQQLLNNPIDLH

ELRLEMIRKGRVQYWC

>sp|C4Y7U0|GET3_CLAL4 ATPase GET3 OS=Clavispora lusitaniae (strain ATCC 42720) OX=306902 GN=GET3 PE=3 SV=1

MDFELEPTLESIIHQDSLKWIFVGGKGGVGKTTTSSSVAVQLALAYPNDEFLLISTDPAH

NLSDAFCQKFGKDARKVEGLSNLSCMEIDPDAAMSDLQTQAQQYNNDPNDPLKSMMSDMT

GSIPGIDEALSFMEVLKHIKNQRAADDGSESNAIQYKTIIFDTAPTGHTLRFLQLPATLE

KLLAKFKDLSGKFGPMLNMLGGGTNQQQDIFSKMNEIQKSVSEVNEQFTNPDMTTFICVC

ISEFLSLYETERMIQELMSYNMDVNSIVVNQLLFAEEDDCKRCQSRWKMQKKYLDQMGEL

YEDYHLVKMPLLGSEIRGVNNLKKFSKFLLKPYDPKVDKALVFELEEAK

>tr|C4Y857|C4Y857_CLAL4 NADPH-dependent diflavin oxidoreductase 1 OS=Clavispora lusitaniae (strain ATCC 42720) OX=306902 GN=TAH18 PE=3 SV=1

MTYSVDSVTILYGSETGNAQDYAHYLARKLRYHSLRPTLASLDSYDMKKLITETRFLIIL

CSTTGQGELPRNAKKFMRFLLKKKLPADLLSHVKCTTFGLGDSSYPKFNHAIKKIHTRLA

QLGCVDLSPRCESDELAPEGVDGFYAEWESALIEALHLHFPNAPQIDENVLLPPEYRLSL

DESKPDLCPVGDLGLTRPAALFRGKITKNTRVTAQDHFQDVRHVVIESADALSYSPGDTV

ALYPQNDEKSVELLLESQPHWLKIADKPLSLPHVDIEGGLISREKLTLRTLLTYHMDIQA

IPQRSFFALLHHFVDSSSEDGQRERDKLREFSQIENSEDLYNYAHRPRRSILETVMEFSQ

NLRIPVEYVLDLFPVIKVRLFSIASKPSPNLIEIVVAIVEYKTIIRRVRRGLCTKWLKSL

EPGSELVFSVHASNLQFSTRQNPSPPVVMVSPGTGVAPMKSLIESAAGSQELYLFYGCRY

RSKDFLFSDLWSSLEAKNFLHVYTAISREESKFKYVQDCMFGEKKLLSDLIVNKGAIFFL

CGSNGKMPTQVRLTLVEILRELVAEPEKYLIDMENEGRYIQETW

>tr|C4Y894|C4Y894_CLAL4 Eukaryotic translation initiation factor 3 subunit C OS=Clavispora lusitaniae (strain ATCC 42720) OX=306902 GN=NIP1 PE=3 SV=1

MSRFFASGYNSDSSSEEEDLLSTSEEELLSSSEEFSTDSEFENESEESSDDDDSDYNASG

PSYFLKKDFLKKGAHDSDSDSDGDGRKVVKSAKDKLLDDLREGVDALYNAKNLEIWSNVL

TEFDRLSRLVIRVGQQKLRVPNFYYKMLGQLDATIAEITAEKDKRKLPADQNRAFNTTRQ

RMKKMGPDFQAYLEYYRDKPEAFDAEEPVEIGIFGANDADDSSAVVGAVSGTTRLVSPLF

TTLRQIADSRGKKNIDKMEQVEILEKLLDEARESGRVFDIICVYQMLLSVRFDAGNSATM

PVSLWKDNQRDFESFLTFLEQNSKSYWVSEKGVSLDDWDVEPPANEDGVRVIMGSVAAVM

ERLDDEFAKGLQTTDPHSTDYIERLKDETALYRLLVRTQIYVQSTTGASEHLARVIVRRM

DHLYYKPDQLISACETAAWENAWPGIGIEDSSLMTKDKAPADVLAALSSFLSKQENAIYG

QHALLCSTYYYAVNSRYSDARELFLSSQMYNHIATADSNIQVLYNRALVQLGLSAFRAGE

IDESHQILNEIANSQRLKELLGQGFNSKYPNQATTAEKQKLLPFHMHINLELLECVFMTA

SLLIEIPALAAQSSTKDSRRKASIKSFKSKLEFHDRQYFTGPPESIKDHLIYASRYLQKG

NWAKSYDLLSSIKIWRLFPDHAQLLLMLKRQLQVEGLRTYIFTYKAIFSKLSLAKLASMF

QLDDENVAALVDKMLSSGEVQGSVENGFVNFVTDAPQRSKLQELAIVMNEKVGLLNEKNE

KTASNGHGRNKNAQQQQQQQQQKEQKEQKEYQQEENSKFRYANVNTNNDEFQVTA

>sp|C4Y8B4|KEX1_CLAL4 Pheromone-processing carboxypeptidase KEX1 OS=Clavispora lusitaniae (strain ATCC 42720) OX=306902 GN=KEX1 PE=3 SV=1

MIVRLFWLAVLWLALANANPLKRLNHVWSQRGVAKDDFLVTSLPGLSENIASDDVPLMFA

GQLELYPENNTHYFFWKFSDQKKEPEAANRTIFWLNGGPGCSSMDGALMEAGPLRIGKDY

KVQLNEGSWHRKGDVVFVDQPAGTGFSYSRDYDVELYQIEYHFLQFLKKYFELFPEDAHN

DIVLAGESYAGQYIPYIAHGILERNKKLADGESPYHLKGLAIGNGWISPNEQSLSFVPFA

VQAGLVSQKDPGWKAILQQHMKCQDLVAASHEDDTFGANSVVDKECEKVLNTILYELVDH

SASQYEQCINMYDYTLRDSFPSCGMNWPPDLSNVNHFLKSDEVMSSLNLVQQISWTECSE

HVGKQMKARHSKPAITLFADLLAEVEILLFHGNRDIICNYMGAESMIKKLHWGGQTGFSA

DSPVLKWFHGDEEAGYVKSERNLTFVNVFDASHMVPFDKPEVSSALVDILFKRFTVADDK

LQTQAKKTNANQNDSPSASENDNESGRTSESASSSPSESAATETESHATRIVRLIQLAVI

IILIWGLCAIYSTYRSKPTSIIKTKPSGRKKNVQWADLMPQEEPAPKGFLSKTLNKLSRT

EHKYVPVDIELGPTDGMDDASSDSGPSNVGTAETPEFVIASDDEENAHEHTEEH

>tr|C4Y8F3|C4Y8F3_CLAL4 Glutathione hydrolase OS=Clavispora lusitaniae (strain ATCC 42720) OX=306902 GN=CLUG_04481 PE=4 SV=1

MKDRVTRSVALAALILLGVYLFPRKMGDEVFAALAVVQPDPNYAPSFEPPAFSRPDYTMR

ASGRGPSLHPDSKLHAKGSRAMVSCDVPLCATMGKRILLAGGNAADAAVTVALCIGSVNS

HSSGIGGGGFIVSSRGNESITIDAREMAPARAHKHMYDGAPLRAQFGGLAVAVPGELAGL

EKLHALHGSGNLTWEQLFEPVVELNRRGWQASDVWVNAARTLHTLVLSPAPELGYNWDFI

YKPHSRRVVDVGDIVTRPNYANTLEAIARNGSSALFYDPEGPIAPLLAARARSSGGILEA

KDFAQYAVNVSPALRFDFAHNGSQYNVATAGGVSSGLALIAGLNFYSALEENGTTSDAVL

QTHRLVEAMKWTASARSHLGDVNSTYWAGSVARYTSREWARDLILTEQYADNRTFAWPHY

KPLYELTEPRGTSHFSVVDENGGAVGMTTTVNLLFGSSVYESTTGIVLNDEMDDFSLPNT

SNAFNLTPSALNFVSPYKRPLSSTAPTIIRRDGQLHLVIGAAGGSRIVTAVLQAIIRSIY

QNMPLIDTIAYPRLHHQLVPECIMAENSTMLDEEFGTAVVQNLHAMNHSFLDTGALTAMN

AIKRTEDGGWEGVADFWRKRGGADGY

>tr|C4Y8F7|C4Y8F7_CLAL4 alpha-1,2-Mannosidase OS=Clavispora lusitaniae (strain ATCC 42720) OX=306902 GN=CLUG_04485 PE=3 SV=1

MVFTCTRPRDPTRRKGLPPHASKSEHLAMNPFSKPHTNSGQSRWASNKQSLPLYKDKPSA

SKSAFSPARGSRSKRVMLFRVFVSTLALYIFYYFVTSDKSASLFQSSSASSWAEAKKEVR

DVFLESWAAYEADAWGKDVYHPVKGSGENMGPKPLGWMVVDSLDTLLIMECQEEFNRAKR

WVKEDLSYDFDYNVNVFETTIRMLGGLLSAYHLSNDDVFVDKAAKLANSLIGGFNSPTGI

PYSSVNLKTGEGIKNHVDNGASSTAEAATLQLEFRYLAKLTGEDLYWKAAEKIMEVLDKN

QPKDGLVPIFVQPDTGKYQGNLIRLGSRGDSYYEYLLKQHLQTNNKEPIYWQMYKESVDG

VKKHLVGKSKPNGLTFIGELEKGIGGPLSPKMDHLVCFYGGLLALGATNGLPLKEAKKSP

YWSQDKADDMKLAEELTYSCYKMYKDVETGLSPEIVVFNTDPSKDEDFTIKPADRHNLQR

PETVESLYYLYQITGDEKYREWGYEIFQSFQKYTRVLTADNKISYTSLNDVTTTKPSYRD

NMESFWLAETLKYLYLLFDDENKLPLNKYVFNTEAHPFPRFDMAPLFSTGWSRPIDDPRN

EHVYEKCVGTADETEKRPEPKIDKKNPPQAAPAAKKVDEEMKEEVKKDPSLKDDEGKKQR

KVEEALKDLQS

>tr|C4Y8L5|C4Y8L5_CLAL4 ATP-dependent RNA helicase DBP10 OS=Clavispora lusitaniae (strain ATCC 42720) OX=306902 GN=CLUG_04543 PE=3 SV=1

MRCPSPVNIDSLPINKMSDDEEYDIAGALSLVGKDVDSSSESDSGFSDFEDEEPQDVISD

NEEEAAPAKKKQKAVPSKETAFPSLELSDDEGSAEKDSKDIASYFAVNNPTAKKAKAGTF

ASFGLSKFILSNIAKKGYRQPTPIQRKTIPLIMENRDVVGMARTGSGKTAAFVLPVIEKL

KSHSAKVGARSVILSPSRELALQTFKQVKEFSKGTDLRSVVLIGGDSLEEQFSSMMSNPD

IIVATPGRFLHLQVEMQLDLSTVEYIVFDEADRLFEMGFAEQLNELLAILPTSRQSLLFS

ATLPRSLIDFAKAGLSNPVLVRLDAESKISDLLQMAFFSTKKAEREASLLCIISDVIKMP

LATQEEIAKIKRESKKADELSDDEDEKPEGDKKKRKKFKKERLPPANVLPSPHSTIVFVP

TKHHVEYVTGLLRDAGYLVSYIYGSLDQRARKEQLYRFRIGLTTLLVVTDVAARGIDIPI

LANVVNYTLPASSKIFIHRVGRTARAGNKGWAYSIVNEQELPYLLDLELFLGKKILVTSM

HEKKCELMKASKGDDYVEPKISYTDRLVLGSLPRIQLENSQEMYESLLRHNYDLKVIKDV

AAKGEKLYHRTRQAASTESVKRTKELVQTGLWDEQHLIFGPNLEKEKEKFLASLANRNIK

ETVFEYSKKGREKEEDSLVTLMQRRRRQIAPLQRKAKERRELLEKERIAGLSHTVQDEIL

KGDAGEVGYSVTAVEADEEELQEAFEDADELHAKRKEKRKSYKDPQFYLSHHAPAEAIQD

QQLALHSSFVNDAAKATFDLDNDDKSQKNSQVMKWDKKKGKYINMKSVEGKKYIIGESGQ

RIPASFRSEKFDEWRKKRNISSASSGMSETADSGKDNNKRFKHKKRDAPKAPDKFRDDYH

KQKQKVNKALESGINVRGFNKPGQRQELRSTEQIRKARQVKEQRKAKNARPSRKR

>sp|C4Y8M4|GUF1_CLAL4 Translation factor GUF1, mitochondrial OS=Clavispora lusitaniae (strain ATCC 42720) OX=306902 GN=GUF1 PE=3 SV=1

MLHIRSWTLPKSSCSTLIWNRGFSKAPVLQVAKVIAVQDKKINLDREKHIQRIQERIEKI

PISNYRNFSIVAHVDHGKSTLSDRLLELTGVIQPGAANKQVLDKLDVERERGITIKAQTC

TMFYHDTNTKEDYLLHLVDTPGHVDFRAEVSRSYASCGGALLLVDAAQGVQAQTVANFYL

AYSMGLKLIPIINKIDLDAADIPRAMDQVETTFELNREDCIPVSAKTGLNVENIIPSIIN

NIPPPQGDINKPLKALLVDSWHDPYVGVVMLVHIVDGKMKKGMKLLSAHTSKKYEVKEVG

VMYPDKTPMDVLRAGQVGYIIPGMKNPREALIGDTFFQNGQHENLEPLPGFEEPKPMVFV

GAFPADGGEFKVMNDHMENLVLNDRSVTLEKETSNALGLGWRLGFLGSLHASVFKERLEK

EYGAKIILTAPTVPYKIVYKDGTEKVITNPDEFPGADQRALNIDHLMEPYVKAIMTIPDE

YIGVVMSLCENNRGIQNEMEYLNTGQVLLKYELPLAQLVEDFFGKLKGATKGYASLDYED

SGYKKSDIVKMELCVSGIPQDALSQVMHRSQVQSRGKEYVARFKEYLKSQLFEVAIQAKV

NNKVVARETIKAKRKDVTQRLHAADISRRKKLLERQKEGKKQMKASGRVHINHEAYQAFL

RRSD

>tr|C4Y8V2|C4Y8V2_CLAL4 Deoxyhypusine hydroxylase OS=Clavispora lusitaniae (strain ATCC 42720) OX=306902 GN=LIA1 PE=3 SV=1

MADVDVDTASIEQLRDILCNKSGDIALANRFRALFNLKCIGADSSDEEIIHKAIDYIAEC

FGDSSELLKHEVAYVLGQIKNIHANKYLANVLEDKNQQIMVRHEAAEAMGAIGDSSSLNL

LEKYFKEDPDIEIRQTCELAIERIKWENSDSAGKEILEKSSFTSIDPAPPLPTDKESKVE

KLQKILNDQEKPLFERYRAMFRLRDLATDEACLALASGFEDPSALFKHEIAYVFGQLCNP

VTVPSLIKVLKDEREAGMVRHEAAEALGSIATEDCLPVLKSFLNDSVDVVRDSAVVALDM

YEYENSNELEYATVKTN

>tr|C4Y8W6|C4Y8W6_CLAL4 Alanine--tRNA ligase OS=Clavispora lusitaniae (strain ATCC 42720) OX=306902 GN=ALA1 PE=3 SV=1

MLKSLARKHNMTTSSHPWSASTVRSTFLDYFKNKSHTYVPSSSVVPHNDPTLLFANAGMN

QYKPIFLGTVDPTSEFATLKRAVNSQKCIRAGGKHNDLEDVGRDSYHHTFFEMLGNWSFG

DYFKKEAIAYSWELLTEVYKLDKDRLYVTYFEGDEKNGLEPDLEAKEYWLSVGVAPDHIL

PGDAHDNFWEMGDQGPCGPCSEIHYDRIGGRNAASLVNQDDPNVLEVWNIVFIQYNREAD

SSLKPLPSKHIDTGMGFERLVSVLQDKSSNYDTDVFQPIFEEIRNITGVRPYTGKFGAED

VDGIDTAYRVIADHVRTLTFAISDGGVPNNEGRGYVLRRILRRGARYVRKYMNCPIGSFF

QQLVDVVIENNSSIFPEIAKNKDMLKEILMEEEVSFAKTLDRGEKLFEQYAIIASKTPEQ

TLSGKDVWRLYDTYGFPVDLTRLMAEEAGLKIDEEAFEKAKEESREASKATNTKGGATLI

KLDVHALSELDNNESITKTNDVYKYGLDNIESKIVAIYDGSKFVDTISEAGQQFGIILDR

TPFYAEQGGQEYDTGSLVIDGSAEFAVTNVQSYGSYILHTGTMVEGTLKLNDNVIATYDE

LRRWPIRNNHTGTHVLNFALKEVLGDGIDQKGSLVAPEKLRFDFSHKQALTPKELEQVEN

ISSEIIKSNKQVYAEEVELTKAKQIAGVRAVFGETYPDPVRVVSIGVAVADLLKDPTNQD

WTKLSIEFCGGTHVAKTGDIKDFVIIEESGIAKGIRRIVAVTGHEAQEVQRIAREFSASL

DSADKLSFGPEKEQRAKELGVALKQLSISVLDKQKLNEKFTKLDKSIKDHLKAKQKEESK

KTLEAVNGWLKDEEKSKTAFFVAHVPISANAKAITEAINLIKKQHKDKSLYLLTGTSEKV

AHGCYISDEAISKGIDASELAKAVSAHIGGKAGGKGNVVQGMGDSPEGIDAAVETVTKLL

AEKL

>tr|C4Y926|C4Y926_CLAL4 Ribokinase OS=Clavispora lusitaniae (strain ATCC 42720) OX=306902 GN=RBK1 PE=3 SV=1

MITVIGSLNYDLVTYTDKVPEAGETFQANAFENHHGGKGLNEALACAKLRSSDKTVVKMI

GNVGSDTFGKELRHSLEQANVDTTFVKTLDNQSSGVAVILVESSGENRILITAGANGELK

PTEEDFRTYFKDSHAAGDFLVLQNEFPFTLQTIEWVKANKPSINIAYNPSPFYEELMTPE

LMSKIDLIILNEGEALSVATNVLSKSDITDFKSTIASNAVKGFSQLAEKLKSMIGKDNCN

TVIITMGSKGAIYTSHEDTAMFQPALQVTNIVDTTGAGDTFFGAVVSHLSEGKSLEKAIE

FATTASGLAIQKKGAAEGIPSYDEVKEASN

>tr|C4Y9B6|C4Y9B6_CLAL4 5-demethoxyubiquinone hydroxylase, mitochondrial OS=Clavispora lusitaniae (strain ATCC 42720) OX=306902 GN=COQ7 PE=3 SV=1

MSFPSSLRGSRCFSTALRTLQNASKKAPKEIDINTVEYGEMSKAQKAFLDRVIRVDQAGE

LGANYIYMGQYAVLANKYPHLKPVLKHMWEQEIHHHDTFNRLQTQRRVRPSLFTPFWKAG

AVAIGAGTALLSKEAAMACTVAVETVIGGHYNQQLRVLMNQYNIPVYDKATGKALSDEQL

TDAIKTSPELRDLKDLISTFRDDELEHLDTAVEHDARKAVPYMLLTETIKLVCRGAIWTA

ERV

>tr|C4Y9G6|C4Y9G6_CLAL4 CAAX prenyl protease OS=Clavispora lusitaniae (strain ATCC 42720) OX=306902 GN=CLUG_04855 PE=3 SV=1

MFPIPSLSFFSNNQALSFLDNSAFDWKSIVTSLLVGKYAFETYINYRQYQVYKRTQPPAS

IKQEITRETFLKSQEYSRATKRFGFFSDAVELVKDLATIKFDLLPRLWGWTGSLCVSLSK

ASVIGRFFGPGIMCQSLVFFAVTTLISTLESLPFSYYKTFVLEEKFGFNKSTLKVWITDS

IKSTFLSITLGTPVVYGFLKIIDYFGVSFVSYACAFVLVVQLVFMTIAPSLILPLFYKLT

PLEDGELKTAIEALAAKNKFPLSQLFVMDGSTRSAHSNAFFVGLPWSKKIVLFDTLIEHN

STEETVAVLAHEIGHWRLNHLPQMLLVSQASVAVTFILFSAFLTNKSLFHSFGFSSVYPP

FIAFTLFNYVSTPVNCLMQFANNLLVRKNEYQADAYAKEQGYTEELASSLIKLSTKNLSS

LNTDWLYSAYNDNHPILADRLSALGYVSKEKVGNVKVDIDELKQE

>sp|C4Y9H0|PFF1_CLAL4 Vacuolar membrane protease OS=Clavispora lusitaniae (strain ATCC 42720) OX=306902 GN=CLUG_04860 PE=3 SV=1

MRAAGCGGTGVAALTTKLSRSISQHQPKSMPQASVNSEQNPSVPNSPSAHKPARSQSAQS

APSRAKWFVRFFRSVFGYRKTSLTFLVALVFAATLLLSWADSSLDFSVDMPTSKHEQAVL

SRSWESLQKIARTKHTYTSEGNDEVHAYLEAHIASLVAKKPYMELDTDKNGTRRVMFDVK

YLSYDSVSYYESNNLVVRVNGSDSSLPALLVSAHYDSVPTSYGVTDDGMGVASMLGLLEH

YSSVAQPKRTIIFNFNNNEEFGLYGAQAFLAHPWFSQIAYFLNLEGTGAGGKAILFRGTD

YGIVRHFSSVRFPFASSLFQQGFNNRLIHSETDYSVYIKAGLRGLDLAFYKPRDIYHTTR

DSIQNTNIKSLWHMLSSSLDFVEHVSSQTIDLDEEVHAEAGKRDLALYTSFWNHFVVFSV

SQVVSANIALLVVVPVASLLLLFIIFRCNKGWGFNFVNAIKYPLSLVASVLVLTFVSQVI

IVPSNPFLVNSSIGLLVATLFSLFLLLNYIVLNGLNLVFKSFKGHQHDEKLIVMCESSFL

TWILLLWSTVKLSHNKFGDDHTGELFIPILFSLQAVACFLGFLGWCFKPSKKVKVSREEH

QPLLSSNGSNYGTQDDDDSLAPSSSLSLQSGFSENCEVHETKSFSYDWLVQFLVIVPISS

LIIFNSGSLILNGLNKSIQESLSAQNLIYKFIQIFVIVWSIPFLPFIFKLNRIIVLALSL

VLLYGFFAVNITDAFNDANPLKLRFLETISMDTSPPTNLVTVSARSMDVVKDILEDMPSL

KDSKTELSIDSLGDGMSLYSYETPLIPHLVPGVKNLTDYLSIDVLKDSSSVSDSPFGLLT

GELKINVPRNRNCKIDFNMSNTVIKVSDTKFMESKRSPVRTVIVYNEDKYENKSSSVIGA

GVPEGFSRDSKGNSVFKDMDGISQLQLNKLNWDKPYHIGFQWVPEIVESESVWSEKIRTK

KLGVNIECYWGDLDQLAEKDKAGNPVVQDRVPAFEELLHYSPSYVSWANRDRGMVSVTKY

IEV

>tr|C4Y9N2|C4Y9N2_CLAL4 Poly(A) polymerase OS=Clavispora lusitaniae (strain ATCC 42720) OX=306902 GN=CLUG_04922 PE=3 SV=1

MSTKTYGVTGPVSLANPTPNEIKLNDLLISELKARGSFESEQATKKRVEVLTLFQNMVQE

FVYTVSKNKNMSDGMARDAGGKVFTFGSYRLGVYGPGSDIDTLVVVPKHVGREDFFTVFE

QIIRKRPELEEITSVPDAFVPIIKIEFDGISIDLIYSRLNVPRVPLDMTLEDKNLLKNID

EKDLRSLNGTRVTDEILQLVPKPTVFKHALRCIKMWAQQRAVYGNVFGFPGGVAWAMLTA

RICQLYPNAVGATIVEKFFTIYTKWNWPQPVLLKPIEDGPLQVRVWNPRLYPHDRQHRMP

VITPAYPSMCATHNITSSTQKVIMKELERGAEIMSLVASGEKTWADLFERHSFFHDYKFY

LCIVAATLADDEEHHKWSGMIESKLRILVQKLEVTDGIALAHPYVKDFSNSYLLKDKKVD

EILNATGTLQGEHLLGTLESPTKPEKPPSEGDSTEKALHITRLYIGLDINLLKSEVGGVR

KLDIQHPCSEFYNLCKGWSTYDTEKHFIQIKNVKLLDLPNDVYVEGEVRPSKATKKRKKD

SKEAQLKRPRSVTTTPV

>tr|C4Y9Q9|C4Y9Q9_CLAL4 Histidine biosynthesis trifunctional protein OS=Clavispora lusitaniae (strain ATCC 42720) OX=306902 GN=CLUG_05130 PE=3 SV=1

MTQSVITLSHFKSHCKVESYFSSAMTFPILPLVSSAAQCELFAFAGQVLLPLSDFAVSKL

ALSAFPPSVKVNIDVSSEIVSCDEIVELLNVGAAQIFVAESQFELAVDAGLPSSRFAVVV

SKPTEALLKSKAAIVVSAPLDDVSIQAAQKNNRIVYFRGSLTQRTAEALAQNYVLVVPAQ

QLETAAKQSQETSQDKINVASVFVSTLTTDRPDGLYTTLVADTNGTALGVVYSSDASIAA

AIETQEGVFQSRKRPHELWYKGNTSGATQRLVKLERDCDCDVVKFVVASREGFGFCHVEE

NYTCFGDGNLAEGTDSYGSGLARLDATLAQRIKSAPEGSYTKRLFEDKDLLVAKMKEELD

ELIEAGASGNRAETAFEAADLFYFAMAYCVRSGVRLADIERSLDIKSMRVTRRKGDAKEK

YIAKTEEKTDAAEQTAKEKKPESQKTAAEEKENTYAMEVLRGDFTRALSRPVQATADIMK

LVLPIIEQVQRDGDKALLELTGKFDGVKLESPVLEAPFPSDLMQISEDMKHAIDLSMSNI

EKFHRAQMPAEAVMTVETSPGVFCSRFAKPIENVGLYVPGGTAVLPSTAMMLGVPAKVAG

CRNIVVASPPSRATGRLTPEVVYVAHRLGASKIVMAGGAQAVAAMAYGTESVVKCDKILG

PGNQFVTAAKMHVQNDTKALCSIDMPAGPSEVLVVADEKADADFVASDLLSQAEHGVDSQ

VILVGIALSDAKLREIEEAVRRQAEVLPRKDIVAKCLAHSYTLLVDSVEEAFKVSNDYAP

EHLILQLEDAEKYVPSHVENAGSVFVGGLSPESCGDYSSGTNHTLPTYGYARQYSGVNTA

TFQKFITAQKVTEQGLRSIGNAVMTLAAVEGLEAHRNAVQVRMDKLGLSS

>sp|C4Y9X0|DRE2_CLAL4 Fe-S cluster assembly protein DRE2 OS=Clavispora lusitaniae (strain ATCC 42720) OX=306902 GN=DRE2 PE=3 SV=1

MSEILLLLHPTVVTEESLVESAKSGLSGKFPEASLTQHIIDRIANDMVELPSSHFDHIHY

VNPNKSHLSIPPSVMAKLFASLKNGGELSGDLPKDQDLDVLMNGFIVKDDGSWSKPAPVS

TVLLRKKKPAETAARKMPTFKKPVSSPVTLTDTSANNTDAEDDLSMKRKLDSTKLAYFSD

DSSGEEDDLIDENELIADSHKFNVNIVVPKKCELPNGKKRKKACKDCTCGLKELEEQEEQ

ATRNLQDTLLGKMAQSATLEAIKIEERLKKSQVQFSAQDLTEIDFTVEGKTGGCSSCALG

DAFRCDGCPYLGLPPFKPGEVVTLDSFGEDI

>sp|C4YA37|LIPA_CLAL4 Lipoyl synthase, mitochondrial OS=Clavispora lusitaniae (strain ATCC 42720) OX=306902 GN=CLUG_04975 PE=3 SV=1

MVHPHLSRTKRTFFSHSSQMISRHIRKTNSLAFVRALSASETAVTPRRKRTVFTDELNKG

PSFEDFVSGKAADVFVDPLEAARKDPEAKLPKWLKVPIPKGRSFHSVKKDVRELKLATVC

EEAKCPNISECWGGKKSEATATIMLLGDTCTRGCRFCSVKTSRTPAKPDPMEPENTAEAI

SRWGLGYVVLTTVDRDDLADGGAAHLAETVRLIKQKAPQILVEVLGGDFRGDLTSCDTLA

VSGLDVYAHNLETVEALTPHVRDRRATYRQSLSILERAKQAKPSLITKTSLMLGFGETDE

QIMQTLRDLRGIGCDVVTFGQYMRPTKRHMKVVEYVRPEKFDYWKEVALEMGFLYVASGP

LVRSSYKAGEAFIENVLRKRKHNVGDSPRLA

>tr|C4YAD9|C4YAD9_CLAL4 GTP:AMP phosphotransferase, mitochondrial OS=Clavispora lusitaniae (strain ATCC 42720) OX=306902 GN=ADK2 PE=3 SV=1

MSLSRPIRMLLLGAPGSGKGTQTSRLLRAFPNIKPLSSGDTLRAEIASGSPIGSEASQYI

DRGELVPDKTMADLITTQLKSRHWLNPSASWLLDGFPRTRGQAETISPLLRESNANLNLV

VELDVDPSVILRRIEARWVHAASGRVYNLDYNPPKKPFVDDFTGESLVKRSDDTAEVFQK

RLDVYEKEIVPLREFYQQQGVLHTVSGDTSDEIFPKLKKIVEEVGNASS

>tr|C4YAE8|C4YAE8_CLAL4 Alpha-1,3/1,6-mannosyltransferase ALG2 OS=Clavispora lusitaniae (strain ATCC 42720) OX=306902 GN=CLUG_05086 PE=3 SV=1

MAKIAFVHPDLGIGGAERLVVDAAVGLQERGHHVTIYTSHCDMSHCFDEISSGVLAVKVF

GDFLPTTILGKFHILCAILRQLYLVLRLILSGEIRAYDFFIVDQLSFCVPLLNAFSTAAA

RVLFYCHFPDQLLAKKGGAVKKLYRVPFDAVEEWSTGTSDQIVVNSTFTKSIFHSTFTHL

SAVDPGVIYPCVDVLQPQQDNASAEQEWAQFMGHSRYFLSINRFERLKNVALAIRAYAKL

EKSARPRLVIAGGFDARVRENVEYLAELEKLCDSLGLVHYTFRGKLVAMPKSTEVVFLPS

VKNSVKNAALKTALLLLYTPTFEHFGIVPVESMLHKTPVLAIDRGGPLESIVNFTGENLD

NATGFNRPNDIDAWAEVLSEFSSSSKDNVRAKLGENGWHRANELFSRDQMSEQFEANLLT

AAKAPKKSLSVWPFAAATVVLVGMILSLWH

>tr|C4YAG5|C4YAG5_CLAL4 Folylpolyglutamate synthase OS=Clavispora lusitaniae (strain ATCC 42720) OX=306902 GN=CLUG_05103 PE=3 SV=1

MTQPEMRVRTYKDAIAALNSLQSNFASIEALKRQPISPLARSELSLAEVHEYVRRMGYQR

SDFNKMNVIHVAGTKGKGSTCAFVESIMQQYRDQGIRKVGLFTSPHLKSVRERIRINGAP

IAPQKFTRYFFEVWDKLSATTSDAEAFPTLQPCHEVKPMYFKYLTLLSLHVFMSEGVDTA

IYEVGVGGRFDSTNVVDRPTVAGITALGIDHTFMLGSTIEEIAWNKAGILKQGCPAVAAR

QTEYPQAESVIEQEAEKAQVSSLEFVGSEVVPEDVPLGLAGTFQRQNAAVAVKLAEHHLK

HLGISVDLADGLPDEFMAGLKHVRWDGRCQTLEKEGYENITWYIDGAHTLESVRVAAEWY

SSAVSKRNTTKILLFNQQSRENATELLAELYERTPHFDHAIFTTNVTWADGTYNSDLVSM

NYSKETVDRLVVQKDLAEAWSTLDQKHNANSRKHVFPDIETGLRFIKSLASERSVEVFVC

GSLHLVGGVLAVLDGEKD

>tr|C4YAM7|C4YAM7_CLAL4 Transcription and mRNA export factor SUS1 OS=Clavispora lusitaniae (strain ATCC 42720) OX=306902 GN=SUS1 PE=3 SV=1

MSDELGAIKSQIQDHLVSSGNYDVISKQLKLRLYESGWFDSVTQAASAELEKQHAEPLNF

ERLYETLRPQAEKMVPPQVREDVMAKIREYLDSTIE

>tr|C4YAP2|C4YAP2_CLAL4 Phospholipid-transporting ATPase OS=Clavispora lusitaniae (strain ATCC 42720) OX=306902 GN=CLUG_05270 PE=3 SV=1

MAQDSNNPFADKNNLIDLDLNEYPTTEHPGERYPVYDSPYTYPTSSTRAGENNANPFSDT

LELSDLEDDSFYGQGSSHGLHHEGSNRQVPLLSTSNEPQQEKAPGFFQNLKAGRKGNASG

AYIGMVDDYTDHMKDTNTGSSDFDIRKIFNRVKGVFRRKNVEDATAALGPRHIYVMDRAK

NSSFGFYGNHISTTKYNFATFLPKFLFEQFSKYANLFFLFTSIIQQVPDVSPTNRYTTIG

TLTVVLLVSATKEVMEDIKRANADKELNNTSVLVLDPETGEFHSKKWISVQVGDIVRVNN

EESFPADLLLLSSSEPEGLCYIETANLDGETNLKIKQAKSETAYLVDPHSLVSDLSHTEI

MSEQPNSSLYTYEGTLNNFGPSSKLPLSPQQLLLRGATLRNTQWIHGIVVFTGHETKLMR

NATAAPIKRTDVERIINLQIIALFSILIILALVSSIGNVAQIQINKKHMPYLYLEGTNMA

KLFFKDILTFWILYSNLVPISLFVTVEIIKYYQAYMIGSDLDMYYAESDTPTGVRTSSLV

EELGQIDYIFSDKTGTLTRNVMEFKACTIGGKCYAEEIPEDGQAQMVDGIEIGFYSFNDL

QAHLRDNLSQQSAIINEFFVLLSTCHTVIPEVNEATGAIKYQAASPDEGALVQGAADLGY

KFTIRRPKSVTIHANATDTDAEYELLNICEFNSTRKRMSAIFRCPDGMIRLFCKGADTVI

LKRLSELEPQPFVSATIRHLEDFASDGLRTLCIASRIVPEEEYQAWATQYYEASTALENR

SEQLDEVAELIEKDLFLLGATAIEDKLQDGVPETIHTLQNAGIKIWILTGDRQETAINIG

MSCKLLSEDMNLLIINEETKADTKLNLKEKLDAISEHQHDMDASVLDSSLALIIDGHSLG

FALESDLEDLFLSLATRCKAVICCRVSPLQKALVVKMVKRKKKRSLLLAIGDGANDVSMI

QAAHVGVGINGMEGMQAARSADVSIGQFKYLKKLLLVHGSWSYQRISNAILYSFYKNVAL

YMTQFWFVFLNGFSGQSLIESWTLTFYNVLFTVFPPFIMGVFDQFVSARFLDRYPQLYQL

GKPRKFFNVTTFWEWIVNGFFPLGFDFRGYDGHDSQLVVPI

>tr|C4YAW7|C4YAW7_CLAL4 Adenylosuccinate synthetase OS=Clavispora lusitaniae (strain ATCC 42720) OX=306902 GN=CLUG_05432 PE=3 SV=1

MLPSGLVNPKCLNLVGSGVVIHVPSFFEELENLEKKGLQGRDRLFISSRAHLVFDFHQRT

DKLKEAELSENKKSIGTTGKGIGPTYSTKASRSGIRVHHLVSEEPEAWEEFKNRYYRLVD

TRMKRYGDFDYDAEAELARYAKYRELLRPFVVDSVPFMHKAIAEKKKILVEGANALMLDL

DFGTYPYVTSSATGIGGVLTGLGVPPKAIRNVYGVVKAYTTRVGEGPFPTEQLNEVGETL

QNVGAEYGVTTGRKRRCGWLDLVVLQYSNNINGYTHLNITKLDVLDSFKEIKVAVAYHYK

GQKLDSFPDDLVKLGKVDVEYVTLPGWEQDISKITKYEDLPENAKKYLDFIENFLNVPVK

WVGVGPARESMLVKP

>tr|C4YAX6|C4YAX6_CLAL4 UDP-N-acetylglucosamine--dolichyl-phosphate N-acetylglucosaminephosphotransferase OS=Clavispora lusitaniae (strain ATCC 42720) OX=306902 GN=CLUG_05441 PE=3 SV=1

MRLLEVSALVAALALARANSPLQTAVAFGLIGFSATSHLVPRLGASFLRVGLSGRDLSKK

PPVAPIPESMGVVPAVTYMVLLVTIIPFVFFKYLVSFQALSDDSDISAVYENQYHAVQHN

NNMFPHNKLAEFLSGALCLLSTVLLGFFDDLFDIRWRHKFFLPAVASLPLLIVYYVDFSV

TSIVVPPFVSRVPGGDAAVDMLRILINAINRAVSAVTGLRFTTLATDYVSSEQPPQLLDL

GVCYYAYMSALSIFAPNSINILAGINGLEVGQSIVLGVIFLLNDLCYLLSASSPAAHDSH

LLSAIFVIPFLGVSAGLWQYNWYPAQVFVGDTYCYFSGMVFAVVGILGHFSKTLLIFLLP

QIINFLYSVPQLFHIVPCPRHRMPKFNVDDGLMYPSFGELKQATAVGTALVSVLAKCGLI

QVKRNSDNQITHFSNMTIINLALVWLGPMREDRLCLVILAFQLVVGLSMIVVRHTVGPWL

FGYDNLSWGVK

>tr|C4YB52|C4YB52_CLAL4 Very-long-chain 3-oxoacyl-CoA reductase OS=Clavispora lusitaniae (strain ATCC 42720) OX=306902 GN=CLUG_05344 PE=3 SV=1

MSVLQLISEVSDSKAAQVLLYAALITGVYKITTFSLALAALLADAFVLPPTNFAKYGAKS

GKWAVVTGASDGIGKEYALQLAAKGLNVVLVSRTLAKLESLASEIEEKHKVQTAIVAFDA

SEDKEENYRQLRETIADLAVTVLVNNVGQSHSIPVPFLETDPKELTDIVTINNLVTLKIT

QTVVPVISKTVKSDRKARGLVLTMGSFGGLLPTPYLATYSGSKAFLQQWSAALAGELKPE

GIDVELVISYLVTSAMSKIRRTSATIPNAKQFVRATLKSVGKRVGAQDRYATSTPYWSHA

LMHFFIENTVGVYSSVANKLNFDMHKSIRVRALKKAARAKKE

>tr|C4YB90|C4YB90_CLAL4 Chromatin modification-related protein OS=Clavispora lusitaniae (strain ATCC 42720) OX=306902 GN=CLUG_05382 PE=3 SV=1

MDTTAVLDKYTQNLSNLPLEVKHLFEELKAKDAQLAEARKRYQTKDSQLHKFIRANGTLT

KHPKEQQLYQKIEEDMKLVQQIQKEKILIANTALFLVSKHMYNFSVDIQKLEREDLLPPL

DADALDGSDSESSATPKPMPEAIRKKRTYSVMKRQKRPKSEDYDYGDEPAAISSRSANPA

RPNGDDADNNLYCFCQRVSFGEMIGCDNDDCKFEWFHWSCVGITSPPKDDEVWYCPDCAP

KMEKRKKRKK

>tr|C4YBA9|C4YBA9_CLAL4 Adenylate kinase OS=Clavispora lusitaniae (strain ATCC 42720) OX=306902 GN=ADK1 PE=3 SV=1

MSVEDLKSVVEKLQDRIQTLEKKAGLVPNVPKSIRMVLIGPPGAGKGTQAPNLKEKFCAC

HLATGDMLRAQVAAKTELGVEAKKIMDKGGLVSDEIMVNMIKSELENNKECKNGFILDGF

PRTIPQAEKLDGMLSERKTPLQSAVELKIDDELLVSRITGRLVHPASGRSYHKLFNPPKK

DMTDDITGEPLVQRSDDNEDALKKRLVTYHKQTEPIVEYYKKAGIWSGIDASQKPAKVWD

DILKCLGQK

>tr|C4YBD9|C4YBD9_CLAL4 GMP synthase [glutamine-hydrolyzing] OS=Clavispora lusitaniae (strain ATCC 42720) OX=306902 GN=CLUG_05517 PE=3 SV=1

MSTEDVPIEVSKVFDTILVLDFGSQYSHLITRRLREFNVYAEMLPCTQKISELSWTPKGI

ILSGGPYSVYADDAPHVDHDIFKLNVPILGICYGMQELAWINGKGVGRGDKREYGPATLN

VEDKDCPLFKGVDHSQVWMSHGDKLHALPTGFKTVATSDNSPFCAIAHEKEHIYGIQFHP

EVTHSVNGKVLLKNFAVDICQANTNWSMENFIDTEIARIRKLVGPTAEVIGAVSGGVDST

VGAKIMQEAIGNRFHAIYVDNGVMRLNETEQVYKTLTEGLGINLTVVDASDLFLGRLKGV

TDPEKKRKIIGNTFIHVFEDEAAKIKPASGQEIEYLLQGTLYPDVIESISFKGPSQTIKT

HHNVGGLLDNMKLKLIEPLRELFKDEVRHLGELLGVPHDLVWRHPFPGPGLAIRVLGEVT

KEQVKIAREADAIFIEEIKKAGLYKEISQAFAALLPVKSVGVMGDQRTYEQVIALRAIET

VDFMTADWYVFEASFLKRVASRIVNEVDGVARVTYDITSKPPATVEWE

>tr|C4YBQ4|C4YBQ4_CLAL4 RNA cytidine acetyltransferase OS=Clavispora lusitaniae (strain ATCC 42720) OX=306902 GN=NAT10 PE=3 SV=1

MGKKAIDSRIPALIRNGVQEKQRSFFIIVGDRARNQLPNLHYLMMSADLKMNKSVLWAYK

KQLLGFTSHRKKRENKIKKEIKRGTRDANDQDPFEAFISNQHIRYVYYKETEKILGNTYG

MCILQDFEALTPNLLARTVETVEGGGLVVILLKSMTSLKQLYTMTMDVHSRYRTEAHGDV

VARFNERFLLSLGSCATCLVVDDELNVLPVSGGKNVVALPPPEDETQTELDSLKESLAEV

QPAGSLVALSKTVNQAQAILTFVDVISEKSLRSTVALTAGRGRGKSAALGIAIAAAIAHG

YSNIFVTSPSPENLKTLFEFVFRGFDALGYAEHADYDAVQSTNPSFNKAVVRVDVKRSHR

QTVQYIAPTDHHLLGQAELVIVDEAAAIPLPVVRRLLGPYLVFMASTINGYEGTGRSLSL

KLIQQLREKSAQADSSDTGRTLREVVLDEPIRYAPGDPVEKWLNKLLCLDASVSKTSRGT

PHPDECTLFRVDRDTLFSHHPVSEAFLHRMMALYVSSHYKNSPNDLQLMSDAPAHQLYVL

LPPQKQGDRRIPDPLCVVQLALEGQISKESVRAQLARGQRAGGDLIPWLVAQQFQDDEFA

SLSGARVVRIATNPDYAGMGYGSRALSLLKDYFEGKLTDISENDVVEKSVARVSDAELAG

ASLQDEIRSRDAASLPPLLLQLSETPPCHLHYMGVSYGLTPSLHRFWKRAGFVPVYLRQT

ANALTGEHTCVMLHTLGGEAAWLTAFARDFRRRFISLLAYDFRKFPASQALAVLEAAGDA

GAAKLARAEVDAHFSPFDLKRLDSYASNLVDYHVILDMLPQMAQWQFAGRTDVSLSSVQS

AILMAVGLQHKDMDTVSAELGLPAAQSMAMFAKIIRKFSTFFRSVLSRDIESQMPQLEDE

AVREMEGETEHIDAAAVELQMTKDLDAEGADAVQALRQKELISALNLEKYAIEDMGDLDE

KEAKKAAARNGVVSVKTKKPKRKTESAEQIYETEMKSTKKQKKKKH

>tr|C4YBR9|C4YBR9_CLAL4 DNA replication licensing factor MCM2 OS=Clavispora lusitaniae (strain ATCC 42720) OX=306902 GN=CLUG_05647 PE=4 SV=1

MRSACTSHVIYPFGQRVFFAYPSHSSQFQLQPVLSLFHLVNFPADFSSFLDFSTLLYFLQ

FLQFPDFSSMSSPRKRQAEADEDLQSDRSTPSLASSPQLPPSSPAIPFEEEEEEIHNDVT

DLNPSDEEEGEDLMENLENDYRRNEQQDTYDLGDGNIDDAEYEEMDAATRRRIDEQLDRR

DELLGTTRTARGREQALLDDDDLGDVNEFGLPVQRRRRRRQYDNDDDMFMDDADIDPFHE

ELSLESLADVKAQSITEWILQPAVSRSIARELKSFLLEYTDEKGRSVYGARIRTLGEVNA

ESLDVSYGHLADSKAILALFLASSPAEMLKIFDIVAMEATELHYPNYSQIHQEIHVRISD

FPNHLSLRDLREKNLNQLVKITGVVTRRTGVFPQLKYVKFDCLKCGVVLGPFIQDSNTEV

KVSFCTNCHAKGPFKLNSEKTLYRNYQRITLQEAPGSVPAGRLPRHREVILLSDLVDVAK

PGEEVEIVGIYKNNYDGRLNAKNGFPVFATIVEANSVKRKETSSVFNSDTGQAAWVEEDE

REFRRLSRERGIIDKIIASMAPSIYGHKDIKTAIACSLFGGVAKNVNGKHSIRGDINVLL

LGDPGTAKSQILKYAEKTANRAVFATGQGASAVGLTASVRRDPITREWTLEGGALVLADK

GTCLIDEFDKMNDQDRTSIHEAMEQQSISVSKAGIVTSLQARCSIIAAANPIGGKYNSTL

PLSQNVNLTEPILSRFDILCVVRDVVNPESDERLATFVIDSHMRSHPANDDDVFEESDEQ

LEPHSRREIVMEKTKQRESEISPIPQDVLAKYISYARTKISPKLHQMDMDKVARVYADLR

RESISTGSFPITVRHLESIIRIAEAFARMRLSDFVSQGDLNRAIKVSIDSFVGAQKVTVR

KQLQKSFMKYTLPSWQ

>sp|C4YBT3|MDM12_CLAL4 Mitochondrial distribution and morphology protein 12 OS=Clavispora lusitaniae (strain ATCC 42720) OX=306902 GN=MDM12 PE=3 SV=1

MSFDINWDKLVSDDSISNSIKDFLNDQFNEISLPSFVDTLSVTGFSLGSIPPNITIRHIG

NPFDEFYKRNVPEEEAKKGQLPVSPELSGSDSDSSEEEPAINNLDSTNQQDFTPLLPEVD

SLRRQNQESKHFNNFNMNNVGLGHMDLDTPTNFFNPHAYRLHSNSVGRNESVKKNDNDIQ

FILEIDYKGDVALEIVVNLLVNYPSAHFITLPIKLKITDLVIHSLAAVAYLENSVFISFL

CDLNDADSDYFTSASMHRRSDAHSRGSHIPPSSGGNITDYVPSNNKERIDVIRSVKIDTE

IGEMENNVLRNVGKVEKFLVEQLRNIIRDETAWPSWICLDFNDDDDDDDDDDDQPSTAST

QHSSHTS

>tr|C4YBZ5|C4YBZ5_CLAL4 Double-strand break repair protein OS=Clavispora lusitaniae (strain ATCC 42720) OX=306902 GN=CLUG_05723 PE=3 SV=1

MPPIASIPAGPNRLRILVTSDNHVGYLENDPVRGDDSWKTFQETMRLAQIHDADMVVQGG

DMFHVTRPSKKALFHVIQALRLNCLGDRPCELELLSDPALALRSGDSLNYEDPNLNVAVP

VFAISGNHDDATGSGLLSPLDVVAATGLVNYFGQIPRDDKISLAPILLQKGTTRLALYGL

NNLRDERLQRLMRDGKVTFQKPRERFFSILCLHQNHARHSISSYVPEDFLPSFLDLVIWG

HEHECILDPQYNPATGFDTLQPGSTVATSLSEGETAPKHVFLVDVLGEEYSIKPIRLQTV

RPFVMRDISLQREHFVEGPASKRDIADFLVSQVEEMIEQAKHDEENEEQDETHEKTSSEN

MLPLIRLRVDHTGDYEVENARRFSNRFVGRVANVNDILLYHQKKASRQPQRKLAEPQARE

PSNVSIQQLLQSVLGDSSLFLVAEDRIFDATKKFIEQDDKEVLAEYVEKAVEDATKSLLQ

IGIDEAEFHTGDTRKGFRQLLNQLRREAKRKPIDDESWQDSPKESVPEPEPVVLSEEDEP

KKRAPRAKAAPKKATTAKPKAAPRRAKRTHDQSLLDDILSLGS

>sp|C4YC37|MTNA_CLAL4 Methylthioribose-1-phosphate isomerase OS=Clavispora lusitaniae (strain ATCC 42720) OX=306902 GN=MRI1 PE=3 SV=1

MSKDTLEAIRFDKENVTLDILDQLLLPYESRYINIKSIQDAFEAIKSMQVRGAPAIAIVG

AFAITVDTHLYLKSGETSKTVADLLNSIDYLVTSRPTAVNLANACNEIKALLVSHFEKSD

LVTEKVWKLLFDYSVSLHEDDLRNNFKIGENGLRFISETLKAQNFKGPFSIVTVCNTGSL

ATSGHGTALGVIRTVHAQLSKSVSNEEFWFEHVYPLETRPYNQGAKLTTYELHYEKIPFT

MICDNMVTSLISTLHKKKNIKGSAAPVKFIITGADRVVKNGDSANKIGTYQLAAIADFFN

STFTKEEDKIKFMVAAPNTTIDLKTETGDEIVIEERPAHELTSLKGPVLREDGSVGEKMT

VGIATPGIQVWNPAFDVAPYQLIDCIVTEDEPFKKVDGKFSF

>tr|C4YC54|C4YC54_CLAL4 ATP-grasp domain-containing protein OS=Clavispora lusitaniae (strain ATCC 42720) OX=306902 GN=CLUG_05871 PE=3 SV=1

MSLNILVVGNGGREHAITWRLAQSSLVNKIFVAPGNGGTSTLGDKVVNVPHLSSSAKDFS

KLQQFALENEVGLVVPGPEQPLVDGISTVFTKVGIPVFGPSAKAAQMEGSKAFSKVFMDK

HNIPTARFQNFTSVEEARKHIETVDYRIVLKADGIAAGKGVLIPQSKEEALQGLDEIMVA

KNFGSAGDEIVIEEFLEGDELSILTITDGYSFFNLPAAQDHKRIGDGDSGLNTGGMGAYA

PAPIATPAVLAKINDQVIRPTIDGMRKDGFPMCGVLFTGIMLTPSGEPKVLEYNVRFGDP

ETQTVLPLLSEETDLAAVFLAAAEHRLDSVDIATKPDTFSTTVVMAAGGYPEAYEKGDEI

TITAPPADTYIFHAGTAEKEGKVVTSGGRVIAATATAPTLRESVDKAYVGVDHVSFKNKY

NRKDIAHRAFRAAEKPSGTTYAEAGVSVDNGNLLVEQIKAKVKSTRRAGADSDIGGFGGL

FDLKAAGFAGEDTLLVAATDGVGTKLRIAQIMDIHDTVGIDLVAMNVNDLVVQGAEPLIF

LDYFATGKLDVNIAAKFVSGVADGCITAGCALVGGETSEMPGMYDAGHYDTNGTAVGAVS

KSGILPKIEEMRAGNVLVGLRSDGVHSNGFSLVRHIVDKCGLDYSAPAPWTDSGRSIGEE

LLVPTKIYVKQLLPAIRRGLLLGMAHITGGGLVENIPRALPKHLQAQVDMSTWEVPAIFK

WFGKQGGVPYEDLLKTFNNGIGMVVIIAPENVDEALAEFRKAGEDPVVLGKLAERAEGEP

GCVVHNIGDLY

>sp|C4YCC3|GET2_CLAL4 Golgi to ER traffic protein 2 OS=Clavispora lusitaniae (strain ATCC 42720) OX=306902 GN=GET2 PE=3 SV=1

MSELSAEEKRKLLRERRQAKMAQGKATDRLNNILSQGSSVKSSNVTSVLDKPEKATTTVM

DLPSRETQSPTPLHDDPEVPDITSLLKEKENEAPDMEAMLQQILGGSGAHTGPGNDGGAN

FLQEMMKAMAEDPSGGSTAEESSYQSQLSQYHAYEQKQWKARFLVVRWIIHTLNFVYHYI

ASGYKLSASPYAFVRAQAVDSHVRTFFTAFLTVEVAVISAYFLVMSQPKFKDFSRENLVS

RILSMASAVVPAVGRYQPLVTRALVYWNGASIFVGDLMLMVFYFGITSVLGN

>sp|C4YCH0|3DHQ_CLAL4 Catabolic 3-dehydroquinase OS=Clavispora lusitaniae (strain ATCC 42720) OX=306902 GN=DQD1 PE=3 SV=1

MVKKVLLINGPNLNLLGTREPEKYGTTTLKDIEMEAHQQVAKHQDAELFTYQNNTEGFII

DRIQEAKQQGVGFIIINAGAYTHTSVGIRDALLGTAIPFIEVHITNVHQREPFRHHSYLS

DKAIAVIAGLGVYGYTAAIEYALNY

>tr|C4XVN0|C4XVN0_CLAL4 Vacuolar protein 8 OS=Clavispora lusitaniae (strain ATCC 42720) OX=306902 GN=CLUG_00049 PE=3 SV=1

MGACCSCLSSSNSDGKYPPLLLAENEREAISALLQYLENRSDVDFFSNGPLRSLSTLVYS

ENIDLQRSAALAFAEITEKDVREVNRDVLEPILILLQSNDTEVQRAACGALGNLAVNNEN

KALIAEMGGIEPLIRQMMSPNIEVQCNAVGCVTNLATQDENKTKIAKSGALIPLTKLAKS

KDIRVQRNATGALLNMTHSFENRQELVNAGAVPVLVSLLSSDDADVQYYCTTALSNIAVD

EENRKKLSATEPKLVGQLVSLMDSPSPRVQCQATLALRNLASDSTYQVEIVRAGGLPHLV

QLLTCNHQPLVLAAVACIRNISIHPLNEALIVEAGFLKPLVALLDYTDSEEIQCHAISTL

RNLAASSERNRLALMNAGAVEKCKELVLRAPLSVQSEISACFAILALADDLKPKLYESHI

IDYLIPLTFSENGEVCGNSAAALANLCSRVSSEHEDYILDNWTQPSEGIYGFLIRFLRSG

SATFEHIALWTILQLLESNNHEIQSLIKENESILSGIKNLSESQQQGSIVPNGADSNEYE

DPKVELFNLTQQILQILG

>tr|C4XVN2|C4XVN2_CLAL4 Uncharacterized protein OS=Clavispora lusitaniae (strain ATCC 42720) OX=306902 GN=NBP35 PE=3 SV=1

MTPSALPLKAPEPEHCPGPESENAGQGDACAGCPNQSICESLPKGPDPDMPIITAKMQNI

DHKILVLSGKGGVGKSTFTSMLSWALAADPDLEVGAMDLDICGPSLPRMLGAEGESIHQS

NSGWSPVYVSDNLGLMSISFMLPDPDAAIIWRGAKKNGLIKQFMKDVNWGEHLDYLIVDT

PPGTSDEHLSVTTYMKDAGIDGALIVTTPQEVALLDVRKEIDFCRKANIKVLGLVENMSG

FVCPNCKGESQIFKPTTGGGEQLCKDMNIPFLGSVPLDPRIGRSCDSGECFFDEYADSPA

SEAILAVVDALRDSVEIPIDKLAIK

>tr|C4XVN3|C4XVN3_CLAL4 Peptidase S59 domain-containing protein OS=Clavispora lusitaniae (strain ATCC 42720) OX=306902 GN=CLUG_00052 PE=3 SV=1

MSAGTSWGNSTGFGANTSNSGGAQPISGGLFGNSSSAFPTFGAKSQGASLPANTQQNANT

GLFGNSSNNASTTLPGSTSSGLFGSSANNTSNNGGLFGNTGSKPTGGLFGNTNSSSAAAG

GGLFGNTNSSSSTTGGGLFGTSASNPTSNTGTSNTSGGLFGNSNKPATGGLFGSSNSTAN

TGGLFGHPNTTATGNSNSTAAGGLFGNSNSTATTGGLFGASTNPLNKPSTGGLFGSSNTG

GMFGASGNTGGLFGASNTSNTNAQGPNIASTAYTPNPYSSDSILSSINATENNMPLSITG

SLFAKPSSIQHRQSASQPPKKTYQSSLLKRLAQTFKIFRSTPDSSIEDLGKSHLKGIFTQ

QNFVGGYSNSNVDTSSYSVSKNHKKTSTLPVANVSVGDVKKLVIKSKPLKFHLINADKVF

NAKKRCILVSSINGGLTTHQNPEESDLEDTEFVEEEHRKVAKNVSKIQVEAEEAEKSESD

DMDTNNGYWCSPSLAYLSKLSPSELSHVENFIVGRLNVGQIAYNFPVDLAGLFASCAERD

VPVSQELFGKIIKIDGSIVKVYSEEEEMNCKPGIGFELNVPATITIKAPPKKNISDQDHI

RRLQNLTGMEFVTYNPLTHYWTFKVKHFSIWGLIDDGEDDDMVTDDAKKLLALKKKQDDQ

EPEASATYSQIYENEAYQRELKRQRIGNYTSGLPGAWDHTANSVAGGSLGVKQELVQNEI

DRELREYKQDKSTDAWAMNLSDITSEGDESEDAKSLNSIALQNPLYPEEAKNYDYLKQIV

SVMPPNTDMEEIVNERAYEPEVSNEEDFDQFGVQSSLPTSKDWLLQLELANDIDSALNPY

LAIPYKKQSALSTVKDIVFSDVEKSKESKNNSRKPLKDESNLISEVSSTAYDNSAVTKLV

QKLLVRSTVSKRENSFPRLQLDKSLDFRAISFLNGHDTDSQLIELASILFDEVDISSNSK

YQAVDLSNKSIKHRLETLEQRNSFISWLKKYKLGSSSEFQGDSLDIIYDTICRGNLKSAV

ELAISSHNTHLAALLTLLDSNDQAVKKIAQSQIEDWKVTGAIEFIPAPIIKIHKILAGAF

DEVSQDIPYHVAIALRLLYGNPVANLSSVLSSVNCEDISDDFTTIIDIYSKFNYDGFKEA

TAQIEKSELSDKVKWLSLQVLSNKTGNLDSSSNDSVSNSFAKSLEESGLWKEAIFVYSSV

HDDSSVEQSIRRVVISNINEIKNDERDNEEFSVSVLGVPRSLIHEAIAIEKSKNGDYWGQ

VEALVEAQLWEKAHVVICKELGPETVISNSESGKSRLQALADMFPEKGAIIPEWNQGAGL

YVKYFESIDAFERHSDIATDDLNFLLCNLALVQVDSFMSQVALKIMTKKVGDVTLDHKQQ

VPDVKRKIHALIMGENERNYFESRLQGLNIR

>tr|C4XVN5|C4XVN5_CLAL4 Kinesin motor domain-containing protein OS=Clavispora lusitaniae (strain ATCC 42720) OX=306902 GN=CLUG_00054 PE=3 SV=1

MDSSNVKVVVRVRPLLPREEDKPLVSMPEEEPGTTILTVPDGKPPKKYVFDDSIWSYNPS

DSNYVDNAGFYRKSGPQLITHFFQGYNVCLLAYGQTGSGKTFTMIGDKTNPGIIPLMIKD

VMRHKESLVDQKIDCEILFSYVEIYNEKVKDLLDNSKQCRVREHPETGPYVENSTLVPLN

SFEEFSTYLNKGNKNRMVASTKMNEASSRSHAVITFTLKQIRFDNEEGSSAVGEPVEEMV

SNIKLVDLAGSERLSRTQMFGQSERIKEGSQINKSLTVLGRCINILAQGSRSVIPFRDST

LTYLLRENLAGNSKTAMVFCISPCDFEETHQTLNYASQVKKIKTKAKANESTLLSAPIDW

EKLQNMEKSVIDTLKEQIQQLTTELSDLKAGASQEPVESLIKFLEREREKQSFEVKYLKS

VVSSQQSRMDELQAQNSYLHQELSGNIRDRIQCDSDILQNELRRQRDDCALHWAEIKQLL

RELDPSHLQIT

>tr|C4XVN8|C4XVN8_CLAL4 Cystathionine beta-synthase OS=Clavispora lusitaniae (strain ATCC 42720) OX=306902 GN=CLUG_00057 PE=3 SV=1

MGHSQVAWIYSMQESVRIYEKETAPHVSSAPKPHLHFFPHRLLFIKETLTMVAPVAADAL

DLIGNTPLIKLNKIPQSFGIKANVYAKVELFNSGGSIKDRIAKNMVLEAEKSGRIKPGYT

LIEPTSGNTGIGLALVGAVKGYRTIITLPEKMSNEKVSVLKALGAEIIRTPTEAAWDAPE

SHIGVAKKLEKEIPNSVILDQYSNEANPSAHYYGTGFEIWDQTEGKVTHLVAGAGTGGTI

SGISKYLKEKNADIQVIGADPKGSILAQPESLNDSSEGYLVEGIGYDFIPDVLNRKYVDR

WIKTDDAESFKLARRIIREEGILVGGSSGSALQAALDVAKDLTENDTVVVVFPDSIRSYL

SKFADDEWMKTNGFEVEENSTAANKTDDFLTHKTIRDLVAGKAPVVTVTLADTVGKTFEL

LQSNGFDQLPVLSSTGNLIGLITLSKILKSLSTKKVQMTNSIRSIFLDFRKLEDFEKSFS

INKKSGFTKKSYEPITLDTSLALLNKFFETHSNAIITDNELKPVQIVTKVDLLSYMTKNI

SY

>tr|C4XVN9|C4XVN9_CLAL4 Transcription elongation factor OS=Clavispora lusitaniae (strain ATCC 42720) OX=306902 GN=CLUG_00058 PE=3 SV=1

MSGSVSSANEGKLIAFTSHPIMDVKEIKSTVSNLEKSSDDTTILRLLNILNDGVEPSEKL

LRETKVGVAINKYRSSSNSEISALVKKMIKKWKEAVQNEKNAKKNASLASASSSTSAHPA

PGSASGKFHTGPRNPKTDGVNTTLYDHSTRNASVSALYTALAIERDDDSKTILRIATEIE

SEVFKSEYSNVNDNYRNRLRTFTMNLRNKKNPELRARLLSGQITPSSFIKMTPNEMAPEA

LKKEIEKLHKQNLFDAQGATEKRAVTDRFTCGKCKHKKVSYYQMQTRSADEPLTTFCTCE

NCGNRWKFS

>tr|C4XVP1|C4XVP1_CLAL4 Ribosome biogenesis protein NSA2 homolog OS=Clavispora lusitaniae (strain ATCC 42720) OX=306902 GN=CLUG_00064 PE=3 SV=1

MYGCIYPDEAHFFTVSFFLSTSYHQQTKMPQNEYIEQHIKQHGRRLDHEERKRKREAREG

HRVAKDAQNLKGWRGKQFAKKRYSEKVAMKKKIKAHQESKVKGPSTPKQDDGEALPTYLL

DRQTNNTAKAISSSIKQKRMEKADKFSVPLPKVRGISEEEMFKVIKTGKSKHKSWKRMIT

KHTFVGEGFTRRPVKMERIIRPSALRQKKANVTHPELGVTVFLPILGVKKNPQSPMYTQL

GVLTKGTIIEVNVSELGLVTAGGKVVWGKYAQITNEPDRDGCVNAVLLV

>tr|C4XVP5|C4XVP5_CLAL4 C2H2-type domain-containing protein OS=Clavispora lusitaniae (strain ATCC 42720) OX=306902 GN=CLUG_00063 PE=4 SV=1

MISAFGHRHLPKPSQTAGRSFPTLSIYGNFLLSWQRMVKSEPESDDVARRRKKRNVGTFP

CSKCQKVFSRSDHLARHYLNHQPKEVYVCNHIISNHKGEKRTCGKTFVRKDLRERHLKRH

FLLQDSKKPEDESEIHTHTTLETDQSKTQLPLVGNPGNSTSGDSNSSAFMQPQFPMAHNS

PSPVHPVPPYQQAQNEQSQNQPVQNAPTQNLQSTNTSGFNNAPHLPHQLSHQLPHQLSHQ

LPQVGPQGIPPHNGYYQSPMQNHYAQGSHQITDSQDHTSTKFQAPDFPLANNRTYRPFPN

WRPDPFPSYYGMQSPPNNNQEINRGTNGAFPQSQNDILSWLFTDSSPEVSERNHFSRPYA

YLESSRPAQSPILPSHREMPPQSLPSDDDVGSSLSYFHNPGPDSLHTLGLQDLNFFSNND

NPLDLALLGPNAENSATHGFERNISMGFNFNTSTGSSNSPSNTNESSTPKTIGESSIPEV

DAKSLAERLETHQNKRNIPQYPQIYVDKSIFDRMIAPLPQLSRESIAEVLKLRSDKVAVE

DIISFYLYGYWESFNTSFSIIHRVSFDTKSNQPLLILAMVLIGCMYYPGTVDDDDDQEVK

MSPEYKLASMIAKPLRYALFQHEDFKSPVKVWILQSLNLLEWCEKNYLSREMHERAHIHH

GTTVQLLRRSPFLGGNPTVTNKAAASTSDTGEEETSDGNSDVEEASNADYVLFQKWIESE

SMKRITFMTFYVDIVDYIKFRHNPQIPFYQLQLLNLPCYEEQLWNSEEVNGSFRKLVKRQ

KKLSRPNHDMRSMKNGNRIKPGMNFLTAIKAIMRSQNLKNGSHKLPVFIKSILFGGLVSI

MHEMQQVELQSKFTMLMANDRLDKGSNQSWKELLTKVFDDWDMGHQSVHEHLFDDSIFQG

SLNQCSFPMYHLVQIIGFSDINHYDIAIFGGSPRNMSVDASAKDLKIVQKKLLSIWTKDT

KIRSVDELINTKSVIHSYWIHWELMLAPLNENGECISNHFAYNWNTGHSSLEMLYVVSIA

TLVLWCYVFSLNGAESNSFAELEGNISLEELRNYKKLASFAAEDGYHYLYRIQNEFIASL

KELGLLEGYVLHSRSRKASSVPLHTVVGKYCELLPSINLKQNISGLCFLVGTKLRKSQWQ

IIRENAKLIINCGLRSVGKKVVHCPDLFDNEFE

>tr|C4XVP6|C4XVP6_CLAL4 MICOS complex subunit MIC60 OS=Clavispora lusitaniae (strain ATCC 42720) OX=306902 GN=CLUG_00001 PE=3 SV=1

MFEITTLGTGRSTPVHHSHHSLWLVKKNLNTYLTSLSMIGAVKSRAVASSTKIACRSLVS

SARNYQLPPKTSPGTPVTSQPVKAEELHEHKPSPVVKEVVKKHNEEEEKKHQKKKFSFTG

FLLKSAALTSVLYGATLYAATKNEKVMDFVIDKQLPYYEQLINLIENGSVEDIEQSWHKL

TSSVQLPSKKQINELTSKWEQQGEHFIEETKKLATGKSSHLSTPAEQLQRAVEFETVSDV

TEKLPKIVLPKDASFADEHVRATIKSFNDLISLIDASSIGPQKDALIKNINTNITLLATK

LNKLNKSFDEEVQNRLKSAETELFSSYTQKELDMTRNMLEEFNQEKTHLENKYKAKLTKE

VEAAREAISQAAVNATSMVRVEQTKRFESMVKEKIDQERNSRLKNLEAVNSRLEEIEAFA

TSLEKQITASSSKSAVQHSLSKLKSLLFDTKEDSPATSYKSYVENLESVTSKSGDEVISL

AVSELKPVLDGESSQSILTIPQLLTAWEQLSPELRSASLLPPNAGLLGHLSSILFSKLLL

PVKGAKPNGKDIESVIARVENSLTRGELDVAVEEVANLKGWSRKLADDWVKEGRKRLEAE

FLVELIDAEAKIL

>tr|C4XVP9|C4XVP9_CLAL4 1,4-alpha-glucan-branching enzyme OS=Clavispora lusitaniae (strain ATCC 42720) OX=306902 GN=CLUG_00004 PE=4 SV=1

MSITDHKSLIKGALDLDPWLEPYSHALISRQLQHKQWLETLQKSEGSLAKFASSYEEYGV

HADPSTKNIVAVAYIPDVVSVSLVGDFNDWNTDSHKYEKVNNFGLWKLVLPPVNGHYAIE

HNSRYKISMVLPSQERIFRLDPWARRVTPSTESTLYEGRFWNPEEAYQFKNTRPSFAKND

GLKIYEAHVGISSPEPKIASYKEFTTNTLPIIHKLGYNTIQLMAVMEHAYYASFGYQVTN

FFAVSSRFGTPEDLKELIDVAHGMGIRVLLDVVHSHSSKNVEDGLNMFNGTDHYLFHGGP

RGAHDLWDSRLFNYSNYETLRFLLSNLKFFIDVYKFDGFRFDGVTSMLYKHHGLSYGFSG

DYNEYFNQDLVDEEAITYLMLAHTLLGELSAKENNFSFTSIAEDVSGMPTLCLPISSGGI

GFDYRLSMAIPDMWIKILKHLSDDEWDLGNIVFNLTNRRYKEKCISYCESHDQALVGDKT

LAFWLMDKEMYTNMSVLSPLTEVVSRGIALHKMIRLITFALGGEGYLNFEGNEFGHPEWL

DFPREGNGESYHYARRQFNLIKDDLLRYKFLFSVRCCHDPLGY

>tr|C4XVQ2|C4XVQ2_CLAL4 U3 small nucleolar RNA-associated protein 10 OS=Clavispora lusitaniae (strain ATCC 42720) OX=306902 GN=CLUG_00026 PE=3 SV=1

MTICSTVRDSQLCIFSHVSSHRFASRNEKRSRESKIQETSAMSSLSQQLKVISEKNASVA

LDRKTRSRIHSRSLIFDPKVAAAQDFDYIYQLGCEGLAELSEIDHRFEKFASTLFSETSI

NFDRNVQTKDLLTQVNKNLDAFINLIAPYYHLSPTLKAMEWLVRRYHINIHNTELLLVSV

LPYHSDPVFVRVMNVIPDDSWPPILSSISGYRREMKCPPVSSILKVFHNDPAFFKLYSEY

LVKQLQNKTVYKEQLVFYLSNTAQVLASHARDPAKLNDAYLPTVLETCNELFIERSFPFS

STLSADVRLTVYAIISVLCSIIPLTNTLVFTLCKSIIQGEMAFQASLRRQTFIVLGQLWN

YYNELDVPSDIELFSGLPAASMLEDASLVESLILEKYNLTKFLYFYFADRLNHQHTDALG

VLKYLDVSTSEFLFDAVSSKLVSFVFASSSLDETSRAQIIVVFEKLHKANAEKLVAILDK

NSKTLNDLEMSLMHTLSSGGEVDTSAHNFDIEDITLDLPTNNYELSQKFVKVRVHRSSFL

DRDSTSDFAKITHVLLETLRSTDITLQASVMIRFCRVAIGTDWGAQISYLLRLALTQAIP

SNIRILALACIRSKLLGKIHTDQSINFYLLVPIILLGFADENKQIRNYFLIVLEIIYKQS

SKLNEGNPKKVKCDLFMESQIYGETDASKRSLISPQDAQVMLETLMKDKGVVSEVVADGT

RVRHVVFNVLFKCVKSGSKKFGSLLLRTFILTQWSLPQWPLALKWRVWSIIGAENISSEG

TDDRFFFVDDVKHYVETRSQWSKEAIDTGVQLQDVDKVIVSLVGGHTTNEKKINKEIDWI

LKSLSTDGHLQVTANERLIELYPTLKSKDVRLKISLDLIDLVVKDNDLLLEFDPVETLQS

LFMTNDAMIDLLGTINIVQQIPDQGVAKRRRRSSSSIQKNMARDDISSMASVHLRKLSVI

LDVLESHLRRKFSEVANPELLQALFRILTDLDYLGNDGKMPVLYAQESLATCMLLSIVDM

KESSSKKKLKFDSNSIRADLIVNSIRLSQSPQVQNRLLLVIAELASLAPEIILHSVMPIF

TFMGAHTIRQDDEFSSSALQQTIAKVVPAITGASSSVSNEIEFLLTSFVTAFQHIPRHRR

VKLFVSLIKTLGCNRSLHLILFLIAQQYSANIAKGKMPECSSLLDFVAALMKTFTADQCL

DSICGFFNLWDTIPESELDKHSDEYSALSGRSVFGSAIVNLTTSELGLLKKRMLSFINQT

LKADEEMSLTSNTASLKMKVSLVLFDDRSNADEKESILSRFNKVSSFILTSLEHFSVKDG

KQDAEIGDELYESLKGLLNLLPLSYYISSVTGSLKNVSDPLSIKIAKNFAVWRAPNLRMR

LTSTALTKSLTISLFGAYCLF

>sp|C4XVQ6|MDM10_CLAL4 Mitochondrial distribution and morphology protein 10 OS=Clavispora lusitaniae (strain ATCC 42720) OX=306902 GN=MDM10 PE=3 SV=1

MYTHMEYLQRCFYKSTSWNEDNIYANVTATSQALLDFSIPSGAKLDVSTQATDHSASSMS

LSNMHTINGSLAYLFSSTPLKNTMGTRDVSLQDAVAGFRIIEPFFSGTHTKTDSSDVNRA

SLLYGRMYFPGTALEAMLIKRISASAQLLVKCINNPHIRKGGTMIVYLQENTPRFSREYI

YSTNEALFGFRCLYNFGKPTKISPALIPKFDNSVVSVGAEFWYAALGMSPGLSTAVRYST

RSTSTGKPLTMTLACNPILGHISSTYNVKTSVSSTVCSKYDFNWFSYASNLSIGFELYNF

FKPSHVFKHYHADQRRNPSIHHDTLAPVADPYVLGTRPTNNSVSYAFPEKPRKRVVNSIQ

NLDDYYHINPSRLHRSPAYDVDDDVSGRSVMEAFQNSVNESNFSSVLKGSTSLSDRMVKL

LWVGRYKDFLVSTGIKVNLNPFTNLPEINKIGVTFSYAC

>tr|C4XVQ9|C4XVQ9_CLAL4 Protein kinase domain-containing protein OS=Clavispora lusitaniae (strain ATCC 42720) OX=306902 GN=CLUG_00044 PE=4 SV=1

MSQVTQYKRTEVIGRGKFGVVYKGYHKQSKKVVAIKVLELDTQYDEVVDVQQEIQFLADL

KNVPNVTHYYGSFLVDTKLWIIMDYCAGGSIRTLLKAGVFEEKYIGVVAREVLSALSAVH

KMGVIHRDIKAANILITNEGNVQLCDFGVAVQLTTTASKRATIAGTPFWMAPEVIREGDQ

YNVKADVWSLGITLYEIATGNPPYCDKGATWAMTMIEKSTPPRLEGREYPVALKECIALC

LDENPEERPSADELLKCKLVKTYKNFPTSTLKEVISRYLLWRDRNSSRDSVFINMEDEPA

AEDNQIQMKWDFDSLSSKEYIIENDIHLNDTEDQLKTNGMDENDYTFTTQPTYTYQTNGG

NDTITNSNAFSFTSSGSKLNTADRSNQGTRNATTNGTTAPKSLMSLFEEDSTSDSEEFND

YKVPQIPNLSNLLSDSSNNNSSPTIEIPDMESLAKISKQPSSTQLSLNRSMSQSYNEEGM

TKLNKPPQLVHSHSSSAALDSRHNSPNSNRPRQRTISNSYGSVPSHPGMDPMGPHTPSFA

SEFSQIRNTPSPSTQPPFPSDSMATASPSKSMRALHSNNNPMLQPINFKSGAENPSSNKP

MASTLSAMSSANTSNQSMNSATNSNQSANHSTASIATSTVPSNSKKEKPSLRIQMPVPFN

SFNLQALTNENTDGKKPDENVNQFGINPALVNNMASMTPVTEKDSLLGEGETRESRADSG

PGAQRHPSSKKISAMMGPKTAPVTIPTQPNTPVFFGSRNPTLTNVGISSATASASNLNAS

TSNANASMKSDKFPKIPELNGEMFSDAVPKSRITQELENMIKLFSQGLDALESAL

>tr|C4XVR0|C4XVR0_CLAL4 Imidazoleglycerol-phosphate dehydratase OS=Clavispora lusitaniae (strain ATCC 42720) OX=306902 GN=CLUG_00045 PE=3 SV=1

MERYAAIRRDTNETKIQIVLCLDGGNISVEQSLFADKKDDEHATQLTGNQVIRVQTGVGF

LDHMLHALAKHSGWSLIIECIGDLHIDDHHTSEDVGISLGLAFHKALGQVKGVKRFGSAF

APLDEALSRAVVDLSNRPYAVIDLGLKREKIGDLSCEMIPHVLESFAQGASITIHVDCLR

GFNDHHRAESAFKALAVAIREATSKTGRDDVPSTKGVLF

>tr|C4XVR4|C4XVR4_CLAL4 Diphthamide biosynthesis protein 3 OS=Clavispora lusitaniae (strain ATCC 42720) OX=306902 GN=CLUG_00031 PE=3 SV=1

METVYDEIEIEDFTYDPITGLLQYPCPCGDRFAIALDDLKDGEDIAVCPSCSLMVRVIFE

PEDLEEFD

>tr|C4XVR6|C4XVR6_CLAL4 tRNA-intron lyase OS=Clavispora lusitaniae (strain ATCC 42720) OX=306902 GN=CLUG_00033 PE=3 SV=1

MIHLQVVHGTVLVFDVEIVARIRSLGIVGVLVGTLPKAPQQNVFLGLPLQLSTYEACWLV

AHGHAQFVDALKYNEIIASNISIDDTEGRIGDAPIQYAVTPNSFPSLTDVDISPAVLSNS

DFLAMQQVAPDFAAKFSAFCYLRNLDYYLMPGLRFGGVFVAYPGDPLKFHSHLIVKVLAP

GQKIDLLELVTSGRLATAVKKAWVLMDEKPQVQKKEPLLDTQGNTRAFSIEWAGFG

>tr|C4XVR8|C4XVR8_CLAL4 Vacuolar protein sorting-associated protein 35 OS=Clavispora lusitaniae (strain ATCC 42720) OX=306902 GN=CLUG_00035 PE=3 SV=1

MAVDNTVVQSCLANIRQQSQLMKENLSKGSLLPALKHCSNFLNELRTSQLTPKQYYELYI

AVYDALEILSNFLLQSYKSKSAKNKDTTFLTDLYELVQYSGNIVPRLYMMISVGTTCMSI

KGPETKEIMKDLIEMCRGVQHPIRGLFLRNYLTQRAKDYFPLSSEEDLEETVDFLITNFI

EMNKLWVRLQHQGHSSERELRYQERKELKILVGSNLVRLSQVIDDFQGGENYSSEEFYKE

RIFPAITEQVIECRDHLAQSYLIDVIIQIFPDEFHFLTLNSLLNDVFLHSHPLLKKSELV

TTLVDRFVTNHKYEEDLVSVENSTSQVNLDDNQNTKKQPASQNSISMTEVFQSFWDFYLN

LQSSQPELPPSEFISILQSLMKLSLTYDPENYENLDKIYSFANDKLSEHTSKSADHEDDK

SAQGLWLDLLITPVRYFSSVKSLIKLSFFHELFSKFSDVKLQKQLAVEIVDKLLEEADGD

STNQTYDNAENIDDVFKYILVLIQETDDSLNTAKDLGVTESIKVDGKEKYVSASFLETQE

KICKTLHLIESPDIFKNLSILLHVKKRYLNKNMANIIYTYPTLITKMTNKLRIAGYQSLR

ATKDGSDQTDLLITSNFKNISIIIDELYQHHHAFHSELILKLYLNLATVADQLQQETIAY

ELFTQCFVIYEENLVLNTSQNQMHNPHESMGGSVSYQAIVMIANRLATSRYFNKENYESL

ITKITLYGSKLLKKQDQCRSVYYCAHLWWWCDLWIDGPSPTLGTDAIDDGEKSKAHSEEQ

KPTEDATDSAEKPENSSNPESSSDDSGEKKAESDDATVQGNTQMLYREPKRVLECLQKAL

RVADSCMDPYLSLKLFVEILNRSLIFNVYGNWLVDSKYINGLIDLIRTNIANFRDEAARD

EDDHETRLLRHIEEYLKRTLVYVQSQQMSEDRFNNVVV

>tr|C4XVS0|C4XVS0_CLAL4 Pre-rRNA-processing protein OS=Clavispora lusitaniae (strain ATCC 42720) OX=306902 GN=CLUG_00037 PE=4 SV=1

MASVPSITPYRSHNDLLKLRQAFYAEPENRKLLREAVNTVNVLRSRGKLPHAVDATAWLV

SAKLSDHKDMEIFSLRSAYAMALVRFVNGMLDPFQQGAHAVALSTIAKSIGLTLSFVDLR

HSATHGQLPSLELLRSMSEKALKWLFDHYWASLTDVATTKRPTYEEKEKPQSVLLSLKIY

KKLRKQHFDLPLSRENPILKSYWNCFDALVAVSKDPVRSSQMVEMLVCQNFLIKTEGQPN

KFKTMLKIYSPLLEGCSPAFLLELVLAIIHHCSPLNDVVFTGTQIEQGQSWCSHLVSSIL

NGNFPIDTKHHKNLSKQNVESLLRTSLGLLEEDSPMVNAVENAIMQKPVPKRVYLPPSLD

EILAPSPYDSPKEASEDYMSPAPKKRRKTELFQANPEWTLTPFGVCP

>tr|C4XVS1|C4XVS1_CLAL4 Ammonia transport outward protein OS=Clavispora lusitaniae (strain ATCC 42720) OX=306902 GN=CLUG_00038 PE=3 SV=1

MTGPSSASVETDKYATSHEDRRPSKVHVSGDGNEFITLGDKKYYRHELMTAFAGTLVPER

YAPYPVHQFGNAAALGLASFALTTFVLGLYLAGAMGIKVPNMVVGLVFFYGGFVEAAAGI

WELIIGNCFAGTVLTSFGMGFWISYGAINVKAFGILAAYADEPDQLNNALGFFMLGWGIF

CFMMLLCTVKSTLVFISLFVTLDIAFFVLAGYYFTGHHQLMTVGGVFCVISACCGWYCAY

AGVATPQNSYLTANPIPLPVLGKSE

>tr|C4XVS6|C4XVS6_CLAL4 Peptide-methionine (R)-S-oxide reductase OS=Clavispora lusitaniae (strain ATCC 42720) OX=306902 GN=CLUG_00021 PE=3 SV=1

MLRSFLTFSRNMSRSEQEWRAILSPQQFKVLRQSGTEAPFSGEYVSTPAGPGIYECVACH

QPLYKGSTKFSAHCGWPAFYEAIPGSLKTIEDRSLGMVRTEMRCSKCDSHLGHIFKGEGY

NTPTDERHCVNSICLKYKPEEKSD

>tr|C4XVT1|C4XVT1_CLAL4 U3 small nucleolar RNA-associated protein 11 OS=Clavispora lusitaniae (strain ATCC 42720) OX=306902 GN=CLUG_00010 PE=3 SV=1

MAKLVHNVQKKQHKERSQTSDRAKYGLLEKKKDYKLRAADFHKKQAALKVLKQKAAQYNP

DEYYHAMTRKKTDENGIAIIERESSESLSAQQLKLLKSQDANYVKTMRLNEMQKIEKLKN

KLEFKSSGKHTIFVDSVEEKEQFNPEEYFKTDSSMLERRENRLRVDQLETSNKLIKRDLF

TDQEERDRLEKEKLNEYRLLKERMKREKQLREVEQKMEMAKELMKNGDRRKLVDSDGNVH

FKWKTQRKR

>sp|C4XVT2|ATP25_CLAL4 ATPase synthesis protein 25, mitochondrial OS=Clavispora lusitaniae (strain ATCC 42720) OX=306902 GN=ATP25 PE=3 SV=1

MLRLFRANPSSVKVWRPISTAVYSVRQNSSASKPISESRVSISEVEKDSKDEKEINEVSL

PWYLRDDITSSLVEKKEILLPEIPPHAPPQVEEFLTLMACDYGMDNIMLFDMTQLPDDHE

YKENNKDVDFIVVATGKSEKHIYKAANELRTHLKHKYNAMPSIEGMVSSAITPSMRRRLL

RRARKGPLATDNDYGKAANSWVICHHDGIDMHMLTAPRREELNLESLWCKPEDADKFSQD

SFVTSESDHIFSGIRRYHTFARAYSSATSDLESIYYKLQSQPVDAAEEELKRLQNLFEQS

FSHPSIKDHGLRFQFWKTLHLARPDLVSLAQVEDALLAKYCSAESLKADMTQEKIDDITE

YVKLLIDTPTRDSHKASVDLAFDRLSKIISTLYTFSNEKFSVAKNPQLVPLLWRLTYVEV

KEPVIGSRDVDRFIQEQVAVATSPGPVITMASNRARDILHLIGYHTKTHPGSVPTASLRE

LILFTYGNAGKWDKFWQEWDNYCFEKNFTPAESVEKWTRICVYLSMRRNKAEALRFLENY

WNNASSVAGSVYKSLQANGEEFNSPDERVAFKRALTNMIAMFETPEKVPFEGILSYVDQL

>tr|C4XVT3|C4XVT3_CLAL4 DNA replication regulator SLD2 OS=Clavispora lusitaniae (strain ATCC 42720) OX=306902 GN=CLUG_00012 PE=3 SV=1

MYIVKNSLHARNRKTRREYSSIVLTIFHYCLVMASASTILHYKRRIKEWEKSFFEKNGKI

PSKTDVKAEKEIWKAYKTYNQLKAKEAESSKENETKSKKSKHAKEEEHAEKPEGEGESGD

EKTIQNSPQQAPSLHAEFGPTPQANGKVLSIFDMVMSPPESSPLKGKRSVQIESSFSSPT

KPVSHLPSPKKHMSSEVFKTPTKAPRKLQFADLTPSNSSPSKKSLLSRLQQVSSPEKEPA

PQNVGTETPLYLGKINKKFSFKDEEEEASPVKQHEPSTPTKMSLSPANFNTTPSPLKPER

LLSFGSRKKLSDLFHECQNLEIDEEFEAQKVEIEQEIEAGAVNGNDDKLDESFVGRKRKR

ITQKRTTRRWKIKPRGENDEATVFEGKDVHAELQKMHEEEQKQLNEYMASGVNDDIIPTD

DDDNGDDDTFVRPEILKPRSMKSKNSSANYQRLKINDPRSKRFKQRMKRR

>tr|C4XVT4|C4XVT4_CLAL4 TRIP4/RQT4 C2HC5-type zinc finger domain-containing protein OS=Clavispora lusitaniae (strain ATCC 42720) OX=306902 GN=CLUG_00013 PE=4 SV=1

MTYDRLIDYAVNAIAQILPLDHDTCLEMIKYALTLPNAEVETHLLDLLGHSEESYKFISR

FLELKREEDENNSHQAKSKVKQTSKSSSPIPSITQNVKKNSAWDHSSQTTTKSNTRLKNN

KDSITISELADVKPSNKLSGQQAKKTKKKNLDSLKDIEAALNELEIEKAQESVSLESSSV

VKRVCNCMATRHPLFEVAPNCLNCGKIICVKEGLQPCSYCGAELLSEKEKIEIIKILNSE

KENLKIKSANSTNKQVQEQPSKLSAPKKIKVAMSAGGNLWKAQEEALKQIEEESKKQREL

EQKALEEKKELERQLQEIEHYERTKDVNPDLLKAQERLETLLDFQSTGAERTRIIDNASD

FEMPDLSSGSMWLSPTERALQLKKQQKQLRKIQSQEKARTGRTKKVMEMVIKDGKVSMVE

KHLIEDNEPDANEIKELDTLAKQEKVDQESSMIKNFWDYEKDQDKWEKPVYVFEPVSEAP

NVEWKDSKVQLDSLDSNELVAMLPS

>tr|C4XVT6|C4XVT6_CLAL4 Protein kinase domain-containing protein OS=Clavispora lusitaniae (strain ATCC 42720) OX=306902 GN=CLUG_00015 PE=4 SV=1

MRLSNAATAVCGRIKTTVASKEKFLFLILFLFWFDSFFFILFLLHSLPIMDNQDQGISPI

QIDSFQKNYYMISRLGYGSFGYALLAKYKKNINNFLDQGKFSVGTMMEPIRNGNLTNVRS

TGLVAVKVMKTQLKNPSDYLRVNEVKFILSIPSHPNLLQIYNLFIDETSGKLNIVMEPMN

QNLYQFIQKHVGRPLSSKIVKGMLYQLLSAIRHIHSHGYFHRDVKPENILVTSTQQYYGA

KHDIPPEMAKDAFVLKLCDYGLAKSVKNKRVLTPYVSTRWYRAPEILLRHANYSRPIDIW

AFASVAVELVNFRPIFCGRNETDQLWQVLKVLGHPGNSHRNDIGGKWLEAIELSQNLGFT

MPYAIGNSIHHILPSSYHELAETIKYCFLWDPDSRPTAEELFRSTYFKEYNTLGKEMLNL

LPTPVSPTESFGGACEEYDEFASASASRDCSEPGVSSHHAHVHPRISGLGQEIQLNSSQE

VSCDYKMVSKSYFQKYTTIIDSDMDSDRSKSSAVGKENVDAFYENVDHYDESFANSSFDS

HKIAC

>tr|C4XVT8|C4XVT8_CLAL4 Lipid droplet-associated hydrolase OS=Clavispora lusitaniae (strain ATCC 42720) OX=306902 GN=CLUG_00017 PE=3 SV=1

MSVTTNKDYPLTSEYFLASKNGPSPKLLAFIPGNPGLIDYYVTYLELLAESNPDFNILAV

SHAGYQTSDDFVAAGKSEKQPYFNLEYQINHKYEILRKQVLRGHTELYILCHSMGAYVTQ

RVVKMLLNDEEVSKVVKIKFIGLICPTIVDIAKSRSGVAFSRLFNYLPLVTVAVWFISLL

HFILPDSTAELIIRKFVISHPVLRDSKSMESRHNSIEATLKIYKSKRIVRQALNLAEEEL

LVIHRDDTLNDWFFRDLPETHGTVIWSFFAYKDHWVHDNTRDYILTRYHGPKSTLVHFEV

GNTNNENCPAITHSFCIDQSVEFAEITCKALSFAV

>tr|C4XVU2|C4XVU2_CLAL4 U3 small nucleolar RNA-associated protein 10 OS=Clavispora lusitaniae (strain ATCC 42720) OX=306902 GN=CLUG_00025 PE=3 SV=1

MLNTHTELIVSSLNAITSVINILGVKAIGLFPKILPPALKIWETTSESRHVSDSEEGSED

ESDNENEDADENESRMLIQGSILMLFSCLVKKMPAFVISNLKKMLQCILLSDLIETSIRA

SILNLVVDHIDKGQVLQSLCNLALNDDIYATDNAADLGLYLSAVKSSVDAIDKKAATAQS

SLFMKWLIKSFGFRTEYGEQKFTDNTIYSIEGSFHQCGISYVLKLNDKSFRPLFASLVRW

AVSGEGSLSTETTEVIRLTAFFKFFNKVEDNLKSIITSYFSYLLDPTIAILKRFQDGSLQ

DTNLRRIILHSLASSFKYDQDDYWTHQSRFETMVDPLLGQLSNIEDSIGKHLVKAISFFI

SNVSSDEYNEKLVHTLIRYISNEHENSSNTKIWTIRVLKTVFQKMGEQWLSFLPTFIPYI

AELLEDDDEEVEMEVRKDLVRVIENILGEPLDRYLS

>tr|C4XVU6|C4XVU6_CLAL4 Ubiquitin-like protein ATG12 OS=Clavispora lusitaniae (strain ATCC 42720) OX=306902 GN=CLUG_00079 PE=3 SV=1

MSTLSHSESDSSSNESINNLESPAPEKVPLSTSTILRKAEIPIAETLETRSTTGNKISIR

FQPIGSAPALNPLSFKVSGTQTIGSISKFLMRRLRLKTVHIYVSSSFQPTPDEKLGDLYG

MYKTNGELILSYCETVAFG

>tr|C4XVU7|C4XVU7_CLAL4 PUM-HD domain-containing protein OS=Clavispora lusitaniae (strain ATCC 42720) OX=306902 GN=CLUG_00066 PE=4 SV=1

MTEAKISVNTTAAPAASSATGNQSHSHNLSINSISSIAEPTTPPTATTGSKSYLWDSAKM

NGTLALPTFNLDSQKAPETTHIRQYSLESDPLETENQRARAYPLNPAIVIAPPVKIDKEY

LASINKTPLSQLKDQIFRLAKDQHGCRFLQKRIDENVVSNSQTREANFEVIFEQVHPILY

ELIIDPFGNYLIQKLIDYCDETNLNLILETLQFNLFSISINQHGTRALQKVIDRMSSDYQ

LSLLIKGLKPYIIELIKDLNGNHVIQKILNKYSPENCQFIYDSIIQDLLVVATHKHGCCV

LQKCLNHVNPSQLMQFSTNILKYNVFMKLVNDQFGNYVLQYLISIDSIDINGQLYSNFVR

FGVSDLCKSKFSSNVVEKLMRNCFNNEFKSVEFSNLKFSLVSQILSSDLNKLINDPYGNY

VIQTLIDTLINPSITYLFDSPGGGRILLPSLQMLVAEDYQNQMDSLQIQIIKRWFRNCKI

VSSFGRRIQSKINIILSGYSKVQRKSYPSQNGSSMPFIPPVSNQSMNANGEFIHTAGFTG

SKQISNRSMSLEPLQLNPYLGYKHSYANSHGQEAFARQAQNGSVQDFGMNQLSFGGYSLA

PHNSNNGAFRSAPRNLPSNAEIPEFRNSEYNIPNNMHNPKHSIAFNPCAVSYANSNEAMF

DYKPHQNAGNSGSVDNSYVTQPGSNGLHHYAPGRPPIDNSAKLVQQNVLAFNAQPQAGKF

GQDFQFGNSNYYNQTHPNIPFNSMGGKGRFSG

>tr|C4XVV0|C4XVV0_CLAL4 Nucleoporin OS=Clavispora lusitaniae (strain ATCC 42720) OX=306902 GN=CLUG_00069 PE=4 SV=1

MFGATSNNTAAPFSFGSSNQSGFGAASNNSTATFGSQQNKPATGLFGSSTATPAPSTGGG

LFGNTATQNQNQNQNSTFGAPASSGGGLFGQKPATTSAQSTAFGSKPSSSGFGTAGSGGG

LFGSASNTGANTNNATNTTGGGLFGSSTNNASTTGGLFGSTNNTSTGGGLFGAKPAAPAG

GNLFGTNSASGGLFGGNSGASNGNTFGQTKPSGGLFGTSNTGSTQLGQNQQQQQQQQQQQ

QTQLTAMTRVGDLPPQFKKELEDLDKYISTQHLIATTLNGDLHKHDSLIKSIPTDVDYLH

VKISSIKQALKFDIEHLKDIKSVNDELTEDINNIMQLIVQLSTPGSKFTSSFQLNDFFVK

RIKKYRDLLDVYEGVINESNEAISGLERSCNETYGNIYNVVEVVKNQYALFMELCETLAE

IHNEVDRYA

>tr|C4XVV4|C4XVV4_CLAL4 5'-3' exoribonuclease 1 OS=Clavispora lusitaniae (strain ATCC 42720) OX=306902 GN=CLUG_00073 PE=3 SV=1

MNSILHTCTHSDNDTLTRMTDDQMYAAIFNYIDHLFDIIKPKEVFYMAIDGVAPRAKMNQ

QRSRRFRTAYEAEENLRKAISQGEVIPKEDPFDSNAITPGTEFMAKLTNNLKYFIHKKIT

EDNRWANIKVILSGHEVPGEGEHKIMQFIRSMKSQDDYNPNLRHCIYGLDADLIMLGLVT

HDPHFSLLREEVVFGPSSRNKSGDIHEQNFYLLHLSLLREYMGLEFQDLEGELPFEYSFE

RVLDDFILIMYVIGNDFLPNLPDLHINKGAFPFLIGAFKESLKHMDGYLNEGGKINFKRF

GTWLEYLSEFELENFEKQNVDAEWFNKRLELISISGEKKRERSGKLLMLKDEKKLVSILK

PWLLETGVLPISQLTDLANKDALPTCSLPSDLAKAHMEFLKTFALETGILIVHSRSNDTY

EATIDVDGISPYETEDEFNERVVNLRKTIKNYQSSNLVATEETMKEAKDLYDEKFVRAKD

EYYKKKLHFSIHDEEKMIEFTRHYLEGLQWVLYYYYKGCASWNWYFRYHYAPRISDVAMG

VKDMLEKNQDEIHFDLATPFKPFEQLMAVLPARSRKLMPDVYRSLMTDEHSPIIDFYPHE

VEIDLNGKTASWEAVVLLSFVDEKRLLEALKPIEAKLTPAETKRNSYGQDVIFIRNPQND

NVYPTPLPGYFQDLEHDQCYEEFFTLPPVDANFKFELPEGAKLGKEALAGFPTLYTVPFE

SKLALAEVKIFNFPSKSESMLLKVGNMWEDISINQFAYKFLGKVVYSKWPNLIESRVTQV

WGLESKFEYVKSGAGRRVVATPLSPEENREFSAIKGNLKSKYEKTKGVILKKIEAVVFVQ

HVKGLVRTQKGAYVKSFHDEADVFPMQLIVENVVNEDERYASRPPVPIEEEFPIGLPVVF

LGAFAYGAPAIVMGYTGDKLNVSIQKIQSALEPDIGRKRYNIENKEIPYVASYDAAKILG

INGLLLSKITGSFMLRDRGMSKVNIGLDLKFDVRRLKVLGYTRKNGKFWEYSPLAIDLIK

EYKSKFPKVFAQLSKLGSRRDMPSTSEIGSPEELKQIVQWLKKAKSGLVQVSLESSSLTK

FSIAAIETYMDNYITRPLTLDSKDVKAVPKDAVINPSTSYSLLQNQRFTLGDRIIYVQDS

GKVPKLSKGTVVAITTVGSKTSLSVVFDHPLVTGNNMNGKLKSNRGLIIDSSLVLNITNR

QLVYHSNASKGKKTLSDAERAALLSKKQAADRAVKEENHKTTNELLALLNKSADKRGDKG

SDKGSENGVDKSAPKSNVEKEKESQSVTDDVRTNPTTIKQIYGQIYSNIMNQGVVMPGMV

EVPPYAASQGMQMPHGPVPQGIPVPQGMPMPGMPVPQGMPVPPAGFPQPIFKQAPNQVSN

GSETVSEDTPSNDRTSGNSRGGRGGSRGNRGSYRGRGRGRGRGRGGKPQN

>tr|C4XVV9|C4XVV9_CLAL4 Protein-serine/threonine kinase OS=Clavispora lusitaniae (strain ATCC 42720) OX=306902 GN=CLUG_00082 PE=3 SV=1

MSSRPIKAGRRCLFQVGSCRFYSKKYHPSPITCHSYIDDDFLSLVSKKQIAQHLSDLGAF

PSFSSILTPQHFYQNEVLMKYSKQKPHPVSLRQLAGYGKTLTKQKILASANFVRIELPIR

LAMRIRDLQTLPFGVVNNFHLAQIYESYYHSFNAFRKIPQVTTLSENDEFCRKISTLLDQ

HVFNLSHLMMGALESCILNTLRQEELDMFMSSMLRSRISRRVIVEEHLSLTEIYKKHPYQ

SKPPDYIGEIFQRCEAREHFNIVADTVKKSMLEHFPNEKNLPDLNIEGDVKTSFPFMVPH

LHYLFGEILRNSYQATIKTHGSSSNKKLPPIKITIIDTKKQVMFRISDEGGGISHKKLAS

IWSFGKNPELARQSLANFHRIPGLQMYSNLQVTPAGSSIVDNRDALGLTSVGDIGQVSTK

EKKSTLEQLTTRPNQYKLGLGLPMCKVYADYWNGDLTMNSLEGYGSDTCLTLSKLGYHSS

VNQLDRA

>sp|C4XVW1|GEP7_CLAL4 Genetic interactor of prohibitin 7, mitochondrial OS=Clavispora lusitaniae (strain ATCC 42720) OX=306902 GN=GEP7 PE=3 SV=1

MSLAMLTRNMLRRTSVRAFASSASNFTAGKDSARKLSPEEAKAEAAKLAIQSLKDVGSVF

SSGSDDAVQPIDTRPVFENPELFGTLNLLHQGQVLKELQEKYDKNWNKLTDEEKKLGYYI

AYGNWGPREKFINWNTQEAPYDLPFRVPSKVRLSNPQANDVVHKLEPLYLSETPVRKEQF

DTSKMDPVTKTFIYITLFVMLFAISRDKNTGESGKPQEIIIEDRYMKSKLEKEQKEKEKE

IEEENRKNQEKQARRKWYYLWLK

>tr|C4XVW3|C4XVW3_CLAL4 Cerevisin OS=Clavispora lusitaniae (strain ATCC 42720) OX=306902 GN=CLUG_00086 PE=3 SV=1

MLFSQSVALSILAALGANAMIIPNIGDVVDVFKNTDLSQFKEDVKEEAKNVGSTVSSAVA

DYESKLSSKLGPLLQNPRELIPHKYIVVFKNDVTPESVQFHQEWVAAKHSEHVAQADSSD

PFFATISDANIEGGISDVFDIANLVTGYSGYFLQSTIDWIRRDPAVAFVEQDSVVRANEF

EVQKGAPWGLARVSHRQPLSLNSFNQYLHDTEGGAGVTSYVIDTGVFVDHTQFEGRAKWG

ATIPSGDADVDGNGHGTHCAGTIASKDYGVAKKAKVVAVKVLRSNGSGSMSDVVKGVEFA

AKSHQADVKAGKKGFKGSTANMSLGGGKSTALDLAVNAAVKAGLHFAVAAGNENQDAANT

SPASAELAITVGASTISDARAYFSNYGETVDIFAPGLNIVSTYIGSDTATATLSGTSMAS

PHIAGLLTYFLSLQPGADSEFFVSEKGVSTAQLKKNLVSFGSKGLLSDIPDDGTPNILAF

NGAGHNLTKFWGESVSEVSVEPVGKTSVLEGKLEDLMSKVETEAKEIVNDVMGFVDGVYK

RE

>tr|C4XVW6|C4XVW6_CLAL4 Protein kinase domain-containing protein OS=Clavispora lusitaniae (strain ATCC 42720) OX=306902 GN=CLUG_00089 PE=4 SV=1

MNVSSGTRTPFLSSRSLPEDDNTFAPPALSAYGIALLSDKTDLASRSSLSGVYGEDIKEN

TALKNSLKSRLSVHFKDNGAAKDSGNASSTNSHSSSIYNSGSHNSSSHNISLSTSNHTLS

GTSFNSSSNFNGDDNDEFEDQSGDTIGKLDLMTATTPGGSDASSTRPSSTGLTNTKRSRL

ARRFKSLGPPKRASEMPPSSGDQDGNSGSSSGKQSPVTPSNDKDPAFTQSSQIQTPIRSS

IMSPAENGVDRKRTITGDSKFFKSLEIMKQKSPGLEPLADVKAKSPVAAERGPTKRSRSQ

NSSVFPGVSNSSSVSAFDPLLSQDKPRIPLQTISSNIMNIETGLSTFRKPKAPKLSFPHN

TSINADSASKYSHTPAVSSVEPLMSTRLSTEDQKTKKVIVINSRRYEKLELIGRGGTSKV

YKVRCMETNNQLAIKKVAFDSFDESCVNGFKGEIELLTKLKNESRVVELIDHVVSDGSIY

LVMECGEIDLAHVFSNRLAAGSSIDLGFVRFHAIEVLRCVEAVHRAGIVHSDLKPANFLF

VKGILKIIDFGIANAVPDHTANIYRESQIGTPNYMAPEALVETNHLNLAPGSEKKSTKWR

VGRPSDIWSCGCIIYQMIYGRPPYGSYSGQQRIMAIMNPQVKIQYPTKGIGEIPVPLSAI

ELMQKCLARNPNDRWTVEECLNSNFLKPRAVSENFVKDLVYSAVNFGFNARHTGEITDEV

YDRLVETVLKQIEDLNYA

>tr|C4XVW8|C4XVW8_CLAL4 tRNA (adenine(58)-N(1))-methyltransferase catalytic subunit TRM61 OS=Clavispora lusitaniae (strain ATCC 42720) OX=306902 GN=CLUG_00091 PE=3 SV=1

MSFYTYKDHIEEGDLVLVFMSRGLIKPLTVKRGECLNTRFGNFEHDRMIGMKYGSQMGGA

KDRGFVHLLHPTPELWTVSLPHRTQIVYTPDSSYIVQRLGITSGSRVIEAGTGSASFTHS

FARTVSDDGRLFTYEFHEPRFLEAQKELESHGLLGRNTMITHRDVCHDGFDIADIPETFQ

KNGGLFCDAVFLDLPAPWTAIPHLPSVVSSTSRVGICCFSPCIEQVDKTIEALEEHGWTD

IEMVEIAGRRWEARKEMVRDLKDVVKRLKDIQGRKTQGIESRRQVKIEPQAGEKRNISEV

DTQSPEPSERPQKVLIHSG

>tr|C4XVW9|C4XVW9_CLAL4 U6 snRNA-associated Sm-like protein LSm1 OS=Clavispora lusitaniae (strain ATCC 42720) OX=306902 GN=LSM1 PE=3 SV=1

MRRNCTWSLMAFTTAAAIVGSVDRKIFVLLRDGRNMSGILRTFDQFANLVLQDTFERIYL

PKLDQNSPVRFAEVPRGVFMVRGENVVMLGELDIDREDDHLAEMQQIPFEQAEKEWKGIQ

ENRIKNEKVKTKKYLKRGLIHDFVKSDLY

>tr|C4XVX1|C4XVX1_CLAL4 E2 ubiquitin-conjugating enzyme OS=Clavispora lusitaniae (strain ATCC 42720) OX=306902 GN=CLUG_00094 PE=3 SV=1

MSSKRRMEKDVMDLMMSNHEVSLIDDSIQQFHVIFKGPEDTPYAGGTWKVRVELPDQYPI

KSPSIGFVNKMYHPNIDENSGSVCLDVINQTWSPMFGLLNIFENFLPHLLRYANPSDPLN

TEASNLMTKDEQRYNEVVKRYVKEYASEDFSATEAKESGDEEENELSDVESLSSVGEDED

DEDD

>tr|C4XVX2|C4XVX2_CLAL4 Succinate-semialdehyde dehydrogenase OS=Clavispora lusitaniae (strain ATCC 42720) OX=306902 GN=CLUG_00095 PE=3 SV=1

MSTVSRRYSTRVASSLKNSSLFKTQGYVNGEWIKSNASFDVYDPGLYPKADSKIARVSCY

SKADYDHAIEAADTAFRTFRKTTGRERSQMLLNMYHLMKENQEDLAKLIVFENGKPYADA

YGEITYAASFFQWFSEEAPHICGDIISSANAANRILSFRQPIGVCGIITPWNFPSAMVTR

KLAAAIAVGCTAVVKPASETPLSALALAQLADDAGFPKGVINVLPSSRAAEAGLAICENP

IVKKVSFTGSTNVGKILMGQAASTMKKCSFELGGNAPFIVFEDTNIDQAVSGVVASKFRS

SGQTCICANRIFVHERIYDEFSERLVAKMADESVLGYGLSEETTHGPVIHERSLEKVKDH

VSDALSKGASLLLGGQPRPELGDYYHDLTILGDVTREMKIFHEETFGPVCPLIKFSTDEE

VLELANDTEVGLAGYFYTNDVSRVFRVAEELNVGMIGVNTGGISEAALPFGGVKESGYGR

EGSKYGIDDYSVVKSMVLGSIH

>tr|C4XVX4|C4XVX4_CLAL4 HTH APSES-type domain-containing protein OS=Clavispora lusitaniae (strain ATCC 42720) OX=306902 GN=CLUG_00097 PE=4 SV=1

MRRCKDDWVNATQILKLCNFPKAKRTKILEKGVQQGLHEKVQGGYGRFQGTWIPLADARR

LADEYGITAEMVPVLYIDVKDPSIIIPKKSKPPSSSTALNKDGTPVKRKYVKKAKKDNLT

PKKMKFDDNLPPQAVFTQDYPPSLSTGEAHILQNNRSLPMQQIPMNSQQIQSSHLPAPFQ

QSDFPANNILNPNQRVHMENFQDYPNYQMQMQNYQQQQQQQHQQQQQQQHQQVVPSARQD

SKSYGYTGAIENVAQQQMLYQQQQHHSKGGSASTNDTNWSQDEHSRDSDTSVSSMEIKNN

KALLEEDNSHPAQLLRFFSEEKAPIPYFLYNPPPDFNINEAIDDEGHTTLHWAASIGNLN

LVHLLLSKGANPLVVSNYGLNPLSKSISFNNCYDLKNFPQIIDALESCLINTDINGRTPL

HYLCQFAKVQTKLPSLSYYLGIIFNKLVFMSESNKGTGVNLMKNVIDHQDVNGDTCLHIA

ARSRCTQFVKFFLSNGARDDLINVNNETAKTIIIQQDLLVYNFDSPNMSLHPSLQAFQPS

HQMPSDDQMNTRRSQGGPRLGTPIQASRHTETPDTQRTTIQDDDEYEEVNDHVSKEHLRT

LLEEQAGTVEENKENIFEDLPKTALRSASPSKPHIGGVNAHIRHNVLRAPSGRGEAKTPS

EITSSPTRSYSPHPPQLDDEGHVIDSSSSYGSSEEQNGRIPVKDVSSMMSGMVNLLTDSY

IQELNGLKIESKRVKSILLEKKDLNNKCIERVKILLNKNGFEDVSSLDEARKVVDEELNV

FAQEIQHKEAQLTCSIEKAQAFELANLVQDNESQIQSQQEGGNGLPDNLELGVALTEAQI

KRVDLVHKVVESVKNFAIDSKMNKYRKLISLSCGLRMEDIDGLIDGIEESLMEKSH

>tr|C4XVX9|C4XVX9_CLAL4 FAD dependent oxidoreductase domain-containing protein OS=Clavispora lusitaniae (strain ATCC 42720) OX=306902 GN=CLUG_00102 PE=3 SV=1

MSIPVVVIGAGVVGLTTALQLKRENSAYDITIVGTFLPGDISLFYTSPFAGANWHSFASV

EDKYLQELDTVGYHELMRLADDPQSGIWRKPNASYYTPQALEEVNGDVSKFNDWFDAMTN

TRILPKDELRPGTVFGKESEGLVLSVPRLLDLFAPKVSCIRNCREKSSTDHPH

>tr|C4XVY0|C4XVY0_CLAL4 Dolichyl-diphosphooligosaccharide--protein glycosyltransferase subunit 2 OS=Clavispora lusitaniae (strain ATCC 42720) OX=306902 GN=CLUG_00103 PE=3 SV=1

MKLSVYIPVSWILLAASTFAYSVEHFGLSLNSQKLYNAKSLPSEVIRVGIEKPKDALTFT

IKLASSERPHQSILLFSDEKGLDYAVFPDYNAQKHSLAIQIAVSSLPTALVRQGKITASL

VIASGDDVNENIHTKVAELTCSDELRETTKEKQAERFGVLPEIHHIFKQDESTVNAIVPL

AFSAVAGVLTLVLFVVWSSIFGENNSNSSGGTYKTGFLVALVAVEHTFLRYYLGASIFTT

ISHVLLLVAPSVFLGSKALNVMAKSRAA

>tr|C4XVY1|C4XVY1_CLAL4 Mitochondrial inner membrane protease subunit OS=Clavispora lusitaniae (strain ATCC 42720) OX=306902 GN=CLUG_00104 PE=3 SV=1

MLPESAKFIGNILSWTLRAGCATHLFNEYVYEFTETKGESMLPTLQAQHDFVHALKKHRL

GRDVEIGDLVVALKPSDPDHRICKRITGMPGDVILVDPSSSSQITNSPNLCIEHDGFNKY

VEVPEGHVWVTGDNLSHSLDSRSYSWLPMALIKGKIVAVNSMDKSLWDDDGKLWFYNFRW

IDNTFVDEL

>tr|C4XVY2|C4XVY2_CLAL4 FAD-binding FR-type domain-containing protein OS=Clavispora lusitaniae (strain ATCC 42720) OX=306902 GN=CLUG_00105 PE=3 SV=1

MYRFGVFRSHFLTPAQLTLRAKCLSVKPSHSFSGRFYSKENTKKNEDKKRQDEQIVKKQA

EEVSFHIKPKTSPSAPAPMDPELGIEKLMSKNNKPYIPKLKHERLTYDYPGLPNEDAFSK

HSNDAKKPKTVNRWSRHVPKILTVLVVLWGAYSVKVWYFAPEKGSDSKELLDPYEFHKFV

ITHKHQVDDDHFLIEIRPKFKNWQYSYYAHYENKSIWNGDRIWSVEIKQPQIMVSRSYTP

LPLYFMKSERTRSGEKDPVLRVIDNDAEDYDKGGVMTFYIKRYEDGEVSRYIVNKEVGDE

IDVRGPHVEYKFPHHPLKQLHERPGFRDLPSKVEAESLLETIKKENGVPDFDNLAFYAAG

TGIAPILQVLFSKNPYRGFTTVHYSAKSSSELGPLERFLFFLEKLDRIKLVSHIDEKPKT

KLKAGDIDKPVPRNYISPMRQEIEGLDEGEIKTQSPEEALKLRMAIMDGPKSSSERTTRA

NEEKRAPRYENALQQATVTSREKKADPALSIVCGPYGYVDFVSGGKLMATNEQGAVSGLL

GAKGWDNSNVFKL

>tr|C4XVY8|C4XVY8_CLAL4 Mediator of RNA polymerase II transcription subunit 17 OS=Clavispora lusitaniae (strain ATCC 42720) OX=306902 GN=MED17 PE=3 SV=1

MADSDRVRLHLDKSLYGKNQDPFLQDEDSIPIAELLPQILRERQSFLNITEDSLQAEIEQ

KNKGIDTFEDNETQPVEETDEDTESAFQKFQKQKMELLGHINSAMNETSLSLDFVSLLMS

GQKPNVSKATMSPYLTKHVPLGSLGSDRLSQNSEPEHNKGDGDAQKVASVGLGWKYQSLN

HISELFKGAGSQLRSQVEVERHYWNSINTVLNHGEVLFSLRDPLTNSRAIGVKYGYGDSG

SNYFDKGLAVLRKDDETGEITFSPITTGSHRLSHKATKFTRVKILSKIDNDFMLTGQSLF

EKKCMEDKSEHKVINDIERARYFLFEEDLFYHLIREAKNLISYNVSIISNKIIIEIYDQI

IEIESVIYDENNEEELSNTYQNINKESSKNNDKAQAILTFLKLMLCCYYNFNLELKQKIP

TSFTKWKQNNTHPLMLRPLIGNIRHEHNVHSMKRIIHRICKSLDSSLFSHEVKEEKFLNL

KADKITNPFKKAITKPISTFTLILQKLATKEHLNVEVEVTTSDIFVNLILILTVTKYKTN

EDLQQNQDGSNVLQLKFSDFTEIEESMNWTILNFLQSQ

>tr|C4XVZ1|C4XVZ1_CLAL4 Adenosine kinase (Fragment) OS=Clavispora lusitaniae (strain ATCC 42720) OX=306902 GN=CLUG_00114 PE=3 SV=1

MSYPLVCLGNPLLDLQVDVDAAYLEKYSLNDNEPILAEEKHMPIYDEVLKMDGLKLIAGG

AAQNTARGAQYILPPNSVVYFGSTGNDVYAEKLKEANAQYGLRTEYQVQESTATGKCAAL

ITGKNRALVTDLAAANLFTPSHLQKPENWALVENARYFYIGGFHLTASPEAIETLGKHAA

ANNKVFAMNLSAPFIPQFFKDPLDKNIPYCDYIIGNETEAAAYSESHDL

>tr|C4XVZ3|C4XVZ3_CLAL4 DNA polymerase V OS=Clavispora lusitaniae (strain ATCC 42720) OX=306902 GN=CLUG_00116 PE=3 SV=1

MSVSRDYYYKLSSEIPQERIEAATGLLSELSAVNKKEEWDYALGRLIKGLSTSRQTARFG

FSMALTELVRELVLKEDYDLSISSMLDKIVDATQVSSSMKGKELRSVLFGRLFGFQALVN

SELLLQKESSSQEDLQKFVRLLVELSGTKSWLRESAMFTMCQFLGSFLNSDMFSEDTLVT

FLQVISDQDLTFTTEGLAVYLVIPQPLRSRVAQKVTGVSLWKNGDPLSSGNLQVLAKVMK

DVDVVSDESSEEAESSNKKNKNSKQKGTWSPRLPFAWEYLVKHFAAKDSDDDSEEIQEEH

SKKRKKSGSSSSKKKAKSDTSGAISFKEFWKVVVDETMFAEKSSSERKYWGFEVFMKFIS

VLPANLVEFSFTPNFMRCLINQSALQNRLLNKISTKAINTIIEVSQADLSKVVPFLKCLI

NEKCGGSWNFDPMTKSKVTDALVGVLGYVEDVNSVSDSQVDELVNSIKEVLVAKFDEALA

SQIEPENTDVLAHKKSNDNILKWVLDKLLVLIRSTKRFKASKSKSLESIFKFLIQHSFFK

AKNAPSVSSNILKLIQDRLNSFLSEVILSKRKEHSWSFYCVKQIKKLEDNDKFELVLELD

SDLDAIKEDGLEMINTIKDAMKRDQAHKDQQYCFELLFSMVLIQLYMGEAETVDVLQELK

DCYVETFSGGKEENDSSVTMTEIILSFVSRKSSLLKKLSTIVWESLLCAKGADGRLMIND

ACFDLLFAILKTKENEEGQNALFEGEDEFNAASEDDDNDEDKENDVEDQGEDQINAESDV

ESNKSITSESESDADDEDEGETDADDNIEKKTNIKLAKALGIPTESSGEVKFDEIDSFGE

DGDDYESESMDDEQMMAIDDELSRIFVERRNALSANSTNKKKAEMLEAKENITLFKSRVL

DLLEAFSKEQPNSIYNLSFIKPIITLMNSTKDKNLGMKAHKLLKTRISKTRITLEEFAQL

YETEEEKSKFKKTQIETLEWLQLQAGKYSSNQAHASACGQSCIIISKALVSIDSSLLETI

INIYTRTLLVWATESKNRIQASMFFDFINWLNSKRSNHT

>tr|C4XVZ5|C4XVZ5_CLAL4 Dipeptidyl peptidase 3 OS=Clavispora lusitaniae (strain ATCC 42720) OX=306902 GN=CLUG_00118 PE=3 SV=1

MSTFLADSEAPIVLLSVKNHFEQLPSKEAQAYAHHLSRASHWGTRAVLRSVSPESETIYD

LVLGIHRALGEPQSNEEYVKALGEGVSAEQVTLYLEYASQFLSNLGNYKSFGDKKFIPKL

DKEAFEKIVAATKDAELLKSFKSVETPLYDTTFALLGWPEKGQLSAYYPDCASVIKKEEI

EAVNAALAERGIMPENTRVQKKSDKEFVVLVASALTSNTTDYYPKESIQLANGASLSIQF

GDHSKEFAKIVEHMKEASKYVANDTQKKMVEYYVESFETGSMNAHKNSQIQWVKDLGPYV

ESNIGFIETYRDPSGVRGEWEGLVAMVNQDRTAKFSTLVNNASNLIPFLPWDKLYEKDVF

TPPDFTSLEVLTFAGSGIPAGINIPNYDDVRLNIGFKNVSLGNVLSANPKKQKKEEVITF

INASLQEKFRKWRDDAFEVQVGLHELLGHGTGKLLQETSPGQYNFDKAAHPEIQTYYGPS

ETWGSLFGASAGSFEECRAELVALYLILSKPLEVLPIFGITDAQEQEDVKLIATILMARA

GVIGLEFWDPESKKWGQPHMQARFGIFKCLHKAGVVSLKHSEGTFDDLEIVVDESKLNKE

AVDALAAFLHSLHVFKTTANVKEGLAFYNDMTEVTEEYACLRDTVLNKKLPRKQLIQANT

FLGDDGSVEIREYDESEVGMIQSFADRRV

>tr|C4XW00|C4XW00_CLAL4 Zinc-regulated transporter OS=Clavispora lusitaniae (strain ATCC 42720) OX=306902 GN=CLUG_00123 PE=3 SV=1

MFDTAIYELSRRDTCDNGNDYNGMMGARISAVFVILIGSAFGAFFPILSSRYSFIRMPSW

CFFLAKYFGSGVIVATAFIHLLQPANEALSDECLGEGWSVYPYAFGICLFTLFLLFFFEL

MAFRLIDKKLEGLGEEGHSHSHFGESSTYVKKDLDSDEEQIGETAQTKTESNAYPSHFSH

AAEHQDQEAVGTPANDQGKEQYYGQLLSVFVLEFGVIFHSVFVGLTLAVSGDEFKTLYVV

VVFHQLFEGLGLGTRIATANWPSHRRVLPWLLALGYALTTPIAIAIGLGVRETYPPNSAH

ALITNGVFDSISAGILIYTGLVELMAHEFLYSNEFKSSDGTKRIIFAYLCMVLGAGLMAL

LGRWA

>sp|C4XW03|AIM36_CLAL4 Altered inheritance of mitochondria protein 36, mitochondrial OS=Clavispora lusitaniae (strain ATCC 42720) OX=306902 GN=AIM36 PE=3 SV=1

MARYKRPTSPKFQRAINMFRLVAGKRPPVRTVLSRGIPCPSFAKAFYSTPQRKIRKPEEG

PKIRYLVLVVFASFGLLHFVTTQVDKKAPKNSFTEREFEQYERETGLRRRHKLINHEKND

QYAFYAVPYAHDVSKAVQLLTKMLPNEKQVKVIDPKQLIEKELEDEGKYSYLLQDLLAYK

KPLPRGLITALMKQEIELFLNTTKGQFDTNILLMNYPQSTDEAIKFENDVSELKTCIVLE

DDFAKSLHDDLSDDDVRKVNNVVGYFDTVGKAEKVNSKVKVLN

>tr|C4XW07|C4XW07_CLAL4 Peptidyl-prolyl cis-trans isomerase OS=Clavispora lusitaniae (strain ATCC 42720) OX=306902 GN=CLUG_00130 PE=3 SV=1

MFRAARSFSSSARALGTKVFITPSINGVKQNPIKFELYDDVVPKTAENFRALCTGEKGFG

YAKSIFHRVIPNFMLQGGDFETGKGYGGKSIYGAKFPDENFEKKHTKPGLLSMANAGPNT

NGSQFFITTVPCPWLDGAHVVFGEVIEGFDVVKDIEAQGSSSGQTKTAIVIEECGEVKE

>tr|C4XW08|C4XW08_CLAL4 Integrase catalytic domain-containing protein OS=Clavispora lusitaniae (strain ATCC 42720) OX=306902 GN=CLUG_00131 PE=4 SV=1

MSNTSDSIEGSTSSEVSFRDSHGLISGLPARSQGVHSAATTVQKVSQVMSRSKINEINPF

DLNDEVFFPEWYYCFKINFVKAVPIAEDYFKYSLEDYRLRFNGSVGSINEAVNAVEQRFH

ECISHLLEEQSLIDPYTFCNREYVENELFADREINEIEDRVKIAIRYNILRAKAVHGDYQ

PLLNHLLAASTSPELIWQTFFRILTTSVTFLNPTQLEDFTKSVQSAMTRSTDVSTQIRRW

YKTTELTRKSFCPTPKDTTRRSHSKNDDNRNMGKTRNAHTYVNNGTQGTHKRGPYKTNSD

MHSNAQNYQKSNKFKSSGTPPVYLVYDNDDISVYPTRTFILDTGSSIHTVNDKALLSNVC

STNRMINHGGLRPVDTVGTLKIQLLDGHQLILPDVHYVPYLPNILSTHRLRDAGDSIFTN

ASLDAINSHTQQVVSSETSETINVPATILPFESEDLPSDISNTPHTSTSKLPSVLIISTQ

SSEDHFSDDSSDSESLFSNDPRSETESYDTLDDASTGKIDALSQQWHSTLGHPGETQFKA

LKKVFNLPKSVVHVPLIQCRGCCTSKTVNRFAKTSRGHTSITRPFEVIHVDVCGPFDNPK

AHDNARYFLTIVDRFSRFVTAIPLARKSETSTMIQDFIRQSFTELRATHYPKQLRSDNGS

EIFNDNLTEFLKAEGIKLCPTHPHSSAENGIAERMHRTLQDKVRTQMTHGNVPPIFWSEA

LRYSALILNWTPRVNLLNETPIRRWYNDDSLTRPELYPFGCTAFVTIPLDIRTNKMAHNS

LECAYLGPDSQRLGHRFFSYELMKVFGSAQAIFRPNEFYFFQYAINPDKLPVNHNRLPTA

YLPGIKVPPPFQRLSGQDIYNDTNALSELFLEQTPSTTSSVTTPITDSPPVVPAASPTLS

IPESSPMPPRKVAKKKRSSIPSTSSAQSSTPSAIPSTTKKSTSKQSASTKSSLKSSTTLT

PSPPTKSPSEPYYRTRSATGSLPSDRRLSTKGG

>tr|C4XW12|C4XW12_CLAL4 Nuclear mRNA export factor OS=Clavispora lusitaniae (strain ATCC 42720) OX=306902 GN=CLUG_00135 PE=3 SV=1

MLAAGSSQRMNRSANQNNGNSNESRRGGRKKMLSRNNQNGTSTGSRPKSGTSTNSAGKQG

NNGNTSQNFHAKGRGSSQKAPNNRRQKTQTQETQTGTNFLDSPLSRGAVSAVVDGPSQNF

TTNEVAQVGAVFEDPSKLGFMKSQQKKKSRATPRYMLSQPRLLVTPPFHQDPWDFENQAK

MTDIEAKNNGSDYQGIYEEFQKMREVERKKMEELGLVDAENTRKDLNDAIYFQGTCLDMC

PVFERTRRALENNVKTLEKDPTTNKISRERAVKAFSRPAAGQPPPMPSDVRPPHVLMKTL

DYIVDNFVDQLPEAHSFIWDRTRSIRQDFIYQNFYGSEAIDCNERIVRIHLVSLHVMAGS

DVEYSQQQELEQFNKALQTLTEIYQDVRNNGGQCPNEAEFRAYHLISHFRDPELEREIQG

LPDHIFRDHHVQLALRFRYLMAQKNVVERGYTNTIGPMDLFVEFFRLAFSEETSFLLACL

LETHFNEIRFYALKSMSRSYHTKGKPMIATALQKMLGFDTIDQLISFVSYYEVDIINDNG

TVLVDLFNKEKLESKYKLSSLNDKPKLSQAFSNQLNYKMRGTLKSFINSGKSNENLNLKN

APGSEVLQSNSRKPSSIKSQVPSFVPSNANGPLTGHSVTSAFAANNLQSSVQLENGKSLG

FGQKEGFSQQPISNTLKEPSISNEQKSGTSFNISDFLSNQNTSLGFSSTDSKTMLSKPNE

HPNEATRSNKSNLSFQAPKMSDKSLRSKGKEEEPSQSFSMDQNNFSPTITPSVSRSLKTP

VFSNPKKEQHKPSIANTNEFTGLAAPPKQNRMKSTDSPTLSINPNPPAQTIANKIDTKGI

AEQIYGQIENSVLDEELAKLLGRLIKHRNRLEERNQIIESFTSELYSAFVSELTHQSTLT

ILADEYCKRITLKHAFSNWQKRLSEKMARKEAKKRRLQELRSIEFSRPTLKRRSLTPDIN

QPFTKRSSPSLSQNTSFEYMQEKQNKISKLWQPLNVKKFVDLLSHGFENHFNKEKEILKC

LFIVEDWSSPYSKWLNTKFSLKLSDDRTHYSNRVESEHFGMDFESLPKNPRSLENSIRNV

CLIVFECGMVTPSAPYGSTEEKLIRDSKTFQKLVQICDRYCLFKVQFLVLFWDTCKSNLR

KEKVEEILKVKEITDADNSVENICICDMSNVNTNVAETLDVGFQRLCQNFTATLTTRGGK

FRMKQLQLRRERMGDDNDLSVQPEVSRTISSHLKTKEEELARKGRELEKRKYLAKHIVTG

NSDHIDLSNASFNNSVFKTPNGSFLNNSVINLNTSFFKNNTTLPHRDNSYFGSFANGSIL

EESTPFASPGPKFTKPLLPKKVQELKELSAAIKAKYRK

>tr|C4XW15|C4XW15_CLAL4 FACT complex subunit POB3 OS=Clavispora lusitaniae (strain ATCC 42720) OX=306902 GN=CLUG_00138 PE=3 SV=1

MVNTDFEKIYLNQSRVNGRMRIADSGLGWKASSNGDSTSNAPFLLPSEEILATHWSRGSR

GYELRVQTKNQGVVMLDGFDAEDFAKMKQELQRNFHLTLEHKEHSLRGWNWGKADLARNE

LVFQIHNKPDFEIPYSEIANSNLTGKNEVAIEMNLTNEDKAGDELVEMRFYIPGTVENET

KKTVKNEDGSEKVEEETEEVNAAAVFYDQLKDKADIGQVAGEAIVSFSDVLFLTPRGRYD

IDMYPTSLRLRGKTYDYKIQYKQIERIFSLPKPDDVHHLIVLQIDPPLRQGQTRYPFLVL

QFSREEETELELNLADDVYDSKYKDRLKKTYDSQTHIVMSQCLKGLTERRLIVPGSFQSR

FLQAGVSCSLKASEGYLYPLDRCFLFVTKPTVYIPYSEVSTVTMSRTSTGVSASRTFDLE

VNLRGGNQSHVFANIDKEEQETIERYCQEKGLRIKNEEKIAKAMLAKAMTGDDDDDDDAD

VDMGSAGEEESDDDDFGSGSESDVAEEFDSNASASDSDEEMASGGEDAKQPPKKKAKN

>tr|C4XW17|C4XW17_CLAL4 Endoplasmic reticulum transmembrane protein OS=Clavispora lusitaniae (strain ATCC 42720) OX=306902 GN=CLUG_00140 PE=3 SV=1

MALYYNLVFGLLVIEMTFFGVLSLPFPRNIRRKVLLTASAPFRSEQVQIAIRCIFGFVLV

LFIDSVNRVYSVSAELHASAPQNAVGAVVNDRSEIQSRRFYAQRNMYLCGFTLFLTLILT

RTYSLVTELVDTKDKLDDYKKNSELSGESTTDSVEVAKLKQEVALKDKQLELLKEQATNL

SKDYDAASTTATKRT

>tr|C4XW18|C4XW18_CLAL4 tRNA-dihydrouridine(47) synthase [NAD(P)(+)] OS=Clavispora lusitaniae (strain ATCC 42720) OX=306902 GN=CLUG_00141 PE=3 SV=1

MSVSEKRDHDAVNGENAKRIHLDDRQKGFAPIKAEYIIENTKAEENYDDEAAEGGDREND

DRSKRSKKGRGQNKKRDLKQQKEEIRLCSSLLDPNDSRECPVGADKCKNTHDIEYYLSTK

PADIEGKCPVFEAIGYCPAGLKCRWLSSHYNKETKTLLKDLAKIEEAKLTNYEINKIEPE

HRIQLQKNKYEFKQATPYIKYLDSLVQNDANIAKRQEDIKNNISTYVEPPFAPAEKKKIN

LNRAKIVSPLTTVGNLPYRRLMKTLGADVTYSEMALALPLLQGTKPEWALPKAHASEYPG

FGIQLATNHHQQASKAAEAISREAPNVSELNLNCGCPIDLLYRQGQGSALLDQPSKMVRI

LNGMNACSGDIPVTLKIRTGTKDNKNTAIPLINRMLADTHVAAITLHGRSRQQRYTREAD

WNYIAEVGKTVQEWNHKKEEDKDSSETNPTWFIGNGDCFTYEQWHNAVNTEGIDSVMVAR

GALIKPWIFEEVDAEQYLDKSATERLEYLRTFANFAVEHWGSDEYGIEVARRYMCEFISF

THRYIPVGILERLPPKLNERPPKWKGRNELETLLGSTDYKDWIKITEMILGKASANFSFI

PKHKSNSFAKN

>tr|C4XW26|C4XW26_CLAL4 Peroxisomal ATPase PEX6 OS=Clavispora lusitaniae (strain ATCC 42720) OX=306902 GN=CLUG_00149 PE=4 SV=1

MPIATNKIEGEKSISTSAALHIVNDPTSLSVDEVLLSFELFVRLFPTSKADRTSQPDSLR

FVLLKFVGVPDYFNSFKIFKVSGIDNSSNNTSCVTITNHSNLIKFGHQFTVDKCVVQSIS

IDSIPILEKVFVAVPPTVYNILHTSPSDSVRSQFVSKLLSENGGVVSENDIIRSINGVAA

LCEPVSQGLVKGSTDIIFVKKNEFEEASFIDDNQEVADENIDLSQYLSSTLELDQDKHEP

EYPKFEVRPLPHDMSVSSRNSRHEDHELYIFINNRDIPSLGFPVFNGDLVKISSGDASIV

VKLFTLVEPNKSFSCGNLYLSPMLIINLNLNDRCEVTLKPCHSSSQNFSDIFPIAKTATI

SRVASPITMDRSYQQSFLSELKESFHSYLKVVRPGDFLPICIDSVLAKALFDVSLSQQSN

SNEEIDSDANIIPKGNPDSVAWFKIIELSGDGSSNSSQQFLIDPLQTALTSSGVEFIASP

ANKYSNWVDYLNLPPVFNYRTSFEDGSIFSYAQEFYRIITTDMSTCRKVDLKTTILLNSM

SRGLGKTTLVRSLAIDLGLNLIELDAFDFIKPGAELKSIGLLTGKIEKQLMGQPEDSSIY

HILYIKHIENLCVQSDENEQGANLSTSLSLKVVQTLQDCLARYRNMVLVVSCNNIDKLSA

NLRQIVKFQIDVSVPSEKERLEIFKYLLDIETRVLNNEMIPDAYEDRVEFEDKLDINKVQ

IDMRGDVSYQSLALQSAGLTPRDLSSIIRKAKQFAIKRLFKLAKNSHIELKKIIKVGNGG

HVVLIPEDFEQAINEARNQFSDSIGAPRIPNVKWEDIGGLDLVKDEILDTIDLPLKHPEL

FSSGLKKRSGILFYGPPGTGKTLLAKAIATNFSLNFFSVKGPELLNMYIGESEANVRRVF

QRARDAKPCVIFFDELDSVAPKRGNQGDSGGVMDRIVSQLLAELDGMSGGDSGEGVFVVG

ATNRPDLLDEALLRPGRFDKMLYLGISDTNEKQTKILEALSRKFQLSDDVDLAEISQRCS

FTYTGADFYALCSDAMLNAMTRTANEVDNKIRLFNEARLSEEKEPVSSRWWFDNVAGADD

IKVLVTMQDFSKSQSEIVPSVSAEELAHYLRVKENFEGGKSNPQQNGNNAEFERLESSSQ

SFEASLSNGHGQNLEAGFSNGHTT

>tr|C4XW29|C4XW29_CLAL4 Pyruvate kinase OS=Clavispora lusitaniae (strain ATCC 42720) OX=306902 GN=CLUG_00152 PE=3 SV=1

MVFASFIRTGNDIKEIRKVLGEDGKDIQIIAKIENQQGVNNFDDILKETDGVMVARGDLG

IEIPAPQVLVVQKQLIAKCNLAAKPVICATQMLESMTYNPRPTRAEVSDVGNAILDGADC

VMLSGETAKGNYPFEAVSMMHNTAITAEKAIDYLPLFNGLRSLALKPTGTTETCAVAAVA

AAYESGASAIVVLSTSGSTCRLVSKYKPDVPIMMVTRNSRAARYSHLYRGVYPFIYDQDK

VENWQEDVENRLRWAVAEAIELGIIKKGDSIVTVQGWTRGSGHSNTVRIVQA

>tr|C4XW31|C4XW31_CLAL4 Mediator of RNA polymerase II transcription subunit 1 (Fragment) OS=Clavispora lusitaniae (strain ATCC 42720) OX=306902 GN=CLUG_00154 PE=3 SV=1

PLFWNHSSTIDESDAELVERASKYTSKQSEYTINVVFSQANSLSFLRTRNDSSTVAEAIL

LKNLSNNVLGNFPTNLKYLTCLDSMSPLDGDLIVYLDNIALYLNAVHAMETNLNPGITDI

RSGWKSRFGKIYLNDPETQRLGVFLHFWKESRRLSEVMKHGATPCNSRVHKAILTIEEGE

SQSLDVLKEAKDQKWHLSTSQGLLQPYIFTFEDDSHLHNHQSVTSFSSPNWALHLRLDFP

VFMPSVHIEYFGITNYGQAKKPREPEVFKSLKEDGAVQFLARRNGENISISFTSDDTAEL

VAVESFEITKLTQLEKIIPTIRNYIVFSSLVDNVMQSPDTTFVASPKSSRPISPKVKGFT

KPFRRGYR

>tr|C4XW33|C4XW33_CLAL4 Acyl-coenzyme A oxidase OS=Clavispora lusitaniae (strain ATCC 42720) OX=306902 GN=CLUG_00156 PE=3 SV=1

MSSIASVKGTVSFSQGPEPKNLIQRERDAATFDLNEMNYFLEGSKERAMETRLFISQFER

DPVMAASPKDYDATKAEHRAITIQKIDRLSRYIEVETFDRLERRLSIVSVFDPSFHTRLG

VHLGLFLGCIRGNGTADQMQYWLLKKEAAYVKNLYGCFGMTELAHGSNVAGLETTATFNE

EDDEFIINTPHLGATKWWIGGAAHSATHCCVYARLIVKGKDYGVKTFVVPLRDADHNLMP

GITIGDIGAKMGRDGIDNGWIQFSNVKIPRFFMLQKYCKVSSDGEVTEPPLNQLSYSALL

LGRVIMVKDAFRWSARMVTIALRYAVGRTQFKAALAKESDPESQLLNYPLHQRRLIPLLA

LTYAFSNGANVLQETSTATMDELDDAVSSEDKQALDASVEKMKSLFIASGSLKSTCTWLT

LNTIDQCRQACGGHGYSAYSGFGKAFNDFAVQCTWEGDNNILGMSVGKQLIKKFAAVKKG

EKATGILDFLGGYEKLAGTNPVLEAKDLKEPKKILFAIEVALVRACIRADQILESKNGDT

DFIGAELVTLSKLLAHFFLLKAFLDKIEATKDQPNLVAVLDLLAQLYGATVILEGFSGTF

LTFSVTTTEAIAHNSTEHIAELCSKIRPHVIPLTDSFMMSDMMINAPIGYYDGNIYENYM

RTVQENNPAKNHKAPYSAALEAMLNRSSLAERQRFEREEEAEEILSN

>tr|C4XW41|C4XW41_CLAL4 Lysophospholipase OS=Clavispora lusitaniae (strain ATCC 42720) OX=306902 GN=CLUG_00164 PE=3 SV=1

MKITVLAFVVCCLAIEWPWEDGEDSSSVQSSVTTVQSGSVEFTKTIRTYAPTSTKCPSGS

IVREASSLHSSEKDYMARRHEQTNENLAAFLSDRARLSNFDAQAFIEKTKNLHNITIGLA

FSGGGYRAMFSGAGELLALDDRYEYSNKSGLGGLLQSSSYITGLSGGSWLVGTLVLNDWM

TVEEAISPDSGIWELENSIFNPSGINVFSTLKYYQSLRTAVEGKANAGFETSITDVWGRA

LSYQFFNPDTTYNGGENITWTSIRALSHFKNQSMPFPIIVANGRSPGTLIVNENSTVFEF

TPYELGSWDPSLNSFVDLNFLGTTLDNGRPKNNTCYTNFDNAGFVMGTSSSLFNQALLRV

QSSSTLNWAVKKLLTLILSPFSENNVDIATYKPNPFFNGEFGASETIASDETLHLVDGGE

DMQNVPLYPLIQKERKVDVIFAYDNSADVHNWPNGSSLVYTYERQFSPQGKGTPFPYVPS

VEEFMTSDLFGKPVFFGCDASNLTDLVGYHESEGNSTDVPLVIYIPNSYHSFESNTSTYE

MSYSPKEIHKFIENGFEVSSRGNYSQDRKWPTCVGCAIIRRQQERLGEEQSEECKDCFAN

YCWTGGLEDSPQRSIGPFKSADLTSASSAASKISSKSDSNFSSSSTDSTPDSSSGLSTSR

TANTNSQSSDSRSSSSRINGSQGKRMGKYIYVLALFANMAM

>tr|C4XW42|C4XW42_CLAL4 Small-subunit processome Utp12 domain-containing protein OS=Clavispora lusitaniae (strain ATCC 42720) OX=306902 GN=CLUG_00165 PE=3 SV=1

MVKSYDRYEQESSFGVIGGQSNIVWLPPAPTQSSKSLGRAASGGLEDILIWDIKTGELMT

RLNDDLTPGASNAKTSSAPSTVIVLAYHEQTNILAAGHNDGTIKIWDLTSGSVMVTFSGH

KSAISVLKFDRNGTRVVSGSADSTVIMWDLVGEEGLFKLKGHKSQITGMHLLSQMERTDD

ELEDYLVTVSKDGLIKLWELKSKQCVETHLAHSGECWALGLNSGKDMLITCGSKDQVKVW

EIDLDRDDGSKIVSKGEFEKQSKARCTDIGFTSVRDASGTYEIFALQNADRTTEVFRVRS

SEEIKKGITKRTKRLKDKGFEEDEILQSLRESEISMLITPFTTVRTTSKVKSCVWVPSNR

KNLDLLLALTNNSIEFHNIPLPEQIRKAQVGDIHSVKAHTIDNLGHRTDIRAMDVSDDNK

LLATASNGELKVWNIRSHNVLRTFSLEGGYALCCKFLPGGTLVVVGFKNGDLELYDLASS

SLIDRVENAHENTSFVSSGTKDDNGSAIWSMDLTPDGKTLVTGGNDKKVKFWNFKVSQEI

VAGTSTVVSSLKLAHTQTLEVTDEVLSVKVSPDSKFLAISLLNNNVQVVFYDSLKLFLTL

YGHKLPVLSIDISFDNKLIITSSADKNIKIWGLDFGDCHKSIFGHQDSIMNVRFIPESHN

FFSSGKDGMIKYWDGDKFQCIQKLPAHQSEVWALSVSRDGSFVVSTSHDHSIRVWSATND

QVFLEEEREKEMDELYENELLESLEGDDVTARKDEDGEEGEQDGVASVRKQTMETLKAGE

KLMEALDIGVEDLDNVEQYELQLQQYQSKKPGAFMPTKPTPNSILVAFGVTGQEYVLNTM

LKIRSTQLEDALLVLPFSYTVKLMRFISIWTNKQNITNNIVNLSLICKVLFFVMRTNAKE

LVSQRDEKLRNYLVEVKTQLRGKLVEASKQLGYNTEGLRFKRNQWKLNHETEFIDEAEQR

EYEEKKAVKRTFATV

>tr|C4XW43|C4XW43_CLAL4 Mediator of RNA polymerase II transcription subunit 15 OS=Clavispora lusitaniae (strain ATCC 42720) OX=306902 GN=CLUG_00166 PE=3 SV=1

MNNLGNGPMGGTWHSTYGISDRQKVIQILSTTLKEIQNGHYDEQKAATMAQEFEKYTFMK

SASREEYLRMIKQKVTQLRSGLMNNPNAQMASNVGPPQNDMGLQNGNFYNQGRQQMSPMR

NNLVPQQQQHQQHQQQQQQQQQQQQQQQPQQKQQSQPQQQQQQQPNRLSRSQPSASQSLQ

PTQQQIQQISFMIRTVPIPPALLAKVPNLPPNVNTWTQIYDCFQKKIIPASAMPIIKEIH

NAHFQHALRQHQQQKLLQLQRMNNTAGNDGVGQLGGENNQSLGSAGNFSASMNNMGNMNN

VSVMNNMGNMNMNTANNMGNMTNMGNMNNMGNISNAGNMANMGNMNNMNNMKMNNMNNLG

GMNNMSMKNVNNSNNMANMGNVAGGMSNMGNMAMNDVNRQIPRNAMGAHGPQIQQQNGYV

GVNKAQNSQNQQQQQQQQQQQPAGRVQMPQAKQQQQISTPASNQAIPVGQQQQAAKTPNI

QITSQDLLKYNSDAMALLGRLQANGSISPNLDQAQKQNFVRKYIYHQKLSLWKAQQKASA

INNPMNLPQSQPAPLQQAQVPPSSMGQGGFPQAPIQSQPQQQQQQQQPQQPQQLQQPQGQ

RRPRSSPVMQQPVPASAMNQQMYAQNMQQKSAPVQAANPVPQVRPAPLALAGGMPPLTDE

MKMKLRSLFEEVSRNNVQLKDVTMLLSEKDKAQVKETMARISQQYANVDSVLSYFYVLTR

NLEGTKRLIQMKHMTKSIMENLQRGIYLAGPDLLEKLRSQYQRYFEYVKEQITLSKQQQQ

QQQQQHPPNRPQNEPIPQRMNQMGPQGHFPTNPPGVQHNVVPHSQPVFNAQGVSGQMGIP

QSQQQQQQQQQHFARNSVSAPIPQQEWQNNSGTMASQNNPLASSPNMPQAGSPLQAPPQA

VGKSGAKVPTKKNIAAGNRRRSTKGAGTPAAGAPTPASLANAIKTPHSISTPQMPPAQSN

KGTPMGVSPNSEAKLAAQNEQMPFGGEMFGANGMDSDLMKRRELSKTNPKEFFFAALSNL

LDISDDEQDKGSGGTSAGNGSTTELKALTQSPLSPSNTGRWNCDVKPHAIASAFCQVNVI

KECSGPSILDECARIVELGASAPTKNGIKRERNEDDDIDLLFDEKKVKVEDSNNPSKRMY

EPLEYDEWVTWFKGLQETRV

>tr|C4XW45|C4XW45_CLAL4 General negative regulator of transcription subunit OS=Clavispora lusitaniae (strain ATCC 42720) OX=306902 GN=CLUG_00168 PE=3 SV=1

MSTRKLQQEFDKLQKKVAEGLQQFDDIHDKIASTENTNQKDKLEGDLRKEIKKLQRSRDQ

VKQWSGDSSNKLDRNVLQDIRSRIENAMERFKEMEKVSKMKQFSNEGLELQAKLGARGLD

EAKKLEATRYITDVLEELKRQNELLSADLAQYSHKKKSGGIQQAIDDLTEKIERNNFHVG

RLELVLRNLDNDQLEPERIDEIRDDLDYYVENNQAADFVEFNEFYDVLELDESLELSQSF

TVDDSPRKETPSPSEPKEKTVKVSDKEATKVKETKAEPKEPKVELKQPKPEATRVEVKEA

RSESRSGAVNEAKESKPEAKETKVEAREVRDTPTRAPPPGLAASPRKKAVASASPSPVLN

NENKTFWDTAGRPAQLAQHRLHSPLPFSAISSQLETSLLNCPDSFDSERPRHYNPTNVHP

SSVDYPQEPMYELHSAGVMRKFDTDTLFFCFYYSEGQDNLAKWNAARELSRRGWVFHRET

KQWFSQEQGKARKDEGYKYFDYQSSWLIRRKDQVEFGADARQTF

>tr|C4XW47|C4XW47_CLAL4 Transketolase OS=Clavispora lusitaniae (strain ATCC 42720) OX=306902 GN=CLUG_00170 PE=3 SV=1

MTDIDQLAISTIRLLAVDMVGKANSGHPGAPLGLAPAAHALFKQMKFNPRNPNWINRDRF

VLSNGHACALLYSMLHLYGYGLTLDDLKEFRQLNSKTPGHPEMHDTPGVEVTSGPLGQGI

SNAVGMAIAQKQFAATYNKEDFVISDNYTYVFLGDGCLMEGVSSEASSLAGHLKLNNLIA

FWDDNKISIDGSTNVAFTEDVIKRYHAYGWNTLEISHGDTDLDGVAKAIEQAKASDKPTL

IRLTTTIGFGSLQAGTHGVHGAPLKADDIKQLKEKFGFNPEETFKVPPEVYEDYAKHVKA

NQEIEAKWYQTFNEYKAKYPTEGAELARRLDGKLPENWTEALPTYTPEDKPLATRKLSEM

LLSSVFPKVPELIGGSADLTGSNLTKAANSVDFQDPSTKLGDYSGRYIRYGVREHGMGAI

MNGIAAFGANYKNYGGTFLNFVSYAAGAVRLSALSHLPVTWVATHDSIGLGEDGPTHQPI

ETLAHLRAIPNLSVWRPADGNEVSAAYKSAIESTSTPHVIALTRQNLPQLEGSSIEAASK

GGYTLVKAENPDIIIVASGSEVSLSVNAAKVLGEQGFKVSVVSMPDFLTFDKQPQSYRLS

VLPDGVPILSVEVMSSFGWSKYSHEQFGLNRFGASGKADDLFKFFEFTPEGVSERAAKTI

QFYKGKPILSPLNRAF

>tr|C4XW48|C4XW48_CLAL4 alcohol dehydrogenase OS=Clavispora lusitaniae (strain ATCC 42720) OX=306902 GN=CLUG_00171 PE=3 SV=1

MSIPTTQKAVIFETNGGPLEYKDIPVPKPKPNELLINVKYSGVCHTDLHAWKGDWPLDTK

LPLVGGHEGAGVVVALGSNVKGWKVGDYAGIKWLNSSCNNCEFCQTTNESNCPDADLSGY

THDGSFQQYATADAIHAAKIPQGTDLALVSPILCAGITVYKALKTANLKAGEWVAVSGAG

GGLGSLAIQYATAMGYRVVGIDGGAEKGEFVKSLGAEAFVDFTQSKDIVADVKKVTNGGP

KGVINVSVSEKAISQSVEYVRSTGTVVLVGLPAGAKVTAPVFDSVVKSISIRGSYVGNRA

DSAEAIEFFTRGLIKCPIKIVGLSELPSVYKLMEEGKILGRYVVDTYK

>tr|C4XW53|C4XW53_CLAL4 Acyl-CoA desaturase OS=Clavispora lusitaniae (strain ATCC 42720) OX=306902 GN=CLUG_00176 PE=3 SV=1

MSSDNLEAVDLTSLNALAAGFNKKAPRIVAAGLGGKLMGTKSLVDVTADQMTKDSETLLE

KDEREREKYMRTAHISEQPWTLSNWHQHVNWLNVILVILIPAFGCYSAYHYPPQLKTVLL

GALMYLFSGVSITAGYHRLWSHKAYDAAVPVRLFFAFFGAGAVQGSIKWWGHSHRIHHRF

TDTNRDPYDARQGFWFSHMGWMLSIANPKNRARADISDLTADPIVVFQHRHYLLLMVTAA

VLFPCLIAGLFWGDYWGGFIYAGILKFFFIQQATFCVNSLAHWIGVQPFDDRRTPRDHVL

TALVTFGEGYHNFHHEFPSDYRNALKWYQYDPTKVTIWLLSKVGLAWNLKTFSQNAIEQG

LFQQQQKKLNRMKNKLNWGPAVSELPVWDKDHFNEIAKEEGLIIISGIVHNVKSFIKEHP

GGQALVRASLGKDATKAFNGLVYAHSNAAHNMLATMRVAVIKDGEVNGDTFGLQEEMMAK

KDL

>tr|C4XW54|C4XW54_CLAL4 CBS domain-containing protein OS=Clavispora lusitaniae (strain ATCC 42720) OX=306902 GN=CLUG_00177 PE=3 SV=1

MSHNPSFSRSSQSPMSNHQQLHPHHGQSSSRKPSVVEMLSSPPPLPADQTQTSIDEISLS

RNTSISSRSSSVIQPTVNWSEILLADLTEANKLITINSSCSVQKAYETLVDHNLTSVPVV

LSQSDDGSTNYLSFDYSDLNTYLLLIMNRISVDDVLPSQDSDGRRPSLQERKDTLTSYIN

RAKKGEEVPVEIIVSLQPKTPFLRFKESDTLLKAVEVFGSGAHRVAIINNDNKISGILSQ

RRTIKFFWDNARRFTSLDYLLNSSLQDLKVISSNPVTIQGDCPLIEALEKMFTERMSSLA

VIDKNKSLVGNISIVDVKNVSSTNNSHLLFKPVTTFISYNLSQKGIEKGQDQFPIFHVNP

QTSLARVIAKLVATQSHRLWIVEARTSHYSVSNGPGATVETALAAESPVASAAATSIQFE

SGRPGKLIGVVTLTDILALFAQCKSGVKIDTQSARNQRRRSSTSTTRSSLDGVSGSITTP

TTPKQPSVPINQEMFRKSYQTGKGDNVFSNE

>tr|C4XW55|C4XW55_CLAL4 Elongation factor 1-alpha OS=Clavispora lusitaniae (strain ATCC 42720) OX=306902 GN=CLUG_00178 PE=3 SV=1

MGKEKAHVNVVVIGHVDSGKSTTTGHLIYKCGGIDKRTIEKFEKEAAELGKGSFKYAWVL

DKLKAERERGITIDIALWKFETPKYHVTVIDAPGHRDFIKNMITGTSQADCAILIIAGGV

GEFEAGISKDGQTREHALLAYTLGVKQLIVAVNKMDSVKWDQSRFEEIIKETSNFVKKVG

YNPKTVPFVPISGWNGDNMIEPSTNCPWYKGWEKETKSGKSTGKTLLEAIDAIEPPSRPT

DKPLRLPLQDVYKIGGIGTVPVGRVETGVIKAGMVVTFAPAGVTTEVKSVEMHHEQLAEG

VPGDNVGFNVKNVSVKEIRRGNVCGDSKNDPPKAAASFTAQVIVLNHPGQISSGYSPVLD

CHTAHIACKFDTLIEKIDRRTGKKLEEEPKFIKSGDAAIVKMVPTKPMCVEAFTDYPPLG

RFAVRDMRQTVAVGVIKAVEKTDKAGKVTKAAQKAAKK

>tr|C4XW56|C4XW56_CLAL4 Glycine cleavage system P protein OS=Clavispora lusitaniae (strain ATCC 42720) OX=306902 GN=CLUG_00179 PE=3 SV=1

MIRSVRLRNRVLPTVSRALLRSSPFIHSPRRCYSSPSTNTPGQDIPESHKCDTSSQNYAR

IYEPAPLGGLDSFQRRHFGPTPKDTEHMLSTLGYDDMDQFLAAAIPPHVLVKRPLQVQPQ

NGYTESEMQEHLASLAGENHIAKSFIGKGYYGTHVPPVVQRNLLESPEWYTSYTPYQPEI

SQGRLQSLLNFQTMVTSLTGLDVANASLLDEGTAAGEAMAMAYHHLRGRKPVYAVDTLVH

PQTLDVLRSRAEKIGVEVVELPLSTADGIAELAKLSDRLCGALAQYPATDGSIHVFSEVA

QIVHENKGLFAMASDLLALTLLRSPAELGADIAFGNAQRFGVPFGYGGPHAAFFATASSF

ARKMPGRLVGVSKDRLGKPALRLALQTREQHIRREKATSNICTAQALLANMSAMYAVYHG

PEGLKKIANRVYGLTAHLAQQISQTNHTVANSSFFDTLTVELSGVSADEILQEAFQKYNI

NLFRVDDKTISVSLDETATVEDVLNLGALFGKQIPVEKLATVPSEYLREDEILPHSVFHS

HHSETAMLRYLHLLQSKDLSLANSMIPLGSCTMKLNGTAQMQTLSMPGFTQIHPFAPSTQ

ALGYQKLVSEFERDLNDITGFAATTLMPNSGAQGEYTGLALIRAYHRSRGEYDQRNVCLI

PVSAHGTNPASAAMCGLKVVPVKCLDDGSVDLADLEAKAEKLSKQLCAIMITYPSTYGLF

EPGVRRAIDIVHSHGGLVYLDGANMNAQVGLTSPGDLGADVCHLNIHKTFALSHGGGGPG

QGPVCVAERLVPFLPSHRFADTPHATAESIRAVNSAPFGSAAVLPVSYSYIKMLGGRALP

YASALAMLNANYMMARLRGSYKIMFVGASSESAIRHCAHEFILDLREFKAAGIEAIDVAK

RLQDYGFHAPTMSFPVAGTLMVEPTESENLAELDRFVEAMLSIRREIEAYANGESWGRVL

KNAPHSMEDIVSTSQEDWEARGYTREQAAYPLPYLKTSKCWPTVARVDDTYGDMNLSCTC

PSVEEVAAY

>tr|C4XW61|C4XW61_CLAL4 Ketoreductase (KR) domain-containing protein OS=Clavispora lusitaniae (strain ATCC 42720) OX=306902 GN=CLUG_00184 PE=3 SV=1

MPIDILSTAIFDGPESIPGWQYFRTYAPYVAAAGAIKYYFGGASSNFDRELHGRVFILTG

GTSGLGAQVAYELASRGAQLVLLCRQTDDPWTIDFIEDLRTKTNNFMIYAESCDLASLHS

VRLFATRWLDNQPPRRLDGVVCCAADCLPRGKVRQATVDGVERQTGVNYLAHCHLLTLLQ

PALHVQPPDRDIRVVVVTCASQAVGELDVTDLTWSRRAYPATRPWTVFGASKLMLGMFAR

SFQRELCRYERKDKAPCNIKVSIVNPGIMRTPSTRRFLSMGTIWGLVLYVLLFPIWFLFL

KDARQGAQSVLWALFSPVLGAQDGGNLVQECKILTRGRKEYWDYDLQDELAAETQKAIEQ

LERQSAVERKRAGKSDKKVSEADLRDKPTSQEDLERKLEAMRSAMAPMGTGRASLFEPAR

RREKKA

>tr|C4XW62|C4XW62_CLAL4 Pre-rRNA-processing protein IPI3 OS=Clavispora lusitaniae (strain ATCC 42720) OX=306902 GN=CLUG_00185 PE=3 SV=1

MEEAVFYIQGLPEKKSEPLASATSIHSGQHVASFRAGAPRHGAALSGIGPNERLFVAGAS

AIIFSYLWGKESVDQRFPVPEQMACLAVANHPETVSDKTNVTSPSSSPLSAPACPWLLAA

GAPSGRLYVWELASGRLLCVKDAHYQALTMIQFSRCGTFAVTAGEDARVSVWRTSDLVTG

TAAPFATFADHTLAVTDICVDSGSLSVVSASRDGTVRVYDIMSKTLRSTFVFSQPVSSVA

RDPAGRALYAALADGTIRQVQMYTVNPHTHMMEAVGSGIVTVPADPELAYTFVHHGSAEV

TSLAMSMDGINLVSGDSEGRVFVTDVVTKQVVRAFTAGASVSHLEVGVFSREKKAAPARM

LIPLKRVVAAQDPLEHSVAVQIRADEHGSAAAKSAPDFAAWLEEKAHQQWEFAQEAPEQQ

KLQKLSAAYNALKENYEKLLAGKEEN

>tr|C4XW70|C4XW70_CLAL4 COP9 signalosome complex subunit 5 OS=Clavispora lusitaniae (strain ATCC 42720) OX=306902 GN=CLUG_00193 PE=3 SV=1

MVDLHRSLLMQASRDSSTDTLCSDSRERSALYSSSPANLYDIKTNPDELAARPWKKSARF

FENARISTLALTKMSMHAKFGGSIEVMGMLTGKIVGSSIVVCDVYPLPVEGTETRVNAQN

EAYEYMVQYLDLLKMVQREEHIVGWYHSHPGYGCWLSGIDVATQSLNQNFQDPYLAIVVD

PIRTIRQRKVDIGAFRAFPPGHASSKKSIRSPSHVAKSKRQDYGMHADQFYSLNISFYHA

VYDSKFIDTILDKSWVSKLLESIEGKNDYHRRFVSKVQELLKLFQEPILIESKEWRRMHE

LCEEFISRTLEPSSNASDSDSLQAILETGDEEDEEDEEDVEDEDVDDDNEEENDEKIEEK

MREEEMGTEMEIGKFKLTGIEKTREGEGTVGHDDDEEMSGSGKKRVFRASSTESRSSAIQ

GLSRKRFAASEGALSQQLIQKMSATQAELNAWRDMTKNVGKAEFKSFLAAKVQRQVFGGL

>tr|C4XW72|C4XW72_CLAL4 C2H2-type domain-containing protein OS=Clavispora lusitaniae (strain ATCC 42720) OX=306902 GN=CLUG_00195 PE=4 SV=1

MPFAKIKIKNNESIRLGERRCFPFSTQPHVTEDANPHAGKRCWLLHFSSTGLECRPLHFS

ERFFFLTPYFFVRTSSAFFFVDHPIFFSRLWIPSNFFLSPALAAPCTKKKSPRRLLLFFF

AGPALPIANYSLRFSVMMSTKQEPRRKDPASRPYKCPMCDKAFHRLEHQTRHIRTHTGEK

PHLCSFPGCNKRFSRSDELTRHSRIHTNPNSRRNKNLSKVVATHQVTPSPQTKTVSVPIG

SAPVGATPVSASAAISIPFSAEADSSKTSGDEPMHSLSSSASSTASSIEKPDTHSDQPST

STRSESNSNTRPALRGPSRSTMNIDLLASAATEELKVLKKGPSTHSGGIDANSRSLPSLT

DYFNTAKNKSAVFDPGHNLQYLSKMALSSKNNSGLASPKAFGTLSSLQKMTPLNPAVTVH

QPTPSRAKIMEDSDLEYVQQRLKKSRPNSPAPNFTLPNSPILGLSTSNTPLMSANSSSTN

LSSFFMTPMNSVVPTADKTPSMSRQTTRTPPPSGAADVHSPGYELESAAPTNLPPLRSLK

LDLPKNLSMAGSAGPQILMGTRPEPASLSHQGMPSADRMTDD

>tr|C4XW73|C4XW73_CLAL4 TOG domain-containing protein OS=Clavispora lusitaniae (strain ATCC 42720) OX=306902 GN=CLUG_00196 PE=3 SV=1

MSPLCSSQESVTKIFEFLVASEALGDKNELVAQELLEAGIEIITLHGLAHVESLVPIFEK

CLAQADTGSKRQDRLRESVIILYGSLGRHLKADDERLHLIVERLLQTLDTPSEDVQYAVS

ECIAPLSQAFEPNLQAHFDNLFAKLWSGSSLAVRKGAAYGISGLVKGAGIRSIYAFDVMR

EISTAADDKKPESREGVAFVLDCLSQSLGAYFEPYVIETLPLVLKSLGDASPGVREATDM

AARQIMKSTTSYGVKKLIPVAISHLAEHAWRSKKGAVELLGSMAYLDPAQLSASLSTIVP

EIVGVLGDSHKEVRRAADQALKRFGEVIRNPEIQAIVPDLIRAIGDPANHTDAALEKLIR

TQFVHYIDGPSLALIIHVIHRGMRDRSAATKKKACQIVGNMAILVDARDLLPYLAALVAE

LEVAMVDPVPATRSTGARALGSLVEKLGEDQFPDLIPRLLATLRDPARAGDRLGSAQALA

EVICGLGLAKLDEMLPIILDAAQSPESHVRAGFMPMLLFLPVCFGSQFAPYLSRIVPPVL

AGLADSDEDIRSTALRAGRLIVKNYASRAVDLLLPELERGLADSSPRIRLSSLELTGDLL

FQVTGVSGKNELSEEQVEVGRALVDVLGAERRDRVLAALFVCRADVSAPVRAAAIDIWKA

LVANTPRAVKEIVPSLTHAIIDRLAGGDAVSRQIAAATLGDVVRRVGSSALARFLPTLED

ALSSGDAPARSGICIALSELVAAASEEAVLAHQDTFIGIVQKALVDDNAEVRAAAAQAFE

ALQEKLGKTVVDSIVPGLLAQASSNPSALSALEELVSARAELFGTLLASLLAPPLDAPKA

QALASLAEAAGSAVYSRLGSIVGALVQAISSSNDKESKDGSVEVLETSLDRILLCADDSG

VHAVMSQLMHFARHEDSNKRAVAGERMAVFFGQTSLDYSSYIPDLVSQLVLGLGDADDRV

VRANAAALLSLVRAQDKPQLARMVRPAHDALNLAGVRDEPLRAFVLGGPACIVPVFAHGL

MYGTGDQRELSAHAIAQIITRTPPEKLRPLATTLAGPLIRVIGERVPPGAKTAILGALAA

LLEKIPQFLRPFVPQLQRTFVRSLSDAGNSELRAGAVSALGVLVAFQPRVDSLVTELVTS

AKTAEPPIRQTMVQAMLQVVARGGTHMSEQSKAAVMGLVEGELGSLEDGAPAVAYARLLG

AVARVLSPEEAAALVRDKILKDGASPFSVLAINSFLREAPSHIFDTGVLGDVVDFVVAAC

ASSQPYISDNAVVALGKLLLLDGKERDDNSSGASFMLGDHLRTLVCELARCAVQPASNSP

DTRRLALVVVRTAARHRYEALKPHWDVLVPAVFSCLRDAIIPIRLAAEKAYLALFNLVDD

VDMKDFTEWFASVGEIKNAVGTVFQPRSIGDYTKRVAGRLAGVEREKISAGGDAEAMFSD

RYEDEKEIWAVGGC

>tr|C4XW76|C4XW76_CLAL4 C3H1-type domain-containing protein OS=Clavispora lusitaniae (strain ATCC 42720) OX=306902 GN=CLUG_00199 PE=4 SV=1

METLHRQLHFPSFSDVKQPAFNITSYTTTSSPSATASSTFSDSQDDLLPDLWLEEPKKYA

SGFPTDFGAPKMPDANNYNTTAMLREASLEYAQSLLPHSLLAPLTEENLDYNDSLSGEQF

FRAGAHAQKKCSHEPHAEHARPNNTHNAPKARRGSSSSTSSKGKNINTQLYKTELCVSYM

KMGGCPYGAKCQFAHGEHDLKSVPRPANYRSKPCSNWAKYGSCRYGKRCCFKHGD

>tr|C4XW79|C4XW79_CLAL4 E2 ubiquitin-conjugating enzyme OS=Clavispora lusitaniae (strain ATCC 42720) OX=306902 GN=CLUG_00202 PE=3 SV=1

MSHPFARRLQKEYKTLSRNPLPKVTMLSESDMTNYKFEMAIDNDIYAGQKYLLQVNIEND

YPVDPPIVRFGRSDDYVVPLHPHIYSNGHICLNILGKDWTPACSVESIVLSIQSMLYNNE

LEERPPDDDRYVASAPKNPKHTLFAYHDDNV

>tr|C4XW80|C4XW80_CLAL4 Zn(2)-C6 fungal-type domain-containing protein OS=Clavispora lusitaniae (strain ATCC 42720) OX=306902 GN=CLUG_00203 PE=4 SV=1

MTSVKQEPSSPPFFSMENLSNLPSESLAEEKFDALDLGELKHSEIDSKPEQQLKQPQLEL

THPELDLKQPLVDQTPFTVPVMANVPSQAPPVALNASVPPPVLQSNMHSAKPAPAADTLT

QSGSKDECNSKEESDMNNLNIEQACDSCRKRKLKCSKEYPKCSKCIQHNWCCSYSPRTVR

SPLTRAHLTEVETRLTRVTQMLRFLLPPGVDVDKLMESGRFEGALRPFREKLRGSEPTSL

SPSACSVFSGEESLNGSLPKKSLTFDRGYDKEKIKREIIDDFVLNNIHTDKRPFGTENRS

GSELGPEMAMLGAGSKQVSSFESTNPVSLTSPSSLLSLDSFDYEVDEELDEKCIKKQKTH

PSEYTSIFDEVMCDFT

>tr|C4XW87|C4XW87_CLAL4 Transcription initiation factor TFIID subunit 8 OS=Clavispora lusitaniae (strain ATCC 42720) OX=306902 GN=CLUG_00210 PE=3 SV=1

MESDKPNIAHRTRNASRKDTVEKPAPKPEKRRRKPDYIPPLSRRLGPYTNLVKPPVSSLE

VSGPIDLAFKKITGLLVKNTAPDFSSEFLCGVSNLASEYMCHLFTSLHELTEVQRHSRPG

VADLQMCLDQHYLTPTDLYHQYERTLQFPKELRPTIAMLKAQVEKSQSEFYAENYTLDKD

DPSLVFHANEQYEIAALVPRQSKKRNYIPDYLPDLPPDYTYQDTGHYMSTLTDLKQIKLK

LVEESRLNEASLYKLIDNENPLRDIDKDISMSSYSDLESDDDGIMSDVGARSISDVESPA

EVTDKSEQRPEEKLDERSQEKKEEKAENILEKMAEDSEENIEAKQDEPQEASSLDTPQPI

VHKDKKFDFVGYAQKCRLATERKAKELELRRQKRQKDIFLRAEKSFSCYAEGPPSLSERE

YFKKVLDGGFKKVIRATRIAERKKKEKIAELLARKAQAEQELEKDNAFEFGFAFNHASNL

SDSEEEDMPIEFDFGDEKDEVISEKPSPGQLAGKAPPNNISDNNVQDDGERGDIQETAES

NHTDDVDDMSKKMNDELDSVLNAAEESTAGVHSDWQMALGADADSDEDELEDL

>tr|C4XW88|C4XW88_CLAL4 RNA polymerase II subunit A C-terminal domain phosphatase OS=Clavispora lusitaniae (strain ATCC 42720) OX=306902 GN=CLUG_00211 PE=4 SV=1

MTVIYLPPSAPFPVTVTAVLCRVGDTIQRHAPVIKYKYWDYQDDPNSKDDPPPKIRVERI

GTFESPIEGEVTELSIAVNDEVAHCEVELCQIEEPCSHTVQYGGLCALCGKAVEDEKDYT

GYNYEDRATIAMSHDNTGLRISLDEATKIEQSSTERLAADKKLILVVDLDQTVIHATVDP

TVGEWQRDPQNPNYPFVKDVQLFSLEEEPIVPPGWVGPRPPPTKCWYYVKLRPGLKEFLA

EVSKLYELHIYTMATRNYALAIASIIDPDGKYFGDRILSRDESGSLTHKNLRRLFPVDQS

MVVIIDDRGDVWQWEANLIKVVPYDFFVGIGDINSSFLPKKNGQLVGPTKKRKTIARLEA

MEEAEESENELREESATPDRADSERSDDAPESDAPDSDSEGSSPVERILEMGGGENNHEL

LIEQRITRSQSIEQQQHERPLAKLQHNLDKIIHDHETPAPESDGKEEEEDNLLYDDDSEL

STLQTALEKIHQEYYKLYAKYKSNPGLPRPDLATIIPRLKSPCLSGVVVLFSGILPLGMN

IDNADIVIWCRQFGVKVVNEVYPDVTHVVCRDPESSLMKPGLTLKVRVAKKVLPDCKIVN

PDWLFACLSSWGKVDEKEYLINVPSQDWYVSESDLEKYMNSLETSKRSEQTPALEMSSRG

REGSLSSIEEYDFNTASAEVDDFLAGISDDDDDDDEGNDDDDDEADMNGDAELEGEKAPS

GGKSTFLQSLYAAPSRKRKTLDDDSDEEIESKKKPKEDNFDELEQELLDELNELEE

>tr|C4XW93|C4XW93_CLAL4 Protein YIP OS=Clavispora lusitaniae (strain ATCC 42720) OX=306902 GN=CLUG_00216 PE=3 SV=1

MDNWQSGPSADEFIIPDEDIPNPNQTSNVPRGVTNQPQPTNSQSGFSWTPKVDLSTAFTP

FITPALPDSNKVSERQYSGGDTLDEPVWHTLKRDLLQISKRLAIVIWPAQLKQLAQKHQA

GLIDLASSNGIRLPASIINASRAVPEDDEEENSSAMVPALDWDLWGPLIFSLAYSVTLGF

AAPNSQTNMVFSGTFSFIWVFYLIGGLNIQLLGGTISFLSAISASGYSMFPVVVGAVVNT

LAIKWRWLRLLIMCFLTTWSIYAAGMSLRCSGVLPGRVFLAMYPVALMYTVLAYLTVIT

>tr|C4XWA0|C4XWA0_CLAL4 alcohol dehydrogenase OS=Clavispora lusitaniae (strain ATCC 42720) OX=306902 GN=CLUG_00223 PE=3 SV=1

MRNAIPKHIRHRKSSNGAACRTQNIYKWGIRASCTANMFSVCEVVTRTKCWPSVAGLSLA

QSSIRRLLSSTHSSLASSALARSYSTAPTIPSTQKAVVFDAPRAPLVYKDIPVPTPKPNE

LLIHVKYSGVCHTDLHAWRGDWDFPVKFPLVGGHEGAGVVVGMGHNVVGWKIGDYAGIKW

LNGSCQQCALCETGNEPNCSHASLSGYTHDGSFQQYATADAIQAAQIPPGTDLAAVAPIL

CAGITVYKALKTANVKAGDWVAISGAGGGLGTLAVQYAKAMGLMVAAIDGPKKADLVRGL

GADKFIDFTSTDDLVGAVREATAGGAQGVINVSVSPKAIAQSVEYVRPTGTVVLVGLPPG

AKVELPVFNAVLKSITVAGSYVGNRADTREALDFFARGLVHSPIKMAGLSELPRIFEEME

KGEVMGRYVVDTSY

>tr|C4XWA2|C4XWA2_CLAL4 Glycerophosphocholine acyltransferase 1 OS=Clavispora lusitaniae (strain ATCC 42720) OX=306902 GN=CLUG_00225 PE=3 SV=1

MSGSSREEVVGKSTGRARFDEPFRRSSASEQDLIDLSCAVEEAEDDESDAMSVASTSLNR

SDSTSSVSFIDLSKLLLIPEFDFGTGFDQMNTSTKKRLNSMKNKTKETLRRLKEDRSASF

KTRKFTQQELIKFQESMNRRLANFDKRVHNSLQSSATEKLFYAFAVSLIAAAGYIIGKYP

TYFPIFHTVLFCFLMPIRFYTYFKIGFQYYLADLCYYVNVLLLLFLWVFPRSKSLFISVF

SLTMGTLSFAVITWRNSLVLHSIEKTTSSIIHVMPPVTMFVLVHQTPKSYIQERYPAIAE

VENWNFVNGIIWTSIYYTIWQVGYHYFITVRRREQIAKGRVTSFTYLKEKNSKSFLGRLV

NKQPYAWMQVTLFTLIQFGYQILTMLPCPIWFRYKHVCATFVSFIFSWSAYNGATYYIEV

FGKRFEKESKQLQIELAELQSRVARQEAERSTPSFSPVSESIPSAESFTELKLDSPRVSA

SEDVI

>tr|C4XWA3|C4XWA3_CLAL4 S5 DRBM domain-containing protein OS=Clavispora lusitaniae (strain ATCC 42720) OX=306902 GN=CLUG_00226 PE=3 SV=1

MFRTTLSRLAPANRCFSSGRAVLQNENKKVTKHIDFLSKFYSPELLQSIKITESLVDPKE

YLEMKKRGGHEISKVAPSNPVEDYSKSDPKWEEAILYPNQANNRTPYPEIPQIQGADRTD

LKLRFDTAAATTTKGGPAVNDQFFDQLHKLTGMEPWYMKKLYVRPIVMKRVSCQTSKGKI

PNFYALTVVGDKNGMIGLGEGKSRDGMRTALYKAHWNAIKNLGPIPRYENRTIIGDIDYK

FHAVKLFIKSAPAGFGLRVNPNIFEVCQAAGIKDLRGKVYKSRNPMNVVKGFVEALTKQT

SLEDLAAGRGKKLVDLRKVYYSA

>tr|C4XWB1|C4XWB1_CLAL4 D-lactate dehydrogenase (cytochrome) OS=Clavispora lusitaniae (strain ATCC 42720) OX=306902 GN=CLUG_00234 PE=3 SV=1

MFSRSFRTSVRLCKRHWPGKAPGLGSRCYSTQSSSSVSGVKVGAALFAGVAGGYFGSQLY

SQTPAKTVHADAKAGKESSDKGSVDASTTPLDTLDSPKYASDEEFAAALEQFRAIVGEEN

VSSSKEVTDAHADTFFSTHHPPEPEKQRPAVVIYPANTEQVSAILKVAHRYRVPVVANSG

LTSLEGHNMHTRGPNSVSLAFGNLNDVVEFHPDDMDIVVQAGLGWQELDQYLLDSEDGKH

LMFGPDPGMGATIAGMVGTSASGTNAYKYGTMKENVVNLTVVLADGTIVKTKQRPRKSSA

GYDLTHLFIGSEGTLGVVTEITVKLQPRSFRELVCIASFPNIRDAAATAQHIISKSGIHA

NAIELVNETTMSFVNESGAVAKKFLERPSLLLKIGGPSESVIRQQLDIVEQIARSNNLIQ

FEQSQNPEDNEVLWGARRAGLWSTFEYGKKVLDDKDDVQIWTTDFAVPLSKLATLIDETN

DDLIASGFRDRFSVLGHVGDGNCHFLLLYNSKDYDRAKDVVDRMVGRALKYEGTCTGEHG

VGVGKRSYLEPELGVNAVDLMRHIKFALDPRAILNPDKVFKIDPHDKLDELLSSGHVHVE

SKCC

>tr|C4XWC1|C4XWC1_CLAL4 Fork-head domain-containing protein OS=Clavispora lusitaniae (strain ATCC 42720) OX=306902 GN=CLUG_00244 PE=4 SV=1

MSRKPLFSQNLNVPISAAETVAETPATHLSTKITTPPHSVYAKRNHSVLDTPVRTSDASS

AARSLLMSPAFSSPQQQQPPVFQNPSSLMKSPRCMAPAFSETAPFSAPAVSGLECAPVLE

DGKKKRPKKTKRDAREGFSLDSNEKPPYSYATLIGMSILTHPDKQLTLSQIYTWISETFK

YYRREDVGWQNSIRHNLSLNKAFVKGAKSKDGKGHFWCIKPECEDLFLKAKNNKKSSYHE

VMDQLATARQQAISMLPSSPPPQAHDERKRPSDTEGENPSKRSRISTDYESDSTGPDFDS

SLLRTPAPALVVSESPAPLLAGKHLTFASSFSCSSNFELSPVQPLETGPLLEPLPPQNGA

FSQSLPAITLNRVVSQHLPQLQPPIPTFTTPRSGVARTPKSGAKTPLRTLRTPSSGTVMR

KLWHSPSYLEEFYYSPFGNTRAVLNSYDDDDMLMRAFDSPRAPGRASLLHELRKADSDPA

EETDVDQTG

>tr|C4XWC3|C4XWC3_CLAL4 Metal homeostatis protein OS=Clavispora lusitaniae (strain ATCC 42720) OX=306902 GN=CLUG_00246 PE=4 SV=1

MSNTNRSLGAYAELRGVSDVDSDSDIFLEAPDSMPQETEAIRNFGGAPEVNSSPDQNPSS

VSTPDANQEHLDRPPDSSNQRVEGSSSNIAVFNNNLQADGDLERQQERGLSAKQKAMKIV

HYIFPFRQTYERLHNGINTGLLQTNNPGQFVGQGTDGVFRNLMAKPDTETTRIAQEQHPP

TYEEAAADATPEYWESTMMSPIYEDEVFVQGLPVGNIANFVWNILVTVAFQLVGFLLCYL

LHTSHAAKHGSRAGLGIYLVTYGHSRIPANLGRPDKIPPRYIPDNPNSLDISKSSSIKSG

GRVDKYQSGFQQHSDSLAREVSSREPYFAYGVIAFGIFMVLKAVYDFYHVKQMERSMLAP

QAQPETHTSTEPADGPSED

>tr|C4XWC9|C4XWC9_CLAL4 ATP-dependent helicase OS=Clavispora lusitaniae (strain ATCC 42720) OX=306902 GN=CLUG_00252 PE=3 SV=1

MLRKKNNGPDGNDWKRMFHLISSMSDGVKREIDIASHGGDVAVIDGEEEEEDDNSSEIQL

TCPICLDVVGSESILIFAGCGHMICDGCSLSFFEQQEIENESTGSDKACCMTCHHDVAVA

DLIEFPLYQKVVYDGYTYDRLNLVFDTNKKKKTSNMDKIANLIRQHKGFLPSAKMNKTLD

LIKDITTSNLDEKIIVFSHFTTTFDLMGYALKQENIKYLRYDGSMNIDSKNATIKDFYEG

NTRVLLLSLKAGNVGLTLTCASHVIIMDPFWNPFVEDQAMDRAHRFGQMKPVHVYKILIR

DSVEDRIMDLQERKKELINAALDEKELKNSSHLGRRELGYLFGLNTLGQIV

>tr|C4XWD0|C4XWD0_CLAL4 Helicase ATP-binding domain-containing protein OS=Clavispora lusitaniae (strain ATCC 42720) OX=306902 GN=CLUG_00253 PE=4 SV=1

MSAQAYDVIVISSDEEDAPNVSISHNFPPSQSEDFHFDSDFLSEHRPDAPHDSTQVESTA

RAAEADSHTQSTSAEEILLGDPQILTQMIQDPPDHSLGQNSMSQYKPDTSQSQMQQGGLE

TYANPTFSNGSMFASSEQRPQSRASPQNQDANVVVVDLSDDDMWISEEEGIQASAISNPQ

ASVAGHIHNSDAESLRRDVREIQNAPPIANGMDSDSDDEIAVLSKEEAERTGAFKASSFE

APAYNQPAPVFHAGSEASSSMANRYEELKIREYFERLSMQQLYQHEHNLHQQLRTLDETR

DVNLRHVADLRGRLSNLTYDQISLRAAMMRNITDSLRETEQIIENSKKVRRYRAVLQSVK

EYRQVPQHARQPSSPQNFPFQLSNFSFNVVPKMADYYNGMPNGPNATNSYGTNVNPYLVN

DEDSVHLRNLFNDIYKEETVQGMAPTPESLSIQLLDHQRKGLFWLLNKEEANSGCILADD

MGLGKTVQTLALIAANKSEDSSCKTTLVVGPVSLLRQWAAEFQSKLKIEHRMKVGFYHGQ

EKKKLNTFQKMARCDIILTSYTTLASEFKQHYDKVTEESLITKGQNVLPDLYSGGQNYVS

PFFAREARFYRIVLDEAQYIKNKLSQTSKATACLKGKHRLCLTGTPMQNSIDELYPILRF

LKVRPYDEESKFKRDISVPIKSNADEMSDYRKVQSMRKLRAVLSAIMLRRTKDSTVDGKP

LIELPKKEVKSVFVSMDQDEKKLYKDLELGIQKKARKLLQRHGKSSHTDI

>tr|C4XWD3|C4XWD3_CLAL4 histidinol-phosphate transaminase OS=Clavispora lusitaniae (strain ATCC 42720) OX=306902 GN=CLUG_00256 PE=3 SV=1

MSFDLSSLVRENIRTLEPYRCARDDFKEGILLDANENTHGPALTSLTSWEEQLELNRYPD

PHQIELKKQTVDFRNRTPNKYLDNYEENKLTPENLCLGVGSDESIDLLMRCICTPHQDKL

LICPPTYGMYNICATVNAIEIVKVPLTVPEFQVDAPAVLDAVKKDPSIKLIYLTSPGNPT

AKLLDVDILIDILKSAADSWNGLIVVDEAYIDFTWDPESPNSKSMSTLVNQFPNLVVMQT

FSKSFGLAGIRLGITFASRELSRYLNAMKYPYNISSLTSAIATRSISPENGLRVMETYVG

NIIKQRNEVLKQLQEIPGVGKNIGGTDANFLLLQILDKDGNPSNDIALKLYHMLAVENKV

VVRFRGKELNCTGALRVSIGTEEENQVLVKKFAQCLASIR

>tr|C4XWD5|C4XWD5_CLAL4 Potassium channel domain-containing protein OS=Clavispora lusitaniae (strain ATCC 42720) OX=306902 GN=CLUG_00258 PE=3 SV=1

MDTNNLRKRLTAAKESLGISKNNTVSGLTSTSLARYQDKLRPLFSDEYYQPLQGINENDT

EDVFMPRAAAVLNLVLRAPILTITNLSVQPGERHFVLWLLISSYLPLISACIAPLANLIS

LVGLVEHWRVNGQTHRSVPDQTPVFALNIFSFVLGIVGNSSLVINFSGKMRYIVTHIVSI

TCWVCAASVLLAAVLISNREFNEDTFYKRSEGFWLAALTIFMYFCCMMTILINLIGYKLR

KYPPTFNLDRKERRLMIFTIAFSVWEGVGTLVMAHLIPGINYGSSLYYCTVSVLTIGLGD

IVPRSHGAKVFALIFSFIGLIIMGLIVAMIRQVVSSSAGPSVFWHLVEKRRVLLLKELRE

RNEPMTREKSFHLMRLLRKRVRIHQLNMSLALSFLTFIAFWLIGAMVFHFTEKWSYFNAV

YFCFLCLVTIGYGDYKLETNFGKVFFVAWAITAVPMMTILISNVCDTLFESAGRLHTIKK

RIFDPKTYYLLLTPSFYFRKMSRDNLEVELEEEEQREEIEENEIQALVEESSGTAISAAQ

LSEEEVDPLTPRQTLEHYQKRSEQILKRFSAMREALLDSVNSPDKKYDAREWKELLEVLE

PEDGGDVDPYFWLGKDSPLRLPIKEPNFALLKIFFRVESDFKGLLAMQKEELRQLNKKVD

S

>tr|C4XWD8|C4XWD8_CLAL4 2-(3-amino-3-carboxypropyl)histidine synthase subunit 2 OS=Clavispora lusitaniae (strain ATCC 42720) OX=306902 GN=CLUG_00261 PE=3 SV=1

MSAKSISTYSSPANRQKTIVIQSMATAPTLSTPQDESVFYAKVAVSKPRRRSNIAQYYSL

DELADAVKKYKRITLQFPDELVGDSAAVAYELQSRLDKASALLQKEDSETGNGQAASQNE

NDKTAEAKETSECRGDVDPAAKTPKQQCSSSTCSTGNNSCACSASCSSSCSNSDAKTSSQ

VWILADTSYSPCCVDEVAAQHVSADVVVHFGDACLNPVASLAAVYVFGRPHIDLDVLVTR

LREKYTPDSQLVLMADTPHTPLLHELQALVPELNTTVADIYIQDKSSVVGYKPRVVQEDD

LRTLNRVFPGISDEAELQNYDLFHISEPKAPRMLQLTTKFQSVTTVDAETGDFSSNPAPA

LARRYRYMHMARSAATVGLLVNTLSLAHTRTLMNSLASQLREAGKKHYLFVVGKPNVAKL

ANFDAVDMWCVLGCDHQGIILDETREYFKPIVTPYELVLALSDELTWTGQWVTDFQEVLT

LLKDENEDNNQEGNSNSMNDESDGDDEPEFDSVTGRYVSARPLRRLRHLQIEQEAEEAPD

PNTAQQNGSSESGASNGKKPSNQLVKKLSSAVAVRGTYSTSAAHLQTREWTGLGSDWTQD

EDDEGAEVEEGRSGIARGYDFDRENRH

>tr|C4XWE3|C4XWE3_CLAL4 Na(+)/H(+) antiporter OS=Clavispora lusitaniae (strain ATCC 42720) OX=306902 GN=CLUG_00266 PE=3 SV=1

MKKHWVSVTILLFPVMTCGWLIVGLFIWILIPHFNFPSSLLVSACVTATDPVLAAAVVGK

GKFAERVPGHLRNLLTAESGCNDGMAFPFIYLSLNLITHSGNAREIVKEWFTLTILWECL

FGVVLGVFLGYTLRKAVAFAEKKGLIDRESFLALFVFLAFNSAGIGSMLGVDDLLVSFAA

GTAFGWNGDFARKTEESHVSTVIDLLLNLSFFVYFGAIVPWPDFNNSEIGLDVWRLIILA

VVIIFLRRIPAVLALKPFTPDIKSWREALFCGHFGPIGVGAIFAAILARADLEKHYTHEE

TPLRKVPDKSFPHYQLIASIWPIICFVVITSIVVHGSSVAVLTLGKRLNRMAITMSFSVT

NTQDQGPSWISRLQKLDKTSTSFSLHRIDTTTVSDKDEQLSRTNTVETSGVKVRPAGGAK

RKKRGKRSKKSTKHEEIGSSPTIERQRPQTEFLQLGKTPSRHPSNLDNEDESPGSTTIAN

SELQTTNETKEIPPEALEEVKKKVSEIQREASGTSQVPTTVYQEGSRVVVEDQDGEIIDS

ASLIDYDKERPDLGRGDAPDGSDMSIHSMGSLRRHMSAFSALSDTSNASGTESMTSKLTR

TLSSNVRKAFVKKDGKGKGKKYYAHQVDNLLLIENADGEIVRRFRVNKHESSRARSRSIV

GRALSLVGIKTRDSATSASGAPSGSTSAVEPSEVKNKLTVPPTVHEDSDRSYGDSESEFE

TEDDFSETEVEKRRRLAALGEISDGRAESDEEE

>tr|C4XWE4|C4XWE4_CLAL4 Ornithine aminotransferase OS=Clavispora lusitaniae (strain ATCC 42720) OX=306902 GN=CLUG_00267 PE=3 SV=1

MSLSSETARKYEEQYSARNYHPLPVVFHKAKGAHVWDPEGNEYLDFLSAYSAVNQGHCHP

KIIDALVEQASKLTLCSRAFSSDVFGEYAKYVTEYFNYEMVLPMNTGAEAVETALKLARR

WGYVKKGIDEDKAIILSAVDNFHGRTLGVISMSTDPEATTNFGPYLNGVGPQIPGEPKGT

LLRYGVIEDVQKAFANAGDKIAAILLEPIQGEAGIVVPPEGYFSQVQELCKKHNVLLICD

EIQTGIARTGKMLCYEHYAGVKPDIVLLGKAISGGVYPVSAVLSSKDIMWTLTPGSHGST

YGGNPLACKVAMAALQVVKDEDLVSRAERLGSLLRSKLEELKSEFDGIISEVRGKGLLSA

IVIDESKANGRTAWDLCLLMKDEGVLAKPTHDEIIRLAPPLVISEEDLLKGVDAIKKSLI

KLPTAAKADHH

>tr|C4XWE8|C4XWE8_CLAL4 Homocysteine synthase OS=Clavispora lusitaniae (strain ATCC 42720) OX=306902 GN=CLUG_00271 PE=3 SV=1

MPSHFDTLQLHAGQEVERPVGARAPPIYATTSYVFNDSKHGAQLFGLETPGYIYSRIMNP

TNDVFEKRMAALEGGVGALAVSSGQAAQFLAIAGLAHNGDNVISTSYLYGGTYNQFKVAF

KRLGIESRFVNGDKPEDFAKLIDDKTKAIYLESMGNPKYNVPDFEKIVKIAHENGIPVVV

DNTFGAGGFLVNPIKLGADIVVHSATKWIGGHGTTIAGVIVDSGNFPWAKYPQKYPQFSE

PSEGYHGLVLNEALGNQAYIGHVRIELLRDLGPALNPFGSFLLLQGLETLSLRVERQSDN

AMKLAKYLEKSPYVKWVSYVGLPSHESHELAKKYFNHKEYFGGALTFGVKDLETQVDDPF

QEAAAKFVDNLEVASNLANVGDSKTLIIAPYFTTHQQLSHEEKLASGVTKDMIRLSIGTE

YIEDIIADFEQSFKKVYGA

>tr|C4XWF0|C4XWF0_CLAL4 RNA helicase OS=Clavispora lusitaniae (strain ATCC 42720) OX=306902 GN=CLUG_00273 PE=3 SV=1

MPDVKTDVFYGGTPIARDLEKLKNKDTCPHIVVATPGRLHALVAEKGIRLGNVKSFVIDE

CDKVLESLDMRRDVQDIFRNTPHQKQVMMFSATLSQEIRPVCKKFMQSPLEIYVDDEAKL

TLHGLQQYYLKLSEKEKNRKLSDLLDSLEFNQVIIFVKSTQRANELNKLLCACNFPSIAV

HSGMKQEERIARYKSFKEFNKRICVSTDVFGRGIDIERINLAINYDLPSEADQYLHRVGR

AGRFGTKGLAISFVSTKEDEEVLEKIQSRFDVKVTEFPEEGIDPSTYMNT

>tr|C4XWF3|C4XWF3_CLAL4 Pyrroline-5-carboxylate reductase OS=Clavispora lusitaniae (strain ATCC 42720) OX=306902 GN=CLUG_00276 PE=3 SV=1

MAIKEGYTFTVLGAGVMGSAVTSAIIKANVKPFPGKIYLCTQDSASVARLRENYPQEYIH

VVADLEEKKKCVAESDVVLLGCKPFHYKGLYEEVSSSLSHKPLVISLLAGVTISQLTIFS

PYIARVMTNTPAQFGCGMATVAFSPEAQEKFKDEVLALANTVGSAVEIAEKNMDAATALV

GSGPAFCLLMMEALYDGGVRMGLPHDVAKLSAAKVMEGTAKMVLETGEHPAVLKSKVCTP

GGTTIGGLLKMEDAGVRGAISRAVEEAAVISASFSK

>tr|C4XWF4|C4XWF4_CLAL4 [histone H3]-trimethyl-L-lysine(4) demethylase OS=Clavispora lusitaniae (strain ATCC 42720) OX=306902 GN=CLUG_00277 PE=4 SV=1

MTFDKTLLKPCPVLLPSAEEFQNPIEYLSREDILQLGNEFGLVKVVPPKGWKPPFSIAPS

FTFHTRIQKLSDLGITTRSRKIFIDGLNRFCNMTGRRNVHSWFVTHNQKIHYYDLYLAVC

RLYPGRNDLMGISQEELTKLNLMFDLPASETILKSEFTDKIKAYAQYLSHNGNNFDFPES

DPEDDTESCLVCRKNHSPTQMLLCDHCNNPYHLKCLSPPLTEVPEGTWYCEKCLIGTGAY

GFEENPELKFNIWGFVEHCKQFESEFFSRYSKDGSPLSLDEIEQLFWNLVESENSELKVR

YGADIHNLRPGEISGFPTMEIPKSPYDSNADGSQYIHHPWNLTRLPFAKGSLLNFINSTI

SGMTIPWIYVGSLLSTFCWHVEDHYTLSANYCHFGNVKKWYGIPSSYADEFEKIMKASAP

DLFQRQPDLLHQLVTLMSPSELSAKGIPCVYADQGPNEFVVTYPRVYHAGFNSGLNFNEA

VNFTMDAWIDFGERSIRDYAEIKKENVFDHFMLVQNILENYLQPNSAFEDNHKNFIGKCI

RSYEQFLHRQKSLVHDLESDRLETVLKVPESKSGLMTPGPATRMSTMKKNKANTEDEDDD

LCDICRTYVSFQYCDVNNKLHRFGKWYHKKSRKTDSSSIAVSNLLTPTESPYLKKGQEDG

MTYSATAVAQSGSAERLQSLQDNNQDLKEEESEEEFGAKVPFSDQMNELISQAKRKASEE

AEEPRSKRRQSSRLQQKSRSPELEAKPEINEEIEDSASPQKKSQYSSLLRQLNQFDHIKL

CLKCTQKMCGDHGERLPKGSHLIVEKPFGEMEAILAGAKRKFLGY

>tr|C4XWF5|C4XWF5_CLAL4 ATP synthase subunit beta OS=Clavispora lusitaniae (strain ATCC 42720) OX=306902 GN=CLUG_00278 PE=3 SV=1

MDGTEGLVRGASVTDTGSPISVPVGRETLGRIINVVGEPIDERGPINSKKRNPIHTEPPS

FVEQSTSAEVLETGIKVVDLLAPYARGGKIGLFGGAGVGKTVFIQELINNIAKAHGGFSV

FTGVGERTREGNDLYREMQETGVINFEGDSKVALVFGQMNEPPGARARVALTGLTIAEYF

RDEEGQDVLLFVDNIFRFTQAGSEVSALLGRIPSAVGYQPTLATDMGALQERITTTKKGS

VTSVQAVYVPADDLTDPAPATTFAHLDATTVLSRGISELGIYPAVDPLDSKSRLLDASIV

GKEHYDVASNVQQTLQAYKSLQDIIAILGMDELSEADKLTVERARKIQRFLSQPFAVAEV

FTGIPGRLVRLEDTVRSFKEVLDGKYDHLPENAFYMVGGIEDVVAKAEKLAAEAK

>tr|C4XWF8|C4XWF8_CLAL4 histone acetyltransferase OS=Clavispora lusitaniae (strain ATCC 42720) OX=306902 GN=CLUG_00281 PE=3 SV=1

MPPTLNTRRRNLLNQIKITNNRIYDSIPEGKRRRAASTREPERVFRSSRSSGKPIEVKAL

PRRKTQSAPTVVPQNGTTLRRIGRPRLFSVTVLEKNPDRYILKIKYNRRNRMARRLLGTK

NQPKPEPEKASYAPSIQTSKRKRGRPPITKPPPVLEDIAPEEPDLDVLETSIDSELPYKG

VLPFPDCTINSTDPTPLDREMFAQFQLEGERRKREQEDAILQLRDDDTEQKESNNSTPMP

QSYYYQKSRIEKIQFREFVIDTWYSSPYPEEYSQSQILYICEHCLKYMKSPMSYDRHQLK

ICTIANNHPPGVEIYRDSEAKIAIWEVDGRKNIEYCQNLCLLAKLFLNSKTLYYDVEPFL

FYILTEIDDHDPSIYHFVGYFSKEKLNNSDYNVSCIVTLPIYQRKGYGSLLIDFSYMLSR

SEFKFGTPEKPLSDLGLLSYRAYWKVTIAYVLRDLHNKYLSQSNDNNIMLSIEILSKLTG

MKPSDVVVGLEQLNALVKSVETGGYAIVINLPVIDKVIAKQEKKGYIKLKQQNLQWKPLI

YGPSGGINSAPAYLASAGNTGAASQASQPIPISNSISMISEFLKDDINNPYTYEEEAYKE

IDSLTSGEVKDNLRRWRTKDGEWDTNSLVVCHPDFANGIPMVRSYTNASNDPKIMDEFQE

QVSEGESELGIDEIIDDEQPSYDESADLEEEASDEAIFEDTQNGGDEDNADEDDADEDES

DEDDAGEDDADEDDADEDDADDADEDDADEELSNSNQTGGGKGDFNNEKEKGLQTYDTYL

EIGPTKGQETSDSEEEVVQRRFRRKKQQPSLTRKSRRLRSSNVVSNGDVLGRRLRTRRNL

GN

>tr|C4XWG2|C4XWG2_CLAL4 proline--tRNA ligase OS=Clavispora lusitaniae (strain ATCC 42720) OX=306902 GN=CLUG_00285 PE=4 SV=1

MILLRRLHTKSVPRLYSVSSELLDSQVLPRIPTHELLARLQYVSPPKPGLVHWMPMGMLV

LNKMKQLVHKHMQDAGAEEISLSALSHSDLWKQTGRWGGAELFRLKDSKGAEYCLAPTCE

EEVTHLVQQNVASYKNLPLLFYQINTKFRDEKRPRSGLLRGREFVMKDAYSFDVSEAEAM

RAYETMSRAYAGVFAALRAPCVRADADTGEIGGSMSHEWHYVHAAGEDTLFECTDCGRTS

NVEMTPSFPAEDAAEPSKTSLEAAVAYFPTIDRSTLVCAYYPVSRAFRPAFLRETVPDID

LSGTLSEEEVLALFRDEATIIDKRVVRIMDSRLDSRSNLPDFPVKFANRSLVSTLTDVPL

VEAEEGELCAHCDSGTLRSSRAIEVAHTFYLGDRYTRALDCTVEVPGADGTVSRQPVLMG

CYGIGISRIIAALAEINRDSHGLRWPAAIAPWHVTVVDAGAQEQAHEVARSLGDMEVRLD

ARPSVRLGRKIRESHAMGIPLVLVVGKRYPTVEIEVRGRQHGRSWETVRQESSFDWIVET

GASHTKHIVDCANAPKVAAALLADM

>tr|C4XWG5|C4XWG5_CLAL4 J protein OS=Clavispora lusitaniae (strain ATCC 42720) OX=306902 GN=CLUG_00288 PE=4 SV=1

MKTCYYELLGVEATATDSELKKAYRKKALQLHPDKNPHDTEGANARFALVRSAYEVLSDP

QERSWYDAHKSQILRDEDDFSASDAPEEMVIPSISVQELLRYFNPSLFAQIDNSLSGMYN

VAGRLFERLAAEEVTHGKYQRLPKFDSYMDDSANVNALDENVLLYPRFGNSHTDYANVIR

NFYNTWSNFSTVKSFNWVDEYRYSAAPDRRTRRLMEKENKRARDAARKEYNETVRKFVGF

IKKRDPRVKKGADQIESNRKRMQRENLERQAKEQKIQRMAEISNHTVQDWQEMDLQELEE

LEAMLREEYNFESDETTDSEFDEFEDNINENYYECFVCNKNFKSKNQFETHENSKKHKEM

VELLKEEMRQEGIELGIDKDDIDLSEFETASSGESGNESTNMPNEEISSNVDESKESFDV

VEDEEKNWSSYEVDDVIESGESENEQIVESVKKNKKNKIKKKGPANIEKDLEDNGNILDD

ELEKIVNGISLEDSDDDWANDKKKKKKPKKRSEASKSPSVTELPLDDKAEKAMPAKVPNG

SERCEVCKEIFTSRNKLFHHVKKSGHAAPISDIKKKGKKKR

>tr|C4XWG6|C4XWG6_CLAL4 Protein SQS1 OS=Clavispora lusitaniae (strain ATCC 42720) OX=306902 GN=CLUG_00289 PE=3 SV=1

MAQLMEVNGADEYEDELDDENSMRLSEEDSDDESIDNQFLGSDEEGLEEILAFARQQKNI

SELEFPPTQTIKKKGRGKKQKPELQTDLDLEIRESLMEQFQYQKSSRRDKKLRKKEKRQQ

QALVDNDLSVKYDYSLHIKEIKQEFETFLHDVSRDTMSFPPLDPHGNKTINKLSSCYNMR

CTRCGGNGLQMYMKVAKTRKTFHYLPDYNQIAYIMRQRPVFKRSDVKSRTKEEIAETDGK

KSRRGPINEAYVKEGDLVGAKAPEIGTNNIGRKLLEKLGWVKGEGLGAHGNKGISEPLMA

TVKKSKTGLR

>tr|C4XWG8|C4XWG8_CLAL4 Cysteine protease OS=Clavispora lusitaniae (strain ATCC 42720) OX=306902 GN=CLUG_00291 PE=3 SV=1

MTSDNQEDGNAIEAQVQLLQNTIAQWWNRVQSTESEQLGSSDVTILGNRFPLEEIYPVIN

SRLWFTYRAGFEPIQKAEDGPSPLAFLKSMIFNVRPSMALGGLFDNQNYSTDVGWGCMIR

TSQSLLANALQMLILGRDHQSPQAIQSAPEKVEKIIQLFGDDYTCPFSLHNFIKVASASP

LKVKPGEWFGPSAASLSIKRLCAKFESNEIPNINVSICESCNLYDEEIRGIFEESESPLL

ILFPLRLGIDKINSIYYPSLLQLLALKQSVGIAGGKPSSSYYFFGFQGSNLLYLDPHNLQ

AASSDPGTYHTSKFQTLSISNLDPLNACWSVNQMTYDDYL

>tr|C4XWG9|C4XWG9_CLAL4 RNA polymerase II subunit A C-terminal domain phosphatase SSU72 OS=Clavispora lusitaniae (strain ATCC 42720) OX=306902 GN=CLUG_00292 PE=3 SV=1

MVADSLKVCTVCASNNNRSMESHKQLKDAGFDVSSFGTGSSVKLPGPSIDRPNIYEFGTP

YETIYQDLLSQEYRKMYEANGLISMLDRNRHVKKAPEKWHNNANAGKFDLVITCEERCFD

SVVEDLMNRMNNKSEDAEEKDVRKVVHIINIDIKDDNENAKIGGKGIVRLVKMIDEYREK

EKQRKIDEGDEDQYPVLLEDNIMKILTEWQREHTHLPTLYTASYY

>tr|C4XWH3|C4XWH3_CLAL4 Ubiquitin carboxyl-terminal hydrolase OS=Clavispora lusitaniae (strain ATCC 42720) OX=306902 GN=CLUG_00296 PE=3 SV=1

MSKPTVLSKPTQMSTHMSNGSKNPDALHSEQDVVKDKTRKAKDDKEKVIKEEDRKKVSVS

KDTQPKDSDSSASSNSKKTLSSSSDGSNDKDKDKENSTKDTAKKAGPRRFRGDVAPFNPS

GQTQLYAQQMQYPQAPQPYPQQQFYSQQPQFIYGNQMLPYYPMAMGYPDLMYQQYQFYAM

ANQFNSPQPYTGHVHANGYVHRKKHPKGQHNHQGHQPYGYQQNGSGSHIIPSDSASPPAS

QSEFVTPENHGPSPQHDPQHDSDPIQPNQPEVPKYSEEFPQPSTEDQETTQAAEIKAQAN

QSHPQLQSQHGSQHTPAATPGSATTDSYAENAKVPISGPLIFNISATELAEERKRSVQLR

RQLAEAKNARANEFIKQVDGDLAVNPYGYYQLKDHNSNDEYYKHNFPSTKTSNESNDNDS

NDPSISSPKANLNWASILQSSKKQPKSAVATKPASSNVTSEVSAGSSVTAKIVETEQYPQ

SLGLLAMKLLSDPNFNLDACEMFEVKPRGLTNSGNICYMNAVLQCLVMCEPFNKLLRHVQ

EKSIGTLGKTSPTPLLDATISFIEDFINIPSSSKPNVSSLNSEGIVVGKPLSPESLYMKL

IENPKFKHLKWGQQEDAEEFLGYLLDGLHEEFVKAEASVSPEMLEMLYNKYSQSLDPHLA

EQLKLKMKSGARLVRSTESKEVEDSEESEGDHSGWSEVGSGKKVNKKRVMEVEPSPITEI

FGGSFRSVLTVPKAKVSQSITIDPYRSIQVDISHKEIETIEDALLRITEVEKIPYKIEAG

KEVIARKQSFIEKLPQVLILQLKRFSFEHQQPQIATSENGEQDSKSISTHHIGTIEKVMK

NIKYGLDLTVPLECLSSGVRPTEKRDYRLIGVVYHHGRNAEGGHYTCDVLRNKREKKWLR

IDDTAVETITADMVVDVPDARDKSAYILMYQRV

>tr|C4XWH8|C4XWH8_CLAL4 Vacuolar morphogenesis protein OS=Clavispora lusitaniae (strain ATCC 42720) OX=306902 GN=CLUG_00301 PE=4 SV=1

MFTPNFTGLFYPHQISKYLSHSYSCDESSLDIEPACRFHNVISQTRRGKHNWRIMATISI

NDTSLENKVTYYHISVTLPLRSISVARRYSEFVSLVENLCSEIGISQRDFPYPLPPKGGL

FNNSTKIVAERKVKLTEFLSNIVRDRDLQNRDSVHKFLGLPVSFRFTPELFKENQQDESS

AKFLIDDDVADINKGQWLSYLRIVRFNASDMGKGTDLASKLAARENANKFIRPNLEKLSL

SLQHLSKSGAIDHGEYSKRNNMLKEIQADVEKVVRGEAPKVSNLPESSSRRIFAKRDDTP

PEETNTTLALNNKELLQQQKLMHQQQDQELEQLRMVIGRQRQIGEAINKEVEEQNEILDR

FSNEVEASSQKVQTARTRTRRIG

>tr|C4XWI0|C4XWI0_CLAL4 RING-type E3 ubiquitin transferase OS=Clavispora lusitaniae (strain ATCC 42720) OX=306902 GN=CLUG_00303 PE=4 SV=1

MSENIAAVSAPAGSCKKDSSPLSSPSHSAPGSPHQNRMPAPESDPESSSSSESAASSSEQ

VSVNAETTSANVRRPSTQDSHEDSRPQATQRPSFGLSFLSSFSNTIRGSVSSAAAAAESS

GHFMSNSPTIGNLSGFAEESPSTATRGSVASAPTSQGPVVRDSQEAEAREETTMCITETR

DEAPESTVAEPDTPESGKDKDGFYSIRLTPIIDHSSSNSGLYFSPVIRRIKPKDSISIGR

YTEKNKSAAHAPQGSSAPVVFKSKVVSRTHALFQCNEEGSWFIKDCKSSSGTFLNNIRLS

QASQESTLWPLIDGDIVQLGLDYRGGTEEVYRCVKMRCEFNRSWQRKVNRFNLDIHNRMK

SLGLNTENQKGEELSECAICLFKLEPCQALFISPCSHSWHYKCIRPIIIKELSTVLSSQL

PVHL

>tr|C4XWI4|C4XWI4_CLAL4 Broad-specificity phosphatase OS=Clavispora lusitaniae (strain ATCC 42720) OX=306902 GN=CLUG_00307 PE=4 SV=1

MTREIVANTDPNVVRIFVVRHGKTDYNAKKIMQGHLDIDMNDEGREQSEKAANHLKDIEF

DYIVSSDLIRCVNTARAIAQKQKKPFGNFPTTPDLRERNMGPVQGMQVQDALEKYGPDFK

NIGEKQEDLVNRVTQVWEQTFKKAVAENHTNTVLCTHGGVIRAFINHLYNVRGYKLAEGM

TFEDLRVPFNTSVTMIDLDKTTGEGLIQNFGSTEHLGGHFVVSNQQLR

>tr|C4XWI6|C4XWI6_CLAL4 P-type Na(+) transporter OS=Clavispora lusitaniae (strain ATCC 42720) OX=306902 GN=CLUG_00309 PE=3 SV=1

MPCTSGVSYLQDRYKGSWPHLGIYQMLRSIKRLALPVSTRKKLFYRLQQINLLEFGMPNS

DSNKEKIPSYERNDTEESSSMSSKYTGADEFQPYRHTVEKVARVFGCDTTNGLTSQAAKD

LLSEHGSNTLGEGDKISYTKILAHQVFNAMIMVLLISMVIALAIKDWISGGVIGAVVLIN

ISIGFIQEVKAEKTMGSLRSLSSPSARVTRDGDDFQIAAEEVVPGDIVHIKVGDTVPADL

RLIDVMNLETDEALLTGESLPVAKNHEEVYSDSSVPVPVGDRLNMAFSSSIVSKGRGTGI

VIATGLNTEIGRIAQSLRGEKGVIRSVNRDGDNSPGVKDYSRAFFGTIKDITGNFLGVTV

GTPLQRVLSWLAIFLFWVAVVFAIVVMAVQKMHVNKEVAIYAICVALSMIPSALVVVLTI

TMAVGAQVMVSKNVIVRKLDSLEALGGINDICSDKTGTLTQGKMVAKKVWIPSVGTFGME

NSNEAFNPEIGDLTYTKYSPKYVKETDEEIDFLPIDKTDKENFATNFKTWLTAATLANIA

TVTQSKDEESGEMVWKAHGDATEIAIQVFCCRVGFPREEFARGFKHIEEFPFDSSIKRMS

AIYTMGDKTKVFTKGAVERILRRCTRWTGNPEEKTDTSELVEMTEDDIAEIEANMDAMSS

QGLRVLAFASRDYDLEKEDISDREAVESNLTFHGLVGIYDPPRLETAKSVKLCHKAGINV

HMVTGDHPGTAKAIAQEVGIIPRNLYHYSQEVVDAMCMTAVDFDALTDEQIDALPVLPLV

IARCAPQTKVRMIDALHRRDKFCAMTGDGVNDSPSLKKADVGIAMGLNGSDVAKDAF

>tr|C4XWJ1|C4XWJ1_CLAL4 Arrestin C-terminal-like domain-containing protein OS=Clavispora lusitaniae (strain ATCC 42720) OX=306902 GN=CLUG_00314 PE=4 SV=1

MVKAKSSHKQAALFDIRLKNVDHDVIVLKGSEHHAADTYLAGKIVLSVTEPLTVKKITLR

LVSTIRLKYSDTNVPKNAMPKVIRFEKKLYEYQWDSNEITKYLNHMYENVHNGTTSNIHN

SSSNNGSSQSMNKSNSVKSHGSTTSLKNLGLSLRSKSSTSLHLHALTSSSTNLTHSASQS

NMSLKNSHTLVSGNYEFPFSAILPGNMPESVEGLPGASVTYKLESTLERGKFHTPMVTTR

HVRVVRTMTTDAVELSETVAVDNTWPKKVEYSLSVPTKAIAIGSGTPISLMLVPLLKGLR

LGEIRVSLVELYSFIGYIPPSHSAERVVTEKTIPQPAVDEEYPLDRWEVTTFLRVPPSLS

KCTQDCDISTHIKVRHKLKFVIGLINPDGHVSELRASLPVQLFISPFVSVRARAEDQLSD

VESVGSGPEDEEEEVLFAQDPSVSELDVPDGLRSNQSASSFTGLIAPPVYEKHIYDQLWS

DVSPMESPIASGSATPRSLRTDVSQFSMASIDTAKLTENLRQLSIQRQMQENEVSQASSA

SNLRGRATFNLDGGDDTDYFSKRPNLSTRSSAFNVAAGTPGIVSPPVHLSRVGSESSMAM

SRVPSYNEAMKSSVDGSLAPIYSPPPPGSHVNIDEANRRFEEISRPTTPVGSVGRNRSFL

SRGSSSFNLKGSAGSSPSTSRNPSTNNLSSYFSEARKR

>tr|C4XWJ2|C4XWJ2_CLAL4 t-SNARE coiled-coil homology domain-containing protein OS=Clavispora lusitaniae (strain ATCC 42720) OX=306902 GN=CLUG_00315 PE=3 SV=1

MFRDRTNLFLSYRRTISRPKKATGSRVVALAEEEEGLMGSRAPRKGKRYKDNASEEAIEM

KPIAPSIFDISSSLDERLGSIKQKTHELSSLYKKLLITRDTEKSKLESRIEDLNYSITKM

FEECYVSIKKFEFLQKNYERLRLDYGADELAIIENYKKTYALKIQDSSVVFRNLQNNYIK

FLKDDDDEETDKLLMSATPSSVALMEDTQNIEEYSKQALEQAQEQIQQNPNSQLIAQRER

EISKLAMGILEISTIFKEMETLVVDQGSMLDRIDYNLTRTVEDLKSSDKELIKAQGYQKR

TTKCKIIFLLCLIVLALFILVVARPHGSTTVEKTKGGNENDNTDTKTEDTTTGDTEIGVD

EALLGGPEEKAGPLLHVLL

>tr|C4XWJ3|C4XWJ3_CLAL4 PH-response regulator protein palI/RIM9 OS=Clavispora lusitaniae (strain ATCC 42720) OX=306902 GN=CLUG_00316 PE=4 SV=1

MQRSTLFTSVSLVLLVVAFAFSILGVVTSPISSSLKLGSTPDYTFGIFGYCHKNKCSSSS

YPVSFGDIDKNENWLLTASTRNTLSKGFIISPISCGLVFLALIFTSASLFVQHSLIKIFS

LVFGFISFVALTVTGVFVVLVFYPHVAWTGWILIAAAALALAALPCLFFSIGVKESVSQD

DESENSAFGGYGKIDNDTSFTSTTLMKQNSDSKVQFNGPVTTAYGENASSFSNEYSYRGM

TGGGGYDVTKNDSQTSLFNSKPNDAKDFTKQRPTAPSLNLNGSTTSFYGDSKMNMHEVPR

TPISAKQKMAPHLVPNSAVTSNSHLPDNTALPYPPSESTITRLDSAKYGVFDHHPEVEGH

KPFTELDDSELNDQESETLPQPQDSDDDSDFTSVSQRPPNVDFPGYYQQAPQHQPYAQHY

QQPYPQQAYQIRPNLSEPIQSPQQSSYYGSDGSSANRGYYQQPQGQRPPQPPQHYQQFAP

QGYGGRSAPFQPRPVPQSKPHGPTISDSVLNNNPDFALGAGGRRKFVPVAARNNASNRPA

RSGARDGPYGMIG

>tr|C4XWJ4|C4XWJ4_CLAL4 1-phosphatidylinositol-3-phosphate 5-kinase OS=Clavispora lusitaniae (strain ATCC 42720) OX=306902 GN=CLUG_00317 PE=4 SV=1

MPDKEPDFVSFPTLPDPETTGQRSFSNIISNTLRRVTNNASHLVHSYAPPRVPDTIAPNF

APDSVPPGAPPAKNTPARPPSPQSASLAQALPAASSTQSVQPAPKPAPPITAPPAPTRAP

LSQTRAPSIHTSTPSVHTTIPVPTKPLPARVTSDARSLRLSTMAPSAHPASVVSRDEVTV

VAEPLELSREGATSPRPRSSSPPPVSPGAAHSTLQNRISSIFNNLPNDIELSDDSASETE

SVGKSGNTSGISLGNDDAKEKSRDLIGTQANSTTNGSTISRSPSRRRRTGVRSSPSSSNF

YTLKSFAPPMTRKISSNFSSAILGGAKSILNTNLAGVASSTTSMVSSISEKKRRKKRKPR

KLSDNPLKNGGMPKKYWMNDAFVADCLNCFKPFTAFRRKHHCRFCGQIFCSDCTLFISYS

HYKNQRQNKDALIVTDSNNYRDKLRVCKACYSDVVVYLSDDSMSSCSESDIDNDDVLSLG

RSENSEDPPTQREYPLARYRTLSITSRKNSVGGDMSLTPTTNRAFLNNGKETEALASASP

AGYSEPAQRSPEGISYKQAPQMAIPTTRTGQAVEIPLTKSSTGAPSSLKSNLTFLALKNA

SNANMHSNPALGADMDMKNAPWYIPYAYPQAPVTGDSMRPNSLENLSSFYKSMVNYRYGR

IASNSDKYQNQSLLPKSSGDQAEEEDFESENEDEQVMSLYTSLNHSSNTQPSISPRPHNV

LSTSMSSVPTLHEFPNMVADDKYLPTTFNKSAGMMQFDFGVNGSTQGVSINEDRSRSDRR

SNARAKASLRRIRGRRTQKFNKNKWSGHPGHLTSSMENGLTSGNNINTSSFSPLSSTPRS

SSVTLNNRLRDASSFLDSSQRQSSHEPQGMNATAEHTQDSQYSLDEIKNGYDSHRSSNPE

AFVLESLDALETNLSESFNRHIDSMITQCLADCDINDPADQNRWSKVLTKCLSNMRRIKI

SDTLDVKQYVKVKKILGGKIEDTDVLDGLFITKNIDSKRMSSYIENPKIALLVFPIEYLK

QKEQFISLRIVDSQQSVFISNLVSRLISLEPDIIVVGDTVCGLAERLLEEANITVLSNVK

PQAVERISRYTNGDIFQSVNDLFLKKSSLGSCRLFEVKRFLYKDTIKTYSFFTGSNIESG

FTICLRGGDEEFLSGAKYATETLIPGFLNSRLEQSFFMDCSLYLQKEETVSQFDILQKYL

SDKATSPDDLKSAALDTMERLGVCNYIKLFETRYLSVSPSVEYPLPPVLANVVETFQDFS

NFYDFNSKIQQLTTEDTTDSAWFEKLHFAFNLESFSTKEDVVNILKFMSDEMTKRKFNEF

NFRSRLWANCMKYTIYQLYPIFHKSIHFLHSTVSIKHATPCHGPLVVVIDYYTDNDKSLG

SFLDQIFQESNKLCDECGEIMLDHFKTYAHDNGKIDLIVEKVENSGFEDTRHKNEHFMWS

YCPECDFSTPITALSDETYSLSLGKFFELCFWSRNVKYQNHCPHDFFKKHIKYFGRNGLV

IRMEYSTIDTYEVVVPKKKHEFVSETDIKLKLEAYSGIEKSASALFQSVAKRLNRVKVDT

FDKAEDGSKKVEELKERLRQQEDLIASKTFNIYNSTSATNYLSLNVIQRNIQELGVQWES

EFNDFEQKYLPTENEITRITQFHLRNLLMDKLHDDSKSKTMERENPSKLVADNSLLDDAK

TSETDENKREPGDGEDGEAIRKDNPPLGIKLRVPSSLIEDKIMQIKQSFENDSNQGFKPP

AKSRSSSIADSSIFDNSSVIQGVKKVQDLTNYFNQMTLEFQRQREETLERNLNKYKATPV

ANSKPIVEIYDNIEDVVDVKHDYDKQTAKLDNNGSNHSEGQEVQSSEVSPESSVHEQSAN

AKRMDNLNASDTKITKGDFRKDDNAKARLDMAQPEKNSLLKSLSNFWADRSATSWDPLEF

PLDSTEHTFADNEVIVREDEPSSLVAFCLYSDDYKQKLSDSASNVENQRDILEINDQYKK

KLHNFTKIEKKFKKNYDTNSKNSELESAMTKEKSSHLKFQFVDGNSDLSCKIFYSEQFEA

FRKACGIDDSFIQSLSRCVKWDSKGGKSGSNFLKTLDNRYILKELSKTELESFVSIAPFY

FKYMSQSTFNTLTTAIAKIFGFYQVEIRNSVNGKTFKMDFLIMENLFFNRTTTRIFDLKG

SMRNRHVKQTGKENEVLLDENMIEFIYERPVFVKEQSKKLLRGSLFNDTSFLSAMDVMDY

SLVIGIDEASHKLYVGIIDWLRTFTWDKKVENWVKGTTLVGKKGKDPTIVTPKQYRTRFR

EAMDRYILEVPDIWYEGNTQ

>tr|C4XWJ6|C4XWJ6_CLAL4 chitinase OS=Clavispora lusitaniae (strain ATCC 42720) OX=306902 GN=CLUG_00319 PE=3 SV=1

MSSRLSVNHAESRKRPRKLPQILLVVLLLGFSLVTLGVTVLLFAHVRTPTSTMGYIKGVY

YSNWSVYGPKHFPSHLDSDHLTHVFYAFLKVDAETGAALFTDPWADVEMPVDGKSGAVGA

LNHLREQNPKLSIVASVGGWGTAAMFQVLAGDRRKRLQFIFTALDLVTTHKFDGLDIDWE

YPSSPDEGQQLAELLRELRHAMDNVDRSLILTVASPSSQEHLANYPLGRMDRYLSFWNVM

CYDFSGQGWSEKTGHHANLYGDNGNNGLSASAAISYYTQKGIPPSKLVLGMPLYARTFYE

PQSLRIGVPFNKSAPYESDIVDYINITDENEVFDESRVGALKFDPSKKLLYSYDNPQCAR

EKARYVARNGLGGGFWWDSKGETQSQQRRLIRAFAQELDRE

>tr|C4XWJ7|C4XWJ7_CLAL4 Ubiquitin carboxyl-terminal hydrolase OS=Clavispora lusitaniae (strain ATCC 42720) OX=306902 GN=CLUG_00320 PE=3 SV=1

MSDFIVSACTGLKTNLSPSDKIYKDDCMFSFDTPENNANGLDICMSCYQAFARAPHKNYT

AEHYQDKRHSLYVNITKRLKPEEERKRILDNEDSTRQTKIPRLEVTDHEQDEFYNVFNSI

YVAPLDTSVPLDECPEPASSLAQQILFSNSASTNDEIKTWEQQVFPCAHSSSIEQTSATA

DFSHCASCDLGENLWVCLTCAAVGCGREQFGSSLKGNSHALVHFEQTGHAVAVKLGSLAA

DEDSCDCYCYSCNDEVKVPQLGAKLLQIGIDLNSAVKTEKSLIELNLETNKNWQFNLDGA

NGDKLEPVFGPGLTGLANLGNSCYLSSVVQALFSYESYDNFFRNLSFDKHVADPAIDLRS

QMLKLYDGLLSGRYSKPNPLDSQGYQAGIKPSSFKTLIGAEHAEFKTNKQQDAYEFLLYL

LDKLDREYGLSLNEEFKFLLTSKVLCTNCKSGTLSTELVDNVSVPLEEIEEGKTEEGKPV

YKEMSLPECFKRFAATEEIENYKCDTCGETSTALKTTGFRSHPKNLIVGVNRIKLENWIP

MKKDVAVDIPDSLQLSSLGAPSFESGETEVSETQNSAFVPNEEAMSMLSSMGFGEARSAR

ALFHTGNKNAEEAMNWLFEHMDDPGIDDPFDVSKEQDGNSAPENLEAIDNLVAMGFSALL

AKKALHLNGNDVNASVEWLFSNPDDDGVIHDNQPVFNIKEESEKLKKKLLEEPMGSGNYR

LKAVVCHKGSSPHTGHYVVFVRIKEEWVLFNDEKVVRCDNNLEDMRKNGYIYFFSQD

>tr|C4XWK0|C4XWK0_CLAL4 Ethionine resistance-conferring protein OS=Clavispora lusitaniae (strain ATCC 42720) OX=306902 GN=CLUG_00323 PE=3 SV=1

MVLSQAFNQLANPNTVNNRRRRLFIPPSTQFPLFSYGREPDQRSFLSLDESGSVYTGTDV

DDVVSVASSRSLPSTLSSAHTSEDQESFHSWLVEEHQRRYNATESDSDEQLLESRSVGSR

SSASHYSDKTFQSVFEQEDFASLKTTYSRELKMLVRYSTPLVITFVLEHFFSIVCLLVVG

HLGKDELAAVSLASMTSTITFAIFEGIATALDTLCPQAYGAGNYELVSTHVQRCFVFSLV

VYLPCALMWWNSNLLLQFVISSPKVLELTTQFLRILILGGPAYIFFENFKRFLQAQGIFE

AGTGVLFVSAPINIFLSWFLVWDSKYGIGYVGAPIATAINFWIMAILLVLYATFVDGSRC

WYGFCSPKELFSKWGQLSHLALPGIVMLESEYLAYELMTLFASYFGTTQLAAQSAVGSIA

SLTYMVPFALSIASSTRIANFIGGQNIYSAQIATRVGLMCGLCLAVANCLVLFTFRWQIA

RLFSKDDEVILLIVQLLAPLVSVLQIFDGVASVASGILRAQGSQKIGGIINFLSYYAFAM

PLAFVLCTYTDLELKGLWLGVGSGMVLIGLTETIVILNSDWESIIVHAGLMNYAEEE

>tr|C4XWK1|C4XWK1_CLAL4 Mannan endo-1,6-alpha-mannosidase OS=Clavispora lusitaniae (strain ATCC 42720) OX=306902 GN=CLUG_00324 PE=3 SV=1

MRFSTIQALSALSLCFLRVFAVTLDVESEDSICEAAWEVIVGELNYYEGTKYGGTVGMFS

PPYYWWNAGEVFGGWVDYWAFCAQDNDTFTELLSNAMYHQAGDDFNYMPSNQSMTEGNDD

QGVWAMAIIQAVERNFTNTGDHSWLYMTQAIFNTMKNRWDTTTCGGGLRWQIFTWNSGYN

YKNSISNGCLFHLAARLYRFTGEKVYLETAEKVWNWMWDVGFIRDSPEFIIYDGADDTEN

CTDLTIHKWSYTYGVYLAGCAYLYNATGNETWETGVAQILDASSYFFNNSIMTETTCAQS

NSCNNDQRSFRSLFARCLSLTAGLMPEHFDQIYNNWIVPSAKAAAASCSGGSDHITCGEN

WSVDGWDNKYGLGEQISAGETIMALITSKNTPLTPKTGGSDAGASVDAGNSTLSSVNRNK

ITITGKDRAGAGVLTAIVLGVILAGAVWMLF

>tr|C4XWK6|C4XWK6_CLAL4 4a-hydroxytetrahydrobiopterin dehydratase OS=Clavispora lusitaniae (strain ATCC 42720) OX=306902 GN=CLUG_00329 PE=3 SV=1

MKQTLTRLAARASALTREEITRELEIINKQLPNEHWSAQFHDSSSEIRATYKLKTFAKTW

ELLNMIALQAQRLRHHPTITNTYNKIDLVITTHDAGNHVTDLDVKLAQAVHTAYAKNFVE

QTPIKNESKNHNDSRASKIIEELTRKYGKK

>tr|C4XWK8|C4XWK8_CLAL4 RNA helicase OS=Clavispora lusitaniae (strain ATCC 42720) OX=306902 GN=CLUG_00331 PE=3 SV=1

MGKYRKRFNEKARSGMLAKQAALKKARNKQFTRHFEDGNDTSNVPQETAVEQDPNADILK

PMTEQEKEERKKRLAENLYSENEKESKMSRAKRKRLDKYIEHQIKREEKKVLLEKLAETK

VDTSMFAPSKLLGTGKQTRREEMIEALELERQGRGNERTKDILYEERDVKEWSEEDDVSD

HADNGNEEESDFDDDFTSTNSTFVDYRPSASGGFGSGFGFGNLPKKTKTSTTKKYTWRLK

VEREEKKRAKIEDENDFISSDEEDQDGSDSEGHETDKESGSEESNSGVSSEIESGSEKVA

SKESHSEEEQEDSDESTGDSEDESEDESEDASEEEEDEEEESESEQPRLLQNKPKHSKVA

ESFKEWAEEQVRKLEGRTEMVLQELKPETKEKYSKGVHNDKAFSDDEDSVPINPNLQRDA

FFVNVTRSPEIQQQRMQLPVFSEEHRIMEAIHHHDCIVLCGETGSGKTTQVPQFLYEAGF

GNVKSSMYPGMIGITQPRRVAAVSMANRVGAELGDHGARVGYQIRFDTTIKNEGTENGTA

MKFMTDGVLLREMMNDFLLTKYSALIIDEAHERNINTDILIGMLSRVVKLRREKDKPLKL

IIMSATLRVSDFSENKQLFKLAPPILKVEARQYPVSIHFDKKTKFDYVEQAYKKACKIHK

RLPPGGILIFLTGQNEITTLVKRLRNEFPFNTRKKQYVEDENTEYRVSNNVAMEAEDVDL

DIALRERKEEEMEEGSDSESDSDEEEGFEETLEEGQSENDPLYVLPLYSLLPTKQQMKVF

ENPPEGSRMCIIATNVAETSLTIPGIRYVIDCGRSKERKLNEETGVQSFEVDWISKASAG

QRAGRAGRTGPGHCYRLFSSALYEEFFPQFSKPEILRMPVESTVLTMKSMGIHQIVNFPF

PTPPERRALARAERLLITLGALDKKTKTVTELGKTMAFFPLSPRYAKILIVGNQLQCLPY

VIAIVSALSVGDPFLDEYELGLKQEAKQKSAGYSSDEEEEEEEDEAEVQADVERKRRMRT

KFFKSRAIFARLDEKSDSLRLLSAVCAFDHVPEAKKSSFLNDHFLRAKVMEEIQKLRKQI

THIVHANAKKDTIAHTEQKGAKLGIPSKQQVSALKQIIASGFIDQVAVRTDTIDADFKLS

KKSSIISVPYSVTVPLSQFGDEVDPHVYIHPNSILASSGSTPPEYLVFQSLNKGSNQKEN

QTTKLRLKPLVDISGKQLANVAKASGLVTYSKPLGHPYGPKEITPTKRECYVVPRYGSGV

GDGKLGWDLPVIKVVQTKKFGSWVTEE

>tr|C4XWL0|C4XWL0_CLAL4 DNA polymerase epsilon subunit D OS=Clavispora lusitaniae (strain ATCC 42720) OX=306902 GN=CLUG_00333 PE=4 SV=1

MPPKGWRKNQQGKFPQNPKDTELVSIDEILFPKSTIAKLAKQITSGENDANMILSKDSLV

ALQRAATVFVSHLSFHARNLAKDADRKNFNAQDVLHALEKAEFMGFVPEVKQKLATFERN

SEAKKLKKADTKAQVPISSDEGPAAKKLKDNTLKSVDSTVDPGDETAQDEEEDDDADTRT

LEDEDEDDEIDAPEEDSEHRDTKTILNKDADLTVDDTEGADGSDTEGQIA

>tr|C4XWL2|C4XWL2_CLAL4 DNA repair protein RAD5 (Fragment) OS=Clavispora lusitaniae (strain ATCC 42720) OX=306902 GN=CLUG_00335 PE=3 SV=1

MHIYLEQRDVLTPPSKLFASLKFMEGKKRFFPSPSRSPNPAETVPSKEALFVEDEDEILE

VEPHQASQATRNGSAQQLSVDVPQTVPRREPLPNNLEAPTPAEPSLSDIGFELFQSQLSS

VIGDASMKAMRHLYAKYFNRPNYIQLASNEYFNGISFDDESSGEASENVQEDKREELKQV

MERMNTEARNEAEMKKAMGWKRYIGTLDVQAWATRPYLRSLRYQQELQLKRLIPNKMKKS

SIVASKFGDSAVIRLYTSEADGSREIGRLPEDITRILSPLIDLEIANFSASVIMETKGRL

SIGDPFFLQIKCYLNNNTFVDHGQVVGEEENSQSKRRKTAKLGITFSQETEAEAALRLKQ

KSISRLFDKLSIIPYDKSSVNTDAEEIPDDDSSVSSTPKPEIAPTEELSLEQLKQFYTAN

QQSDFLENLPETTTPPEENFALTLRPYQKHGLSWMLSREKELDLLEELSVNNSEVLSTQK

RQAIRNQDDEVMDPLWSTFKWPQDPANPDLSTSESEFFYANLYNGELSTEKPVLKSFLKG

GILADEMGLGKTISTLSLIHSVPYDSATARTGTRNYASKTTLIIVPMSLLSQWKKEFDRS

NKNSNHRCHIYYGDSVQTDLSLLLCNRSENIPIVLLTTYGTVQSEWTRINKLRDANGKLP

KIGLFSVEFFRIVVDEGHNIRNRTTKTAKSVHELESRRKWILTGTPVVNRLDDIFSLVKF

LRLEPWSNFSYWKTFVTLPFEQKKFQQTLDVVKSILQPIFLRRTKNMKQKDGTPLIDLPS

KEVVVEELEFSDREQLFYDFFKSRAYNSFKEGLKSGELLKKYTQILTHILRLRQVCCHPD

LVAASSELDDSWKEELAAFEEPIKKEKFWSETMMKEKLYSLYAKVHIEDSECSICTQAPI

SIGELTLTECGHQYCFHCILEHIEFQTNNGSEPLCPDCRHPISKYRLFKVLSKSTSKKKI

RSTR

>tr|C4XWL8|C4XWL8_CLAL4 Phosphate transporter OS=Clavispora lusitaniae (strain ATCC 42720) OX=306902 GN=CLUG_00341 PE=3 SV=1

MSFYHQFDYIFAIAIIFAFLDAFNIGANDVSNSFSSSVSSRSLKYWQAMILAAMMEFLGA

LLVGNRVSDTIRKKIIETDTFENEPTVLMLAMAMALVGSSIWLSIATLIGMPVSTTHSIV

GGVIGVGIATKGADNVIWGWKGFAQIVASWFIAPGISGCIASIVFLIAKFTVLEIKDTQK

AVRNALVLVPILVYVTFSVLTMLIVWKGSPKLKLDDLSTGTTVGAIFGVGAVACFLYMLF

IFPVVKRKVYYQDWRVKWYDIFKGPTFYFKSTDDIPPIPEGVQLTIDYYAGRRPNENANS

KKIDEENIEALEDSKQSVTSYTSNSVVSTEKPEKTSTLWFRLLKQGPKQWPHLMWLVVSH

GFTKDVISDQVNHSGVLGANVKDMHKKSKFYDNRLEYLFSLLQVITACTMSFSHGANDIS

NASGPLSTVYLVWSDKSLKATPPLWILGFTGGALVIGVWTFGYKIMSVLGNKMILQSPSR

GFAIEFGAAITVVMATQLAIPVSTTQCAVGATVFVGLCNQDLRSVNWRMVTWCYMGWIFT

LPIAGLISGILTGIIINAPRLGVEYFPSN

>tr|C4XWL9|C4XWL9_CLAL4 F-actin-capping protein subunit beta OS=Clavispora lusitaniae (strain ATCC 42720) OX=306902 GN=CLUG_00342 PE=3 SV=1

MSAEDKFDASLELLRRLDPKAVSDNVGAICTLLRSENEELASDLLSSVDTPLTVAKCGDT

NKSFLCCDYNRDGDSYRSPLSNKYYPPTGDDESPYPSASLRQLEVKANESFDIYRDLYYE

GGGLSSVYMWDTAEDESADSLEEGFAGVVLFKKETDDHSGKWDSIHVFEIVPESASMASY

KLTSSVILDLQNKSAGSKSLSLSGTLTRQIETTQQLSIEGGNLELAHLVNLGQLVEKAEY

NIRNLLQEVYFDKLKNIMLRDLRSVGDISEKEADDSKHSEMIKGLQGL

>tr|C4XWM2|C4XWM2_CLAL4 Clathrin light chain OS=Clavispora lusitaniae (strain ATCC 42720) OX=306902 GN=CLUG_00345 PE=3 SV=1

MADKYPELEVSADEQDVQGDFLSREKELLGDEFQTEQDNEVLAESDDDIQEFKEQFPEVD

NSIAAEPQEESEDDEFENFGSAPPVSQELGESEPLKEWRERRTLEIEEREKANRKKKEEI

VAAARQSIDDFYDNYNNKKDQHAKDVLKEEEEFLEKRDGFLKRGTLWDRVNELVSEVGEV

SDDGRDKSRFDALLKKLKGKENVPGAGGY

>tr|C4XWM3|C4XWM3_CLAL4 Elongator complex protein 2 OS=Clavispora lusitaniae (strain ATCC 42720) OX=306902 GN=CLUG_00346 PE=3 SV=1

MSLSEHVSQDAIFIGANRQPQVSDCVPNLVAFGAKNNVALWNPVASDRKGVYHTLKHGDG

DVTCVKFVPNSDFLLSAGEDGRLNLFAKVNDSPTLFCVQDSVVLEKASIVCMAVVDNVTF

VTGNTLGDVVIWQISGAQLEAKHTFRVKFNFYPTSLALEKIGQRYLLVVGGTSCDLHVYS

FDPEVSGLESCAVLTGHEDWTKCLAFVTEKEGSSYILASGSQDRYIRLWRLRLNEAIDDS

DEDDSKLILLSNKQYKFPFGDGRAAFSFDALIMGHDDWVTGLSWHPSYSRFSNGNEKKLQ

LLSSSADTALMIWEMDEESGIWVCGSRLGELSIKGASTATGASGGFWSCLWFEDAEDGSH

HILANGKTGALRQYQSKDSEHKSWESILGVTGAISEITDVVWSVDGSFFYVTSLDQTTRL

YGPWALNRPETSHKSLTWHEFSRAQIHGYDMICLDNLSATKFVSGGDEKILRVFEMTQSI

NNVLHKFCGVNSSLEAGETLPEAASLPVLGLSNKAANEQLEAGEAAQREEDYARDKKETE

VKDDVLAVLSGPPLEDHLQRHTLFPEIEKLYGHGYEITCCATSPDGKLIASACRSNSAKH

AVVRIFNAQKDYQLVDEVLKGHNLTITSLEFSPDGRYLVVVSRDRQMSLWKVKNESEGTF

DLVDINPKAHTRIIWDCSWAPQENGMCYFVTCSRDKSIKLWEVKDSAVACVATTKTEDAV

TSVACFQQGLINKNYVIAVGHENGQVSLFAVQTDAEEKAFRQIIAFDDQILPAGRVSKVA

FSKKCIDGKMQLAVGSHDTSVRLFSINRDIV

>tr|C4XWM7|C4XWM7_CLAL4 T-complex protein 1 subunit delta OS=Clavispora lusitaniae (strain ATCC 42720) OX=306902 GN=CLUG_00350 PE=3 SV=1

MAQTQTQAPSNATFRDKEKPQEVRKSNILAARAVSDAIRTSLGPKGMDKMIKTKDGKIII

SNDGATILKHMAILHPAARMLVNVSAAQDIEAGDGTTSVAILAGSLLGAAEKLLNKGIHP

TLIGESFQRAAARVCQVYLDMSHKITLDDRDILIKAANTSLSSKIVSQHSSLLSPLSVDS

VLKVMNEAQDNVDLNDIRLIKKVGGTIDDTELVDGVVLTQNVLKSAGGPTRTEKAKIGLV

QFQLSPPKPDMENNVVVNDYRQMDKILKEERAYLLNICKKIKKAKCNVLLIQKSILRDAV

NDLALHFLAKLNIMVIKDIERDEVEFLTKAIGCKPIADVENFTEDRLGSADLVEEFESSG

SKIVKITGVHSKFTKPTVSVVVRGANNLVLDETERSIHDALCVIRCLVKEKAMIAGGGAP

EVEASRVLIKEATQLSGVEHFVYQEFAQALEVIPTTLAENAGLNPINVVTDLRNRHEMGE

KNAGISVRRSGATNTFTENVLQPVLVSTSAITLAAETVKSILRIDDITFSR

>tr|C4XWM9|C4XWM9_CLAL4 Amino acid permease/ SLC12A domain-containing protein OS=Clavispora lusitaniae (strain ATCC 42720) OX=306902 GN=CLUG_00352 PE=3 SV=1

MPEKELNYIATNEVSSDKGGIIHVNVFEEQDNKPPMGRWENFVDGFRRVDEAELGLDPNL

SEAEKIAIITANSPLSRSLKPRHLQMIAIGGAIGTGLFLGSGKVLRTGGPASVVIAYILI

GCMIFSTVHALGELAVTFPVSGAFVTYTTRFIDPSWGFAMAWNYALGWIVVFPLELVAAS

IAVSYWDSTTNPAAFVAAFYFFIVFVNFFGVKGYGEAEFIFSAIKVTAVVGFIILGIVLV

CGGGPKGGYIGGKYWHHPGSFHNGFKGLCSVFVTAAFAFGGTELVGLAAAETANPRKSLP

SATKQVFWRISLFYVVSLILVGCLVPYNDPTLANSDGTARYSPFVIAIKNAGISGLPSVM

NVVIMVAVLSVGNSSVFASSRTLTALAAAGQAPKFFGYIDKRGRPLYGIITQLLFGLICF

ISASHKQGIAFDWMLALSGLSSLFTWGSICLCHIRFRYALKFQGRNTDELAFTSQVGVIG

SYFGLIFNILVLIAQFWIAVWPVGGKPNANDFFQSYLSAPVVLAFYLGHKIWKKNWKLYI

KVEDLDIDTGRRDLDLDLLKQELAEERAHIASLPFYKRAYHVMC

>tr|C4XWN4|C4XWN4_CLAL4 Zn(2)-C6 fungal-type domain-containing protein OS=Clavispora lusitaniae (strain ATCC 42720) OX=306902 GN=CLUG_00357 PE=4 SV=1

MEQRAKNEKETPPLFALDPLGRAFPPLASPLPPHMATWTPPNLAEAPQAGAPVAPEPQRN

GRLRVNRACKRCRTHKIKCSGVLPCTNCTRHGTVCAFGREEPPAKVRRTTSPEQERDDNA

AYTRYLEQRVRYLENLQAQMLQAQAGGVGEMGHRNDSSRPSENVEDDLERPDVMVVPTDV

AGLSRRSSTKWRSNHRSQLALVNVLCRDMYDELSPQAQTQVSVPRVQYFAWNMSGCHYLS

PPRFPPPPALAMSAERKAHYVDYFFREINPLYGVVHETVFREQLAASDGDTKSNFAVLFN

AMLALVYALSIRFTEFSAATGPDMAMLRLEEDLFQYAHSVVQGISFEWESFELIQCWLLV

TLYLRVTHRQTSTYNALGRAVHMCRAMDLGRPVEAGMAPYERLKARRIFECVFCIDRLVG

FLGGRRRTYPFMDISRAPPKRDYASARDDWHTLPAFAMGQIARALDMVRPEADDFACART

RAIDAELESLGAWLEENGFGDDIGKTDEAEYVSPAVRAQVRLHFYDVVLAVHSRSMFALS

GLPMGYGMRLEVVRAAGAGIIRVVQQLEARALLYMPWYTTLSLVFSAAVNCLVFIRANVA

VREARQVLRGAMALVERLQDSPVRDATGKLVFRERFKMVRECSWVLKMANHIMSLLFAES

ASGLHELGIDHGPGDVNKQYFAQFGLDGAPAQDALEKLAHDQNQRAHTPGYGADIVESLR

WFDHWLDFDGA

>tr|C4XWP2|C4XWP2_CLAL4 Sm domain-containing protein OS=Clavispora lusitaniae (strain ATCC 42720) OX=306902 GN=CLUG_00365 PE=3 SV=1

MVWVPRRAIWCTHVSCLHRLTKHRRNIRFPTMSAPVSVTKKTKMADLINFRLQISTLDGR

SFTGQLLAFDKHMNLVLADTEEARTTKKSYQELAKAKVGSQVKVNEDKRFLGLIILRGEQ

VVSVVIKSGPTADIKKRMSQLKQSRPVKTPVSQKEKLKGPAKAR

>tr|C4XWP4|C4XWP4_CLAL4 FACT complex subunit OS=Clavispora lusitaniae (strain ATCC 42720) OX=306902 GN=CLUG_00367 PE=3 SV=1

MSEVVIDASAFHKRLSIIQKNLVADKYDFSSLLFLAGARDDEATYKKSTVLQLWLLGYEF

AHTAIFINQDKCVIITSEGKAKHLAHLTSPPTANSSKVEIWTRTKDVDHNKKLFEKLVDE

LKAPTPVEGKSKIGTIVKDSFKGKFIDEWKEISGPANLEFEDAALFLSKSVELKDSEEFG

CIQMAAKASVVMMESFTNDMMVLVDEEVKTTNVDISEKLDKKIDSNKWFTKTGLGKKLLS

SNKDFDPEQIDWCYSPIVQSGGEYDLKPSALSSEKSFVGDGAIIASLGLRYKNYCSNVGR

TFLIDPNPEMESNYDILLELQEHIAKNLLKSGAVAKDVYTGAIEFLKSKKPELVDNFTKN

AGWLTGLEFRDSTLVLNGKNERELINGQVFSLTIGFNNLTNASATNPKLKNYALLITDTY

KVSEGEAYLLTNFQKNRREVTFYFKEDGNAVKKENGDRKLKTEKDITTEKKLAVNEANSK

ILKSKLRHENAAADDVNAEKVRQEIQRKLHEKRQQEGLARFSQADATDASDFRPVFKRYE

SYVRESQIPSNVRDLKIHIDYKSHTIILPICGRPVPFHINSFKNGSQNEEGDYTYLRLNF

NSPGAGGNISKRTELPYEDNPEDQFVRSITFRSKDRQRMVDVFKAIQDLKKDSVKREAEK

KQLADVVTQGNLIELKGSRVKKLEQVFVRPTPDTKKIGGVLQIHENGLRYQSSFRADQRI

DILFSNIKHLFFQPCKEELIVIIHCHLKSPIMIGKRKTLDVQFYREASDMAFDETGGRKR

RYRYGDEDELQQEQEERKRKAMLDKEFKTFAEQISDASKGLIDLDIPFRELGFQGVPFRS

AVFCMPTRDCLISLIDPPYLVVTLEEIEIAHLERVQFGLKNFDLVFVFKDFHKPVVHINT

IPMELLEDIKNWLTNVDIPFSEGQMNLNWGALMKTILSDPYSFFADGGWRGLTGEGDSDE

EESEEEESEFEASDDDPSDEEVASEEESEEDDYSSDGSEGSGSDEESEGDDWDELERKAA

REDSRRGRE

>tr|C4XWP5|C4XWP5_CLAL4 DNA primase large subunit OS=Clavispora lusitaniae (strain ATCC 42720) OX=306902 GN=CLUG_00368 PE=3 SV=1

MSRYSTTLIFMFRQTKRRVAGRRHFENVAAYAESAVYPSRLSFYDTPPLQEITLEQFETW

AIDRLRVLIEIESCLARGKSVRDMEGIVRPLLLKHLPSSDVDERRKDHYSHYTLRLVFCR

TDELRRRFVRAETALFRLRYNMLQPKEQHEFVRLNADKLHWEYISAEEKSALAEALYSAS

GAAVRALLADSGEPPLSAEHLRAHMRAESYIRLPFEKVGALVASRSVYVSRGRAYVPAAL

QSSLLAAEFSSSLSNALMKTFQALPRLEEDDRVLPLLSNLSRSFASVQYDATETEGLSAA

QVTSPDVTQHYPLCASHLQSTLVAQSHLKYIGRQQLGLFLKGAGLGVDEALRFWAGQFTK

SGTINQDTFNKEYRYNIRHMYGLEGGRINYKPWDCNTILSKPKPSRGEAHGCPYRDFSPD

ALAERVASMGVAQHDVQGVLDHVARHEYTVACTRVFELTHKVDENLHITHPNLYFDRSRQ

LSKKEART

>tr|C4XWP9|C4XWP9_CLAL4 Carbonic anhydrase OS=Clavispora lusitaniae (strain ATCC 42720) OX=306902 GN=CLUG_00372 PE=3 SV=1

MFRVRHILAPRYRQLSYSLSKMGRENIIHYQLEHDHETDLKGSSPPSKPALEAPRAALDS

PYTLSKSSTMADFLANNRFFVDSIKHNHSNQVFELNGAGQSPHTLWIGCSDSRAGEQCLA

TLPGEIFTHRNIANIVNSNDISSQGVIQFAVDVLKVRKIIVCGHTDCGGVWASLSNKRMG

GVLDLWLNPIRHIRAANNEALSALKDDPRARAKRLAELNVVASVLALKRHPSASMALKKG

EIEVWGMMYDVATGLLSEVEVPTDEFEDLFHVHDGDEEYNPH

>tr|C4XWQ0|C4XWQ0_CLAL4 Helicase OS=Clavispora lusitaniae (strain ATCC 42720) OX=306902 GN=CLUG_00373 PE=4 SV=1

MADEVIDLEIKKYLQDNEITEEQKSFNELDGEAKLARLDNLIQRSQLYSQIILDNIMEKS

LEKKRMAKKEAKKGSETKAANDSESDEGVYVRRRRQKVASSHSSSSSDSDSHSDSDSASS

VPSNSTAKSPIISESPTDGKIERNEHKKKTEDESPVTKSSHPTKPILVDSDESSGDDEFF

SMASGSEIEGKSQGANKTTRDKDRVTTAQSPRNTKQDTRSGNTKKEDFNQANNSKTNNIS

SGRKASSNIEVTEVLSGDSSDDLVITGEKSRVSARDTRHSKFAARKRRPEDSSSDDDHKR

SRISNDTRMKHKNVSKKSAKTRRALVKAQANHDSMQPALLTGCTMKDYQLEGLEWLITLY

ENGLNGILADEMGLGKTLQSIAILCHLFEHGVKGPFLIVAPLSTVSNWCREFENFAPKLK

VMQYTGDKESRKSYIFGASSFKKHRWNVVVTSYQLVVRDFRKMSRINWKYLVVDEGHRLK

NFDCLLVQFLRRLKVENRLLLTGTPLQNNLKELWSLLNFILPDIFQDLELFESWFDFESM

GEAAAIDVSSEDKAKISNEVQDRLVKSLHTILKPFMLRRMKKEVMKDLPPKKEYIVYTEL

TPLQKIFYKSIIHGDLKHTVLTFHLKEYLLCNHPDLFKTEEDLNFVDGFLDFNQGRLSNK

RERVAKYDRKTLMMLEQDSMKEFVVSDYSDDGSSSSENDADVHNVAESDTTIDLAANAEA

VDKEFADLLADFQTNQSQQNAISNQESKMDIVDVQNQPGSKISILLNSEDSESERRPQSI

IDGRKNSAVKSDDSENQMNHIKKDSSEFNNKTKQEPQPNAASEEPEVIEILSGDSDSETE

LRAVKDTPRHELQSNVILKLLPRFTRNLSRMRLLNRVMQLRNVCGSHYVYYEPLADDNED

DAYLAHLIFRNSGKVQLLKQLLDPLLKGGHKVLIFSQFTKILELIGVCLDEDKIKYSLLT

GNIFQDEREDEIALFNSEDEDSTKIFLLSTRAGGLGINLTTADTVILFDSDWNPQMDIQA

MGRAHRIGQTKPVKVFRFVVRDSVEEILLFKSFSKRALEQKVIKSNNFGKSTVAGQLIDK

NIDLTKITRLSNIWNIEQRLDHAKSSINYSTSRVLIESQEPLEGVTEEEMNELMDRSSEC

YARETSEFANITTFEVSSAEAE

>tr|C4XWQ1|C4XWQ1_CLAL4 glucan 1,4-alpha-glucosidase OS=Clavispora lusitaniae (strain ATCC 42720) OX=306902 GN=CLUG_00374 PE=3 SV=1

MIFFYSYPMQLSLFLAWAVAASAFYIPFRLPQIPFLVSLDTKTQDEYMTDPQFELWVAQQ

SNYSFQYILENIGGVSSILDKSEVAPGVVIASPSKEHPNYFYNWIRDSALTLRSVIYYLD

DGAEHLRHAEELRAVVEQYMEINYELQRLSNPSGDFDDNTRSGLGEPKFMPDASVFKENW

GRPQSDGPGLRVSTISNYIAYLKKYKLDFSNNFLGNTSNVYYGIVRPDLEYIMANWKKDS

FDLWEEINSVHLFNALTQLRALKDGHKLAKHVGDSSEFLNLLETSFNELKNYILDSNTGF

TQFSLPYLVETPSLVQQRKRAGLDAATLLASLHAHNLEYGNTDVIPFDVEDGHVLSTISA

MVADMKYRYPINHDKIRLPQSIGVALGRYPEDIYDGYGTSEGNPWFISTATAAEVLYKWI

FKALKSNEDIVLTSEHEHFFAPFFPDDKFTTSKTIVYGSEEFGTLLVQMFNYSDSFLQVI

QAHVDSVSGKMSEQFNKYHGFMQGAESLTWSYSAFYNSVRWRLKTSTELDKYFEQANRTH

>tr|C4XWQ4|C4XWQ4_CLAL4 Fatty acid hydroxylase domain-containing protein OS=Clavispora lusitaniae (strain ATCC 42720) OX=306902 GN=CLUG_00377 PE=4 SV=1

MVSAVYHDYSGFSNATTFSESYQNFSNIGSLNPVEKLWAAYYYWMNNDVLATGLFFFLMH

EIMYFGRCLPWAIIDRIPYFRKYKIQESKLPSNTEQWECLKSVLTSHFLVEAFPIWFFHP

LSEKIGMSFSVPFPSWSKILFQLGLFFVLEDAWHYWFHRGLHHGVFYKYIHKQHHRYAAP

FGLTAEYAHPVEVALLGMGTVGIPLVWCYFTRDLHLFTISLWICLRLFQAVDAHSGYEFP

WSLHHFLPFWAGADHHDEHHHFFIGSYASSFRWWDFVLDTEAGPKGKQSRESRMKAQAEK

KVQ

>tr|C4XWQ5|C4XWQ5_CLAL4 Major facilitator superfamily (MFS) profile domain-containing protein OS=Clavispora lusitaniae (strain ATCC 42720) OX=306902 GN=CLUG_00378 PE=4 SV=1

MPRKSTSPSSSAHSLSSETSGQNSRANSPARLAQDEASAGLARLDSSISADPSIPSSLSH

TVSRARSRASSYSVREIYGGLPDQEIRLRRTATRTTIIDSLQQRVDTRQDDFYEHLPDAP

VPVANGGEEFSSFDPELVTWDSPDDPANPRNWSNAKRIRQTVLVSLYSLISPMSSSILSP

AMTEIARSLNIHKRVIESLCVSIMVLAFALGPLVIAPLSESDRVGRRPVLNLSIWLVALF

NLVSGFAKTPAQLCVFRFLGGLGGCAPLNVGAGALADMWSDNERQFAMAAYSLGPTLGPI

LAPILASFVVTGLNWHWCFFVLTFFNVAVAAVGLLLLQETYPPRLLKLKAERLRRETGNE

HLHTIYEIADGETTSERVLHTISRPISLLVGHPMVFGLGSFMAFTYGFMYLFIVTFPSVF

KGTYGFSTNIAGLMYIPFGLGFVFGTVFWSVVIELIYKRKTASNGGVAKPEFRLPCLCFS

GIGIPVGLIWYGWSAQKKLHWMMPCIGSAIFSFSLVAVFQTIQNYLIDMNNRFAASSVAA

AAVFRSFFGFAFPLFATPMYDKLNYGWGNTMCAFIALALGLPFPVFCLMYGERLRNWANR

RFDEKQAKRDQRNLERLREQNEKEYLEE

>tr|C4XWQ9|C4XWQ9_CLAL4 GTP-binding protein OS=Clavispora lusitaniae (strain ATCC 42720) OX=306902 GN=CLUG_00382 PE=3 SV=1

METAPKETTNATILLMGLRRGGKSSICKVVFHNMQPLDTLYLESTTKPISEQFSSLIDLS

VMELPGQLNYFEPTYDSERLFASVGALVYVIDSQDEYLNALTNLSMIIEYAYKVNPKIHI

EVLIHKIDGLSEDYRIDAQRDIMQRTGDELLDLGLEGVQVSFYLTSIFDHSIYEAFSRIV

QKLVPELPSLENMLDNLVAHSCIDKVFLFDVNSKIYVATDSSPVDIQTYEVCAEFIDITI

DLDDLYVDGDGREAKSSEIRSTSYLANGSVLYLKQMIRGLALVALIRSDTENEKMDELLT

VIDHNVGLFKKSLMKMWASAKFQTQD

>tr|C4XWR1|C4XWR1_CLAL4 Dol-P-Glc:Glc(2)Man(9)GlcNAc(2)-PP-Dol alpha-1,2-glucosyltransferase OS=Clavispora lusitaniae (strain ATCC 42720) OX=306902 GN=CLUG_00384 PE=3 SV=1

MPDLPSLVVPFFTSMTAYIAACMRTYVPLPFIDEYFHLRQAQAYCASDYAVWDPKITTPP

GLYVLGAFWARVLAAWGVSAPCGTTMLRALNLLGGTLVLPLALQGALERNYWKTNIAALP

LLYTYFFLFYTDVWSTIFVVWAILAVTTFPTAKGAIICNVAAFASMWFRQTNVIWLAFAA

VVFVDRRRKRQASFVAELKSFFSQCLRDWPLLLPFAINIGLFAAFVVYNEGITFGDKDNH

KVTVHGTQIFYCNAFLAVMLAPLWFSWSTAKSYIRFAVTGRNGVNTVFTAVAFAVIYFII

VNLSVVHPFLLADNRHYTFYIFRKIIRRPYANFLLVPAYHFSTWVVFHLFLKSQSASSLT

LGPMGILAWAGACVLTLVPSPLFEPRYYILPLVTLRLFTKPDPGTRTHMLEFVWYSLINA

LVFIVFFSYEFSWPTELAPQRIIW

>tr|C4XWR5|C4XWR5_CLAL4 peptidyl-tRNA hydrolase OS=Clavispora lusitaniae (strain ATCC 42720) OX=306902 GN=CLUG_00388 PE=3 SV=1

MPLLSAISKYQKMGHSCSAQLATFGVLVFAAGYSLHGFLNRTSITRKRFSRRSETDTEST

ESGTESEYESEDEIDIDSTALNDIPGEVRMTLVVRQDLKMGKGKAAAQCSHATLALYKKM

SDSSSAAYNPELLRRWERGNGQAKVTLQVPNQEEMDLLFAKAMSLGVNAYIVHDAGRTQI

AAGSATVLGLGPAPKLVMDQITGELKLY

>tr|C4XWR7|C4XWR7_CLAL4 anthranilate synthase OS=Clavispora lusitaniae (strain ATCC 42720) OX=306902 GN=CLUG_00390 PE=3 SV=1

MPFETFYWNAKPSLNGRNGKSVMLISPTSCSYHSHIAHFTISLISSQTCQLPNIFSENTM

QIDIQPSLTGLAEIVAQDKDNVLPPNIYPIYKYMATEDLTVHSAYLRLADLNNDKRSETF

LFESSVNGDSVDRYSFIGLRARKIIKTGDNEAVFAPEFCNVDPISVLEKELSNCRQAQLP

GLPPLSGGAVGYISYDCIKYFEPKTRRDLKDTLQVPEAVLMLCDLIVAFDHVYQRFQIIN

NVHVDPETDLEQAYSAAVKEIRWVEQKLKEQRPLEELNPHQPPIRPHQTFTSNIGEEGYK

RHVTTLKAHIKAGDVIQAVPSQRVARPTSLHPFNIYRHLRTVNPSPYLFYVDLVDFQIIG

ASPELLVKADKKSQITTHPIAGTIVRGKTPEEEERNASILRSSLKDRAEHIMLVDLARND

VNRVCTPESNRVERLLTIQRFSHVMHLVSEVTGVLRPDKTRFDAFRSIFPAGTVSGAPKV

KAMEIIGELEKEKRGVYAGAVGHWGYDGKTMDTCIALRTMVYKNGVAYLQAGGGIVFDSD

EYDEYVETMNKMRANNDTIVQAEKIWIQKVGSEEA

>tr|C4XWS2|C4XWS2_CLAL4 5-methyltetrahydropteroyltriglutamate--homocysteine S-methyltransferase OS=Clavispora lusitaniae (strain ATCC 42720) OX=306902 GN=CLUG_00395 PE=3 SV=1

MVQSSVLGFPRIGGQRELKKVTEAYWNKKVSVDDLLAKGKELRAHNWKLQKDAGVDIIAS

NDFSFYDQVLDLSLLFNVIPDRYKKYDLAPIDVLFAMGRGLQRKATETEAAVDVTALEMV

KWFDSNYHYVRPTFSHSTEFKLNTEAGLKPVVEYLEAKELGVETRPVVLGPISYLYLGKA

DKDSLDLEPISLLEKFLPVYKSLLTELYKAGAKTVQIDEPVLVLDLPESVQKLYQSTFEQ

LVGEGLPEIVLTTYFGDVRPNLAAIKSLPVSGFHFDFVRGAEQFDDVVSILNSKQVLSVG

IVDGRNIWKTDYAKASAVVKKAIEKLGADRVIVATSSSLLHTPVDLKNETKLDPVIADWF

QFATQKLDAVVSIAKEASGEDVSAIYAANAKSIKARQESSITTNPKVQERMATINESLST

RKSPFPTRLAAQKEKYNLPLFPTTTIGSFPQTKDIRVNRNKFTKGTITKEEYEKFIEQEI

ETVIRFQEEIGLDVLVHGEPERNDMVQYFGEQLDGYVFTTNGWVQSYGSRYVRPPIIVGD

VSRPKAMSVKESVYAQSLTKLPVKGMLTGPVTCLRWSFPRDDVSQKVQSLQLGLALRDEV

QDLEAAGITVIQVDEPAIREGLPLRAGQERSDYLNWAAQSFRVATSGVADSTQIHSHFCY

SDLDPNHIKALDADVVSIEFSKKDDANYIREFSDYPNHIGLGLFDIHSPRIPSKEEFVAR

IEEILKVYPADKFWVNPDCGLKTRGWEETKASLTNMVNAAKEFRAKY

>tr|C4XWS5|C4XWS5_CLAL4 1,3-beta-glucanosyltransferase OS=Clavispora lusitaniae (strain ATCC 42720) OX=306902 GN=CLUG_00398 PE=3 SV=1

MLFKSLLASALVAGAVADLPAIEIVGNKFFYSNNGSQFLMRGVAYQSDSANATADETFVD

PLGNPKTCKRDIPYLQELETNVIRVYALNTSLDHTECMNALSDAGIYVIADLSEPDLSID

RSDPKWDVTLLERYTSVVDKFHNYTNVLGFFAGNEVTNEVSNTDASAYVKAAVRDTKAYI

KEKGYRSIPVGYSANDDVDIRVDLADYFACGDEDERADFFGINMYEWCGASSFKASGYSN

ITAQYEHLGIPIFLFRVRMQQGHST

>tr|C4XWS6|C4XWS6_CLAL4 1,3-beta-glucanosyltransferase OS=Clavispora lusitaniae (strain ATCC 42720) OX=306902 GN=CLUG_00399 PE=3 SV=1

MTDVWSGGIVYMYFEESNKYGLVSIDDSGSVSTLDDFNNLKSELASISPSLASASSGVSA

SVTSCPSVGAFWAAATALPPTPDQDICNCMVKAAGCVVDDSVDEEDYGDLFGIVCGLVDC

SGISGNATTGKYGSYSGCDSKAQLDFALNLYYESNGKDSSACDFSGSASTQKPATASSCS

AYLSSAGVSGLGSLSGDIAAASAATTDAAASGSAAASGSGSGSASGAASTSGSSNSTSSS

SKKSGAASTNSAWTSVFGAMVLAVGFLFA

>tr|C4XWS7|C4XWS7_CLAL4 Mitotic check point BFA1 OS=Clavispora lusitaniae (strain ATCC 42720) OX=306902 GN=CLUG_00400 PE=4 SV=1

MRDVRVRICCVYMAQSVMYPQCQLFNMESDPKSSTIRNKNEKLRKWGEKAGPDADDILDD

LDGLDGNIANDFKTLRISGIDDVVATSSPVKRKAIRPSISPGFKMDTGAPDDEDFLFDED

FEGQLNPENIRLNQLRHTPLQKDNRFSPKTFSSASPVRRRSLSDYSEGTDTDITEMNEED

FEDIDDIFGKEESGIYSSGGKKQNNANISRASQILTEKKSQLKKQADLEDKELFEKYHHR

HGEEVNTLRLKDLKRYPVISNLDEDPLENERTVNYEYTRDDNESFEDGFDLNVPMRLEAT

KLKQFQSQPNIATLSSKISMPAFPKSLKPSAPTKFRSTMDLPAQLKDEHPLFNNSNKIIR

KLDRMPSFRHSKEADRKLSNDDRLNIDMERKKQQLLEKYMEITEKQKQLNSSPRRAARKQ

RATKKGVGLLKFLNDRTAAPIVNGNDKMRFNAVNRRWEGNDHDLLRFEEQNDTLKAKKQP

ALITSKEFSSNNAKISGNMLYDSENMRWVNLDPVAEENLDVFDEMSDLEPNDIPRYHKYP

VERGVSAFTQRTTSTVSSDRSSSQSSAGEEFQLSNRLVSRFEKEEQKIRKKTQNWFGHHE

QYRIDKERHFDSEYFWEIRKMVMDNSRG

>tr|C4XWT0|C4XWT0_CLAL4 1,3-beta-glucan synthase OS=Clavispora lusitaniae (strain ATCC 42720) OX=306902 GN=CLUG_00403 PE=3 SV=1

MAKLVRHLTSNEEIVSSNSAEGIIFPVLNRNTFSFCNLFVTQMLQNNFESVNPKIETESL

HDNLLYPAWCSDNGASVEIETIRSIFDRTALVFGFQADNSANMFEYLMSLLDSRASRMSC

TSALISVHADYLGGDSSSYKKWYFAAYYDLDRQYSDAKDIKRKWNQWPRFSSGVIPNSPE

FENDRSSFWGMDYAWRLQMSKYSEEELLEQLVLYLLIWGEANNLRFMPECIFFIYKCASD

YLFCQEEKPAAPEFSFLNDIVTPIYLYIRDQQFDLKDGKLCRKRGLDHAQIIGYDDVNSF

FWYPSNLEKLRIANDKTLHSIQKEHRYKELRNVQWKTVFQKTYLETRSWGHVIVNFNRIW

VIHLSAFWYYFVINTPALYTRNYYHALNTKSAPQVQLTVVALGGSVSCMVSLLSTIGEWF

FVPRSSLGCQPLLARFTLQVFLLLALTAPSVYILIFKGWNVYSPMGCAIGGCQLGFSLLT

TAYLSATPADKLFSFIKRKADPNTIKTTIFTSSFAKMTSKSALYSLLLWLTVFTSKFLES

YFFLTLSLKDPLNILLTMDTSRCAGDIWLKKLICQNFAKICAGLLLLTNFLLFFLDTYLW

YIICNCIFSTIIAYSAGTSIFKPWKNRFSKLPERIISKIVFSLNEKDGDFAITKIWNCIV

ISFYKEHLLSVEQVNKLIYQKETDDDKLAIGGFREPIFFNFQEDSASEKLSDFFASNAEA

SRRISFFARSLSSSLQAPIPIEGLPSFTVFAPHYSEKIILEIKELLKENEKSKISLLEYL

KKLHPAEWRAFVKDTKL

>tr|C4XWT3|C4XWT3_CLAL4 Mitochondrial carrier protein OS=Clavispora lusitaniae (strain ATCC 42720) OX=306902 GN=CLUG_00406 PE=3 SV=1

MSSDFLPPDEIKTIDKHSAEYVLRSGLAGGISGSAAKTLIAPLDRIKILFQTSNPEFLEF

RGSLFGLGRAARKIVASDGFLGLFQGHSVTLLRIFPYAAIKFVAYEQIRSILIPNDIHET

AARRFMAGSLSGLASVFFTYPLDLVRVRLAFETRALAHAARHPHHADFVAHHRGRIWAIV

RSVYAEHPPSRDSDPRWLKFMRKTVPAALVPLSNFYRGFGPTILGMVPYAGVSFYTHDLI

HDIFRSRMLAPYFVTERAPGSTRVVKKSRAGENVSSRDSRAPLRAHAQLVAGGLAGMFSQ

TSAYPFEVIRRRMQVGGAVDQGGFLSFRSTVRLIFAESGVRGFFVGLTIGYIKVIPMVAC

SFYVYERSKTLLGI

>tr|C4XWT6|C4XWT6_CLAL4 Alpha N-terminal protein methyltransferase 1 OS=Clavispora lusitaniae (strain ATCC 42720) OX=306902 GN=CLUG_00409 PE=3 SV=1

MPAKHINDTENSADSGVPDANINYSDAIAYWSSVPASVNGVLGGFGEQTPVPKADIIGSA

TFLRKLESRMACPDGARLTIDMGAGIGRITRDLLWKVSDRCDLLEPVEPFVAQMERELAD

VKRRGRLGDIYAQGMQEWRCSPEKMGQYWLVWCQWCVGQLPDDELVAFWQRCREALMPNG

TVVVKENIAPVEDVFDPTDSSVTRTDAKFRELFARAGLRLIASDVQKGMPRELYPVRMYC

LKAAEV

>tr|C4XWT8|C4XWT8_CLAL4 DNA replication complex GINS protein PSF3 OS=Clavispora lusitaniae (strain ATCC 42720) OX=306902 GN=CLUG_00411 PE=3 SV=1

MRLVDVLCDISPSSSSMGYYDIDDILADSEKVPCRFTLTVPGLGYLEGNPGKAIEKDTKV

ELPMWLAEVLATSLVSAGTEDSFVELLQPEFVGPRVLNAIRADPTSVDLHTIASNFYRLA

EKWSALFNDTELVEVVMAMLKERAVEIDNYASNTSNLVNSNFLYSLDEFERALYRATYES

KKQMRTWGNT

>tr|C4XWT9|C4XWT9_CLAL4 RNA polymerase-associated protein OS=Clavispora lusitaniae (strain ATCC 42720) OX=306902 GN=CLUG_00412 PE=4 SV=1

MYGCLTQVQHRLPLFSPPSAMDEPVDVSYYLGGASAESLSSLDVAIGNGEIVSIDELPED

AHELIGFLQAEKCEVKYWVMVAQAYAQEGKAGDAQKIVQAALDQAHFSDSDRDLVRSVLG

WVYLALASEGTDKSDNLARAAAEFANLDDSSPQVLSARAVMHLHEDHADQALQAFDKLLK

ADPANCFALMGKAQITLSKTQNYTSALKLYQQVLVLDPLMKPDPRLGIGLCFWFLKDRPM

ALSAWERALQLDPKSFKAKLLLNLASFDNVFTNSLSDEQFLEGYKRSLTELTHLQLEKTN

DSVILLALASFYFSKGKHDLVEKIVGKVVSAFSADDKPSRKLSSFATKVLSQASFWLGRV

AYAKEDFTQSQKYFHEAIRLNENNLLAKLGLGQSQLSRGSIEESLITFESILKTNPKCLE

VNYCLGVLYAQQSSKTKQEQAIHMLERYIRLSNNRGLAVENKNEAEALLNKEPVALNAYL

TLSKLYEPKDLSQSLVYLHKAIESRKQTGRDVPLEVYNNIGVFNFSKNSVDVAIENFELA

LKKLDDLKDAELQKDLKVTISYNLARSHEVSDQNKAIEMYNSLLQECPHYFSAKLRLLFL

DAVSTNETSKEEIEKEIKELLAQNASDLEIRSFYGWFIKTFGKKIGLKPDADTVHQKETL

VDFDSHDCYALISLANIYCVMANDLKSSKDEEKRKKYFVRAIELYTKVLSIDPKNVFAAQ

GLAIVYIENKEYHKGLDILRKIRDSLNDISVYLNLGHVLVELKQYSKAIESYEVAFVRYT

DSQDVKIMGFLGRSWYLRGIAEKNLSYLKKALEYSEEALNKHSGRNGSLVFNVAFIQFQI

AEFVSKLPVEQRTVEDINEAIINLNEAIQSLNKLASDDEKHPPFPKEDLKARANLGTNTL

LNRLNVCLEETKENIAQLNNKLEEAKRLREEEEARKAQELESVLAEKRTKEEELAKERAR

LQEQAQQWAEEARANNIVADDSDDDKAFSEEGGKKKGKKQANGKKKGGKRKKKDFINDSD

EEEEAQLTGGEDNAEEPPLGSDAEEEKPKATKRKRRAIEDDDEEKEEEEANGNKKKKAFK

SEEMVEDSSDGDDDGLF

>tr|C4XWU3|C4XWU3_CLAL4 ATP-dependent RNA helicase OS=Clavispora lusitaniae (strain ATCC 42720) OX=306902 GN=CLUG_00416 PE=4 SV=1

MAPKNGKKSNGGKKPNGNKPNGGKKPTGNKKTGGKFVRSKPPTLKQKNARQNKIIKADQL

SWKPVEIPDNLGDYSGFFGLEELEGVDVRMVNGKAEFIAKDSDVEMSEEEEEEQEEGEEK

EEPAEEPADAEEEQTEAEGEEEFTGFDDEVKSDQQEKKSQKKASKTESKKNKKDKKDVPE

KDDAALEKEDEEEKLVAFSVLDMPLPDDDIDLPQWQNVPLSAYTLSGLQALGFKQPTPIQ

KSAIPLALEGKDVIGKATTGSGKTLAYGIPILEKYISKIAEIKTLRQKKTIPAPTGIIFA

PTRELAHQVSDHLNKIAKYAPLASNGIVSVTGGLSIQRQERLLSYGPGIIVATPGRFLEL

IEKSEEFTKRMALTEIIVLDEADRLLQDGHFEEFERILDLLGKNRDKAYGWRWQTLVFSA

TFSRDLFFKLDKSGKGTKASSGALVANDEILQLLQKKLKFKDSKPALCDANPKEIVSGQI

TEALVECGPAERDLYLYYFVLMYPGSTLVFANSVESVRRLVPLLENLNIPAFSIHSSMIQ

KQRMRALERFKEATQKNKSAVLVATDVAARGLDIPSIDHVAHYHLPRSADTYIHRSGRTA

RAGKEGVSVMFCSPQEASGPFKKLRKLVASSAEGGKFNAKADVKLLPIEYDIVQQIRERV

RMAGKLAGSTMSNTATRKENSWLKQAAEDLGFDNLSDLDEFEDDVIKKQKKRLERSKLTK

EEQKALRAQLKESLAIPLRKNHRRSYITSGLENLAHQIVSGATSETVLGRKSIKALDELK

QTKGKARVEKKKQKQKQKARS

>tr|C4XWU5|C4XWU5_CLAL4 25S rRNA adenine-N(1) methyltransferase OS=Clavispora lusitaniae (strain ATCC 42720) OX=306902 GN=BMT2 PE=3 SV=1

MLRRSRSITQNAKAPKSLKPQQARQLIRRFHVLLKNKATIIKSLAREDASLTENNYKDIL

GKKYAAAYGQFKLTKHLEQFQPDGSWEKLVPMLARIDAEIDQRGGLHVYQMASTVGQNES

RGGDSSKRLVAWFKELGRTARSSLEIGCLSADNAISTCGLFGTVTRIDLNSQNPKIEQQD

FMQRPLPTSDSDKFDVISCSLVLNFVPTPAQRGAMLRRITQFLRPESSSLFLVLPLPCVA

NSRYFDASVLEKIMQSLGFRQVRYYEAKKVAYWLYDWNGKCSEIKLRKKEIHKGSSRNNF

FIDVNNQSSDEA

>tr|C4XWU6|C4XWU6_CLAL4 Major facilitator superfamily (MFS) profile domain-containing protein OS=Clavispora lusitaniae (strain ATCC 42720) OX=306902 GN=CLUG_00419 PE=3 SV=1

MSQEDINTTGTTPANGSILEKEKNNEEQLVDVLPNKGFKDYISISVFCLFVAFGGFVFGF

DTGTISGFVNMPDFKRRFGQKRKDGSHYLSNVRTGLVVSIFNVGCAVGGIILSKIADMQG

RRMALMVSMVIYVVGIIIQIASQHAWYQYFIGRLISGLAVGMVSVVSPLFISEVSPKQIR

GTLVCCFQLFITMGIFLGYCTTYGTKKYSDSRQWRIPLGLCFAWAIFLVVGMLSMPESPR

YLMEKKKIDEAKKSIARSNKISEEDPFVHTEIQLIQAGIDREALAGKASWKELVTGKPKI

FRRVIMGIMLQSLQQLTGDNYFFYYGTTIFQAVGMNDSFETSIVLGIVNFASTFAGIYAI

EKLGRRMCLLTGSASMFVCFIIYSLLGSQHLYKHGTSNVSSNTRKPTGDAMIFITCLYIF

FFASTWAGGVYCIISESYPLRIRSKAMSVAQAANWMWGFLISFFTPFITSAIHFYYGFVF

TGCLLFSFFYVYFFVYETKGLSLEEVDELYASDTLPWKSAGWVPPSLEHMAHATRYAGEE

KPEDEQV

>tr|C4XWU7|C4XWU7_CLAL4 Major facilitator superfamily (MFS) profile domain-containing protein OS=Clavispora lusitaniae (strain ATCC 42720) OX=306902 GN=CLUG_00420 PE=3 SV=1

MKKKPLSINHHLHNMSQEDVTSGSTPINGSLLETEKKLEENLEHQMPEKSFKDYISISIF

CLLVAFGGFVFGFDTGTISGFVNMPDFKKRFGQHKADGEHYLSNVRTGLIVSIFNVGCAV

GGIFLSKIADVWGRRMGMMVAYGYLRRRYHCPNCISITLGTSTLLAVLYSGLAVGTISVV

SPLFISEVSPKQIRGTLVCCFQLFITMGIFLGYCTTYGTKKYTDSKQWRVPLGLCFAWAI

FLVVGMLSMPESPRYLIEKAKIDEARRSIARSNKLSEEDPAVHTEIQLIQAGIDREALAG

KASWKELITGKPKIFRRVLMGVILQSLQQLTGNNYFFYYGTTIFKAVGYD

>tr|C4XWU8|C4XWU8_CLAL4 Major facilitator superfamily (MFS) profile domain-containing protein OS=Clavispora lusitaniae (strain ATCC 42720) OX=306902 GN=CLUG_00421 PE=3 SV=1

MGMMVAMVIYVVGIIIQIASQSAWYQYFIGRLISGLAVGTISVVSPLFISEVSPKQIRGT

LVCCFQLFITMGIFLGYCTTYGTKKYTDSRQWRIPLGLCFAWAIFLVTGMLSMPESPRYL

IEKSKIDEARHSIAISNKLSEEDPALYTEVQLIQAGIDREAMAGKASWKELITGKPKIFR

RVIMGIVLQSMQQLTGNNYFFYYGTTIFQAVGMKDSFQTSIVLGIVNFASTFVGIYAIEK

MGRRACLLTGSIAMSALFIIYSLLGTQSLYKHGTSNDTSNTRKPTGNAMIFITCLIIFFF

ASTWAGGVYCIVSESYPLRIRSKAMSVATAANWMWGFLISFFTPFITSAIHFYFGFVFCG

CLIFSIFYVYFFVYETKGLSLEEVDELYASDTVPWKSSGWTPPSVEHMTQSAGYADQGKP

TEERV

>tr|C4XWU9|C4XWU9_CLAL4 Aminotransferase class I/classII domain-containing protein OS=Clavispora lusitaniae (strain ATCC 42720) OX=306902 GN=CLUG_00422 PE=3 SV=1

MLRRASRPFSVAARLAKWNEIPLAPPDKILGISEAFQRDANSKKINLGVGAYRDNSGKPI

VFPSVKKAEKILLEKETDKEYTGIIGSKAYQTAVRNFIFNNSGKDVSGAKLIKDNRIVTA

QTISGTGSLRVIADFLVRFYSSKHILVPKPSWANHVAVFSDAGMSTEFYTYYDVKANGLD

FESLKSSLSAAADESVVLLHACCHNPTGMDLTPEQWDEVLAIVQEKKLFPLVDMAYQGFA

SGSPYKDIGLVRKMNKLVAEGKINSYALCQSFAKNMGLYGERTGSVSIVTESGEQSAAVE

SQLKKLIRPMYSNPPIHGSRIVETIFSDEALLNEWLADLDAVVSRLNVVRDKLYEKLDKS

NYNWDHLLKQRGMFIYTGLSAEQVVELREKYSVYATEDGRFSISGVNEHNVDYLADAINQ

VIKK

>tr|C4XWV0|C4XWV0_CLAL4 phosphoglucomutase (alpha-D-glucose-1,6-bisphosphate-dependent) OS=Clavispora lusitaniae (strain ATCC 42720) OX=306902 GN=CLUG_00423 PE=3 SV=1

MFQTIATTPFQDQKPGTSGLRKKVTVFQQPHYTENFIQAILEAIPEGAENATLVIGGDGR

YYNDHVIQLIVQIAAANGVSKLIVGQNGLLSTPATSHVIRSRHATGGIILTASHNPGGPK

NDMGIKYNLANGGPAPESVTDRIFAISKKLVQYKLWPLDKVDLSKIGTFTAGPITIEVID

TTADYVELMKEIFDFPLIKKFVESSSDFSVVFDALNGITGPYGHRIFVEELGLPESSVQN

CVPKPDFGGLHPDPNLTYARTLVDRVDKENIAFGAASDGDGDRNMIYGAGAFVSPGDSVA

IIAEYADSIPYFKKQGVYGLARSMPTSGALDLVAKHKKLNVYEVPTGWKFFCSLFDAKKL

SICGEESFGTGSNHIREKDGLWAICAWLNVLADFSVQFPEEKASIKNVQEKFWEKYGRTF

FTRYDYEGVSSEGAEALVADFAKIVESSKPGTDLGSGHVVAEAGNFSYTDLDGSVSKNQG

LFVKLTSGLRFIVRLSGTGSSGATVRLYLEKHTSDASKYGLGASEFLADDISFVVELLKF

QHFLNKSEPDVRT

>tr|C4XWV1|C4XWV1_CLAL4 GATA-type domain-containing protein OS=Clavispora lusitaniae (strain ATCC 42720) OX=306902 GN=CLUG_00424 PE=4 SV=1

MSEIQRFPAMVDANPVHPNAKTTTQINNGTTTTKSNISSPVCRNCKTQTTPLWRRDETGQ

VLCNACGLFLKLHGRPRPISLKTDTIKSRNRIKQPNFAKSGPNTPELKSKDAKSGKRSPK

SAKKKSAPSPPPEMHTHSPLATHTRAPFPQGFAPSGHVAGLPHQVQSLHYPSSTPTQFAP

GLQRITSPLLLSTTASVSNARATSSLASPKGLSAVQAAGALENMSNELGPSATFKSGVSL

NGGSLMGGMKRSATPVLSTPSTALAGPKLPALSTEERNNVIASPSFGPQFHLDHSTKSEP

SDQPRPALPPLQHVQPAQAQLPRFQSNFGSVTGSGSSTPVAPISAPVAAPIASLTGGNEQ

RSSSGASANGTGSATSNNNTGNNEANSQHGNSGPANSGPVKSSPGNSASGNSGPSSGPGR

DPSGSDNARASNNGNNNGNNNGNNNNGNNGNGESAYEINLLKTRISELELVNDLYRTRIM

ELEAMEQAARLREGSMRKRLDEILNLQGQDSVLQRALHEPSKRPKLG

>tr|C4XWV5|C4XWV5_CLAL4 37S ribosomal protein OS=Clavispora lusitaniae (strain ATCC 42720) OX=306902 GN=CLUG_00428 PE=3 SV=1

MFRSAARFFGTKSTALDAAPVKIQPYLAAKQFVPEDASKSKVRALRRRENRMKRQIIRDV

NNLKKHSLKREEFKVDPVLGAENNKFISRMYQELEDPANLAYGYNREEFEKLLYGAQKAA

LDSSIGGEVITEQITKTEEKKKRALLTILNMKNSNAADKRKLATEMARKEFARFEGDTGS

PEVQAAIMTVKIHFGFEHVKKAPKDKAHIQSVRELVQQRQSILKYLKRDDPEKYYYTIAK

LGLTDDVVTREFQHGQTIFPGLPGVGRQAVGQVVGEAKEERTALC

>tr|C4XWV6|C4XWV6_CLAL4 Kinetochore-associated protein OS=Clavispora lusitaniae (strain ATCC 42720) OX=306902 GN=CLUG_00429 PE=4 SV=1

MCAYSHSGVYLLVILLHSGMETEKIRYDRLNQVVKKAVEQTIKRSLMPEQLEKCFPTISK

MDGGPEALEMARKQMQKYFHSTCFKQFEHIFENRDIHRKLDELDEIIHDAQQRRDLSSAE

SDISVDTLTASQLIEATVASTRQDSVKKLTMIYDQLVLDNKQLFQELRELAQQSDSLKQD

IEGSIQALSSGIDDLKRQNLDSSVDTLMAEVFTES

>tr|C4XWW1|C4XWW1_CLAL4 Importin subunit beta-2 OS=Clavispora lusitaniae (strain ATCC 42720) OX=306902 GN=CLUG_00433 PE=3 SV=1

MWTADPQALEQLAAIFKATLSSSKAERSSANDALASARLQPHFENYLCDLLVRDLGVSAD

VRAAAGINLKNCVLKQSSADRSYLLATIFAGLRSSHNMVRNITGNVITSLFSACGPSGWP

QALPSLLDIAADESVPLPTREAAVSALAKICEDSGSSLDDASLAALAQQLLHVTQSPQAS

ASLRSGAVLCLNQLVPLKSSHAFVEQYLQALFTLTGDHDSGVRKNVCSAFVSIMECKPQT

LVPHMGGVVSFCVHSMQDENEDVAMEACEFLLTLADSPEKAHRVSFRPHLQQVVPVLLEK

MVYSEEQIFLMQILDEKDDARVADRDEDVRPNAVRSKNAHSVSKTPAAKKIEQDSDADSD

DDGDSDDDDDSDDELDSWNLRRCSAATLDALSLDYPQEVISVALPLLQEKIVSPEWPVRE

AAILAFGAISKSCVDLARDKLPTLVPFLVERMKDAETRVRSIACWTLSRYATWVCAEAHE

GGTYSSYFPPTFEAVVGLALDKKKIVQEAACSALASFVETADVELLDRYVGALLAHFAQC

FASYQRKNLLVLYDCVNTFVDKIGPEVFGRSPEYANTLLPPLFANWESLQDDDTDLWPLL

ECMSVVASTMGEAFAPYAVPVYERAVKILANAISLNQHVHTDPLIEAPEKDFIVTSLDLI

DGLVQGFKAHSVELMKQHGANVMELVLVCFEDHDEDVRQSAYALLGDLAIFACHDMVQPY

MDRIVVCIGHEINNRSYSSYAVTNNAIWSFGEIAIKADPDSIKPYVGNIVGLLVPLLKSA

DTQETLVENAAICLGRLGLAGGSPEIAALLPDFVYSWCAQMMYVMENEEKETAFLGFLNT

LQLNPDQGLGGLNNQQGRKNLAVLVSCIGNYFEPSDKLKEMFYQVLMSYKSLLGDAWETQ

VLGLVDADTRGFLQTSYGV

>tr|C4XWW4|C4XWW4_CLAL4 Peptide hydrolase OS=Clavispora lusitaniae (strain ATCC 42720) OX=306902 GN=CLUG_00436 PE=3 SV=1

MADENSPLLESSFDRDMDDFDNQSKRTTESQNVERMLRQRFWWFCILGVAAIVALQLSFL

PRTSCNRDFRRWYDLHLTKTDVKRLFLVQSQRGRPEDDGLTVEDHVDHWLGAFSMLNAKT

TAGISASTAPELAAMVQQNMRDFGFETNSHIYPLSPLVKEPLSSAVRLVDVQSGRVLYNA

KTHEAGSPTPAFFPFGANGSVEAPLTFARAGTPEDFSALERHGLSVAGRIVVFSHSSTSD

YLLADKIALAEQRGCAAVVVYGDVDAEDAISRNFKPFAVPEPGIRIPLSYSAAQPILSTL

GPAMEPFQNWPYAPQPLDDTLCLQVAAQFAPDSLYATNIVGSLEGVLNDGVILVGASRDI

LTSSNPLSGHAIMLEVMRRFQLLRNMGWRPLRAIRFVSWDASRSAAQGSHAMVLDSSVLK

TNLPVLAYINLDEDVVMGDHFSVDANPLFNNILRYTAKFVPFPRNSTHYRRISKNDNDMI

SDDEEDDDISLYHYWYKQSKAYINNRVGSAFAGKDASSFQSSFAVPTINFKFAQSPNHND

SVYVPESSFYSLDWLSQNVDKSYALHSSLVRFLGLLVLSLGEHEVVDYKTADLFQQAQSF

FSDFVDANKDTLKGWEDEVVQDSLLSKSTLLADLKDRMDGDADKHTQFKFRALVQQMKEI

FGELQSQSEIYDAYNVDVQEMWIEDYPWYKMFKKAQIYAKFKVTNYKLLRIEKGLTLTPD

ETEKTGGVNETHHLMYEVPQGQHSRAELHQRGAFASMYEAAETGNMERMIKIMVGSYERL

KSIYKKIS

>tr|C4XWW6|C4XWW6_CLAL4 Phosphatidylglycerol/phosphatidylinositol transfer protein OS=Clavispora lusitaniae (strain ATCC 42720) OX=306902 GN=CLUG_00438 PE=3 SV=1

MVAFNRLSTALLAASAKALSLVSLFDNAVDSLLPQVVTTFPNPDEKPVPGNSPIVQCDAT

QPQLLDLQKVVIDPNPPAKGENLTFVATGFLAQDIVDGAYVDVDVRYGFIRLIHQTYDLC

EEVTNVDMECPIKKGEQVISKSVEIPAEVPPGKYMVNARAYTGEDVLIACLSATVEFPPN

>tr|C4XWX1|C4XWX1_CLAL4 Vacuolar protein-sorting-associated protein 36 OS=Clavispora lusitaniae (strain ATCC 42720) OX=306902 GN=CLUG_00444 PE=3 SV=1

MSKESFLHVWQSVAINKSSRPVLSEDEHTLYVKEGVGLYQGRAKIVNHQDGRVYLTNKRI

VYVDARQAVAVSLRDTARAEHVERFLRASPKVKVYLARGGTRAAPEQWRWTCRICSFRNT

VPSKEGAQCVACGIRPAPNELTTQTNTTTTNTNTTTTNTTTTTATNTNKSTNTNTISTNA

TNTNANANTNPTSANTNTNTTFVSGILDASVSSLSRNNSSATLETSAAKTPTAAKGLACP

ACTFLNHPSMRACELCSTPLQAAAPDPPRSDANPARLALDGPEEYTGGAPYIKFSFRAGG

DTDFHTHLVAAIDAEKWAALERANKVGKGEVQPQEPARRTGGGILHLEQQSENRRRENEQ

VLASSLDDLQQLMAQAQDLIALATSFRPVVRKNAARDLARDMRSLAAACGAVRAGSALFH

AELARHMCEFLLARDLSSAAAMITMPDLFARYNRFRVSAQGLAAGLVSTEDIRTSLELAN

SLALPVQIKKYSSGLEVVARRTLESEGVAARIVAFLKDVQDGTSAETYFEGDTDRQWRGA

SVAEVAAHFGWAYAVCVEELDRCIDQQLVVYDRHVSGTFYYANAFGTWAQETRCKV

>tr|C4XWX3|C4XWX3_CLAL4 Branched-chain-amino-acid aminotransferase OS=Clavispora lusitaniae (strain ATCC 42720) OX=306902 GN=CLUG_00446 PE=3 SV=1

MHLSTQKSTHTSTYKQPVLFCASRPHFSLCNFLLSSGATMSFLARSCFKPANFSRFVSMS

APLDASKLKITKTTTPKPKLANDKLAFGKTFTDHILEVEWTAEKGWAAPTITPYHNFSFD

PSTIVFHYGFEAFEGMKAYRDKQDRIRIFRPNKNMERMNGSAERIVLPTFDSEEFIKLIE

EFLRLEQDFVPRGEGYSLYLRPTIIGTTATLGVSTPDKALLYMIASPVGPYYSTGFKAVS

LEATDYAVRAWPGGVGNKKLGANYAPCVKPQLEAAKRGYQQNLWLFGEEGYITEVGTMNV

FFVFQDASGKKELATAPLDGTILEGVTRDSVLTLARARLPEDEWIVSERKFTIGEVKERA

AKGELVEAFGAGTAALVSPIKAIGWRGEDINVPLAGGDAGELTMQVADWIKKIQYGEDEF

EGWSRVVV

>tr|C4XWX7|C4XWX7_CLAL4 Very-long-chain (3R)-3-hydroxyacyl-CoA dehydratase OS=Clavispora lusitaniae (strain ATCC 42720) OX=306902 GN=CLUG_00450 PE=3 SV=1

MPQSAHPQRWLIAYNSISASLWSVVLFNTLFLGALLGQPLLFEKSSTILTLVQSCAVVEI

YNSVFGIVKSPVFTTVSQVLSRLLIVLGIVQVLPESPANHHWVYITLCLSWSITEVVRYS

YYASNLRDSSAIPSWLTWMRYSLFYVLYPTGVASEMSMIYMSLGEAEKVVGKWYSWLLFA

ILFTYPPGLYTLYTYMIRQRKKVLGKKPKKTD

>tr|C4XWY0|C4XWY0_CLAL4 Adenylate cyclase OS=Clavispora lusitaniae (strain ATCC 42720) OX=306902 GN=CLUG_00453 PE=3 SV=1

MSFLKRDKSRTSLRFPGLAAGQSDSRPTFGLRRAHTERPKESHLWDFHERETEGPEKEDQ

GADGEDEKEDGARLDRWPGETENMDERKNAGGNERDGDENHEPENTEQTDEADKTDKDKT

DKTDKTDKTDKTDKLESVQSRGSQSEPNHASQSEYHESQSEHNDYEQNQNNQNNQNNQNS

QNTSVSQNHESQNLSVDPYADARSLHSTHSTHSDTHGEPRDRRDPPRVVRDDYRGFHRSG

VHAIVPPLAHPIKPSFRKRGSGLLGKWMHQRPGGNASSASSMAGEPDRAKHKFRLPSISS

HHVSSSSSRPPSSDESDRRSSVQSGRGSVSGPHLSAGGMFDLDLSAADLSAIVKKPASAR

PSLAKTASAGSMEEDRHAWKAPDSWDVTEETPRDASDVSSSASASAEASRRSSEVADARR

GDSRPSFPVRSLPVLFGSVAGRTGNAGGPNHIIRVFKEDNTFTTILCPVDTSTADLLSIV

QRKFFLESISNYRLALYVANNTKVLEPFEKPLRIQMGLLALSGYSESDNLRMIGREDMSF

VCKFVVENMFLRNLTHEEESLLSKDYVDVNISNLKLKTIPIIFHQHTYEIEKLNVSNNPA

IHIPLDFIQSCTNLSSISFAHNGCSKFPTNFLEATNLVSLDIGSNFLDEIPPRFNCLQHL

QHLKLNSNQLYFLPSTFGTLSNLVSLNLSSNYFQVYPECLSDLVNLQDLDLSYNDLSTIP

SSISNMTKLTKLNLCTNKLSGKLPDCFKNLSNLKRLDIRYNKITNVDVLGSLPNLEVLYA

SKNNIAGFSDKMESLRLLHFDRNPITNLEFQITLPRLTVLDLSKAKITALPGEFISKMPH

IEKLVLDKNHLVTLPDELSALPRLTHLSLYANNLQHLPDSIGQLVSLQYLDLHSNNIETL

PSSIWNLKSLSTLNVSSNMLSSFPSPSLELASKMSSPKTGNDSAGKSNQNQVMASSAQFN

SLADSLLSLTLADNRLNDDCYEAISLLVNLKQLNISYNDLLEIPDGALTRLTKLTELYLS

GNNFVKLPVEDVEDLADLRLLYVNNNKLTSLPSELSKLKNLMHFDVGSNHLRYNISNWPY

DWNWCWNKKLKYLNFSGNKRFEIKQSHLKNPDTGEDFDSLLVLKDLKVLGLIDVTLTTPS

VPDQSVDKRVRTTASELDNVGYGVSDTMGIRDCVSFRDVFIQKFRGNENEVLLCSFDGKL

SKPQTKGHLISYLAKQIFVHNFTAELNKIKSDDDIHDAMRRAFLSLNKEINSALAAKKGG

YFTNNPTWPELSNLDLEDDGLAGCAMSVLYLKDHMLYCGNVGDTEVVLTKNNDEHQILST

KHDPTSRHEFERIRSSGGYVSGDGALDGALKVSRGVGFFNYVPHTNSCPSLTTVKLSGMD

DLIIVASKILWDYLTYDLAVDIVRQEKDDLMIAAQKLRDYAICYGATDKISVTVICVGDQ

RKKRKNMGSMFKNLGRDVEFFANKKRRDRGAPAGDTALRRLDSEIDPPVGELALVFTDIK

NSTLLWDAYPVAMRSAIKLHNTIMRRQLRIVGGYEVKTEGDSFMVSFPTPTSALLWCFNV

QNQLLNEDWPTEVLETNECCEVTDNAGNIIYRGLSVRMGIHWGSPVCELDMVTRRMDYFG

PMVNRTSRIESSADGGQIAVSSDFLRELKQLFKLHDEIQSKKLTLEDAYEGNVGVGQIIE

KEIASLDAKGYSFFELGERKLKGLETPEMITLAFSKNLEIRYEIFKEKANRSTEYPTRII

GALPVDAVYRLRTISLRLENICSALSSGLNNDETFSQSSSDVISRKVGSDFLERDIVGLF

NHIVTRVENCVATIELRQSLQTFRGEPYFDRRSVWELMDEVKQLLAQMPQQNAIL

>tr|C4XWY2|C4XWY2_CLAL4 Glutamine synthetase OS=Clavispora lusitaniae (strain ATCC 42720) OX=306902 GN=CLUG_00455 PE=3 SV=1

MASITEQTAILSKYFDLDQRGKVLAEYVWIDSEGNTRSKCKTLSKRPTSVEDLPEWNFDG

SSTGQAPGHDSDIYIRPVAFYPDPFRKGDNIIVLTECWNNDGTPNKFNHRHECAKLMAAH

ADEEVWFGLEQEYTLFDQYDNVYAWPKGGFPAPQGPYYCGVGTGKVYARDVVEAHYRACL

HAGINISGINAEVMPSQWEFQVGPCEGIKMGDELWMARFLLQRVAEEFGVKISFHPKPLK

GDWNGAGCHTNVSTKAMRQPGGMKDIEDALSKLAKRHKEHLALYGSDNDLRLTGRHETAS

MDAFSSGVANRGASVRIPRSVAKEGYGYFEDRRPASNIDPYLVTGIMVETICGAIPDADM

TKEYARESE

>sp|C4XWY8|UTP25_CLAL4 U3 small nucleolar RNA-associated protein 25 OS=Clavispora lusitaniae (strain ATCC 42720) OX=306902 GN=UTP25 PE=3 SV=1

MKRPGSNKRPAKSKDGAESRPKRANRGRQQLRTITRTARRDVDAPVEETPESSESEAEEA

APEEKKFEQGKAYDALLTLLQSEHPEKSDKKKTASRHEEDDVAGVNLEEDDDDDDEEEEE

EENDEVEDDANEHGDPFEVHFGVDDEAVAAKAALLSARWPVAHKAKVGAYAVTTHASPGE

KTAACEAPRAQTLGAPVKKRVADAFAARHPAPFSPLEQELAHHIFSYRDVLYASRTYDNT

VHRKLYAAHVLNHVFKTRDRVLKNNDVLKNYHSAQQQGKATGPEPEPRDQGFTRPKVLIL

LPTRNAAYEVVEQLIGMSGAAQQENRRRFKAQFHADGGPPDSKPADFRAMFHGNSNDFFS

IGLKFTRKSVKLYASFYGADILVASPIGLAMILEDPKQAKREYDFLSSIEVLVVDRANEL

EMQNWDHVHTVLRYINKIPKDFHGADFSRIRMWAINDHARLLRQTLVFCDFLTPSVSNVI

THSQNIAGKTRFRPETTAQTCVMNSVGLRLKQIFQRFDAPDPQSSVDARFRFFVNSVLPS

LSKQTSYDDGLLIYVPSYFDYVRLKAHMKTHTKLDFSAIDEYSSRAKITRARHFFSTGKT

KLLLYTERLHHYRRFELAGVKNIVLYGPPENPLFYKELLRFVGKSVFKGDADTDLSFIKT

IYCKWDAAALERICGAEKAAVLCNSVNEMFEFR

>tr|C4XWY9|C4XWY9_CLAL4 ERCC1-like central domain-containing protein OS=Clavispora lusitaniae (strain ATCC 42720) OX=306902 GN=CLUG_00462 PE=3 SV=1

MAENIDAASFASILAGVRQMREEYDTRPPSENQANQNQANQNQANQNHTSQNQANRPNQT

NQNQTNQTNQTNQTSQNQTNQNQNQTNQANQNQNQAQIIQPGQANRNSPGLHPSIHQPNH

LRERQTSRQSSPSPRGTPGHSTRQTSRPPPSAAAIQVAPSQKGNPLLDSSQMKLTPWAYS

SDILSDYYISATVQVLFLSLKYHRLRPEYVWRRIEKLKGSLVGQKDDTLRVLLVVVDIDA

PQESLRHLSGICVKHDLALVVAWSFEEAGTYVACLKQHEQTRSHVGSSLQGVRKGDYNSS

VVGALTTVRAVNRTDVAGLLAHCRSFKEIVLRSAVGGVSVPGIGERKQQTLHDAFTEPFV

WNKE

>tr|C4XWZ3|C4XWZ3_CLAL4 Carboxypeptidase OS=Clavispora lusitaniae (strain ATCC 42720) OX=306902 GN=CLUG_00466 PE=3 SV=1

MKASQLLLWAAALASANAFSVPYLREMEQFFFGVSPEIEIGATKFETLQEQIAGSGDINI

QYAWKDLISEVGEEKAVQLFSKYQESENVKKDRMNNKHNEFAKLSKTHTETVRSQKFSNY

ALRVSESAPEILGLDTVKQYTGYLDVEDLGKHFFYWFFESRNDPENDPVILWLNGGPGCS

SSTGLFFELGPSSINATLQPVRNPFSWNSNASVIFLDQPVGVGYSYTDSEQISSTAAAAK

DVYIFLELFFQKFSKFAQNKFHIAGESYAGHYIPSFAAEIINNADRSFDLSSVMIGNGIT

DPLIQYKYYRPMACGEGGYKPVLDVETCERMDKDYPKCAALAKICYDAPSALTCVPATYY

CSSRLENPYSETGLNPYDIRKKCTDEGGNCYVEMDYLDDYLNLDYVKEAVGASNIDIFTS

CDDTVFRNFILSGDEMKPFHQYVAELLEKGVPVLLYEGDKDFICNWLGNHAWSDALDYSK

HDFFEVQPLRPWHTKEGKLAGEVKNYGIFTFLRVYDAGHMVPFDQPVNSLDMVNRWIAGD

YSFGY

>tr|C4XX01|C4XX01_CLAL4 BRCT domain-containing protein OS=Clavispora lusitaniae (strain ATCC 42720) OX=306902 GN=CLUG_00474 PE=4 SV=1

MSILTVLKKVDLLTVDSGPPLFGFAIEIFSLPSTFILSSFSYNSLTLLFLLYIAITTFLF

FLPSFLFFPSLLSVISFPPFSSSFFLLIVFPLSYSYIHVMLHSFTSKRANFYPTDAFDLN

SPCYWRLMILQPARSRAPVSSSISTTIINTSAVCRSLIFLSMSKPFQGLTFCCTGIQSSQ

RHEVADKIVALGGTHYTDLMSSVTYLVVGSRNTEKYRYSVRYRHDVTFLSVSTILDAHAR

WTAGDDVSAALLRVQLPLFSSFVVCVARVDRPSSEEVSRLFGERFRTPPRDAIPPHALKD

AFSAQDIVSVLAHHGAKVSSTLTPACNVIVGTDAAGRRFTMAQQWGIPAVHPLWIYDSCL

RGAALDLDDYRVSTSGTNLYNNASFVWKKLYALRVQRAAQTAPDDEPQRIDKTALKKSSH

IWSSIMDRTRSHTARLVRDSTWDDASEDEEETPVLLNSSKTKAGTSSSATIKEAATSTLF

LGLKFLAVGFSIPQQKVLQRVVDSHQGEIAEGADDETITHILLLVRNGPQANLMLSMLPS

AMKRRINSKEVAVVTDWFIERSIFYNRICLDSWCKPLQGLVPSATRYKVCISGFTGVELL

HIEKLIGYLNLEFCDVLNANRDLLIININLFKPAFIKNSPKLFEYKHKDILHCPVYQNGD

DSKSVSMISAKNKISAAKKWGIPIVSVAYLWEMIERSTNKANLQVPDILDLSWCIFAPQT

MARPTTLLDYVRKMSNNFSTQTPSEGTEEMQVQLPSPRKAKNKLKYGRLAGGGESLTEKL

KKAREDNEDTLQKGPDDTHDSDDMPTQVGYENQDSLHDKQELMRKLEGPERATKRRRKVT

>tr|C4XX07|C4XX07_CLAL4 NADP-dependent oxidoreductase domain-containing protein OS=Clavispora lusitaniae (strain ATCC 42720) OX=306902 GN=CLUG_00480 PE=4 SV=1

MTTLISSVQLTRATKYKLNDSHYIPVAGFGVYKLPKNEAGDLVYEALLQGYRHIDTAVYY

ENEEECAEAIAKFLKDHPDKAAREDIWFTTKIRPDQLGYEETKKAVAEIAARVKPHIEYV

DLMLLHAPSKSKKTRLDSWKALQEFASDPANGVLKIKSIGVSNYGVEHLKELMEWDGLTI

PPSVNQLELHPWLPRQELRAYMLKHGILAEAYSPLTRGIKFKDPELIALSKKFNMSMAEI

LLKWSYLQGFIVLAKTATKERIKENLSVLPEAPKDDPLEEKTNWGKVDLDLELLKALDKP

DSHEVLIIQDDPTIYHNTDE

>tr|C4XX12|C4XX12_CLAL4 Squalene synthase OS=Clavispora lusitaniae (strain ATCC 42720) OX=306902 GN=CLUG_00485 PE=3 SV=1

MGKLLQLLAHPTELRAAVQYFCFKSSMYPLDLKNAPESLKKCYVFLKLTSRSFAAVIEEL

HPELRDAVMIFYLVLRALDTVEDDMTIDPNVKVPLLRSFHEKLSLKDWNFDGNGPNEKDR

IVLVEFHHILTEFHKLRPEYQDIVKKITKKMGNGMADYILDENFNTNGLETVKDFDLYCH

YVAGLVGEGLSDLLVLARFADNQVQAENYRLSESMGLFLQKTNIIRDYHEDLEDGRSFYP

REIWSKYTDSLPNFFKKPEEREKGVECINDLVLNALNHVIDVLTYLSLIRESTSFNFCAI

PQVMAIATLAEIYNNKKVLETNVKIRKGTTCKLILNSRTLPGVVDIFRHYVRVINHKSDV

RDPNYLKIGIKLGEIEQFCETMYPHKASLPPYVQQKRKPINENIINRGNFDRDGAAMISV

EEFKCNIALAMGATTIAALVFFLYSALF

>tr|C4XX13|C4XX13_CLAL4 GTP-binding protein OS=Clavispora lusitaniae (strain ATCC 42720) OX=306902 GN=CLUG_00486 PE=3 SV=1

MSQSSRKKLLLMGRSGSGKSSMRSIIFSNYSAFDTRRLGATIDVEHSHLRFLGNMTLNLW

DCGGQDVFMDNYFTSQKDHIFKMVQVLIHVFDVESKSINKDIEIFVRALTNLQKFSPDAK

IFVLLHKMDLVQIDKREELFAIMMEKLQKISNPFQFKLVGFSTSIWDESLYKAWSSIVCS

LIPNMSLFNSNLLKFNQVLDAEEIILFEKSTFLVISSTASIQKQLQKQHASELAGSTESL

DPKRFEKISNIIKTYKQSISKLRTNFQNLVIRGSNGSHFYIDVLTDNLFIMIVLKDKSSQ

ESCQTTREDFVILDNIKAARRWFEKIESGK

>tr|C4XX19|C4XX19_CLAL4 Nuclear cap-binding protein subunit 2 OS=Clavispora lusitaniae (strain ATCC 42720) OX=306902 GN=CLUG_00492 PE=3 SV=1

MEILEETYTNSAERLDKPSQYLIRKAIRRGNFDNITKALQSKTVYVGNLTHFTTEEQIHE

LFSKCGTIDRIIMGLDRNKLTPCGFCFVIYKDEEGSLNAMKYLKGTYLDGQSLEIDLDPG

FREGRQFGRGIYGGQASQEQMDNGFPRRERGGFRGGFRGGFRGRGRGRGGPRGGFRGRGG

FRGGFRDSFTPDRV

>tr|C4XX30|C4XX30_CLAL4 Increased recombination centers protein 22 OS=Clavispora lusitaniae (strain ATCC 42720) OX=306902 GN=CLUG_00503 PE=3 SV=1

MLSCLLLHTNKNVATPNLSPFWKFTIQFSFLINHSKLMGLLMSARLDIASRDYNTSHWTR

QSAISTLAIYSETMKFSTLALGLLSFVGAVFSEEVEDFFGQASTENKLSNFKIDYIIEEY

PDVDPSDVAQLSAGEEIKLLYTITNEEDTDLTVVGLGGAFRDPISGDYKVNLTSNSVNPL

VITPGESAQVGQKIRLDIDSGSYYLTPQVYVAFQDELKAIQARGQLAIVSEVPVSFLDPQ

LLFLELLFVAISGGLGYFVYTTYFKTYFKRTAPTKKVPVAAKSTGFDPSWVPSHHQVPQK

RTKSRKAY

>tr|C4XX31|C4XX31_CLAL4 phosphoinositide 5-phosphatase OS=Clavispora lusitaniae (strain ATCC 42720) OX=306902 GN=CLUG_00504 PE=3 SV=1

MRLYLNEYPRTFVVADSQFSLIIRHPNPQYSSHEHSHRHAHIHLEGRKAKEARSKNKSGQ

NKVMVEFVRTEALTLKGFSDITPGRTREKVLTGFLGFLNVKGQIFLGFITRSTKVASPKL

GENIHMIDSVDFFCLNSDHYDGWINKEDEVHSSASSEEVESVQTGYPAGSVKRFLSSGNF

YFSKNFDVTTTMQERGSPTSSSHAFNSSDSYFSRFMWNSYMGSELLEFRSTLSLQERESF

DSAGFLITIMRGYAKSVNLKFSDRENALVTLISKQSCKKKGPLFGEWGCDEAGAVSNFVE

SEVIIYTESFCLSYVILRGNVPSFWELQSNFSKKSLLSSKKSKKVVFTRSSEASQHAFIR

HFNDLGKTYGDIHVVNCLSQDPHTYKGELNKNFKSLLDAAIKSKDECNGDLPDAAVMQQT

PTNNYKLSYTDMPISTSYMKKVGYSTINPADVVGPLNNAVIDFGAMFYDLKRYSYIGRQL

GVMRVNSFDCLSKANFVSKAICQEVIELALRDMNIKAPHDLYIQHAKLWSENDDVLKHLT

LNYQANMTKIQNSKSSAAKHSLKQQLTWKYMNVVGEVKQSDVAMLKLLGRLEDQDGVVLY

NPFHQYVSLELKKRSSEFSYSKEIKIFASTFNVNGDVSHDDNLREWIFPSKHDVDKDYDI

IFVGFEEIIELTAGNMMNVKSDNFIAWEKEIKKILEESKYTKEKYVSLWSWQMGGIAVLL

FIKESHVSNISDIEGSVKKTGLGGMSANKGGIGISLTYAKTSLCFVCSHLAAGFSNIDER

HQDYKTIAKGILFSKRKKIKDHEGVIWLGDFNYRIDLQNEHVKNLIKAGSFQKLFEYDQL

NRQMASGESFPFFNEMEITFPPTYKFDNNTKVYDTSEKQRIPAWTDRILSMSKGETLKQE

VYDCEEDVIFSDHRPVYAIFKASVAMINETAKKEITHDIYESYKKDVGDINVLITSSDIS

QFVDDKNSDDMPPPSSDAKKWWLEKGAAAKVSIPELENAPPEGENAMVINPRFPINPFVP

TDEPEFIRKADLLKLVESKS

>tr|C4XX33|C4XX33_CLAL4 2-isopropylmalate synthase OS=Clavispora lusitaniae (strain ATCC 42720) OX=306902 GN=CLUG_00506 PE=3 SV=1

MPMLADPSIKYDAFPPVNLPNRQWPSKTMKKAPRWLSTDLRDGNQSLPDPMSFDEKKAYY

HKLVDIGYKEIEVSFPSASQVDYDFTRYAIEHAPDDVWIQVLSPCREELIKRTVESLKGA

NKATVHIYLATSDCFRNVVFGLTKEQSKNLAVKCAALVRSLTKDDPSQSGTQWAFEFSPE

TFSDTDLDYALEVCEAVKEAWQPSEENPIIFNLPATVEMATPNVYADQIEYFSTHISERE

KVCISLHPHNDRGCAVAAAELGQLAGADRIEGCLFGNGERTGNVDLVTLGLNLYSQGISP

KIDFSNLGEIIDIVERCNKIPVAARAPYGGSLVVCAFSGSHQDAIKKGFNQYNKQLKESN

GKSVKWKLPYLPIDPADIGRTYEAIIRINSQSGKGGSAWVILRNLELDLPKGLQVDFSKS

VQKQAEIKGQELTNEELCDLFKNEYFINYETDGHNHHYKLRDYSLSKAVDGARTIEVEIE

VGGQVKKITGSGNGPISAFSNAISKDLGIYNDVLHYHEHSLGADSNSRAATYIEAKLEDG

STRWGVGIHEDVSQASFLSLVSILNSLHARQNK

>tr|C4XX34|C4XX34_CLAL4 Major facilitator superfamily (MFS) profile domain-containing protein OS=Clavispora lusitaniae (strain ATCC 42720) OX=306902 GN=CLUG_00507 PE=3 SV=1

MSSNSTDFEKEIANANEVTVSDPFHYDEEERKHRLSKRKRAADIFTIVCSGFALISDGYQ

NNVMTMLNTLFATLYPKEYGTNMKTNVSNASLVGTIFGQVAIGILSDRMNRKQAVVIATL

FLVFGTIMCAASHGKSVNGMFWMLIIFRGVTGFGIGAEYPSCSVSANEAANETVRRRGGV

FCLVTNLPLSFGGPFALIIFLIVYQITGTKDSLWRTMFAIGAFWPLSVFYFRYKMATSEL

FKKSAIRKNVPYFLAVKYYWRRLLGTCLCWFLYDFVTFPNGIFSGTIISSVLKGKDKKNM

EKIAEWNLLLGIIAIPGVIVGAYLCDKIGRRNTLALGFSGYIVFGLIIGCSFDKIKDITG

LFIVFYGLMMSMGNLGPGDMMGLTSSECFATPVRGTFYGFSAAIGKVGAVVGTKSFTPIQ

NNIGKKWTFIIAAICGLLGVATALLCIPQLTDDDLMKEDINFKNYLVQHGWTGHFGFEEE

LDSVNTNSDEDVEKENIVAEPSKK

>tr|C4XX38|C4XX38_CLAL4 DNA topoisomerase (ATP-hydrolyzing) OS=Clavispora lusitaniae (strain ATCC 42720) OX=306902 GN=CLUG_00511 PE=3 SV=1

MANVADSQEILLLMKSPKRHRGKTSSYVCHREMAETEHQSMKFAATVRVMNILIDQLQNR

QVSTMRDIYYKDVTLFKGRQAHSNNVLDAIANSLNVSLQSDLGVYPTPKGVIWSNNPIEI

TVGEQKKMSFDPCNEPLLICHRETSENLSVVGDVDMVVVFEKEAILKSFMCFVKKIATGV

KLILATGKGFPDRATLSFLGGLQACLPSTPMVYFVDSDVYGLQIFWSYMQMSELLAQNAK

LGGAFICDDRTEWLTMDKREWIRSVNFLQRNARTECCPSVSVPVEQHLQLMGRELRRSLL

LFKKAEINVIKDDEASFQSVNQYLWSKITLYVPGTGDLT

>tr|C4XX39|C4XX39_CLAL4 Inorganic pyrophosphatase OS=Clavispora lusitaniae (strain ATCC 42720) OX=306902 GN=CLUG_00512 PE=3 SV=1

MSYTARQVGAPNTLDYKVFIEKDGKPVSPFHDIPLYANKEKTILNMIVEVPRWTNAKMEI

SKEQKLNPIIQDTKKGKLRFVRNCFPHHGYIHNYGAFPQTWEDPNQVHPETKAKGDNDPL

DVCEIGEQVATVGQIKQVKVLGVMALLDEGETDWKVIVIDVNDPLAPKLNDIEDVETHLP

GLLRATNEWFRIYKIPDGKPENQFAFSGECKNKKYADEVIAECAEAWDKLIKGVSDAGEI

DLTNTTLASTSTYAESVDVPAATNEPAAPIDKSIDKWFFISGAH

>tr|C4XX42|C4XX42_CLAL4 Repressor of RNA polymerase III transcription MAF1 OS=Clavispora lusitaniae (strain ATCC 42720) OX=306902 GN=CLUG_00515 PE=3 SV=1

MQGSNLSSSLLDDSIEDTSAIDESPFGPLKNVSTRKTFAYLIAILNTTYPDHDFSNLQPT

TENFDRVESPELLMDRFNSIMISLGKKEDLLSWVWDTINVYMDLFPSRSPKLSAQAVNSS

RKNSFTSGTMSTSPKTDPSPNLDPGCKIYEFSPSDQSILEDLNHPYQTSWSYYWFIYNKK

KKRVCFLYLTGLNRLHYSQVNENPDRAYDNDEIDRTSKNDDSDDFMEEDLEQDDDEDDDN

DDVMGDLEI

>tr|C4XX49|C4XX49_CLAL4 GPI ethanolamine phosphate transferase 2 OS=Clavispora lusitaniae (strain ATCC 42720) OX=306902 GN=CLUG_00522 PE=3 SV=1

MMTTWWSLLASCLLQILGFCIFLKGFFPSKVVLDGFSEFSDSYSPFNVDGKAQFDKVIFM

VVDAMRSDFMFSENSNMKYFHSLVSEGNAIPFTAYSNPPTVTLPRLKGITTGGAPSFVDA

ILNVADDKDDSQGLSKTDSWISQFKRKGSNLHFYGDDTWLKLFPPSEFFDKYEGTNSFFV

SDFTEVDFNVTRHLDSELNDSTWDALILHYLGLDHIGHKGGPESVYMKPKQMEMDDVLKR

IYESVLAKSENTLMVLLGDHGMNEIGNHGGSSPGETHPGMAFISSKFSQSLSKKPFPVSG

TENYEYYSVISQIDLVPTLASLLDFPIPKNNLGIIIPEFLDLWGNEVQQNRILLENCNQL

MKLLTSKFSPEAEELQNFEVLLSRLQETTKSGKEEYYSFLTRAQELLAESATNYSYYDIW

FGVILIMFSFILSSLQLINFFECDQRVYCGANVVFVLSTLVYSFHFHGSSLIEEEYQIWW

FLTITFLSFFQISVKPKKFSLFVKCLLGVRVIRAWSNTGQKFSTPLTVSTTLCNFPNVLW

SLNCLTYVTISWLVAFEGGWQNLFSRDKSKLPTKALTKLVAWVIALSISIPSFIFKSCQY

YIDGKDTPVWMKFSIEIFTSLLEIEADDKKEIQRVNISLSKTFFAIFVAFFALRILINQW

HNNIRSTLITDLVNMITFLLIHQSKAEVIPLFLVFLSVRHSFRGLIKESTSLNINEKVLS

ITAFMLCMQNLSFFSIGNTNSLATVDLSNAYNGIAEYDVFLVAFMTFISNYAVVIFWSFT

ALELIFVTVGELAPTNFKTQFRNAILGRSILTTFFYGLSMMSLVGSCINLRFHLFIWSVF

SPKLLYFASWSILVNIGIDIIIASLFSI

>tr|C4XX51|C4XX51_CLAL4 chitin synthase OS=Clavispora lusitaniae (strain ATCC 42720) OX=306902 GN=CLUG_00524 PE=4 SV=1

MVNPFEENDPFFDASEPSLPNAAFTSDRTSPRRLYQIDREQEENDNSSFGGIPESVRSSP

NHRQSPNIFTRSSTHAKQFAANILQTPTPSLNFETHKTPRARYTLDESPKRERQGGIIIS

EEASLPNEYDGSPHRLEFRRSLQSSNRSERLLEPPQPIFSRETFVEANEAGMKDQADEQT

LHSHGDSEYGYDAYQKEYEDVETFSDRNTVYTSDSYVTGVESDYFGNSLDKDMMRNVSEN

YVPNREKTLTKRKVRLVGGNSGNLVLENRIPDELKKVLTRTESPFGEFTNMTYTACTAEP

DDFVSEGFTLRAAKYERETEIVICVTMYNEDERSFARTMHGIMKNVAHLCSRSKSSVWGK

DSWKKVQVIIVADGRNKVSESVLQLLTATGCYQENLARPYVNNKKVTAHLFEYTTQISID

ENLEFKGDEKSLTPVQVLFCLKEKNQKKINSHRWLFNAFCPVLDPNVIILLDVGTKPDNH

AIYNLWKAFDRDSNVAGAAGEITAMKGKGWINLTNPLVATQNFEYKMSNILDKPLESVFG

YISVLPGALSAYRYIALKNHDDGTGPLASYFKGEDLLTSHHEKNSKTNFFEANMYLAEDR

ILCWELVAKRNESWVLKFVKSATGETDVPETVPEFLSQRRRWINGAFFAALYSLRHSNRI

WLTDHSFARKFWFCVEFTYQFISLLMSFFSLSNFYLTFYFLTGSLIHNKGLGRGGFWIFT

IFNYLCICVLTSIFISSIGNRPQASKNIFKSLMILLSVCSLYALIVGLYFVIHTIQLYGM

GGSSSYVLASIVVSLLATYGLYTLMSILYLDPWHMFTCSLQYFMMIPTYTCTLQIFAFCN

THDVSWGTKGENNSKEDRTKYVIQQNEAGEFEAVVVDYNIDEVYLETLYNIRAKRSNNKV

KRNSEKKEIVDGEDYAKDVRTKVVLVWLLSNLIFILIMLQVYDAGQTKKNYYLAFILWLV

AVLAFFRAIGSMGYLLQNLARRLVEANSKWKHKREGYAYTNTNSLN

>tr|C4XX54|C4XX54_CLAL4 Amino-acid acetyltransferase, mitochondrial OS=Clavispora lusitaniae (strain ATCC 42720) OX=306902 GN=CLUG_00527 PE=3 SV=1

MSIYSCFRKVTQVYFTIMSNFKTLNRQFISNFKTTKYVTDAKRNLILSILKSTTTKREAK

NYLYKYQNQFDFSDISFENTTVAPKLEKRDHQRELFINRFLRGQDPFLNVYDEEETKLRK

IPLRVALFKICVMDGSIELWEGITETFRRLVHLGISPIIVLDNASVETTNFKARTSHAMH

QAYKLNQYLAKKAEEETSTIKSSIVRSPFIRKGEQLTISSLEQILIPLYQGIIPILQPIE

YTSATGVQKFVDANEALYTICLDLLNHSSLLTLEKVVIADKLGGIPSIERNQTSHVFINL

SQELSDIISEMYIGFLETEVRDQHLKNLHIMNDILELSAQKSGNNDTTGIITTPEVIALN

NDSLNPVIYNVLTDRPIISSSLPSSLERTPQVSTSILKKGFEVTVLDQFSYDNHFTFSNL

VRDGLVDEDKLFNLISDSFRRPLDKEKYRSRIQNSIASIIIVGDYDGAAIVTWEELADGQ

RVAYLDKFAISVANQGLPSLADIIFKLVSQSHQGELIWRSRANNPVNKWYFERCRGSLGL

QDSPWKLFYTGDIFKNRLGARLDSSKHDSIDITSKLRMYSDLIESIEPSFN

>tr|C4XX57|C4XX57_CLAL4 Phospho-2-dehydro-3-deoxyheptonate aldolase OS=Clavispora lusitaniae (strain ATCC 42720) OX=306902 GN=CLUG_00530 PE=3 SV=1

MSQTPMPEEYDDTRILGYDPLIPPALLQNEIRATRTSLDVVLKARVESSQILAGKDDRCL

VIVGPCSIHDTDAALEYANRLKKLAAELEQDLVIIMRAYLEKPRTTVGWKGLINDPNVDN

TFDINKGLRVSRQLYADLTGKVGIPIGSEMLDTISPQYFSDFLSFGAIGARTTESQLHRE

LASGLSFPIGFKNGTDGNIGVALDAVQASSKGHHFMGVTKNGLAAITTTKGNDNCFIILR

GGKNFTNYDPESVAKAKNDIAKSNAPHIKLMIDCSHDNSKKDYKNQPKVLDSVVEQISQG

EDSIIGVMIESHIHEGKQSMPSGNQGKSALKYGVSITDSCVSWETTVDMLKGLSKAVQAR

RAKKN

>tr|C4XX58|C4XX58_CLAL4 Protein BTN OS=Clavispora lusitaniae (strain ATCC 42720) OX=306902 GN=CLUG_00531 PE=3 SV=1

MSIWVIDRRRIFLAFFIFGLLNNVLYVVILSAAVDLVGPSTPKAVVLLSDIIPSLTVKVL

SPFVMHLIPYRQRIWTLVALSSSGMAIVSLTNQDATVSKVLGIVMASASSGLGEVSFLQL

THYYETTSALGGFSSGTGGAGLAGSFLFMVLTNVLGIQVWIALLLFAAAPLGFILAYYFM

LPAPVLDQSYSAIDDADMSEFGLDPVDQEEGFPTMTAYASRKELIIQHIKNTFGEIRPLI

RPFMLPLCTVYVAEYVINQGISPTMLFPLKDLPKWLFSSYRDIYVVYGFLYQLGVFVSRS

SINFGIRVSNLSILSVLQVLNVLITLCQSIFDLPFSNIWLVLMLIFYEGLLGGLSYVNTF

MSVSEQAPPEKREFSMGCVGISDSFGVMLAGCINLWLETKLCELQVNRGRSWCRTG

>tr|C4XX60|C4XX60_CLAL4 Mannosyl phosphorylinositol ceramide synthase OS=Clavispora lusitaniae (strain ATCC 42720) OX=306902 GN=CLUG_00533 PE=3 SV=1

MRKELKCILWAHFALVMFLVYLSFDLLCLIVDNPNADALLDVELNPVDGKLNRPLVIPKI

IHQTYKTEDIPEHWQAGQQACIDLHPDYQYILWTDEMARDLIAEHYPWFLDTWDSYPYPI

QRADAIRYFALVHYGGVYIDLDDGCKRRLDPLLTVPAFVRITDPTGISNDVLGSVPKHPF

FLKAVDNLKKYKRNWLVPYITIMFSTGPLFLSVMLIQFKRKGITEAWKVRILMREDYKNN

DLSFFTIAPGSSWHLDDAALIKSLANHIGMAVFGGFLLAGFVFLLEWWFYQWCIRTNFSK

KFKSLASRIPWKRRNEKRRLRKDSNLPVNIDFEKEENLV

>tr|C4XX62|C4XX62_CLAL4 Exosome complex component OS=Clavispora lusitaniae (strain ATCC 42720) OX=306902 GN=CLUG_00535 PE=3 SV=1

MTKAGELSTNERSYIFNALKKGFRLDGRALDEMRKPLITLSKTEYGYVEVELGDTKLAVR

VSCEIGKPFEDRPFEGTFTINTEISSMASPMFENGKNSDEEVLISRLIEKAVRRSNALDL

ESLCVIAGEKVWHIRADINFLNYDGGLIDASCIGVMTALQHFKKPDVSIQGTDVTIYSFD

ERQPVPLSILHVPICVTYSFFNPGDKEENIKGALNQEIAIMDANSKEEAIRDGSLVITMN

KNRELIQLSKNGGLPIDGVALLSLAQNSFAVAEKLTDQIKALLKEDEEERYKNMHLELLE

VGAAR

>tr|C4XX67|C4XX67_CLAL4 tRNA (guanine(9)-N1)-methyltransferase OS=Clavispora lusitaniae (strain ATCC 42720) OX=306902 GN=CLUG_00540 PE=4 SV=1

MFFAWTGHREISLTTMSETGEMGNDLKRQKTEPADGLGFQKPQVPEGMSKNAWKKLQKQK

KWDEEKAEYRLKRKEKKKAARARKAERKSQGLSDDDNYHQQAKKAIPEKQKPSGIKVIMD

CEFDELMSDKEIVSMSNQITRCYSAKRHSDYDVDLVVSSFNKRLKQRFDKSVSDYPRWKN

ITFVENDKLTDLLPTDAEELSKCVYLTADTDTELEELEQGHTYIIGGIVDKNRHKKLCLN

KAKELGLKVGRLPIGKYIQMNGRQVLATSHVYEIMCMWFEQGNDWQKAFNAVLPPRKIKG

EAEAKDETETKDGEEPKDEEETADDGKENGNEHESEDKENRDEENNVDGE

>tr|C4XX69|C4XX69_CLAL4 Zn(2)-C6 fungal-type domain-containing protein OS=Clavispora lusitaniae (strain ATCC 42720) OX=306902 GN=CLUG_00542 PE=4 SV=1

MARGCVCRVPQLSMTHVWHGDVAKGICPNAVYLRIATRNAEHITLSFFLRASTPQHSETS

LLKMMEVQTQNDKPRVSAAAQESAGQKRKRSRAVIVCTYCRRRKVKCDKQSPCSNCVKVG

IADTCTYDSHTDKKGKDKYEMELALPLGQQPLDSVAKEANIGVSGQFAVASGAGTAKSAQ

NGPKRIRSEKGLTASAPSSAYSAASSHEQSAGNLAYGTTNRAPSGPIMGPSNGAPHVSGV

SPAGSVPNYGEPNASSTASMSSASGATTNFTSPSTYSGGGQTPGYAGFERPANSTIKTMP

RSRVDLKQADTQTIETQKSEIEVLKQRLQQIEKTLMGSPKSLPALSAQPNAFPPMPMQPS

APPSSQLQYQGPPQPSSMQPLGVQPAPFQLGQRPSYYPGMTPGTIPMPSSSAFTPLSQRN

SFSEDPMLAMLPPLNSNLKRSGGSQSPLQANSVGPFPQSVKPAASESSSATGCESACNAT

STACNTSPATTETPPKLTIENPKAEDFLIGVNIYSNASDTINFYENYSSLHFKDNLRRSN

FGPFAWTSLMKRDYGLRLIWDHIIAQKQSTKDDSSALAFPQQTNEITAENTNTILHADKS

ELSEKQFRKRALQADGYEDIVPYNTILEAKKERDIQKQTLNQSTLPLGLTFYDGQIDREL

QLIEKVRVVLPKKRVIWKLIARFFKCVYSYFPFLDEEYFRRDVSRIIGPESYEDESVPDI

KIERKLDLATVGILLIVLRLAYLSVFSNNTELNEQILRSEAPTLEVQSIKCLMLNPININ

IIDVAELCFEQFKLLRRSNFTVLQLALYIRLYHTYAPEDGDGADGGDSQVLNAVLIQMAY

SLGLNREPDEQCTDLKINNLSRKIWSYLTLADLHLAYAFGNPMSIDTMYADVKPPLYIPG

GENLLDKEADRLIVDRFNTCTFWYPDIRRILKLTLNVNGRVPLPELCSLLSNAELDWFQK

CGTLSEALKCGGLGVKSTVERNYTVKIYLALRVSFLAIFFHIFLHYERKNDHVSFFYLKK

CLLITTADIMPHYETLLCKSEVVSDMIINPTLEMAVHKANIIYLAAIIRVNFAVYHLRQS

SEHDQRCKNDKQYLTYFQKLCQLSSCLTRASEYSISAISKISNRYYYAWRITKGQTFMLK

TVTSTQFYESNYHAAYSLYSTRFSCQQIDELICICETTLSKFRHTEFRTYGFSREVNDQL

VKCQQYSCDPVRNASNSSESTSTSGFSASTDSISTDDPTNRVTNTEVDKLWLQLLSMKHD

QLFNEDYREAPEVMVTNGNGTTNKPVSQNHEANAGAATTNDFARFGYDMEMENRYDCFSD

LPFDQVFNF

>tr|C4XX78|C4XX78_CLAL4 Golgi apparatus membrane protein TVP18 OS=Clavispora lusitaniae (strain ATCC 42720) OX=306902 GN=CLUG_00551 PE=3 SV=1

MAFSIQTIFSNLFNGFSQDFRKKNFSLYGQWIGLFTIFLCLALGIANIFHFNLVVIFAII

CIVQGLVVLFVEVPFLLRICPLTDTFTRFIHNFDDNWPRCGFYLLMSAIQWLSLTIQATS

LIAVALFFLFSSLCYLFAALKHQEYIKSSFNVAGDGNDLESQAASHIVRNVL

>tr|C4XX80|C4XX80_CLAL4 Phosphatidylinositol transfer protein SFH5 OS=Clavispora lusitaniae (strain ATCC 42720) OX=306902 GN=CLUG_00553 PE=3 SV=1

MSLVASHKLDEDQTSKLQQLIDSIEEIVGATENREYDEIYGYRMAPDGQEHVETAARNEI

LLKFLIASEYDVAVAKKKLTATLNWRRKFKVLSAAYRETYDPELEKLGVITDYKNNKDNF

RVVTWNLYANLKSPKKLFAQFGVDGDKNEEELEGTMFLRWRVGLMERALSLLDFSNAHNN

KIAQVHDYNNVSMFRMDPGMKAATKQIIHVFGENYPELLSKKYFINVPLLMGWVFTFFKA

TGFMSAATLKKFEMLSSGDLSSAFGKDNLPKEYNGGQENPQVPSIFTSAVTKTEVPAYGA

IVMEKFFKDRTIEKEINTVD

>tr|C4XX82|C4XX82_CLAL4 GPI ethanolamine phosphate transferase 1 OS=Clavispora lusitaniae (strain ATCC 42720) OX=306902 GN=CLUG_00555 PE=3 SV=1

MPCIATFAHNHQTTNRLSFFPMFHRHLFVALGVAFHFFYLWSIFDIYFVSPLVHGMAQHK

ATQDAPAKRLFLIVGDGLRADKTFGRVHHPESGEDVYLAPYLRSLVENNATWGISNTRMP

TESRPGHVAMIAGFYEDVSAVTKGWKENPVDFDSFFNQSTHTYSFGSPDILPMFAYGDSV

VPGRIDVCMYGHEFEDFTQSSIELDAFVFNHVDSLLEQAQTNATLDAELRRDGNVFFLHL

LGPDTAGHAYRPYSAEYYDNIRYIDEKLADLVPRLEAFFGDESSAFVFTADHGMSDFGSH

GDGHPDNTRTPLIAWGAGVRKPELASPEQLAKQHPQVAGYEDEYFATWGLDQYARHDVKQ

ADIASLMAYLIGANYPANSVGELPLAYIEGAAEDKIAALYENALAIVEQYIVKESEVYGH

QFRYKPYDPFVERPVAEQKRQIEHLLAEFRHDPTPEREEELVALVESLMKCALDGLTYLQ

TYNWVLLRSIVTLGFFGWIGYSLNVFLKLFILKSFTQPPHSTPLLALFGVLATALNYLLF

YQRSPFNYYMYAAFPLYFWYTILNERSLFAQGLRVFLFGISTPTKAFTLVSFLGMYESIV

YGFFNRHMFSLLFVLTGLYPWMVRKPLPLQTKLTWLVSCICMCTFTNLDVVKVESLAQIN

VSSAIAFIVGTIGAGKVFQRNIPAVVKKLVVAQLATIPVMVFATNVSVVSLQARTGLPLY

SQVLGWVTFVVSLVVLPVFHALHACSDYQLRLLIIFLTFIPTFVILTISFELFFYVSYSL

VLLQWLQIETSLKFTRKEIEESKESSKSLPQGYWLQTIRVSIIGFLFLQFAFFGIGNVAS

ISSFSLDSVYRLVPIFDPFAMGALLMLKLIIPYVLLSTCFGIMNSQLEIRKFTISTLIIS

TSDFLSLNFFYLVRTEGSWLDIGVSISNYVLAILSSLFMLILELVSTVILRGVKFEDQDT

KISEILADENPISSRLRRRKHN

>tr|C4XX83|C4XX83_CLAL4 Multifunctional tryptophan biosynthesis protein OS=Clavispora lusitaniae (strain ATCC 42720) OX=306902 GN=CLUG_00556 PE=4 SV=1

MTQKMGVLKSQSVIKFAPYNFSMSQTPKHVLMIDNYDSFTWNLYQYLCQSELCAKVDVYR

NDEIDIETIENVIKPDLLFISPGPGHPSTDSGLSKRAIEYFKGKIPIFGVCMGQQCMVEV

FGGEVTFAGEIVHGKTSTVKHDGKGAFAGIPQGVAATRYHSLAGTKGKIPDCLEVTATTE

SDPEVVMGVRHKKYTIEGVQFHPESILTEAGHELIENVLRMNGGTWEESQASKPKGNILE

TIYKQRIEDYAAIEAQPGKSFADLQVSLDLGVAPPLVNFYQRLASTKTRGETIILSEFKR

ASPSKGDINISAHPGKQALTYARNGCSAISVLTEPKWFKGSVDDMSLIRQVIANEKDRPA

VLRKEFIFSKYQILEARLAGADSVLLIVKMLKDAQLLQTLYEYSLSLGMVPLVEVNDSDE

LKVALALTNNNSKADSLVIGVNNRNLTTFDVDLNTTSSLVSEAKNSGREGDVLVLALSGI

TTSQDVKKYKYEDNVDGFLIGESLMRAEEKGEAGTFLHELIHI

>tr|C4XX85|C4XX85_CLAL4 Replication factor A protein 3 OS=Clavispora lusitaniae (strain ATCC 42720) OX=306902 GN=CLUG_00558 PE=3 SV=1

MDAASIRVNASTIKDFAGRVVRVVGRVDSFDAVSDSARLDAGGAIDVSIHSNDKLEVGKV

YEIIGKVGVSDYKVNVYSVLPLSDGVNVDVANQLAKFVQKVPELFY

>tr|C4XX91|C4XX91_CLAL4 Mitochondrial phosphate carrier protein OS=Clavispora lusitaniae (strain ATCC 42720) OX=306902 GN=CLUG_00564 PE=3 SV=1

MLCWINSSQIGDVPRSYRRKRKESTRKIFPKACTHTQARRRTCCHRFASCATALQFGSSK

AVGLAWAWSGGVRPDTAGARPETYGCARKFARCAWKQANKNRSCDSARAFSFFAYISPGA

KGIFSFSSLFHTCVYIFIMAAKQQTLPTFTVGDYASFALAGAMGCGVTHGAMTPIDVVKT

RIQLEPTVYNKGMIGSFKQVVSSEGAGALLTGLGPTVLGYSLQGAFKFGGYELFKKTFIE

QLGYDTASKYKNSVYIGSAALAEFFADIALCPLEATRIRLVSQPTFANGLIGGFSRILKE

EGVGSFYNGFTPILFKQIPYNIAKFLVFERAAEAIYGAIPTPKAELSSGAATAVNLGAGI

IAGCAAAIVSQPADTLLSKVNKTKKAPGQSTIGLLAQLAKQLGIRGSFTGLPTRLVMVGT

LTSLQFTIYGSLKSALNCPKAVEL

>tr|C4XXA4|C4XXA4_CLAL4 non-specific serine/threonine protein kinase OS=Clavispora lusitaniae (strain ATCC 42720) OX=306902 GN=CLUG_00577 PE=3 SV=1

MSSIYTSDLKNHRRAPPPPGARPEASSRFNSAPRKHSGWVYVKDDGIFTSFRWNKRYMAF

GDRSLNLYKQEPEDPARDMDVSYPLSLVSAINLKQNSGPSKGASTLELVFKSGSKPLLVA

VKSSTEYLDWLDTFSTKCPLAQIGSGGTVAVGNTGVSSPINFTHKVHVGFDPASGSFTGL

PDAWKSMLQHSKITNEDWKKDPVAVIEVLEFYSDISGSAQNTPVASPALYQGPPQMSSIH

DAAKHAPSKTDEKSAGVRLAPKPPLPYHMTQKQKESASPLGNLLQQNNDLVPVRRAPPPP

RTDASRPSQNAHAHAVQQSSSQSSQSSSQSSLSSQSSHSQQKPSLSQQHPQRHQSPPSAS

LQRKPQGAAYPPPRTSPPALPNVHPNVKISPVKEKTPPSRVSPDLKPLKLKASNNAQTNV

SSKAPAKLATGPAQTKTAKQIKKEREALNNRQVLAKLKTVVNNNDPSNLFRIISKAGQGA

SGEVYLAESLIPGQKGKKVAIKQMDLQAQPSKELIINEILVMKDSQHDNIVNFLDSYLRG

DNDLLVVMEYMEGGSLTEVIENNECKLNERQISTICHETLKGLQFLHKKHIIHRDIKSDN

VLLDAKGNVKITDFGFCAKLTDQRSKRATMVGTPYWMAPEVVKQKEYDEKVDVWSLGIMT

IEMIEGEPPYLNEEPLKALYLIATNGTPKLKKPELLSNSMKKFLSICLCVDVRYRASTDE

LLEHPFIQYKSTKIEELAPLLEWKKHAHDEH

>tr|C4XXA8|C4XXA8_CLAL4 Transcription initiation factor IIE subunit beta OS=Clavispora lusitaniae (strain ATCC 42720) OX=306902 GN=CLUG_00581 PE=3 SV=1

MHITSFQYAHTRALYIRTAKNRFPPPQLFFMSDLSSQLSAFKNRIRSGASVAVKRAVTPK

PVKTPEPPKRSGDSDLAQAIKRQRQNDSALSGAHLSTRLHLAVEYIKSQDAPVPISKLQG

YLSFDISDTLLPLLKEIDRIKYDPANNTLEYMSLHNIGNAEDLLNFLRSQPTFKGTSVKE

LRDGWSGCLDAIAELEEENKILVLRNKKENAPRLVWANLGGEIGTVDEEFVEMWSKIRVP

DRDSLYQALIDNSLKPTGADPHELKKKPQQQERKQRKARRGKITNTHMKGILKDYSQLV

>sp|C4XXB1|AIM21_CLAL4 Altered inheritance of mitochondria protein 21 OS=Clavispora lusitaniae (strain ATCC 42720) OX=306902 GN=AIM21 PE=3 SV=1

MDIPRIPQRPKRSKSSEENTPSSTPSSTPAPIVPARPRKNNGAAASSEGSGEFPPIPRRP

QRKKNIGETDSEADEKPKDAESDEGWNDSEGKTPEKDIHAALKENADSDKPDIGDSEAEK

QPEVDGKDPALNLEKALEANSEEAAIPSEDVTEDVTKSEETKSEDIEASVHIEEKVERES

EVADTKGDIEVDDGIEVDDAKEVPISELSETDSAFKSEPTDDAANPVSEEAVKPTEPSED

AEDELVLDRESKTARESEPLEAEDVEEQHDQAMEKASSVTQIGETPSDAKISEDTSESNK

GDEDVKENVLLEESGSEPKDSETGSPFQVEESAIVPSEGKPPAEVAETSQTTNKSAESLH

SEENLTQAKPATVSDQQPTEKQEPSEQEIPKQESSKQPSKPIVPTRPNKSVPSVPKRPVR

SVPASSSPESVQESDAKKAPPPKPKKLSSKIAAFQQMFNQPEAVPTAPQHPKSGKLSSEK

IGFAANLQNVMGRGIALPGMANPQMFQRASTDLEEETADVHAEPEQNPQVSKAPQRARGP

RGKRLPKAIKETTVKVEPRFQLRVASLWQVEFNKPKETEKVDDDLEEDYEVEPEFVDSVR

EPESDKETVAEPETSTKDDSVQTESLSNEESTSELPEAITTEHVLDSP

>tr|C4XXC4|C4XXC4_CLAL4 Patatin-like phospholipase domain-containing protein OS=Clavispora lusitaniae (strain ATCC 42720) OX=306902 GN=CLUG_00597 PE=3 SV=1

MDSRTDRADIPADIIEHLFTQHPQLRPAAAVPRPGLCARAQALARALPILGRLAPAPDSK

QALIDQLLAQQQNATTYRQWYDASAQLDELSGNNLWKATPQCRHYDHRLVQKNLEEMRSA

RLRKDHKLLLYLIRTKWTRNVGNMGALGLYRHSHVGTKRLVEEYIEECRQCLHYLVHDPS

VHLNDRYLLGMLIQTRKNIGRTALVLSGGSTFSLSHVGVLVALLENSLVPRIISGSSSGS

IIASILCCHTNDEIGELLMGITERRFTIFGTESEKDGRLKVFLQRIGHLLKFGTFFDISG

LRQTMYDFVGNITFREAYNRTGKILNVTVSPATKHEQTRLLNYLTAPNCLVWSAICASCS

LPGIFPSNSIYEKNPSTNKIHEWNNDESSKYVDGSVDGDLPILRLSEMFNVDHIIAVQVN

PHVSPILKVSVSSVGGKADSELSETVKGLLNNAYDFITSEIIHALQILHEMNIYQNLTLK

LISLLSQSYSGDITILPQLELRDFLKVFQNPTPQFMLDFILKGAKAAWPKITVINNHCGV

EFALDKEISFVRGRLIAEANSRIAQGFTDFPKAFDSNDYLVSSPVLAEEANAVSPAKQRA

KAKIRRHNSVTSGEARWQRHSPVVHRKRNSVSFVDRNLSFRGKSTTSLNSMGGKERESER

ESPFFKTPQAKYISNGGDGDSQSTRGTSFDFRSPHHEKYLDTDYVPASPKSEPREPRDKA

NLPRVSRAGSLRNSYVGLNRIKNSGEGDSPLSSPKSLEKAYLELEKNFKNLQPATLRQHF

KKPKEEKRLSFYLEKGGDMNGKQKSDEQKNDEQNNGQKKWTESCIRCGSRNQEKWNNNK

>tr|C4XXC5|C4XXC5_CLAL4 ribonuclease Z OS=Clavispora lusitaniae (strain ATCC 42720) OX=306902 GN=CLUG_00598 PE=3 SV=1

MFSVSTICHKTSDCQHPMVMLTSREGSRYFFGKVPEGAQRVLNENGVKLGKLKSIFLTGT

VQTWSDIGGLPGLFLTISDATSRGIDVFTNSSSVMAYVVATWRYFVFRKGIELNVLDTQS

ENLIGDSTTVFRPVKIPSRSSSPMGASAKILSQLKKLTSLMFPPDSAANSRDPASHKSDP

SENEIQTHVRLPEPSELVDVGSQPALSFVIRFLPVRGKFDPVKAKALGVEPGINYRKLTM

GDSVLNSKNELVHSHQVLAEPKSFRKLVIIDIPNANYLENTLRSDEWFLQSEEAGQELPG

LVYHFLGDDIDFRSADYISFISQFPQDCQHVISHSSIADDTLVFRTAAVHLLKLKCVLNN

SFSLPYIEKHSPLDLSPNTHKLSSLQYFTIDPSGVSLDEQNIISETWESLYDAEIPSSEV

LAGTDKSTILQNGILPLSPIPNASSLKDHVQVVTLGTGSALPSIHRNVLSNLVRIPYRDE

ETQEIRFRAILLDGGENTIGTLMRNFGHDSSKQLKQIFSELRLIYLSHLHADHHLGIISV

ISAWAEANKNNTDKLYLVIPWQYNNFITEWYRLEQYTSNIDMSRIVYLSCEDFMRTPEAQ

LKQFTLDEFEEKYDSNRLTDRIPKEDSALPKTSRIDMLYRDLNLANIRTVRAIHCYWSYS

VSLCFSLSSSETFKVSFSGDTRPSTRFIESGSDSDLLIHEASLDNDLIEEAIAKKHSTVV

EAVRVAQLMGCPKVILTHFSARFSEKHSFIRDAEEYDRLCENLKAYIGRSTTNVFTMNES

KLGFDDIDICYASDFMTIRYNDLACQKPFYAKLSELSTSATSEAEVAKSQKEQLKKSEKR

EAKRLQRLSKKKRRLSNESV

>tr|C4XXC6|C4XXC6_CLAL4 Telomere replication protein EST3 OS=Clavispora lusitaniae (strain ATCC 42720) OX=306902 GN=CLUG_00599 PE=3 SV=1

MYIRLFLGILKYSFSPAQCSIAMDYPIVLLNSWVLGLVTGCIRDQSIYSTNLLVKNFIKF

ESPTCTNLNVTPLLRVFAFTKSTDSGEISAVLHDSSHKILVLFTKECIERFESRYGQRIT

YHTVHSLLLVKQANLRFLTLFQLRSKFGVVGGLRISPKVALVYLEISDVDFFQRDQIWVS

PLAEKMLHYVYGDVEYEKKYGQQNVSPQGIADFVLENDDGLISDEESLPSLVKTCF

>tr|C4XXC8|C4XXC8_CLAL4 Large ribosomal subunit protein uL2 C-terminal domain-containing protein OS=Clavispora lusitaniae (strain ATCC 42720) OX=306902 GN=CLUG_00601 PE=3 SV=1

MFSLVRRWGTMLPALSRAQTVSTVSAAATLATSAADDMTDLEKKDAIYRRQRKLAMAQVK

VKSYANGHLQPGSTHFKKPIHDHLHKGPPLRELTVAKKQTAGRNNTGHITIRGRGGGHKR

RVRLVDSHRLTGGRQTVVRIEYDPNRSGHIALLKHNETGDLSYILASSGLRAGDVVESFR

PGLPADFLREMEENNNGEIDEALLNSRIMQRGNCLPLHMLPVGSIIHNIGLHPNGRGQLV

RAAGTFARLLSKHPAQGKVIVRLSSGEHRYVNMKCHATLGVVSNKEHQATSWGKAGRSRH

RGFRPQVRGVAMNAFDHPHGGGRGKSKSNKVSQSMWGLKKFAKTRKFKRVNRWKVQDRPR

R

>tr|C4XXD1|C4XXD1_CLAL4 thioredoxin-dependent peroxiredoxin OS=Clavispora lusitaniae (strain ATCC 42720) OX=306902 GN=CLUG_00604 PE=3 SV=1

MAQLRRSARVQASSTAQTAPPAKKQKTERKPVEAKPKTAEPQLDIGDAIPDITLANENGE

DINLAEEAKKSKFVVIFAYPRASTPGCTRQACGFQRNFEAFKKLGATVFGLSADQPKAQL

NFVSKQGLGVPVIAGPIERIDWSFRCPQIAFWCYQIPSGFC

>tr|C4XXD6|C4XXD6_CLAL4 IPT/TIG domain-containing protein OS=Clavispora lusitaniae (strain ATCC 42720) OX=306902 GN=CLUG_00609 PE=4 SV=1

MASKEDMVLDHSLLLATSHDDQDILNEFLDQRVFDSMNANSPNPSDHVKLEDFENFHSEL

FSRLKDEQTPIKAEAVSPAHSSPPLFRDATDELGMASAFTGPSSPQSAFSKSRNLQFDNY

DVVRKEMSRVKFGNCNTNPCPQSVDVADASYLDFSADALSKLPYTLEVLDLPTYSRVETQ

TKLQFRLSPPPPQNLLHIPQDLISKSKFCLQNPVDSLPNVLRQNMLYMDAYVLTSDLSRS

CNVCSRCIKREQKRASRSKPGPSDCSTGNSPTNGTSNMLKNNPNAWADDKMMKKAIILNC

KEIISLPEPSGLANDQFQAFDFSARLVCYCRHHKEQNGFKLLFVLKDHSGQVVAKSLSTP

IMIMDRKKNSAAGGKESLGSDSRMANLDLSENLEHDLDSGLHPLSPKSIDDSPTEGLANT

DTNTDSNTASRGLKRKKLSFDDSFNSSTNPMFNGGSGFSPLSNSDTNASIHNMNGKNSIV

GGVHNFGSSSSVAPQAVTQSLSIARQGSSQHLQHYQQPSLNPTIQKIIPAQGPIRGGIEV

TLLGFNFRPGLIVKFGTKNALATHCWSETTIVTYLPPASQPGQVLVSFENHDDIFSSGQQ

SIFTYTDDTDRQLIELALQIVGLKMNGKLEDAKNIAKRIVGSDGANGAAGHTPSPNGTPG

MNQTMNQANMDWYSNAHKAVEVLSRSDLSTEEILINFLTLVDLPNCPIIIPNWQLCNAEG

QSLLHLATLKNYSHLIKFLITHGCKIDIQDNQGLTPLFFASMCGNRHLIKAFVDCKSNWN

LKLSNDKFLKDYCDMNVLDIFHGLESDQALGGETSSYICSGSDVGEDGLNKSFSVDSLNS

MFMVDYSKHISRMVMDSSEVHDGEDSALGASQHSLTKGTSELPTPKSPTPFANDSDDFAD

SERDSEDNLSFCEDLDHNDADQEDEYTDDYDDDYSSSDYDTDTQERVLQEGSSSSLMRSE

PNLWQKVKNAVFSNDTEMDLPTYDDLFPFSNSRASAGHELDDDAAPGSKSFGVTEDAQED

VASDSSEDMVMSFINHPRKTVQNDKMLLFFWTPVLVMIICFFSFVSITGYKVEMVEQLKQ

VCRNTIGNLVVGHERLARVFSTEPMRHVVEL

>tr|C4XXD7|C4XXD7_CLAL4 uroporphyrinogen-III synthase OS=Clavispora lusitaniae (strain ATCC 42720) OX=306902 GN=CLUG_00610 PE=3 SV=1

MPCVAECGKRVLFLKNKTTPKDAYEDVFSSSGHKPVFVPLLNHAPIDIDQTAEYLASETF

LTETDSFIITSQRAVEVFHECLSVIGSRDPAKVEQIIAKTGYTVGPATEEILRAKGFSDV

RGGSLAGNGSKLADIILEEKRGCPKKIVFFTGVIRKDIIPVKLKQEGVDLEEVVIYKTEP

KAGILDNFVSCCEEKVDWLVFFSPQGTEEIVEHIQKGKISLDGVRIASIGPTTLEYLNGK

GIESHAMAPKPTAAALLEEIMK

>tr|C4XXD8|C4XXD8_CLAL4 Acetyl-coenzyme A synthetase OS=Clavispora lusitaniae (strain ATCC 42720) OX=306902 GN=CLUG_00611 PE=3 SV=1

MPEHLAMDHEGAQQPPQGFFDRSHSAPNLSSLDEYNAMYKQSIDDPATFFGNQARDLLSW

STPFNVARYPASPKDDFANGDIPAWFLGGQLNACYNAVDRWAFEDPNKPAIIYEGDEPGT

GRIISYGELLKDVCKLAQALVKAGVKKGDTVAVYLPMIPEAIVTLLAIVRIGAVHSVVFA

GFSSTSLRDRIVDADSRIVITADESKRGGKTIETKKIVDEALKDLPQVRNVFVFKRTGNK

HVPFYPGRDLWWHDELAKHGNYSPPVAVDSEDPLFLLYTSGSTGKPKGVQHNVAGYLLGA

LLTTKYTFDVHRDDIIFTAGDIGWITGHTYVVYGPLLNGATTVVFEGTPAYPDYSRYWQI

VDEYKVNQFYVAPTALRLLKRAGPSFIEKFKLDSLRVLGSVGEPIAGEVWHWYNEHIGRG

KAHIVDTYWQTESGSHLLTPLAGVTPTKPGSASLPFFGIDAHVLDPVTGKRLTEDGVEGV

LAIKNAWPSIARGIFNDYNRFIDTYLGNYKGYYFTGDGAARDKDGFYWILGRVDDVVNVS

GHRLSTAEIEAALIEHEMVGESAVVGYADDLTGQAVAAYVSLKSQFKSDDKDAIDAIKKE

LILTVRKEIGPFAAPKLILLVNDLPKTRSGKIMRRILRKVLAGEEDQLGDTSTLSDPSVV

QQIIDVVKETRK

>tr|C4XXE2|C4XXE2_CLAL4 Histone H2A OS=Clavispora lusitaniae (strain ATCC 42720) OX=306902 GN=CLUG_00615 PE=3 SV=1

MRAVFIQSKQTTRYYCFSSVTMSGKGKVHGGKGKSAESNKMTSSHSARAGLQFPVGRIKR

YLKRTAQNKIRIGSKSAIYLTAVLEYLTAEVLELAGNAAKDLKVKRITPRHLQLAIRGDE

ELDNLIKATIAYGGVLPHINKALLLKVERKKGQK

>tr|C4XXE7|C4XXE7_CLAL4 RNA polymerase I-specific transcription initiation factor OS=Clavispora lusitaniae (strain ATCC 42720) OX=306902 GN=CLUG_00620 PE=4 SV=1

MFEEPYRHYVRHQKNRKGNKLRYLVENLTHTRLLRQLESDLSVTLSPETKARLIHRIKEE

DAKKYLPEETYEQWYPRLPETAESQKKHKTEDFDLSLRQTLIHELRSAQTETTDAVEPAT

RGTEAEAGAEAEAEAEAADSSIETDIRTDYISDAMDTSESDESSDEEADLSDKTTDSAYD

DELDGDALYACKKARVWDHSFDSVQSWSRKQYTLRKWGLEPLGQNDGANKYSIRHQHTQS

INALLHINMMREKWDLAYKLFCVLVRLKQVDVRLVWPLGVEILLQRRKQLELTGSTSKLD

MLKAQSFLEWLMQIYPVFRFNTISHNSNMGPVFRSGSTTHAPMFVVSLLWQLLVERKYLK

LRETLDELLMTPPYSSEGLFYLISAYCSLAENIHLASLYSNFDQSPDFPAEDDQLGDLAD

DIMLIGSKETIKARILENHTRVRLLLESCDKYNFEYPKDIIKAEIEGIASILKGESDFVS

LVDSFKTPEAAKSEFALVNGRLIPHSPGSRAIPPQFLSRVICGKSKKAKKQSWVWFWCTL

SKDGKDAICDICGQILRRPSSSTISLSRHIKTHGITEESFSDKKISLLKKDVEALLPSQA

PPVAENGTPRVLDWSAVTRRKAKNARQKEGRFNNSQKTLSEESAAPVGLLPTEDNVSASD

DSDADSDHADLNMNMSGDRRTSVEEGNMYQNVEVGQEGRTNAISEQINDTITSVPRTKIS

PKSSPVPPGLKKSPISDKHLHSACLPATNVSTNVFESPNMGSEEQASPESTHGYDSGKAK

SKQSSFVAKYTEGFSMHESSESEEDDLQFFANSKSQFSHQKIFSPDSTFDSRFDEAEVSK

NFHEFATQTQRSQKKDNSDGSSEQEDVEAQDHLTSFGFESSRDVSENKDDKSSDLENNFE

DANDEMFPNPLEEANPVLSESEDYTDHFDSALESFPKSKQEEMEFDFDFD

>tr|C4XXE8|C4XXE8_CLAL4 RING-type E3 ubiquitin transferase OS=Clavispora lusitaniae (strain ATCC 42720) OX=306902 GN=CLUG_00621 PE=3 SV=1

MAKYSFLLILYGTFSTVLFGLSLFTSASRAYDYFTLIHELTGSFHLTLLVNFVVFCFILW

GLVSTKLLFGDLRIIETEHLVDQLPFYGLSLLFILFNDDNVLLSMFWGGTTILLKSYHII

NQDRLEHLQLMTVNNLHNINSPSLIFRTFASSIFMFYLLVSIVIDVFMAKLLVFDVFQGV

SSIGSLLYGIQFAVMATDSFAYLWKVGLNVYELMFYRCVPHPDASVRTPTQQSVPTDGAE

HSGSGDEDLFDDDNDDDETEQVWENKPLYTQTLEILSSVIKSVFYLIFSYMLYVHSNLPP

PISMIQGGIVSMLQVVQKTKRLAAFLSQAKSLDKQLEDATVEDLNAADYMCIICRDNMHS

PEAYEARRHKPLIPRRRPKKLRCGHILHMGCLKDWLERSSVCPLCRKNVFALEASTPPQP

QGTAQVPQETNPPNHGMHPTRTFPSGVPIPTSEATNVSVHGTNGNQSTNNMFTGRVPSGW

TAFPITRTGPDRFTIRVSPTQSGTLIIRENTGDAQLYTLGTN

>tr|C4XXF0|C4XXF0_CLAL4 Kinesin-like protein OS=Clavispora lusitaniae (strain ATCC 42720) OX=306902 GN=CLUG_00623 PE=3 SV=1

MYVLLLPDCMDQSKYTITTAISCTAHINNISSSIMSENIQVIVRCRARSKREVAANSPPV

IELPNDVFSAQEPYVGVSTDQPSLLSARSSFPGGKVFKVDQVYGPNADQQLLFENVALPL

FDDFVKGLNVTILAYGQTGSGKTYSMCGDLEGEHAGIIPRVLSRLFSVLNGDYMVKLSCV

ELYKEELRDLVNDELDLAPIKSKLRLVSDTSGPGPSSSTIIHNLSQIHIDSSEMGFNILK

KCLTKRRTGSTRVNDLSSRSHAIFTINLYREVKNAAGSSEYRISKMNLVDLAGSEDINKS

GAINERAREAGSINQSLLTLGKVINSLSEGKEQKHIPYRESKLTRLLQGSIGGKTKTALI

ATISPAKINVHETISTLNYASKAKNIRNLPQSSFDSEMILKKVLVSDLSGPNLSHDQRFD

GKQRQGEWYQDECPELRGTQ

>tr|C4XXF7|C4XXF7_CLAL4 Serine/threonine-protein phosphatase OS=Clavispora lusitaniae (strain ATCC 42720) OX=306902 GN=CLUG_00630 PE=3 SV=1

MGNSPSKSKNGLSLSRTDTGSSAKSGRSVRSRLSTSNHQDSPKVSRKNSSASLPTSFRSN

SSAFDENGLHASMAAPEPDKPILLRRNTNDTEHSRVSVTGDELAPLSRSSTNHSARILSP

TPGALTPQHTGGNSADDRAETMSVSSKRSARSASSSLQASRRGSSRNVAEYSDPHTHTNN

VIDIEDFIQRLLDAGYSGKRTKSVCLKNEEISLICAKAREIFLSQPSLLELSSPVKVVGD

VHGQYSDLIRIFTKCGFPPSTNYLFLGDYVDRGKQSLETILLLLCYKIKYPENFFLLRGN

HECANVTRVYGFYDECKRRCNIKTWKLFIDTFNTLPIAAIVAGKIFCVHGGLSPVLNSMD

EIRNIARPTDVPDFGLLNDLLWSDPADTINEWEDNERGVSYVFSKVAINKFLSKFGFDLV

CRAHMVVEDGYEFFNDRTLVTVFSAPNYCGEFDNWGAVMSVSEELLCSFELLDPLDSVAL

KQAMRKGKHERRQAQSLQQSRAN

>tr|C4XXF9|C4XXF9_CLAL4 Protein arginine methyltransferase NDUFAF7 OS=Clavispora lusitaniae (strain ATCC 42720) OX=306902 GN=CLUG_00632 PE=3 SV=1

MLRFARLLSRSATRLEKNPLIHENGLYYGKFTKEEYEEARKFVTTQYEKLQQEIKGTHNV

RENIGKMPQFPAQGPSRSVQNLSDFFSETIKTTGPVSLSAYMRQCLTHPDFGYYTTRDPL

AAGGDFITSPEISSVFGEMIGMWLFSVWQAQGSPQKIQVVEFGPGRGTLIHDAMAVFNRF

AKVSVSIVLIEASPVLRKEQAKLLCPGVEQFEKVPTPENPAGFDSCLSKWGHRVMWVDTE

KDVPSEVSYVVAHEFFDALPIKSFVRKEEGWRELLVDSADHRPALSGLESPAPADSTSDS

TATDSSSSTSGLTATSDTTATSDTSTSDSGSTSNGPTDSLFLTTAPRETPSSAIPSVSPR

FRDLPVGSRVEICPDAELYLSRIVELVKKGQNAGAALIIDYGLANDIPSNSLRGIYKHKF

VSPFFSPGNVDLSVDVDFENLRLLAAPHVDVFGPTEQGDFLHELGIGVRFDQLIQRANSM

ANKESLYESYVRLTGKDENSMGKIYKFLALLPKNSTRPAGFGTD

>tr|C4XXH1|C4XXH1_CLAL4 Guanine nucleotide-binding subunit alpha OS=Clavispora lusitaniae (strain ATCC 42720) OX=306902 GN=CLUG_00644 PE=4 SV=1

MGCSASTLAEDSQAYSKQRMMSEAIDRSLLLSSEAEHKQTRMFLLGAGESGKSTVLKQMR

LLHKNTFSDFERRQYAEVIWLGLVEAMKTLLLNARKFHIPLACDQPGSPLEPHKRVIWRT

RNAATDHDPSVGDFDFGSRAPPQTYDTAHLLSDSWRDDAFDMSSSGALQGLQGGSQRPSA

SGTPPSRDQIARAVEALWAQDPGVRRSLAQSHRFQMESSAAYYFDNVHKFKDTTYRCSDE

DILRGRIKTTGITENSFLIRNGVLKVLDAGGQRSERKKWIHCFQDIDVVLFVLAVSEYDQ

TLYEDTRVNRMHESFALFEALCNSRWFHKTPFILFLNKVDLLEEKLPRSPITNYFPDYDR

DPNSVGDVLDYMEAALLRLNKSKKPVYVHRTCATDTAGMSFVLSAVTDMIIQQNLKESGI

M

>tr|C4XXH2|C4XXH2_CLAL4 Calcineurin-like phosphoesterase domain-containing protein OS=Clavispora lusitaniae (strain ATCC 42720) OX=306902 GN=CLUG_00645 PE=4 SV=1

MPKLRPSHKLLALLAALWLVLFTYHEHIVPRTTAARCQWAPVEPAQTNVLLVADPQLIDN

HTYVGRNELLLGLSKHTVDVYIKQNYRALVEQLKPDRIFFLGDYLDNGRSSSDVYYEREF

ARFEAIFARWPQYKRGHTWFTDVPGNHDVGFGDGVRAEAQARFAAHFGSPNAVHLVNGVQ

FVSLDTPSYSSQSGEVRRASHDLVSSLMDTKTDHPRVLLSHVPLYRDTEALPCGPLRESA

RFDQGRGYQYQSALDPLVSAELLGKLKPQLVFSGDDHDYCDVQHVGSAREVTVKSISMAM

GIRYPAVQLLSYVAHGSDLSYDTHMCYLPPPYRDIVAYVTMAVASAVTLAVWSAIQRKPH

RSRDASDTAQKVSAFLEGAETGEPVPQYTVTVPRWAQMWYGLCSFCRRWHVGAFFRHAAI

LGAAAVAMYLVSVWLV

>tr|C4XXH5|C4XXH5_CLAL4 Splicing factor YJU2 OS=Clavispora lusitaniae (strain ATCC 42720) OX=306902 GN=YJU2 PE=3 SV=1

MSERKAINKYYPPDWDPSQVPKKRKNTNPNAEKVRLMLPFSMKCLQCNEYIASRRKFNAR

KEITPEKYMGIKIIRFHIKCPRCNNGIVFRTDPKSAGFAPVEGGVRNYESLAPQEKIKPL

ETEDEIFERLEREETENKRFQEQKAKRKNNPFWQAKDQGEKDVLESLEDKLADQQREQEI

HDHLAYLQAKSARLQQSGGVDHVTNVVHAQVSQALAKEKAIEDNDEDLEKAKVIFSTREK

RPMPASVSGVITVKKPKRKPVDEVKSAIIEKKDEVKESKTQTTGKPSLAVSALAGYSSSD

EE

>tr|C4XXI1|C4XXI1_CLAL4 Cation-transporting ATPase OS=Clavispora lusitaniae (strain ATCC 42720) OX=306902 GN=CLUG_00654 PE=3 SV=1

MPRSHSSPLRRANSMGSRGSRQNSISSIDTVLVDDNNYEMFSGATSEIIPSSISSFHYPH

HFGSRHASDASVLRETSPLLSSSHEPSNDLNLRTISSNATSASAETSGGFRFFSPDDIER

APGGSTLENPEDPVDYNTNWDYSVDRFEDHEEAPPLSTSRTSGSAVPSKFYEQKGSRPAE

QERLSRSPYQRAESDNESDAESILQDRFRRRSTDPGTEDSASSSSASFQSDAEDDSMQDF

CPTSLYQRFYLAEEDIVIGVAGYSNCLWKSVAYYLLCLCSFGAAYLVFRWLPRYRVRLMG

NPCPLGKADWCVVENEFGELTIVQVGKTRFGERLSHFMTVTQNQESDDPSLQAQDTKEAN

PVIPYIHSFEYRYIRFFYNPVEDIFKTNSTWYDMHWLNVKNLREGTSQTLYEYRESIFGK

NNIEIKEKSNLGLLADEVLHPFYVFQIFSIFLWLADDYYYYAGCIFVISLVSVMNSLFET

KATVRRLKEMSKFSCDIRAWRNGFWTQIDSNDLVPGDVFEVDPSMSLVPCDALLINGECV

VNESMLTGESVPVSKISATRDTVSYLSENFTHPVLSKSFLYNGTKLLKMKSSNDEPVLAM

VLKIGFNTTKGSLVRSMLFPKPTGFKFYEDSFKYIGFMTLIACIGFIYSTYNFIQLGLAK

RIMILRALDIITIVVPPALPATLTIGTTFAVNRLKKRNIFCIAPTRVNVGGKLDIVCFDK

TGTLTEDGLDILGVHAVKNAEGRKEIVFEDLVDNVKSLAPKTPSESPYGIQSGPQLLGCM

ASCHSLRLIDDVLVGDPLDVKMFEFTNWHFAEEFGGSSVPMVYESVGKETYGYKILKEYE

FVAALRRMSVLADKDDKRYVYTKGAPEVMLDICDPATIPSNFEELLHQYTHGGYRVIACA

QKSVSKKINHNGLERESAESDLQFCGFIIFENKLKPSTKGTLQELREAAIRTVMCTGDNV

LTAVSVGRECGLIHSSVSQVFIPRFAGEDEQNITGGTGLIWEDIHDANKRLDSVTLHRLS

TDTRDIMGEYILAITGDIFRYILAELQQEDLTHAILMRCNIFARMSPDEKHELVEQLQKI

DYTVGFCGDGANDCGALKAADVGISLSEAEASVAAPFTSRVFEISCVLDVIKEGRSALVT

SFSCFKYMSLYSAIQFITVTLLYKKGTNLGDFQFLYIDLVLILPLAIFMSWSGPYAKLVV

KRPTANLVSPKVLVPLVSHIAVILVFQLYVWLSIKKEPWYIAPEPSDDDDHVKSSDNTVL

FLFTNFQYILHAVVLSTGPPYRAPLHTNKPFLATVVFCLALSVGIFSIDSESWWGDFMQL

TNMSHGAYVMLLLAAAANFFVASYGESALYKKMAQGVKKALFGNRHSKKRFKRLGREMVP

VV

>tr|C4XXI3|C4XXI3_CLAL4 ribose-phosphate diphosphokinase OS=Clavispora lusitaniae (strain ATCC 42720) OX=306902 GN=CLUG_00656 PE=3 SV=1

MSTNSIKLLASDVHRGLAELVAQRLGLKLTPSELKRDSSGEVTFSIGESVRDEDIFIICQ

IGSGVVNDRVIELLIMINACRTASARRITVILPNFPYARQDRKDKSRAPITAKLMADMLT

TAGCNHVITMDLHASQIQGFFDVPVDNLYAEPSVVRYIREKVSYKDAIVISPDAGGAKRA

AGLADRLDLNFALIHKERARANEVSRMVLVGDVTDKICIIVDDMADTCGTLAKAAEVLLE

NNAKEVIAIVTHGILSGNAIKNINNSKLKAVVCTNTVPFEEKLDLCPKLDTIDISAVLAE

AIRRLHNGESISYLFKNAPLS

>tr|C4XXJ3|C4XXJ3_CLAL4 leucine--tRNA ligase OS=Clavispora lusitaniae (strain ATCC 42720) OX=306902 GN=CLUG_00666 PE=4 SV=1

MSATIKLEKTDRRDTLVEIEKKYQKFWDENKFFEVDAPTLEEDPEDDPDQLRETFPKHYI

TMAYPYMNGVLHVGHSFTLSKAEFATGFERMRGKRALFPLGFHCTGMPIKSSADKIKREV

EQFGADFSGVPAEDEEEEEQVKEEVKKVDPTKFAAKKSKAVAKQGRGKYQFEIMLQLGLS

KEEVAAFADPQHWLQYFPPLVKRDVSAFGGRVDWRRSMVTTPANAYYDAFVRWQINRLKD

CGKIKFGERYTIYSEKDGQACLDHDRSSGEGVNPQEYVGIKIEVTEFAPEAQKIFAENNF

DLSKNKVYLVAATLRPETMYGQSCCFVSPKITYGVFKAAEGVYYITTKRAFKNMCYQKLT

PTRGDHTATLTIGGGAIVGSKILPPLALLSNLRVLPMETILASKGTGVVTCVPSDSPDDF

VTTRDLANKPDYYHIEKDWVNTEIIPIVRTEKYGDKAAEYLVNELKIQSPKDQVQLAKAK

ELAYKEGFYNATMLVGKYKGEKVEEAKPKVKADMIAAGQAFVYNEPESLVVSRSGDDCVV

SLEDQWYIDYGEESWKSQALECLASMETYSKETRNGFEGVLEWLKNWALTRTYGLGTKCP

WDESQLIESLSDSTVYMAYYTIARFLHSDYYGKIPGKFDIKPEQMTDEVFDYIFTRREDV

KSDIPSSQLKAMRREFEYFYPLDSRVSGKDLVPNHLTFFIYTHVALFPKKMWPRGVRANG

HLMLNNAKMSKSTGNFLTLEQCVEKFGADASRVALADAGDTIEDANFDEANANAAILRLT

TLKEWCEEITSSQDKLRTGEYNFFDQAFENEMNFLIEQAYEQYELTNYKSALKYGLFDFQ

SARDYYRDSVESMHKDLVQKYILNQALLLAPIAPHFSEFLYKEICGQTGSVQTAAFPRAS

KPVSQGLLDSLEYVKELARAVREAEAVVLKSKKGKAEVDPSKPASVTLLVASSFPEWQDE

YIELVRQLFEANTLNDNKVIKEKVGKDMKRAMPFINFLKQRLAKESPKTVFNRKLTFDEG

ELLKQVVPNLAKSTTTVKIAEIKLISFPHGSKIGTDILTGEEVEITATGKVVEAAVPGEP

GIIIKNTE

>tr|C4XXJ8|C4XXJ8_CLAL4 orotate phosphoribosyltransferase OS=Clavispora lusitaniae (strain ATCC 42720) OX=306902 GN=CLUG_00671 PE=3 SV=1

MQASQKVFLDHALECQALKFGSFTLKSGRQSPYFFNMGLFNTGKSLATLAAAYAQAIIES

GIKFDILFGPAYKGIPLAAVTVAKLAELDPENYGDLGYSFNRKEKKDHGEGGSIVGCALE

GKKILIIDDVMTAGTAINEAFEIISAEKGTVVGCVIALDRQETTADSNQSATQAVSARYG

IPVFSIVSLTDIVNHLKEKLTPEKLAAINEYRTKYSPSS

>tr|C4XXK2|C4XXK2_CLAL4 CAAX prenyl protease OS=Clavispora lusitaniae (strain ATCC 42720) OX=306902 GN=CLUG_00675 PE=3 SV=1

MNFYALLFCLAVASSYVLAIRVHVPPRLACLDRNAPELVRFRFWRISLLCVFTVLFVPWV

NWALLGLYPSYISAIRQLGLVPGFSLSGSVLVDSANIAKTTLKMALLYIGPLAQYALSKP

DLASDLSSSYLSVWGFRDHVFGPITEELVYRAAVVSVLRPIADAHKITIWAPSLFGVAHL

HHGIQLLRDGVPLTDASAQVAFQFIYTTLFGILANHVYLSTQCNLWCAVAMHAAANLGSF

PSFELRHKHPRLFIVYCASLVAGAVSFYKLL

>tr|C4XXK3|C4XXK3_CLAL4 Homoaconitase, mitochondrial OS=Clavispora lusitaniae (strain ATCC 42720) OX=306902 GN=CLUG_00676 PE=3 SV=1

MLFRRGLSCSAVLARGQNLTEKIVQKYAVGLPSGKKVFSGDYVSIQPAHCMSHDNSWPVA

SKFMGLGADKVHDNRQIVCTLDHDVQNRSEQNLTKYANIQKFAERQGIDFYPAGRGIGHQ

IMIEEGYAFPLSLCVASDSHSNTYGGVGALGTPIVRTDAAAIWATGQTWWQIPPVAKVEL

VGSLRPGVTGKDVIVALCGVFNHDEVLNHAIEFVGDGVSSLPVDYRLTIANMTTEWGALS

GLFPVDDVLVDFYHQRLQKLPQPHPRVNKETVSQLEKNREQSDSDAVYAKHLRIDLASLS

PCVSGPNSVKVSAPLYELAQQQIPINKAYLVSCTNSRLSDIQAAADIIRGRKVAPGVEFY

VAAASANVQSDAEATGAWEDILAAGAVPLPAGCGPCIGLGTGLLKDGEVGISATNRNFKG

RMGSKDALAYLASPEVVAASAVLGRIGGPEELDGTPVPAPGEIVKSIEIAKPAESGDASA

SVDVLDEFPKSIEGELILCDADNINTDGIYPGKYTYQDDISKEKMAEVCMENYDSEFKNK

TKASDIIVSGYNFGTGSSREQAATAILARGMKLVVAGSFGNIFSRNCINNALLTLEIPAL

INQLREKYSGSNELTIRTGWFLKWDVTKALVTITDSEGNVILEQKVGELGTNLQDIIVKG

GLEGWVKAQLAKA

>tr|C4XXK6|C4XXK6_CLAL4 Dipeptidase OS=Clavispora lusitaniae (strain ATCC 42720) OX=306902 GN=CLUG_00679 PE=3 SV=1

MDSADRFRALAKKHPIIDAHNDFPYSVRVQLHYEVENDDAFTFKQGLTCHTDLKKLHVGG

VGVQFFSCFIECKDDNPLYQNFNKPTTVVRDTTEQIDFVRRLTRMYPDDLKLATCAQDAL

DAFEKEGKLAIAMGIEGLHQVDASLGVLRTYFDMGVRYATLTHNCDNPFATAASSVAGGL

PDKGLSDFGRKCVREMNRLGMMVDLSHVSVQTMHDALEEAQAPVIFSHSSAFALTHHVRN

VPDEVLEKVKKNGGVVCINFYPAFIKRPGEDSATIDDAVAHILHVANLIGWDHVGLGSDF

DGIPEGPKGLEDVSKYPDLIKKVMEKSQATDEQVAKLMGGNLLRVWKENERVAAELKKEK

VIDENWPDRIWEFFSYCKEFPEVYPGSYQKHQNIYKDAQKLDVIGDMKTNN

>tr|C4XXK9|C4XXK9_CLAL4 Peptidase M20 dimerisation domain-containing protein OS=Clavispora lusitaniae (strain ATCC 42720) OX=306902 GN=CLUG_00682 PE=3 SV=1

MQFFLFFVAWFSLVLAVRPFHFKAPQEALISVFDSQSLSDYVVDSKYSLLELHKKLVEIK

SISDNELNVGQWLGAYLKKAGLTVELGKVDGENDRYNVYAYLGKKRDTSIVLTSHIDTVP

PYLPYYVNGTQIHGRGSCDAKASVATQVIAFLDMVSQGILKEGQVSLLFVVGEENSGSGM

RKATESLGASWDIAIFGEPTENKLAVGHKGILLFDVEVFGKASHSGYPELGVSATEILVP

LLANLQNLELPTSDILGPSTLNIGKIEAGVAANVVPAYAKATIAIRVADDLARVVHLVRS

VVEDTPHVGPFSFFGTEPQFLDYDVPGFGTLVAAYTTDVPNLGLPLKKRYLYGPGTIHVA

HGANEYVENQDLLDAISGYKKLIQHALSEV

>sp|C4XXL5|GEP3_CLAL4 Genetic interactor of prohibitins 3, mitochondrial OS=Clavispora lusitaniae (strain ATCC 42720) OX=306902 GN=GEP3 PE=3 SV=1

MLKAQIQTGLQLLQRAAVSHMRPSSCTSMLMRMRVHLAPRALQSQRSLSSSEFSPLIADA

ILPKCSSCGVLLQNEDRSKPGFYLAPGSSRDFRKETDVVYEKHLASLEEADRDLLLNGAQ

GLQVSGQKETPKSQKQPANKVSCIRCRDSHYRSQFPLDQFAVAAVSDVMHSIPAYANLAY

VVSATDFPMSINEDVFRHRSPREMQFVVTKNDLFFQKNSVASKYGLQFYQDYFWRMFNVP

VENVHCVSGTVDWNTEKLFDSLRDNTYFIGCVNSGKSTLIQSLVHLAHKRRLALPNAKRD

RALQKIDNQVISTQQEPKTRQALIKHNRALASAFKKQNGPGASYMPGFTRGNLPFELSRS

VTIYDVPGFSSAQTAQLYDFLAPQAIKALHKGQKVYKAGTYKSHYETLKSGQVLTVGGLF

FLQAPKNTMFQVKNLISHPHHIFKDMEKAMDVWRSPEIYPALKNVFVVPGTRNNAPTPLV

KHIVPSFYGSIDLVLRYLGYVSLKPTGAKDPESGPLVVYLPKEVDAIIRQPITKYISRTL

SGRDANGNVLRKENWVQKSVTEVKRYTGKTPFTSRLIPAVGEDASADEAEVMERCVEKIK

GHAVAHAQISEETKYANWL

>tr|C4XXM4|C4XXM4_CLAL4 Acyl-coenzyme A oxidase OS=Clavispora lusitaniae (strain ATCC 42720) OX=306902 GN=CLUG_00696 PE=3 SV=1

MVAATFVSNLPAPEPAASMAQERARAAFDPAEMHQILEGTPEKAQQILGLYQSLERDPVL

APSFYDYEMSRDENRLQTTRRIARMAQYVEKESPEDFWRRLGLVTAYDPSLGIRIAVNLG

LFVNCIKGNGTAAQYKYWCVEKEARHMKQVWGCFGMTELAHGSNAAGVETTATFDEKTDE

FVINTPHIGATKWWIGGAAHSATHSSVYARLIVNGKDYGVKTFVVPLRDSQHNLMPGVSI

GDIGSKMGREGVDNGWIQFSGVRIPRFFMLQKFCRVERDGTVKMPPLEQLSYISLLEGRV

GMAADSYRICARFITIAVRYAVGRRQFKKEEKKKDSDNLETQLLDYPLHQRRLMPYLALT

YAAALGTDRLERQHKFVVSSLEEAVNNDDQNGIKRSLGDTKALFVDSAALKSTLTWLAER

CLSETRQACGGSGYSAYSGFGQAYADWAVQCTWEGDNSVLGMSAGRSVMKKVADVLTKKS

GISHNKVSGQGKTLSIENGNVSKHQLGVSKTNSRAVSSSSPGLFNSDTLSFLDNASSYLT

GSYLESDFAPKTVLTALEALIVRVAASALESKDGWDSVSYERVLLSRLRCHQYLLHTLVQ

VLDRKKTPDLQEPLEKVARLYALTSIVETYAREFIAHGIMSGEASLIVTSKLIPQSCLEV

RQQAIPLTDSFQIPDTLLNSAIGTHDGNWYENYFRVVKSHNDARKTKAPYSHELEGFLNR

PNLDARERHERGHEAQKKLSG

>tr|C4XXM5|C4XXM5_CLAL4 Acyl-coenzyme A oxidase OS=Clavispora lusitaniae (strain ATCC 42720) OX=306902 GN=CLUG_00697 PE=3 SV=1

MSVVSNSVDSAAPPNPRALLAAERAQSKFDPATMNYFLETSKETSDTIKEFVQQLERDPI

MNPGAKQYELTKEQQRVVTARRIARLAQYAETQNDPTFQGLRASIMSVFDPQVGTRLGVN

LGLFIGCVRGNGTKDQYDYWYKQKETGYVRGIYGCFGMTELAHGSNVAGLETTATFDEKT

DEFIINTPHIGATKWWIGGAAHSSTHCSVYARLIVKGEDYGVKTFVVPLRDANHNLMPGV

AVGDIGAKMGRDGIDNGWIQFSSVRIPRFFMLQKFCKVTAEGDVTLPPLEQLSYSALLGG

RVSMVMDSYRWSAKAVTVALRYAVCRRQFKSKSQADGSETQLINYPLHQRRLFPFLAQAI

VFSAGSWKLEHTFNEVLDTLDKAVETNDMKRIFASIDAMKSLFLDSAALKSTGTWLTAEC

IDQCRQSCGGHGYSSYSGFGKSYNDWVVQCTWEGDNSVLAMSVGKPVIKAVVAVLEKGAK

EKGSLSFLNNAAKYDNDEIVFSNTSDLLDFSNVLRAIEVLITRIGIQGAKVVADNGGNFD

SVGAPSLAIAKLKAHHYFLEEFHRRLQSNEYKELNPYLELIGKLYCASNVIEKFAGDFLT

YNVIPGSVAREITNVTLPALCAEVRKNVVAYTDAFQFSDSMLNSSLGKYDGDVYENYYNT

VKTLNPPENHKAPYSAEFEAALNRGPLDARERYEKGAEVAAIFV

>tr|C4XXN1|C4XXN1_CLAL4 Ribosome assembly protein 3 OS=Clavispora lusitaniae (strain ATCC 42720) OX=306902 GN=CLUG_00703 PE=3 SV=1

MAPPSVNNKQKRNRRRKKRRTEDFSSDSDSDSDSSSSSAPSDKEEEKEKPTANINIDDID

IESDTEHATERANDEPLSAETQQQLKKIKFTSTANQSIAEAKETVRKDRQQLENEFLAHM

ATGFANDLDELRKKPDFNEKSIVILAKTLQSGSNMFDEETLNALLK

>tr|C4XXN5|C4XXN5_CLAL4 Calcineurin subunit B OS=Clavispora lusitaniae (strain ATCC 42720) OX=306902 GN=CLUG_00707 PE=3 SV=1

MGLASSKVLDSLMDGTNFDREEIDRLRKRFMKLDSDGSGTIDKNEFLAIPGISSNPLAQR

LMDVFDEDNDGTIDFQEFITGLSAFSGKTSRDEKLRFAFKIYDIDQDGFIATGELFIVMK

MMVGKNLSDAELQQVVDKTIMENDRDGDNKLSFEEFRNAVDSTSVANVLTLNDF

>tr|C4XXN9|C4XXN9_CLAL4 Endoplasmic reticulum-Golgi intermediate compartment protein OS=Clavispora lusitaniae (strain ATCC 42720) OX=306902 GN=CLUG_00711 PE=3 SV=1

MDNFSSKVRVFDAFPKVAPEASVRSQRGGFSTILTVFCGLLIIWIQIGGYLGGYIDRQFS

VDNETRKDLNINLDMVVAMPCQFISTNVMDITSDRYLAGEVLNFQGTGFYVPEFFALNRE

NNDYDTPELDEIMQETLRAEYGIAGARVNEDAPACHIFGTIPVNHVRGEFFIVPKGSMYR

DRSSIDPKAYNFSHVISEFSFGDFYPFITNPLDFTAKVTEENRQAYRYFAKLVPTHYEKL

GLVVDTYQYSLTEIHNVDHNRGIPPPGIFFDYSFEPIKLTIREKRIGFFAFVARLMTVLS

GLLIAAGYLFRLYEKLLALLYGKKYVERDTEKRSGGLLDKSFTEKNI

>tr|C4XXP1|C4XXP1_CLAL4 Protein kinase domain-containing protein OS=Clavispora lusitaniae (strain ATCC 42720) OX=306902 GN=CLUG_00713 PE=3 SV=1

MLNMLLLNIEPADDKGPRYIRADHYKKVSFGFRIMSNLSPSFNTRFASIGIATDADAARP

PKKPAKKSYPASLYPENYHRETATTPSRAPVLSTAKRAVSRHSNPLKQINSSSTATKRHL

NRPLAESADPETQSPCKSKPRKAPDLSSCSFMKETSSSRNRQALAEKQARQAERSSIQTR

AASHSEIYNRRPGHLGTKKRLFPKSAKSTDLASLATKNANVPEHTSPRHVSTAYTNRDKE

DVYARLYNNALRSKTRLDKSRNTSNRRPSSRSRSPNCPSIPLEATMFDGSRSMDSRKPWT

VNDVYQIIFAADPSIFNETNSSDFSIPPVAGTDPRQLLDSTEGAALSIFERGELMRKSNV

YYLPNLGSGRDESSINISSFKNNYGFDDLEGNYVIHQGDHIEYRYEILRILGTGSFGNVV

LCADRKYQNHVDSRKVAIKIIKNELDWSLQAVSEIKLLKSLNTPPHASQYIMNYCDHFHF

RGHMCIVSEVLSLNLYTFLELTSFSGVRLALLRKFARDILRGLSFIHDTHIIHCDIKPEN

IMIKLPPYYDPNQTSISDFLVKIIDFGSSCYENETSYSYIQSRFYRAPEVILGAQYGSEI

DVWSFGCVIAELFSGTPLLPGKSELEQMGLILEMFGAPSCTHIINERKRLMRQVKIARSK

GLSDPLVSDPSSLLSKGKPPVDERKIKKTLLYSLFNLEGKLNLKFLNTQLQSVSHKTSAP

SPFKRTIKPNSKSIEVALRLHSSHEDRNDISHFSKFLHSIFRFERETRATATELLGHVFL

NE

>tr|C4XXP3|C4XXP3_CLAL4 ABC transporter domain-containing protein OS=Clavispora lusitaniae (strain ATCC 42720) OX=306902 GN=CLUG_00715 PE=3 SV=1

MSHFSKSRSDSYARSIVNAYKSKRPILLNISYLLLFIAAFTGATSTSNKKVRKESTEKKA

KEKKTPRLSRNDLKTLLKSAVPGVSDSVVGYLAGHIVLLVARAFLTIEVASLDGKLVGAL

VTKRLRKFARLLAFWMFLGIPASFVNALLNWTKEKMRQSLRRNINDSILHDYLPDNLDPN

YYALLQSADSDIKDPDQRISTDVSRLAQALASLPGQLLKPSLDLFLCARELSRSGVGSGE

GTLALGLLTHFSTTILRFFSPPFAKLASERASLEGQLRSAHSKVVANNEEIAFLRGHPRE

LDYVDYCYYQLERFLKIEYSKRAIHEIAQTFIVKYFWGAAGLVLCSAPVFINKYFGKAED

PNATSDFITNRRLLLSASDSIDRLIYSRKYLLQVVGHASRVTSIQKALNTIKQKRLGNIS

ESSTGTVTYGNEITFDRVRLVTPADVTLIESLSFSIKPGQHLLIAGPNGAGKSSMFRMLG

GLWPCKEGHITIPTSDNMFYLPQRAYLCKGSLREQITYPHTLEEYKQNKHGKTDKDLKNI

LDILDLGDLLQSDSDWDAVKNWKEELSTGAQQRLAMARLYYHQPQFAVLDECTSAVTPSM

EQFMYQHAQEIGISLISVAHRPALWHFHNFLLKLDGEGGYFFGPLDASRRLKFEEERLML

EKNLRDVPQLRERLEELKNVSTAHHIRHESSQRSLTNMA

>tr|C4XXP7|C4XXP7_CLAL4 Zn(2)-C6 fungal-type domain-containing protein OS=Clavispora lusitaniae (strain ATCC 42720) OX=306902 GN=CLUG_00719 PE=4 SV=1

MAREKRTKPCSNCKRSKVKCEYVESLPCTRCVNSGLSSSCHFVPKLPSLKLPSLDVQRQN

KYPGPDILPSNFLSQQGGSVPPQANPVLYYPQAIPVQPEAKSIANHSAPLHQSPSNPSLV

NISKAEVASTPNRLVGPQNGRSSYFYPSQKVKNNSIADQNRLEIHDSRQNPHLHQKNSLS

VSQADAEWKITMENKLNSFDSKFDHLLQALRENQRALGHEMELKNRYAAENEYFRQQHIE

LSSRVPTPLIGSKRSYSESTSASPPKKTKYGDDDFRENVISLEEAKALFSYFDTNISQQL

FGFEIKQFSVDVIWEISPILICAICTIASMHYPDPVKSSKRDALQKHLHDLCAKLLFKGR

PRTEEEGFNAIVALVLCSFWLSDSQRFTGLALQLAKEFQFNHPSASKTQSSLSEKDKLKL

WYLLYILDGQQSMTLNRQYLLDGNDESFKNSRSVLLSRTSSIKSSPTQAIDQSEVSKGRQ

TDQKESKNSKSSDESKAPSFTDLRLVSQVEYNQALNEAFKGNAWELIAPSAFGIPSKSNL

ELDKWMVSWTVLLAPMNCGSVWSSKSTLIYYNFAKMHINSSIVRHLQMDTGSEDVVFPRW

ENYSQVSEKQKSPAIDHGARQDSDDEDEDDDDEFISNKELVSQDETLLGLNIAVNAAQTV

LNLVLDDNDILSELKYVPVHIHIMLYYAALLLVNPPTCVTDGEKNEAYFQKIIENLKTVR

ILSKKVNMNIPTDKQFGERFINSLDNVFLERSTRLKSELNESNLDLSVRAELLKELSSLQ

YFDSKLEMVLDTKESNRGSSPKPERISAWPGSHHGHP

>tr|C4XXP9|C4XXP9_CLAL4 RNA helicase OS=Clavispora lusitaniae (strain ATCC 42720) OX=306902 GN=CLUG_00721 PE=4 SV=1

MLKLATILLCSYMSHIQKGRGYSRKRDYSPDDEPIDDTSVINSEYPQPNNAPVITPEHQS

EQANATIARQDNVGGQPTTKEHKGLGKESEAATSVSNDNGPDIDHLRKKSRRNYIHSKFE

TALDDLRKDVALLESRENRSATETKALNEKKRILDLMVSKEEQPDAEFEIPDEYFTSGGA

IDQKRKRELLKSKYVEVSEKEEKKKRFENQWELDQLKKAQTIVSSNPDEIQLPTQPTYDF

VFDESQFVDFSESEPTSHEPVEPTDIPKVSHEIEEVRKSLPVYQLRQSFLDTIEKNQVLI

VVGETGSGKTTQLPQYLYEAGYTKAPNSDIPLKIGCTQPRRVAATSVATRVAEEVGCVLG

EEVGYCIRFDDSTSQKTAIKYVTDGMLLREFMADPLLSTYSALMIDEAHERTVSTEIVLT

LLKDIIKERKDLKLIVASATINATKFSEYFDGAPIFNIPGRRFPVDICYTKNPEANYIQA

AITTVFQIHLKEEIPGDILVFLTGQEEIETMEETLNDACQKLGDSIKKMIVAPIYANMPP

KLQKRIFEPTPHDARKVILATNIAETSITIDGVRYVVDPGYVKENVFNPSTGMESLVVVP

CSRASADQRAGRAGRVGPGKCYRLYTKWSFYNELQANPTPEILRVNLSTIVLLLLSMGIT

DLVHFDFMDPPNSQTLIKSLELLYALGALNSKGELTKTGRRIAEFPMDPMFGKCLLSSDE

FGVTAEILSIMAMLSESGSLFFRPKDKKEQADKKKETFAHDLGDHFVLLNIWEQWSESGF

SNIWCEDNFLQYKTLRRVKDVRTQLENLCRKIGLDVEQREDIEEQDVKIQKTLLSGFFPN

VARLSKLGTNYVSLKKNQSVFIHPSSSLFPVKPPPKIILYHELVLTSKEYMRNCMIVEEK

WIQEIAKHYYQKEIKNISGRP

>tr|C4XXQ1|C4XXQ1_CLAL4 alanine transaminase OS=Clavispora lusitaniae (strain ATCC 42720) OX=306902 GN=CLUG_00723 PE=3 SV=1

MIVAPHMSVHPFSPLFFKTRAYFSAGRHVSYLKVNRVIEKKKFRLLSIMSSLRLSSRSLL

LKRLYASFKPAPQLTVSDLNESTLEAKYAVRGKIPIRADELRSEIDSNPDGHGLPYDRII

SANIGNPQQLDQQPLSWYRQVLSLMQYPTLINKISTLDKKTADSLYPPDVVERARKLLKT

TGSVGAYSHSQGDITVRKSVAKFISERDGYAAHPQNIFLTSGASSAVSYLIQVLSSSPNA

GFLIPIPQYPLYTASIALNNAKPIGYFLDEDSHWSTDPVQIRRLIDENKANGVDLKALVV

INPGNPTGAILTEKDIAEIIDIAAEHGLVLIADEVYQENVFEGKFVSMKKVYAQLLEKDP

ETYKHVQLASLHSTSKGVSGECGQRGGYMELVGFSQEVRDIIFKLASINLCPVVSGQALV

ELMINPPKEGEPSYELYQKETTGIHDDLMKRASLLYKSFCAMTDVQCNKPQGAMYLFPSL

KFTKETYSKLFEASEELNLTPDEYYCTELLEKTGICCVPGNGFGQVPGTYHLRTTFLPPG

TKWIEEWTKFHEDFVSKYKN

>tr|C4XXQ9|C4XXQ9_CLAL4 GPI transamidase component OS=Clavispora lusitaniae (strain ATCC 42720) OX=306902 GN=CLUG_00731 PE=3 SV=1

MNNIGNFAYYKKESVRCIFLTATELLISYINLTMTTSQEVHYITKARRSIIFGVLLFILS

CGVPLFLYTTSIHRAELPSHEVEAKINSFRDLVRFNIPVYVRIDPSHESLVQEWQDSLDS

RNNNDGLGGKWSLQLKLQDEEALKNEVYTLDVNIVSTSDQNSYKVSSSSGNISLNVEPTA

NGSEFVTNVLLNSIFGDERKQILSILGIKEDEKDSDIVFPYSNTYNVVFNLFVENGRPVE

WDVESAIQAMNPVFDALSNYANFKISTQVQLYSKLGSSTRYAEDLKANIIPKSELSTFIN

FGDWNLITHDINPSINFILYFSKSNYEKVPLLVEGSNTNSFLIPQWGGVYIYNRKMPIIN

DTIFKLNTYELQPIFEIFTSQLFELLGVPKNSESPWIRIDSLNRVIALKNLKRSLENLSS

LIKLSTSLNEISIPESTKFHTEQALAYYDQAMTELANSHFEEAIVLASKSVESSDKAFFE

KEMVQQAYFPSEHKLAVFSPLLGPLCTIVLIGFLSTVKDMKSKKQGPKKVQDKDE

>tr|C4XXR2|C4XXR2_CLAL4 Aconitate hydratase, mitochondrial OS=Clavispora lusitaniae (strain ATCC 42720) OX=306902 GN=CLUG_00734 PE=3 SV=1

MLSARRSLRSPVSFIRRSLATSSDFASAFEVPHEYKSRTPPYAKLVGNLQTVQKVTNGKP

LTLAEKILYSHLVDPEETFASVNGDLAQVRGQQYLKLNPDRVAMQDASAQTALLQFMTTG

MSSTVVPASIHCDHLIVGKEGADADLPSSIATNKEVFDFLQSCGEKYGIQFWGPGSGIIH

QIVLENFSVPGLMMLGTDSHTPNAGGLGAIAIGVGGADAVDALTGTPWELKAPKILGVKL

TGQLSGWSSPKDVITHLAGLLTVRGGTGYIVEYFGDGVESLSCTGMATICNMGAEIGATT

STFPYQEAHRRYLVQTGRGPIADAADVALNKFGYLKADENAQYDKVIEIDLSSLEPHVNG

PFTPDLSTPISKFGQTAKEESWPSKVSAGLIGSCTNSSYQDMSRAASIIRQAEEAGLKPK

IPFFVTPGSEQIRATIERDGLIDVFERNGAIVLANACGPCIGQWDRTDVSKDSSDFNAIF

TSFNRNFRARNDGNRNTMNFLTSPDVVTAMIYSGDMNYNPVTDSIEKENGEAFKFAPPKG

EELPHDGFIKGRSEFYPEANPQPKPEVQINVSPESDRLQILEPFAPWSGDELSTTVLLKV

EGKCTTDHISAAGVWLKYKGHLENISNNTLIGAVNKETGEVNKAYDLNGDAYTIPDLMIK

WKDEGRPWIVVAEHNYGEGSAREHAAMSPRFMGGSIILVKSFARIHETNLKKQGMLPLTF

ADESDYDKISAGDQLTTVDLKDMVAKNGDNGGVLKVKVTKRDGSEFEILCKHTMSKDQID

FFKAGSAINYIGNLKKQQAAESSA

>tr|C4XXR3|C4XXR3_CLAL4 Vacuolar proton pump subunit B OS=Clavispora lusitaniae (strain ATCC 42720) OX=306902 GN=CLUG_00735 PE=3 SV=1

MLSDKELFELNKKAVTEGFKIKPRISYNTVGGVNGPLVILDNVKFPKFNEIVNLTLPDGT

TRAGQVLEVRGSKAIVQVFEGTSGIDIKKTRVEFTGENLKIAVSEDMLGRVFDGSGRPID

KGPKIFAEDYLDINGSPINPYARIYPEEMISTGVSAIDTMNSIARGQKIPIFSASGLPHN

EIAAQICRQAGLVRPTKDVHDGHEENFSIVFAAMGVNLETSRFFKQDFEENGSLERTSLF

LNLANDPTIERIITPRLALTTAEYLAYQTERHVLTILTDMSSYADALREVSAAREEVPGR

RGYPGYMYTDLSTIYERAGRVEGRNGSITQIPILTMPNDDITHPIPDLTGYITEGQIFVD

RQLSNRGIYPPINVLPSLSRLMKSAIGEGMTRKDHGDVSNQLYAKYAIGRDAAAMKSVVG

EEALSTEDKLSLEFLEKFEKNFIAQGAYENRSIFDSLDLAWSLLRIYPKEMLNRISPKIL

AEFYERARDQDDDEEEEEEENVDKSHDLIDA

>tr|C4XXR9|C4XXR9_CLAL4 Trehalose-6-phosphate synthase OS=Clavispora lusitaniae (strain ATCC 42720) OX=306902 GN=CLUG_00741 PE=3 SV=1

MSQGKVLVVSNRLPVTITKKDDGSYDYSMSSGGLVTALQGLKKSTEFQWLGWPGLEIPEA

EQETMNKDLMDKFKCTAIFLSDTIADLHYNGFSNSILWPLFHYHPGEMNFDENAWAAYIE

ANRKFASKVASQVEDNDMVWVHDYHLMLLPQMLREILGNSKKNVRIGFFLHTPFPSSEIY

RILPVRKEILVGVLSCDLIGFHTYDYARHFLSSVARIVSDVKTVPNGIEYQGRSISIGAF

PIGIDVDKFTEGVKKPNAVERIKQLKQKFKDVKVIVGVDRLDYIKGVPQKLHAFEVFLQE

NPEWIGKVVLVQVAVPSRGDVEEYQNLRATVNELVGRINGQFGTVEFVPIHYLHKSIPFE

ELISLYHISDVCLVSSTRDGMNLVSYEYIACQEEKKGSLILSEFAGAAQSLNGAIIVNPW

NIEELSEAIKEGLTLPEEKKALNFKKLFDYISKYTSGQWGETFVKELYKCNSDENTSK

>tr|C4XXS0|C4XXS0_CLAL4 Protein YIF1 OS=Clavispora lusitaniae (strain ATCC 42720) OX=306902 GN=CLUG_00742 PE=3 SV=1

MYNPYASAPEAYPRQGNLHHPQPHHVKPSYSPSQQYQQPAANQQYSQQFQPQHDMPQQGF

PQQGFPQQGFPQQQNAFPQGTSPAAQGSNSMPYSNFLSDPAASMAAQFAKSGFGQSNNYI

QQNFGSYMPVAGDLKYYFKVSNSYVWNKLRLILFPYRHRNWSRMTTAESAGSSGAGVSYA

PPSEDINAPDLYIPLMSFISYILLWALFSGLRGDFHPEVFGYLASQTLACSFLDILIFKI

GLYLLNCSTQSSFWDLVSFSSYKYVAITVLLCWKNLFGGSWLAFYSVLFVLVGSLSLFLM

RSLKFLVLPVGAGDTSANTISNNQRRLRVQFLFLYSVVVQFMIVIFMSR

>tr|C4XXS1|C4XXS1_CLAL4 DNA polymerase epsilon catalytic subunit OS=Clavispora lusitaniae (strain ATCC 42720) OX=306902 GN=CLUG_00743 PE=3 SV=1

MPATATTTRFKGSTSARFVKNTSKPTKITSRYRVSKQAEDESVQLNPMLNDSVTKRDVVQ

RIDQLDAMMGFERFDQGEYDGSKPRKGWLINMHATTVPTDDYLAGYSGVDFYFLDQEGGS

FKATLQYDPYFFLMVVPGKEPDVEESLKRLLEDFHVKSISRELKEDLSLPNHLVGLKRTL

IKLTFHNTTDLIGARRLLNPIIKENQAKRDTRDVFRIYNFNNPSGISSATDGFQSNESKQ

DASTDPTTFIDDIREYDVPYHVRVSIDNNVRVGKWYDVYAKHGKITFEEDKEKIAFADPV

VLAFDIETTKAPLKFPDAKVDQIMMISYMIDGEGFLITNREIISEDIEDFEYTPKPEYPG

NFTIFNEPDEKHVLMRFFEHIRDVRPTVIATFNGDFFDWPFVENRTNFHGMDLFNEIGFA

KDDEDEYKSKYCVHMDCYRWVKRDSYLPQGSQGLKAVTTAKLGYNPLELDPELMTPYAYE

RPQILSEYSVSDAVATYYLYYKYVHPFIFSLCTILPLNPDEVLRKGTGTLCEMLLSVQAY

EGNILLPNKYTDPIERFYEGHLLESETYVGGHVESLEAGVFRSDIATDFKIDPTAIDQLL

RDLRETLKFTIEVENHKSMEDVENFEEVYIAIKKQLESLRDSPVRNETPLIYHVDVASMY

PNIMTSYRLQPDSMKSEEDCAACDFNRPGKNCDRRMTWAWRGEFYPAEQNEYGMVKRTLQ

NETFPGTRPWLPARSFDELPYAEQASLIKKRISDYSRKVYNRIKQTKTVNREAIVCQREN

PFYVDTVRGFRDRRYEFKTLAKVWKGKASKCKESISKDEANKMVILYDSLQLAHKVILNS

FYGYVMRKGSRWYSMEMAGITCLTGATIIQMARALVERLGRPLELDTDGIWCILPKSFPE

NFNLKCKDGKKIFVEYPCSMLNYLVHQQFTNHQYQDLIDADTFKYKTRSENSIFFEVDGP

YKAMVLPTSKEEGKGLKKRYAVFNKDGSLAELKGFELKRRGELQLIKNFQSDIFKLFLEG

DSLESCYQAVSTVANNWLDVLETKGGMLEDEDLIELICENRSMSKALSEYEGQKSTSITT

AKRLGEFLGEEMVKDAGLACKYIISAKPLESPVTERAIPVSIFSSEHKEIYLKKWLKDPS

LTDFDPRSVIDWDYYRERLASVIQKIITIPAALQNVKNPVPRVSHPDWLQKRIRVKEDKF

QQTSLSKFFNKASNEEVKTKAIKDIEDFGEVDEGVPKTKLGKVTVKKRKMREAQRLQAEE

DEKAVAILNSPCPSMTEDYVSFLQYQKAKWRVQSKNRDRRKKLFGENVDSSYRSSVGSMF

PPTSRECCWLGLGNFRI

>tr|C4XXS8|C4XXS8_CLAL4 IMP-specific 5'-nucleotidase 1 OS=Clavispora lusitaniae (strain ATCC 42720) OX=306902 GN=CLUG_00750 PE=3 SV=1

MTSRYRVEYALKSHRRDEFIEWIKGLLAVPFVLHSDIENYSQEYVLEGLENEMDVDDSQL

ERTCQARYLEIFNDVEKLVDNTIFLDTFEPDEQHVSRSRLRSLVPTLGRFFTRLPLKEAF

LIEDKKRSISRRRLVSPSFNDVRSILNTAQVLALTSNAQSKLKLVTFDGDVTLYDDGKSL

TSDSPLVDCLVNLLSLDLFVAVVTAAGYPGHQGAFEYHKRLLGLIEVLRETDKLSEKQKS

NFLVMGGESNYLFRFSSESGNLEFIDGQEWYLPMMRQWDQLKLIEIMNETHTHLRFLRKK

FRLDDDSKTKIIRKERSLGIIPLHGYKINRETLEEIVLSLSAKLHSLLHPRGPQGVNLLS

TDENTASRKSTSDFRVCAFNGGSDVWVDIGDKALGVQALQRYLCRFHVDDDTCPISKSES

LHIGDQFASVGANDFKARLSACTVWIASPRETREILEDLIEGLRNRGSQNGKKQY

>tr|C4XXT3|C4XXT3_CLAL4 Diacylglycerol O-acyltransferase OS=Clavispora lusitaniae (strain ATCC 42720) OX=306902 GN=CLUG_00755 PE=3 SV=1

MALIYIPRQMARLFTVSETGCVTSFCGKKFVDYFPIRVYKTCELEPTFTNVLVESEDVPD

DEDDLVSEYARTFIDDIFKFFGLKKRLNDSDSSLAELRNASKFGSDDKGSIKNPLRYKRV

STGPRYIFGYHPHGVISMGVMGMAATNALRNEPYEPPLKFLKPLFHDPSKGERLFPGIGY

IFPLTLTSQFALPFFRDYLLGLGLTSASAKNIKSIINNGDNSVCIVVGGAQESLLNDMVG

DTRVGIGYKAPPDSDDEDENEEKKEKKRKPIRLVLAKRKGFVKLAIELGNVSLVPTFAFG

EADVYRLVKPSPGSWGYSFQQWMKRAFQFTLPFFSARGIFIYDFGFLPYRTPINIVVGRP

IYVPSGLLSEQQEVPKSSSRGRQSSLTNLLSLGKTRKPQNRKHIPQDMLDHYHGLYVEEL

KRLFEENKGKFGYGDVELILE

>tr|C4XXT6|C4XXT6_CLAL4 V-type proton ATPase subunit a OS=Clavispora lusitaniae (strain ATCC 42720) OX=306902 GN=CLUG_00758 PE=3 SV=1

MQLWTENSPAQISDEAVFRSSPMILVQFYATLELARDLVFVLGNLGHVHFRDLNSRLTPF

QRTFVNELRNIDTIDLNLTFLHSMMVKYETLKGDPFANLSSEESGLPSSSEMDDIKHQVE

EIHDRIRNLDDSFISLELKKMKYVENRHVISCVNDFHKSTLTDRNDDHDDFRRFEISDDE

EALLRDDSQRPSFGIDEVAVTLNDYSMFNSLSGTIARDKIPILRKVLWRTLRGNLYFNEI

PISEMLPASTDRKSELIAKSVFIIYIHGEYLKTRVRKIVQSLDGTIYDNVNGSASARLET

LDDLNLKIEDLSSVVESTKSHIITELLFAQEKFFDWYLIVKRERCIYEVLNKFDEDSTRK

FLVGEGWIPKSELENTRQAIKLLVRSKVSQPHTSTDSNFINLSTDTLSTPVNGASSPGTT

ENTLFAVGDEYDASHDQGSDEEENYDFVAVVNELSTNRTPPTYHKTNKFTSGFQAIVDTY

GIATYQEVNPGLATIITFPFMFAIMFGDLGHGFIVFLVALYFIKNERFYNAQRNKDEILE

MAFNGRYIVLLMGIFSMYTGLMYNDIFSKSMTLFKSGWKWEYPEGYDFGKDGAISLTATK

IKGKTYPFGLDWVWHGAENGLLFTNSYKMKMSILMGYVHMNYSLMFSLVNFRFFKSRVDI

IGNFIPGFLFMQCIFGYLALTIIYKWCVDWLGTQRQPPGLLNMLINMFLAPGSIEDPLYT

GQKFVQVALVVVALMCVPWLLLYKPMVLRRENNRAREMGYRDINSHLDHVLQLHEEEEAL

EQVGNGHLTRDDDDNLADLGSEDEVDQLSEHFTFPNDIEPMHHNSGGHGEEEFNLMDVVI

HQVIHTIEFCLNCVSHTASYLRLWALSLAHAQLSSVLWTMTISNAFGVTGTTGIIMTVCL

FAMWFTLTVCILVMMEGTSAMLHSLRLHWVEAMSKFFEGEGYPYEPFTFDACVI

>tr|C4XXT7|C4XXT7_CLAL4 ERAD-associated E3 ubiquitin-protein ligase component OS=Clavispora lusitaniae (strain ATCC 42720) OX=306902 GN=CLUG_00759 PE=3 SV=1

MFALRLLLAVLYLASAGAVSGESAYERAQALAKNAPSRYYGMSPRYTFKNIDDGISVPQY

DTELHQYTEAPLFPLNLHPTTEERIALLEEAAQAGSVEAKVALGDIYAFGEHAVPVDYLR

ARSYYESAVADSPHGHAYFMLGFMYSTGLFGEMEMDKTKANVYYEFAAANDDLNALLVLA

YQNFQGVGRPENCALAQFYYSRAARIAMKEAHRQGLDSSLEKLSHNIRIPDFNGGIFGPK

VSESPSSVYTKVDRFLKTRDSLRDNDIDSHDSEMAEYYFEALQDYHGSYFLPKRWPNAFA

SALKCAYLGKKNVAGKEDWAVSNVDRYVYSRCMNLVGHMYVKGHGTERNLTRAYTWLTAA

AAIYANDKDALDMAYLRMYDPVYTGKLSLGCQEALLQSINNGSAHAAFIYSNYLNGVRDD

PFTPTYTSKTYSLLSSAAMAGHYASMFYFADAVESGFADSVGEQFTCTDLVSYYKMFVES

SEDTLLPHLNYAFEALTYGDYKNALLGYAIAAEQGLSNAQVSASYLLHQMEPLFSWKRKV

FDPIRVRDALRYLELASAQEDIDATILLGDIYSKGIPDANVSTDYSKAFAYYSKAASAAS

PHGCYKLGYMYEYGLGSANNTVDFFMAKRYYDLSVKYSQEYRVSKADDAKGNTYPIGIAL

LRLRLKLLLSRDKRDDYEDSSSGWFSTFKSLGKSQTTGEDNDRALEKAQAHHEGGRFDDV

EEYEIFDYMVLMFTAFIFIFMFLRNFRRQAGVVRGAHNRDNADDNAQANVNPAPGGNFQV

FFFAI

>tr|C4XXT9|C4XXT9_CLAL4 Nucleolar protein 58 OS=Clavispora lusitaniae (strain ATCC 42720) OX=306902 GN=CLUG_00761 PE=3 SV=1

MAYVLTETAAGYALLKAADKKIHKSSSLLEDLNSAEKVAEQFKIHRFEKFQSAANALEEA

NAVIEGRVSDNLKKLLEDVKLEKKATLIVSEAKLGNAINKLGLNYSVVSDAASLDLHRSI

REFLPELLPGLDDSTLKQMSLGLAHSIGRHKLKFSADKVDTMIVQAIALLDDLDKELNTY

AMRCKEWYGWHFPELAKLITDSVAYARIILTMGVRSNASETDLAEILPEEMEEQVKSAAE

VSMGTEITDVDLANIKALAEQIVDFAAYREQLSNYLSSRMKAIAPNLTSLVGELVGARLI

AHAGSLTSLAKAPASTIQILGAEKALFRALKTKHDTPKYGLLYHASLVGQASGKNKGKIA

RVLAAKAAVALRYDSLSEDRDDSGDFGMEVRAKVESRLSALEGRDLRTTSKVIRDAKKID

ITEARAYNADADATAPLPEAPVEDSDDESDDEEVTKKRKRDDDEEESSKKSKKDKKDKKE

KKEKKEKKEKKEKKEKKEKKEKKKEKKEKKDKKEKKEKK

>tr|C4XXU0|C4XXU0_CLAL4 Amino acid permease/ SLC12A domain-containing protein OS=Clavispora lusitaniae (strain ATCC 42720) OX=306902 GN=CLUG_00762 PE=3 SV=1

MPEKELNYITSRDQASSNDKNGMVYVDAFEAQDAKPPMGRWEHFVDGFRRVNEADLGIDP

NLSEVEKAAIMTANSPLSRSLKGRHLQMIAIGGAIGTGLFIGSGKVLHNGGPASVIIAYV

LIGCMIFATVHALGELAITFPVSGAFVTYSTRFIDPSWGFAMAWNYALQWLVVFPLELVA

AAIAVSYWDDKTNPAAYCAAFYVFIVVINFFGVKGYGEAEFVFSAIKVTAVVGFIILGIV

LVCGGGPKGGYIGGKYWHHPGAFNNGFKGLCSVFVTAAFAFAGTELVGLAAAESANPRKS

LPKATKQVFWRITLFYVVSLILVGCLVPYDNPDLANGDGTARYSPFVIAIKNAGISGLPS

VMNVVIMVAVLSVGNSSVFGTSRTLAALAAAGQAPKFFNYIDKRGRPLWGIITQLVFGLI

CFISASNKQGEAFDWMLAISGLSSLFTWGSICLCHVKFRMALKAQGRGTDELAFTSQVGV

YGSYFGIILVILVLIAQFWLAVWPMGAKPDAEAFFKAYLSFPIVLAFYIGHKIWKKNWRW

YIPASEVDIDTGRRELDLDLLKQEIAEEKAYLASLPFYRRAYHVVC

>tr|C4XXU3|C4XXU3_CLAL4 Geranylgeranyl transferase type-2 subunit beta OS=Clavispora lusitaniae (strain ATCC 42720) OX=306902 GN=CLUG_00765 PE=3 SV=1

MNAHWILQDTPPTMSKPFNKDLHVKYIQDLDSKIAKQSYEYWLSEHLRINGLYWGLTALA

TMDRLDALPEDEVISFVLTCFDEEKGGFAAFPGHDAHVITTLSALQILLIYNSMEVLGEE

KTKRIGDFVLSLQLPDGSFKGDEFGEIDTRFVFVSLYILTLLGRTEEKVMDSAASFILDC

KNFDGGFGMYPGAESHAAQMYTCIGALALCDRLDSVSPRTANWLSERQVLPSGGFNGRPE

KLPDVCYSWWVLSCLAMLQKAHWVSFEKLEEFILSCQDLERGGFSDRPDNQTDVFHTCFA

IAALALMFPEKYELKPIDPIFCLPKEVAQKIHRFSKQEK

>tr|C4XXU6|C4XXU6_CLAL4 beta-glucosidase OS=Clavispora lusitaniae (strain ATCC 42720) OX=306902 GN=CLUG_00768 PE=3 SV=1

MTILEKVNLTTGTGFASGPCSGNTGSVPRLGIPSFCLQDGPNGVHATDFVTHYPSGLATG

STFNKALMYLKGKAIGREFRGKNVHMALAPVVGPIGLKAQGGRNWESFGADPYLQGVGGG

QMVKGMQEEGVVAVVKHFIANEQEYFRQEGEHPADIVASISSDIDERTLHEVFMWPFADV

VRAGCGGIVCSFNRVNGTYSCENSYLLGKMLKEELGFQGFVVSDWGAQHSGVNSALAGLD

MSMPGDIYDEWLEGKSFWGSSLTKAVYNNSVSQERLDDMASRILAPFFASAGLYLPMENE

VPNFSSWTLDTYGPQFPIQKTGPIVQQNKHVDVRSTFGDDIALGLAREAIILLKNEGNSL

PIRKNIRKMLVAGLGAGQDPDGLNCRDGTCSNGFLTSGWGSAAVRNALTITPFQAIAQKA

HSRGILVEYNHNNWNLEAIDEMQEVDMAVVVVNAASGEGHIEIDGNYGDRKNISLWHNGD

ELIKHVAEKCSRTVVIVNAVGPVDMEPWINHKNVVAVLFTAPLGQFGGVALAEVLFGEVN

PSGKLPFTIAKKKSHYVPLVTESEDEEPHDDMKRGFYLDYRYFDKHSISPRFCFGHGLSY

SEFVFSDLRIKEKNQPLQFLSPGKNYLPVTSRNKEDNRDPEEALFPENLQRQPGFIYPYL

EEESLSDSDEEDFSYPPGYSTTHQSTPSTAGGGLGGNPSLWTQVYDISARVTNIGRYGGA

YSVQLYIEFPNSEFISPPKILRGFDKVYLEPHSNGTVHFPILQRDLSVWNTEKAQWVIQK

GVYRISIASSSEKVELYGEIDLSG

>tr|C4XXU9|C4XXU9_CLAL4 Amine oxidase OS=Clavispora lusitaniae (strain ATCC 42720) OX=306902 GN=CLUG_00771 PE=3 SV=1

MSPSIHPFDGIYDHEIKIASKLIKDAHAASDNVHFIQMDRLDPPKKDMIKYLEAERYGAP

LPSIPRVLYVYYYTNTLEFNKALVNVTVGHIITKARLPHGVVGPFLPEDVQEWEDYCLTH

PAVKAEIEKLKLPKGYTVRNDPWIYATDDPNEKRPLVQFFMYVLANNGHSESNHYSLPLK

FSPVFEQHTKKFVRIDFLPGGFDETTTPTGPWHVVPGVEYHPDLNGETVLRNPKPLLVQQ

PEGPSFDMSGNKITWQGWEFRVAPSVREGFAVYDGWFKGRQVFYRISLSEMTVPYGDPRA

PYHRKQAFDLGDCGFGVNGNKLGLGCDCLGVIKYLDTRTTNGTGEPKVLPSTICLHEQDA

GILYKHVNYRTENAVVARKREFVVQTIATVANYEYAVNLKFVNDGSIDIEVRATGILSTM

PIDENVTVPWGTIVGPNVMAAYHQHILSFRIDPAVDGHKNTVCYDDVVKMPRSKLNPYGV

GFVTERTYLDKAGSVEQSPFTNRSYKIINENVVNPTTKGNVGYKVVMPARQMILADEDSF

NVKRAKFATKQLWVTKYQDNQLFAAGEFTNQSQTDTGVGEWVKGEDPVRNEDVVVWATLG

FTHIPRAEDFPVMPVEIHNIHLVPFNFFERNPALDIPQATNSFNKSVLAQVGDDVAPSCC

KSTL

>tr|C4XXV3|C4XXV3_CLAL4 Pre-mRNA processing factor 4 (PRP4)-like domain-containing protein OS=Clavispora lusitaniae (strain ATCC 42720) OX=306902 GN=CLUG_00775 PE=4 SV=1

MAQTFAPVVNDRGKTDRQISLLIPTDDQEVRSELRKLGEPITLFGEGKAERRERLVRLVS

ERKHTNFEFAEVQDDSMDSDEEDEGEEEEEFYTPGDDALLEARKDILAYSIDRAMQRVDK

QRKVSASYNFAQVLRHRRNINSQLKQFEPQGSFTVKGNTRALNGVRINSSNSLVACSSWD

GHVTIFKRDTQESISQAVRSKPGYHTEKATLAWSPSESTTLASAGAEGTLNIWRVDDGSL

TNTYRRDNAHSGRIARTEFHPSGKYLATTSFDLTWKLWDVLRPEKEILEQEGHAKEVFAC

SFHPDGSLLATGGLDAIGHIWDLRSGRTIATLEGHIQGIYSMDWSPNGYHLASGSADCSV

KIWDMRKAHGAELFSIPAHTKIVSDVRFVQNTHSSPLAQPVADENEENTQTLDVSGTYLA

TSSYDGTVKLWSSDNWLHVKSLVGHTDKVMSVDVGKDASCIVSCGWDRTIRLWGKV

>tr|C4XXV6|C4XXV6_CLAL4 Protein transport protein SEC23 OS=Clavispora lusitaniae (strain ATCC 42720) OX=306902 GN=CLUG_00779 PE=3 SV=1

MDFEEAEDINGVRFAWNSFPSTKAEASKIVVPTGVLYTPMKYREDLPIAAYDPCYCQNSQ

CRSILNPYCSVDPTGFWICPLCGTRSQLPPHYQNITQENLPIELSPLSSTIEYITAKPVQ

QPPIFCFVVDLCQDEDNLKALKETLVISLSYLPPNALVGLITYGTMVQVYDLAAENVNRS

YIFRGDKEYTDKQVTEMLNKPVVVQPGQMNVPNSLHRFFLPLEEVEYQLTTILENLSADP

WPVAHGDRPLRCTGSAINVATTLLANTFSGFGARIFLFSAGSCTLNPGMIVSNKLREPIR

SHSDIDKDNAKHYKKAVKFYDAVAAKAVKNSHTVDLFGGCLDQVGFLEMKSLCSKTGGVL

LLSDAFTTSIFKQSFLKLFSKDSEDYLLMGFNATLDIKCSRELKVSGLIGHASSLGVKTP

NVSENEVGVGGSSQYRICSLSPQHTYAIFFDVANVSPLPPNAQSYIQFITHYQHSSGTYR

MRVTTVSNILTGDEQILAQSFDQEAAAVLMARVTLFKSEQDDGADVLRWIDRMLIKLCQK

FADYRKDVEESFRLHPQFALYPQFIYYLRRSQFLQVFNNSPDETAFYRHILMTEDTNNSL

IMIQPTLTSFGLDLEEPEAVLLDSVSIKDDRILLLDTFFHILIFHGRTIAQWRQAGYQDQ

EEYADFKRLLEDPKLEAGELLIDRFPLPRFIDTEEGGSQARFLYSKLNPSTSYNSQDISK

GAVVLTDDVSLQVFMSHLQKLVVSSSN

>tr|C4XXV7|C4XXV7_CLAL4 Casein kinase II subunit beta OS=Clavispora lusitaniae (strain ATCC 42720) OX=306902 GN=CLUG_00780 PE=3 SV=1

MSSDPEDDYVPWIQQFCEAFGHDYFVPVAQEFIDDDFNLTGLSSQVPYYREALYTILDYQ

VETADGNNSSAAAGASGGSSGGKRGDLPNKALLSHSAELLYGLIHARYITSKPGLTAMAS

KFERSEFGICPRYYCDGMHLIPVGATDIPGQETVRLYCPCCSDIYLPSSSRYLNIDGAFF

GTSFPGLLVKMFPEIDNQCRLRINRVNQDNFGLRLFGFKIHESSASGPRMKWLRQMPETE

QEKAEFDSCELSLPEEDTNLELEAASEEADDDDKTMASDGN

>tr|C4XXV9|C4XXV9_CLAL4 Protein HIR OS=Clavispora lusitaniae (strain ATCC 42720) OX=306902 GN=CLUG_00782 PE=3 SV=1

MSSLQRRMETQSTHELERPLASMTRHNGVVTCVKFSPDGRFLASASDDKIVLIWEKDDDQ

NRPKQFGETEADLEHWTVRKRLVAHDNDVQDISWSPDGALLISVGLDRSIIIWSGTTFER

IKRYDIHQSMVKGIVFDPANKFFVTASDDRTVRIFRYYRKFNDVSSSNYEFQMEQIIMDP

FKKSPLTSYFRRMSWSPDGQHIAVPNATNGPVTSVVIINRGNWATDVSLIGHEAPCEVCS

FSPRIFRLDKSAKTEKLTTILASGGQDRTLAIWSTALTRPLVVAEDIVQNSITDICWSPD

GESLFLSALDGSITCVAFDKGELGEVVSEDVNDSQLVRYCGTRDSAIFPESAEQLLLESI

MDSTESASSQKIHSTQGEPSQKVNTSSITKDAAKQSLNHVEGTSAKPNDTSENSATLSKH

ASNEKSFNLIEARQSPKPTLLSQKVKIMKNGKKRVAPLLVSQLSGSSKSSSSIPSSTKVS

KQFDIASKMSQTSYFLPRLGLQTSVHGLRSRAIKQDDEDKNRDQDNDNEDMGFDESHASR

MQAISTASVRRKIRKYKKFLMESRYPTPFKMVSLLPEVLYKDQAVMNNELHKIIHIKDMQ

VSTNSDLISTAALDSINENLLFRVIVNSIEHMGKPFTDLLEHEESHQNGMDLDEKNDYVT

SIVEIRNGSEWAEDDESMNMDVTQRVDFQDPTQVIVTNDEKASRKSYILYFPFKIQQVVP

IIFEGKLAFYVLVSFHGTVQIIRAESGIYVTPSMEMGSNVVVAKQNGAYLLLLTSSGLLF

SWKFPNYGKGQRMIEAVLKGVSVAPALNCETALPETSEHVEKKNISVPHVSVENLRALEV

DERTGMPYIILENSSTVYSYCASMMIWTKVMDSWHYLCINEQKNSGDLFPNTASVVMEKS

FNDFQEKVQKGARSKYTFNESNQELQESMAYRLRESIEFINA

>tr|C4XXW1|C4XXW1_CLAL4 Anaphase-promoting complex subunit 4-like WD40 domain-containing protein OS=Clavispora lusitaniae (strain ATCC 42720) OX=306902 GN=CLUG_00784 PE=3 SV=1

MDASRQGSPTPSHRAPKYPPSTETPRSPSRSTRNLSPPKLGEAQSYPSVSPRRRLNGRSL

FSDRHIPNRTGVDLQAAFSLSAQDVMPELRSARSGAPERDERAGNEIELRKEAEANRTFS

SVLKAELFGDNVPMAAAELARPKAVSPGRTNSSDVPSTSANNIRSANSLPVTIASGRTTP

PQPAGGAGGSGTPRQSANLFTYQSPTKSRPVSRDLSHELFSLSPVRSDSQRFLLSPQKKT

RSISKVPYRVLDAPELSDDFYLNLVDWGSQDVLAVGLGDSVYLWDGSTQSVERLCVLENK

DKVTSLSWIASGTHLAVGTSKGLVEIWDATKIKCVRTMTGHKLRVSALAWNEHILSSGSR

DRTIYNRDVRVQSHYINSFDSHKQEICGLKWNVEENKLASGGNDNNIFVWDGLDTKPLHK

FSEHSAAVKALAWSPHQRGILASGGGTADKTIKVWNTLTGTRINNVETGSQVCNLIWSKN

SNELVSTHGYSRNQIIVWKYPSMQQVAQLTGHTYRVLYLSLSPDGETIVTGAGDETLRFW

NVFEKNRNDEPPSSVLLEAFSQLR

>tr|C4XXW3|C4XXW3_CLAL4 NADH-ubiquinone oxidoreductase subunit OS=Clavispora lusitaniae (strain ATCC 42720) OX=306902 GN=CLUG_00786 PE=3 SV=1

MFRYVPKRLGSYAPMKMSTRTSIISVHRDTKEDNQQMPFEFTAENLKRAKEIIAKYPPQY

KKGACMPLLDLGQRQIGFTSISVMNYVAKMLDMPPMRVYEVATFYTMYMRHPMGKYNIQV

CTTTPCQLCGSDEIMKAVTDFLKIKPGQTTPDKLFTLQEVECLGACVNAPMLAVGDDYHE

DLTPEKTVELLQKLKDGKPVEKAGPVSGRHSCEPLSGRKVLKAAEPTDIRKFTRADL

>tr|C4XXW4|C4XXW4_CLAL4 Cystathionine beta-lyase OS=Clavispora lusitaniae (strain ATCC 42720) OX=306902 GN=CLUG_00787 PE=3 SV=1

MSKKYSIETELVWTKSGDQHNASVPPLYQSATFKQDSLSNMGEYDYTRSGNPTRTFLQNH

MAKIMKATQTFAVTSGMGCLDVIVRLLRPGDEVIAGDDLYGGTHRLLTYMHKKGDLTVNH

YDTTNTELLKSKITPRTKMIFLESPTNPLIKVCDVRAITEHAHAVNPDLIVVFDNTMMTP

LLMTPLDLGVDIHYESATKYLNGHHDIMAGIIATRDPALAERIYFVINSTGCGLSPFDSW

LLSRGLRTLAVRVERQQQNCVKIAEFLHQAGFKVRYPGLRSHPQYALHQSQCTGAGAVLS

FETGSIEISEKIVEATEIFGIAVSFGCVNSLISMPCKMSHASIDAKTREERDFPEDLIRL

CIGIENVDDLINDLARALTKSGAVRINERDELYNAQPKL

>tr|C4XXW5|C4XXW5_CLAL4 Replication protein A subunit OS=Clavispora lusitaniae (strain ATCC 42720) OX=306902 GN=CLUG_00788 PE=3 SV=1

MAEQYGVVPGTLKEALSRSTCHLWHGKGLRVQVTNSKVINESSATERRYRCVLSDGRYSV

QGLIDSRCNAYLEANGFTRYSIITINQYTIAKTMKRILLIADLTVDVPKAERIASDVTSL

DQYFDQNPAEDIATGVVSNGTPQQTTPQPQAPAAQSKRSAAPERPVNPIESLSPYQNNWT

IKGRVSYKSDVRTWSNAKGEGKLFNVNFLDESDEIRATAFNDMADKFYNLLQEGKVYYVT

KARIQQAKPKFSHLSHPYELSLDKDTEITECFDTSDVPKINMNFTKLNQIQSAEKDTIID

VIGVIQNVNDVFQITAKSTGKPFDRRNITIVDDSNFAIDLGLWNATAVDFNIPVGSVIAV

KGAKVQDFGGRSLSLTPSGSLLSNPDIPEAYQLKGWYDSKGSSENFQSLKTETGSSTDSL

KDRKTIALAEQLDLGTKEKPDYFSIKATINYFKTDNFCYPACNNEVNTNGRGPSPCNRKV

IQESDGSWRCERCNLTFAEPHYRYILNCSIMDHTGQIWATFFDEEGKKLFNKTAGELMAL

KEMEEGENHEFMELVSSVTMKEFNFRLRARQESFNGNMRVRYNVVSIADVDFNAESQHLA

KQLESVL

>tr|C4XXW8|C4XXW8_CLAL4 FeS cluster biogenesis domain-containing protein OS=Clavispora lusitaniae (strain ATCC 42720) OX=306902 GN=CLUG_00791 PE=3 SV=1

MLRSRFFSPTSTFVRYASQQGNKRFMQTIPGTSKGVNVDFGRLNYKLEPQISSEKRPQGN

RSSRWAAHGLATPAMPREAPKNNTDEVITIAMPPARKSRASRFKNVESKEQKTEQKVAQV

APEQVQTTPEPPAASAAEEQPKVEQTTKAKKPRRQLRPRKALITLSPGAVEHLRALLDQP

NPQLIRIGVRNRGCSGLTYHLEYVDKPGKFDELVEQDGVQVLIDSKALFSIVGSEMDWID

DKLSSRFIFKNPNSKGTCGCGESFMV

>tr|C4XXX5|C4XXX5_CLAL4 Structural maintenance of chromosomes protein OS=Clavispora lusitaniae (strain ATCC 42720) OX=306902 GN=CLUG_00798 PE=3 SV=1

MVNNPLSFLSQDKAREFIASSTEQSRFNYFSEGTNIQSILRNYQEASRNILSLQNRSAAA

KDYYEQACKKYAESERAYKKYKHSHTLRQQLEKINGKIFWYNVEVLERRIRKKESEIKEM

EDELSNLEKKKEQFATEIERDQEQASLIDKEASLLREKLDAHLLDLSKSEVEHAEATKQI

HAYRADLLNYKREIEEFGKTIEKYKNEIEFEQKKIDDANGGSKEKMAERLEMLQKRQQML

IENRDELQEQLHTISGTSPEMESLTQQLEEHRIAKAAIQKKISDFERNKNNKYAAFGHNI

AYLMRDIEKETRWHSKPIGPVGCFVSVKEGYDKWTDLINATLQKTLDSFVVCDEHDRRLL

NQYMRGKKIFKNIIVRKFEAFAYNGYAPEGCATVLDTLDIHNDDVKFTLIDSSGVEEMVI

CESMREAESITQNRNVKHAFCLKDRSSGVRVSRRDGQLSRDPIFYSNDLRRLAGKELSHD

FQGSLEELNHEEARIHQKRNKLKAEEQAKKKNLQTELEKKKKFLKEINNQIFNLERLLSE

EGDHGKIDSLKEQIIHSENQIKTREGMSMELLQNLNESKKEIKEMAEKLQGMNATKQKLT

DEITEMQNNVEKYNQNVQLLEAEIEGHSKKQEEISIEIRAKLNKKESDEKKYDELKSAAE

EHCSREEIEIADSDTTESITNEFRAIQNAIEEAERNTNKTFEEIQNEVLQSKELKDKCET

SLNDLEHARVTLENDLNSRFDNLNITIKEKLTRAKSAFEQCLALRGFKGKLEFDFGQKRV

VTEVQTKDDKDTRAVSSLSGGEKSFTQIAFLLSIWKVMKPRVCGLDEFDVFMDSVNRTIA

IRLLIHELQSSNAQSIFITPQDIAVVGDLKDSDDVRIHRIKAPRDD

>tr|C4XXX7|C4XXX7_CLAL4 Membrane insertase YidC/Oxa/ALB C-terminal domain-containing protein OS=Clavispora lusitaniae (strain ATCC 42720) OX=306902 GN=CLUG_00800 PE=3 SV=1

MLKLGARSSRLRPLRAFSPFVRFNSTSEAQNSVVSEIKTSLPSFENTSLPETLMTSDQIG

YLQSIGLADGYGPTALIERMLEYTHVYSGLPWWGTIIAGTFIARAFMFPLYVKSSANMAK

MARVKPELDQLMNDIKTGDNEDRMLAMRKRTKLYKENGIKTAHSLLPMAQLPLAYGFFQA

TRKMAAYPVEGFSTQGAYWFQDLTQVDPYLGLQILTALVVTGMMRSGGETGAQAMNPMMK

KVMTWLPFASILITQSMSSAVVLYFAANSVFSFFQSLVLKNKYFRKFAGIPPIVKPVVVP

GAKAPPATLGEWWNDFNSRMKQQSHTKMAKTNKQLEAIEKRRDAANEGFIKRH

>tr|C4XXY1|C4XXY1_CLAL4 Inositol-pentakisphosphate 2-kinase OS=Clavispora lusitaniae (strain ATCC 42720) OX=306902 GN=CLUG_00804 PE=3 SV=1

MDTTRLLADEWKYFAKGNANLLFKYVGPEESLRNYLLRLRMKKDAGTYIPTLSITEFIDT

KCKALFPTEIISTKLVELDDTFLSSLDTNGYDLMPGEKYGLLLPNMLAQCNDHLTLSKHC

SLHLREELGIVKSAVIELKPKWLYDNLDNYCRTCSILQSKGHERHFCTLDLLSTETVARG

VKDLLGKLPDAIKASLKNSDFPIESLLIEYFGGSESILQKLKAKQDSLDDGTILANVKSE

SDVSDQLALRMTLRDVGVFVVIERGQCDGQVINIRDHGEFTVAARIYDLDLKPKARYKHW

VSTELKLGDVYNSSNSDWPHCVKKTESQK

>tr|C4XXY3|C4XXY3_CLAL4 MAGE domain-containing protein OS=Clavispora lusitaniae (strain ATCC 42720) OX=306902 GN=CLUG_00806 PE=4 SV=1

MSKRRRTEIEDDGDYTEENRTPLAEDSATRESSPSAEDELRSSAIVSILLRMVMTRSARN

QKIRREHFTAILHKHNVKGNTKAIISQVDAELKEVFGLKLDQSTNDIVLVSNLQKDTKLL

LQKLVENGDGSEFKGNPYDTFYFMNKEKRNETTVSMSDSIFGGIIMLLIGLVIVNENRIR

ESDLLDGLSQFGFSENLNIVVPNLNKTTQEVLTEATKKEYLQKLVAQAHGGEPAIVSYAL

GKRTLREFDAHTLLEFLNEIVEQEELKPKCIETVKRCFPDDTFENSASGNNVQS

>tr|C4XXZ4|C4XXZ4_CLAL4 PH domain-containing protein OS=Clavispora lusitaniae (strain ATCC 42720) OX=306902 GN=CLUG_00817 PE=3 SV=1

MSSHLSASLIRLKLLDCLRAGDIKKVESLIEDLQSVQSTIETTELIRLRETVLHYAVQVA

PLATIQALVESKKFSLDVNSQDMDGNTPLHLAVAAGRLNVVKYLLSLPNINDTVLNADRK

QPVELSKDTDIAQLMQYERAKFVERAATDLRRMFSNRDFAGLENILVNNPRAAELLDING

ADPETGNTVLHEFIRKEDFEMCDWILKHGGDPFKRDKRGKLPIDLVSSKADPIRRLLKVA

SRDQNMMDPVASTNNAIKAGNAPTYKGYLRKWTNIASGYKLRYFVLDSSGIVSYYTNQGD

TINACRGSLNLGFATLHLDSSEKLKFEIIGKNGIKWHLKANHPVETNRWVWTLQNAITLA

KDNMKRKQNASRRSVAEDAPKALEDTEDERRRSSLLHVPGRRKHKKSPSQVSLSSFTPSD

AEDSASITSEPRSTSAKSNTHGSLSQINERKAVQNDRSSMEGLHYGSEDFDYDLEDSGSD

YDGDSSADEPADQLAKYSMDQIQSTKRAIKVELASLISLFKDLGANATSDGNDETFNVGM

KTLSNVQDLIDQLDTYNSIRDQKLLKKIDRQQEVNKLWESSIRQLENEIQKREEALALYD

GKKYKLKKLLESKGMSPAHVSNEQATGLMNSQSITNEESLAKTETKTDQSQLFKEIFNDS

EDEFFDADDFDEDVEESASAKAGETTVEENPTTETTVDKSTDKSESQNTLTNPDETKKIE

PVATEDEAASGKTATKGTESISEGGQKAVTDSQMKSLAKLKEEGSFLGYEKPPRKKLAMD

EDDRPKIGLWGILKSMIGKDMTHMSLPVSFNECTSLLQRLAEDIEYNDLLNKAASIPDST

LRIVYVATFAASEYASTIDRIAKPFNPLLGETFEYSRPDQSFRLLTEQVSHHPPISACHA

ESVKWDYYGENAVDTQFRGRSFDVKHLGKMFCKIRPDNGVVDKEGHKVQEELYSWKKVNT

SVVGIIVGNPTVDNFGQMEVRNHTTGDVLIVDMKQRGWKASSAYQLSGIASDAHGKERWA

IGGRWNSKIYAKKITGLSEKKRRNSLIDAEGEHQTSDPYSGRKFLVWQAAPRPKIPFNLT

SFAVTLNEINDNIKPWLAPTDTRLRPDQRAMEDGRYDEASREKHRVEEKQRAARKRREQK

REIYKPNWFVRSNHPVTGDQYWHYNDKYWPKRKNKELAGVADIF

>tr|C4XXZ6|C4XXZ6_CLAL4 Myotubularin phosphatase domain-containing protein OS=Clavispora lusitaniae (strain ATCC 42720) OX=306902 GN=CLUG_00819 PE=3 SV=1

MDPLKAITFENVLLSRRGHYLKGSLILSDFHMVFSFAQEKDTKPREIWLCYPVIGRMCKA

RRSSWSVSPVASMTGAKLPLIDPPTDTDFDRYSASHIRVYCKDFTYYSFDFVNDSTCSEV

YHKLSLLITSPKFKGSIKEFYAYDYKPNMLESRLESSGWSLYSAEDEYRRQDLISEDSET

QPWRLTSINETYRFCPSYPRTWVVPSSISDSILKHAGKFRSKQRLPAVVYKHRVGENGNV

IARCSQPLVGLNLQNRSIQDEKLVEEIFRSQQLERAARLSNDSEFSEQPQRNLIVDLRPL

TNALAQHALGAGTENVDNYKGRQSHGTNSQDSESGNVRHFQNVDKIFCNIDNIHVMRDSV

NKLISILSDLDRYPVSSNGDTMYPLLQQSLTKTQWLHRLSVILQSVDRITKSVHLNNTNV

LIHCSDGWDRTSQVSALSQLCLDPYFRTMKGFMVLIEKEWISFGFKFATRADHGGCIGAL

IPRSQTDSQDSPNDSESISSGKQNSPSESTDLPPSKTYSNGGTKEAVTSFLQRAATHIKN

TASAAASSASQKSLDSSGEQSNYMVYKGSNECSPVFHQFLECVYQIMRQHPTKFEFNSRF

LKRLFYHYYSCQYGTFLFDNEMSLSLHQAHKETVSLWDYFLSRPTEFTNASYHKPSNDDV

LFINFSDVKWWFELYGRTDEEMNGLSNSLDKKFAQLGLNHSSSEVSTQESTT

>tr|C4XY01|C4XY01_CLAL4 Cell wall mannoprotein OS=Clavispora lusitaniae (strain ATCC 42720) OX=306902 GN=CLUG_00824 PE=3 SV=1

MKFQSVALVSILASLASSAPVSPSNTWSTLTPTATLPKSASAITSTSGTFALSIRTVSAS

VSSGISVNKRDVISQISDGQVQADTKSATATASVINQISDGQVQENTKTTKKPEPTASVI

NQISDGQIQEQTKKTTASVINQIGDGQIQQQTKAKTTASVINQIGDGQIQQQTKAKPTPS

ASVINQISDGQIQQQTKTKTQKNTASAVAQISDGQIQEQTKTKTQKNTASAVAQISDGQP

QQHTSAPVASQVSDGQVQATSSPDEATVEETCYDSDALTIQLKDGELRDSKGRVGAIVAN

RQFQFDGPPPQAGTIYAAGWSFVPASYAGVNEKSSDTTDEGLKLALGKQTVFYKCLSGDF

YNLYDESIGDQCSAIEIFVLEAVQC

>tr|C4XY04|C4XY04_CLAL4 U3 small nucleolar ribonucleoprotein protein MPP10 OS=Clavispora lusitaniae (strain ATCC 42720) OX=306902 GN=CLUG_00827 PE=3 SV=1

MCGPASSGSRCSSTSTLRFFSTTRSSRIKNSLVYNHRRRYSSVMEFLKSLAENPANIFKL

AQKEETVEQLNTITKSLLDPIAKEHSVLDEIYVDGLDATQVFGQAQMVLSGVGEALLFEK

IPELKEKYGTTEVNSEDDDGAELNEDEENLEDKLSGDEENLDDDSEEEFFSTEEPEDFTA

QELEHSEDEENDNEEANDSEASEEENVDASDAESETTKVASDNESSGPKKDVFGLNDEFF

DIDNFNKQIVALEDDGDDDEEEIDLFADLSDAEESEEQEMDYYDEFFDKPGQEKKTAKKS

KKAKSEEDEELDEDRELDDKEYDQAVDSAMFDLFADEDEEVEKQQNTKQMSSFEKKQSQL

QAEIAKLEAELVAEKKWTMKGEVRSKDRPMDSLLDDEEAPLLEFDRTSKPVPAITEEVTE

SIEDLIKRRIRNDEFDDLPKRLITDVSRFVQKQKTEVSEQKSEKSLAEIYEDVYHGVDPD

EKITEELQAAHDEISDLMTSLNYKLDSLCSAHYIPKPHQFKSLEVKVTDAAASISLEDVQ

PTHVSDETKLAPQEVYKIGDDKVKADGAKGVSQVQLKSGLAYSKDELDRDDKQRLRRAKK

RAKSKHFNELKEMREAREKQKPEGGDRKRQKVSDVIDTLSKAKNVTVIDKKGRLTDAKGN

AKKNQSAPSGSNLLL

>tr|C4XY08|C4XY08_CLAL4 glucan endo-1,3-beta-D-glucosidase OS=Clavispora lusitaniae (strain ATCC 42720) OX=306902 GN=CLUG_00831 PE=3 SV=1

MSIFKLLALGCSIAGVLAAESSSTQRNSSTANGNAVDKIVFENVGYTGYYYDVESYTDLT

SDDCSCKLDKSFTVFEGTNAPLNEELSVHLRGPISLKGFGYYVSDDSSKGTWQRKAYYNS

SSQTADNVTFLVNGGEFSPCVGRALTYASSNGTGIASSATILEDDNLVESNEEYTIMSSV

KCASSGASNDCGYYREGIAAYHGFYGDIKMFLFEFEMPEATDDKDTTYHNMPAIWFLNAK

IPRTSQYNANTSCSCWASGCGELDIFEVLNSTKPLQLDSTIHDFQGTDDIQNGLEATDYF

ERDTSGVMRGGVVFGNDRTITVFMDDSMVIDESVAASDVQKWLGSASSEGVKSLSSASMD

TSTTSASAAASGSSSSKKSDGTSSSPTSLWFSAVTLVLSSLLYL

>tr|C4XY11|C4XY11_CLAL4 separase OS=Clavispora lusitaniae (strain ATCC 42720) OX=306902 GN=CLUG_00834 PE=4 SV=1

MADLSTRDSLLHTLSSHVGRYSNPLSLRSSNSNSYRLPKSELLDQKCLHIINSPSDPELV

SLAYRYLYLQASSNIRDLISQHQRYIVQLSSSKKHTLVLQELWTVASVLQGSRKLGQFSL

SSWMCLVDGLSPQNVEPSLASFVVSHHFLVLQSILQYLSASLQSVVKGSSAMTLSVFEKV

AQSFLSDSNHQMCISKFAPNTTAVQKYERNNAKVAAGFLKVAKFLRSKLHANETLTMGIT

LLELKTCELKMRIGQFDDTSLPDLTSASHITPFIDDLKSAISLHPLPVPKLESSINSYLV

SRPRSSSLNSIHGILSQLHFVEDHDLLSKLQKLLSITSPTEACEALNVSLSSLVSENPNR

TSAFRSLGVVLRFLFSKIDKIEKCQAFINISDTIISKIRTQSADTSFNVYVTEVLSPLST

ILFQMKQTKRVKQITKLCYEASHSPEALKLSAQLDLSTTDKDISDPSKLQNRIRHLISQL

CSFDDLEDALDVLDKYTEYCPGHDIVPLVIIPIVDSLFQTTKSFQAVLDPVSKLNPNLKT

QLISLMIQDLDRRGSEKALRIQSFVQQFSDENTPNFLHLYKAASLADTLSIPNLNPSTPF

EALCTAGIYIFCLSRGAGSYEEILKSAKNYLECWLNSEENTQQSSEIEVFNEIMFELFHL

SHFQLVQSLTENFLLVTKATTYAVLQLELLLCRSMLETTNISEVPSVLKRAGSLMKGLNT

SDRGISYSQVAEWKLLQFDYFISMKDIGKSSEKYQEILQFLDTKPGFNLTESSHKLSFNQ

KLGNFLIVARFSILTSKMNSLNGDFVTSLRNAKLAIKILNSILRKINMSAELKEIREDTN

FFISQAYFSAFEASRHLGLSKDAMYYTNELRDFNSCCKYPIFKAFYHFALAVFYSFAGRA

EPSDEEFMSGQSIATQIACSATDTCMALSSKLITLFSGDGTFDDDNLLSDALERFEKSSN

LLIGLSSKYITKAALGLDYIVFFNFSKHPSISIDNDRRKMLLKTTLEVKEKLQTLEKALF

ADCGTNIRWTAKILPSATYSTCDFKDISNNLSFCKEILLRIAQEDDQLMYLNVSLTRYVS

SLLSCCVFMLSFVATMKEEFAFDLLNTVTSLSDFAKALPSANQKLLISKDNTKKNNDLMP

SKGEANELLTLNDKTINMKLQKCLPDSCVVVSLDICELSGNLIITKFESGQTLPFVFKLP

FRKNNGSFKSFADISACLQRIIEDSNGSTKKSVTTSVKTKEDRRNWWKMRFELDQRLKNL

LDDVEDELLKGFKGVFFFTDRQSQEYNAFRNRLNNIWSSFLQSIDNEQHASLQLNQGLVD

LYYNLQFSGHEKHYADIRDLVTYTFTELTGSNDFARVSSTMTTTLLSSIQGLYSFAATSK

NEHIFLIPSNACASFPWESMECLRGRSVSRMPSVFQLIDLLSLHPGFSFPNTEPENVFYL

VNPGKDLPKTEAEFGPLLQSMPNASGLCGEKPEEEHMLEQLYSSNLFLYFGHGGGEQYVR

ASKMIKRKTENNTNTLPPALLMGCSSGAFQDNGNLEATSNIFSWLSCGSPLVVSNLWDIT

DKDIDRFSMSVLYKWGFFTESDKQPAACEDICKAVANSRNLCTLRYLNGAAPIVYGLPFS

YR

>tr|C4XY18|C4XY18_CLAL4 Upf1 domain-containing protein OS=Clavispora lusitaniae (strain ATCC 42720) OX=306902 GN=CLUG_00841 PE=3 SV=1

MRTPMRTLSTFHQISAPTMGSTTTSSDEHETRAAAGTPVLDPSVPIDGGAPSQPLSLTDE

TEHLTAYQHGNGNESEKDGEHDHEAEPTEACSYCSTSDPASLARCGTCQRWFCNGSSSKA

GSHIVAHLVHSKHSEISLHEQSALGGDPLECYNCGSRNVFALGFVAAKQESVVVLLCRMP

CAQARDINWATNEWQPLVEARALLPWVAAQREPARDVKPAQMARLEAQWRVKRDATLADV

DADADGARDLAMALLRYPDALEYQHVWAPLVEAEAACDKFMKESRVLDHLSVTWGTVASG

RHTASFIVSTYETSELHVAVGDEVVLHHRDFGAAEWSGAGVLVAVPGARTDIYTVELQPS

VVPPQATTGFSAEIVWKGTPYQRMQHALFRFATSNESVSAYVYHKILGHDVVEVEFDGEN

SLNNKRCSALNASQRAAVAHAIASPLTLIQGPPGTGKTVTSAAIVRELVRLRRSRVLVCA

PSNVAVDHLALKLRAAGLKVVRLAARSREDIESEATPLALHTQVRAAIPRRVRTLVDKQA

SGGELDARAKARIARSWRAAEQKLISSADVICTTCVGADDRRLEEYEFPIVLVDESTQAT

EPEALIPITRGAKQVVLVGDHQQLGPVVLDPAASAAGLRRSLFERLVSMGHVPLRLEVQY

RMHPALSEFASNMFYEGSLLNGVTSDDRTRPGADFPWPVPDRPMMFWANYGKEEIGANGS

SYLNRVEAMNVDKIIARLVRDGVSPDQIGVITPYEGQRVYIWQYLKLNSTVPKSALNELE

VEVSSVDAFQGREKDYIILSCVRANEDRDIGFLKDSRRLNVALTRAKFGLIILGNPRSLS

KNKLWNSLLVHYRERGCLVEGPLDNLNLSLVPLGQTSNQEKSFISGPRNTDFDTQSMISH

VVDGDDPEFHYDAPQESPLHDDVVWPSLSNNAASRLQEKKSQPSAYPKIAEDDLKAFASA

FASGLNL

>tr|C4XY25|C4XY25_CLAL4 Fluoride export protein OS=Clavispora lusitaniae (strain ATCC 42720) OX=306902 GN=CLUG_00848 PE=3 SV=1

MKVSFWRPSLCIVFFSIWGVLARMGLVALTTYSGAFLGGVIWANCAACFIMGLAAKTNDI

WRKVSPTHGSKGAIPMYVGVTTGFCGTCSSFSSFILESFEKTANMPSEHFHYPNRAYGIM

EALAVLIAHMAISASSYVVGQHAGSLIDRIGSFSPTFYRRTEYAIHIVGLAMYGGVIALL

ATEKHGAWRPWMFSCLFTPWAALIRHAAAKLLNPRTPRFPMGTFAVNVGGTLLLAVFTIL

SRGRRRPFSNSPVIRGKMGCHVLWGLDNGFCATVTTISTFVAELYSLSLGSAYLYGLVSV

ALGFVIVLLIVGSYDWAVGFTAAVC

>tr|C4XY26|C4XY26_CLAL4 ADP/ATP translocase OS=Clavispora lusitaniae (strain ATCC 42720) OX=306902 GN=CLUG_00849 PE=3 SV=1

MAADNSFLVDFLMGGVSAAVSKTAAAPIERVKLLIQNQDEMIKQGRLARKYDGILECFKR

TAAEEGVGSFWRGNTANVIRYFPTQALNFAFKDKFKKMFGFKKEESYAKWFAGNLASGGL

AGATSLAFVYSLDYARTRLANDAKSTKAGGGERQFNGLLDVYKKTLASDGIAGLYRGFGP

SVVGIVVYRGLYFGLYDSLKPVVLVGPLEGSFLASFLLGWTVTTGASTASYPLDTVRRRM

MMTSGQAVKYNGAFDCFKKVVAAEGVASLFKGCGANILRGVAGAGVISMYDQLQVILFGK

KF

>tr|C4XY32|C4XY32_CLAL4 Pseudouridine synthase II N-terminal domain-containing protein OS=Clavispora lusitaniae (strain ATCC 42720) OX=306902 GN=CLUG_00855 PE=3 SV=1

MGIIALKSHLRMFRYLPRRMNGIFAVEKPKGVTSSKVVLQLQSIFDTSDVFARDLAESKQ

KVHEQLTKGTKWSASKIANRVKKTKIKVGHGGTLDPLASGVLIIGVGTGTKKLSYYLGEC

TKTYETRAVLGQSTTTGDSEGEVLTRTEVDHVTLDDLKKAARKFVGKSKQTPPIYSALKV

DGKPLYEYAREGIPLPKAIKARDVTVESFTVHDDFGPHPDFGPIKVVPDAEGNTVEAMLA

NNPTLNDHEIYFSDEFMADPNVSDEEKNTHIKPRLVDPNTPQPDSLPVFHATATVGSGTY

IRSLISDLGRAVGSSAFMVELVRSKQSDWELGKNVFKVTDFTERDSRIWGPVLKRVLDNG

PEVLVDEEFEKVSQTLGPLLAEEKKRAAEELKQENQDGKKDEAEVKHGNEDEEKEKTDDN

EKDVQETADLQAEKRRKIEVSAMTNEQ

>tr|C4XY33|C4XY33_CLAL4 Ras modification protein ERF4 OS=Clavispora lusitaniae (strain ATCC 42720) OX=306902 GN=CLUG_00856 PE=3 SV=1

MNDPSTKYANATQEPLTYFNYHEYLTDKYTPAHNPTKTKHRLDDASSISSHSSKQKVSLV

INHFPNPYTTKGSALYHTTRIVRIPRIFDTVEHSYMVPQFSTWAPGAEPAAIHYGKENGF

VSLASYDGTVFGPTSVTPLVPGYLSANELREIVDRLNEFLLQAMSPNKTTTFENIVEFFT

GTLYSRIFAGKAGATHLKEGIARMEEYVEQLNRNFLSKRHELLRLISPVDSAFLSLDFQI

PKPPATTEYEIRQDDTSQEDSPKV

>tr|C4XY35|C4XY35_CLAL4 Holocytochrome c-type synthase OS=Clavispora lusitaniae (strain ATCC 42720) OX=306902 GN=CLUG_00858 PE=3 SV=1

MGWFWADSPRRNISQGSAPAVCPVNHAALPASACPVKHSGSDDVEVLNPLNNMPMAISSE

RAPGQRVVLSTERTISSIPRGESNDQGFWEYPSPQQMLNAMMRKGKGQGIDEEAVESMVD

VHNFLNEGAWQQILEWEKKYTETTRVEPRLERFTGRPNDLSPRARMFLYLGKVFPQTFNT

QPPFDRHDWTVLRSMGKDQGWEKVRYVIDYYSAPDDEDTGMPAFMLDTRPALDNPRNAFD

RFDHWFTPLWKKAMGEGEYGH

>tr|C4XY41|C4XY41_CLAL4 Electron transfer flavoprotein-ubiquinone oxidoreductase OS=Clavispora lusitaniae (strain ATCC 42720) OX=306902 GN=CLUG_00864 PE=4 SV=1

MIRHTSRSFRLPGLRRAWSGRNLWPNHSATSSTRSAPRVAYRNFSVASRQHMRPTPSFRK

EIPFSQLSEEDRSLLTEERAADNVDVCIIGGGPAGLATAIKLKQLDNEEGNGDLRVVVLE

KAPDFGSHIVSGAVLEPRALKELFPDSEFLNEDGSGIPLPPDLVTLVEHDDMKYLTSEYA

FSLPEPPQMKNHGKNYIASLSSVVQYLAEQATDLGVETYPGVSVSELVYGANGAVKGVAT

RDVGIGKNGAPKSSFERGMEFHARITVLAEGCHGSLTKSAVAKFDLRQSSDPQTYGLGIK

EVWRVRDENFERGFVGHTMGYPLSAGVYGGGFQYHFGDGLVAVGLVVGLDYANPYISPYQ

EFQKMKTHPYYSNVLEGGECVSYAARALNEGGYQSIPKLHFPGGLLVGCSAGFMNVPKIK

GTHTAMKSGIVAAESIFDAIKELDAVDEETDMEENVFDLAQYEEAFKQSWAYEELYEVRN

VRPAFNLAGGPLGFLTGLAHSGLTTMITRGSEPWTLSHSHGDAEATRNAKDFSPIEYPKP

DGKLTFDILTSVSRTGTYHQDDEPCHLRIPNQDHRKHAELSWPRYKGIEQRFCPAGVYEY

VEDENEPLGVKFNINSQNCIHCKTCDIKVPDQDINWTVPEGGDGPKYYMT

>tr|C4XY42|C4XY42_CLAL4 ferric-chelate reductase (NADPH) OS=Clavispora lusitaniae (strain ATCC 42720) OX=306902 GN=CLUG_00865 PE=3 SV=1

MRNFIWLFLIQLAMAGNIIIYDKEYVFRSCNSVIWYQGSFCMPRNLSEESYEPLESYECY

CSNKNGLATLMGCLAVEGQNNDAMYKKVVKECAKYGAQVSTSDLSAAYQTYLKDAKDVYG

NKSYDMSKPVDYPIKLSDKQSVIISRDTYHVFFRNFNYSLSYGGICLAYWALMVVGAFVC

NWVVYLFPNSRLFLNGPISRTYRRYVEYPALLGKKRNVPQRFLYFFEFLIPTRAESLIVF

GFFAVCVILCAVNIDFVEGQQLYPGKQAFITRMIVDRTGIMCSMLTPLFFLFAGRNNILQ

WITGWKYSTMIIYHRWIARIAVTMAFIHSVGFTWIYSKEKYYTTAMKEAWLRWGVVATTC

GGLICFQGLLFFRRRFYETFLVLHILLAVFWVVGMWHHLKAKDYQQLVYPAIAVWGLDRV

LRVLRLFWFGMPVAQVTWLLDDTLKVEVPKPKSWPSVPGGHAWLHFGYGMYIFQSHPFTF

IDSPTKENTIVFFCKVKGGITKTLVKALHEKPGKTMPIRVTVDGLYGETSPVKHHSSIVY

VAGGSGVSGVYSEAMAMSRKLSTTSKRVRLVWILRDFVSVAGFTDEIKATRNSGLLTSVY

ITQPEATVAAKAFLERNNSSSSDEKTRIEEKVRLEEKYGPGFAQEEKQGMSQGSDNSRQD

AFGLAGLEYEFPDIDFHVGRPDLNALIARETEQAPASIAFIGCGHPILIDDLRYSIIEYM

DKTDKRIDFYEQLQVWA

>tr|C4XY45|C4XY45_CLAL4 Endoplasmic reticulum-Golgi intermediate compartment protein OS=Clavispora lusitaniae (strain ATCC 42720) OX=306902 GN=CLUG_00868 PE=3 SV=1

MSSRPRLLSLDAFAKTVEDARVKTASGGVITLVCVLIVLFLIRNEYSDYMSVVVRPELVV

NRDVNRQLDINLDITFPDVPCGVMSLDILDMTGDLHLDIVESGFEMFRVLPSGEEISDDL

PLLSGAKKFEDVCGPLTEDEISRGVPCGPCYGAVDQTDNKRCCNTCEAVRMAYAVQEWGF

FDGSNIEQCEREGYVEKMVSRINNNEGCRIKGSAKINRISGNLHFAPGVPLSRNGRHSHD

LSLWTKYSNKFSIDHKINHFSFGEDPSASRRLASTDDSQEPSIHPLDGFHFDLKKKNHVA

SYYLSVVSTRFEFLDGKKEAVDTNQFSVITHDRPIVGGRDDDHQNTMHAQGGVPGAFFHF

DISPMKIISREEYAKTWSGFILGVVSSIAGVLTVGAALDRSVWTAEQVLRGKKDM

>tr|C4XY46|C4XY46_CLAL4 Large ribosomal subunit protein mL46 OS=Clavispora lusitaniae (strain ATCC 42720) OX=306902 GN=CLUG_00869 PE=3 SV=1

MIGRAFSRSYSAPTAPAIRSTLLLSRTPVITADLPEFQKQYYRYQNELWKRLMWTFPKWF

YYREGTLSEQKFRELNKNPVYNNPNLEFVGGRPELRQQRDRRFKQELSLPKTYQENAKEE

EDTQSESLARKIVPNSRITKADEAKDLTSLERALSRTLYLVVSQDKGKSWKLPSFPNNGS

ALHTTAEEGLYSIGGDQLNYFNVSRKPCHVHNGAEGKEFFIKSHILSGQFSGQQPDLKFL

WLTKEEVGEYLDKEYFAEISHLMSEV

>tr|C4XY47|C4XY47_CLAL4 Protein NRD1 OS=Clavispora lusitaniae (strain ATCC 42720) OX=306902 GN=CLUG_00870 PE=4 SV=1

MSAVGEFESILKELSTLKAPGVSGTRIKKLTDIAVKNVSEESKIVPALYASCKATPSSHK

LGALYVVDSIVRVYMDEAKKRNEVINAQAPEGTFAAGVFKISELVESLIDDAMELSIIPS

TNIKIGKLVDIWERAQTFSPETIKKIRDKHFRSTTPPGTPPGKKADLASASVSSGADAPK

PTSDSGSILSALASLAKSNDTPTPPAAASPAPQKDAAANNATNILSQLSVLAGGSSGSAV

ASPSPPTSQTQPQQNQPNKEYIFNMLQQMQGNNGQNGPPAPNMPQMPQMPQGMPQGMPQG

MSQGMPQGMPQGMPQIPQMHQMQGMQGMQGMPGMQGMQGMQGMQGMPGMPGMQGMQGMHP

QRGGYNNGPGNFNDRRGRDDVGGYSRRNRSRSPSGRHGRGHDNYGSPHEEKKSLSQGYNA

SEGEMNVPGAPHFRPRNVGFDSTLPQGSFKVMSRTLFIGGVPRGMDEKQLASHLRPYAEV

QSVILNSERKHAFVKVYSRKEAEQVIQSFNKDGALPLRTRWGVGFGPRDCCNYQHGVSIV

PIQRLTDADRAWVVQAQWGGTGGQPLQSGLVIDEPDIEIGTGISSKAMSKKMPTNSARNG

PKSNRPGEPDEQFVKATMASGQQDFLAYGQGAANPLSGLFNNSNNNNNNNAPPPQGYPGQ

MPQNNSLPNFPPNYGGQNGNLSAQLANFFSNGQQR

>tr|C4XY48|C4XY48_CLAL4 AAA+ ATPase domain-containing protein OS=Clavispora lusitaniae (strain ATCC 42720) OX=306902 GN=CLUG_00871 PE=3 SV=1

MQSLTRIIPRAPARILRPTYIRPLALGVSAWHVRRTTYRNMSSSTMSMLSASEQAANSDI

SNAEVQAEFYKRLLATNYPQIVVQRFETPGIASNPECAQLYIEALNKVGKRAKAEQVTRD

LAAGTNAAVAGNAGHSPFSYGMGSRTEPVHVIVSESGLTILSKWLKWLIPVALLTYGASN

AFNYLVENGTIFRNSEVVDKSVDVSQSTVRFKDVCGCDEARAELEEIVDFLKDPSKFTGL

GGKLPKGVLLTGPPGTGKTLLARATAGEAGVPFFFMSGSEFDELYVGVGAKRIRELFGQA

REKAPAIIFIDELDAIGGKRNPKDQAYAKQTLNQLLVELDGFSQTEGIIIIGATNFPESL

DKALTRPGRFDKEVIVELPDVRGRVDILKHHMQNVETAENVDPTIIARGTPGLSGAELMN

LVNQAAVHASQLSAPAVDMSHFEWAKDKILMGAAKKKMVITEEARKNTAYHEAGHAIMAM

YSPAATPLYKATILPRGRALGVTFQLPEMDKVDMTKKECFSRLDVCMGGKVAEEMIHGPE

NVTSGCSSDLANATGMARAMVASYGMSDVIGPVRMSDDWESWSPKIRDLADNEVRSFLVE

SESRTRKMLAAKKTELKRLAEGLLEYETLTKEEMEKIVKGEPINKPKTISNTVIKSSGSG

RLEVINEPTQPVPLAEA

>tr|C4XY49|C4XY49_CLAL4 Condensin complex subunit 2 OS=Clavispora lusitaniae (strain ATCC 42720) OX=306902 GN=CLUG_00872 PE=3 SV=1

MTSVLPKKRSGDQKKRKSSAVLHDLSNKGRVISGRSVSNTKRKPSGTRHVSRGDMFVGRD

EEDANSSFADDAIHFHENRSTIMSNFEEWIKLSTDNKITTKNSWQFALIDYFHDMNVIKD

GDNINFQRASATLDGCVKIYSSRVESAASETGKLLSGLATKKGQIELEDAQEEGEEDGEA

EDSANSKKERRVNRIVESTLVSFDALKIKKLDQELAIDPLFKKALADFDEGGAKSLLLNT

LNIDSSGRVVFDATTNSMNPDEEHESESKQDDMDVDKKSIDFSKLRNMFVSSGPELETLS

ICPSISELKLALSDVNKAKTVLGDVNDRFNADEEEERNLADSVPPEENLPDYDYDMDFEA

QDEKREDVNLDNIENEIQKAQDVLKSYDDTQFDEGVTTATVMDQDLMAYFDEKMRTNWRG

PEHWKVTTLKQSKNLDGLSKKGQTPQPETAPKRIRKEDITINFFEEEEEDFLENIFQKHK

NPFQISKRPEECKIDDFHVLPDDIQFNSQRLVNLFLKPKKSIVTFSKRSKNHSNESNLND

KAYTDENYFAGKYLEQEKALEEEARQEKLAASFLQAEMEDYDNDNFGGIDFNDVLGGEDG

TVGGEEETKNDNGSQFVTGGRKARPEYVNFSKVAKRVDVKLLKDNLWKSIKKEENEKEKS

VSPEEQSEEDRKKTFTEVVETISEMYRPEEKKDLSTSFCFICLLHLANEHGLSIAATDAH

DDLLIKGF

>tr|C4XY51|C4XY51_CLAL4 1-(5-phosphoribosyl)-5-[(5-phosphoribosylamino)methylideneamino] imidazole-4-carboxamide isomerase OS=Clavispora lusitaniae (strain ATCC 42720) OX=306902 GN=CLUG_00874 PE=3 SV=1

MTRFRGCIDIHAGQVKQIVGGTLTQDDTQASKDTKENFVSTKPASYYADLYKQNSVEGCH

VIKLGSNPANDEAARLACIIWPGHLQVGGGINDSNALQWLDQHKASHVIVTSWLFSQDDG

RTVFDWAKLESLSTLVGRDRLVVDLSCRKVSESKWVVAINKWQTLTDSELSAEFLARAAK

YCAEFLVHAADVEGLCNGVDEELVTKLGEWCPPRFEGRIVYAGGARSITDLDLVARLSGG

KVDLTYGSALDIFGGSLVRFEDVVRYNRA

>tr|C4XY52|C4XY52_CLAL4 DNA-directed RNA polymerase RpoA/D/Rpb3-type domain-containing protein OS=Clavispora lusitaniae (strain ATCC 42720) OX=306902 GN=CLUG_00875 PE=3 SV=1

MEVDSAESGPRVTIRQTERDHVDFILRGVDLSVANSLRRTMLSEVPTLAIDLVEIDVNTS

VLADELLSHRLGLIPLNSEGIENLSYSRDCTCDQYCPNCSVKLELTAKCDSDSTMNVYSS

DLAKFHNGSKLGDPVIRDPAHRGPLICKLRKHQELRLTCIAKKGIAKEHAKWSPCAAIGF

EYDPWNKLKHTDFWYEDDAEAEWPKSANCEWEEAPDPEAPFDYNAKPANFYVDVETVGSL

APNEVVVRGIETLQLKLAGIAVELNKDTVEANDAATGGFTTYGRSPGGNNSPDNGYSYGE

GFGSSAW

>tr|C4XY57|C4XY57_CLAL4 AAA+ ATPase domain-containing protein OS=Clavispora lusitaniae (strain ATCC 42720) OX=306902 GN=CLUG_00880 PE=3 SV=1

MEELGIQMPVVPALALPKSKEWNAPHQEEQQDVYVKLKKLEKELDLLLLQEDYIKDEQRH

LKRELVRAQEEVKRIRSVPLVIGQFLEPIDENTGIVSSTTGSNYVVRILSTLDRELLKPS

SSVALHRHSNALVDILPPEADSSISIVGDNQKPDVTYADVGGLDVQKQEIREAVELPLTQ

GDLYSQIGIDPPRGVLLYGPPGTGKTMLVKAVANSTTAAFIRINGSEFVQKYLGEGPRMV

RDVFRLARENSPAIIFIDEVDAIATKRFDAQTGADREVQRILLELLNQMDGFDQTSNVKV

IMATNRADTLDPALLRPGRLDRKIEFPSLKDRRERRLIFSTVASKMALAPEVDLDSLIVR

NDPLSGAVIAAIMQEAGLRAVRKNRYMILQSDLEEAYAAQVKTGTEHDKFDFYK

>tr|C4XY59|C4XY59_CLAL4 Mannosyltransferase OS=Clavispora lusitaniae (strain ATCC 42720) OX=306902 GN=CLUG_00882 PE=3 SV=1

MAHSNWRSVYFITWGLRILLAIGASYIHPDEHFQSFEVLSSRFFGFSTNIPWEFSSVNPA

RSYVPLLVTYYPVLKVAELFQLSPIQSYFIARLALMVLSWIVIDLCLYKMLPTKQERIKA

IYFTSTSYITHVYQSHTFSNSIETILVVLCVYMINELRFLLSTPSEKVRNTEIATLGLAI

GACASFGIFNRVTFPAFLALPFFIYAKCALTWIWLPVLTLLSFVFTSCVCVVVDTLIYKR

ISLSILAQNPFNWSQYVLTPLNNLVYNSNYANLSQHGIHPYYTHILINLPQIMGPGLLFL

FWRFKNRYYQTTPFLSAVGALIVLSFVPHQELRFLIPVVPLLCCCFDLTVFSETPGSTPV

PVTLIMNSWLVFNLVLSLLMGIYHQGGVVPALSYFQERFYAKNETGVTQIWWRTYSPPTW

LLGDMSNSTQYLDLANAFSPGNKPNTVIDAMGAEYEKVQSALDFAKSVNPQQRIFLVTPV

ASYKQFFNASLFNETWTYPAHLDLDHLDFSKAQSLQLGLGIYELL

>tr|C4XY65|C4XY65_CLAL4 enoyl-[acyl-carrier-protein] reductase OS=Clavispora lusitaniae (strain ATCC 42720) OX=306902 GN=CLUG_00888 PE=3 SV=1

MFQFANVSKKTLLSNSAFAARRMLTAQAVVFTEHGEPKDVLSTQTYTIDENNLAATEVVV

RTLGAPVNPSDINQIQGVYPSQPEKTTALGTSAPSAVAGNEGLFEVLHVGKDVTGFAPGD

WAVPTSVNMGTWRTHALWDEEQIMKVPNPAQSQERGKTPLTIAQGATLSVNPLTAYLMLT

NYVKLRPGKDWFIQNGGNSAVGKFASQMAKLLGFNSLSVIRDRPNLEEVATELREKYGAT

KVITEEQNSSREFSAEVKSWLKETGGEIRLAMNCVGGKSSTAVARKLSPNGIMLTYGGMS

YQPVILPTSLHIFKNITSAGFWVTQLLKGNPELKRDTMDRVIAWYEAGELVDSPSIEKKY

EGDLAETFKRAVADSKDGKQIVTF

>tr|C4XY67|C4XY67_CLAL4 Major facilitator superfamily (MFS) profile domain-containing protein OS=Clavispora lusitaniae (strain ATCC 42720) OX=306902 GN=CLUG_00890 PE=3 SV=1

MSEKNLTESLHPTDSSSGGSVKKDYHDDPLVLAQTEREGERGNILSQYSEKQTMQMGRNY

ALKHGLDADLFGKAAALARAPLDFNSMQFLSEEDKISLNTELTKKWHIPKKLVAVIALGS

MAAAVQGMDESVVNGATLFYPKVMGVTTMKNSDLIEGLINGAPYLCASIFCWTSDFWNRK

MGRKWTIFWTCLISAVTCIWQGLVNLKWYHLFLSRFFLGVGVGVKSATVPAYAAETTPAT

IRGSLVMLWQFFTAVGIMFGYVSSLAFYYVGDHGISGGLNWRLMLGSACIPAIIVLFQIP

FVPESPRWLMGKNRHGDAFESLCQLRHTRLQAARDCFYQFVLLNEEGSYEGIPYYKRLYE

MFTIRRNRNGALGAWVVMFMQQFCGINVIAYYSSSIFVESNLSEIKAMLASWGFGMINFL

FAIPAFYTIDTFGRRKLLLTTFPLMAIFLLLAGFGFWIPKHKRDGRLACITTGIYLFSAV

YSSGEGPVPFTYSAEAFPLYIRDIGMGFATATCWFFNFILAFTWPRLKNTFKPQGAFGWY

AAWNIVGFFLVLWFLPETKGLTLEELDEVFGVSLRKHASYRTKELVLNFRKYVMRQKVEP

LPPLYVHQRLAVTNPDWNEKTEVVHEEEI

>tr|C4XY72|C4XY72_CLAL4 Ubiquitin-like protease family profile domain-containing protein OS=Clavispora lusitaniae (strain ATCC 42720) OX=306902 GN=CLUG_00895 PE=3 SV=1

MSLRKDFSSLRKQRYSGTSPRNSLIGPVHKIVIPTDDVNAGALYVMHPSNPSEMVRSQDV

RTCFFLPIAILDSGLGEVSKISLGVSKVDYNLNLIEASTEGKIRLRVSTTEEYIYFVKGK

AFLDKMAIPKDAIRKIACNEDCSKLLIVLEKRFGFFFVEFTKPYAKFIEFLASWLDDKLK

VLPEEDVISLHDLCIKRRQDLQKDPQLYEVVTSDPTSENVKEDSDNILRNGLPAELNDPN

IATQKVYMGRKTRAVTKLLQEDPEMSFQADNTGEFDEDDNSADEDEPVIQETPAPFDPPL

KHTLANGKKFIVAFNDFKTLYNNDWINDTLIDFFIAYEIDKAVNEFKSVDESNVYAFNSF

FFTKLMSKAEDQEVPDYYENIKRWLSKVDLMSYEAIIMPINEHLHWYCCIIKNLPKLLRY

AKRAQKRKARGLENEKDSPHNSEIVAEIFVFDSLRQTHPNIVSPLKTLIAEYCRDKHGVE

IDTDSIRMVTARVPRQRNFNDCGIHVIYNVRKWLREPSVCERMWKKFAKNQKNYFSGSER

SGLRKTFIDILLDLHSKQPSSSEDASAASPDEHESDDEIELISYHSSKPEEAPPKDANET

NSQEAESNDKPQDSPSSTPNGANKETLMRKSSSEQLGIESSAKKKVKCIEAVEKSPVANT

IRTLDPRVVGTEIPQTDSFESNEGGIVYQIEHPQIRRLCMKMRLKPHTIKFLNEFFEDHS

KAYNEDKQKTISEFVSNYNYFNPQIEVKQCELLIKRFKETLQGPMAPVDEPFVIEEVEES

GEELNRSVSDLRISSDEPKRRKGNTERSTPEATKRFMRETDVKQCSPRLKERSPKTDASP

SLSDAAVDILEDEVQIVSEKAYTSPGRQMRSRLSRRPKNLIQSNLEKLQGKSQTFMSIKG

TASPPRKNTVVTVPDDESKLQDKDGNGRTNSTRDKVNTRSELLGPKRRRVQSSNEPL

>tr|C4XY75|C4XY75_CLAL4 thymidylate synthase OS=Clavispora lusitaniae (strain ATCC 42720) OX=306902 GN=CLUG_00898 PE=3 SV=1

MTASTNPAEQAYLDLCQKIIEEGEFRPDRTGTGTRSLFAPPQLRFDLSNDTFPLLTTKRV

FSKGIIHELLWFVEGCTDAKKLSAKGVRIWEGNGSREYLDKMGLTDRREGDLGPVYGFQW

RHFGAEYKTCDDDYSGQGFDQLQDVIKKLKTNPYDRRIIMSAWNPPDFPKMALPPCHVFC

QFYVSFPQPSSPEPYAPKSTKSQARPKLSCLLYQRSCDMGLGVPFNIASYALLTKMIAHV

VDMDCGEFIHTLGDAHVYLDHIDALKTQLERTPKQFPKLFIKESRKDEIRSIDDFTFDDF

EIVGYEPLPAIKMNMSV

>tr|C4XY76|C4XY76_CLAL4 Shugoshin N-terminal coiled-coil domain-containing protein OS=Clavispora lusitaniae (strain ATCC 42720) OX=306902 GN=CLUG_00899 PE=3 SV=1

MARLSIGSQYFQAKERRESMVYGQNASKPETDKGDPDFPNPSSNTNSGDLPSTVDNYIAQ

NKSLAMKNSEMASRMSEMEAKMSELIRQNALLKNGANGQRNSEKLEQRLRQIETSILERL

GHVLATLSEIRAEESLPENPMVSVVEAALPGRSKPVTSTPTTDNLASFLGEKAEYWGDNE

GETTERSRANSVSPDVDMAMIEGQSREEATELQNERNILPESSQGFSVHMDTSEHNNLAT

VEELVSSPEKGQTKRIKLHPETEKQSLDRSSRRKPVNYRLSQTKNDDFKDDTESENNKTS

RHTINKSKATRKPLSNVTNRRRAALRRKAKDIVTREEEVPDKEIFEFVEPEELETQTRSA

LSRTKRRGVI

>tr|C4XY77|C4XY77_CLAL4 DASH complex subunit DAD2 OS=Clavispora lusitaniae (strain ATCC 42720) OX=306902 GN=CLUG_00900 PE=3 SV=1

MTYIQHMIKSHQQSRCTTPRLSSMSSLQSKIEQKREELENLQELKKFTEILVKQLEQIEE

KFATMADGAESVSLVLSNWKNVMNSVSLASLGLLKYSSKDFKENVPLPECLVRIKLDKDD

EPDIEGDKEESGVAKSPQGDK

>tr|C4XY79|C4XY79_CLAL4 Ribosome production factor 2 homolog OS=Clavispora lusitaniae (strain ATCC 42720) OX=306902 GN=CLUG_00902 PE=3 SV=1

MVDLMALKKPDAKKFSKKNAIRPFEDASGLEFFSEKNDTSLMVFSSHNKKRPNTLTFARF

FNHKVYDMIELTIQENPKLFSDFKKLTFPVGLKPMFTFNGPAFDSHPVFQQIKSLFMDFY

RGDETDLQDVAGLQYIISLSVGEIEDPNSSVLPLLHFRVYKLKSYKSGQKIPRVEVDEIG

PRFDFKIGRRVTPAPEVEKDALSKPKQLQAKVKKNVTTDFMGDKVAKIHMGTQDLSKMQT

RKMKGLKAKYDQVDDAEFDEEDYVADDEPAAKKQKV

>tr|C4XY80|C4XY80_CLAL4 Peptidase A1 domain-containing protein OS=Clavispora lusitaniae (strain ATCC 42720) OX=306902 GN=CLUG_00903 PE=3 SV=1

MRAGILFLVAAASAYMQLDFKVMKGHEFKHFQKRASTNSIIENEALFYLANIEMGSDNQK

VGVLLDTGSSDLWVPQTGCVYGESVEWVQNASPAEAAANSQQCEGYGSYSPSSSSTFKRV

YDDVPFSIQYADESYALGYWAQDSVSFGPSALSSLTFGVADNVTSDIGVLGIGMMDLESS

NSSLADSFEYKNFPAALKDEGLIKSNSYSLYLGTNNVSEGSILFGGVDHGKYDGKLYRMH

IANEFALLGDGPIYSNVILDGISGYGFEAVQQTPALLDSGTSLQYLPGKYVDAVASYLEA

TTFDDDQGMYVVPCSLLQSTDSISYYFSGVEIVVPIRDLLYQYDQCYLGMIDSGDGTTIL

GDTFLKSAYVVYDFDNLEIALAQAAVEPRAETIEDIVTAIPSAVKAPFYDYTSLEIVYTR

VDGNATLTTRHVAEPTFSFNTGPRPSINLGGTAYATLTGPVDVSGTAEPSSYGPSDAISA

SPSSGSVAATVTAEPSGASASGYASSASSSSSASSGASSAAGASSGASSAVGASASAVVT

TDASGNVITVTDSLVSYYATCSCEEESTTSESPSISYTTYTTEIVITTSCTDIGSESTYT

TTEITEVVVTVPCSTETGSVTSGASSSEVSEYVSTYTTVSSVYGTPSVPVDVVSSGVPVG

PAVSSAPAPSSSIPVSGFVSSSTSSGSAVPSTISPIDSGASALRRGALSMVTGALAALFF

I

>tr|C4XY82|C4XY82_CLAL4 non-specific serine/threonine protein kinase OS=Clavispora lusitaniae (strain ATCC 42720) OX=306902 GN=CLUG_00905 PE=4 SV=1

MDGFVTSFKKLRERVSPAKSTDPATSYSVADEKVVLRDEDFCRKRPSSTDDPFTDKSSKV

SKTSKEDSSAFVSTLNRTESTGDFIKDSNLRSAILKEKSPVSEPTDQALASGKAESKDVV

EADPEEDEVTLEVLEEMHKLESCFPILAERYRLLDKIGEGTFSTVYKAEAINGAVMLGSQ

VWKSPPLQKSVAKKQRKTKKNPLVALKQIYVTSSPNRIYNELNLLYMLSGNSHVAPLLDI

LRYNDQVLAILPYYQHADFRDFYRDLPIKGMKKYLWELFHGLEFVHSKGIIHRDLKPTNF

LYDPFKGKGVLVDFGLAEKMPSMVSYSNKSSNACPCLSKEKNTANRVHSKRLNIKAAYPK

VDQRPPRRANRAGTRGFRAPEVLFKCTNQSTKIDIWSAGVIALSLLSRKFPLFNSPDDID

ALIEIILIFGVEKLQKCAELHGCGLEVTIPAVQASNYNGNLIKFITVLLSYEEENECLPP

DSVIHDTLKYLDKNNYSLLKPTFNSSEEMTTADYNKFKEDLEGYNDLKHLLQLLYGCFSM

DPSKRLSASLLLKSPFFSELDTHKDDEILL

>tr|C4XY85|C4XY85_CLAL4 DNA-directed RNA polymerases I and III subunit RPAC1 OS=Clavispora lusitaniae (strain ATCC 42720) OX=306902 GN=CLUG_00908 PE=3 SV=1

MSNIVGIEYNRVTNTSSTDFPGHSAEDHAWDIEKFKRTFDIDIKYIAGRNANFDLRHIDT

SIANAFRRIMIAEVPSVAAETVYVFNNTSVIQDEVLAHRIGLIPLNVDPDALVWIDPAAD

EAERFNDTNTIVMSLDVSCTRNPHAPAGTTDPKLLYRNSSVYARDLKFEPQGRQAELFKD

SPVVPCDPDILLAKLRPGQEISLRVHCVLGVGSDHAKFSPVATASYRLMPVIDILEPISG

EQAVRFQKCFPSGVIGIDEKGEAYVADARKDTVSREVLRHEEFSGKVKLGRDREHFIFNV

ESTGAMSPAEIFLKSVRVLKNKVDYLRNCPVGQ

>tr|C4XY87|C4XY87_CLAL4 U3 small nucleolar RNA-associated protein 22 OS=Clavispora lusitaniae (strain ATCC 42720) OX=306902 GN=CLUG_00910 PE=3 SV=1

MAKRKLDEVQASAVPNDDAGIDAVPESINGNNSDAGSDSDVEEEEEEDEAKTEEETIRQQ

GRPTKKQRKQLSAQEIQVARETAELFKSNIFKMQIDELVSEVKIKESNVSRIEKVLHRLH

GCISQIPASESLTLEEAENLINPKKVVIPFPDPKPTKTNYKFGYLPPEDVSLVGSFGLKT

GIAQREGMSIDISLTMPKSLFSPKDYLNYRALYKRAFYLAYMAEHLIHLTKKNNLPVKIS

YCYLNDDVLCPMLKLESIKTDNKDDLCFHKTKFSINLIAAFPFGVFEPKKLLPDRNCIRV

QSDSEELPPTPYYNSSILSSSTYDYYLKFLYASKKSADAFKDACTLGRLWLQQRGMGSSI

NKGGFGHFEFAITMAALLNGGGANGNKILLHGFSSYQLFKGTIKYLATMDLTSGYLSFSG

EIGDSISCKYNADAGFNTPTIFDKNVKLNILWKMSNSSYQALRLQAISTYSLLNDVVYDR

FDPILLRKTGLNCMKYDLVYNVELPEDLYESFGALEKISFITFDNYVKHKLYVILKTALG

ERVTSLEILNEKVSNAFPLTRRKPTQPHCTKYTIGLELNADECDKLVTKGPNDIDEEQAA

KFRAFWGSKSSLRRFKDGTIQHCVVWTVEKNEPLVFQIMNYALNTHVHRLISQHASSNAS

KFNARLPTPSNALASNSGVTSTANFTLLKSSFEDLCKIMYNLEMPLGIKSILPASPALRN

TSILQPVPFAVSDPNFWNEVVLQFETSSRWPDEIKALEKTKAAFLLKMSDTLNKETTYKT

FLSRDHSIPFNEDVTILNVMAPSGFGFKIRVLTERDEILYLRAVENAGTQKAIVQNIYLK

FNQRYIGSIKHTRTINILNSSFPYYSPTVRLLKQWLDAHVLLGHFTDELVELIALKPFVD

PAPYTVPNSVEKGFLQVLDFLASWNWKDDALILDLVKRSDAVVTDLDNKLSDKLSVQAYQ

LIQSNFEKIRKSDPSAMKTQFFVGSKDDASGILWSDEVTLPIASRLTALARAAISLVKDT

AFDQATMDLFFTPALNDFDFFMKVKGSNLNKSSGIKREGEFKNLNTNLTSYPTDITSRYD

LSSVLVDELKKKFGNIIIFSSRKCPVLFGEGDNVVCGVFVPTAMSKKKFRVSLGMDVKPT

GDGDEVVINKQEIFDQISLLGGDLIKSIKFRK

>tr|C4XY91|C4XY91_CLAL4 Casein kinase II subunit alpha OS=Clavispora lusitaniae (strain ATCC 42720) OX=306902 GN=CLUG_00914 PE=3 SV=1

MHTKISPPKPGNPTMSSKVYSVARVYADVNKQKPQEYWDYEAHVIEWGDIRNYEIVSKIG

RGKYSEVFEGVNVLNDQPCVIKVLKPVKMKKIYREVKILKNLTGGPNIVGLLDIVRDQQS

KIPALVFEQVDNVDFRILYGKFTIPDIQYYFTQLLIALDYSHSMGIIHRDVKPQNIMIDP

KAKKLRLIDWGLAEFYHAGMDYNVRVASRYHKGPELLINLQQYDYSLDLWSVGCMLAAII

FKKEPFFRGDSNNDQLVQIAKVLGTKDLMAYVNKYRIKLSDEYNSILGNYPRKPWSAFVN

KDNQHLVSPEVVDLIDKLLTYDHQLRPTANETREHPFFSL

>tr|C4XY94|C4XY94_CLAL4 Nucleotide exchange factor SIL1 OS=Clavispora lusitaniae (strain ATCC 42720) OX=306902 GN=CLUG_00917 PE=3 SV=1

MAISSSSLTSIFTGSHFAFFPFLFNFASLSTCFLISPLYAHTSPPDSCTQESHSYFSSIT

SVFFIIMKYSALIISIFVSLAAAGLIDPTLEVVCPDSNVAHCYPKVFKPTDEWQTVREGQ

DIPPGLHVRLNMETGEREAKLVSEEDSDAQVPVLVEGAQVESEVPIEQKIQETLKKYKQE

QMNFRKSKVSESELSDYASSVEELLHFGKNADVSRLEKALDTLIDLSHDIDFGERLTRDP

KVFSSLLDAAQLTSSESQIPEKSFRIMGSSLRNNPAAVTNVFEKQDDNFMVTLFATLESP

ETSDVVRKRIAGVIHALASDMAFAYQFFNVEDASKSLGFSRLLRIFPKADHETQERIAML

FEDLNLLPSSPDKRDEDSPKPQERVSEMLQTLVPASQSEEQLQNMFKSLVELHRNHDLRP

SKQFLQWLSKETEMRKNTKNVRDKTDADFDTFMLEARHLVFGNPNALRKADEF

>tr|C4XY95|C4XY95_CLAL4 Glutathione synthetase OS=Clavispora lusitaniae (strain ATCC 42720) OX=306902 GN=CLUG_00918 PE=3 SV=1

MTFKDFPELSETSQAELVEKLLQWALANGLVMYPPNYEVHSTNNAPVTLYPTPFPKDMFE

RAVNVQQGFNKLYVSVVANQKNWLIDLIEGLSDYDKDFTGKLYETYIKAVEFGKGKVVQP

LSLGIFRSDYMYNQADHSIKQIEFNTVSVSFGGLSSKVGQLHNYLNNSGAYDESYSNKYY

SEHDLPISDSIDQIANGLAQGNYFYNGEKQNTATVVVFIVQNGERNCFDQRHIEYALLNN

FNIKSYRFSLEQVESHLTVNSGKLYVKKTMDEVSVVYFRSGYSPVDYESDPESTWAARLF

LENSAAIKCPSVLTQLSGAKKVQQVLTSRTTVENFLPDASEEEVNELLATFVKIYPLDNS

EEGQLAKKLVAEHPENFVLKPQREGGGNNVYKENIPKYLKSLPEKEWEAYVLMELIHPPT

HKNKIIRNSDVYHEEIISELGIFGTVAFNEETGEILANENAGHLLRSKFSSSDEGGVAAG

FGCVDNVYLY

>tr|C4XY98|C4XY98_CLAL4 aspartate--tRNA ligase OS=Clavispora lusitaniae (strain ATCC 42720) OX=306902 GN=CLUG_00921 PE=3 SV=1

MSAEETKKLEDLSINKPAAPEGEVILGEDGQPLSKKALKKLEKEKEKARKKAEREAQLAK

EKAEKEAQAANDPAKENYGKLPLIVSDSKTGIKRIQIKDLSAANDGEEVVFRARAHNSRQ

QGATMAFLTFRQQDSLIQGLIKANGSTVSKQMVKWAGAINLESIVVVTGVVKKVEEPIKS

ATIQDAEIMISKIYTIQETPEQLPMLIEDAIRSDADAEAAGLPVVNLDTRLDARVIDLRT

PTNQAIFKIQHGICALFREFLSSKGFTEIHTPKILGSASEGGSNVFEVSYFKRSAYLAQS

PQFYKQQLIAADFEKVFEVAPVFRAENSNTHRHMTEFVGLDLEMAFEEHYDEVMEVLENL

FIFIFTELKNRYAKEIATVRKQYPVEEFKLPADGKMVRLHFKEGIAMLRAAGKEVDDFED

LSTENEKLLGKLVRDKYDTDFYILDKFPLAVRPFYTMPCPEDPRYSNSYDFFMRGEEILS

GAQRIHDPELLKERMKAHEVDYTMDGVSDYVDAFTYGCAPHAGGGIGLERVLMFFLDLKN

IRRASLFPRDPKRLRP

>tr|C4XY99|C4XY99_CLAL4 CCT-beta OS=Clavispora lusitaniae (strain ATCC 42720) OX=306902 GN=CLUG_00922 PE=3 SV=1

MSVQIFGEQASEERAENARMSAFVGAIAVGDLVKTTLGPKGMDKLLQSASDPERSMVTND

GATILKSIPLDNPAAKVLVNIAKVQDDEVGDGTTSVTVLGAELLREAEKLVEKKIHPQTI

IEGFRIARNTAIAALENVAVNNAEDTQKFRADLINIAKTTLSSKILSQDKDHFAELAVSA

ILRLKGSTNLNHIQIIKKAGGKLADSYLDEGFILEKKFGINQPKKITDAKILVANTSLDT

DKVKIFGAKFKVDSTSKLAELEKAEKLKMKSKVEKIKKFGINVFINRQLIYDYPEQLFTD

AKINSIEHADFDGVERLALVTGAEVASTFDNPDKVRLGHCDSIEEILIGEDTFLKFSGVA

AGEACTVVLRGATEQGLDEAERSLHDALSVLSQTTRETRTVLGGGCSEMIMSKAVDQAAA

NETGKKSLAVEAFARALRALPTILADNAGFDSSDLVTKLRSAVYNGITTSGLDLNSGKVA

DMRDMGIVESFKSKRAVVSSAAEAAEVLLRVDNILRAKPRTADRNH

>tr|C4XYA0|C4XYA0_CLAL4 Nuclear polyadenylated RNA-binding protein OS=Clavispora lusitaniae (strain ATCC 42720) OX=306902 GN=CLUG_00923 PE=4 SV=1

MLYEVSVLRYGNVAGQFFCLAVIYVRAPEHQKTPMGFLRQKKKNYYATTFFLPHRYILIS

NTMSYDPEGPTAKQLLPLLVNELTSTYGIPDDAQDVAEYINVLIGNNRTAADICSEVKEV

VNIPIDEPFIGRVFAEIAKLEQSRQEQAQTQTQPQAQTQETSQFQQPNFQSEQPQPESQP

QSFPNEQPQTFQPESVFNQPQNFQNNNSVFSNATQNTQNGQSFPTEQNFASGQNFQNGQN

FTNENAFPQSAFPSYSGNQDVPADSEDKSMHVDFQKLGNELRRAAPQGPKGARGGKSGIS

KDFQSNSRNQKKSFGMKNAANLEKALALSSNAQTVNMAPFVPRAPKGRCKDFPYCKNREC

QFSHPTKKCFAYPNCPNPPGTCDYLHPSEDGELMQELEKTKRQFMDRKKERMMVPQVTLC

KYGILCSKELCPFGHPTPANKDAKVIIPKWCRENKNCQNENCEYAHSSPNYQAPVQAPAP

AAQYIKIPGGSGPKFMNKTGPKFSNFNKRTPTTLEQCKFGMNCTNQSCPKRHSTSSVACR

GGYNCTRLDCTFNHPIEEDCRFGNECKNKYCYFRHPDGRERALFENSSDSTSNRSFAVPE

DQVMEQAVQQ

>tr|C4XYA5|C4XYA5_CLAL4 Globin domain-containing protein OS=Clavispora lusitaniae (strain ATCC 42720) OX=306902 GN=CLUG_00928 PE=4 SV=1

MLSGSTSMRRPTQLGRDIPKSYSTQSLANAREDASIPDTQSENSDMYFAPSYSDYTHENY

KVTRVGTRDSCNSAYSNQSVYRVSLDFSPKEIALVRYTWNRMLVDDAEPKISLPGAFARP

KKMAQSSLHASSTFCTQLYSNLLSMDPELEQAFPSLRHQAVSMAGVMSLAVNSLDNLSSL

DAYLEQLGKRHSRILGIEPFQFEMMGEALVQTFVQRFGSKFTQQLEVLWIKFYMYLANTL

LQFGLDPVLRLEPGAPQVFPRQSRPESVFSGDTLSLLNSARRVSQSTGLTEVSSVLEKSA

APKVRAPPAQKRRLRLKKKSDCVIV

>tr|C4XYB3|C4XYB3_CLAL4 non-specific serine/threonine protein kinase OS=Clavispora lusitaniae (strain ATCC 42720) OX=306902 GN=CLUG_00936 PE=3 SV=1

MSSAAAIAAAAASSNNQKMSAPNSSSSSANNNSVVGLHYKIGKKIGEGSFGVLFEGSNII

NGVAVAIKFEPRKTEAPQLRDEYRTYKHLQGCDGIPNAYYFGQEGLHSILVIDLLGPSLE

DLFDWCGRRFSVKTVVQVAIQMLSLIEEVHRHDLIYRDIKPDNFLIGRQGFPDENKVHLI

DFGMAKQYRDPRTKQHIPYREKKSLSGTARYMSINTHLGREQSRRDDLEALGHVFFYFLR

GQLPWQGLKAPTNKQKYEKIGDKKRTTSASVLCEGLPKQFAEYLESVRSLPFEAEPAYEE

YRMLLLSALDDLGLHADGDYDWMHLNGGKGWDSSINKKPNLHGYGHPNPPNERERRHREQ

RRNRPHHSAQPGGNIPNSAGNSNLNNMSQSAQQQQQLSATQSHQQKLQHLINRPLPPIKQ

ELQPNSSMGGNMNNDMSGNQAMMRNSQTNNGQRVNQYEQQQHVEEEKKGFWSKFCCQ

>tr|C4XYB7|C4XYB7_CLAL4 Ubiquitin-conjugating enzyme E2 6 OS=Clavispora lusitaniae (strain ATCC 42720) OX=306902 GN=CLUG_00940 PE=4 SV=1

MVSRQAQKRLTKEYKSIAANPPPFITAKPNEDNILEWHYVITGPPKTPYEGGQYHGVLRF

PADYPFKPPSISIITPNGRFACNTRLCLSMSDYHPDTWNPAWSVSTILTGLLSFMTGTEQ

TTGSISTTDGVKQKLAAESKRWNTSQNSRFGSVFPELAQQNAQDLAAQERLEQEMLARNA

QQPSPSPLQMDQLDPEDRARLLVQQEHAPIGRSLAAAGIFLAAVVAMYLTF

>tr|C4XYC7|C4XYC7_CLAL4 Major facilitator superfamily (MFS) profile domain-containing protein OS=Clavispora lusitaniae (strain ATCC 42720) OX=306902 GN=CLUG_00950 PE=3 SV=1

MHESLYTSHRITPHLAWTTALSCLGTLQFGYHLAVLNAPQQILSCQMHIPGPFPSYSDTF

WGRHGFDQCISLDSQDIAWMNTMFTVGGLLSSTIAGSHTVASICGRRNMQILCAGLYAGG

SVIFALSNSFGTLNAGRFLSGLGAGASMVVSPVFISEISPFNHRGLMGSLLQFGVAIGIL

LAQLIALLWGNDQQWRMLFVFGTLMALFQFVFLFSTVESPKWLIMHRGEISRASDILQSL

RSNVRATKYEIHHWRRLSTTQTGKAQETSSLLEDPVNQETQTNVGDEDSQPEDGFYPLST

AMSRRGSIDPSTLSLQEYFTSRRYRREIIATAIIMTAQQLCGMNAITFYGVSVLSNIVPN

GTNVLLLTCSLALCNVVSALAISPFIDRWNRKSLLLLSVSTMAFCSVLIAGGIVNGLDIL

AAVACFGFIIGFSIGLCQIPFLMVSEFCNHETISKSQSFGTMLNWLANIAIACMFPLLRS

YLGGYTFLLFTVIGIFYLFAIVWWVPETKGKLNYADIWNKDELP

>tr|C4XYC9|C4XYC9_CLAL4 Sensitive to high expression protein 9, mitochondrial OS=Clavispora lusitaniae (strain ATCC 42720) OX=306902 GN=CLUG_00952 PE=3 SV=1

MFMYGRFWATAKCIPLPKPHQSIFAVRHSSTTNFSDEKLRQIIETSKVGIARREQENKTK

TGHTPAPIEESKKSTYKMPPSTDKLESNLSSNSISTNDANEGKKQVNAMAQALQARLGEL

RGTLSVVSKALNDLTGYSAIEKLKTLVIDFEEELRQAKKNLKAAKLEYTEAIQNRSDMQK

EINELLTRKHNWSSGDVERFTELYKNDHINQQQEEQAERKLEAAEQNVDTVQNLLTQSIL

TRYHEEQMWSDKIRQVSTWGTWLLTGVNVLLFIVAAFFVEPWKRRRLVDAFQKEMQVKVD

AFSEDIKNLSGQIAAATEAKDERTSVETPPLLEKADFFQLNFSSIRTWSDAKRWFRATAS

ALQNPQVGTYMMDKTEFWTFTAVFVAVGCTLGSALSYAFLSYK

>tr|C4XYD0|C4XYD0_CLAL4 FK506-binding protein OS=Clavispora lusitaniae (strain ATCC 42720) OX=306902 GN=CLUG_00953 PE=3 SV=1

MSSLLPISTYNLALEPFNPTPALDDEFPVTVRLTMAAVDPEAVDDKEEPSVIRILKRSRA

FIDDDEDDLEAEEADELDSDEEEEEEEEEEKPAKSKKSSKAKKDEDDEEDDEEEDDEEDS

DIEDDEEIEEFILCTLSPKVQFQQTLDFTISPYEEVHFVVTGSYPVHLSGNFVEHPGDDE

DDYDSDELDDEYYEDDEDEYNLTPDEDELMDIEDLEDASDVEGKITELVEADEEKSKKRA

AEEENSPKAKKAKEDKKKVQFSKELEQGPTPSKKEKKDKKEKKEKKEKKEKKEEKVEEKK

EDKKEEKADKAKKFPTKTLLGGVITEDRKIGSGQGAKSGNKVGIRYIGKLKNGKVFDKNT

SGKPFVFNLGKGECIKGFDLGVAGMAVGGERRVIIPPKMGYGSQALPGIPANSELTFDIK

LVSLK

>tr|C4XYD3|C4XYD3_CLAL4 Tricalbin protein OS=Clavispora lusitaniae (strain ATCC 42720) OX=306902 GN=CLUG_00955 PE=4 SV=1

MSKEEVEKQTKKKTATQAAVKNPADGSNKQSRHKVPEEKMKSEPYTKPGFNWRQIGSWDN

INKDDAHKVLKASKNIEEYVMDHFYGDWYHNSVIPIGVCFFSWAFARIGFSFLWLFVVLL

GASSVYYAEFRRFNRDVRDDMRRAKAARRLEEESETMEWLNSFLAKFWVIYMPNLSEMVM

YQANQVLNDAAPGYGIDNLSLDEFTLGTKAPRVDSIKSYTQKGKDHIEMDWAFSFTPNDT

DDMTKNEIKKKVNPKVALGVTVGKAFISKTLPILVEDMSFTGKMNIKLKLNDNFPHVKTV

SIQFLEPPVIDYALKPVGGDTFGIDIMSFIPGLSTFVNTLIHATLRPMMYAPNSLDIDVE

EIMAQQSNDSIGLLSVHIKRIIDLKSTTDIKDNVFHPYVQLGLSNNPKVIEKTKVKKDTT

QPVYLETKNLLVSALDGNHLLLNVFHMVPDQKDDINLGLLEVPLADLLQTEVQTGMVKNI

LESGKVVGKIEYDLKWSPALKPVTLEDGTREEYSDVQTGIMKLLVSGATNLDISHSVTGV

LNPYAEIYINNKFVKRSRNLRETNEPDFGVSFESFITQQSETQIQVLVKDAAEDLVVGKL

DTNLQDLIFETSRGQQWLSAPSVAEGGEPAKFKIGAKWKAINVDDEEVEIQKNASIGGLR

LHLLRASGLKNLEAVGDVDPYVKVIQDGKLKGKTPTIANTSDPVFNNVFYVPVANEHQHI

LMDIFDAEPEGKDRPLGSCAVAVKDFLKKNSKGYYLGYDGSKEIVEQPVLYNGGNCGTFY

FSVSFIPTIPVYTHLQLSNYDQYLELKKKKEEEEKKQAEEEEKLMKENPTQYEWIELAED

NIGDAPKVKMTAEESVKYRSGNVVVHLLSGSFNKPDVYVHVLFDDQAYPSVVTPKCSNRS

LTAPNVGEGFVRDLPNSKVVFRLSKKAEVMEEKEVLVEKIFNTMDLLKRAYQKPITVHVD

QRNTLRVQMEFVPFASNLDPLDTVLDVGHIRLDLLNGEKLKSVDSNGKSDPLCAVKLNGI

EIYRTDKKRKTLDPLWNESVEFPMLSRSRDTLMLEVYDWDLTHDDELLGRVVLDLSQIPP

NETTSFRAELDTSGFVNLRATYRPEFIRPKLNKKGGLQVDLNGVAGVPMKLVGGAVGGAT

DLASTAVSSGVGAVADGFTKGGSFLKKGLGRKKRSGEKDSEADNSVMSDQVSETSHQTGQ

TNNTSKRSNGKSKSDKYEEPPPMEPSNDNDDSIEALPNIVPELLPPPQKPTAQRHMRNAS

GTSDLSSLSSGTGGADAIPGRVNIVSAEGFKSSALEVKVSLSTQPKAKGIYKTRTAKAQG

GKFEWNESRVFKASPDSSLVVFVREHHAFGRNVVVGTAQIPLQDYVDKDEIITVPVEEGQ

VTIHLRYMSVL

>sp|C4XYD5|RS3A1_CLAL4 Small ribosomal subunit protein eS1A OS=Clavispora lusitaniae (strain ATCC 42720) OX=306902 GN=RPS1A PE=3 SV=1

MAVGKNKRLSKGKKGLKKKAVDPFARKEWFDIKAPSTFENRNVGKTLINKSTGLKNAADG

LKGRVFEVCLADLQGSEDHSYRKVKLRVDEVQGKNLLTNFHGLDFTSDKLRSLVRKWQSL

VEANVTVKTADDYVLRVFAIAFTKRQANQVKKTTYAQSSKLREVRKKMIEIMQREVSNVT

LAQLTSKLIPEVIGREIEKSTQSILPLQNIHIRKVKLLKQPKFDLGSLLALHGEGSTEEK

GKKVSSGFKDVVLETV

>tr|C4XYD7|C4XYD7_CLAL4 Origin recognition complex subunit 1 OS=Clavispora lusitaniae (strain ATCC 42720) OX=306902 GN=CLUG_00960 PE=3 SV=1

MAKRQKDLEDWKIVLDDDEPSDPSTPSRRSRRNAVRGASTSVKLHRETDDTHLEVGNCVL

INGHSRTQRSGNSFYAVIMDIQIGVKNFLDLQVVPLVHIKNVRSDDLPPEYSNAEPANEV

FVTSELEFVQLKDFVEKIQVLNKSDYSEIATDDTSTSTIFLCRRGCDRFKEKFSSEFDFK

DWQALVKKNFNHALNYIAEMTSVIVSPTKANRKTRSLKSQLEQVSSPSRGRYSKMFDSDT

SESEVSETETETEEKLNEEENEMDDEIEEPEEAVKQKTPRKRSSKSATTTPSPRKRSKRD

NEAVKRLQSVLSPLKKGFKVKSGSTISSLPSLSRAVDKSDASKHSMIDTSSEAFKQLKEK

LHTSTRIDSLPCREDEFTSLLLTLETAVREETGCSVYVSGTPGTGKTATIKEVIASLKEI

VSYDGLREFDFLEINCLKLLTPNSAYEKFWEYLSGIKVTPSNAALLLEEYFSRDVPDPDR

KPLIVLMDELDQIVTKNQNVMYNFFNWPTYANAKLIIIAVANTMDLPERLLSNKISSRLG

LRRIQFVGYTYEQLGQIIKQRLEMLAEKNKRRVTVSPDAVGFASRKVASVSGDARRALTI

CRRAVEIAESEYLATAKDTEKLPESDQEYSIQISHISKAINETTNTPLANLVNSLSFASK

LALVSVLLRTRRTGLAENTFGDICDEMRNTLRLLTAKESSSALNEISSSASYIDLLYSHG

VLHDSPQSLNIRVFKMAEIINELVEQGILVQQNIRSERYRLIHLNVSGEEVESILKRNAE

VAAML

>tr|C4XYE1|C4XYE1_CLAL4 Serine/threonine-protein phosphatase OS=Clavispora lusitaniae (strain ATCC 42720) OX=306902 GN=CLUG_00964 PE=3 SV=1

MSNKAVANRNQMKNALQVITNKNSSEKPPRDLSVYITEEGEKLSTTERVVPSVSPPASFS

PPDSDVFLPNGLPNCAFLKDHFFNEGRLQHHQAIHILQEATRALSMEPTLLTVPAPVTVC

GDVHGQYYDLMKLFEVGGDPATTQYLFLGDYVDRGSFSIECLLYLYSLKLNYPKTFWMLR

GNHECKHLTDYFTFKSECLHKYSKEVYENSLESFNALPLAAIMNKQFFCVHGGISPELKS

LDDLATFDRFREPPTKGLMCDLLWADPIEDYDEDNMDQTFVRNTVRGCSYAFTYKASCQF

LERTGLLSIIRAHEAQDAGYRMYKRTKTMGFPSLLTMFSAPNYLDTYNNKAAVLKYENNV

MNIRQFNSSPHPYWLPHFMDVFTWSLPFVGEKVTDMLVSILNICTEEELEEETPIAHGSA

QPSPILPTSPSLPDSASDSEELTIDEKKAALRRKVMAIGRVARMYQVLREESENVAHLKS

LNSGTLPRGSLSHGREGFQNTLMTFESARAADLENEAMPPMPEELKKREAERQAKIRREL

SASPSPSFSRLMRRLSG

>tr|C4XYE2|C4XYE2_CLAL4 ornithine decarboxylase OS=Clavispora lusitaniae (strain ATCC 42720) OX=306902 GN=CLUG_00965 PE=3 SV=1

MHPSVDVISEDGYVLTPQKHSFEASGSVTSGHKRSSLASSDLVRSALVDQVNQIDHDVCA

AGDEDSFFVADLGQVFRSFQLWKETLPRVQPFYAVKCNTDPHVVSLLGRMGANFDCASKT

EIDTVLGLGFTPDRIIYANPCKTNSFIRHACEKNVNLTTVDNANELYKLKRFHPRTRILI

RIVTDDESAQCRLSTKFGCTVEVAVNQLLPLCRELDLDVAGVAFHVGSGATCFDSFFHAI

KDARRIFDAGEQLGLRLTILDIGGGFEPCSFDAATQVVNRALDTFFPTTYAQQHGMQFIA

EPGRFMVANAFTLATHIIARRDLDSNSGMEAMLYINDGVYGNLNCVLFDHQHPEAKVLKH

AGKMFPGEMDGETQSQHYNFSIWGPTCDGLDCVSSKTALPANVGVGDWLYFPELGAYTSA

ATTSFNGFSGCAKVNYVCSVDLGEYGY

>tr|C4XYE3|C4XYE3_CLAL4 Vacuolar protein sorting-associated protein 27 OS=Clavispora lusitaniae (strain ATCC 42720) OX=306902 GN=CLUG_00966 PE=3 SV=1

MSWFRATDPVQELDTKIEQATSESIPNGELDIAVGLEITDIIRSKKVPAKQAMRCLKKRV

TKVYLNPNLLTSTLKLVDLCVKNGGNHFLAEFNTKEFIDYLVDYIFKVHYDTKSYKVYSS

EAKYGVSTLLLKFLKEWSLYFKGRFEPNYVESHYKTLVNQGYDFPEVDVAAVDAGANFIE

TTAPLDWLDGKECMICYTPFSVMNRKHHCRACGGVFCQTHSSKNIPLFSLGIHQPVRVCD

DCYEIHKGKNDKERNRRNNGASGAESVPVADSEEDQIRKAIELSLQETSISAAPASTQPP

PPPRNPPTQEAEEDMDPDLKAAIEASLKEAEASKQPKQYVEERQEQPPAAAPEVDFYSNI

MPFDTNTYAAPAPAQYNETGMEAPVYNAPNYPPNVQAAYTGVNQFSNVQGPPPYGTSGSV

FDATNPWSQHQKQQQQQQQQQQQQQQQQQHPRGGSQPTGNPVRMEPNKTSESLTPEEEDA

INLYIRLMNGIKNDRVKQANIIYDKDLSELNAKVVKLKPKLNKSLRSAIESYGFFLQMSN

KISTITRLYDSFLETKLNMAYNMHNISSSAAEYTGASLPNVYNESAHRYSSDTLGHNNGA

QKPVRPTIDTQSVRPRGSRVASFTGSQVSEVEKRKQGPFVDSPYSHPEQSRYDNQSQKYG

LTNRSRGNSQTTPYPVDDGSQYPGYRKVYPEPNERRRSQSSYGRNMSNHGSSHFMPSPST

FLPGDANTRNKPSDGPDIENGSSTPNESSFEPSAPNFEPSAPSFEPSAPNFEPSAPNFEP

SAPNFEPSAPNFEPNAPNFESNAPNYEQTEETFVPEDKRELEYGPEANLPSTYNSKTYRE

SSSPTVSKNESKRRSVYPSEPSYLPEDNSDSESVASMYPPVPGFSEEEDKTPEPHKEHAS

TRFPSLSDLKDLNSSGTSLPDVRKTSSRESKRYKSEPEPLIEL

>tr|C4XYE4|C4XYE4_CLAL4 Protein ROT1 OS=Clavispora lusitaniae (strain ATCC 42720) OX=306902 GN=CLUG_00967 PE=3 SV=1

MLAYFISLLLLSCAFAAPNMEELEGTWTSKSNTVFTGPGLYDPVDELLIEPALPGISYSF

TKDGHYEEALYRIKSNPQNHSCPIASLTFQHGTYEILSNGSVVLTPIAVDGRQLLSDPCG

YSAEKAKYVRYVQPTWFEKYQVSINSYHGRYMLQIYQFDGSPMQPLYLAYKPPMMLPTTA

LNPTDKASETSSSLSRKVKRSLENQYRTNAVKKFSSEKFDKFWWMSVGVLGVGSAAWFLR

>tr|C4XYE6|C4XYE6_CLAL4 Ethionine resistance-conferring protein OS=Clavispora lusitaniae (strain ATCC 42720) OX=306902 GN=CLUG_00969 PE=3 SV=1

MPDTSETTGLLSVTSARSNEVSEEEFINYVQQRRASIASVGTDHIPPHFALSAKRSRNSV

DDTLSAARSNISVANSSINAILSHNSEVLLGEPKTSLYTETKVLLEYSVPLVITFLLQYS

LTVASVFSVGRLGSKELGAVSLSSMTANISGYAIVQGVSTCLDTLCAQAFGRKDYTTVGL

YFVRCTYLLALLYIPIFILWVFCSKSILVFLVGESQKELAELAATYLQILALGLPGFILF

ENLKHFLQAQGIFHASTYVLLVCAPLNALLNYLLVWNKHIGLGFIGAPLSVVISDWLMCF

FLLWYALYVDGYRCLPRLSLFDRRYFQNWRKMTDLSVPGVLMVEAEWLAFEIITFTASTF

GTDVLAAQSIVSTTCVLLYQVSFAISIAASTRIAWYVGAASKKALLIATKASIYTAAVFG

CLNAIILFSLRDFFASLYTNDPNVLHIASQVLIVGALYQINDFLACTTGGILRGQGRQKI

GGYLNLISYYLIALPCAFVFAFYFDLKLVGLWMGMVIALLFISVAQVYFVIFSDWDKIIR

SCINEGVLEEGNVHIDAHSVLPAMSSSNIV

>tr|C4XYE7|C4XYE7_CLAL4 C-8 sterol isomerase OS=Clavispora lusitaniae (strain ATCC 42720) OX=306902 GN=CLUG_00970 PE=3 SV=1

MWVKFLSIAVAFFAVLEASFQTWLPSRFVFDPKLLQSLAQETLAAHPDGNATAIMIDLTP

KLQAAYPGLINDLNWDDWMFNNAGGAMGTMFILHASISEYLIFFGTAVGTEGHTGVHYAD

DYFTILTGMQSASLPNAVLPEEYRPGDQHHLQRGYVKQYSFDRGGFALELAQGWIPVMLP

FGFVSFASSTLDYYSFARTTYFTAKDMIINLLRGKF

>tr|C4XYE9|C4XYE9_CLAL4 NADP-dependent oxidoreductase domain-containing protein OS=Clavispora lusitaniae (strain ATCC 42720) OX=306902 GN=CLUG_00972 PE=4 SV=1

MWHISTVKSPPHLLINCYLSHHFTPVLTMSLSNNKFQLSNGNSIPAVAYGVGTKWFKFGR

NELDANVVNAIKSALAKGFVHIDGAEIYNTNGEIGEAIAGLDRKNVFLTDKYFSGDSSHT

RISPHGKPYDSLKHSLQNELKVDYVDLYLLHAPFISKESHGFDLAEAWKSMEQAQKEGLT

KNIGVSNFAVKDLEEILSVAEIKPVVNQIEFNAYLQNQTPGIVEFSQKNGILVEAYSPLA

PLVKGEKGEFTDYLDTLASKYKKLPSQILLRWVLERGVLPVTTSGNEERQSQLVNIFDFS

LTPEEVEKIKTLGEKHPTLRQYWTKEYSKYD

>tr|C4XYF2|C4XYF2_CLAL4 Phospholipid:diacylglycerol acyltransferase OS=Clavispora lusitaniae (strain ATCC 42720) OX=306902 GN=CLUG_00975 PE=4 SV=1

MSEKLHNRKKKVVEDENVIEEFDADEVDDSNTPTTEDAPVSRSHSRHSRHKQAKDKSEIK

KKLYQTRRATFVLGAVIGLLITIVITAASSDDIKSELDKLVSFDSVSDLFEDLKGSFPQG

SQSMLEFDESKKGTELHGSAESFSVGKRMKKVHNLTEKYNVVLVPGVISTGIESWSINDE

GDCPSTPHFRKRLWGSYYMLKTMVLDKACWLKHIKLDPVTGLDPPNIKLRASSGFDAADF

FVAGYWIWNKVLQNLAVIGYGPNTMTSAAYDWRLAYLDLEKRDGFFTKMKLSIEMSKKLS

GEKTYLVGHSMGSQIVLYFLKWVEAEGEFYGNGGPNWCNEYLAGFINISGSLLGAPKAIS

ALISGEMKDTVQLNQLAVYGLDKFFSKKERVEMLRTFGGVPSMLPKGGDVIWGNITAAPD

DPTNHLITNTSDVEISGTKSDTFGTFIRYKAKSNESASITGIEEDEKDFSMMDSIDLLLN

TSPKWFRDRVKEQYSFGIAQTKEELEENNHKHSKWSNPLEVTLPKAPDMKIFCFYGVGNP

TERAYTYKEDLTSGLPLVIDTESKNPVYFGDGDGTLSLMTHTICHEWQKEGSRFNPAGID

VKVVEIKHEPDRFDIRGGAKTAEHVDILGSAELNELVLKVVSGHGDSINNSYVSELADIA

KRLEI

>tr|C4XYF7|C4XYF7_CLAL4 V-type proton ATPase proteolipid subunit OS=Clavispora lusitaniae (strain ATCC 42720) OX=306902 GN=CLUG_00980 PE=3 SV=1

MATTSTELAPFFAPFLGFAGCSASMILSCVGAAIGSAKSGIGISGIGTFKPELIMKSLIP

VVLSGILSVYGLVVSVLIAGGLNPTEEYTLFKGIMHLACGLSVGFACMASGYAIGIVGDE

GVRQFMHQPRLFVGIVLILIFAEVLGLYGLIIALILNTKGA

>tr|C4XYF8|C4XYF8_CLAL4 RuvB-like helicase OS=Clavispora lusitaniae (strain ATCC 42720) OX=306902 GN=CLUG_00981 PE=3 SV=1

MVGQLKARKAAGLILKLVQAGQIAGRAVLIAGPPSTGKTAIAMGLSQSLGKDVPFTAIAG

SEIFSLDLSKTESLTQAFRKSIGIKIKEETEIIEGEVVEIQIDRSITGGHKQGKLTIKTT

DMETIYELGNKMIEGLTKEKVLAGDVISIDKASGKITKLGKSFTRARDYDAMGPETKFVQ

CPEGELQKRKEVVHTVSLHEIDVINSRQQGFLALFSGDTGEIRSEVRDQINTKVAEWKEE

GKAEIVPGVLFIDEVHMLDIECFSFINRALEDDFAPIVVMATNRGVSQTRGTSYKSPHGV

PMDLLDRSIIIHTSNYSADEVRTILSIRANEEEVELTADALALLTKIGQETSLRYASNLI

SVSQQIASKRRSANVELVDVKRAYTLFLDADRSVQFVTEYSNKFIDDNGNVMLGSNGKAE

DDKMQTD

>tr|C4XYG3|C4XYG3_CLAL4 Mitochondrial import receptor subunit OS=Clavispora lusitaniae (strain ATCC 42720) OX=306902 GN=CLUG_00986 PE=3 SV=1

MVLSPVLSSLLCCNLCLQSLSWGLEAMYSRQAVSLPPDSAVNYFARYNAGNWIATASLQA

QGALVGTFWRKVTDKVEAGLETQLVASAKPIVDSLMGTPIGYEPVVEGQTTLGAKYEYRQ

SVFRGQIDSTGKVGVFMEKRVLPTVSILFSGEIDHVKNASKLGVGLQFESAGSEQLMLMQ

QGLVDANGNPVPGAPQL

>tr|C4XYG4|C4XYG4_CLAL4 Inheritance of peroxisomes protein 1 OS=Clavispora lusitaniae (strain ATCC 42720) OX=306902 GN=CLUG_00987 PE=3 SV=1

MASDPFPDEPVASHEKAPEKKKKRRPRKKKNAVTKDVQSKSADIALDPPPLPEQSVPMSP

RKQTLLRHQKSNLSEDEPLFLSEVKELKKNRRPLTQNHMPRSENEKFNVLLASYPMDDRT

TIFRAPFARIVVYHEQVPDSGINASSGTLLGHGEFTIFQLHNGDITYLACGPSFVYPLLR

KLKILRVSRSHFILPLVNPQRYWKMEVVSQEDAVLEELEKVFQKFVNYTSLFLVSSPVDD

GEKYEKEKESADSHAGHVQLSIPDMFPLRGSNEQTENPISPHASSPNFQAKGNQFTPFFN

NIPESPPSVPVSPNQFHLYDSKSPISPSRSPNLEHDHFGFLSHPNAYMPIQSKLTKPLVK

KANNSQIANPYHHTSQQSDSSSMDSLLDEYEENVSMTRSINYNGSRMPSRTASFVSISHH

PLNNYARGNVLILPERSVAGSIIGDKNKHEDDGFFPTTSLSQYNRNRRNSQNGLSRRSSV

SELYTSVSNWMDPSTNGPKLSHSRSNYTLASRQSNRPINVKDTYQDIYRSITSHNVAALA

GREKNSEALPEPKYPSGDSYYSKLLVTDQFSRKPVSTKTQANTKSTSERLNRRSSRISSS

DVYNLISRKEEKIEASSGIGRFFGW

>tr|C4XYG5|C4XYG5_CLAL4 Rhomboid-type serine protease 2 OS=Clavispora lusitaniae (strain ATCC 42720) OX=306902 GN=CLUG_00988 PE=3 SV=1

MSTLTWESSIQRLKNTPALSVGIIIFSFLFYVLYPTDSKSLLLLPSSPLDLNLNAISFYI

FPHVNIFHWLINIVTLFPLLSRYERFHGTVYTGVTLNLLAVVTALQYCVVGLFLYPSDAV

AGLSGICFSFLTYFCYKEHEMKPVIMSLNFAGHELQVPTIYFPFFNLFLIALIIPSTSFF

GHLAGIGAGYLLAMGKLSVLYPPAKIILAIEKFLAPGIAKLQNIVLYIREEDAASERSVT

YRPVLSGDLESNHSTSETPIEGFERRLGT

>tr|C4XYG6|C4XYG6_CLAL4 Mediator of RNA polymerase II transcription subunit 9 OS=Clavispora lusitaniae (strain ATCC 42720) OX=306902 GN=MED9 PE=3 SV=1

MPKGHSSWCTGSSSPLGSHQHFKYLHTNLPTADGVMASNGSSKSPLIEDTSMEPYTDPLQ

KLADIELLPDLFALMQSLENGEIQAKDFDNNAGAIRLKVSNIWSYLHEVDGICETVEERE

KKIASIRHCNSEKIAFLKSFQEQVVKRLSKEDTA

>tr|C4XYG7|C4XYG7_CLAL4 non-specific serine/threonine protein kinase OS=Clavispora lusitaniae (strain ATCC 42720) OX=306902 GN=CLUG_00990 PE=3 SV=1

MPHYHPMKTPNVMDLILNSISKCFPCCPDISSPQISVNGTKYKIIRLLGEGGFSYVYLVS

QKNNASILYAMKKIRCPYGSSDATFRNAMREIKSYHRFAPAKSPYIVHTIDEAIVSEPDG

SRTFYVLLPYFKHSLQDIINYNVLNGVTMDEEEVLKIFIGVLSGIQTMHKYKKTGSDPGE

EDSQNEQDAMLPMGLDDDTGAMESTEMSELCPYAHRDIKPANVMLSAEGLPVLVDLGSCM

KARVSVKSRQQALALTDFASEHCTLPYRAPELLDVATNAEITEATDIWSLGCLLYCCCFG

FSPFEKLEMEQGANLSVAIAQAKYNIPQDIKGYSPEIMDIIRSCLQLDPTKRPTVDELLE

KCLALQGM

>tr|C4XYH4|C4XYH4_CLAL4 Histidine kinase/HSP90-like ATPase domain-containing protein OS=Clavispora lusitaniae (strain ATCC 42720) OX=306902 GN=CLUG_00997 PE=3 SV=1

METSRNTHTRPFLQLYNRQLSHSGFLFLFNLAKSNLLKMSDTKVETHEFTAEISQLMSLI

INTVYSNKEIFLRELISNASDALDKIRYEALSDPSKLETEPELFIRLTPKPEQKVLEIRD

SGIGMTKADLINNLGTIAKSGTKAFMEALSAGADVSMIGQFGVGFYSLFLVADHVQVISK

HNDDEQYVWESNAGGKFTITLDETNERIKRGSIIRLFLKEDQLEYLEEKRIKEVVKRHSE

FVSYPIQLVVTKEVEKEVPVEEEEEAKDEEDKKPKLEEVEDEEGEKKEKTKKVTEKVTET

EELNKTKPLWTRAPSDVTQEEYNAFYKSISNDWEDPLAVKHFSVEGQLEFKAILFIPKRA

PFDAFESKKKNNIKLYVRRVFITDEAEELIPEWCSFVKGVVDSEDLPLNLSREMLQQNKI

MKVIKKNIVKKLIETFNEIAEDQEQFEKFYTAFAKNIKLGIHEDQQNRPALAKLLRYYST

KSTEEYTSLSDYVTRMPEHQKNIYYITGESIKAVEKSPFLDALKAKNFEVLFLVDPIDEY

AMTQLKEFEDKKLVDITKDFELEETEEEKKQREKEIEEFEPLTKALKEILGDQVEKVVVS

HKLVDAPAAIRTGQFGWSANMERIMKAQALRDTTMSSYMSSKKTFEISPKSPIIKELKKK

VEADGAEDKTVKDLTTLLYETALLTSGFSLEEPSSFAGRINRLISLGLNIEEDDEVEAEV

GATSTASTDEPAVESAMEEVD

>tr|C4XYH5|C4XYH5_CLAL4 XPA C-terminal domain-containing protein OS=Clavispora lusitaniae (strain ATCC 42720) OX=306902 GN=CLUG_00998 PE=3 SV=1

MKDSKGGFLDDNDKFGLQGPEEESQTLKDWKEKQKQDHIVKDLAPPLDLANAPKCFECGS

LEIDPNLFTNFGARVCRRCMREKQDKYALLTKTECREDYLLTDPELRDTSLLKRIEKPNP

HGFSRMQLFLRYQVEEVAWKKWGSSEGLDAEWEKRERVRLERKEKRYREKMKEMRKKTRA

EEYTQKLRNGESLGERHEHSWSAPLAVGDNEKMVRRRCIDCGIETEEIII

>tr|C4XYH8|C4XYH8_CLAL4 Aconitate hydratase, mitochondrial OS=Clavispora lusitaniae (strain ATCC 42720) OX=306902 GN=CLUG_01001 PE=3 SV=1

MPIRPSAPGEFFFSSQPFAKTSMLSASRRTAAKVPRSIRGLATSGLTRDSKVHQNLLEDF

SYIQYKKHLENVNIVKQRLNRPLTYAEKLLYGHLDNPHEQEIERGVSYLKLRPDRVACQD

ATAQMAILQFMSAGMPQVATPSTVHCDHLIQAQVGGVKDLARAIDLNKEVYDFLASACAK

YNLGFWKPGSGIIHQIVLENYAFPGALLIGTDSHTPNAGGLGQLAIGVGGADAVDVMANL

PWELKAPKIIGVKLTGRMSGWTSPKDIILKLAGITTVKGGTGAIVEYFGDGVSTFSCTGM

GTICNMGAEIGATTSVFPFNNSMVDYLNATGRSEIAEFAKLYQKDFLAADEGCEYDQIIE

IDLNTLEPHVNGPFTPDLATPVSKMKETAIANGWPLEVKVGLIGSCTNSSYEDMTRAASI

IEDAATHGLKSKSIFTISPGSEQVRATIARDGQLKTFEDFGGVVMANACGPCIGQWDRRD

IKKGEKNTIVSSFNRNFTSRNDGNPATHAFVASPEMVTAYAISGDLGFNPITDTLKDAEG

KEFKLAEPHGVGLPPRGYDKGEDTYQAPPADRASVEVVISPTSDRLQRLTPFKPWDGKDA

LRMPILIKAVGKTTTDHISMAGPWLKYRGHLENISNNYMIGATNAENGEANNVKNHYTGV

YSGVPDTAAAYRDAGHKWVVIGDENFGEGSSREHAALEPRFLGGFAIITKSFARIHETNL

KKQGLLPLNFVNVADYDKINPDDEIDILGLTELAPGKNLIMRVHPKNGEAWDCELSHTYN

SEQIEWFKYGSALNKMAAAHSN

>tr|C4XYI0|C4XYI0_CLAL4 Metal resistance protein OS=Clavispora lusitaniae (strain ATCC 42720) OX=306902 GN=CLUG_01003 PE=4 SV=1

MTYPRKPLVCGATAFLHPLVPTAENAFNPCFLTALVWLISAVIGVSGAVQLYSTWKKPVY

GNLSAKSTGFFHWFRCSLVFLHTLLVCSLFSSISVVERYADHKLIPFGTLALVSATIVTP

LHVIEPTRVTIPSGSLLCFWPVMAILELVLYFQDSSTQWPVLLNSKAPSFELLASITSIS

IFILEYSKKSWRPTHELLIHYHTSETLVASLHQPNFIDRITFSWMNNLITNSYKKQTLEV

DDLPEISGFATEDFTQKLQLHWGGVEGKKSKAHLAGSLLKAFGPLMLSCLLYDAADSLLG

FVQPQLLRLLIIFIGDKLENPQDNPVLKGALISASMFAVTIIQTALSNQYMLKVLEVGLG

CRSSLTSLIFRKSLRLSTNARAERSTGDIVNLISIDTPRIQTCAQEIGTLIIAPTELFIC

IYSLWQLLGKASLAGVVAIIVIMPINTVIVRFSKRLNKLQMKLKDYRNNITNEILVSMKS

LKLYSWEIPMLNRLLDARNNKELKNLKKIRIINQVANLIWVSLPFMVTLATFSAFVCFES

VPLTSEIVFPALSLLNLLSRPILSFPMLINYMTEASVALDRISDFLLEEEVDDKLVKFVS

GEYSEALRMKRISFVWNRPVENITEIREDMDFSPIRYALKDINIEVKKGHFICAVGKVGS

GKSSLLSAIIGQLDAVDAHNPFTKAAPIELGGTIAYCSQNPWIMNASVKDNILFGFRYDE

DYYNKTIDACELLPDLNVLPDGDATQVGEKGITLSGGQKARLALARAVYSRADIYLLDDV

LSAVDSHVGKSIVSKVLSRTGLLAGNTVILATNNIGVLSRADKIYLFESGSIVEAGSYSE

AVHNQDLPKLNQLLQESGHLDYSRSRTMSPPIENSDHESTETCVSSPTLRKEFPRKASVA

TFDWNPFKKNVSSRSQPTAEISAKGKVKWKVYWQYIKACSLIWTCVWLVVNVAATLASVL

SNYSLKKWADRNSAFGDNKGALKYIAIYAVLGFSTSILNLCKGVIFWIHLGIRGGQVVHD

GMARRLMKAPMSFFERTPVGRIMNRFSNDINKVDDALPRSFNSFMGVALKTIMTFIVVGT

AIPPFAFVAVVLFFIYGYYQKYYISVQRELKRLVSISRSPFYNSISRKIDR

>tr|C4XYI2|C4XYI2_CLAL4 Mitochondrial pyruvate carrier OS=Clavispora lusitaniae (strain ATCC 42720) OX=306902 GN=CLUG_01005 PE=3 SV=1

MAASAQHASKFQRFLNSETGPRTVHFWAPVLKWSLVIAGLNDIQRPVEKISGTQQAALFC

TGAIWTRWAGFVIKPKNYLLASVNFFLGGVAGYQLLRIVHYRTSLGDSPAQVFKYICQGE

PDAAAASKSS

>tr|C4XYI7|C4XYI7_CLAL4 MPN domain-containing protein OS=Clavispora lusitaniae (strain ATCC 42720) OX=306902 GN=CLUG_01010 PE=4 SV=1

MMSFISRPHILEHSTLSEMAPKLPPPPPPPKGNRSDGRRGPPPPPPPPNARIPPPNGRHV

AMPVRVPPPPPPPQNGIQRPAKMMKTESSKKSDEEEKSDTRAKMTTQQHLDSSFVNAKSG

KQNRGLVQPIKTELPPEHLRKIMINHGDLSSNKVASDKRSHLGSLKYMPHAILKLLENMP

QPWEASREVKVLYHITGAITFVNEIPRVIEPVYTAQWATMWIMMRREKRDRKHFKRMRFP

PFDDEEPPIDWMENIEDVTPPEPIQPDLEEDPRFSQIDWIFDDKPFIEDRNVVNGDSYKS

WNLDVATMATLYKLAQPLLPHVTDANYYYLFNKEAFFTAKSLNMVVPGGPKFEPLFKIDR

DKEDYTEFNSLDRIIFRVPIRTEYRIAYPHLYNSFVKNVHPCWFHDPINNFVVHRDDEAS

DSAFLFSEEYNPIVPHKFSTTKAESKLLDFDINEINGNINLSPFMATDNEGEELRIEPDN

AKAALDLWWAPYPFNRRSGKTIRAQDVALVKSWYKERPPSNVPTKVRVSYQKLLKTYVLN

ELKNPPGSKSGSKAANQKKVHLLKSLKATKYFQQTTIDWVEAGLQICRQGHNMLNLLLHK

RGLTYLHLDYNFNLKPTKTLSTKERKKSRFGNAFHMIRELLRIIKIIVDSHVQFRIGNID

AFQLADGIYYILNHLGQLTGIYRYKYKVMHQIRACKDLKHVVYSNFNNKIGKGPGCGFWQ

PAWRVWLSFIRGLIPLLERWLGNLLARQFEGRRSNDVVKTVTKQRIDSYYDLELRAQVMH

DILDMIPEGLKQSKSALILQHLSEAWRCWKANIPWKVPGLPKPIEDIIEHYIKAKADGWI

AIAHYNRERIRRGATVEKTVVKKNLGRLTRLWIKNEQERQNNFAKEGPYVSPDQAVIIFQ

TMVNWLESRKFNPIPFPPISYKHDTKLLVLALENLKESYNANARLNSAQREELALIEQAY

DNPNECLARIKKYLLTQRIFKEVGLEMMDHYDHLVPTYAIDPLEKITDAYLDQYLWYEAE

KRSLFPNWVKPSDDEIPPLLVYKWCQGVNNLEDVWSTSRGESNVMLQTSLSRVAENIDFT

MLNRLLRLITDPNIADYITSKNNVSLSYKDMNHVNQFGLIRGLQFGSFIYQYYCLAIDLL

ILGLDRATELAGPVQQPNPFLHFKDAKTETANPIRLYCRYLDKVYIFFRFNKEDADGLVQ

DFLRENPDPNFENVVGYNNHKCWPKDSRMKLMRSDVNLGKATFWEISGRVPSSITKMEWE

NTYASVYSPENPNLLFSMCGFEVRILPKCRMSEDSSSQEGVWDLVNQSTKEKTAKAYLQV

SREEVDKFQNRIRQILMSSGSATFTKVAAKWNTALISLFAYFREATVATESLLDILVKCE

TKIQNRVKMGLNSKMPSRFPPAVFYTPKELGGLGMLSASHILIPTSDLKWSKQTDTGITH

FRAGMTHQEERLIPTIFRYITTWENEFLDSQRVWAEYAIKRQEAAEQSRRLTFEDMESSW

DRGLPRISTLFQKDRQTLAYDKGHRVRREFKQFSLARYNPFWWTNNHHDGKLWNLSAYRT

DVIQALGGIETILEHTLFKGTGFDSWEGLFWEKASGFEDSLKFKKLTNAQRSGLSQIPNR

RFTLWWSPTINRANVYVGFLVQLDLTGIFLHGKIPTLKISLIQIFRAHLWQKIHESLVQD

ICQVLDKELEVLHIDTVEKQAIHPRKSYKMNSSCADIVLTSTYKWNVSRPSLLNDRNDSM

DSATATKYWIDVQLRYGDYDSHDISRYTRAKFLDYTTDSVSAYPSPTGAMVGVDLAYNVY

DVYGNWFSGFKPLMQNAMKEIMKANPALHVLRERIRKGLQLYQSQPQVALLNSSNYAELF

NDETQLFIDDTNVYRVTVHRTFEGNLTTKPINGAVFMLNPKSGQLFLKIIHTSVWAGQKR

LGQLAKWKTAEEVAALVRSLPREEQPKQLIATRKGVIDPLEVHMLDFPNIAIRSSELHLP

FASALKIDKLGDVVLKAGEPQMMLFNFYDDWLTSISSYTAFSRVILILRALGISQERTNM

ILRPDASVITQSHHIWPTLTDEQWIDVETQLRDLILNDYAKKNNVNVQSLTQSEIRDLIL

GQEIRAPSAKRQQIADIEANKKDDNGIANAQEQLTALKTTTHNVHGEEITTITTTNYEQS

NFSSKNEWRNRAIAANNLHLRTKNIFVSSEDFVDDDSITYIMPRNILKKFVQISDVRTQI

GAYIYGTSPEDNIQIKEVKAIVLVPQLGNSHSIQFPSKLFNKSNNEYLSNLELLGWIHTQ

NNDLNMLSPIDVTTMSNFHKNNEDLWNRNLVGMTVSFTPGSVTLAAYSLNDEGYQWGYTN

KDMISNAPQGFSPTFSKKEQLILSDKIVGTFLVPDDGLWNYSFIGPVWDPNGSFDLKIDI

PISFYDQSHRPLHFTTFSEIEGNELEATQEDNFA

>tr|C4XYI8|C4XYI8_CLAL4 Nitrogen permease regulator 3 OS=Clavispora lusitaniae (strain ATCC 42720) OX=306902 GN=CLUG_01011 PE=3 SV=1

MNYYLFSQVFTVVYTISLRSLRLFALLVHTFTPPKYSSTHTFDIMSYNLPCPSLMGIFFV

VSNHNGPHFVFHYPPELSNVTLQSAGAEESDEDFDDEDFDFEESIQEKESPNAWDVNHRE

YYLGTKKDITSFLDAQRHIRGQEKKKSSRPKVSNSKTSQSLSNSAPTSGDTSTVSAFSTS

NEPTSKQILGFEPEHLCEMLCPPKDLCNKRFELTLERVVFLGLPIHVFSNGSWRSKKQKA

KSNQEHQGNGYDSGSDDNDSKSSMHMFHMVFVMNPSEIERDYRIDEMFYYITSKIALVLR

SEQSRHDYVWNQVRNISRLKEEWRTESQNNPSMTTLSNFLLSKSSLCKLMADCYEAISSS

KVANLLINNKLRSFQIPIKLEFHSLPEMTVPFIPGSYLTSAVNQFGKTGLVSVGETTRYR

ANNLMNILLGGKSADGGDEFEDSNDSLDQEDKSNANDVIYFSLLLLDDPEAIIRDMKVEQ

QSGLATFVRSLRPVDSLVKVADRLSYASDSKSAISLGEVKSFALHLIYWRKARAIPPLNS

RSIYIVSPMAPLTTNLQSDIAKFKRDFTAVPSLPVFLKLLSTRSKKPRQFASIIPSRDHK

ETYLMALGWLIRYGYVTQLHTYIWLKVSRKIKMRVEEDMENELGNGTKRNKDTEYKEPPA

SDNKVGNHVTVDNSSKKVESASQQKLSGSIDEEIDNIQRRFEWSGESPDIIMEDDEDTIL

VDPGRASSIERRWINRIIHDECHLSSELTSIFFKVLKYMNGRNSLELLLLKENISRTDLR

KLLVAIEDHIISVRHW

>tr|C4XYJ0|C4XYJ0_CLAL4 ADP-ribosylation factor OS=Clavispora lusitaniae (strain ATCC 42720) OX=306902 GN=CLUG_01013 PE=3 SV=1

MGLSFSKLFAGLFGHKEMRILMVGLDAAGKTTILYKLKLGEIVTTIPTIGFNVETVEYKN

ISFTVWDVGGQDKIRPLWRYYFQNTQGIIFVVDSNDRDRIAEAREELQQMLNEDELRDAL

LLVFANKQDLPNAMNAAEITEKLGLHSIRQRPWYIQSTCATSGDGLYEGLEWLSTNLKNA

>tr|C4XYJ3|C4XYJ3_CLAL4 Rho-GTPase-activating protein OS=Clavispora lusitaniae (strain ATCC 42720) OX=306902 GN=CLUG_01016 PE=4 SV=1

MSFADSFWTYDYHSGHEVLFDVLFEGVKENEDFIQLFNRRMDSELQYGLSLENLPGQVRP

TSKRHTDDDYVSTIKNAFSKMNENFSRQGKYHIEVAQNIQGMVLDPFSKWCKEHESRVAF

SEATIAEKFKAYKSKKTALEKLQKKYFNKCRVLEEFRSRYTDEELAEIPQEDTSFDNSAD

NENISETENELNENTYTFAGAVYDYKNARKLLSDIISNIELTSHKVPILGTYHNVSSGSA

ITQWLLDNMPEYKGNIAKAESFGQDLIENNFIRLIGSMNASKSFINSSQFLYQWKPLAFE

FTKLYERDGSRANVAEPTTVLTKTNQFTNYFEDVKQAIGVASVDFNDKSQYPKLVKQVES

LDIAYFESTKDLDMTRCKFEETVMDHLAFMQKCELDRLRAVKKVMFDFLSIFSNKWAALK

GVSDELFVVEETIHPSSDLKFLIENYGTGRFEPRVTLYDNYYNSNIKQTFGVDLNVKARL

ERKAVPSIIQATLSFLDNAYPDLENDTERMNLWTEPVHLSKVHELRFKLNELTDAAEMKD

ILKKSEPKVVTNVLKLYFMELPDSIVPHSYFDLIMTLYQNYPAESQDKNVNKSRLTGLQN

TLSELPVCSLATLDAILTHLNRLVNIISSANEDLGSSLRLRLCKEFGALVLRPKRDNEGA

EGKNLHSNTVAVEIIQQEFVTDLFAHKDNIFGELRRRNSNKPSRASSAASGTGPRNSDNQ

PRVSDSTTASNGSDAAKLRLETKLKRAVTKAAGKNNLSSKGDNMSGPGSSSKTSSSKSPP

TTPVKSKSDLSSKSSGTGLKRSSSPNKKKLSSLIEKQSKSTGASTSSQTGNDVENETHDS

ISHENDMPDVARLTMESSTPKKGDEGASSESSDLPPVPPKKSSSTKEVIVVD

>tr|C4XYJ6|C4XYJ6_CLAL4 Zn(2)-C6 fungal-type domain-containing protein OS=Clavispora lusitaniae (strain ATCC 42720) OX=306902 GN=CLUG_01019 PE=4 SV=1

MEVHMNQPLLPVGLPSQSAFENNDTEKSTKKLQEVKVLADGNVMKLQKTRQRRILSCIHC

HSKKIKCSRVQPVCDNCNKLGIECKYFVNKRLSRGRRKNSSPSQTSAKSSREPTTSKSSV

KLEHNSPKFITSQDPGSIAAHSSTHLSDSIPKSRSPEQMKDDPITFPADAPLNYVSMTTM

KSGVSPPSAKVESIPPVALPSESTNPMLQAPVVNSVSNNITNNFFNTNYANNGGNQQKNT

SSEDLYSFNLSSFTFTSGQSLNGNAVPNKIFPVNSSGKFDQDSPRKSDEPYRSPSVGVLK

KTDSNSNINGILNGNSQSAITNFVDGPRSTVPVVTNISNNIKVATSSTYTLQNLSSDISD

TENSQLNNPSLHNSLNAYPSNPATTMNYLYGTNTYYESHNLLSDLINHLPGSKERSLELL

DRYQNSVHIIFPVMVNLPEFIKEHEKFWDKFDPNKTNSSSCFSPESHGDFNLLQFYTLYF

PILYAATISEFEEYDNLLLNQDINKYLKGFNNICQYYNYPHGLKSIPLLLGNVIIQSTSP

NPSTMEMSQIMRYAKFLQLHKDPVKSLHISDWKIVRFRRVLWWVIFGLDALSSHNFCLPP

VCKMDDFNVELPEAEDPVYDSSGHLIHKKLNVAILSMTIKFKYDRILSELVNQLHNALSP

NISTDEVERIKSMIIGLFHYIHQSILKMNEFFKANPPKTVQEVNLLHFIKNHSWSYVDRA

MMLLHKKILLNEPKGDAGSLEVTKDTPALSFRKGSGSLALSEYEDTFGRLQETNIIKNFD

NLSISQLKFNQHELFTYENMHNNLIPSILHNLNDFLKYNDFIKFGKFNWYVKRTIPLDSI

IMLFVVICVKFKYDFMTLNELVIYVNLINKALFILNRKYFKNEKYKRMLSLTNLTWEFIL

KKHNVVRVMNAINHRKDVKVEFFDYQVSGYMNLSELFNVMDVPQPALICDGENYGAIKDN

DDVSSFNEPSNRWADQELMGQSFYSSNILKQLDDRSDTDLQGQKLELLQLNEKIYYDLRN

NFVDIIDYCTFYSSLENVLHELMDYINSSGNIAH

>tr|C4XYJ7|C4XYJ7_CLAL4 Major facilitator superfamily (MFS) profile domain-containing protein OS=Clavispora lusitaniae (strain ATCC 42720) OX=306902 GN=CLUG_01020 PE=3 SV=1

MKLVGRTNTFGLSGRPLRLAVTIIATTGFSLFGYDQGLMSGLITGTEFNKEFPATSLTRP

HVDKHHVTVIQGAVTACYELGCFFGALFALFRGERIGRKPLIFSGSLIIIGGTIISVTAF

REHWGLGQFVIGRVITGIGNGLNTATIPVWQSEMSKAENRGRLVNLEGSVVAIGTFIAYW

LDFGLSYVDNTVQWRLPVAFQIFFALILLLGIINLPESPRWLISQDRKQEAMEVLSKLGG

LPIDDDEVYAEVTLIQDAVNRFSRSQVGFKDLFSGGKTQHFQRMLIGASTQFFQQFTGCN

AAIYYSTVLFETTIFHGKRRLSLILGGVFATVYALSTIPSFFLIDTFGRRNLFLTGALGQ

AISFTISFACLVDPTTQNAKGAAVGIFLFITFFGFTILPLPWIYPPEINPLRTRTVATAI

STCTNWLTNFGVVMFTPIFMDASAWGAYLFFAVLNYLFIPVIFFFYPETAGRTLEEIDII

FAKAHVDGRQPWRVAATLPKLNYKQIEEHGNQLGLYDGDFEKEGFEVKEDVSSTASDDRG

EEGVMTTQV

>tr|C4XYJ8|C4XYJ8_CLAL4 ADIPOR-like receptor IZH3 OS=Clavispora lusitaniae (strain ATCC 42720) OX=306902 GN=CLUG_01021 PE=4 SV=1

MSTTIVSGVRNRNHTNKLPPQVTETQTSESLLIEKLDAFLSSIESRLERFERYFQVVGAG

ASETLVESEGNGKNPTQSHNRRGSTASLSSLKTLSATHMNQVYEQLRSVKDHVLQTSITN

LEYLYKTLDEKYNDLFSNDESSLILPEEDVTNEALSTNIINTIQFFEQKLSHIDQFIKSR

TPAATDNYDHDSKFNRFRFFNFNKALKEAEEKYLHYYQLPLSWRENRYIIHGYRFSLQHK

DILKSMFHFNHNETGNIWTHIIGTVLMLYLGLVHFPKTDVFAANTWVDNMVMYVFLLAAI

ECLVSSVLWHTYSCFAHLQIRNRFACVDYTGITVLITCSVISAEYCALYNSSKLLYLFVG

FSIISGLSGLLFKLVAIL

>tr|C4XYK6|C4XYK6_CLAL4 Cell division control protein OS=Clavispora lusitaniae (strain ATCC 42720) OX=306902 GN=CLUG_01029 PE=3 SV=1

MNFLRRRKKSDDDDLGEETVSRIQKSRKPPNTAFRQQRLKAWQPILTPKTVIPFLFLLAA

IFAPLGVAIVYYTYNVEKLQIDYSKCATLATDSYAKVPSKYTNWHFRHKNTDPDFQWRVI

NGTDADGDRTETCFVKFNLPRDLKPPIYLYYRLTNFYQNHRKYVDSVDPKQIAGHALDAD

SLSTNCKPLRSVGDGSDEKVIYPCGLIANSLFNDTFISPVLLNSKTGSDNETYVLTSKGI

SWASDRKHQFKKTTYKPEDIVPPPNWAKMFPQGYNSSNIPDLSQWEQLQNWMRTAGLPTF

FKLYSKNTTTTFTSGTYEIQIGLNYPASMYGGTKSLVITTNSVFGGRNMTLGIVYLIVAI

VCLVCAIGFFSQHLIKPRRIGNHNFLQEGGAEQAPAHFREQL

>tr|C4XYL2|C4XYL2_CLAL4 DNA-directed RNA polymerase III subunit RPC3 OS=Clavispora lusitaniae (strain ATCC 42720) OX=306902 GN=CLUG_01035 PE=3 SV=1

MTSALTESAKTQSPFSYLYTRVALNHLGETSAIIISTLISYGRLTAKDISIKSKLTLKQV

KIALVSLIQLNCIQYWKENSSKSVFYTFNDRGIITLLHAGDIIARIKSQYGEKAAELVQN

IIENGNITVKDFISTLSDEQEKYDNMAILVKLFNDGWLHRLQIFNYHPAEDLWNKMYQET

LKNTPRSATTSEIKRVAEATEKTKIKFNDLFLSGNESKDVFIVESGIHQLKSSITLGFNL

SRFEKHLRSRALSDMAKSRLGLLTGFIYEQCCSLIEQKSADLRHRYQDISGLITNPEEER

VFLSSVENALVDSKMTVFTIRDLMRHLPPDLDLRNSILTQNFLKPAKRVNVNGTDQPHKK

IKLEDGSAITEMDIEFESDFHAENGDGPLSMTLVAEHLRLLTFNTVPFLFEVAPGSYTIP

FLQLSKHVKQFHYEALIKTTMGTNALRILKCIKAMKLVDEKAISNSVLLKERTVKNELYH

LINMNVIEIQEVPRSADRAASKTFYLFRHKDASSYRFLSHALAFNMGDILTNIESFKAEH

KILLEKCEREDVKGHEEELLLESELKTLRDLQLREISNIGRFNRVKWLFTVFGTL

>tr|C4XYL4|C4XYL4_CLAL4 Autophagy-related protein 13 OS=Clavispora lusitaniae (strain ATCC 42720) OX=306902 GN=CLUG_01037 PE=3 SV=1

MSDQQSIQKQNAKVIQVFQNFFTKTSSIIVSARSASDSPSSTADNTRSFKINKWFNLNIA

TPTDEWLRSEIKLWKSQAEASLLPPMIIETYLDLRQLNAREIVMLEDEHGKLWTVAEGKS

KKQEVVIERWLIEFDQLDVLDSGLDELPLIYKQAIVLLRVVFTITRLLPAFKLKKIVSKV

SSKNLALCNRLIDGKQPISSKGRIGLSRSIIPSQHLSGSHMTQKSFSPIRTTLGSLRVSV

AYRNHFNFKVVDHEERLSDQFLLTDKEKADEAEKLQNANSQGMVSRTNSISYSDNETTNH

IGAARIDRRHEDYLHRTEDEEKFSVSPCTSIQQDANQRPKIGITGTGAVRPSIQPFKVGS

ISTSPPPSGLSGTPTSYASGSIERRISITSHRSGSNASLAALLRNPKGSLSSSNTPTTIA

ISNSQNNSNAMSFPRSISSSHGSHLQSDEHYGENVSSTPRFSSSFGSRQSRRFSGASGRN

NDTNASLLGTSMESGSSGALLSGLYIDDDISSFVRMIDGKSTLKLTNSNTDSKTVPHSSD

SNSHLEALSRFQLLKSQHQQLGDSVSASLILHRNRSGSRESHGVSGSSKAASHKSSHSSS

PQPSFSPNSYDNGPLPSISSRLHTSGIDSRVGSASPRSLMGSKGSYTSPNLSYFKPSTNK

LAASPSTATTIAHATHHPGTVHKEGIKGLATSPSIYSEAKHPIRYEDVFEDDEDAGGFYH

SPEGGKRHSQGRDNDMSYDNDNDDLLFEMTDTK

>tr|C4XYL6|C4XYL6_CLAL4 SWR1-complex protein 4 OS=Clavispora lusitaniae (strain ATCC 42720) OX=306902 GN=CLUG_01039 PE=3 SV=1

MSANDILDVLNIKRDDTPQPAKKKQKVEYAAPQKGAMARELYNLLGPNTPPVSLSLGANK

ARDRKQKVSPWTKMPFKPNKKNEHAPQLYHWQKGSEELLKQESAGKPYFFDKFSVSLDIP

ELVDEATYDHFMEEILQEEKRRNEQELKDKEEEDSVPSSETTTDGSSEKVATLKNEQAVK

LSKDMKDTKSNGRTDGKVKTETDTPGTQQKTRGLNKSGDGGAEEEKEAKEGDEIDEDEEE

DDEDEDEVKDNEGSQEEKESDKKEKDEKARQTKVANSSKATTDVQDNNSITDKHTNPPWS

YKETQLLFDYCKVFELKWYVIADRFPYDRTMEELKEQFYRISAKILNHQGNANPHLVESL

ESYSKDAEIDRKQYLESLLKRTPAEIAEEESLVIEARRFELAAKKMLMERSHLLTLLDSP

QSSQSVQQYLSSSGMTNLYNKLMIMDKQQKKKMSNKSNSANTDPVPPSIPVAASSSSYKK

DRGFQTHLQQYLSGLVKQSSTKGEPSPIQQLLSKRLTTKEEEAYGLHYHANERLTPGVIL

RSTQRLPGLQQRQSVFKSVNSMLQELDIPTAGSTTWKPVMPTRKTMAKYDELIRAVVALL

DVKKGKDKLEAEIELIKSQRGLQ

>tr|C4XYL8|C4XYL8_CLAL4 Nucleoporin OS=Clavispora lusitaniae (strain ATCC 42720) OX=306902 GN=CLUG_01041 PE=4 SV=1

MDFKNLSYMQLSCVQQDEKVCTMDNRRVMKAYRDRVKEKVLPYPSRAGLFSRVKRYFSGS

AIPNNRQHEQTRRPTQVAETAANKSGYILPESSTPANNTMIADQSTNRVLSSFFQEKGDQ

PLSQIEYEGVMSLLEKSKASITLPLPDSPSEKKKTSGADVSSSSTVQHNHTFAPYSQTTL

RNTSMYEGNSTSFATPDYKPVYHTFSDNSRANVSVKRVYQFSGLPSPYRTRIKAPNMVSR

KARRIDTVAQSGSNSTLLPSNQTVDSTSTKVMSNTANSLLSILGGNDANEKEQSQEVYRP

LHNPYATNKRRFNVEEERVTKKPVLGADDITKTVSYNKSEDLPQSIREESSSSAPISQSA

VPRSVPDTLSFSSSNEKAQKSMAEAKSAKEAAPAEETGLESGSPDLSQKGFKSMQTSTES

ASPMPESKPQQSLFGSNSNGPFLATTNGTKPSFNFGATTKPTSKLEKVKNPSFEPESNAQ

KPASASNGFSFGAKKDQAPLQSFSFASQSTKPNSTSNDNATSTLKFADTNKPEQPSNGFN

SAKAQSSQAPTFSSANGKSVNGSGKRESFEFSFPPVETFTANVDEAQVEQYKSLFEF

>tr|C4XYM0|C4XYM0_CLAL4 1,3-beta-glucanosyltransferase OS=Clavispora lusitaniae (strain ATCC 42720) OX=306902 GN=CLUG_01043 PE=3 SV=1

MKSYIKGKGWKFPVGYSSNDDEDIRVAIADYFSCGELEERADFFGINMYEWCGDANFQSS

GYKDRTEEYKNLTIPVFFSEYGCNAERPRKFTEIGTIYSDEMTDVWSGGIVYMYFEEANK

YGLVSASGDSVSTLEDFSYYSKEMNSISPSLAKTADVGSSSTVTLSCPASASTWKASPSL

PPKPDQDVCDCVSSSLQCVLSDKVDEDDYGDFFGTLCGLVNCSAVSADGDKGKYGAVSFC

SSKDKLSYLLNMYYEDQDQHSSACDFSGSASLRSTSSLPKSCSAMVSSVSAGAVVTGSSD

SSSGSSSGSSSESGSGSSNSDSQSSSSSSSSSSKKSSGYVAARPASKGELAAIVAMALCF

VGGFSSFLF

>tr|C4XYM1|C4XYM1_CLAL4 Protein PXR1 OS=Clavispora lusitaniae (strain ATCC 42720) OX=306902 GN=CLUG_01044 PE=3 SV=1

MGLAGTKVKQRFGLDPRNTAWSNNTERFGHQYLEKMGWTPGKGLGLVNHATTTHVKVSIK

MDNAGLGAKLAKKTKKDEFDSGECAGLDVFQRILGRLNGKEEQVNNELERKRIDNFVNGK

WGMHFIRGETLKSTWDADCKKLVGGSATGTKRRPSMSEESPSKKRKAGTDLDVKKKEKKE

KKEKKEKKEKKEKKEKKEKKEKKEKKEKKEKKEKKEKKEKKEKKEKKEKKEKKEKKEKKQ

NLVATEEVTKASMLQPRDNKDKEIATRLSARAKWIRQKRASVMDAKALNEIFMVTK

>tr|C4XYM4|C4XYM4_CLAL4 Protein-lysine N-methyltransferase EFM5 OS=Clavispora lusitaniae (strain ATCC 42720) OX=306902 GN=EFM5 PE=3 SV=1

MDSDDEPLTLSSHALAALQQFKDEEETRKKQFEKLYQESEDRFEDQQVSIDDFKEDWQLS

QFWYSDETAEILAKALLEGADEDTIICIASAPSVYAAIRKLPKELVPTKHIYLLEYDKRF

SVLAGKDHFFFYDYNEPDNIPSVIRNKCHRLLIDPPFLERECQTKSAQAARNLLSSDDGS

KTKNGDRKFKLISSTGERMRDIIKETYPETHLTSFLPEHKNGLSNEFRCYASFECPYWNF

DDNV

>tr|C4XYM7|C4XYM7_CLAL4 Golgi apyrase OS=Clavispora lusitaniae (strain ATCC 42720) OX=306902 GN=CLUG_01050 PE=3 SV=1

MITAYISTCIDVVTKTRGVYFSSFAMADDKYGIVIDSGSSGSRIQIYRWEDPSKKKEHAS

PEELASPPKIIQEDGWSHKISPGISTFAEKPSKVWSQHYKQLIDFASKVIPANKISETPI

YILSTAGMRLLPKSKREKILKKTCSELQKNTKYFISDCDEHIQVIDGATEGIYGWLALNY

LMGQFNNFKPDQADHESIGFMDMGGASTQIAFVPSSQEEIAKHDEDLSTVVIKNINGDGQ

VWRVFVETWLKFGANQARARYLKNTLALSADSHSKGKAKVIQDPCMPSGAVIEDYKFENK

EYIIQGTGNYESCLKDIFPLLMKHLSCKDEPCLFNGVHAPKMNFEKDKFVGVSEYWYTAN

DVFHSSGEYNFHSFNEKAKAFCESSWDDIMKNSKNGEYSNLPDKVLLEACFKASWVINVL

HEGFELPRLGIEVKEPSESSATKELDNAHVPLKIADSVNGEELSWTLGKILLVASSQVPS

NDEVHVGIKPSAMSLSTSSDEYDSDEEFGPSMNFVYSFLFLLLFVLFFYRFGNSSLRKIG

KLLRKQGNVKLPSTCKAVLNRVKAHSPSSIRYRINKAINYMELQEQDDINLDLEEGSMIS

SPSSQPANPPDISVLRTRSTINLSDLDEGNSPVDFMNKPFVNVKKSNSFYTHRGDSRDSL

SRMSSSASLSRGKHT

>tr|C4XYN0|C4XYN0_CLAL4 Translation machinery-associated protein 20 OS=Clavispora lusitaniae (strain ATCC 42720) OX=306902 GN=CLUG_01053 PE=3 SV=1

MFKKFGKDEIHSRSNIKSSVQRGLKSNFVSQFEKLEPVIDAIIPKKSQAILIKCEGKLSL

YSVDGEVVLFQHFDDLIPDLHIVHKYPDCFPKIQVDRGAIKFVLSGANVMCPGLTSAGAK

LPDENLEKDTIVTIYAEGKEHALAVGKLTMSTDDIKSINKGVGIELFHYLGDGLWHLDES

N

>tr|C4XYN3|C4XYN3_CLAL4 DNA ligase 4 OS=Clavispora lusitaniae (strain ATCC 42720) OX=306902 GN=CLUG_01056 PE=3 SV=1

MSVSTSFLDNVKEPENFNPEPAPFDLIISELVLKLESTNHENMAPFGTVSAKKVNIIEEF

IKLWRTHFGVNISPALSLIFPNKDSRKYFIKDVALTRLVIKLLRLQQGSTDYSIIKNWKK

SYQQKIQLTGSNEHRSTGDLPLIIARIMSRRRDPNSVVETSVTVTEVNDVLDKLSQNTSA

SDQLEVLDPFLDRLTITEVRFFFQIILKESMLSFFERSFFIAWHPDAFNLYKVCNDIKKV

FWLLTDPEKRLTPQQLCVQPMYSFIPQSSKSLTLSYENLCKKMKSGMSLSDKDPKLVNLY

KDEGIQDHFLIEEKIDGDRMLMHMVEGNFKWHTRRRRDYTFIYGENYHLGSLTKHLTGAF

HKNVKSIVLDGEMVAWSKDRQLVLPFGTLRSAAVQEALRQFDVVDVYEGNNSWPLFIIFD

ILHLNGQDLSNLPLFYRKDLLSKVVTNVPHRFEILEWVKASTPDDIKRNMQRIVSERNEG

IMVKSFLSKYRVHSRDSTWIKVKPEYLENFGENLDLVVVGKIGRIKTSYICGLRDEEEEG

CFKSFCMVANGFSKAVYRQIESKLYNFWVNYKERKPPEDLVKFGTRKPDFWINPANSIVL

EIKARSIEVTAETPYAAGSTLHNLWCRAVREDKGYEECITLQQYQELKMRYSTDVFKQQT

VNRARKRGFENSIYDRFATKKRQRIVPKSDVFQGLHFVVATDARDLQSHELITKEEIERR

VKEYGGSILLHPKQENIQARNIIILGQYVTPRISMWVKEGFDIIRPQWVFDCIRYHTILG

IEPQHIEQSGNAKLRRSASETTDKYGDSYVISFPSSKGLFKWLKKIPMSQQLSAGCLEDC

RKDFYNAEKLSNMSLFAGSHFYVLEGGDNSTDNAIHKQAQRRILRFGGSLSLDATNCTYV

VVPDEVLTNKKLQEAVRITSRKIAEGYEEGKKIPIIVMQSFIDESIHQAEIVASEDFRVV

I

>tr|C4XYN4|C4XYN4_CLAL4 Amino acid transporter transmembrane domain-containing protein OS=Clavispora lusitaniae (strain ATCC 42720) OX=306902 GN=CLUG_01057 PE=3 SV=1

MANFAGEDFTDEDDDESSFDEGYEEVFDEESSLLHERRGPQKRKKPKKHEQKGTASQLKV

FFLLFKALVGSGILFLPGAFMHGGLLFSTVTMVLFGVLTYACYVVLIKSKSVLGKSSFGE

LGYLTYGNPLKYCIMVSIILSQIGFVATYILFTAENMKSFIHNSLHISIEKSTLVIIQCI

LLIPLVLIRDLTKLSFTSLLSSTFIVIGLLIIFFFCGEQLAHEGLGPNIVQFNGRTWSML

IGVAVTAFEGIGLILPIQASMAQPEKFPFVLSMSMFVITLLFVSIGVIGYTSFGENVQSI

IILNLPSGNAAVQSIMLLYSVAVFLTGPLQLFPAIRIGESALFNSRLFLTKEQQSENNGK

LMQNSGKHNPHIKWMKNVLRSLSVVLISTVAYLNADNIDKFVSFNGCFACIPLVYIYPPM

IHLKTYNAEPQQSKIIKVFDVLLIIVGTLAMVYTTYQILFSI

>tr|C4XYN5|C4XYN5_CLAL4 Zn(2)-C6 fungal-type domain-containing protein OS=Clavispora lusitaniae (strain ATCC 42720) OX=306902 GN=CLUG_01058 PE=4 SV=1

MINEEVGAPSASSKRGKTAPKKEMPLSCQRCRLRKVKCNFAHPCSSCVKLGVECIQVPND

MRKKRPPANYISSMEKKIDALSKFFTSMQRVPAEDRNKFFEANATNIASYLNEDNTQAKA

SSADGKVPSSGKKRKTEDVRSTPSPTPITKIRPVYGPTSVYDNYTDHHRPKTQSLRKESR

EMTVLQNLSKDPDVLHCLKLFFTWQYPDHNMFVFREAFFIDFFNPKPNSLYCSSVLVYSI

CALGSRMSDVDTIYSRSISYYNEARSFLLSNLEHPSITSVQSFLLLAFYDICNGNNSTGW

MLSGDAMRMGFDLGFQLNPEVWFLKRHGTDFKPLDMAIRSRIYWGCYMADHFISLILGRP

SLLKSSDASIPETSDLPELEWIDDYKYIPENVTNISDPLKNIINLISISDKMLNDIFTRS

DHESVSHKESEQNEDLNLVSRLNKLFSYNEQIMQWKANLPQDLNWDQESLKNTSDNPTIS

VVRYYYYIVILCLNRPFVGIVRDFKDKEHLSPSVFCHNAIEDLYVAIQRFESAHGLRRAS

IFIVYCSILSISVILLTATTEQLQDDKKTKLKYFLRVLAGCSKT

>tr|C4XYN6|C4XYN6_CLAL4 Pyruvate decarboxylase OS=Clavispora lusitaniae (strain ATCC 42720) OX=306902 GN=CLUG_01059 PE=3 SV=1

MSDSISLGTYLFARLNQKPLGLNSIFGVPGDFNLTLLDKISEVKDLHWRGCTNELNAAYA

CDGYSRVKGNGSAEGLGFGALVTTFGVGELSALNGVAGSYAEHVGMLHIVGIPSVDAQKN

QLLLHHTLGNGDFSVFHKMSSHISGTTGVINDPALAPDVIDRVIREAYINQRPSYLAFPA

NMVDVPVPKERLNKPLDLNIPKNDPELQEEVIEEVIDKIRKAKDPVIIVDACCARHNANK

EASELIKISNFKYATTPMGKGTKDIDEQNPKFTGVYVGSLSYPHVKEAVEKSDLVLSVGA

LLSDFNTGSFSYSIQTKNVVEFHSDYTKVKGATYPEIRMKELLGKLIQSQALKQVFKEHL

HQSNCERTFTTPDAQKLRGLK

>tr|C4XYN7|C4XYN7_CLAL4 Alpha-1,3-glucosyltransferase OS=Clavispora lusitaniae (strain ATCC 42720) OX=306902 GN=CLUG_01060 PE=3 SV=1

MPDTKSVNHSDGDENTRRYSLFNIWCASLALKALLAVGYHSTDFDVHRNWLAITYNLPVA

KWYLENTSQWTLDYPPFFAYFEWFLSHMVPNFVMEDGCLSIVEKGLYSLPTILFQRLSVI

ASEVVLFVSLQWYLDTSKSKREASRAFVVASCLVLSPGLMIIDHIHFQYNGMMYGILVLM

INSARLERYLLCGFWFSVLLCFKHIYLYLAPAVFIYLLRAYCLNMSYDTKKSFLHNAFKM

VRWMNLIKLGSIVVSVFTVAFGPFIYYNVIPNLLERLFPFSRGLTHAYWAPNIWAIYSFL

DRILIQVYLRIPLLRVPLHKILMFDESNLTNTKMLSSSTRGIVGDIEFLILPTITPKLTF

YLTLFYQTMALIPLFLQPNFQRFIGALTLCGYASFLFGWHVHEKAILIVIFPITFLVTRD

KRLLNPFNLLVSCGYVSLFPLIYTCEEWLIKVTFTLFWYIIYYFQFRKVVRVPNNVHKEA

SFFSVERVTNLYILGLIPIITSVTLIDLFQHKFEILQKLEFLKLMLVSVYCGIGIICSWN

GFNWLYFVDESIWDDIDS

>tr|C4XYN8|C4XYN8_CLAL4 Mitochondrial fission 1 protein OS=Clavispora lusitaniae (strain ATCC 42720) OX=306902 GN=CLUG_01061 PE=3 SV=1

MFRRSYPALEELNQNISPEQIRVLKDQLESEHPPTPQSEFNYAWALLKTDSLASQKEALD

ILAVLYRDVPSLRREALYYLSLGSVKIGEYSNARRYAEALLEKEPDNTQFKALKQAIDDQ

VTQDGLIGLGVAGGVLAIGLGVMGALMRKKR

>tr|C4XYP6|C4XYP6_CLAL4 ATP-dependent helicase OS=Clavispora lusitaniae (strain ATCC 42720) OX=306902 GN=CLUG_01069 PE=4 SV=1

MTKPQETPRKENVLQVPSSSPINVRSSPVSTSAMPPSINESRSSDERHKEEVRKRFYSHK

NWPIISKRFYYLNEGELFKAMVKGKGNLRDISQWLAENHNLGSRMKQFDDAKRKMQAADA

ENRKQVLSKFSPNAVSNHQDYPVSSETKVEVGAPVENATRESPATPIKEEYADEDASPIK

IAGKARQKRSLSAIDTVKAPDTKVQLSKPKVSILEKYRNRQQMRIDSIFKNPPELEDRKK

RRLVRANDMYVATPEPSSASPPISLGNPGALTSAFGYSDDSIPRLKNNAPQLGGTGDIDE

LERLEERIKENKRKKKGSKVVDSDEELEDEVFSDDMSAEDDEGMYSTGLTSIDGQILDFL

NKAPINDIVEICNIQPNVAELLISKRPFNTIYEVAEDRFEQDTPEPEVKKRNQRKTLGMK

IVESTEFSLKGYRAVDSLVKKCSEYGNLISRQMSRWGVKVTGEGELSMVDVEVDDLEKPN

NDGDDYDDDDIGSKKRNLPYVKHKPTLLAPEIELKTYQQVGINWLNLLYHNNLSCILADE

MGLGKTCQVISFMAYLKATSEKKRPHLVVVPSSTLENWLREFNKFCPDLIVQAYYGSQSE

REDLRYELQETEFDVLVTTYNLATGAPSDFKFLKNQHFDMIVYDEGHMLKNSNSERYNKL

MRLKADFRLLLTGTPLQNNLKELVSLLAFMLPQLFVEKREDLQGLFNKKASVDSTKDYNP

LLSQQAINKAKTMMTPFVLRRKKAQVLKYLPKKHHDIVRCPMTPTQRAIYDEYIEKGKST

KLERERRKQLTGKDAELARKSPVASSSNVMMSLRKASMHPLLFRKNYTDDMLKEMSKRIM

KEPEYVNASRAYIEEDMSVMSDYELNALCEKFPNTLSSYVLDKEKWHDSGKVAKLLDLIK

QTIMKKEKILVFSLFTQMLDILEKVLSFANISFLRLDGQTSVDTRQDIIDRFYEDEDIPV

FLLSTKAGGFGINLVAANNVVIFDQSFNPHDDKQAEDRAHRVGQTNEVMVTKLISENTID

ENILMLAENKLQLDQSISNEPNESKFEEKASSMFEKLLFGG

>tr|C4XYQ0|C4XYQ0_CLAL4 Ribosome biogenesis protein NOP53 OS=Clavispora lusitaniae (strain ATCC 42720) OX=306902 GN=CLUG_01073 PE=3 SV=1

MHPHLEKTTSGSSPNISTPKMESKPKGQPSRHGKKAWRKNIDVDDIQAGLEAKRENEVLL

GDDSDFIIDNEGDSKSKTPAFIRKTSEILANKSKVPSVSSRKVKHKVSKSQANRLMALAG

RSVTTSKVQARSEKEGIIRASSLDVWGTASEEELPEVYKKTPFLSYTAAKKAPKTLSHQP

VALETPAPLETVIHAGKSYNPDLKSWKDLINKEFNLENSIEMKRQEMEEHQKRIQYLIAT

LDDKEFEESDDEEGKEEEGAGENEDEDDSYKLSLNERTELKIKTKTRRNKEMRHKQRLEL

EKKLKDLKKQIHDLSNLETIESEVEKKLSSVKKRSERKYRRHGKHEVGSRMRGVSMMTSI

ETMNQKYSI

>tr|C4XYQ1|C4XYQ1_CLAL4 mitogen-activated protein kinase kinase OS=Clavispora lusitaniae (strain ATCC 42720) OX=306902 GN=CLUG_01074 PE=3 SV=1

MSDPSPMFKAPIKRSYNPQSPSIPSPISLGHGQNDSSDTNLTSDSHNPAPETVVPLPFTV

LPQKQRAPRRKPPPIDFTKIDGSTSYTPDSHPQPRSEVTNTSPESQQDSPAAATVSNTPL

SGQSIDHLTPGDWNILANTDQIVELSKLGEGNGGCVSKCVLRSRSSVFALKLINADPDPN

IQKQILRELQYNRLCNSPNIVKYYGTFIVQKSSMIGIAMEYMAGRSLDAIYKRVIELDPT

NRINEKVLGKIAESVLRGLSYLHSQRIIHRDIKPSNILLDRQGNVKICDFGVSGEVDNSV

ATTFVGTQYYMAPERIMGKPYSVSCDVWSLGLTLLEVARGSFPYHLQMDSNPLGPIELLS

LILEYQPRLEDIPEDGIFWSDSLKNFISYCLKKNAEERPSPQQMLQHPWCVGQRNIRVNM

QKFVYRLWGDDLD

>tr|C4XYQ2|C4XYQ2_CLAL4 Replication factor C subunit 1 OS=Clavispora lusitaniae (strain ATCC 42720) OX=306902 GN=CLUG_01075 PE=3 SV=1

MEDSEEEFKPNDEEEEEEDDFEPEVVEEEDSEAPVDEDEDLLVQEIEPEPKPRKRKRESA

SPAPKASPAKKQPTATSGSTTRNVAQDSSSGTADEILSQIDDAELPEAMETEGKLNYFAL

KNKQQNVAAPSGNVELPIAAPNCLGGLTIVFTGVLPNLDRDAAESIAKQYGARVTKSISK

KTSLVVIGEEAGPSKVKKIKDFKIKAISEDGFIELLRRMPADGGDGSNAQAAKKKREEEE

RIIREEALAQERLEQKKELERKKAAEKATKAAQGPKQESSRAASPPREIPNSEKLWTVKY

APTSTAQLCGNKGQIQKLKNWLANWFDNAKTNFKNKGADGSGIFRAALISGPPGIGKTSA

AHLVAQELGFDILEKNASDVRSKSLLNSNIKSVLNNTSVVGFFKHQGEKEHQTNERRFCL

IMDEVDGMSSGDHGSAGALSAFCRITNMPMILICNDKSLPKMRTFDRVTLDLPFRRPSEM

EMKSRLLTIAFREKLKLDPTVIGQLVQATGNDIRQIINLMSTVSKTQKNINHDNAKEIAN

SWKKHSMLKPFAIVSQFLGGGIFNPNAHHSLNDKIEMYFNDIDFTPLMIQENYLNSLPSV

STPKEHLERVAAAADAISQSDQINSLIRSSEQQWSLLPFHAVMSTVVPAKEVSGRMTSYP

AFTSWLGQNSKTMKYTRMLQELQYHTRMRTSTSKSELRLDYVPVLSKKLSQPLIDEGEAG

IEEVMDNMDYYYITKEDWDNLIDFGVGPNKGANILAQIPSNVKGAFTRRYNGAAHPMAIL

KTGNSVGGGVGKQKADFEDVVEDDTINDDEEPEVEDNGKFDPKKDKFIKEVKPKKGRKTG

TTGSAASKKSTTSKARASASKNTTSSPASKKRKVTK

>tr|C4XYQ6|C4XYQ6_CLAL4 GPI-anchored wall transfer protein OS=Clavispora lusitaniae (strain ATCC 42720) OX=306902 GN=CLUG_01079 PE=3 SV=1

MLSQFLRKVGCARSFNGTSVHTRRSAFYRCIKGLMSLKELKEKFVSDLSGGPIDEIYAVT

AIALAGFFSHSILKKYVFPNFDVLNSQPAKFLVEFYFDVMLLLQSITIYSSQIGKLYFNA

LFVPIVAMIYVAFTSTKKGPSKQKHKTSTELLGRKSFITAYRAQMMIITNLAILAVDFHA

FPRRFAKVETWGTSLMDMGVGSFVFSMGLANSRAVIKKRAAPSTQKDGYFRLVYNSTVKA

FPVLILGIIRLVSVKSLEYQEHESEYGIHWNFFITLGLLPVVLGLLDPILNFVPRFFVAL

AIGSVYEFALQKTGLTAFILNPANRKENLLTMNKEGICSFFGYLSIFIFGQSLGSFVLTT

RKTPNNLLGMYSLKSKGKKWLTVSTTEGLLIASVITMTIFYFVKESVYTGNISRRLANFP

YVMWVVSYNCAFLLGYDVIEKLVGPISSSILDSINNNGLAIFLSANLLTGLVNMSINTLA

VGHGITYVILTVYALIWTMLAMFLHARKIYIKL

>tr|C4XYQ8|C4XYQ8_CLAL4 Fumarate reductase OS=Clavispora lusitaniae (strain ATCC 42720) OX=306902 GN=CLUG_01081 PE=3 SV=1

MSTNKRIYDAIVVGSGLAGLTASLELQAKGLSVALIEKTDKLGGNSAKASSGISGAPTRF

QDPNEGDSVESFRGDTIKSGKGLSNPMLVDILTKNSADAINWLSDKFNVDLTAVARLGGH

SHSRTHRGSGALPPGFAIVSALSKRLQESSNVEIMKSCRFQGFLKNDGKVDGISVSNSDN

ENLSVNGKNVIIATGGYSADFTDQASLLKRFRPDLVDFPSTNGQQTTGDGQKFAERDLGA

KLLHMDQIQVHPTGFIQLKDSDTIHSKWKFLCGELMRGIGGILLSPINGRRFVNELTTRD

NVTQGILSHCMISKDRPAISIIVIGEEDYSKAKSHIGFYMSQQLIFKGTYADVLNKLETV

IPGHNLSADDIEAGLEEYNRKITSVDDLGREYFGNSVGPEFYFGFITPVLHFSMGGIEIN

ENAQVVTTSGTIEENVFAIGEASGGIHGGNRLGGCALLESVVFGRHVANYITKQ

>tr|C4XYQ9|C4XYQ9_CLAL4 mRNA 3'-end-processing protein OS=Clavispora lusitaniae (strain ATCC 42720) OX=306902 GN=CLUG_01082 PE=3 SV=1

MYNNKIVCKHWLRGLCKKNDHCEFLHEYNLRKMPECLFYSKNGYCTQTPECLYLHIDPQQ

KIPECSQYEKGFCPEGPKCPNRHIRKIMCPLYLTGFCPKGPDCDYSHPKFDNLPPKLRVE

HKRLIKGETKEVEKISYDQPLTEVVRDRR

>tr|C4XYR5|C4XYR5_CLAL4 V-type proton ATPase subunit F OS=Clavispora lusitaniae (strain ATCC 42720) OX=306902 GN=CLUG_01088 PE=3 SV=1

MSSNDAMTKRSLLAVIADEDSVTGLLLAGVGQVSNEPGKESNFLTVVPGKTSVEEVEEAF

ERFTTSRDDIAILLINQHIADLIRHKVDTYTNAFPAILEIPSKDHPYDPEKDSILKKVRR

LFGE

>tr|C4XYR7|C4XYR7_CLAL4 Mitochondrial carrier protein OS=Clavispora lusitaniae (strain ATCC 42720) OX=306902 GN=CLUG_01090 PE=3 SV=1

MVTQESELFSALKRTIKQDSNASLIAGGISGAVSRTIVSPFERAKILLQLQGSEAQKAYQ

GMFATIWKMYKEEGWRGWFRGNTLNCIRIVPYSAVQFAVFEKCKELLVRRKPPGQQTLTD

TDRLIAGSIGGIASVAVTYPLDLVRARITVQTASLAKLNKGKLVEAPGVYATMVNVYRNE

GGLLALYRGIVPTTLGVAPYVAINFALYEYLRDSMDSSTKDFSNPMWKLGAGAFSSFVGG

VLIYPLDLLRKRYQVASMAQGELGFQYRSVAHALQTIFQKEGFFGAYKGLTANLYKIVPS

MAVSWLCYDTLKSAIANW

>tr|C4XYR8|C4XYR8_CLAL4 4-aminobutyrate aminotransferase OS=Clavispora lusitaniae (strain ATCC 42720) OX=306902 GN=CLUG_01091 PE=3 SV=1

MTEIRPKLSVMLQNSDLMILNITSQLQYHNLLLTFSFFTLLTSFFFEPNFYAWSALSLPN

RNALQLALFCRCAKYFRYRMPNSVECAKLLDWPHYIKQWLDAFFHRLPPTMSICEKYFPE

EPKEPKVATSFPGPKSQDAIKSLGTVFDSRPVYFVADYEKSVGNYIVDVDGNTYLDVYAQ

IASIPLGYNNPALIEAAKSDKMVRAIVDRPALGNFPGKDTEEIISELLKVAPKGQDKVWS

GLSGADANELAFKAAFMWYQAKKRGYTASFTEEENRSVMENAAPGSPDLAILSFKRAFHG

RLFASASTTCSKPVHKLDLPSFKWPKAEFPSYAYPLDANAEVNKKEDERCLVMVEEYLKH

WKSPIAAVLIEPIQSEGGDNHASAFFFQGLRDLTLKYGALLIVDEVQTGVGATGKMWAHE

HFNLSPAPDMVTFSKKFQSAGYYFHDPEIIPNQAYRQFNTWCGDPARMILAGAIGSEVVK

HNLAEHASRVGDYLYGKLESLFSKYSELVTDLRGKNRATFIAWSFKKPELRNKFLVDMKS

VGINIGGCAEDSVRLRPTLVFEEKHADILVAGIDKILSGY

>sp|C4XYS2|AIM41_CLAL4 Altered inheritance of mitochondria protein 41, mitochondrial OS=Clavispora lusitaniae (strain ATCC 42720) OX=306902 GN=AIM41 PE=3 SV=1

MFASLRRFQATPAYGALLSSLKVDLKKAMIAKNNMEKTTIRSILSTIKNNEIDGGAQTEF

ELSKVLGKMVKQRVDSAREFQQQKRDDLAEIESKESEIIKKYVESLPVASDEEIKEKLTH

FLNEIKANDANTHVGAIFKQINDDLASSWGAAPSVIKSMVPSLYKDIFKK

>tr|C4XYS3|C4XYS3_CLAL4 Dihydrofolate reductase OS=Clavispora lusitaniae (strain ATCC 42720) OX=306902 GN=CLUG_01096 PE=3 SV=1

MFSCEPGRSLFISHGCLIISARVSSTWLHHIHMIKSLKPVTSVIVAALSPKYGIGAQGKL

PWRLKQEMKYFKDVTSAARAGSINAVVMGRKTWESIPKKFRPLPNRLNIVLSRSFSNEEK

DGVLYFNSIDSIMSNLAQSNYWYHDKPIDKIFIIGGAEIYNSVMKGDLVDNLLVTNIRYV

GNPEAEPVLDTFLDWDMSLWEQSNVSRIREFSDVEFEEGIIKEGDYEYEYTMWERRK

>tr|C4XYS6|C4XYS6_CLAL4 histone acetyltransferase OS=Clavispora lusitaniae (strain ATCC 42720) OX=306902 GN=CLUG_01099 PE=4 SV=1

MEFSPYLEGLHHVVHLRTIAYQVKPLIVSGPPTLKSKHLLYVVEEASQCLVLAVEVYVYF

TCDNGVLHRHFFVSKADTSGLGTRRLSAAGIIAQFIKYLVSLAPDAYLANVSWRKQHQTE

TTASAKKSEFSVVNTLMDLSHQLKSDPAFYDSISFYHARDLPLSRTSAHSTDKRWNYNSI

STSLSLFTRSANAYIFPKSEKNPGKHVADGNGLLKWWISVLHKSLGDCWDCKADIPGSDP
[truncated: 3,259,170 more chars]
